# miRNAome expression profiles in the gonads of adult *Melopsittacus undulatus*

**DOI:** 10.7717/peerj.4615

**Published:** 2018-04-09

**Authors:** Lan Jiang, Qingqing Wang, Jue Yu, Vinita Gowda, Gabriel Johnson, Jianke Yang, Xianzhao Kan, Xiaojun Yang

**Affiliations:** 1 State Key Laboratory of Genetic Resources and Evolution, Kunming Institute of Zoology, Chinese Academy of Sciences, Kunming, China; 2 The Institute of Bioinformatics, College of Life Sciences, Anhui Normal University, Wuhu, China; 3 The Provincial Key Laboratory of the Conservation and Exploitation Research of Biological Resources in Anhui, Wuhu, China; 4 College of Foreign Studies, Anhui Normal University, Wuhu, China; 5 Department of Biological Sciences, Indian Institute of Science Education and Research, Bhopal, Madhya Pradesh, India; 6 Department of Botany, National Museum of Natural History, Smithsonian Institution, Washington, District of Columbia, USA; 7 School of Basic Medicine, Wannan Medical College, Wuhu, China

**Keywords:** Gonads, Sex-biased, miRNA, Adult, *Melopsittacus undulatus*

## Abstract

The budgerigar (*Melopsittacus undulatus*) is one of the most widely studied parrot species, serving as an excellent animal model for behavior and neuroscience research. Until recently, it was unknown how sexual differences in the behavior, physiology, and development of organisms are regulated by differential gene expression. MicroRNAs (miRNAs) are endogenous short non-coding RNA molecules that can post-transcriptionally regulate gene expression and play a critical role in gonadal differentiation as well as early development of animals. However, very little is known about the role gonadal miRNAs play in the early development of birds. Research on the sex-biased expression of miRNAs in avian gonads are limited, and little is known about *M. undulatus*. In the current study, we sequenced two small non-coding RNA libraries made from the gonads of adult male and female budgerigars using Illumina paired-end sequencing technology. We obtained 254 known and 141 novel miRNAs, and randomly validated five miRNAs. Of these, three miRNAs were differentially expressed miRNAs and 18 miRNAs involved in sexual differentiation as determined by functional analysis with GO annotation and KEGG pathway analysis. In conclusion, this work is the first report of sex-biased miRNAs expression in the budgerigar, and provides additional sequences to the avian miRNAome database which will foster further functional genomic research.

## Introduction

MicroRNAs (miRNAs) are all non-coding RNAs that play vital role in post-transcriptional regulation of various animals and plants (Bartel, 2009). Almost thirty thousand entries have been released in the latest miRBase database (v21) (Kozomara & Griffiths-Jones, 2014) where each miRNA can target hundreds of messenger RNAs (mRNAs) in diverse binding sites, resulting in the enhancement or suppression of gene expression (Lim et al., 2005). The importance of miRNA is evident from their evolutionary conservation and by the various biological processes in which they are involved, including development and physiology (Friedman & Burge, 2014; Skalsky et al., 2014). Animal miRNA are involved in neuronal cell fate, cell proliferation and differentiation, metabolism, aging, apoptosis and organ morphogenesis, suggest that miRNAs are particularly critical in the development, health, and aging of animals (Ameres & Zamore, 2013).

Sexual dimorphism is a universal trait among animals where morphological and behavioral differences between genders play an important role in their sexual selection. Gonads are the principal reproductive organs that are involved in sexual differentiation wherein they are involved in the production of sex hormones and gametes. Although sexual dimorphism is most visual in birds (example peacock and peahen, hummingbirds, songbirds, paradise fly-catcher), the miRNAs involved in gender differentiation among Psittaciformes are not known. Most recently, few miRNAs that are involved in gender differentiation have been reported in many non-avian species, including human (Ali et al., 2016), fruit flies (Marco, 2014), sheep (Torley et al., 2011), pigs (Mai et al., 2016), marine bivalves (Rosani, Pallavicini & Venier, 2016), *Schistosoma mansoni* (Marco et al., 2013) and the yellow catfish (Jing et al., 2014). In birds, previous miRNA studies have mainly focused on miRNAs that regulate germ cells in various stages of chicken embryo development and breeder cock testes (Table 1). While the latest release (v21) of the miRBase database contains a total of 1328 mature miRNAs in birds, these sequences are based exclusively on domesticated poultry species and do not represent the vast diversity of Class Aves.

**Table 1:** Previous studies on the avian miRNAs.

No.	miRNAs samples	Reference
1	Identified 84 miRNAs in 0.5–5-day-old chicken embryos	Darnell et al. (2006)
2	Discovered 449 new miRNAs in chicken embryos	Glazov et al. (2008)
3	Detected 160 miRNAs (14 novel) in the embryonic chicken	Hicks, Tembhurne & Liu (2008)
4	Found 663 miRNAs in breeder cock testes	Wu et al. (2016)
5	Suggested that the MIR202* of chicken might play a critical role in regulating testicular development	Bannister et al. (2009)
6	Observed that miR-101, miR-31 and miR-202-5p of chicken had roles in testicular and ovarian development	Cutting et al. (2012)
7	Found 55 differentially expressed miRNAs between the ovaries of laying and non-laying ducks	Yu et al. (2013)
8	Detected 353 differentially expressed miRNAs between the ovaries of laying and broody geese	Xu et al. (2014)
9	Identified 93 differentially expressed miRNAs between the ovaries of mature and immature chickens	Kang et al. (2013)

Parrots, comprising the parakeets, cockatiels, macaws and cockatoos, are members of the Psittaciformes order. Currently, within the order Psittaciformes at least 397 species are recognized across 94 genera (Gill & Donsker, 2017). This order is classified into four families: Strigopidae (New Zealand parrots), Cacatuidae (Cockatoos), Psittacidae (African & New world parrots), and Psittaculidae (Old world parrots). Parrots are generally recognized as the most remarkable intelligent animals along with corvids, chimps, dolphins, and humans. The budgerigar (*Melopsittacus undulatus*) is a small parrot from Australia and a popular domestic pet throughout the world (Del Hoyo et al., 2015). It is also the most widely studied parrot species, frequently used as a model organism for behavioral studies, feather pigmentation and neuroscience, specifically in the fields of behavioral and neurosciences due to their easy availability, small size and easy breeding in controlled conditions. Budgerigars were also well-fitted for genetic mapping experiments, the polyketide synthase was abolished by the R644W substitution, which blocked the synthesis of yellow pigmentation in the budgerigars (Cooke et al., 2017). Several studies have shown that vocal learning in adult budgerigars is gender-biased. In males, right-sided dominance of molecular neuronal activation was found in answer to mate calls in the CMM male (Eda-Fujiwara et al., 2016), several studies found that vocal learning in adult budgerigars is sex-biased (Striedter et al., 2003). These differences in learning have been well documented at various developmental stages in males and females as well (Hoeschele & Bowling, 2016). Nevertheless, the biological functions of gonadal miRNAs in budgerigars are largely unknown and the sexual differential expression (DE) profile of gonadal miRNAs in budgerigars has not been reported.

In the current study, miRNA expression profiles were collected in two groups of budgerigars (male testes and female ovaries) using Illumina paired-end sequencing technology. Based on the new data generated for *M. undulatus* and the existing information from miRBase, we achieved the following goals: (1) identification of known miRNAs and novel miRNAs in *M. undulatus* gonads; (2) nucleotide bias in these miRNAs; (3) identification of those that are differentially expressed.

## Methods

### Ethics statement

Animals in the current study were authorized by the Ethics Committee of Anhui Normal University (Anhui, China) with authorization number #20150612.

### Tissue collection, RNA preparation and sequencing

Six adult 1.5-year-old budgerigars (three males and three females) were obtained from Wuhu (31°33′N, 118°37′E, southeast of China) in 2015. Total RNAs from six gonadal samples were extracted using TRIzol reagent (Invitrogen, Carlsbad, CA, USA). The quality and integrity of the RNAs were examined using an ND-8000 spectrophotometer (Nanodrop Technologies, Wilmington, DE, USA) and a 2100-Bioanalyzer (Agilent Technologies, Santa Clara, CA, USA), which having a RNA integrity number >7.0 (Fig. S1). To reduce the individual differences between samples, two RNA pools for deep sequencing were prepared using equal amounts of the extracted RNA from three ovaries and three testes. The Truseq Small RNA Sample Preparation Kit (Illumina, San Diego, CA, USA) was used to isolate small RNA (sRNA) to construct a miRNA library according to manufacturer’s specifications. The resulting cDNA products were sequenced by the Illumina Hiseq 2500 sequencer (Illumina Inc, San Diego, CA, USA). The resulting sequence data have been submitted to the Short Read Archive at NCBI and are available through accession SRR5664259 and SRR5664260.

### Analyses of sequencing data

The low-quality reads and adaptor contamination were identified by FastQC v0.11.5 (https://www.bioinformatics.babraham.ac.uk/projects/fastqc/) using the PHRED algorithm and removed using cutadapt v1.14 (Martin, 2011), resulting in fragments corresponding to RNAs of 14-41 nt in length. Subsequently, analyses of the length distribution and clustering of sRNA reads revealed the characteristics of the sRNAs. Next, the resulting sRNA sequences were aligned against expressed sequence tags (ESTs) stored in NCBI (https://blast.ncbi.nlm.nih.gov), Rfam 11.0 (Burge et al., 2012), and RepBase (Jurka et al., 2005). The aligned sequences were removed, and the small RNAs were annotated into different categories, such as rRNA, tRNA, small nuclear RNA (snRNA), and small nucleolus RNA (snoRNA), and reads mapped to those were removed as well. The Bowtie v1.2.1.1 (Langmead et al., 2009) and the SOAP2 (Li et al., 2009b) software were used, allowing for ≤1 mismatch, to map with the budgerigar genome (v6.3) (Accession: NW_004848282.1) (Ganapathy et al., 2014). Conserved miRNAs were identified in the budgerigar by matching the unannotated data to precursor and mature miRNA sequences in miRBase v21. Venn diagram drawn using BioVenn (Hulsen, Vlieg & Alkema, 2008).

### Prediction of potential miRNAs and analysis of miRNA function

miRDeep2 software was used to discover known and novel miRNAs from sequence data (Friedländer et al., 2012). The Dicer-binding sites and the free energies were combined in evaluating these candidate miRNAs to assign them score numbers that correspond to the reliability if the miRDeep2 predictions (Gong et al., 2017), then, the miRDeep2 predictions were used to map the remaining results with the genome (mismatch < 1). The criteria we used to distinguish miRNAs from other classes of small RNAs amounted to a score of total reads >5, and a true positive prediction ≥90%. Mfold program based on the free energy minimization, was used to predict their propensity to form hairpin loops as potential pre-miRNAs (Zuker, 2003).

To investigate differentially expressed miRNAs, each library was normalized to transcripts per million (TPM) by DESeq (Anders & Huber, 2010). Three miRNAs that were differentially expressed between ovaries and testes were determined to be statistically significant using *p*-value ≤0.05 (*q* < 0.01) and a minimum fold change of 2 (Benjamini & Hochberg, 1995). Samtools (Li et al., 2009a) were used to extract 3UTR sequence. Using 3UTR regions of budgerigar for targeting. The potential target genes for the seed sequence of three significantly differentially expressed miRNAs and eighteen miRNAs were predicted using miRanda (score ≥150, MFE (minimum free energy) <−20 Kcal/mol) (Enright et al., 2004), seedVicious (Marco, 2017) and TargetScan (Yatsenko et al., 2005) software. The basic functional targets were classified using Gene Ontology (GO) annotations (Ashburner et al., 2000) and the KEGG pathway database (Kanehisa et al., 2017), false discovery rate (FDR) <0.05 was defined as statistical significance.

### Quantitative PCR validation

Five randomly selected miRNAs were used to validate their expression profiles by real-time quantitative PCR (Q-PCR). All reactions were duplicated three times to validate the reliability of the predicted miRNAs. Primers to detect miRNAs were designed using miRprimer2, as previously described (Busk, 2014). The cDNA was synthesized using Rayscript cDNA Synthesis KIT (GENEray, GK8030). Based on the protocol of the AceQ TM Q-PCR Probe Master Mix (Vazyme, Q112-02), the PCR reaction and temperature conditions were performed in a two-step Q-PCR method using the ABI 7500 qPCR instruments (Applied Biosystems Inc., Foster City, CA, USA). The housekeeping gene 5S ribosomal RNA was used as an internal normalization control. The Q-PCR reaction mixture (20 µL) contained 5 µL cDNA product, 0.5 µL primer forward and 0.5 µL primer reverse, 10 µL Taqman Mix with 0.4 µL ROX, and 3.6 µL ddH_2_O. PCR cycles were as follows: 2 min at 95 °C, followed by 40 cycles of 10 s at 95 °C, and 49s at 60 °C. All reactions were duplicated three times, and after amplification, melting curves were analyzed for all reactions. We calculated relative quantification expression results by the 2^−ΔΔCT^ method (Livak & Schmittgen, 2001). Analyses were performed in R (http://www.R-project.org/) using the *t*-test function with a *p*-value ≤0.05, which was used to detect the significantly differentially expressed miRNAs between ovaries and testes.

## Results

### Small RNA sequence profile

**Figure 1:**
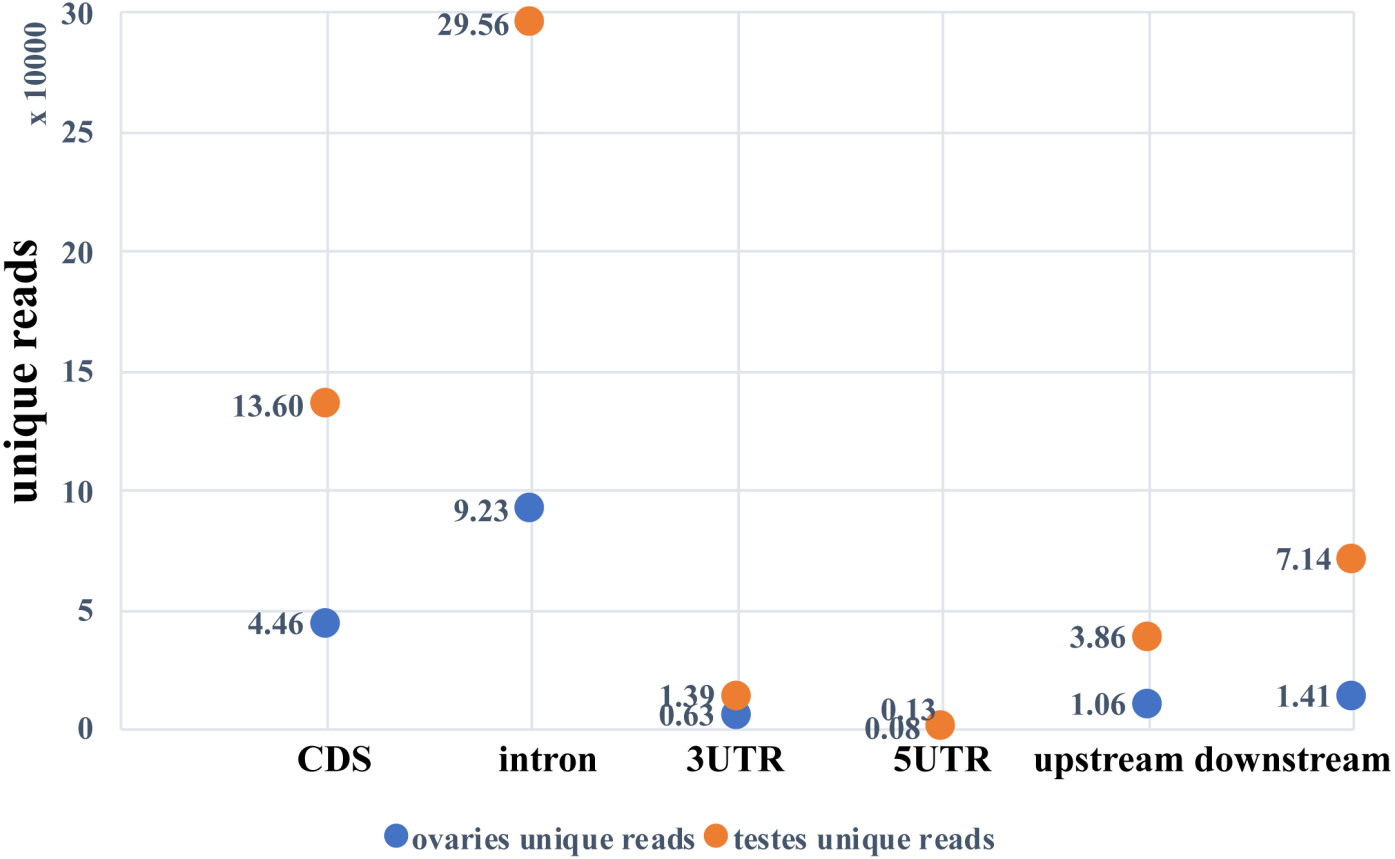
Unique reads mapped to the genome.

**Figure 2:**
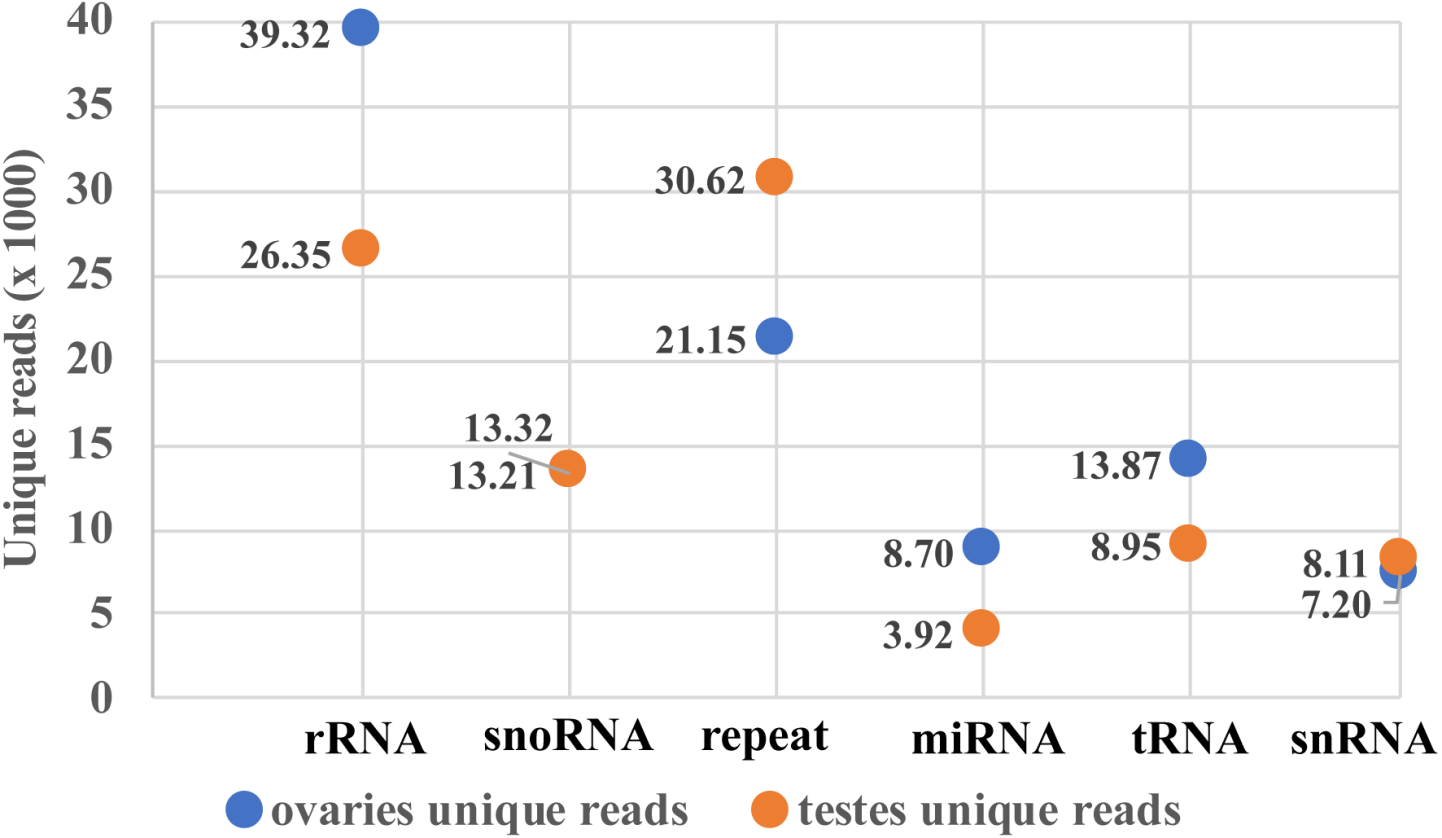
Mappable unique reads about small RNA classification.

Two small RNA libraries were sequenced using Illumina Hiseq 2500 in a single lane. A total of 9,191,836 and 6,383,927 raw reads were obtained from the ovarian and the testicular samples, respectively. After filtering for adaptors and low quality reads, 82.03% and 84.43% of the total reads were recovered (Table 2). The proportion of clean and unique reads that matched to the *M. undulatus* genome were 6,927,475 (91.88%) / 448,234 (78.34%) and 4,646,370 (86.21%) / 2,007,263 (84.68%) in the ovarian and testicular libraries, respectively. Fewer ovarian reads mapped to CDS, intron, and 3UTR upstream and downstream, compared to those of the testis; however, more ovarian reads mapped to 5UTR sequences (Fig. 1). We also mapped unique reads to the Rfam, RepBase, EST database and miRBase (v21). The sequences that mapped perfectly to the pre-miRNAs and mature miRNAs in miRBase (v21), were considered to be mappable reads. To avoid influencing miRNA identification, the unmappable reads were removed by searching against noncoding RNAs (tRNA, rRNA, snoRNA, and snRNA) deposited in the RepBase and Rfam databases (Fig. 2). Finally, 7,540,057 cleaned sequences of ovaries representing 572,201 unique reads and 5,389,781 cleaned sequences of testes representing 2,370,340 unique reads were used for subsequent analysis.

**Table 2:** Raw reads and clean reads in *M. undulatus gonads*.

Category	Total reads			
	Ovaries		Testes	
Raw reads	9,191,836	100%	6,383,927	100%
Clean reads	7,540,057	82.03%	5,389,781	84.43%

### Identification of new members of known miRNAs and novel miRNAs

The unique reads from the two deep-sequencing libraries were mapped to the budgerigar genome. The secondary stable hairpin structures were predicted for identification of new miRNAs (see Fig. S2). The abundances of known miRNAs that mapped to the budgerigar genome and miRBase v21. Predicted pre-miRNAs were analyzed and represent 101 miRNA families, including 254 miRNAs previously identified in Aves (see Table S1). Differences in expression frequencies among miRNAs reads were detected.

MiRNA family represents sequences that evolved from a common ancestor. The let-7 family is a conserved miRNA family, both in sequence and function and plays a vital role in animal development. To date, more than thirteen types of let-7 family miRNAs have been identified in animals, and the seven detected in this study, mun-let-7, mun-let-7a, mun-let-7c, mun-let-7e, mun-let-7f, mun-let-7g, mun-let-7i have not been previously described. These members of let-7 family in budgerigar are highly differentially expressed.

**Figure 3:**
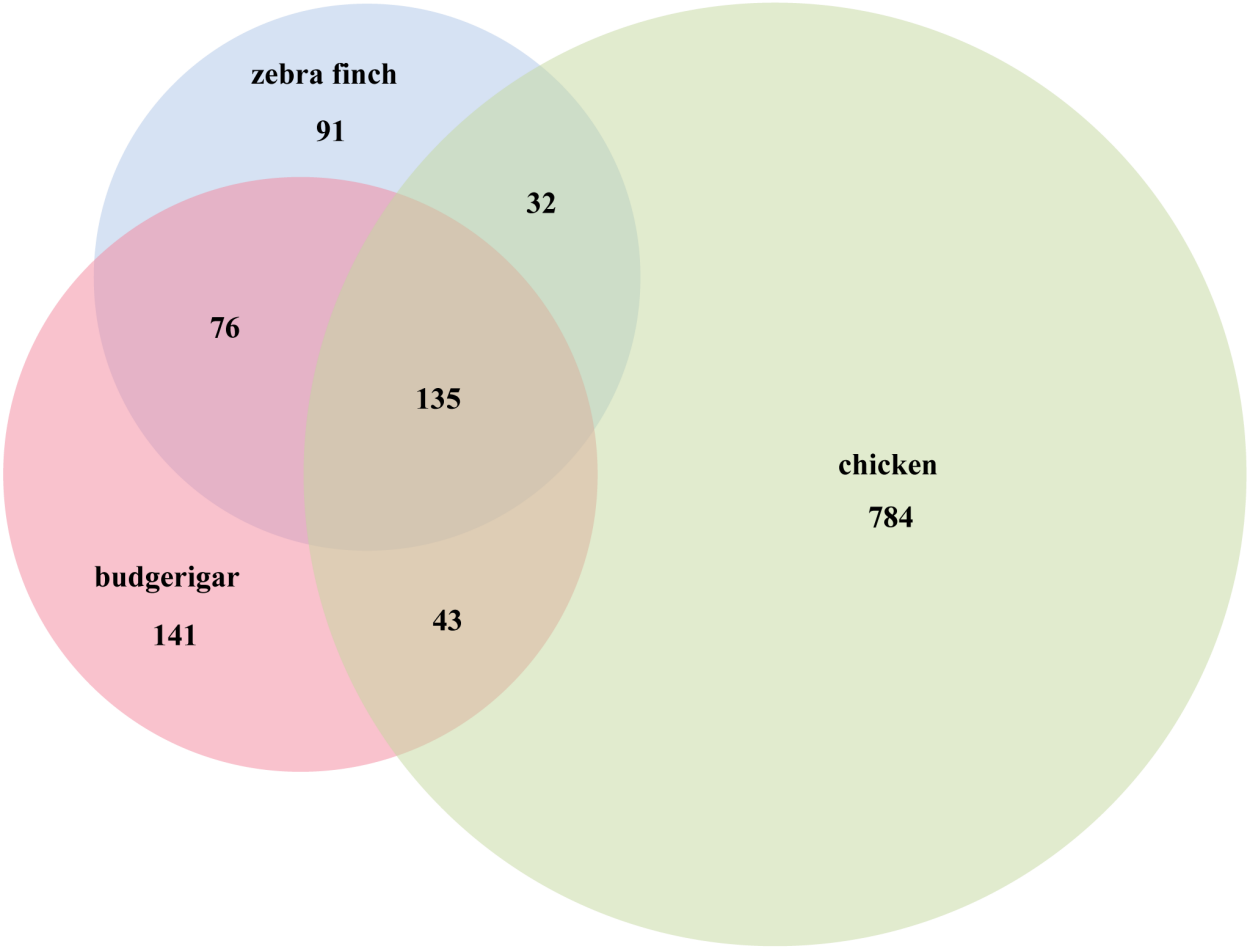
Budgerigar miRNAs compared with zebra finch and chicken in miRBase v21.

We identified several putative novel miRNAs using the reads that did not map to known miRNAs. A total of 141 novel miRNA candidates were predicted (Table S2). In total, 395 miRNAs were identified including 254 known and 141 novel miRNAs from the two libraries. Furthermore, 282 of these were co-expressed in male and female gonads, while 113 were gender-specific: 88 female and 25 male (Table S3). Compared with the Aves database (zebra finch and chicken) in miRBase v21, we used Venn diagrams to compare three species (zebra finch, chicken and budgerigar), 135 miRNAs were found co-expressed in the three species, 76 miRNAs were found in zebra finch and in budgerigar, and 43 miRNAs were found in chicken and in budgerigar (Fig. 3).

### miRNA nucleotide bias

**Figure 4:**
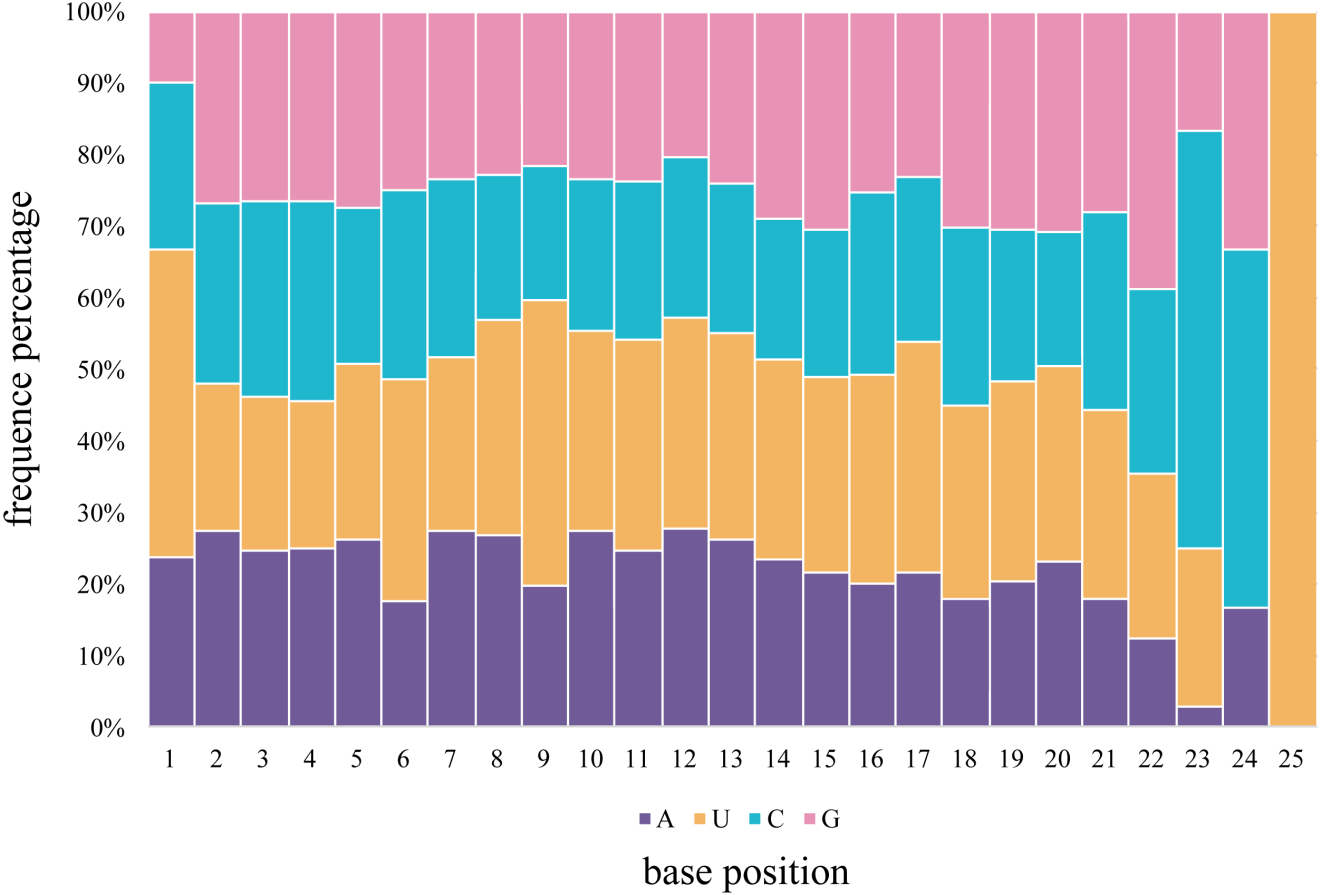
The percentage distribution of base composition at each position of *M. undulatus* miRNAs in all combined tissues.

The first 5′ end nucleotide of the budgerigar miRNAs, of any length, was not frequently uridine, as seen in 42.8% of gonadal samples, various nucleotides are detected at the 5′ end of miRNA sequences. In general, U is the predominant nucleotide at 5′ end of all miRNAs of all lengths. Adenine accounts for 23.8%, Cytosine accounts for 23.3%, Guanidine accounts for 10.1% across all miRNA nucleotide positions. Averaged across both gonad samples, 66.6% of the nucleotides consisted of A+U in the first bias (Fig. 4). In the seed region of the miRNAs, i.e., the second to eighth nucleotide positions, A-U was the most frequently observed pair.

### Differentially expressed and sex-biased miRNAs identification

Notably, three of the miRNAs (mun-215-5p, mun-novel24-5p and mun-novel10-3p) were expressed differentially between the gonadal samples with statistical significance, differentially expressed miRNAs were detected using both the absolute value of fold change >2 and *q*-value <0.01 (Table 3). Consequently, 11 of these were among the 141 novel miRNAs found in the testes but not found in the ovaries. Likewise, mun-miR-203-5p was expressed in the testes, exclusively. Of the remaining detected miRNAs expressed in both gonads. For instance, mun-miR-194-5p and mun-miR-375-3p in ovaries, and mun-miR-2954-3p, mun-novel101-3p, mun-novel102-3p and mun-novel8-3p in testes were expressed more than average. Due to these, 21 miRNAs were selected for following functional analysis.

**Table 3:** Three significant expression miRNAs and eighteen miRNAs in *M. undulatus* gonads.

miRNA name	Ovaries	Testes	Ovaries_TPM	Testes_TPM	FoldChange_Log2	*P*value	*Q*value
mun-miR-215-5p	61,621	81	18,361.36013	230.9738828	−6.312799391	1.05E–06	0.000494547
mun-novel24-3p	0	943	0	2,688.992241	Inf	1.99E–06	0.000494547
mun-novel10-3p	0	489	0	1,394.397885	Inf	3.83E–05	0.006351634
mun-novel15-3p	0	152	0	433.4324715	Inf	0.001679481	0.129097846
mun-novel68-3p	0	97	0	276.5983535	Inf	0.003671809	0.202765425
mun-novel31-3p	0	27	0	76.99129428	Inf	0.005862203	0.224116531
mun-novel45-3p	0	27	0	76.99129428	Inf	0.005862203	0.224116531
mun-novel72-3p	0	30	0	85.54588253	Inf	0.005261171	0.224116531
mun-novel29-3p	0	30	0	85.54588253	Inf	0.005261171	0.224116531
mun-novel46-3p	0	23	0	65.58517661	Inf	0.007334974	0.260391575
mun-miR-203-5p	0	16	0	45.62447068	Inf	0.014360624	0.419837066
mun-novel36-3p	0	12	0	34.21835301	Inf	0.02575755	0.609595344
mun-novel72-5p	0	10	0	28.51529418	Inf	0.037043893	0.836750495
mun-novel66-3p	0	9	0	25.66376476	Inf	0.045293686	0.865806236
mun-novel7-3p	0	9	0	25.66376476	Inf	0.045293686	0.865806236
mun-novel102-3p	1	194	0.297972446	553.1967071	10.85839792	0.002078034	0.129097846
mun-novel8-3p	2	59	0.595944893	168.2402357	8.14112813	0.015983738	0.427715185
mun-novel101-3p	9	202	2.681752018	576.0089424	7.746771562	0.013714368	0.419837066
mun-miR-2954-3p	218	803	64.95799333	2,289.778122	5.139556933	0.040127921	0.836750495
mun-miR-375-3p	2,228	11	663.8826108	31.3668236	−4.403616818	0.040406463	0.836750495
mun-miR-194-5p	2,669	5	795.2884597	14.25764709	−5.801670415	0.009804135	0.324843664

### Quantitative-PCR analysis of gonadal miRNAs

**Figure 5:**
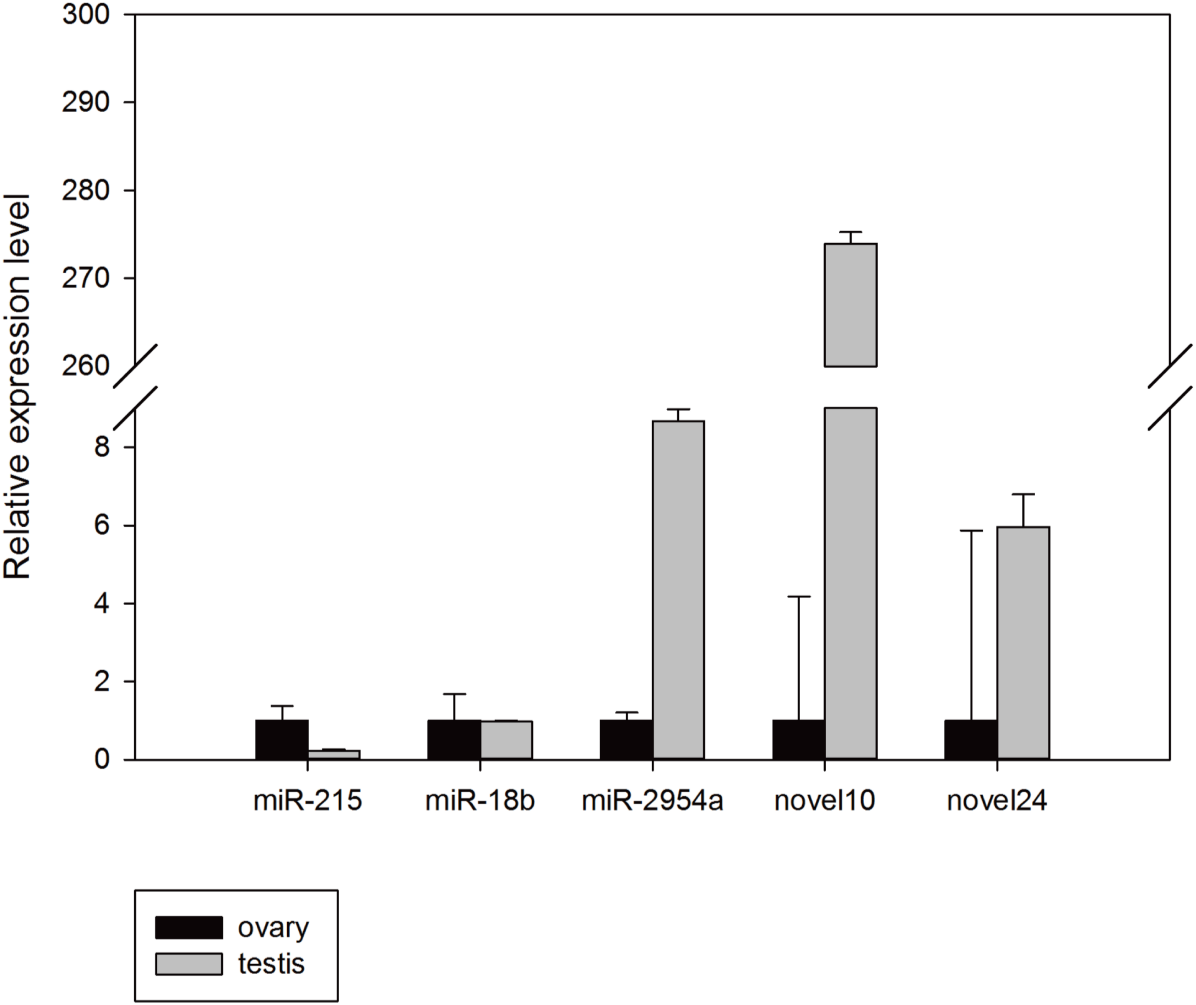
Random validation of miRNAs with expression using Q-PCR.

To confirm the reliability of the miRNA-seq data and bioinformatic predictions, stem-loop Q-PCR assays were conducted. Mun-miR-215-5p, mun-miR-18b-5p, mun-miR-2954-3p, mun-novel10-5p, and mun-novel24-3p were randomly selected for validation. All results were concordant with their relative expression trends for our miRNA-seq analyses basically, and confirmed that these miRNAs exist in the budgerigar. The expression level of mun-miR-18b-5p, mun-miR-2954-3p, mun-novel10-5p, and mun-novel24-3p in the male samples were higher than in the female samples, whereas mun-miR-215-5p expression was higher in the ovaries. There were a minor differences between the miRNA-seq analysis and Q-PCR assays results. For example, mun-novel10-5p and mun-novel24-3p expression were not detected in the sRNA sequence data of the ovaries (Table 3), but weak expression was detected in the validation experiments (Fig. 5).

### Putative target prediction for known and novel miRNAs

The putative target genes for these 21 miRNAs were predicted by miRanda, seedVicious and Targetscan. One thousand, five hundred and sixty-seven genes were determined to be feasible targets and had complete complementarity to the seed sequence of 21 miRNAs. These predictions suggest that a single miRNA might target more than one mRNA, such as mun-miR-2954-3p, which is predicted to target 129 budgerigar genes (Table S4). Similarly, one gene can be controlled by one or more miRNAs. For instance, mun-novel102-3p could target FOXG1, and FOXP1 has three miRNAs target sites (mun-novel45-3p, mun-novel102-3p and mun-miR-2954-3p), and mun-novel7-3p, mun-novel36-3p, mun-novel102-3p and mun-miR-203-3p could target FOXP2 (Table S4). The miRNAs identified in this study can target multiple transcription factor genes, such as GATA4, GTF2E1, GTF3C3, JUN, LZTFL1, MBTPS1, MTF1, NFYA, NFYC, PBX3, RFX4, and SOX10. Moreover, computational analysis of the 21 miRNA sequences with known, functional miRNA–mRNA regulatory modules suggested these miRNAs may target genes encoding KPNA3, PKP4, CHN1, SMG7, FAM53A, and calmodulin, which are included in a series of important physiological processes and metabolic networks.

### GO annotation and KEGG pathway analyses

**Figure 6:**
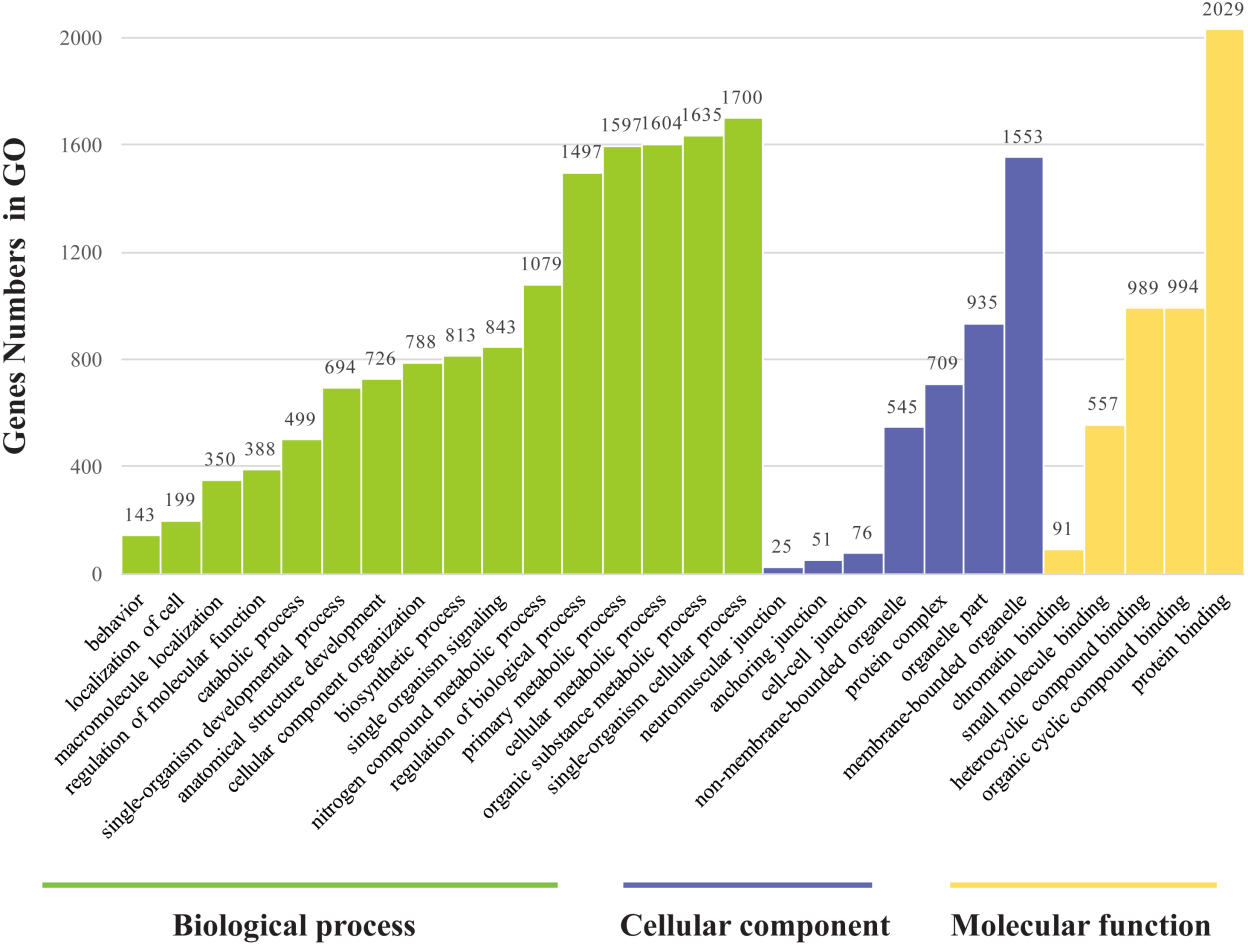
Gene ontology classification annotated by gene2go for target genes of differentially expressed miRNAs. The figure shows partial GO enrichment for the predicted target genes in ontologies of molecular function, cellular component, biological processes.

The genes found to be potentially regulated by miRNAs from this study were annotated using GO annotation and KEGG pathway analyses. Gene Ontogeny annotations were classified as cellular component, biological process and molecular function, using GO rank 2 with *p*-value ≤ 0.05 (FDR < 0.05). We found that many of the miRNAs detected in this study were involved in the organogenesis. Seven subcategories within “cellular components” were found, with “cell part” and “membrane-bounded organelle” being most represented. Sixteen subcategories of “biological processes” were also identified, with “single-organism cellular process” being most abundant. Furthermore, many genes were assigned to five subcategories in “molecular function,” with the largest proportion in “protein binding” (Fig. 6). Notably, mun-miR-215-5p, which demonstrated significantly biased expression in the ovaries, is likely a primary modulator of a protein complex involved in signaling and/or catalytic enzyme activity, like RABGAP1, PRR5, FGF13, SDCBP, TIAL1, PRDX4 (Table S4). From the GO term, we found nine GO annotations were related to female, which the detected miRNAs could regulated some genes, like FKBP4, C1QBP, VMP1, AKT1, BMPR1B, ADAMTS1, PHB2, IF2B2, MED1, DACH2, CHD7, AK8, TBP, TAF4, DACH1, LHX9, TYRO3, KIT, EIF2B2, DACH2 and PDGFRA, thirteen GO terms were found related to male and six GO annotations wete detected as gonadal function as well (Table S5). In addition, FOXP1 (mun-miR-2954-3p, mun-novel102-3p and mun-novel45-3p) and FOXP2 (mun-miR-203-5p, mun-novel102-3p, mun-novel36-3p and mun-novel7-3p) were detected to regulate vocal learning, moreover, VDAC1, CNTN2, GRIN1, FOXP1, FOXP2, HIF1A, VDAC3, CAMK4, PRKAR1B, ITGA8, GRIA1, PLK2,HMGCR, KCNAB1, CHST10, RELN, FGF13, ABI1, APP, SRF, LIS1, AK8, CTNND2 and ATAD1 were illustrated to regulate learning ability (Table S5). The KEGG pathway analysis demonstrated that the target genes were related to significantly expressed miRNAs. According to the KEGG pathway analysis (*FDR* < 0.05), three pathways were significantly enriched, such as the cell communication (*FDR* = 0.00317745), excretory system (*FDR* = 0.00317745) and signal transduction (FDR = 0.00446146) (Table S6).

## Discussion

MiRNA research is needed in more phylogenetically disparate avian species to obtain a more accurate concept of the miRNAome. Furthermore, some important avian miRNAs involved in regulating gonadal sex differentiation and development were demonstrated (Table 1). However, avian studies of sex-biased miRNA expression between ovaries and testes are limited. The role of non-coding RNAs in the gonads is an area of active research. Here we used Illumina sequencing to investigate the differentially expressed miRNAs in the male and female gonads of budgerigars. We observed numerous miRNA families in our data that may potentially work as key regulators of gene expression. For example, the let-7 family has been shown to function as a heterochronic switch, and loss of these could cause periods of cell fate reiteration in adults. In contrast, increasing the gene dosage would led to premature expression in adult fates (Reinhart et al., 2000). Consequently, the let-7 family miRNAs are considered highly conserved in Animalia (Hertel et al., 2012). Let-7 and miR-125 are associated with polycistronic transcripts and work as two key regulators of development in Bilateria.

MiR2954, which is known in chicken (Garcia-Riart et al., 2017; Liu et al., 2017) and zebra finch (Lin, Balakrishnan & Clayton, 2014), is an bird-specific gene (absent in the mammalian lineage) and is encoded on the Z chromosome which is known to result in its higher expression in males than females (Lin, Balakrishnan & Clayton, 2014; Luo et al., 2012). It has been proposed that this might affect the neurogenomic mechanisms that lead to sexually dimorphic bird song habituation (Lin, Balakrishnan & Clayton, 2014). Based on bioinformatical and experimental analysis from chicken and zebra finch, miR-2954-3p is male-biased and Z-linked miRNA, which targets across a range of bird species, could help the study field about the evolutionary dynamics of partial dosage compensation and the genetic architecture underlying gonadal characteristics (Warnefors et al., 2017). The bioinformatical prediction and the qPCR validation in the present study has confirmed that there is a male-biased expression of miR2954 in *M. undulatus*, further corroborating its involvement in sexual dimorphism. Putative genes targeted by miR2954 include a TLE4 transcription factor family, which might be associated with nervous system function, including Ca^2+^/ calmodulin-dependent protein kinase IV (CaMKIV), SCAMP1, and SMARCA2. Mun-miR-2954 is also related to development, environmental adaptation, the nervous system, signaling molecules and interaction, and substance dependence as indicated in the KEGG analysis. Previous study in zebra finch has elaborated miR2954 could target FOXP2 to regulate vocal learning and detected higher male expression in many tissues (Fu et al., 2014), however, we haven’t detected mun-miR2954-3p target FOXP2, whereas mun-miR-203-3p, mun-novel102-3p, mun-novel36-3p and mun-novel7-3p might regulate FOXP2, mun-miR-2954-3p, mun-novel102-3p and mun-novel45-3p might regulate FOXP1, which related to vocal learning in the budgerigars and we also measured higher male expression in gonads.

Further investigations of GO analysis, miRNA in current study might regulate several sex-related genes. For instance, FKBP4 was considered to be markers of hypospermatogenic testis (D’Aurora et al., 2017), we found higher male expression of mun-novel7-3p and mun-novel72-3p could target FKBP4. mun-miR-194-5p and mun-novel31-3p might regulate SALL1 which the transcriptional regulators of adipose-specific sex-different genes (Karastergiou & Fried, 2017). LHX9 is needed for ovarian function (Workman, 2017), mun-novel72-3p and mun-novel8-3p could regulate it. Mun-miR-215-5p which significantly expressed in ovaries could target PRDX4 (sex-linked gene) (Tippabathani et al., 2017). In mice gonadal development, miR-181a suppressed granulosa cell proliferation by targeting ACVR2A, we detected that mun-novel7-3p could regulated ACVR2A as well (Zhang et al., 2013). Currently, mun-novel7-3p could also target AKT1 (with prior report for sex differences (Seney et al., 2013)). And mun-novel31-3p could target RNF2 (associated with regulation of genetic imprinting (Li, Zhang & Wu, 2017)). mun-novel68-3p might regulate HSF2 which related to sex-determining (Literman et al., 2018), mun-miR-2954-3p and mun-novel72-3p could target KITLG (sex development) (Hersmus et al., 2017) (Table S4). Of all the miRNAs tested, we found three gonad-enriched miRNAs (mun-miR-215-5p, mun-novel10-5p and mun-novel24-3p). In the present study, the presence of these miRNAs in the gonads suggests that they might serve a similar function in the budgerigar. These miRNAs may produce sex-specific responses to potential biological mechanisms that have not yet been described. Although the physiological and biochemical functions of mun-novel10-5p and mun-novel24-3p remain unclear, their differentially expressed patterns indicate that they might play important roles in sexual differentiation and development.

## Conclusions

In this study, the whole gonadal miRNAome of budgerigars was sequenced, consisting of a total of 12,929,838 clean reads and 2,942,541 unique reads. Moreover, differential expression of 254 known miRNAs and 141 novel miRNAs were analyzed in the gonadal tissues of budgerigars. The majority of these miRNAs were evolutionarily conserved within chordates while some of them were budgerigar- or avian-specific. In conclusion, this work describes the characteristics of sex-biased miRNAs of *M. undulatus* and adds new sequences to the avian miRNAome database to facilitate further functional genomic research.

##  Supplemental Information

10.7717/peerj.4615/supp-1 Table S1 Identification of conserved miRNA families in with corresponding isoforms

10.7717/peerj.4615/supp-2 Table S2 Prediction of novel miRNAs

10.7717/peerj.4615/supp-3 Table S3 The basic expression data of predicted miRNAs

10.7717/peerj.4615/supp-4 Table S4 The list of predicted multiple miRNAs target genes

10.7717/peerj.4615/supp-5 Table S5 The list of GO enrichment analysis on sex-related, vocal-related or learning related GO term

10.7717/peerj.4615/supp-6 Table S6 Most significantly enriched KEGG pathways for gonadal related genes

10.7717/peerj.4615/supp-7 Figure S1 RNA integrity number analysis by Agilent Bioanalyzer (testes marked as A, B, C; ovaries marked as 1, 2, 3)

10.7717/peerj.4615/supp-8 Figure S2 The secondary stable hairpin structures were predicted for identification of new miRNAs

## Additional Information and Declarations

### Data Availability

The following information was supplied regarding data availability: SRA database accession number SRR5664259 and SRR5664260. Jiang, Lan; Kan, Xianzhao (2018): budgerigar_mirnaomes_rawdata. figshare. https://doi.org/10.6084/m9.figshare.5187874.v1.
