# Supplementary material for: miRNAome expression profiles in the gonads of adult Melopsittacus undulatus
Source: PeerJ. 2018 Apr 9;6:e4615. doi: 10.7717/peerj.4615 (PMC5896495; doi:10.7717/peerj.4615)
Supplement: Figure S1 [file peerj-06-4615-s007.pdf]

Supplemental Figure S1. RNA integrity number analysis by Agilent Bioanalyzer (Testes marked as A, B, C; ovaries marked as 1, 2, 3).

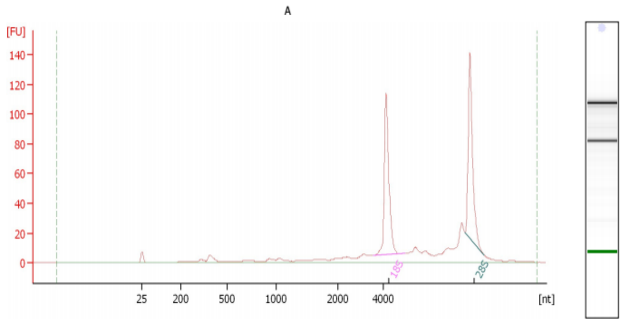

Overall Results for sample 1 : A

RNA Area:	477.0	RNA Integrity Number (RIN):	8.9 (B.02.08)
RNA Concentration:	915 ng/µl	Result Flagging Color:	
rRNA Ratio [28s / 18s]:	0.9	Result Flagging Label:	RIN: 8.90

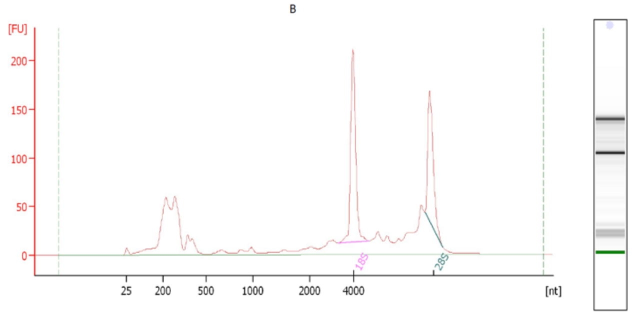

Overall Results for sample 2 : B

RNA Area:	1,401.2	RNA Integrity Number (RIN):	8.2 (B.02.08)
RNA Concentration:	811 ng/µl	Result Flagging Color:	
rRNA Ratio [28s / 18s]:	0.6	Result Flagging Label:	RIN: 8.20

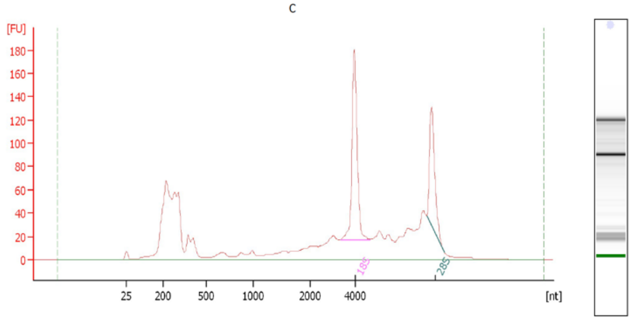

Overall Results for sample 3 : C

RNA Area:	1,452.1	RNA Integrity Number (RIN):	7.3 (B.02.08)
RNA Concentration:	841 ng/µl	Result Flagging Color:	
rRNA Ratio [28s / 18s]:	0.5	Result Flagging Label:	RIN: 7.30

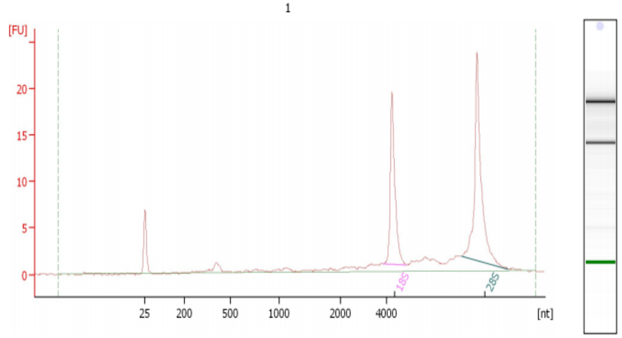

Overall Results for sample 2 : 1

RNA Area:	82.5	RNA Integrity Number (RIN):	9.2 (B.02.08)
RNA Concentration:	158 ng/µl	Result Flagging Color:	
rRNA Ratio [28s / 18s]:	1.4	Result Flagging Label:	RIN: 9.20

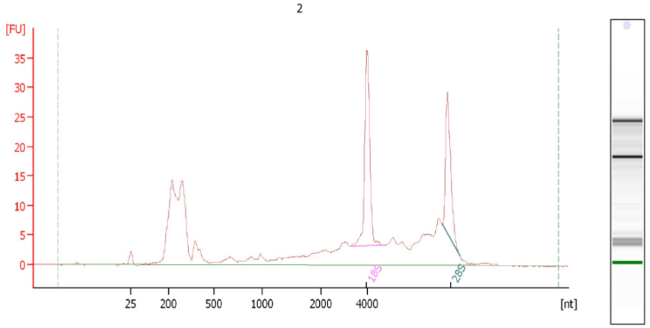

Overall Results for sample 5 : 2

RNA Area:	286.9	RNA Integrity Number (RIN):	7.3 (B.02.08)
RNA Concentration:	166 ng/µl	Result Flagging Color:	
rRNA Ratio [28s / 18s]:	0.6	Result Flagging Label:	RIN: 7.30

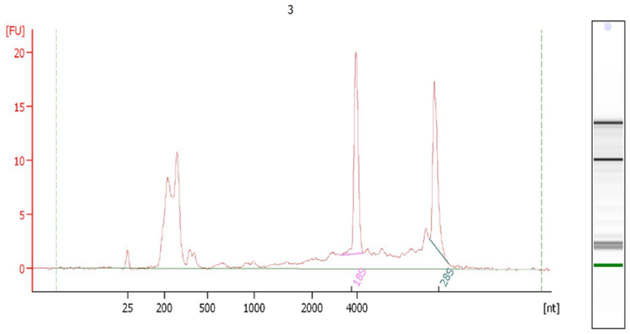

Overall Results for sample 6 : 3

RNA Area:	138.4	RNA Integrity Number (RIN):	7.8 (B.02.08)
RNA Concentration:	80 ng/µl	Result Flagging Color:	
rRNA Ratio [28s / 18s]:	0.8	Result Flagging Label:	RIN: 7.80
