# Supplementary material for: miRNAome expression profiles in the gonads of adult Melopsittacus undulatus
Source: PeerJ. 2018 Apr 9;6:e4615. doi: 10.7717/peerj.4615 (PMC5896495; doi:10.7717/peerj.4615)
Supplement: Figure S2 [file peerj-06-4615-s008.pdf]

Provisional ID	:	mun-novel38
Score total	:	6.1
Score for star read(s)	:	3.9
Score for read counts	:	-2.3
Score for mfe	:	3.6
Score for randfold	:	1.6
Score for cons. seed	:	-0.6
Total read count	:	7
Mature read count	:	6
Loop read count	:	0
Star read count	:	1

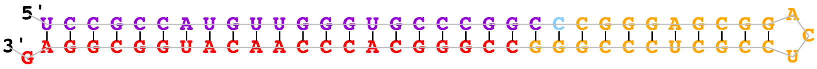

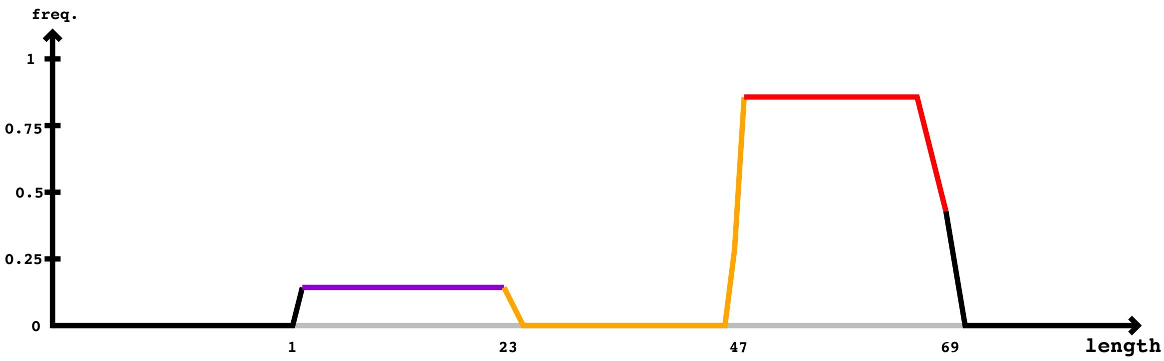

Star

Mature

```
5'- agacgagagaccccucagcuccacuccgccauguugggugcccggcccgggagcggacuccgcucccgggccgggcacccaacauggcggaguguggcugagguaaugcgg   -3'    obs
    agacgagagaccccucagcuccacuccgccauguugggugcccggcccgggagcggacuccgcucccgggccgggcacccaacauggcggaguguggcugagguaaugcgg          exp
    ...((.......(((((((..(((((((((((((((((((((((((((((((((((...)))))))))))))))))))))))))))))))))))..))))))).....)).    reads    mm    sample
    ........................uccgccauguugggugcccggc.................................................................    1        0     seq
    ....................................................................gUcgggcacccaacauggcg.......................    1        1     seq
    ....................................................................gUcgggcacccaacauggcgg......................    1        1     seq
    ......................................................................ccgggcacccaacauggcgga....................    1        0     seq
    ......................................................................Ucgggcacccaacauggcggag...................    1        1     seq
    ......................................................................ccgggcacccaacauggcggaC...................    2        1     seq
```

Provisional ID	:	mun-miR-92
Score total	:	61003.5
Score for star read(s)	:	3.9
Score for read counts	:	60993.1
Score for mfe	:	2
Score for randfold	:	1.6
Score for cons. seed	:	3
Total read count	:	119647
Mature read count	:	119625
Loop read count	:	0
Star read count	:	22

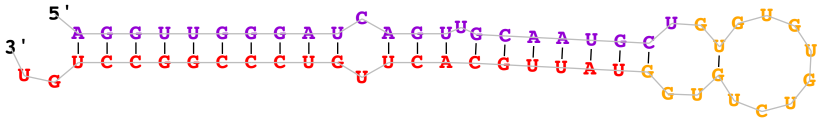

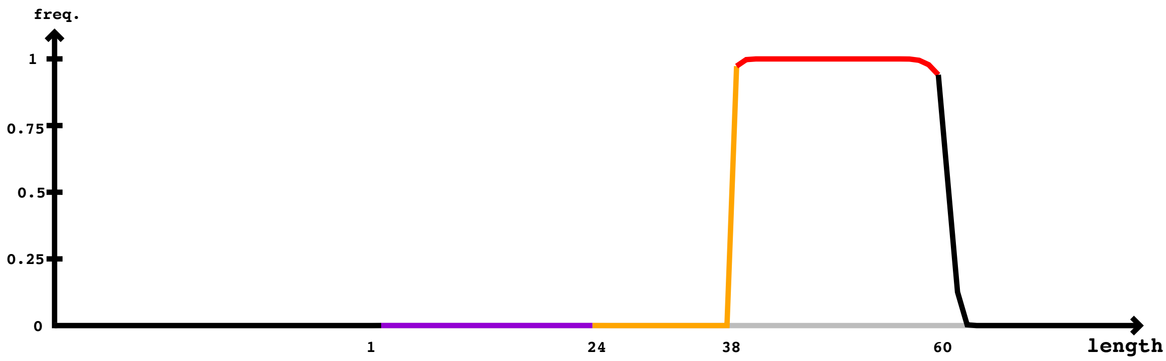

Star **Mature**

```
5'- ugaguguggaaaaguaguaccccuuucuacacagguugggaucaguugcaaugcugugugugucugugguauugcacuugucccggccuguugagguugguggggauagag -3'   obs
    ugaguguggaaaaguaguaccccuuucuacacagguugggaucaguugcaaugcugugugugucugugguauugcacuugucccggccuguugagguugguggggauagag        exp
    ...................((((..(((..((((((((((((.(((.(((((((..(........)..)))))))))).))))))))))))..))).....))))......      reads   mm   sample
    ...............................cagguugggaucaguugcaaug..........................................................      2       0    seq
    ...............................cagguugggaucaguugcaaugc.........................................................      1       0    seq
    ................................agguugggaucaguugcaaugc.........................................................      1       0    seq
    ................................agguugggaucaguugcaaugcu........................................................      15      0    seq
    ................................agguugggaucaguugcaaugcugug.....................................................      1       0    seq
    ..................................guugggaucaguugcaaugcu........................................................      1       0    seq
    ...................................uugggaucaguugcaaugcu........................................................      1       0    seq
    ................................................................Cgugguauugcacuugucccggccugu....................      2       1    seq
    .................................................................gugguauugcacuugucccggccugu....................      1       0    seq
    ...................................................................gguauugcacuugucccggccugu....................      1       0    seq
    ....................................................................guauugcacuugucccggccugu....................      1       0    seq
    ....................................................................Auauugcacuugucccggccugu....................      2       1    seq
    ....................................................................Cuauugcacuugucccggccugu....................      1       1    seq
    ....................................................................Uuauugcacuugucccggccugu....................      1       1    seq
    ....................................................................gCauugcacuugucccggccuguu...................      1       1    seq
    ....................................................................guauugcacuugucccggccuguuU..................      1       1    seq
    .....................................................................uauugcacuCgucccggc........................      22      1    seq
    .....................................................................uauugcacuugucccggc........................      27      0    seq
    .....................................................................uauugcacuugucccggcU.......................      4       1    seq
    .....................................................................uauugcacuugucccggcA.......................      6       1    seq
    .....................................................................uauugcacuAgucccggcc.......................      1       1    seq
    .....................................................................uauugcacuugucGcggcc.......................      1       1    seq
    .....................................................................uauugcacuugucUcggcc.......................      1       1    seq
    .....................................................................uauugcacuugucccgAcc.......................      1       1    seq
    .....................................................................uauugcacuuguUccggcc.......................      1       1    seq
    .....................................................................uauugcacuCgucccggcc.......................      72      1    seq
    .....................................................................uauugcacuugucccggcc.......................      466     0    seq
    .....................................................................uauugcacuugucUcggccu......................      3       1    seq
    .....................................................................uauuUcacuugucccggccu......................      2       1    seq
    .....................................................................uauugcacuugucccggccu......................      1358    0    seq
    .....................................................................uauugAacuugucccggccu......................      1       1    seq
    .....................................................................uauugcacuCgucccggccu......................      514     1    seq
    .....................................................................uauugcacuAgucccggccu......................      1       1    seq
```

Star Mature

```
ugaguguggaaaaguaguaccccuuucuacacagguugggaucaguugcaaugcugugugugucugugguauugcacuugucccggccuguugagguugguggggauagag
.....................................................................uauugcacuugucccggccG......................	14	1	seq
.....................................................................uauugcaAuugucccggccu......................	1	1	seq
.....................................................................uauugcacuugCcccggccu......................	1	1	seq
.....................................................................uauugcacuugucccggccA......................	62	1	seq
.....................................................................uauugcacuuguccUggccu......................	1	1	seq
.....................................................................Gauugcacuugucccggccu......................	1	1	seq
.....................................................................uauugcacuuguccAggccu......................	1	1	seq
.....................................................................uauugcacuuguUccggccu......................	1	1	seq
.....................................................................uauugcacuugucccggcUu......................	2	1	seq
.....................................................................uauugcacuugucccggAcu......................	1	1	seq
.....................................................................uauugcacuuUucccggccu......................	2	1	seq
.....................................................................uauugcacuugucccggccC......................	3	1	seq
.....................................................................uauugcacuugucccgAccu......................	1	1	seq
.....................................................................uauugcacuugucccggccuA.....................	20	1	seq
.....................................................................Gauugcacuugucccggccug.....................	3	1	seq
.....................................................................uauugcacuugucccggccuU.....................	112	1	seq
.....................................................................uauugcacuugucccggccCg.....................	2	1	seq
.....................................................................uauugcacCugucccggccug.....................	2	1	seq
.....................................................................uauugcUcuugucccggccug.....................	1	1	seq
.....................................................................uauugcacuuguGccggccug.....................	2	1	seq
.....................................................................uauugcacuugucccgAccug.....................	1	1	seq
.....................................................................uauugcacuugucccggccug.....................	4232	0	seq
.....................................................................uauugcacuuguUccggccug.....................	2	1	seq
.....................................................................uGuugcacuugucccggccug.....................	1	1	seq
.....................................................................uauugcacuugAcccggccug.....................	1	1	seq
.....................................................................uauugcacuuCucccggccug.....................	1	1	seq
.....................................................................uauugcacuugucccggccAg.....................	1	1	seq
.....................................................................Aauugcacuugucccggccug.....................	2	1	seq
.....................................................................uauCgcacuugucccggccug.....................	2	1	seq
.....................................................................uauugcacuugucccAgccug.....................	1	1	seq
.....................................................................uauugcacuugucccgUccug.....................	1	1	seq
.....................................................................uauuAcacuugucccggccug.....................	1	1	seq
.....................................................................uauugcacAugucccggccug.....................	1	1	seq
.....................................................................uauugcCcuugucccggccug.....................	1	1	seq
.....................................................................uauugcacuuguccUggccug.....................	1	1	seq
.....................................................................uauugcacuugucccggGcug.....................	2	1	seq
.....................................................................uauugcacuugucccggccGg.....................	48	1	seq
.....................................................................uauugcacuuguAccggccug.....................	1	1	seq
.....................................................................uauugcacuugucUcggccug.....................	1	1	seq
.....................................................................uauugcacuCgucccggccug.....................	1	1	seq
.....................................................................uauugcacuuAucccggccug.....................	1	1	seq
.....................................................................uauugAacuugucccggccug.....................	2	1	seq
.....................................................................Cauugcacuugucccggccug.....................	1	1	seq
.....................................................................uauugcGcuugucccggccug.....................	1	1	seq
.....................................................................uauugcacuugucccggUcug.....................	1	1	seq
.....................................................................uauugcacuuguccAggccug.....................	1	1	seq
.....................................................................uauugcacuugucccggAcug.....................	1	1	seq
.....................................................................uauuCcacuugucccggccugu....................	4	1	seq
.....................................................................uauugcacuugucccggccCgu....................	12	1	seq
.....................................................................uUuugcacuugucccggccugu....................	2	1	seq
.....................................................................uaAugcacuugucccggccugu....................	2	1	seq
.....................................................................uauugcGcuugucccggccugu....................	15	1	seq
.....................................................................uauugcacGugucccggccugu....................	1	1	seq
.....................................................................uauugcacuugucccggccugA....................	5025	1	seq
.....................................................................uauugcaAuugucccggccugu....................	8	1	seq
.....................................................................uauugAacuugucccggccugu....................	9	1	seq
.....................................................................uauugcUcuugucccggccugu....................	2	1	seq
.....................................................................uauugcacuuguAccggccugu....................	5	1	seq
.....................................................................uauugcacuugucccCgccugu....................	3	1	seq
.....................................................................uauugcacuugucccUgccugu....................	19	1	seq
.....................................................................uauugcacuuguccGggccugu....................	5	1	seq
.....................................................................uauugcacuAgucccggccugu....................	1	1	seq
.....................................................................uauugcacAugucccggccugu....................	2	1	seq
.....................................................................uauugcacuugucccggccuCu....................	3	1	seq
.....................................................................uauGgcacuugucccggccugu....................	1	1	seq
.....................................................................uauugcacuugucccAgccugu....................	5	1	seq
.....................................................................uauCgcacuugucccggccugu....................	6	1	seq
.....................................................................uaCugcacuugucccggccugu....................	6	1	seq
.....................................................................uauugcacuuUucccggccugu....................	11	1	seq
.....................................................................uauugcacuuAucccggccugu....................	8	1	seq
```

Star **Mature**

```
ugaguguggaaaaguaguaccccuuucuacacagguugggaucaguugcaaugcugugugugucugugguauugcacuugucccggccuguugagguugguggggauagag
.....................................................................uauugcacuugucccggccuUu....................   102     1   seq
.....................................................................uauugcacuuguUccggccugu....................   33      1   seq
.....................................................................uauugcaGuugucccggccugu....................   1       1   seq
.....................................................................uauugcacCugucccggccugu....................   5       1   seq
.....................................................................uauugcacuuguccAggccugu....................   18      1   seq
.....................................................................uauugcacuCgucccggccugu....................   8       1   seq
.....................................................................uauugcacuuCucccggccugu....................   4       1   seq
.....................................................................uCuugcacuugucccggccugu....................   2       1   seq
.....................................................................uauuAcacuugucccggccugu....................   4       1   seq
.....................................................................uauugGacuugucccggccugu....................   3       1   seq
.....................................................................uauugcacuuguccUggccugu....................   18      1   seq
.....................................................................CauugcacuugucccggccUgu....................   18      1   seq
.....................................................................uauuUcacuugucccggccugu....................   11      1   seq
.....................................................................uauugcacuugucccgUccugu....................   14      1   seq
.....................................................................uauugcacuugucccggccugG....................   414     1   seq
.....................................................................uauugUacuugucccggccugu....................   16      1   seq
.....................................................................uauugcacuugucccggGcugu....................   4       1   seq
.....................................................................uauugcacuugucccggccGgu....................   1       1   seq
.....................................................................uauugcacuugucccgCccugu....................   1       1   seq
.....................................................................uauugcacuugCcccggccugu....................   18      1   seq
.....................................................................uauugcacuugucccggccAgu....................   3       1   seq
.....................................................................uauugcacuugucUcggccugu....................   12      1   seq
.....................................................................Aauugcacuugucccggccugu....................   36      1   seq
.....................................................................uauugcacuugucccgAccugu....................   9       1   seq
.....................................................................uauugcacuugucccggUcugu....................   2       1   seq
.....................................................................uGuugcacuugucccggccugu....................   6       1   seq
.....................................................................uauugcacuugucccggccugu....................   70312   0   seq
.....................................................................uauugcacuugucccggAcugu....................   14      1   seq
.....................................................................Gauugcacuugucccggccugu....................   41      1   seq
.....................................................................uauugcacuugucccggcUugu....................   6       1   seq
.....................................................................uauugcacuugucccggccuAu....................   28      1   seq
.....................................................................uauugcacuugGcccggccugu....................   3       1   seq
.....................................................................uauugcacuugucccggcGugu....................   1       1   seq
.....................................................................uauugcacuugucGcggccugu....................   4       1   seq
.....................................................................uauugcacuugucAcggccugu....................   19      1   seq
.....................................................................uaGugcacuugucccggccugu....................   1       1   seq
.....................................................................uauugcacuugucccggcAugu....................   4       1   seq
.....................................................................uauugcaUuugucccggccugu....................   6       1   seq
.....................................................................uauAgcacuugucccggccugu....................   4       1   seq
.....................................................................uauugcacuugAcccggccugu....................   14      1   seq
.....................................................................uauugcacuuguGccggccugu....................   2       1   seq
.....................................................................uauugcacuugucccggccugC....................   169     1   seq
.....................................................................uauugcacuugucccCgccuguu...................   3       1   seq
.....................................................................uauugcacuuguccUggccuguu...................   7       1   seq
.....................................................................uauugcGcuugucccggccuguu...................   2       1   seq
.....................................................................uauugcacuugCcccggccuguu...................   1       1   seq
.....................................................................uauugcacuugucccggccuguu...................   11742   0   seq
.....................................................................uaAugcacuugucccggccuguu...................   1       1   seq
.....................................................................uauugcacuugucccggccCguu...................   2       1   seq
.....................................................................uauuCcacuugucccggccuguu...................   1       1   seq
.....................................................................uGuugcacuugucccggccuguu...................   1       1   seq
.....................................................................uauugcacuuguAccggccuguu...................   2       1   seq
.....................................................................uauugcacuugucccggccuUuu...................   43      1   seq
.....................................................................Aauugcacuugucccggccuguu...................   7       1   seq
.....................................................................uauugcacuugucccggccuAuu...................   13      1   seq
.....................................................................uauuAcacuugucccggccuguu...................   1       1   seq
.....................................................................uauugcacuugAcccggccuguu...................   1       1   seq
.....................................................................uauugcacuugucccggcAuguu...................   1       1   seq
.....................................................................uauugcacuugucccggccuguC...................   67      1   seq
.....................................................................Gauugcacuugucccggccuguu...................   14      1   seq
.....................................................................uauugcacuugucUcggccuguu...................   1       1   seq
.....................................................................uauugcacuugucGcggccuguu...................   3       1   seq
.....................................................................uauuUcacuugucccggccuguu...................   2       1   seq
.....................................................................uauugcacuugucccggcUuguu...................   1       1   seq
.....................................................................uauugcacuCgucccggccuguu...................   3       1   seq
.....................................................................uauugcacuugucccggccugGu...................   14      1   seq
.....................................................................uauugcacuugucccUgccuguu...................   3       1   seq
.....................................................................uauugcacAugucccggccuguu...................   1       1   seq
.....................................................................uauugcacuuguUccggccuguu...................   1       1   seq
.....................................................................uauugcacuugucAcggccuguu...................   2       1   seq
```

Star **Mature**

```
ugaguguggaaaaguaguaccccuuucuacacagguugggaucaguugcaaugcugugugugucugugguauugcacuugucccggccuguugagguugguggggauagag
.....................................................................uauugcacuuguccGggccuguu...................   1      1    seq
.....................................................................uauAgcacuugucccggccuguu...................   3      1    seq
.....................................................................uauugcUcuugucccggccuguu...................   2      1    seq
.....................................................................uauugcacuugucccgUccuguu...................   3      1    seq
.....................................................................uUuugcacuugucccggccuguu...................   1      1    seq
.....................................................................uauugcacuugGcccggccuguu...................   2      1    seq
.....................................................................uauugcacCugucccggccuguu...................   1      1    seq
.....................................................................uauugcacuuCucccggccuguu...................   4      1    seq
.....................................................................uauugcacuugucccggccuguA...................   6035   1    seq
.....................................................................uauugcacuugucccggccugAu...................   626    1    seq
.....................................................................uauugcacuugucccgAccuguu...................   1      1    seq
.....................................................................uauugUacuugucccggccuguu...................   4      1    seq
.....................................................................uauugcaUuugucccggccuguu...................   1      1    seq
.....................................................................uauugcacGugucccggccuguu...................   1      1    seq
.....................................................................uauugAacuugucccggccuguu...................   1      1    seq
.....................................................................uauugcacuuAucccggccuguu...................   2      1    seq
.....................................................................uauugcacuugucccggccugCu...................   12     1    seq
.....................................................................uauugcaAuugucccggccuguu...................   1      1    seq
.....................................................................uauugcacuugucccggccuguCg..................   1      1    seq
.....................................................................uauugcacuugucccggccuguAg..................   485    1    seq
.....................................................................uauugcacuugucccggccuguuA..................   687    1    seq
.....................................................................uauugcacuugucccggccuguuU..................   12518  1    seq
.....................................................................uauugcacuugucccggccuguug..................   35     0    seq
.....................................................................uauugcacuugucccggccuguuC..................   145    1    seq
.....................................................................uauugcacuugucccggccugAug..................   3      1    seq
.....................................................................uauugcacuugucccggccuguuAa.................   190    1    seq
.....................................................................uauugcacuugucccggccuguuga.................   6      0    seq
.....................................................................uauugcacuugucccggccuguugG.................   2      1    seq
.....................................................................uauugcacuugucccggccuguAga.................   54     1    seq
.....................................................................uauugcacuugucccggccuguugU.................   20     1    seq
.....................................................................uauugcacuugucccggccuguuUa.................   29     1    seq
.....................................................................uauugcacuugucccggccuguuUag................   1      1    seq
.....................................................................uauugcacuugucccggccuguugGgg...............   1      1    seq
......................................................................auugcacuugucccggcc.......................   7      0    seq
......................................................................auugcacuugucccggccu......................   10     0    seq
......................................................................auugcacuCgucccggccu......................   8      1    seq
......................................................................auugcacuugucccggccug.....................   59     0    seq
......................................................................auugcacuugucccggccGg.....................   1      1    seq
......................................................................auugcacuugucccggccuU.....................   1      1    seq
......................................................................auugcacuugucGcggccugu....................   1      1    seq
......................................................................Uuugcacuugucccggccugu....................   1      1    seq
......................................................................auugcacuugucccggccuUu....................   1      1    seq
......................................................................auugcacuugucccggccugu....................   1351   0    seq
......................................................................auugcacuugCcccggccugu....................   1      1    seq
......................................................................auugcacuuguAccggccugu....................   1      1    seq
......................................................................auugcacuugucccgUccugu....................   2      1    seq
......................................................................auugcacuugucccggccugG....................   9      1    seq
......................................................................auugcacuugucUcggccugu....................   2      1    seq
......................................................................auugcacuugucccggAcugu....................   1      1    seq
......................................................................Guugcacuugucccggccugu....................   1      1    seq
......................................................................auugcacuugucccggccugC....................   1      1    seq
......................................................................auugcacuugucccggccugA....................   12     1    seq
......................................................................auugcacuugucccUgccugu....................   1      1    seq
......................................................................auugcacuugucccggccuguu...................   719    0    seq
......................................................................auugcacuugucccggccuguC...................   14     1    seq
......................................................................Uuugcacuugucccggccuguu...................   1      1    seq
......................................................................auugcacuugucccggccuguA...................   90     1    seq
......................................................................auugcacuugucccggccugCu...................   1      1    seq
......................................................................auugcacuugucccggccCguu...................   1      1    seq
......................................................................auugcacuuUucccggccuguu...................   1      1    seq
......................................................................Guugcacuugucccggccuguu...................   1      1    seq
......................................................................auugcacuugucccggccugAu...................   3      1    seq
......................................................................auugcacuugucccggccuguAg..................   6      1    seq
......................................................................auugcacuugucccggccuguuC..................   9      1    seq
......................................................................auugcacuugucccggccuguug..................   1      0    seq
......................................................................auugcacuugucccggccuguuU..................   512    1    seq
......................................................................auugcacuugucccggccuguuA..................   51     1    seq
......................................................................auugcacuugucccggccuguuAa.................   30     1    seq
......................................................................auugcacuugucccggccuguugU.................   1      1    seq
......................................................................auugcacuugucccggccuguuUa.................   2      1    seq
```

```
                              Star                                  Mature
ugaguguggaaaaguaguaccccuuucuacacagguugggaucaguugcaaugcugugugugucugugguauugcacuugucccggccuguugagguugguggggauagag
.......................................................................uugcacuugucccggccu......................   4     0    seq
.......................................................................uugcacuugucccggccugu....................   18    0    seq
.......................................................................uugcacuugucccggccugA....................   1     1    seq
.......................................................................uugcacuugucccggccuguu...................   32    0    seq
.......................................................................uugcacuugucccggccuguC...................   1     1    seq
.......................................................................uugcacuugucccggccuguA...................   10    1    seq
.......................................................................uugcacuugucccggccuguuC..................   13    1    seq
.......................................................................uugcacuugucccggccuguuA..................   18    1    seq
.......................................................................uugcacuugucccggccuguuU..................   204   1    seq
.......................................................................uugcacuugucccggccuguugU.................   1     1    seq
.......................................................................uugcacuugucccggccuguuAa.................   9     1    seq
.......................................................................uugcacuugucccggccuguuCa.................   1     1    seq
.......................................................................uugcacuugucccggccuguuUa.................   1     1    seq
.........................................................................gcacuugucccggccugu....................   2     0    seq
```

Provisional ID	:	mun-miR-103
Score total	:	4316.3
Score for star read(s)	:	3.9
Score for read counts	:	4306.1
Score for mfe	:	1.6
Score for randfold	:	1.6
Score for cons. seed	:	3
Total read count	:	8458
Mature read count	:	8456
Loop read count	:	0
Star read count	:	2

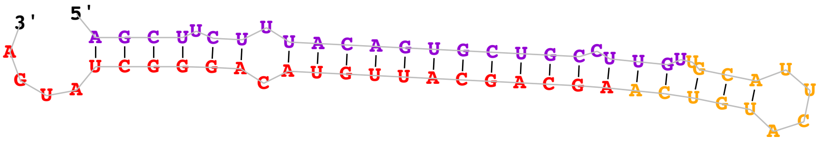

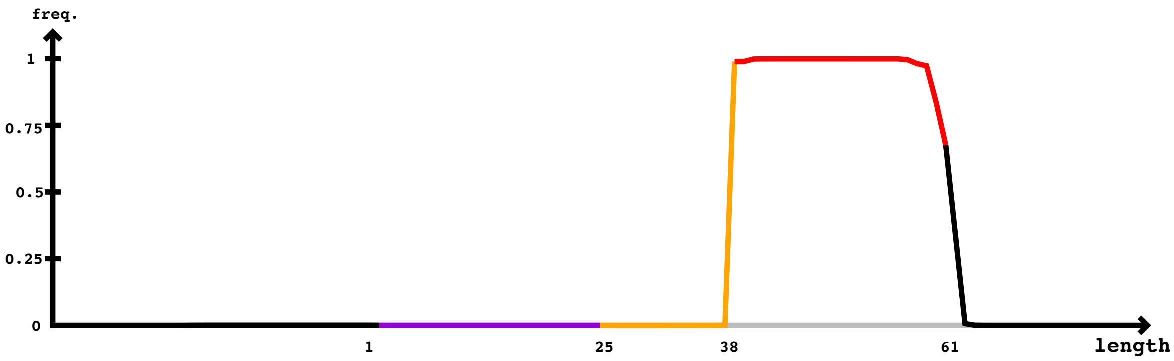


```
                                  Star                                   Mature
5'- uuccacagcuguuccuguaucuuugugcuuucagcuucuuuacagugcugccuuguugcauucaugucaagcagcauuguacagggcuaugaaagaacagagacaugcucuc  -3'  obs
    uuccacagcuguuccuguaucuuugugcuuucagcuucuuuacagugcugccuuguugcauucaugucaagcagcauuguacagggcuaugaaagaacagagacaugcucuc        exp
    ....((((......)))).(((((((.(((((((((.((.(((((((((((.(((..(((....))))))))))))))))).)))))..)))))).)))))))......... reads  mm  sample
    ............uccuguaucuuugugcuuuc................................................................................ 2      0   seq
    .............ccuguaucuuugugcuuuc................................................................................ 1      0   seq
    ..............cuguaucuuugugcuuuc................................................................................ 1      0   seq
    ................................agcuucuuuacagugcugccuugu........................................................ 2      0   seq
    ..............................................................caugucaagcagcauuguac.............................. 1      0   seq
    ..................................................................ucaagcagcauuguacagggcu........................ 1      0   seq
    ....................................................................aagcagcauuguacagggcuau...................... 1      0   seq
    ....................................................................aagcagcauuguacagggcuaugU.................... 1      1   seq
    .....................................................................agcagcauuguacagggc......................... 24     0   seq
    .....................................................................agcagcauuguUcagggcu........................ 1      1   seq
    .....................................................................agcagcauuguacagggcG........................ 1      1   seq
    .....................................................................agcagcauuguacagggcA........................ 3      1   seq
    .....................................................................agcagcauuguacagggUu........................ 1      1   seq
    .....................................................................agcagcauuguacagggcC........................ 1      1   seq
    .....................................................................agcagcauuguacagggcu........................ 118    0   seq
    .....................................................................agcagcauuguacagggcuU....................... 12     1   seq
    .....................................................................agcagcauuguacagggcCa....................... 2      1   seq
    .....................................................................agcagcauuguacagggcua....................... 54     0   seq
    .....................................................................agcagcauuguacagggcuG....................... 1      1   seq
    .....................................................................aUcagcauuguacagggcuau...................... 1      1   seq
    .....................................................................agcagcauuguacagggcuau...................... 1146   0   seq
    .....................................................................agcagcauuguacagggcuUu...................... 3      1   seq
    .....................................................................agcagcauuguacaUggcuau...................... 1      1   seq
    .....................................................................agcagcauuguacagggcuaA...................... 5      1   seq
    .....................................................................agcagcauuguacagggcuaC...................... 2      1   seq
    .....................................................................agcagcauuguacaggCcuau...................... 1      1   seq
    .....................................................................agcagcauuguacagggGuau...................... 1      1   seq
    .....................................................................agcagcauuguacagCgcuaug..................... 1      1   seq
    .....................................................................agcaCcauuguacagggcuaug..................... 1      1   seq
    .....................................................................agcagcauuguacagggcuauU..................... 258    1   seq
    .....................................................................agcagcauuguacagggcuaug..................... 1045   0   seq
    .....................................................................agcagcauuguaUagggcuaug..................... 1      1   seq
    .....................................................................agcagcauuguacagUgcuaug..................... 1      1   seq
```

Star   Mature

```
uuccacagcuguuccuguaucuuugugcuuucagcuucuuuacagugcugccuuguugcauucaugucaagcagcauuguacagggcuaugaaagaacagagacaugcucuc
.....................................................................agcagcauugAacagggcuaug.....................   1      1   seq
.....................................................................agcaAcauuguacagggcuaug.....................   1      1   seq
.....................................................................agcagcauuguacagggcuaGg.....................   2      1   seq
.....................................................................agcagcauuguacagAgcuaug.....................   1      1   seq
.....................................................................agGagcauuguacagggcuaug.....................   1      1   seq
.....................................................................agcagcauAguacagggcuaug.....................   1      1   seq
.....................................................................agcagcauuguacagggcuauA.....................   40     1   seq
.....................................................................agcagcauuguacagggcuaugU....................   1259   1   seq
.....................................................................agcaUcauuguacagggcuauga....................   1      1   seq
.....................................................................agcagcauuguacagggUuauga....................   1      1   seq
.....................................................................agcagcauuguacagggcuauAa....................   14     1   seq
.....................................................................agcagcauuAuacagggcuauga....................   1      1   seq
.....................................................................agcagcauuguaAagggcuauga....................   1      1   seq
.....................................................................agcagcauuguacagggcuauga....................   3831   0   seq
.....................................................................agcagcauugAacagggcuauga....................   1      1   seq
.....................................................................agcagcGuuguacagggcuauga....................   1      1   seq
.....................................................................agcagcauuguacagggcuaugG....................   9      1   seq
.....................................................................agcagcauuguaUagggcuauga....................   1      1   seq
.....................................................................agcagcauugGacagggcuauga....................   2      1   seq
.....................................................................agcagcauuguacagggcCauga....................   2      1   seq
.....................................................................agcagcauuguacagggcuauUa....................   3      1   seq
.....................................................................agcagcauuguacagggcuUuga....................   1      1   seq
.....................................................................agcagcauuguGcagggcuauga....................   1      1   seq
.....................................................................agcagcauuguacagggcuaugC....................   99     1   seq
.....................................................................Ugcagcauuguacagggcuauga....................   2      1   seq
.....................................................................agcagcauuguacagggcuaugaa...................   78     0   seq
.....................................................................agcagcauuguacagggcuaugaG...................   2      1   seq
.....................................................................agcagcauuguacagggcuaugUa...................   9      1   seq
.....................................................................agcagcauuguacagggcuaugaU...................   268    1   seq
.....................................................................agcagcauuguacagggcuaugaUa..................   8      1   seq
.....................................................................agcagcauuguacagCgcuaugaaa..................   1      1   seq
.....................................................................agcagcauuguacagggcuaugaaU..................   18     1   seq
.....................................................................agcagcauuguacagggcuaugaaa..................   19     0   seq
.....................................................................agcagcauuguacagggcuaugaaaU.................   3      1   seq
.....................................................................agcagcauuguacagggcuaugaaaAa................   1      1   seq
......................................................................gcagcauuguacagggcuaug.....................   1      0   seq
......................................................................gcagcauuguacagggcuauga....................   2      0   seq
.......................................................................cagcauuguacagggcuau......................   2      0   seq
.......................................................................cagcauuguacagggcuaug.....................   1      0   seq
.......................................................................cagcauugCacagggcuauga....................   1      1   seq
.......................................................................cagcauuguacagggcuaugU....................   3      1   seq
.......................................................................cagcauuguacagggcuaugC....................   1      1   seq
.......................................................................cagcauuguacagggcuauga....................   64     0   seq
.......................................................................cagcauuguacagggcuaugaU...................   1      1   seq
.......................................................................cagcauuguacagCgcuaugaaa..................   1      1   seq
.......................................................................cagcauuguacagggcuaugaaa..................   2      0   seq
.......................................................................cagcauuguacagggcuaugaUa..................   1      1   seq
.........................................................................agcauuguacagggcuaug....................   1      0   seq
.........................................................................agcauuguacagggcuauga...................   2      0   seq
.........................................................................agcauuguacagggcuaugaU..................   1      1   seq
..........................................................................gcauuguacagggcuauga...................   1      0   seq
```

Provisional ID	:	mun-novel65
Score total	:	8.6
Score for star read(s)	:	3.9
Score for read counts	:	1.7
Score for mfe	:	2
Score for randfold	:	1.6
Score for cons. seed	:	-0.6
Total read count	:	15
Mature read count	:	7
Loop read count	:	0
Star read count	:	8

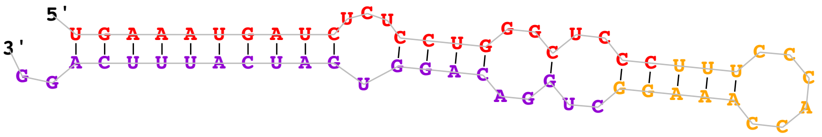

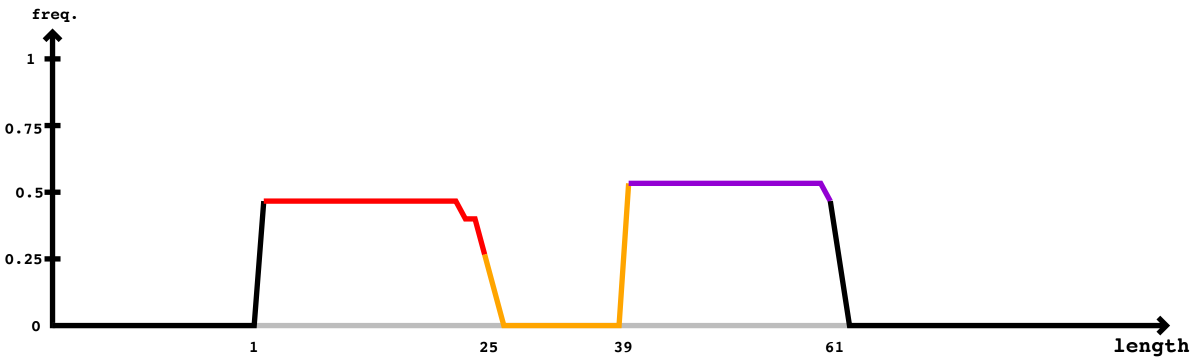

Mature Star

```
5'- agaugugguuggagagggugugaaaugaucucuccugggcucccuuucccaccaaaggcuggacaggugaucauuucagguccuucccagcccagccuguucugugaagaccug   -3'   obs
    agaugugguuggagagggugugaaaugaucucuccugggcucccuuucccaccaaaggcuggacaggugaucauuucagguccuucccagcccagccuguucugugaagaccug         exp
    ((.((.((((((..((((..((((((((((...((((..(..(((((......)))))..)..)))).))))))))))..))))..))))))))..))..(((....)))....    reads   mm   sample
    ....................ugaaaugaucucuccugggcu.........................................................................    1       0    seq
    ....................ugaaaugaucucuccugggcucc.......................................................................    2       0    seq
    ....................ugaaaugaucucuccugggcuccc......................................................................    3       0    seq
    ....................ugaaaugaucucuccugggcucccu.....................................................................    1       0    seq
    ..........................................................cuggacaggugaucauuucag...................................    1       0    seq
    ..........................................................cuggacaggugaucauuucagU..................................    2       1    seq
    ..........................................................cuggacaggugaucauuucagg..................................    2       0    seq
    ..........................................................cuggacaggugaucauuucaggu.................................    1       0    seq
    ..........................................................cuggacaggugaucauuucaggA.................................    2       1    seq
```

Provisional ID	:	mun-miR-24
Score total	:	3000.5
Score for star read(s)	:	3.9
Score for read counts	:	2990.3
Score for mfe	:	1.8
Score for randfold	:	1.6
Score for cons. seed	:	3
Total read count	:	5877
Mature read count	:	5869
Loop read count	:	0
Star read count	:	8

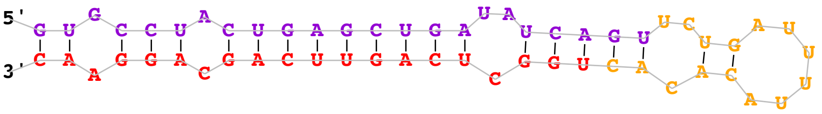

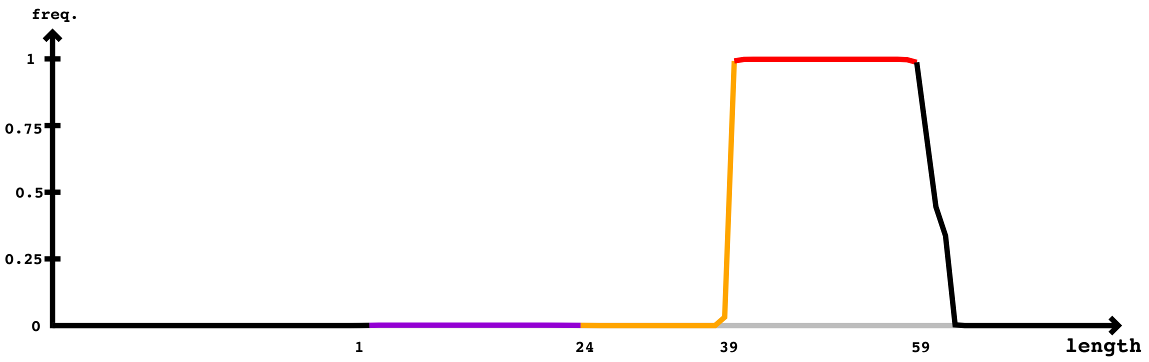

Star Mature

```
5'- uguuccaggcuguugauggacccguccuccggugccuacugagcugauaucaguucugauuuuacacacuggcucaguucagcaggaacaggagucgagcccuagagca  -3'   obs
    uguuccaggcuguugauggacccguccuccggugccuacugagcugauaucaguucugauuuuacacacuggcucaguucagcaggaacaggagucgagcccuagagca         exp
    .((((..((((...((((....))))((((.((.(((.(((((((((..(((((..((......)).))))).))))))))).))).)).))))...))))...)))).   reads    mm      sample
    ..............................ggugccuacugagcugauauca.........................................................   1        0       seq
    ...............................gugccuacugaUcugauaucagu.......................................................   1        1       seq
    ...............................gugccuacugagcugauaucagu.......................................................   2        0       seq
    ................................ugccuacugagcugauau...........................................................   1        0       seq
    ................................ugccuacugagcugauauc..........................................................   1        0       seq
    ................................ugccuacugagcugauauca.........................................................   1        0       seq
    ................................ugccuacugagcugauaucag........................................................   1        0       seq
    ................................ugccuacugagcugauaucagu.......................................................   1        0       seq
    ....................................................................cuggcucaguucagcaggaU.....................   1        1       seq
    ....................................................................cuggcucaguucagcaggaa.....................   1        0       seq
    ....................................................................cuggcucaguuAagcaggaac....................   1        1       seq
    ....................................................................cugAcucaguucagcaggaac....................   1        1       seq
    ....................................................................cuggcucaguucagcaggaac....................   163      0       seq
    ....................................................................cuggcuUaguucagcaggaac....................   1        1       seq
    ....................................................................cuggcucaguucagcaggaaca...................   9        0       seq
    ....................................................................cuggcucaguucagcaggaacU...................   3        1       seq
    ....................................................................cuggcucaguucagcaggaacagA.................   1        1       seq
    ....................................................................cuggcucaguucagcaggaacagU.................   4        1       seq
    .....................................................................uggcucaguucagcaggG......................   1        1       seq
    .....................................................................uggcucaguucagcagga......................   8        0       seq
    .....................................................................uggcucaguucagcaggaa.....................   28       0       seq
    .....................................................................uggcucaguucagcaggaU.....................   28       1       seq
    .....................................................................uggcucaguucagAaggaac....................   1        1       seq
    .....................................................................uggcucaguucagcagUaac....................   1        1       seq
    .....................................................................uggUucaguucagcaggaac....................   1        1       seq
    .....................................................................Gggcucaguucagcaggaac....................   4        1       seq
    .....................................................................uggcucaguucagcaggaaA....................   66       1       seq
    .....................................................................uggcucaguucagcaggaaG....................   6        1       seq
    .....................................................................Cggcucaguucagcaggaac....................   1        1       seq
    .....................................................................uggcucaguucagcaggaac....................   2165     0       seq
    .....................................................................uggcucaguuUagcaggaac....................   1        1       seq
    .....................................................................uggcucaguucagcaggaGc....................   5        1       seq
    .....................................................................uggcucaguucagUaggaac....................   4        1       seq
```

Star **Mature**

```
uguuccaggcuguugauggacccguccuccggugccuacugagcugauaucaguucugauuuuacacacuggcucaguucagcaggaacaggagucgagcccuagagca
.....................................................................uggcucaguucagcagCaac....................	1	1	seq
.....................................................................uggcucaguucagcaggaaU....................	70	1	seq
.....................................................................uggcucaguucagcaggUac....................	1	1	seq
.....................................................................uggcucaUuucagcaggaac....................	1	1	seq
.....................................................................Aggcucaguucagcaggaac....................	2	1	seq
.....................................................................uggcucaguucaUcaggaac....................	1	1	seq
.....................................................................uggcucagCucagcaggaac....................	1	1	seq
.....................................................................uggcucaguucagcaggaaca...................	578	0	seq
.....................................................................Aggcucaguucagcaggaaca...................	1	1	seq
.....................................................................uggcucaguucagUaggaaca...................	1	1	seq
.....................................................................uggcucaguucagcaggaacC...................	3	1	seq
.....................................................................uggcucaguucagcaggaacU...................	66	1	seq
.....................................................................uggcucaguucagcaggaacG...................	1	1	seq
.....................................................................uggcucaguucagcaggaaAa...................	14	1	seq
.....................................................................uggcucaguucagcagUaaca...................	1	1	seq
.....................................................................uggcucaguucagcaggaaUa...................	3	1	seq
.....................................................................uggcucaguucagcaggaacaA..................	23	1	seq
.....................................................................uggcucaguucagcaggaacaC..................	11	1	seq
.....................................................................uggcucaguucagcaggaacag..................	593	0	seq
.....................................................................uCgcucaguucagcaggaacag..................	1	1	seq
.....................................................................Aggcucaguucagcaggaacag..................	1	1	seq
.....................................................................uggcucaguucagcaggGacag..................	1	1	seq
.....................................................................uggcucaguucagcaggaacaU..................	3	1	seq
.....................................................................uggcucaguucagcaggaaAag..................	1	1	seq
.....................................................................uggcucaUuucagcaggaacag..................	1	1	seq
.....................................................................uggcucaguucagcaggaacagC.................	7	1	seq
.....................................................................uggcucaguucagcaggaacagg.................	18	0	seq
.....................................................................uggcucaguucagcaggaacagA.................	204	1	seq
.....................................................................uggcucaguucagcaggaacaAg.................	1	1	seq
.....................................................................uggcucaguucagcaggaacagU.................	1703	1	seq
.....................................................................uggcucagGucagcaggaacagg.................	1	1	seq
.....................................................................uggcucaguucagcaggaacagUa................	5	1	seq
.....................................................................uggcucaguucagcaggaacagAa................	2	1	seq
.....................................................................uggcucaguucagcaggaacaggU................	7	1	seq
......................................................................ggcucaguucagcaggaac....................	5	0	seq
......................................................................ggcucaguucagcaggaaca...................	2	0	seq
......................................................................ggcucaguucagcaggaacaC..................	1	1	seq
......................................................................ggcucaguucagcaggaacagU.................	23	1	seq
......................................................................ggcucaguucagcaggaacagUa................	1	1	seq
......................................................................ggcucaguucagcaggaacaggU................	1	1	seq
.......................................................................gcucaguucagcaggaacag..................	2	0	seq
.......................................................................gcucaguucagcaggaacagU.................	1	1	seq
```

Provisional ID	:	mun-novel29
Score total	:	1.5
Score for star read(s)	:	-1.3
Score for read counts	:	0
Score for mfe	:	1.8
Score for randfold	:	1.6
Score for cons. seed	:	-0.6
Total read count	:	126
Mature read count	:	126
Loop read count	:	0
Star read count	:	0

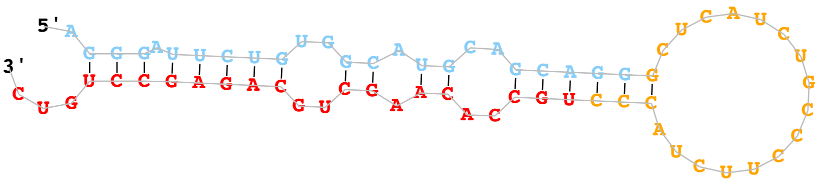

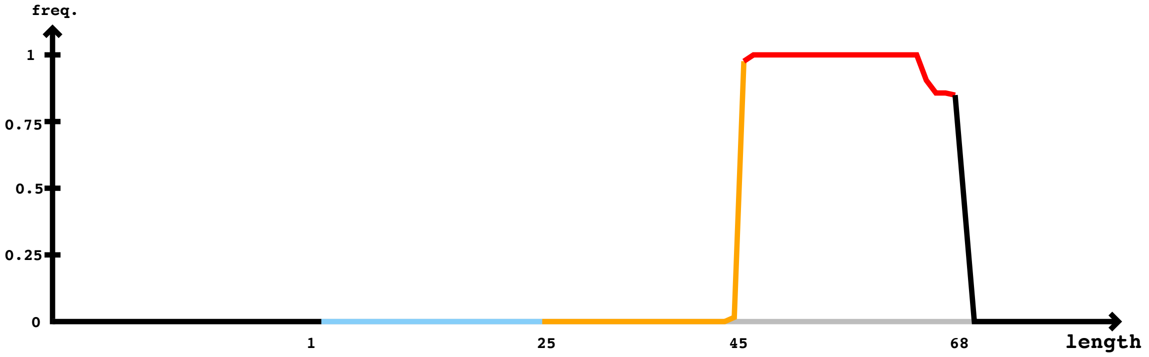

Star Mature

```
5'- ugcauggggggcauaaggccacaaggagggauucuguggcaugcagcagggcucaucugcccuucuacccugccacaagcugcagagccugucuucccacaggugcucc -3'  exp
    .(((((((.(((.....)))..(((..(((.(((((..((.((..((((((................))))))..)).))..))))))))..)))))))....)))...  reads  mm  sample
    .....................................................................cugccacaagcugcagagccugCc................  2      1   seq
    ......................................................................ugccacaagcugcagagcc....................  8      0   seq
    ......................................................................ugccaUaagcugcagagcc....................  3      1   seq
    ......................................................................ugccacaagcugcagagccu...................  4      0   seq
    ......................................................................ugccacaagcugcagagccugu.................  1      0   seq
    ......................................................................ugccacaagcugcagagccugCc................  75     1   seq
    ......................................................................ugccacaagcugcagagccugCcu...............  30     1   seq
    .......................................................................gccacaagcugcagagcc....................  1      0   seq
    .......................................................................gccacaagcugcagagccu...................  1      0   seq
    .......................................................................gccaUaagcugcagagccu...................  1      1   seq
```

Provisional ID : gi_525932624_ref_NW_004848298.1__43172
Score total : 188.2
Score for star read(s) : 3.9
Score for read counts : 178.1
Score for mfe : 1.7
Score for randfold : 1.6
Score for cons. seed : 3
Total read count : 361
Mature read count : 269
Loop read count : 0
Star read count : 92

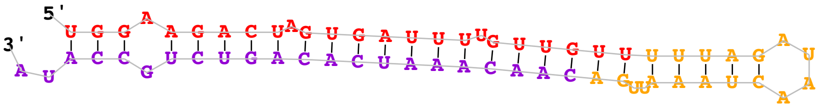

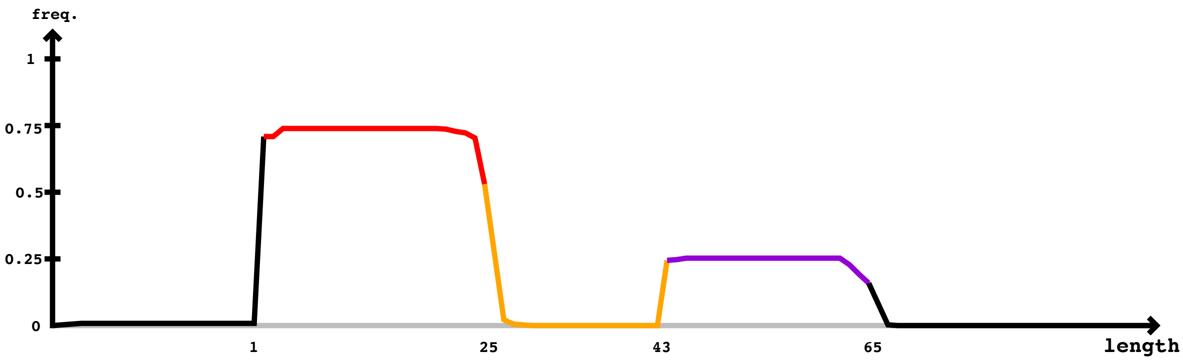

Mature Star

```
5'- gauguuggccuaguucugugugaagacuagugauuuuguuguuuuuagauaacuaaauugacaacaaaucacagucugccauauggcacagaccaugccucuacaggacaag -3'  obs
    gauguuggccuaguucugugugaagacuagugauuuuguuguuuuuagauaacuaaauugacaacaaaucacagucugccauauggcacagaccaugccucuacaggacaag       exp
    ............(((((((((((.(((((.(((((((.(((((((((((....)))))..)))))))))))))))))).))))..((((.......))))...)))))))...       reads  mm  sample
    gauguuggccuaguucugug.............................................................................................       2      0   seq
    .auguuggccuaguucugug.............................................................................................       1      0   seq
    ....................uggaagacuagugauuuug..........................................................................       1      0   seq
    ....................uggaagacuagugauuuugu.........................................................................       3      0   seq
    ....................uggaagacuagugauuuuguu........................................................................       2      0   seq
    ....................uggaagacuagugauuuuguug.......................................................................       6      0   seq
    ....................uggaagacuagugauuuuguuU.......................................................................       1      1   seq
    ....................uggaagacuagugauuuuguugu......................................................................       61     0   seq
    ....................uggaGgacuagugauuuuguugu......................................................................       1      1   seq
    ....................uggaagacuagAgauuuuguuguu.....................................................................       1      1   seq
    ....................uggaagacuagugauuuuguuguu.....................................................................       161    0   seq
    ....................uggaagacuaAugauuuuguuguuu....................................................................       1      1   seq
    ....................uggaagacuagugauuuuguuguuu....................................................................       13     0   seq
    ....................uggaagacuagugauuuuguuguuAu...................................................................       3      1   seq
    ....................uggaagacuagugauuuuguuguuuu...................................................................       1      0   seq
    ....................uggaagacuagugauuuuguuguAuu...................................................................       1      1   seq
    ....................uggaagacuagugauuuuguuguuAuu..................................................................       1      1   seq
    ....................uggaagacuagugauuuuguuguuuuuU.................................................................       1      1   seq
    ......................gaagacuagugauuuuguugu......................................................................       1      0   seq
    ......................gaagacuagugauuuuguuguu.....................................................................       6      0   seq
    ......................gaagacuagugauuuuguuUuu.....................................................................       1      1   seq
    ......................gaagacuagugauuuuguuguuA....................................................................       1      1   seq
    ......................gaagacuagugauuuuguuguuu....................................................................       1      0   seq
    ......................gaagacuagugauuuuguuguuuu...................................................................       1      0   seq
    ..............................................................caacaaaucacagucugcc................................       6      0   seq
    ..............................................................caacaaauUacagucugcc................................       3      1   seq
    ..............................................................caacaaaucacagucugcca...............................       9      0   seq
    ..............................................................caacaaauUacagucugcca...............................       4      1   seq
    ..............................................................caacaaauUacagucugccau..............................       1      1   seq
    ..............................................................caacaaaucacagucugccau..............................       11     0   seq
    ..............................................................caacaaaucacagucugccauU.............................       14     1   seq
    ..............................................................caacaaauUacagucugccaua.............................       12     1   seq
    ..............................................................caacaaaucacagucugccaua.............................       27     0   seq
```

```
                    Mature                                          Star
gauguuggccuaguucugugugaagacuagugauuuuguuguuuuuagauaacuaaauugacaacaaaucacagucugccauauggcacagaccaugccucuacaggacaag
..............................................................caacaaaucacagucugccauCu............................	1	1	seq
..............................................................caacaaaucacagucugccauau............................	1	0	seq
...............................................................aacaaaucacagucugccauau............................	1	0	seq
................................................................acaaaucacagucugccauau............................	1	0	seq
................................................................acaaaucacagucugccauauU...........................	1	1	seq
```

Provisional ID	:	mun-miR-146c
Score total	:	5191.4
Score for star read(s)	:	3.9
Score for read counts	:	5181.5
Score for mfe	:	1.4
Score for randfold	:	1.6
Score for cons. seed	:	3
Total read count	:	10175
Mature read count	:	9274
Loop read count	:	1
Star read count	:	900

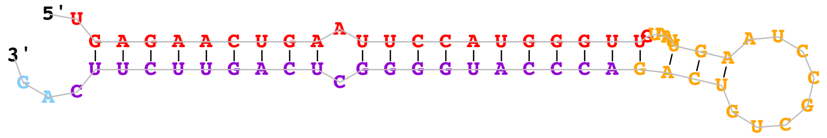

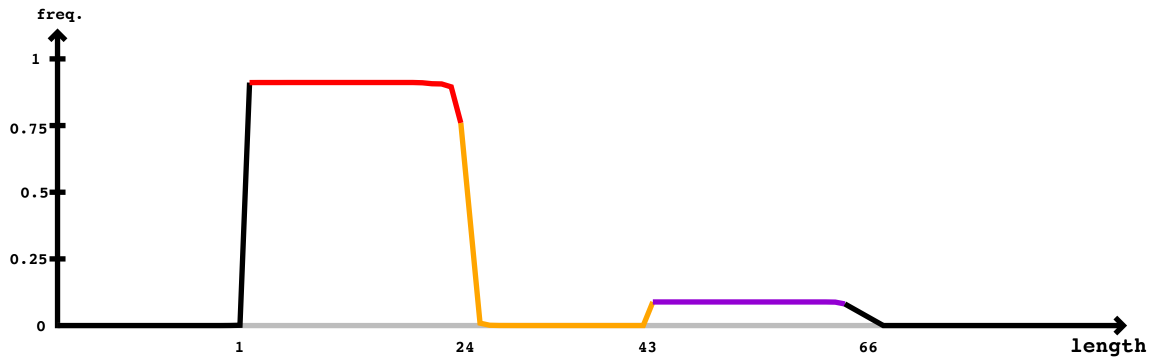

Mature   Star

```
5'- gucguuuauucucagcuuugagaacugaauuccauggguuguaauugaauccgcugucagacccauggggcucaguucuucagcuuggauauuucugucuucauacaca   -3'   obs
    gucguuuauucucagcuuugagaacugaauuccauggguuguaauugaauccgcugucagacccauggggcucaguucuucagcuuggauauuucugucuucauacaca         exp
    .........(((.((((..((((((((.((((((((((.....(((........))))))))))))).)))))))).)))).))).........................   reads   mm   sample
    ................uCugagaacugaauuccauggguug.....................................................................   2       1    seq
    ................uCugagaacugaauuccauggguugu....................................................................   1       1    seq
    .................uugagaacugaauuccaugggu.......................................................................   3       0    seq
    .................Cugagaacugaauuccaugggu.......................................................................   1       1    seq
    .................uugagaacugaauuccauggguG......................................................................   1       1    seq
    .................Cugagaacugaauuccauggguug.....................................................................   3       1    seq
    ..................ugagaacugaauuccaug..........................................................................   11      0    seq
    ..................ugagaacugaauuccaugg.........................................................................   35      0    seq
    ..................ugagaacugaauuccaugA.........................................................................   1       1    seq
    ..................ugagaacugaauuccauggg........................................................................   8       0    seq
    ..................ugagaacugaauuccauggAu.......................................................................   17      1    seq
    ..................ugagaacugaauuccaugggA.......................................................................   3       1    seq
    ..................ugagaacugaauuccaugggG.......................................................................   1       1    seq
    ..................ugagaacugaauuccaugggu.......................................................................   80      0    seq
    ..................ugagaacugaauuccauAggu.......................................................................   7       1    seq
    ..................ugagaacugaauuccauggguu......................................................................   1326    0    seq
    ..................Agagaacugaauuccauggguu......................................................................   2       1    seq
    ..................ugagaacugaauuccUuggguu......................................................................   1       1    seq
    ..................ugagaacugaauuccaugggCu......................................................................   13      1    seq
    ..................ugagGacugaauuccauggguu......................................................................   1       1    seq
    ..................ugagaacugCauuccauggguu......................................................................   1       1    seq
    ..................ugagaacugaauuccauggAuu......................................................................   9       1    seq
    ..................ugagaacugaauuGcauggguu......................................................................   2       1    seq
    ..................ugagaacugaauuccaugggAu......................................................................   3       1    seq
    ..................ugagaacugaauuccauggguG......................................................................   3       1    seq
    ..................ugagaacugaauucUauggguu......................................................................   6       1    seq
    ..................ugagaacugaauuccauggguA......................................................................   10      1    seq
    ..................ugagaacugaauuccaAggguug.....................................................................   1       1    seq
    ..................ugagaacugaauuccauggguGg.....................................................................   4       1    seq
    ..................ugagaacugaauuccauggguug.....................................................................   3681    0    seq
    ..................uUagaacugaauuccauggguug.....................................................................   1       1    seq
    ..................ugagaacugaauuAcauggguug.....................................................................   1       1    seq
    ..................Agagaacugaauuccauggguug.....................................................................   4       1    seq
```

Mature   Star

```
gucguuuauucucagcuuugagaacugaauuccauggguuguaauugaauccgcugucagacccauggggcucaguucuucagcuuggauauuucugucuucauacaca
..................ugagaacugaauuccGuggguug....................................................................   1      1     seq
..................ugagaacugaauuccaugUguug....................................................................   1      1     seq
..................Ggagaacugaauuccauggguug....................................................................   4      1     seq
..................ugagaacugaauuccaugggGug....................................................................   5      1     seq
..................ugagaacugaauuccauUgguug....................................................................   1      1     seq
..................ugaUaacugaauuccauggguug....................................................................   1      1     seq
..................ugagaacugaauuccaugggAug....................................................................   1      1     seq
..................ugagaacugaauuccauggguuA....................................................................   109    1     seq
..................ugagaacugaauuUcauggguug....................................................................   1      1     seq
..................ugagaacugaauuccauggguuU....................................................................   402    1     seq
..................ugagaacugaauucUauggguug....................................................................   2      1     seq
..................ugagaacugaauuccauggguuC....................................................................   4      1     seq
..................ugagaacugaauuccaugggCug....................................................................   9      1     seq
..................ugagaacugaauuccauggAuug....................................................................   13     1     seq
..................ugagaaAugaauuccauggguug....................................................................   1      1     seq
..................ugagaacugaauuccauggUuug....................................................................   2      1     seq
..................uAagaacugaauuccauggguug....................................................................   1      1     seq
..................ugagaacugaauuccaugAguug....................................................................   1      1     seq
..................uCagaacugaauuccauggguugu...................................................................   1      1     seq
..................ugagaacugaauuccauggguuAu...................................................................   31     1     seq
..................ugagaacugaauuccauggguuCu...................................................................   1      1     seq
..................ugagaacugaauuccUuggguugu...................................................................   1      1     seq
..................ugagaacugaauuccGuggguugu...................................................................   1      1     seq
..................ugagaacugaauuccauggAuugu...................................................................   17     1     seq
..................ugagaacCgaauuccauggguugu...................................................................   1      1     seq
..................ugagaacugaauuccauggguuUu...................................................................   63     1     seq
..................ugagaaAugaauuccauggguugu...................................................................   1      1     seq
..................ugagaacugaauuccauggguugu...................................................................   2905   0     seq
..................ugaCaacugaauuccauggguugu...................................................................   1      1     seq
..................ugagaacugaauuccauggguugC...................................................................   13     1     seq
..................ugagaacugaaGuccauggguugu...................................................................   1      1     seq
..................ugagaacugaauuccauggCuugu...................................................................   2      1     seq
..................ugagaacugaauuccaugggCugu...................................................................   7      1     seq
..................Ggagaacugaauuccauggguugu...................................................................   1      1     seq
..................ugagaacugaauuAcauggguugu...................................................................   1      1     seq
..................ugaUaacugaauuccauggguugu...................................................................   1      1     seq
..................ugagaacugaauuccaugggGugu...................................................................   2      1     seq
..................ugagaacugaauuccauggguugG...................................................................   8      1     seq
..................ugagaacugaauuccaAgguugu....................................................................   1      1     seq
..................ugagaacugaauuccaCgguugu....................................................................   1      1     seq
..................ugagaacugaauuccauggguugA...................................................................   314    1     seq
..................Cgagaacugaauuccauggguugu...................................................................   1      1     seq
..................ugagaacugaauuccauggguuUua..................................................................   1      1     seq
..................ugagaacugaauuccauggguugua..................................................................   15     0     seq
..................ugagaacugaauuccaCgguugua...................................................................   1      1     seq
..................ugagaacugaauuccauggguugCa..................................................................   1      1     seq
..................ugagaacugaauuccauggguuguU..................................................................   60     1     seq
..................ugagaacugaauuccauggguuguG..................................................................   1      1     seq
..................ugagaacugaauuccauggguugAa..................................................................   2      1     seq
..................ugagaacugaauuccauggguuguaa.................................................................   2      0     seq
..................ugagaacugaauuccauggguuguaU.................................................................   7      1     seq
..................ugagaacugaauuccauggguuguaau................................................................   1      0     seq
..................ugagaacugaauuccauggguugAaauu...............................................................   1      1     seq
...................gagaacugaauuccauggguug....................................................................   1      0     seq
...................gagaacugaauuccauggguugu...................................................................   1      0     seq
......................aacugaauuccauggguugu...................................................................   1      0     seq
.......................acugaauuccaugggCug....................................................................   1      1     seq
........................................guaauugaauccgcugucag.................................................   1      0     seq
............................................................acccauggggcucaguucu..............................   6      0     seq
............................................................acccauggggcucaguucuA.............................   1      1     seq
............................................................acccauggggcucaguucuu.............................   69     0     seq
............................................................acccauggggcuUaguucuuc............................   1      1     seq
............................................................acccauggggcucaguucuuU............................   2      1     seq
............................................................acccauggggcucaguuUuuc............................   1      1     seq
............................................................acccauggggcucagGucuuc............................   1      1     seq
............................................................acccauggUgcucaguucuuc............................   1      1     seq
............................................................acccauggggcucaguucuuA............................   1      1     seq
............................................................acccauggggcucaguucuuc............................   684    0     seq
............................................................acccauggggcucaguucuuca...........................   30     0     seq
............................................................acccauggggcucaguucuucU...........................   93     1     seq
```

Mature **Star**

```
gucguuuauucucagcuuugagaacugaauuccauggguuguaauugaauccgcugucagacccauggggcucaguucuucagcuuggauauuucugucuucauacaca
............................................................acccauggggcucaguucuucaU..........................    1    1    seq
............................................................acccauggggcucaguucuucaA..........................    8    1    seq
............................................................acccauggggcucaguucuucaC..........................    1    1    seq
```

Provisional ID	:	mun-miR-34a
Score total	:	63.8
Score for star read(s)	:	3.9
Score for read counts	:	53.1
Score for mfe	:	2.2
Score for randfold	:	1.6
Score for cons. seed	:	3
Total read count	:	116
Mature read count	:	114
Loop read count	:	0
Star read count	:	2

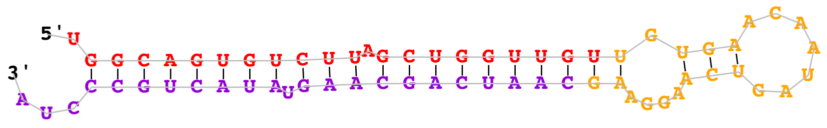

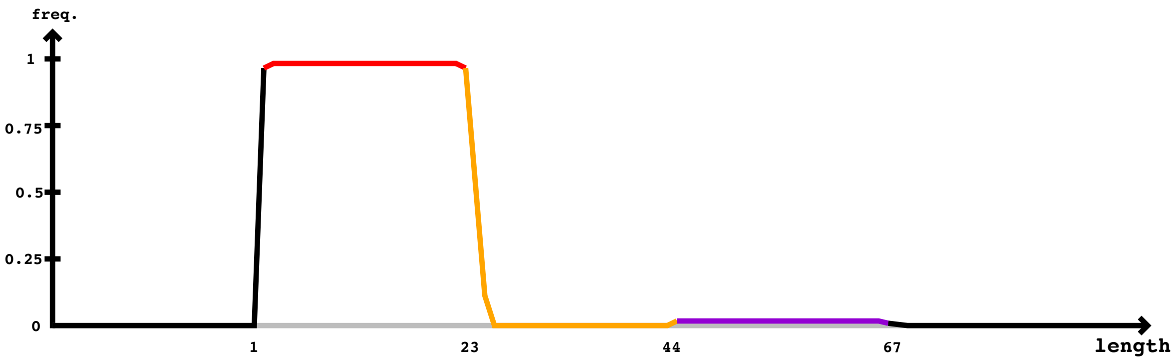


```
                          Mature                                 Star
5'- gccagcugugaguguuucuuuggcagugucuuagcugguuguugugaacaauagucaaggaagcaaucagcaaguauacugcccuagaagugcuacacauuguugggccugc   -3'   obs
    gccagcugugaguguuucuuuggcagugucuuagcugguuguugugaacaauagucaaggaagcaaucagcaaguauacugcccuagaagugcuacacauuguugggccugc         exp
    .((((((((((..(((((..(((((((((((.((((((((((.(((.......)))....)))))))))))))).))))))))..)))))..)).))))..)))))......    reads   mm   sample
    ....................uggcagugucuuagcugguug.......................................................................    2       0    seq
    ....................uggcagugucuuUgcugguugu......................................................................    1       1    seq
    ....................uggcagugucuuagcugguugu......................................................................    56      0    seq
    ....................uggcagugucuuagcugguuguu.....................................................................    40      0    seq
    ....................uggcagugucuuagcugguuguA.....................................................................    1       1    seq
    ....................uggcagugucuuagcugguuguuU....................................................................    12      1    seq
    .....................ggcagugucuuagcugguuguu.....................................................................    1       0    seq
    .....................ggcagugucuuagcugguuguuU....................................................................    1       1    seq
    ...............................................................caaucagcaaguauacugcccu...........................    1       0    seq
    ...............................................................caaucagcaaguauacugcccua..........................    1       0    seq
```

Provisional ID	:	mun-let-7e
Score total	:	587.2
Score for star read(s)	:	3.9
Score for read counts	:	576.7
Score for mfe	:	1.9
Score for randfold	:	1.6
Score for cons. seed	:	3
Total read count	:	1143
Mature read count	:	1135
Loop read count	:	0
Star read count	:	8

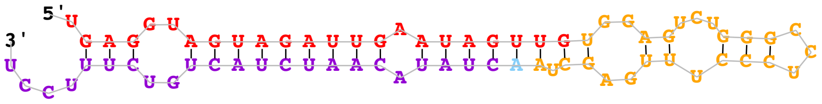

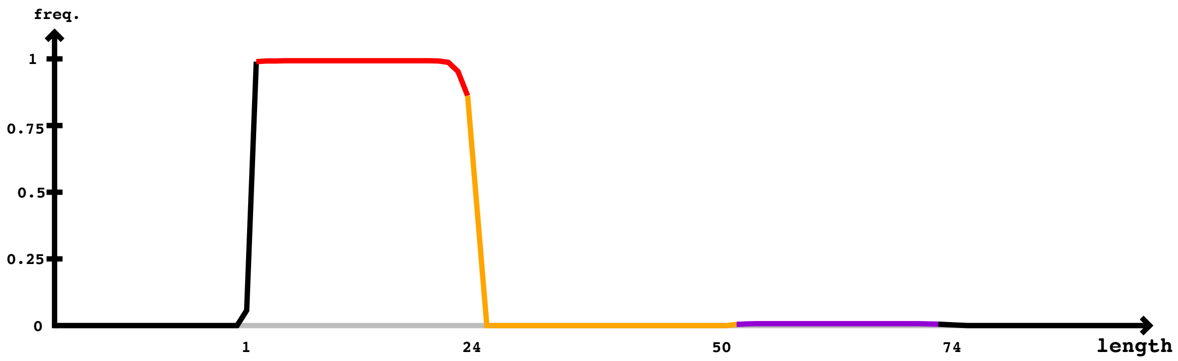

Mature **Star**

```
5'- cccaagcuguccuugaggcugagguaguagauugaauaguuguggagucugggccucccuuugagcuaacuauacaaucuacugucuuuccuaaggagacagucaggucuuc  -3'  obs
    cccaagcuguccuugaggcugagguaguagauugaauaguuguggagucugggccucccuuugagcuaacuauacaaucuacugucuuuccuaaggagacagucaggucuuc       exp
    .((..(((((((((.(((..(((..(((((((((.((((((((..((...(((...))).))..)).)))))).)))))))))..))).))).))).))))))..)).....  reads  mm  sample
    ..................cugagguaguagauugaauU..........................................................................  1  1  seq
    ..................cugagguaguagauugaauaA.........................................................................  1  1  seq
    ..................cugagguagGagauugaauagu........................................................................  1  1  seq
    ..................cugagguaguagauugaauGgu........................................................................  1  1  seq
    ..................cugaUguaguagauugaauagu........................................................................  1  1  seq
    ..................cugagguaguagauugaauagA........................................................................  3  1  seq
    ..................cugagguaguagauuCaauagu........................................................................  1  1  seq
    ..................cugagguaguagauugUauagu........................................................................  2  1  seq
    ..................cugagguaguagauugUauaguu.......................................................................  14  1  seq
    ..................cugagguaguagauugaauaguA.......................................................................  3  1  seq
    ..................cugagguagCagauugaauaguu.......................................................................  1  1  seq
    ..................cugagguagGagauugaauaguu.......................................................................  1  1  seq
    ..................cugagguagAagauugaauaguu.......................................................................  1  1  seq
    ..................cugagguaguagauugaauaguuA......................................................................  5  1  seq
    ..................cugagguaguagauugaauaguuU......................................................................  29  1  seq
    ...................ugaggAaguagauugaauag.........................................................................  1  1  seq
    ...................ugagguaguagauugaauaA.........................................................................  2  1  seq
    ...................ugagguaguagauugaauaU.........................................................................  2  1  seq
    ...................ugagguaguGgauugaauagu........................................................................  1  1  seq
    ...................ugagguaguagGuugaauagu........................................................................  2  1  seq
    ...................ugagguaguagauugaaCagu........................................................................  1  1  seq
    ...................ugaUguaguagauugaauagu........................................................................  1  1  seq
    ...................ugagguaguagauugaauaUu........................................................................  1  1  seq
    ...................ugaggCaguagauugaauagu........................................................................  2  1  seq
    ...................ugagguaguagauugaauagA........................................................................  10  1  seq
    ...................ugagguaguagauugaauagG........................................................................  1  1  seq
    ...................ugaggAaguagauugaauagu........................................................................  1  1  seq
    ...................ugagguagCagauugaauagu........................................................................  1  1  seq
    ...................uAagguaguagauugaauagu........................................................................  1  1  seq
    ...................Agagguaguagauugaauagu........................................................................  1  1  seq
    ...................ugagguaguaUauugaauagu........................................................................  1  1  seq
    ...................ugagguaguagauugGauagu........................................................................  1  1  seq
    ...................ugagguGguagauugaauagu........................................................................  1  1  seq
```

```
                   Mature                                                Star
cccaagcuguccuugaggcugagguaguagauugaauaguuguggagucugggccucccuuugagcuaacuauacaaucuacugucuuuccuaaggagacagucaggucuuc
...................Ggagguaguagauugaauagu........................................................................	1	1	seq
...................ugaggGaguagauugaauagu........................................................................	2	1	seq
...................ugagguaCuagauugaauagu........................................................................	1	1	seq
...................ugagguaUuagauugaauaguu.......................................................................	1	1	seq
...................ugagguaguagauugaauaguA.......................................................................	15	1	seq
...................ugagguaguagauugCauaguu.......................................................................	1	1	seq
...................ugagCuaguagauugaauaguu.......................................................................	1	1	seq
...................ugagguaguagauuAaauaguu.......................................................................	2	1	seq
...................ugagguaguagaCugaauaguu.......................................................................	1	1	seq
...................ugagguGguagauugaauaguu.......................................................................	1	1	seq
...................ugagguaguagauugaGuaguu.......................................................................	3	1	seq
...................ugagguaguagauugaauaUuu.......................................................................	1	1	seq
...................ugagguaguagauuUaauaguu.......................................................................	1	1	seq
...................ugagguaguagauAgaauaguu.......................................................................	1	1	seq
...................ugaggCaguagauugaauaguu.......................................................................	5	1	seq
...................ugaggGaguagauugaauaguu.......................................................................	8	1	seq
...................Ggagguaguagauugaauaguu.......................................................................	4	1	seq
...................ugagguaguGgauugaauaguu.......................................................................	1	1	seq
...................Cgagguaguagauugaauaguu.......................................................................	2	1	seq
...................ugaggAaguagauugaauaguu.......................................................................	7	1	seq
...................ugagguaguagauugaauaguC.......................................................................	2	1	seq
...................ugagguaguaUauugaauaguu.......................................................................	1	1	seq
...................Agagguaguagauugaauaguu.......................................................................	3	1	seq
...................ugagguaguagUuugaauaguu.......................................................................	1	1	seq
...................ugagguaguagauugaauagGu.......................................................................	2	1	seq
...................ugagguaguagauugaauaCuu.......................................................................	1	1	seq
...................ugagguaguagauugaauaguG.......................................................................	1	1	seq
...................ugagguagCagauugaauaguu.......................................................................	1	1	seq
...................uAagguaguagauugaauaguu.......................................................................	2	1	seq
...................ugagguaguUgauugaauaguu.......................................................................	1	1	seq
...................ugagguaguagauugaUuaguu.......................................................................	1	1	seq
...................ugagUuaguagauugaauaguu.......................................................................	1	1	seq
...................ugagguaguagGuugaauaguu.......................................................................	1	1	seq
...................ugagguaguagauugGauaguu.......................................................................	11	1	seq
...................ugagguaguagauugaauaguuA......................................................................	264	1	seq
...................ugagguaguagauugaauaguuC......................................................................	6	1	seq
...................ugagguaguagauugaauaguuU......................................................................	656	1	seq
...................ugagguaguagauugaauaguuUu.....................................................................	10	1	seq
...................ugagguaguagauugaauaguuAu.....................................................................	11	1	seq
....................gagguaguagauugaauaguuU......................................................................	2	1	seq
....................gagguaguagauugaauaguuA......................................................................	1	1	seq
......................gguaguagauugaauaguuU......................................................................	1	1	seq
.....................................................................cuauacaGucuacugucuuu.......................	1	1	seq
.....................................................................cCauacaaucuacugucuuuc......................	1	1	seq
.....................................................................cuauacaaucuacugucuuuccA....................	3	1	seq
......................................................................uauacaaucuacugucuuuccC....................	1	1	seq
......................................................................uauacaaucuacugucuuuccuU...................	1	1	seq
.......................................................................auacaaucuacugucuuuccuU...................	1	1	seq
```

Provisional ID	:	mun-miR-196
Score total	:	113.4
Score for star read(s)	:	3.9
Score for read counts	:	103.6
Score for mfe	:	1.3
Score for randfold	:	1.6
Score for cons. seed	:	3
Total read count	:	215
Mature read count	:	191
Loop read count	:	0
Star read count	:	24

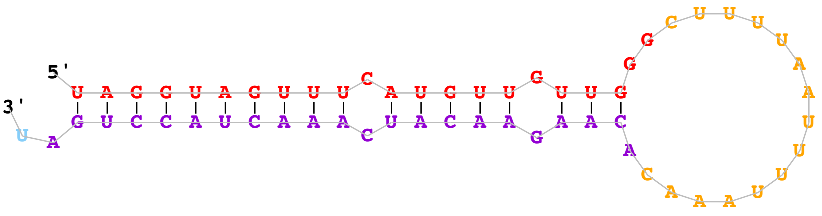

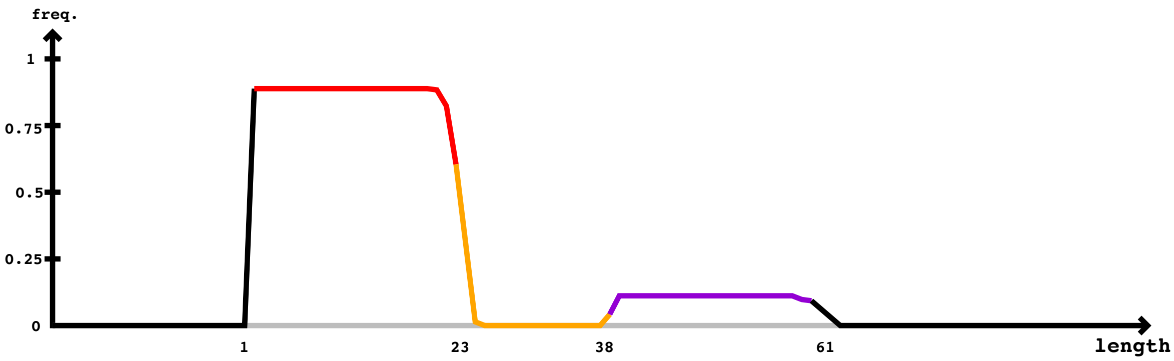

Mature **Star**

```
5'- cuagaacugcucggugaauuagguaguuucauguuguugggcuuuuaauuuuaaacacaagaacaucaaacuaccugauuuacuccaguuauccuccucgcucuguuugcug  -3'   obs
    cuagaacugcucggugaauuagguaguuucauguuguugggcuuuuaauuuuaaacacaagaacaucaaacuaccugauuuacuccaguuauccuccucgcucuguuugcug         exp
    ....(((((...(((((((((((((((((.(((((.(((...................))).))))).))))))))))))))))).))))).........((.......))..   reads   mm   sample
    ...................uagguaguuucauguuguu..........................................................................   1       0    seq
    ...................uagguaguuucauguuguug.........................................................................   13      0    seq
    ...................uagguaguuucauguuguugg........................................................................   45      0    seq
    ...................uagguaguuucauguuguuUg........................................................................   1       1    seq
    ...................uagguagCuucauguuguugg........................................................................   1       1    seq
    ...................uagguaguuucauguuguuggU.......................................................................   3       1    seq
    ...................uagguaguuucauguuguuggA.......................................................................   13      1    seq
    ...................uagguaguuucauguuguuggg.......................................................................   77      0    seq
    ...................uagguaguuucauguuguugggU......................................................................   34      1    seq
    ...................uagguaguuucauguuguugggUu.....................................................................   3       1    seq
    ........................................................acaagaacaucaaacuaccu....................................   1       0    seq
    ........................................................acaagaacaucaaacuaccuga..................................   8       0    seq
    .........................................................caagaacaucaaacuaccu....................................   2       0    seq
    .........................................................caagaacaucaaacuaccug...................................   1       0    seq
    .........................................................caagaacaucaaacuaccuga..................................   6       0    seq
    .........................................................caagaacaucaaacuaccugau.................................   5       0    seq
    .........................................................caagaacaucaaacuaccugauu................................   1       0    seq
```

Provisional ID	:	mun-miR-125-1
Score total	:	21487.8
Score for star read(s)	:	3.9
Score for read counts	:	21477.5
Score for mfe	:	1.7
Score for randfold	:	1.6
Score for cons. seed	:	3
Total read count	:	42139
Mature read count	:	35960
Loop read count	:	1
Star read count	:	6178

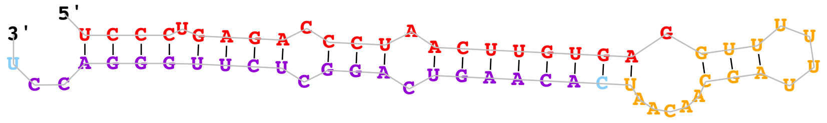

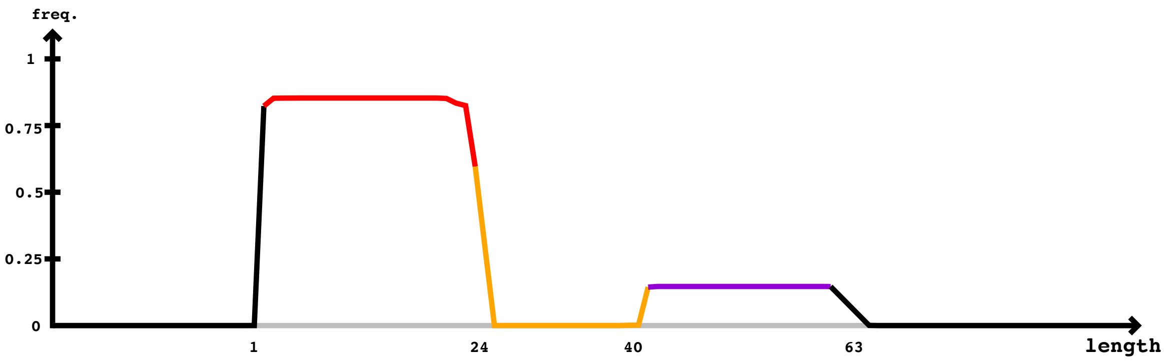

Mature Star

```
5'- ucauauuggacuuuuccuagucccugagacccuaacuugugagguuuuuuagcaacaaucacaagucaggcucuugggaccuaggcggaggggaaccagcagcuuuggacu  -3'  obs
    ucauauuggacuuuuccuagucccugagacccuaacuugugagguuuuuuagcaacaaucacaagucaggcucuugggaccuaggcggaggggaaccagcagcuuuggacu       exp
    ..........(((..(((((..((..(((.(((.((((((((.(((....)))......)))))))).))).)))..))..))))).)))....((((.....))))...  reads  mm  sample
    ucauauuggacuuuuccuag...........................................................................................  1      0   seq
    .cauauuggacuuuuccuag...........................................................................................  1      0   seq
    ...................Cucccugagacccuaacuugu.......................................................................  1      1   seq
    ...................Cucccugagacccuaacuuguga.....................................................................  2      1   seq
    ....................ucccugagacccuaaAuu.........................................................................  1      1   seq
    ....................ucccugagacUcuaacuu.........................................................................  3      1   seq
    ....................ucccugagacccuaacuuU........................................................................  23     1   seq
    ....................ucccugagacUcuaacuug........................................................................  22     1   seq
    ....................ucccugagCcccuaacuug........................................................................  1      1   seq
    ....................ucccugaUacccuaacuug........................................................................  1      1   seq
    ....................Acccugagacccuaacuug........................................................................  1      1   seq
    ....................ucccugagacccuaacuuA........................................................................  18     1   seq
    ....................ucccugagaUccuaacuug........................................................................  1      1   seq
    ....................ucccugagacccuaacCug........................................................................  1      1   seq
    ....................ucccCgagacccuaacuugu.......................................................................  4      1   seq
    ....................ucccugagacGcuaacuugu.......................................................................  1      1   seq
    ....................ucccugagaGccuaacuugu.......................................................................  3      1   seq
    ....................ucccugagGcccuaacuugu.......................................................................  3      1   seq
    ....................ucccugagacccuaacGugu.......................................................................  1      1   seq
    ....................ucccugagacccuaacuugG.......................................................................  18     1   seq
    ....................ucccugagacccuaUcuugu.......................................................................  1      1   seq
    ....................uccAugagacccuaacuugu.......................................................................  7      1   seq
    ....................ucccugagacccuGacuugu.......................................................................  1      1   seq
    ....................Ccccugagacccuaacuugu.......................................................................  4      1   seq
    ....................ucccugagaUccuaacuugu.......................................................................  17     1   seq
    ....................ucccugagacUcuaacuugu.......................................................................  312    1   seq
    ....................ucccuUagacccuaacuugu.......................................................................  3      1   seq
    ....................ucccugagaccAuaacuugu.......................................................................  2      1   seq
    ....................ucccugagacccuaacuuCu.......................................................................  1      1   seq
    ....................ucccugagacccAaacuugu.......................................................................  1      1   seq
    ....................ucccugagacccuaaAuugu.......................................................................  4      1   seq
    ....................ucccugagacccuaacuugA.......................................................................  260    1   seq
    ....................Gcccugagacccuaacuugu.......................................................................  17     1   seq
```

```
                    Mature                                       Star
ucauauuggacuuuuccuagucccugagacccuaacuugugagguuuuuuagcaacaaucacaagucaggcucuugggaccuaggcggaggggaaccagcagcuuuggacu
....................ucccugagaccGuaacuugu.......................................................................  1      1      seq
....................ucccugagacccuCacuugu.......................................................................  2      1      seq
....................Acccugagacccuaacuugu.......................................................................  9      1      seq
....................uAccugagacccuaacuugu.......................................................................  2      1      seq
....................uUccugagacccuaacuugu.......................................................................  4      1      seq
....................ucAcugagacccuaacuugu.......................................................................  4      1      seq
....................uccGugagacccuaacuugu.......................................................................  2      1      seq
....................ucccugagacccuaacuGgu.......................................................................  1      1      seq
....................ucccugagacccuaacCugu.......................................................................  2      1      seq
....................ucccugagacAcuaacuugu.......................................................................  1      1      seq
....................ucccugagaccUuaacuugu.......................................................................  31     1      seq
....................ucccugagaAccuaacuugu.......................................................................  3      1      seq
....................ucccGgagacccuaacuugu.......................................................................  1      1      seq
....................ucccugagacccuaacuuUu.......................................................................  4      1      seq
....................ucccugagacccuaacuugC.......................................................................  2      1      seq
....................ucccugagacccuaaUuugu.......................................................................  3      1      seq
....................ucccugaUacccuaacuugu.......................................................................  5      1      seq
....................Acccugagacccuaacuugug......................................................................  4      1      seq
....................ucccugagaUccuaacuugug......................................................................  1      1      seq
....................ucccugagacccuaacCugug......................................................................  2      1      seq
....................ucccuAagacccuaacuugug......................................................................  1      1      seq
....................ucccuUagacccuaacuugug......................................................................  1      1      seq
....................ucccugagacccCaacuugug......................................................................  1      1      seq
....................ucUcugagacccuaacuugug......................................................................  1      1      seq
....................ucccugagacccuaacuuguA......................................................................  115    1      seq
....................ucccugagGcccuaacuugug......................................................................  1      1      seq
....................ucccugagacUcuaacuugug......................................................................  94     1      seq
....................ucccugagacccuaaUuugug......................................................................  1      1      seq
....................ucccugagaccUuaacuugug......................................................................  1      1      seq
....................uUccugagacccuaacuugug......................................................................  6      1      seq
....................ucccugagacccuaacuuAug......................................................................  1      1      seq
....................ucccugagacAcuaacuugug......................................................................  1      1      seq
....................ucccugGgacccuaacuugug......................................................................  1      1      seq
....................ucccugagacccuaacuuguU......................................................................  119    1      seq
....................Gcccugagacccuaacuugug......................................................................  4      1      seq
....................ucccugagacccuaaAuugug......................................................................  3      1      seq
....................ucccugCgacccuaacuugug......................................................................  1      1      seq
....................ucGcugagacccuaacuugug......................................................................  1      1      seq
....................ucAcugagacccuaacuugug......................................................................  5      1      seq
....................ucccugagacccuaacuugGg......................................................................  6      1      seq
....................ucccugaCacccuaacuugug......................................................................  1      1      seq
....................ucccugaUacccuaacuugug......................................................................  2      1      seq
....................Ccccugagacccuaacuugug......................................................................  4      1      seq
....................uccAugagacccuaacuugug......................................................................  3      1      seq
....................uccUugagacccuaacuugug......................................................................  1      1      seq
....................ucccugagacccuaacuugAg......................................................................  2      1      seq
....................ucccugagacccuGacuugug......................................................................  1      1      seq
....................ucccugagacccuaacuuUuga.....................................................................  6      1      seq
....................ucccugagacccuaacuugugC.....................................................................  98     1      seq
....................ucccugagacccuaacuugCga.....................................................................  6      1      seq
....................ucccugagacccuaacuugugG.....................................................................  3977   1      seq
....................ucccugagacccuGacuuguga.....................................................................  4      1      seq
....................ucccugagacUcuaacuuguga.....................................................................  1942   1      seq
....................ucccugagacccuaacuuguCa.....................................................................  3      1      seq
....................ucccugagacccuaaAuuguga.....................................................................  3      1      seq
....................ucccugagaUccuaacuuguga.....................................................................  3      1      seq
....................ucccugagacccuaacuuCuga.....................................................................  3      1      seq
....................Gcccugagacccuaacuuguga.....................................................................  17     1      seq
....................ucUcugagacccuaacuuguga.....................................................................  1      1      seq
....................ucccugaUacccuaacuuguga.....................................................................  4      1      seq
....................uccAugagacccuaacuuguga.....................................................................  4      1      seq
....................ucccugagacAcuaacuuguga.....................................................................  4      1      seq
....................Ccccugagacccuaacuuguga.....................................................................  9      1      seq
....................ucccugagacccuaGcuuguga.....................................................................  3      1      seq
....................ucGcugagacccuaacuuguga.....................................................................  2      1      seq
....................ucccugagacccuaacuuguUa.....................................................................  10     1      seq
....................ucccugagacccuaacCuguga.....................................................................  4      1      seq
....................ucccugagacccuaacAuguga.....................................................................  1      1      seq
....................ucccugagaccAuaacuuguga.....................................................................  8      1      seq
....................ucccugaAacccuaacuuguga.....................................................................  1      1      seq
```

```
                    Mature                                      Star
ucauauuggacuuuuccuagucccugagacccuaacuugugagguuuuuuagcaacaaucacaagucaggcucuugggaccuaggcggaggggaaccagcagcuuuggacu
....................ucccugagacccuaaUuuguga.....................................................................   3       1   seq
....................ucccCgagacccuaacuuguga.....................................................................   5       1   seq
....................ucAcugagacccuaacuuguga.....................................................................   1       1   seq
....................uGccugagacccuaacuuguga.....................................................................   1       1   seq
....................uccGugagacccuaacuuguga.....................................................................   2       1   seq
....................ucccugagGcccuaacuuguga.....................................................................   5       1   seq
....................ucccugagacccuaUcuuguga.....................................................................   2       1   seq
....................ucccuAagacccuaacuuguga.....................................................................   4       1   seq
....................uAccugagacccuaacuuguga.....................................................................   2       1   seq
....................uccUugagacccuaacuuguga.....................................................................   3       1   seq
....................ucccugagUcccuaacuuguga.....................................................................   2       1   seq
....................ucccugagacccuaacuugGga.....................................................................   16      1   seq
....................uUccugagacccuaacuuguga.....................................................................   13      1   seq
....................Acccugagacccuaacuuguga.....................................................................   17      1   seq
....................ucccugagacccCaacuuguga.....................................................................   5       1   seq
....................ucccuUagacccuaacuuguga.....................................................................   2       1   seq
....................ucccugagacccuaacuuguAa.....................................................................   343     1   seq
....................ucccugagCcccuaacuuguga.....................................................................   1       1   seq
....................ucccugagacccuaacuugAga.....................................................................   4       1   seq
....................ucccugagacccuaacuugugU.....................................................................   2776    1   seq
....................ucccugGgacccuaacuuguga.....................................................................   3       1   seq
....................ucccugCgacccuaacuuguga.....................................................................   1       1   seq
....................ucccAgagacccuaacuuguga.....................................................................   3       1   seq
....................ucccugagaccUuaacuuguga.....................................................................   24      1   seq
....................ucccugagaAccuaacuuguga.....................................................................   4       1   seq
....................ucccuCagacccuaacuuguga.....................................................................   1       1   seq
....................ucccugagacccuaacuuguAag....................................................................   1       1   seq
....................ucccugagacccuaacuugugUg....................................................................   7       1   seq
....................ucccugagacccuaacuugugaA....................................................................   23916   1   seq
....................ucccugagacccuaacuugugagU...................................................................   35      1   seq
....................ucccugagacccuaacuugugaAg...................................................................   154     1   seq
....................ucccugagacccuaacuugugagA...................................................................   18      1   seq
....................ucccugagacccuaacuugugaAgu..................................................................   3       1   seq
.....................cccugagCcccuaacuugu.......................................................................   1       1   seq
.....................cccugagacccuaacuGgu.......................................................................   1       1   seq
.....................cccugagacccuaacuugA.......................................................................   1       1   seq
.....................cccugagacUcuaacuugu.......................................................................   2       1   seq
.....................Uccugagacccuaacuugu.......................................................................   1       1   seq
.....................Uccugagacccuaacuuguga.....................................................................   3       1   seq
.....................cccuCagacccuaacuuguga.....................................................................   1       1   seq
.....................cccugagacccuaaUuuguga.....................................................................   2       1   seq
.....................cccugagacccuaacCuguga.....................................................................   1       1   seq
.....................cccugagaccUuaacuuguga.....................................................................   2       1   seq
.....................cccugagacUcuaacuuguga.....................................................................   30      1   seq
.....................Gccugagacccuaacuuguga.....................................................................   2       1   seq
.....................cccugagacccuaacuugugU.....................................................................   5       1   seq
.....................cccugagacccuaacuugugG.....................................................................   209     1   seq
.....................cccugagacccAaacuuguga.....................................................................   1       1   seq
.....................Accugagacccuaacuuguga.....................................................................   1       1   seq
.....................cccuUagacccuaacuuguga.....................................................................   1       1   seq
.....................cccugagacccuaacuugugaA....................................................................   959     1   seq
.....................cccugagacccuaacuugugaAg...................................................................   10      1   seq
.....................cccugagacccuaacuugugagA...................................................................   2       1   seq
......................ccugagacccuaacuugugG.....................................................................   7       1   seq
......................ccugagacUcuaacuuguga.....................................................................   1       1   seq
......................ccugagacccuaacuuguAa.....................................................................   1       1   seq
......................ccugagacccuaacuugugU.....................................................................   1       1   seq
......................ccugagacccuaacuugugaA....................................................................   11      1   seq
.......................cugagacccuaacuugugG.....................................................................   1       1   seq
.......................cugagacccuaacuugugaA....................................................................   2       1   seq
........................ugagacccuaacuugugG.....................................................................   7       1   seq
........................ugagacccuaacuugugU.....................................................................   1       1   seq
........................ugagacccuaacuugugaA....................................................................   6       1   seq
..........................................gguuuuuuagcaacaauc...................................................   1       0   seq
..........................................................ucacaagucaggcucuuggga................................   1       0   seq
..........................................................ucacaagucaggcucuugggac...............................   7       0   seq
..........................................................ucacaagucaggcucuugggacA..............................   1       1   seq
..........................................................ucacaagucaggcucuugggacU..............................   35      1   seq
..........................................................ucacaagucaggcucuugggacc..............................   1       0   seq
...........................................................cacaagucaggcucuugggac...............................   2       0   seq
```

```
                    Mature                                  Star
ucauauuggacuuuuccuagucccugagacccuaacuugugagguuuuuuagcaacaaucacaagucaggcucuugggaccuaggcggaggggaaccagcagcuuuggacu
...........................................................cacaagucaggcucuugggacA..............................  1     1  seq
...........................................................cacaagucaggcucuugggacc..............................  1     0  seq
...........................................................cacaagucaggcucuugggacU..............................  18    1  seq
............................................................acaagucaggcucuuggg.................................  2     0  seq
............................................................acaagucaggcucuuggga................................  9     0  seq
............................................................acaagucaggcucuugggac...............................  165   0  seq
............................................................acaagucaggcucuugggaA...............................  10    1  seq
............................................................acaagucaggcucuCgggac...............................  1     1  seq
............................................................acaagucaggcucuugggaU...............................  5     1  seq
............................................................acaagucaggcucuugCgacc..............................  1     1  seq
............................................................acaagucaggcucuugggacc..............................  942   0  seq
............................................................acaagucaggcucuugggacU..............................  2198  1  seq
............................................................Ccaagucaggcucuugggacc..............................  1     1  seq
............................................................acaagGcaggcucuugggacc..............................  1     1  seq
............................................................acaagAcaggcucuugggacc..............................  2     1  seq
............................................................acaagucaggcucuugggacG..............................  7     1  seq
............................................................acaagucaggcucuugggacA..............................  63    1  seq
............................................................Gcaagucaggcucuugggacc..............................  3     1  seq
............................................................Gcaagucaggcucuugggaccu.............................  2     1  seq
............................................................acaagucGggcucuugggaccu.............................  1     1  seq
............................................................acaagucaggcucuugggaUcu.............................  1     1  seq
............................................................Ucaagucaggcucuugggaccu.............................  1     1  seq
............................................................acaagGcaggcucuugggaccu.............................  2     1  seq
............................................................acaagCcaggcucuugggaccu.............................  1     1  seq
............................................................acaagucaggcucuugCgaccu.............................  1     1  seq
............................................................acaagucaggcucuugggaccG.............................  49    1  seq
............................................................acaagucaggAucuugggaccu.............................  1     1  seq
............................................................acaagucaUgcucuugggaccu.............................  1     1  seq
............................................................acaagucaggcucuugggaccu.............................  1833  0  seq
............................................................Ccaagucaggcucuugggaccu.............................  1     1  seq
............................................................acaagucaggcucuugggacUu.............................  12    1  seq
............................................................acaagucaggcucuugggaccA.............................  50    1  seq
............................................................acaagucaggcucuugggaccC.............................  1     1  seq
............................................................acaagucaggcucuugggaccuC............................  9     1  seq
............................................................acaagucaggcucuugggaccua............................  46    0  seq
............................................................acaagucaggcucuugggaccuU............................  565   1  seq
............................................................acaagucaggcucuugggaccuaC...........................  1     1  seq
............................................................acaagucaggcucuugggaccuaU...........................  24    1  seq
.............................................................caagucaggcucuugggaA...............................  1     1  seq
.............................................................caagucaggcucuugggac...............................  2     0  seq
.............................................................caagucaggcucuugggacU..............................  4     1  seq
.............................................................caagucaggcucuugggacc..............................  21    0  seq
.............................................................caagucaggcucuugggaccG.............................  2     1  seq
.............................................................caagucaggcucuugggaccA.............................  1     1  seq
.............................................................caagucaggcucuugggaccu.............................  53    0  seq
.............................................................caagucaggcucuugggaccuU............................  13    1  seq
.............................................................caagucaggcucuugggaccua............................  1     0  seq
..............................................................aagucaggcucuugggacU..............................  1     1  seq
```

Provisional ID	:	mun-let-7c
Score total	:	3498.8
Score for star read(s)	:	3.9
Score for read counts	:	3488.3
Score for mfe	:	2
Score for randfold	:	1.6
Score for cons. seed	:	3
Total read count	:	6854
Mature read count	:	6848
Loop read count	:	1
Star read count	:	5

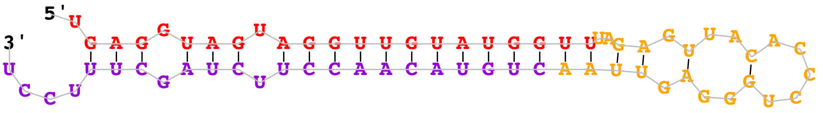

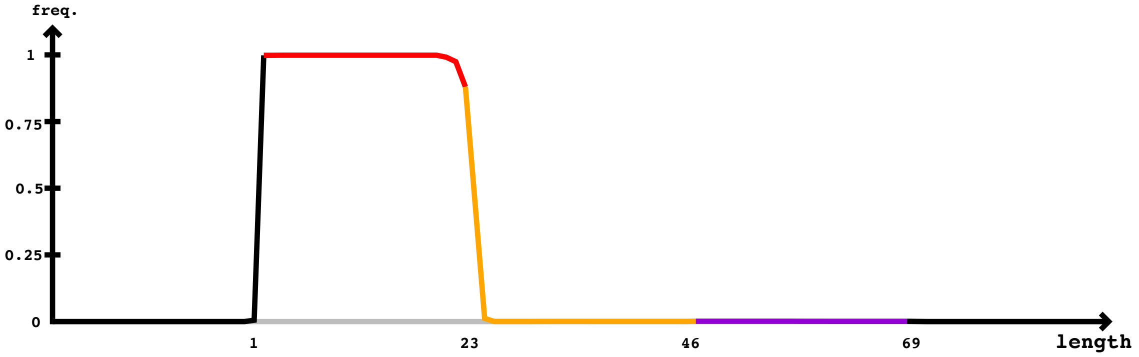

Mature Star

```
5'- gaagcugugugcauccggguugagguaguagguuguaugguuuagaguuacacccugggaguuaacuguacaaccuucuagcuuuccuuggagcacacuugagccauc  -3'  obs
    gaagcugugugcauccggguugagguaguagguuguaugguuuagaguuacacccugggaguuaacuguacaaccuucuagcuuuccuuggagcacacuugagccauc       exp
    ...(((((((((.((((((..(((.(((.(((((((((((((..((.(..(.....)..).))))))))))))))).))).)))..))))))))))))...))).... reads  mm  sample
    ...................uugagguaguagguuguaug..................................................................... 1     0   seq
    ...................uugagguaguagguuguaugg.................................................................... 1     0   seq
    ...................uugagguaguagguuguauggu................................................................... 14    0   seq
    ...................uugagguaguagguuguaugguu.................................................................. 16    0   seq
    ...................Augagguaguagguuguaugguu.................................................................. 1     1   seq
    ...................uugagguaguagguuguaugguuu................................................................. 2     0   seq
    ....................ugagguaguagguuguaug..................................................................... 12    0   seq
    ....................ugagguaguagguuguauU..................................................................... 37    1   seq
    ....................ugagguaguagguuguaugU.................................................................... 7     1   seq
    ....................ugagguaguagguuguaugA.................................................................... 4     1   seq
    ....................ugagguaguagguuguauUg.................................................................... 1     1   seq
    ....................ugagguaguagguuguaugg.................................................................... 102   0   seq
    ....................ugagguaguagguuguauggA................................................................... 16    1   seq
    ....................ugagguaguagguuguaugAu................................................................... 1     1   seq
    ....................ugagguaguagAuuguauggu................................................................... 1     1   seq
    ....................ugagguaguaggGuguauggu................................................................... 1     1   seq
    ....................ugagguaguGgguuguauggu................................................................... 1     1   seq
    ....................ugaggGaguagguuguauggu................................................................... 2     1   seq
    ....................ugagguaguagguuguauggu................................................................... 611   0   seq
    ....................ugaggAaguagguuguauggu................................................................... 2     1   seq
    ....................ugagguaguagguuguaugCu................................................................... 1     1   seq
    ....................ugagguaguagguuguaugguu.................................................................. 4767  0   seq
    ....................ugagUuaguagguuguaugguu.................................................................. 1     1   seq
    ....................ugagguaguagguuguaugguA.................................................................. 78    1   seq
    ....................ugaggGaguagguuguaugguu.................................................................. 10    1   seq
    ....................ugagguaguagguuUuaugguu.................................................................. 3     1   seq
    ....................ugagguaguagguuguaugguG.................................................................. 3     1   seq
    ....................Ggagguaguagguuguaugguu.................................................................. 2     1   seq
    ....................ugGgguaguagguuguaugguu.................................................................. 1     1   seq
    ....................ugagguaguaggCuguaugguu.................................................................. 1     1   seq
    ....................ugagguaguagguuguauggAu.................................................................. 2     1   seq
    ....................ugagguaguagguuAuaugguu.................................................................. 1     1   seq
    ....................ugaggCaguagguuguaugguu.................................................................. 1     1   seq
```

Mature   Star

```
gaagcugugugcauccggguugagguaguagguuguaugguuuagaguuacacccugggaguuaacuguacaaccuucuagcuuuccuuggagcacacuugagccauc
....................ugagguaguGgguuguaugguu..................................................................   3    1   seq
....................ugaggAaguagguuguaugguu..................................................................   5    1   seq
....................ugagguaguagCuuguaugguu..................................................................   2    1   seq
....................ugagguaguagguuguauggCu..................................................................   2    1   seq
....................Agagguaguagguuguaugguu..................................................................   4    1   seq
....................uUagguaguagguuguaugguu..................................................................   1    1   seq
....................ugagguaUuagguuguaugguu..................................................................   1    1   seq
....................ugagguaguagAuuguaugguu..................................................................   2    1   seq
....................ugagguaguagguuguaugguC..................................................................   1    1   seq
....................ugagguGguagguuguaugguu..................................................................   3    1   seq
....................ugagguaguaUguuguaugguu..................................................................   1    1   seq
....................ugagCuaguagguuguaugguu..................................................................   1    1   seq
....................ugagguaguagguuguUugguu..................................................................   1    1   seq
....................ugaUguaguagguuguaugguu..................................................................   1    1   seq
....................ugagguaguaggAuguaugguu..................................................................   1    1   seq
....................ugagguaguagguuguaugUuu..................................................................   1    1   seq
....................ugagguaguagguuguaugAuu..................................................................   1    1   seq
....................Cgagguaguagguuguaugguu..................................................................   4    1   seq
....................ugagguaguagguuguauCguu..................................................................   1    1   seq
....................ugagguaguagguuguaugguuG.................................................................   25   1   seq
....................ugagguaguagguuguaugguuC.................................................................   1    1   seq
....................ugagguaguagguuguauUguuu.................................................................   1    1   seq
....................ugagguaguagguuguaugguGu.................................................................   1    1   seq
....................ugagUuaguagguuguaugguuu.................................................................   1    1   seq
....................ugagguaguagguuguauggGuu.................................................................   2    1   seq
....................ugagguaguagguuguauCguuu.................................................................   2    1   seq
....................ugagguaguagguuUuaugguuu.................................................................   1    1   seq
....................ugagguaguagguuguaugguAu.................................................................   33   1   seq
....................ugagguaguagguuguaugguuA.................................................................   122  1   seq
....................ugagguCguagguuguaugguuu.................................................................   1    1   seq
....................ugagguaguaUguuguaugguuu.................................................................   1    1   seq
....................Ggagguaguagguuguaugguuu.................................................................   2    1   seq
....................ugagguaguagguuguaugguuu.................................................................   833  0   seq
....................ugagguaguagguuguaugguuua................................................................   4    0   seq
....................ugagguaguagguuguaugguuuG................................................................   1    1   seq
....................ugagguaguagguuguGugguuua................................................................   7    1   seq
....................ugagguaguagguuguaugguuAa................................................................   2    1   seq
....................ugagguaguagguuguaugguuuU................................................................   64   1   seq
....................ugagguaguagguuguaugguuuC................................................................   1    1   seq
....................ugagguaguagguuguaugguuuagaguuacU........................................................   1    1   seq
....................ugagguaguagguuguaugguuuagaguuacacU......................................................   1    1   seq
.....................gaggGaguagguuguaugguu..................................................................   1    1   seq
......................agguaguagguuguaugguu..................................................................   1    0   seq
......................agguaguagguuguaugguuu.................................................................   1    0   seq
..........................................uagaguuacacccugggaguuaU...........................................   1    1   seq
.................................................acacccugggaguuaacuguacaaccuucuagcuuuccu....................   1    0   seq
...................................................acccugggaguuaacuguacaaccuucu.............................   1    0   seq
....................................................cccugggaguuaacuguacaaccu................................   1    0   seq
.................................................................cuguacaaccuucuagcuuuccA....................   1    1   seq
.................................................................cuguacaaccuucuagcuuuccu....................   4    0   seq
```

Provisional ID	:	mun-miR-338-2
Score total	:	5
Score for star read(s)	:	3.9
Score for read counts	:	-1.8
Score for mfe	:	2
Score for randfold	:	1.6
Score for cons. seed	:	-0.6
Total read count	:	8
Mature read count	:	7
Loop read count	:	0
Star read count	:	1

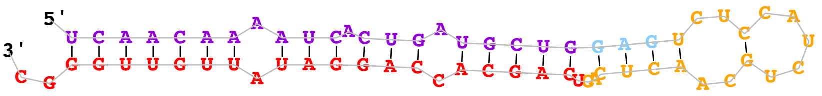

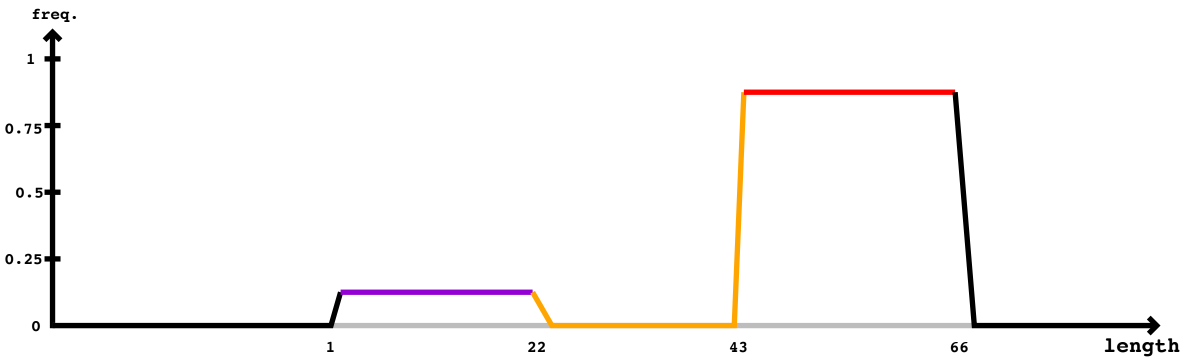

Star Mature

```
5'- uggcccccgaugucaaugcacccccuccucaacaaaaucacugaugcuggagucuccaucugcaacucacucagcaccaggauauuguugggcaggaggagcaguggugggggc   -3'   obs
    uggcccccgaugucaaugcacccccuccucaacaaaaucacugaugcuggagucuccaucugcaacucacucagcaccaggauauuguugggcaggaggagcaguggugggggc         exp
    ..((((.....(((.(((.((.(((.((((((((.(((.(((.((((((((..(.....)..))))...))))).))))))).)))))))).))).)).))).))).)))))    reads   mm   sample
    ............................ucaacaaaaucacugaugcug................................................................    1       0    seq
    .......................................................................ucagcaccaggauauuguugggc....................    7       0    seq
```

Provisional ID	:	mun-novel43
Score total	:	0
Score for star read(s)	:	-1.3
Score for read counts	:	0
Score for mfe	:	0.3
Score for randfold	:	1.6
Score for cons. seed	:	-0.6
Total read count	:	650
Mature read count	:	520
Loop read count	:	0
Star read count	:	130

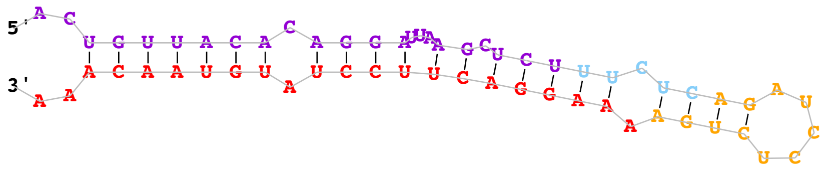

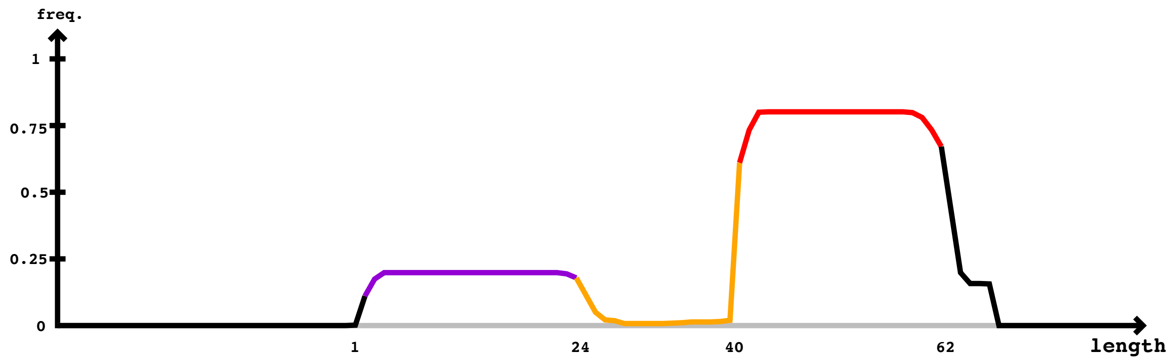


```
                                Star                                    Mature
5'- caguugcaguauucccucuguggcaaauaaacuguuacacaggauuaagcucuuucucagauccucugaaaaggacuuccuauguaacaaacaacauaagcuuuuuaaucu  -3'   obs
    caguugcaguauucccucuguggcaaauaaacuguuacacaggauuaagcucuuucucagauccucugaaaaggacuuccuauguaacaaacaacauaagcuuuuuaaucu        exp
    ..((..(((........)))..))........((((((.((((...((.(((((.((((.....)))).))))))))))).)))))))........................  reads   mm   sample
    .............................aacuguuacacaggauuaagcucu..........................................................  1       0    seq
    ..............................acuguuacacaggauuaagcu............................................................  1       0    seq
    ..............................acuguuacacaggauuaagcuc...........................................................  10      0    seq
    ..............................acuguuacacaggauuaagcucu..........................................................  49      0    seq
    ..............................acuguuacacaggauuaagcucuu.........................................................  9       0    seq
    ..............................acuguuacacaggauuaagcucuuu........................................................  3       0    seq
    ...............................cuguuacacaggauuaagcu............................................................  2       0    seq
    ...............................cuguuacacaggauuaagcucu..........................................................  18      0    seq
    ...............................cuguuacacaggauuaagcucuu.........................................................  5       0    seq
    ...............................cuguuacacaggauuaagcucuuu........................................................  12      0    seq
    ...............................cuguuacacaggauuaagcucuuucu......................................................  5       0    seq
    ................................uguuacacaggauuaagcucu..........................................................  3       0    seq
    ................................uguuacacaggauuaagcucuuu........................................................  5       0    seq
    ................................uguuacacaggauuaagcucuuuc.......................................................  4       0    seq
    ................................uguuacacaggauuaagcucuuucu......................................................  3       0    seq
    ................................uguuacacaggauuaagcucuuucucagauc................................................  1       0    seq
    .......................................................cucagauccucugaaaaggacuuccuauguaacaaCc...................  1       1    seq
    ........................................................ucagauccucugaaaaggacuuccuauguaaca......................  1       0    seq
    ........................................................ucagauccucugaaaaggacuuccuauguaacaaacaac................  1       0    seq
    .........................................................cagauccucugaaaaggacuuccuauguaacaaU....................  1       1    seq
    ..............................................................ccucugaaaaggacuuccuauguaacaaacaaca...............  1       0    seq
    ...............................................................cucugaaaaggacuuccuauguaacaaacaaca...............  2       0    seq
    ................................................................ucugaaaaggacuuccuauguaacaaacaaca...............  2       0    seq
    ...................................................................gaaaggacuuccuauguaacaaacU...................  1       1    seq
    ....................................................................aaaaggacuuccuauguaacU......................  1       1    seq
    ....................................................................Caaaggacuuccuauguaacaa....................  1       1    seq
    ....................................................................aaaaggacuuccuauguaacaaacaaca...............  1       0    seq
    .....................................................................aaaggacuuccuauguaa........................  2       0    seq
    .....................................................................aaaggacuuccuauguaaA.......................  1       1    seq
    .....................................................................aaaggacuuccuauguaac.......................  11      0    seq
    .....................................................................aaaggacuuccuauguaaca......................  23      0    seq
    .....................................................................aaaggacuuccuauguaacU......................  5       1    seq
    .....................................................................aaaggacuuccuauguaaGa......................  1       1    seq
```

Star Mature

caguugcaguauucccucuguggcaaauaaacuguuacacaggauuaagcucuuucucagauccucugaaaaggacuuccuauguaacaaacaacauaagcuuuuuaaucu			
....aaaggacuuccuauguaacaa.....................	39	0	seq
....aaaggacuuccuGuguaacaa.....................	1	1	seq
....aaaggacuuccuauguaacaaC....................	8	1	seq
....aaaggacuuccuauguaacaaU....................	203	1	seq
....aaaggacuuccuauguaacaaa....................	39	0	seq
....aaaggacuuccuauguaacaaCc...................	27	1	seq
....aaaggacuuccuauguaacaaac...................	5	0	seq
....aaaggacuuccuauguaacaaaA...................	1	1	seq
....aaaggacuuccuauguaacaaaU...................	21	1	seq
....aaaggacuuccuauguaacaaacU..................	1	1	seq
....aaaggacuuccuauguaacaaacC..................	1	1	seq
....aaaggacuuccuGuguaacaaacaaca...............	1	1	seq
....aaggacuuccuauguaacaaaU...................	6	1	seq
....aaggacuuccuauguaacaaacC..................	1	1	seq
....Caggacuuccuauguaacaaacaaca...............	1	1	seq
....aaggacuuccuauguaacaaacaaca...............	73	0	seq
....aggacuuccuauguaacaaacC..................	12	1	seq
....agCacuuccuauguaacaaaca..................	1	1	seq
....aggacuuccuauguaacaaacU..................	10	1	seq
....aggacuuccuauguaacaaacaaca...............	20	0	seq
....aggacuAccuauguaacaaacaaca...............	1	1	seq
....ggacuuccuauguaacaaacaaca...............	1	0	seq

Provisional ID	:	mun-novel90
Score total	:	1.1
Score for star read(s)	:	-1.3
Score for read counts	:	0
Score for mfe	:	1.4
Score for randfold	:	1.6
Score for cons. seed	:	-0.6
Total read count	:	354
Mature read count	:	346
Loop read count	:	7
Star read count	:	1

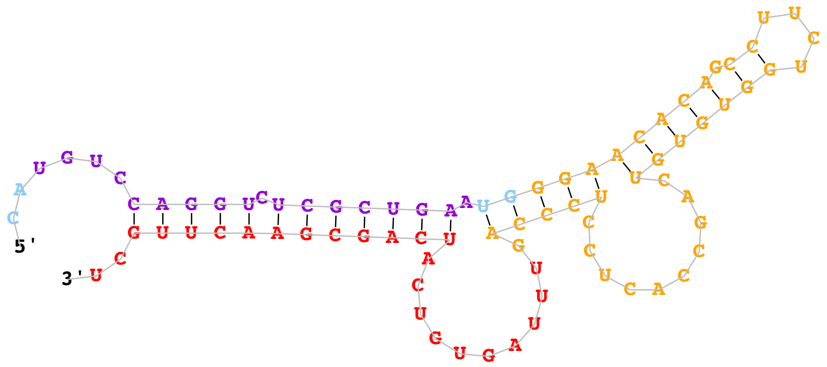

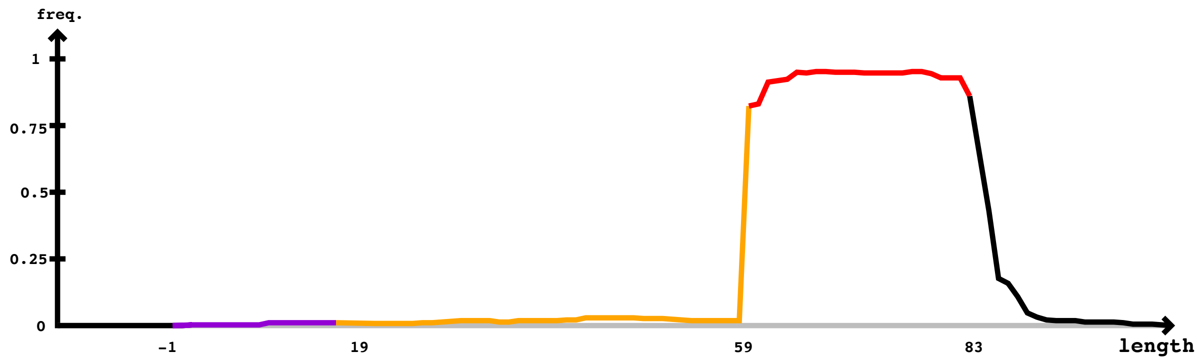


```
                Star                                        Mature
5'- aacucuccagcauguccaggucucgcugaaugggaacacagccuucuggugugucagccacuccucccaguuuagugucaucagcgaacuugcugaggguacacucaguucccu  -3'   obs
    aacucuccagcauguccaggucucgcugaaugggaacacagccuucuggugugucagccacuccucccaguuuagugucaucagcgaacuugcugaggguacacucaguucccu         exp
    .((((((.((((..(((.(((..((((((((((((((((.((....)))))))..........))))).))))))))..))).).))...))))))))))..............    reads   mm   sample
    ............uguccaggucucgcugaa....................................................................................    1       0    seq
    ....................ucucgcugaaugggaacacagccu......................................................................    3       0    seq
    ....................................cacagccuucuggugugucagccacuccucccaguuuagu......................................    1       0    seq
    ......................................cagccuucuggugugucagccacucc..................................................    1       0    seq
    .......................................agccuucuggugugucagccacu....................................................    1       0    seq
    ........................................gccuucuggugugucagcc.......................................................    1       0    seq
    ............................................ucuggugugucagccacuc...................................................    1       0    seq
    ..............................................uggugugucagccacuccuccc..............................................    1       0    seq
    ..............................................uggugugucGgccacuccucccagu...........................................    1       1    seq
    ...................................................ugucagccacuccucccaguuCaguguc...................................    1       1    seq
    .....................................................ucagccacuccucccagu...........................................    1       0    seq
    .....................................................ucagccacuccucccaguuuaguguca..................................    1       0    seq
    .....................................................ucagccacuccucccaguuuagugucauc................................    1       0    seq
    ....................................................................aguuuagugucaucagcgaacuugcu....................    1       0    seq
    ......................................................................uuCagugucaucagcgaac.........................    2       1    seq
    ......................................................................uuuagugucaucagcgaac.........................    1       0    seq
    ......................................................................uuCagugucaucagcgaacu........................    3       1    seq
    ......................................................................uuuagugucaucagcgaacu........................    5       0    seq
    ......................................................................uuuagugucaucagcgaacuugc.....................    11      0    seq
    ......................................................................uuuagugucaucagcAaacuugc.....................    3       1    seq
    ......................................................................uuuagugucCucagcgaacuugc.....................    1       1    seq
    ......................................................................uuuagugucaucaUcgaacuugcu....................    1       1    seq
    ......................................................................uuCagugucaucagcgaacuugcu....................    8       1    seq
    ......................................................................uuuagugucaucagUgaacuugcu....................    44      1    seq
    ......................................................................uuuagugucaAcagcgaacuugcu....................    1       1    seq
    ......................................................................uCuagugucaucagcgaacuugcu....................    1       1    seq
    ......................................................................Cuuagugucaucagcgaacuugcu....................    1       1    seq
    ......................................................................uuuagugucauGagcgaacuugcu....................    1       1    seq
    ......................................................................uuuagugucaucagcgaacuuCcu....................    1       1    seq
    ......................................................................uuuagugucaucagcCaacuugcu....................    1       1    seq
    ......................................................................uuuagugucaucagcgaacuuAcu....................    8       1    seq
    ......................................................................uuuagugucauUagcgaacuugcu....................    1       1    seq
    ......................................................................uuuagugucaucagcgaacuugcu....................    67      0    seq
```

Star / Mature			
aacucuccagcauguccaggucucgcugaaugggaacacagccuucuggugugucagccacuccucccaguuuagugucaucagcgaacuugcugaggguacacucaguucccu			
....uuuagugucaucagcgaacuGgcu....................	2	1	seq
....uuuagugucaucagAgaacuugcu....................	1	1	seq
....uuuagugucaucagcAaacuugcug...................	5	1	seq
....uuuagugucaucagcgaacuuAcug...................	1	1	seq
....uuuagugucaucagcCaacuugcug...................	1	1	seq
....uuuagugucaucagcgaGcuugcug...................	1	1	seq
....uuuagugucaucagcgaacuugcug...................	5	0	seq
....uuuagugucaucagUgaacuugcug...................	4	1	seq
....uuuagugucaucagcgaacuugcugU..................	7	1	seq
....uuuagugucaucagAgaacuugcuga..................	7	1	seq
....uuuagugucaucGgcgaacuugcuga..................	1	1	seq
....uuuagugucaucagUgaacuugcuga..................	52	1	seq
....uuuagugucaucagcgaacuugcuga..................	7	0	seq
....uuuagugucaucagcgaacuugcugC..................	1	1	seq
....uuuagugucaucagcgaacuuAcuga..................	9	1	seq
....uuuagugucaucaAcgaacuugcuga..................	1	1	seq
....uuCagugucaucagcgaacuugcuga..................	1	1	seq
....uuuagugucaucagcAaacuugcuga..................	1	1	seq
....uuuagugucaucagcCaacuugcuga..................	1	1	seq
....uuuagugucaucagUgaacuugcugag.................	1	1	seq
....uuuagugucaucagcAaacuugcugag.................	1	1	seq
....uuuagugucaucagcgaacuugcugCg.................	1	1	seq
....uuuagugucaucagcgaacuugcugag.................	2	0	seq
....uuuagugucaucagcgaacuuAcugagg................	2	1	seq
....uuuagugucaucagcgaacuugcugagg................	3	0	seq
....uuCagugucaucagcgaacuugcugagg................	1	1	seq
....uuuagugucaucagUgaacuugcugagg................	6	1	seq
....uuuagugucaucagAgaacuugcugaggg...............	2	1	seq
....uuuagugucaAcagcgaacuugcugaggg...............	1	1	seq
....uuuagugucaucagcgaacuugcugaggg...............	4	0	seq
....uuuagugucaucagUgaacuugcugaggg...............	7	1	seq
....uuuagugucaucagcgaacuuAcugaggg...............	2	1	seq
....uuuagugucaucagUgaacuugcugagggu..............	2	1	seq
....uuuagugucaucagcgaacuugcugagggu..............	1	0	seq
....uuagugucaucagcgaacu........................	1	0	seq
....uuagugucaucagcgaacuugc.....................	1	0	seq
....uuagugucaucagcgaacuugcu....................	1	0	seq
....uuagugucaucagcgaacuugcugag.................	1	0	seq
....uuagugucaucagUgaacuugcugaggg...............	1	1	seq
....Cagugucaucagcgaacuugc.....................	1	1	seq
....uagugucaucagUgaacuugcu....................	1	1	seq
....uagugucaucagcAaacuugcu....................	1	1	seq
....uagugucaucagUgaacuugcug...................	2	1	seq
....uagugucaucagcgaacuugcuga..................	1	0	seq
....uagugucaucagUgaacuugcuga..................	6	1	seq
....uagugucaucagUgaacuugcugagg................	2	1	seq
....uagugucaucagcgaacuugcugagg................	2	0	seq
....uagugucaucagcgaacuugcugagA................	2	1	seq
....uagugucaucagcgaacuugcugaggg...............	2	0	seq
....uagugucaucagUgaacuugcugaggg...............	4	1	seq
....uagugucaucagUgaacuugcugagggu..............	1	1	seq
....uagugucaucagcgaacuuAcugagggu..............	1	1	seq
....uagugucaucagcgaacuuAcugagggua.............	2	1	seq
....uagugucaucagAgaacuugcugagggua.............	1	1	seq
....uagugucaucagUgaacuugcugagggua.............	1	1	seq
....uagugucaucagcgaacuuAcugaggguac............	1	1	seq
....agugucaucagcgaacuugc.....................	1	0	seq
....agugucaucagcAaacuugcugag.................	1	1	seq
....gugucaucagcgaacuugc.....................	2	0	seq
....ugucaucCgcgaacuugc.....................	1	1	seq
....ugucaucagcgaacuugc.....................	5	0	seq
....ugucaucagcgaacuugcu....................	2	0	seq
....ugucaucGgcgaacuugcuga..................	1	1	seq
....ugucaucGgcgaacuugcugagg................	1	1	seq
....ucaucagcgaacuugcugagggC..............	1	1	seq
....ucaucagcgaacuugcugagCguacacucaguuccc.	1	1	seq
....ucagcgaacuugcugaggguacacucGguu....	1	1	seq
....acuugcugaggguacacu.........	2	0	seq
....ugcAgaggguacacucagu.....	1	1	seq
....ugcAgaggguacacucaguu....	1	1	seq

Star Mature

```
aacucuccagcauguccaggucucgcugaaugggaacacagccuucuggugugucagccacuccucccaguuuagugucaucagcgaacuugcugaggguacacucaguucccu
..........................................................................................ugcAgaggguacacucaguucccu    1    1    seq
```

Provisional ID	:	mun-miR-20a
Score total	:	5.3
Score for star read(s)	:	-1.3
Score for read counts	:	0
Score for mfe	:	2
Score for randfold	:	1.6
Score for cons. seed	:	3
Total read count	:	3635
Mature read count	:	3622
Loop read count	:	0
Star read count	:	13

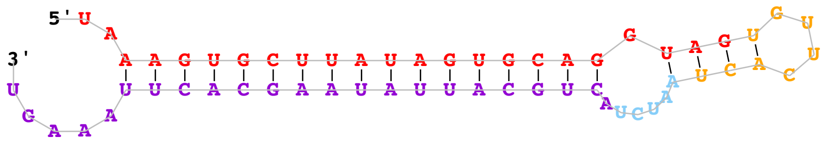

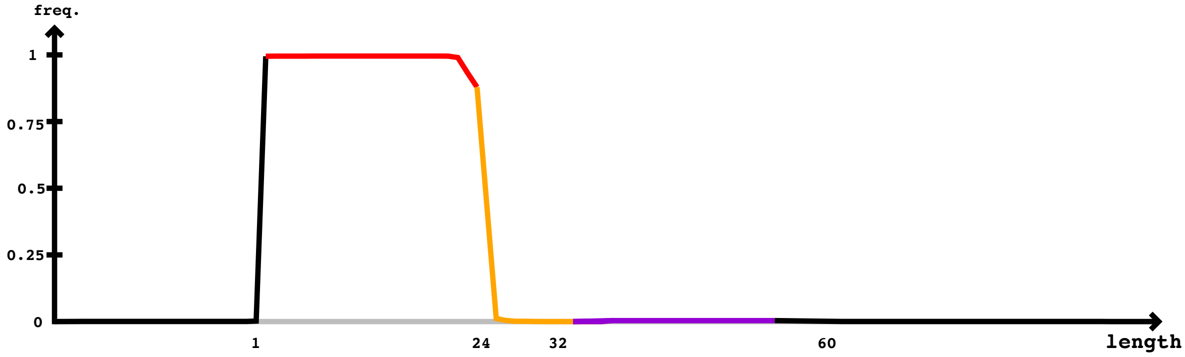

Mature **Star**

```
5'- aaugacagcucuuguagcacuaaagugcuuauagugcagguaguguucacuaaaucuacugcauuauaagcacuuaaaguacugcuagcuguagaacuacauguucagcaugcc -3'  obs
    aaugacagcucuuguagcacuaaagugcuuauagugcagguaguguucacuaaucuacugcauuauaagcacuuaaaguacugcuagcuguagaacuacauguucagcaugcc       exp
    ....((((((...((((.(((.(((((((((((((((((.((((....)))).....)))))))))))))))))..))).)))).)))))).((((.....)))).........   reads  mm  sample
    aaugacagcucuuguagcac..............................................................................................   1      0   seq
    .augacagcucuuguagcac..............................................................................................   1      0   seq
    ...................cuaaagugcuuauagugcaggu.........................................................................   3      0   seq
    ...................cuaaagugcuuauagugcagguaU.......................................................................   2      1   seq
    ....................uaaagugcuuauagugcag...........................................................................   1      0   seq
    ....................uaaagugcuuauagugcagA..........................................................................   1      1   seq
    ....................uaaagugcuuauagugcagU..........................................................................   1      1   seq
    ....................uaaagugcuuauagugcagg..........................................................................   17     0   seq
    ....................uaaagugcuuauagugcaggu.........................................................................   200    0   seq
    ....................Caaagugcuuauagugcaggu.........................................................................   1      1   seq
    ....................uaaagugcuuauGgugcaggu.........................................................................   1      1   seq
    ....................uaaagugcuuauagugcaggA.........................................................................   2      1   seq
    ....................uaaagugcuuauagugcagguG........................................................................   9      1   seq
    ....................uaaagugcuuauagugcaggua........................................................................   176    0   seq
    ....................uaaagugcuuauagugcaggGa........................................................................   2      1   seq
    ....................uaaagugcuuauagugcagguU........................................................................   10     1   seq
    ....................uaaagugcuuauagAgcagguag.......................................................................   1      1   seq
    ....................Gaaagugcuuauagugcagguag.......................................................................   2      1   seq
    ....................uaaagCgcuuauagugcagguag.......................................................................   1      1   seq
    ....................uaaagugcuuauagugcaggGag.......................................................................   5      1   seq
    ....................uaaagugcuuauagugcaUguag.......................................................................   1      1   seq
    ....................uaaagugcuuauagugcGgguag.......................................................................   1      1   seq
    ....................uaaagugcuuauagugcagguaA.......................................................................   50     1   seq
    ....................uGaagugcuuauagugcagguag.......................................................................   2      1   seq
    ....................uaaaUugcuuauagugcagguag.......................................................................   1      1   seq
    ....................uaaagugcuuauaguUcagguag.......................................................................   1      1   seq
    ....................uaaagugcuuauagugcagguaU.......................................................................   125    1   seq
    ....................uaaagugcuuauagugcaggCag.......................................................................   1      1   seq
    ....................uaaagugcuuauagugcagguaC.......................................................................   12     1   seq
    ....................uaaagugcuuaAagugcagguag.......................................................................   1      1   seq
    ....................uUaagugcuuauagugcagguag.......................................................................   1      1   seq
    ....................uaaagugcuuauGgugcagguag.......................................................................   2      1   seq
    ....................uaaagGgcuuauagugcagguag.......................................................................   3      1   seq
```

Mature **Star**

```
aaugacagcucuuguagcacuaaagugcuuauagugcagguaguguucacuaaaucuacugcauuauaagcacuuaaaguacugcuagcuguagaacuacauguucagcaugcc
....................uaaagugcuuauagugcagAuag....................................................................... 1 1 seq
....................uaaagugcuCauagugcagguag....................................................................... 2 1 seq
....................uaaagugcuuaCagugcagguag....................................................................... 1 1 seq
....................Aaaagugcuuauagugcagguag....................................................................... 1 1 seq
....................uaaagugcuuauagCgcagguag....................................................................... 2 1 seq
....................uaaagugcuuauagugcagguag....................................................................... 2041 0 seq
....................uaaagugcuuauagugcaggAag....................................................................... 2 1 seq
....................uaaagugcuuauagugcaggAagu...................................................................... 1 1 seq
....................Aaaagugcuuauagugcagguagu...................................................................... 1 1 seq
....................uGaagugcuuauagugcagguagu...................................................................... 1 1 seq
....................uaaagugcuuauagugcagguagu...................................................................... 791 0 seq
....................uaaagugcuuauagugcaUguagu...................................................................... 1 1 seq
....................uaaagugcuuauagugcagguaCu...................................................................... 2 1 seq
....................uaaaUugcuuauagugcagguagu...................................................................... 1 1 seq
....................uaaagugcuuauagugcagguaAu...................................................................... 1 1 seq
....................uaaagugcuuauGgugcagguagu...................................................................... 2 1 seq
....................Gaaagugcuuauagugcagguagu...................................................................... 1 1 seq
....................uaGagugcuuauagugcagguagu...................................................................... 1 1 seq
....................uaaagugcuuauagugcagguaUu...................................................................... 15 1 seq
....................uaaGgugcuuauagugcagguagu...................................................................... 1 1 seq
....................uaaagugcuuauagugcagguagA...................................................................... 73 1 seq
....................uaaagugcuuauagugcagguaguU..................................................................... 23 1 seq
....................uaaagugcuuauagugcagguagug..................................................................... 1 0 seq
....................uaaagugcuuauagugcagguaguA..................................................................... 3 1 seq
....................uaaagugcuuauagugcagguagugu.................................................................... 1 0 seq
....................uaaagugcuuauagugcagguaguAu.................................................................... 4 1 seq
....................uaaagugcuuauagugcagguaguUu.................................................................... 6 1 seq
....................uaaagugcuuauagugcagguaguguuU.................................................................. 1 1 seq
....................uaaagugcuuauagugcagguaguguuc.................................................................. 2 0 seq
....................uaaagugcuuauagugcagguaguguuca................................................................. 1 0 seq
.....................aaagugcuuauagugcagguag....................................................................... 1 0 seq
.....................aaagugcuuauagugcagguagu...................................................................... 1 0 seq
.........................ugcuCauagugcagguagu...................................................................... 1 1 seq
.........................................................acugcauuauaagcacuuaaa.................................... 1 0 seq
.........................................................acugcauuauaagcacuuaaag................................... 2 0 seq
.........................................................acugcauuauaagcacuuaaagu.................................. 10 0 seq
................................................................................acugcuagcuguagaacuacauguucagc..... 1 0 seq
```

Provisional ID	:	mun-miR-146a
Score total	:	98802.7
Score for star read(s)	:	3.9
Score for read counts	:	98792
Score for mfe	:	2.2
Score for randfold	:	1.6
Score for cons. seed	:	3
Total read count	:	193788
Mature read count	:	193356
Loop read count	:	0
Star read count	:	432

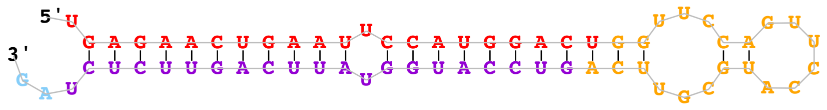

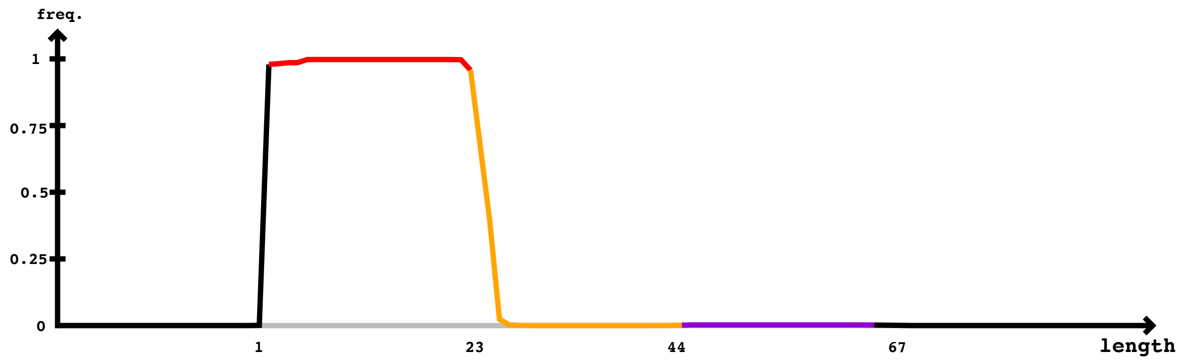

Mature **Star**

```
5'- accuuggcaguucccagcucugagaacugaauuccauggacugguuccaguuccaugcguucaguccaugguauucaguucucuagcuuggcugcaucgaacuucacaacua   -3'   obs
    accuuggcaguucccagcucugagaacugaauuccauggacugguuccaguuccaugcguucaguccaugguauucaguucucuagcuuggcugcaucgaacuucacaacua         exp
    ......((((..((.((((..(((((((((((.(((((((((((...((......))...))))))))))).))))))))))).)))).)))))).................   reads   mm   sample
    .................Aucugagaacugaauuccauggacu......................................................................   1       1    seq
    ..................ucugagaacugaauuccauggac.......................................................................   2       0    seq
    ..................ucugagaacugaauuccauggacu......................................................................   5       0    seq
    ..................ucugagaacugaauuccauggacug.....................................................................   1       0    seq
    ..................ucugagaacugaauuccauggacugU....................................................................   3       1    seq
    ..................ucugagaacugaauuccauggacugA....................................................................   1       1    seq
    ...................cugagaacugaauuccau...........................................................................   1       0    seq
    ...................cugagaacugaauuccauAga........................................................................   6       1    seq
    ...................cugagaacugaauuccauggac.......................................................................   6       0    seq
    ...................cugagaacugaauuccauggacA......................................................................   1       1    seq
    ...................cugagaacugaauuccauggacu......................................................................   46      0    seq
    ...................Gugagaacugaauuccauggacu......................................................................   3       1    seq
    ...................cugagaacugaauuccauggacug.....................................................................   16      0    seq
    ...................cugagaacugaauuccauggacugA....................................................................   5       1    seq
    ...................cugagaacugaauuccauggacugU....................................................................   29      1    seq
    ...................cugagaacugaauuccauggacugUu...................................................................   5       1    seq
    ....................ugagaacugaauuccaug..........................................................................   11      0    seq
    ....................ugagaacugaauuccaugA.........................................................................   1       1    seq
    ....................ugagaacugaauuccaugg.........................................................................   35      0    seq
    ....................ugagaacugaauuccauAga........................................................................   4       1    seq
    ....................ugagaacugaauuccaugga........................................................................   109     0    seq
    ....................ugagaacugaauuccauggaA.......................................................................   168     1    seq
    ....................ugagaacuAaauuccauggac.......................................................................   2       1    seq
    ....................ugagaaAugaauuccauggac.......................................................................   2       1    seq
    ....................Ggagaacugaauuccauggac.......................................................................   7       1    seq
    ....................ugagaacugaaAuccauggac.......................................................................   2       1    seq
    ....................ugagaacugaauuccauggac.......................................................................   7357    0    seq
    ....................ugagaacugaauuccUuggac.......................................................................   1       1    seq
    ....................ugagaacugaauucUauggac.......................................................................   6       1    seq
    ....................ugagaacugaauuccauggaG.......................................................................   2       1    seq
    ....................uUagaacugaauuccauggac.......................................................................   2       1    seq
    ....................ugagaacugaauuccauggCc.......................................................................   2       1    seq
    ....................ugagaGcugaauuccauggac.......................................................................   11      1    seq
```

Mature Star

accuuggcaguucccagcucugagaacugaauuccauggacuggguuccaguuccaugcguucaguccaugguauucaguucucuagcuuggcugcaucgaacuucacaacua

....................Cgagaacugaauuccauggac...	3	1	seq
....................ugagaacugGauuccauggac...	2	1	seq
....................ugaAaacugaauuccauggac...	1	1	seq
....................ugagaacugaauuAcauggac...	1	1	seq
....................ugGgaacugaauuccauggac...	1	1	seq
....................ugagaacugaauuccaGggac...	1	1	seq
....................ugagaacugaauuccauAgac...	3	1	seq
....................uCagaacugaauuccauggac...	1	1	seq
....................ugagaacugaauuccaugUac...	1	1	seq
....................ugagaaUugaauuccauggac...	1	1	seq
....................ugagaacugaauucAauggac...	5	1	seq
....................ugagaacugaaCuccauggac...	1	1	seq
....................ugagaacugaGuuccauggac...	1	1	seq
....................ugagaacCgaauuccauggac...	3	1	seq
....................Agagaacugaauuccauggac...	1	1	seq
....................ugagaacugaauuccauggaU...	17	1	seq
....................ugagaacugaauuccauUgac...	3	1	seq
....................ugagaacugUauuccauggac...	1	1	seq
....................ugaUaacugaauuccauggac...	1	1	seq
....................ugagaUcugaauuccauggacu..	3	1	seq
....................ugagaacugGauuccauggacu..	19	1	seq
....................ugagaacugaGuuccauggacu..	23	1	seq
....................ugagaacugaauuGcauggacu..	4	1	seq
....................ugGgaacugaauuccauggacu..	2	1	seq
....................ugagaacugaauuUcauggacu..	25	1	seq
....................ugagaacugaauuccaAggacu..	6	1	seq
....................ugagaacugaUuuccauggacu..	1	1	seq
....................ugagaacugaauCccauggacu..	7	1	seq
....................ugagaaUugaauuccauggacu..	4	1	seq
....................uAagaacugaauuccauggacu..	13	1	seq
....................ugagaacGgaauuccauggacu..	2	1	seq
....................ugagaacugaauuccauggacG..	242	1	seq
....................ugagaacugaauuAcauggacu..	14	1	seq
....................ugagaacAgaauuccauggacu..	3	1	seq
....................ugagaacugaauuccauggacA..	2008	1	seq
....................ugagaacugaauGccauggacu..	1	1	seq
....................ugagaaAugaauuccauggacu..	15	1	seq
....................ugagaacCgaauuccauggacu..	16	1	seq
....................ugagaacugaauuccaCggacu..	3	1	seq
....................Ggagaacugaauuccauggacu..	43	1	seq
....................ugagaacugaauuccauggCcu..	2	1	seq
....................ugagaacugaCuuccauggacu..	2	1	seq
....................ugagaacugaauucGauggacu..	6	1	seq
....................ugagaacugaauucAauggacu..	12	1	seq
....................ugagaacuUaauuccauggacu..	12	1	seq
....................ugagaacugaauuccaugCacu..	2	1	seq
....................ugagaacugaauuccauggaGu..	3	1	seq
....................ugagaacugaauuccauggacC..	12	1	seq
....................ugagaacugaauuccauAgacu..	3	1	seq
....................ugagaacugaauAccauggacu..	4	1	seq
....................ugagCacugaauuccauggacu..	1	1	seq
....................ugagaacugaauuccauggacu..	68827	0	seq
....................ugagaacugaauuccCuggacu..	2	1	seq
....................uCagaacugaauuccauggacu..	2	1	seq
....................ugagaacugaauuccauggGcu..	13	1	seq
....................Cgagaacugaauuccauggacu..	25	1	seq
....................ugUgaacugaauuccauggacu..	2	1	seq
....................ugagaacuAaauuccauggacu..	6	1	seq
....................ugagaacugaauuccaugUacu..	14	1	seq
....................ugCgaacugaauuccauggacu..	1	1	seq
....................ugaAaacugaauuccauggacu..	3	1	seq
....................ugagaacugaauuccauCgacu..	6	1	seq
....................ugagaacugaaCuccauggacu..	6	1	seq
....................ugaUaacugaauuccauggacu..	11	1	seq
....................ugaCaacugaauuccauggacu..	4	1	seq
....................ugagaacugaaGuccauggacu..	3	1	seq
....................ugagaacugaauuccauggaAu..	25	1	seq
....................ugagaGcugaauuccauggacu..	118	1	seq
....................ugagaacugaauuccUuggacu..	6	1	seq
....................uUagaacugaauuccauggacu..	18	1	seq

Mature Star

```
accuuggcaguucccagcucugagaacugaauuccauggacugguuccaguuccaugcguucaguccaugguauucaguucucuagcuuggcugcaucgaacuucacaacua
....................ugagaacugaauuccaugAacu......................................................................	3	1	seq
....................ugagaaGugaauuccauggacu......................................................................	2	1	seq
....................ugagaacugaauuccauggUcu......................................................................	4	1	seq
....................ugagaacugaauuccauUgacu......................................................................	17	1	seq
....................ugagaacugaauuccauggaUu......................................................................	9	1	seq
....................ugagaacugaauucUauggacu......................................................................	34	1	seq
....................ugagaacugaauuccGuggacu......................................................................	9	1	seq
....................ugagaacugaauuccaGggacu......................................................................	4	1	seq
....................Agagaacugaauuccauggacu......................................................................	34	1	seq
....................ugagGacugaauuccauggacu......................................................................	12	1	seq
....................ugagaacuCaauuccauggacu......................................................................	6	1	seq
....................ugagaacugaaAuccauggacu......................................................................	8	1	seq
....................Ggagaacugaauuccauggacug.....................................................................	22	1	seq
....................ugagaGcugaauuccauggacug.....................................................................	43	1	seq
....................ugagaacugaauuccaAggacug.....................................................................	2	1	seq
....................ugagUacugaauuccauggacug.....................................................................	1	1	seq
....................ugagaacugaaAuccauggacug.....................................................................	3	1	seq
....................ugagaacugaauuccauggaAug.....................................................................	8	1	seq
....................ugagaaGugaauuccauggacug.....................................................................	1	1	seq
....................Agagaacugaauuccauggacug.....................................................................	17	1	seq
....................ugaUaacugaauuccauggacug.....................................................................	7	1	seq
....................ugagaacugaauuccauggacuC.....................................................................	5	1	seq
....................ugagaacugaUuuccauggacug.....................................................................	1	1	seq
....................ugaAaacugaauuccauggacug.....................................................................	3	1	seq
....................ugagaacugaauuGcauggacug.....................................................................	4	1	seq
....................ugagaacugaauuAcauggacug.....................................................................	7	1	seq
....................ugagaacugaauuccaugUacug.....................................................................	5	1	seq
....................ugagaacugaauuccauggUcug.....................................................................	1	1	seq
....................ugagaacuAaauuccauggacug.....................................................................	2	1	seq
....................ugGgaacugaauuccauggacug.....................................................................	8	1	seq
....................ugagaacugaauCccauggacug.....................................................................	6	1	seq
....................ugagaacugaauucUauggacug.....................................................................	5	1	seq
....................ugagaacugaauuccaCggacug.....................................................................	3	1	seq
....................uCagaacugaauuccauggacug.....................................................................	3	1	seq
....................ugagaacugaauucGauggacug.....................................................................	4	1	seq
....................ugagaacAgaauuccauggacug.....................................................................	1	1	seq
....................ugagaacugaauuccauAgacug.....................................................................	7	1	seq
....................uUagaacugaauuccauggacug.....................................................................	6	1	seq
....................ugagaacuUaauuccauggacug.....................................................................	4	1	seq
....................ugagaacugaauuccauggaUug.....................................................................	13	1	seq
....................Cgagaacugaauuccauggacug.....................................................................	11	1	seq
....................ugagaacugaauuccauggacGg.....................................................................	171	1	seq
....................ugagaacugaauuccauggGcug.....................................................................	9	1	seq
....................ugagaacugaauAccauggacug.....................................................................	2	1	seq
....................ugagaacugaauuccauggacuU.....................................................................	844	1	seq
....................ugagaacugaauuccauggacAg.....................................................................	6	1	seq
....................ugagaacCgaauuccauggacug.....................................................................	9	1	seq
....................ugagaacuCaauuccauggacug.....................................................................	2	1	seq
....................ugagaacugaGuuccauggacug.....................................................................	7	1	seq
....................ugagaacugaauuccauggacug.....................................................................	35955	0	seq
....................ugaCaacugaauuccauggacug.....................................................................	4	1	seq
....................ugagaacugaauuccauggacCg.....................................................................	5	1	seq
....................ugagaaAugaauuccauggacug.....................................................................	9	1	seq
....................ugagaacugaaCuccauggacug.....................................................................	6	1	seq
....................ugagaacugaauucAauggacug.....................................................................	5	1	seq
....................ugagaacugaauuccaugCacug.....................................................................	3	1	seq
....................ugagaacugaauuccauggacuA.....................................................................	180	1	seq
....................uAagaacugaauuccauggacug.....................................................................	4	1	seq
....................ugagaacugaauuccauUgacug.....................................................................	14	1	seq
....................ugUgaacugaauuccauggacug.....................................................................	2	1	seq
....................ugagaacugaauuccUuggacug.....................................................................	5	1	seq
....................ugagGacugaauuccauggacug.....................................................................	1	1	seq
....................ugagaacugGauuccauggacug.....................................................................	4	1	seq
....................ugagaaUugaauuccauggacug.....................................................................	3	1	seq
....................ugagaacugaauuccauCgacug.....................................................................	3	1	seq
....................ugagaacugaauuccauggacugU....................................................................	61454	1	seq
....................ugagaacugaauuccauggacuAg....................................................................	5	1	seq
....................ugagaaAugaauuccauggacugg....................................................................	1	1	seq
....................ugagaacugaauuccauggacugA....................................................................	6504	1	seq
....................ugagaacugaauuccauggacuUg....................................................................	1	1	seq
```

Mature Star

accuuggcaguucccagcucugagaacugaauuccauggacugguuccaguuccaugcguucaguccaugguauucaguucucuagcuuggcugcaucgaacuucacaacua			
....................ugaAaacugaauuccauggacugg..	1	1	seq
....................ugagaacugaauuccauggacugg..	279	0	seq
....................ugagaacugaauuccauggacugC..	230	1	seq
....................ugagaacugaauuccauggaAugg..	1	1	seq
....................ugagaacugaauuccauggacugUu...	3471	1	seq
....................ugagaacugaauuccauggacugCu...	26	1	seq
....................ugagaacugaauuccauggacuggA...	8	1	seq
....................ugagaacugaauuccauggacugAu...	345	1	seq
....................ugagaacugaauuccauggacuAgu...	1	1	seq
....................ugagaacugaauuccauggacuggu...	8	0	seq
....................ugagaacugaauuccauggacugguu..	1	0	seq
....................ugagaacugaauuccauggacugAuu..	17	1	seq
....................ugagaacugaauuccauggacugUuu..	343	1	seq
....................ugagaacugaauuccauggacuggAu..	1	1	seq
....................ugagaacugaauuccauggacugguuc...	3	0	seq
....................ugagaacugaauuccauggacugguucc..	3	0	seq
.....................gagaacugaauuccauggac...	10	0	seq
.....................Uagaacugaauuccauggacu..	1	1	seq
.....................gagaacugaauuccauggacu..	60	0	seq
.....................gagaacugaauuccauggacA..	2	1	seq
.....................gagaacugaauuccauggacug...	74	0	seq
.....................gagaacugaauuccauggacuC...	1	1	seq
.....................gagaacugaauuccauggacugA..	32	1	seq
.....................gagaacugaauuccauggacugU..	340	1	seq
.....................gagaacugaauuccauggacugC..	2	1	seq
.....................gagaacugaauuccauggacugUu...	17	1	seq
.....................gagaacugaauuccauggacugAu...	1	1	seq
......................agaacugaauuccauggac...	9	0	seq
......................agaacugaauuccauggacA..	1	1	seq
......................agaacugaauuccauggacu..	31	0	seq
......................agaacugaauuccauggacuU...	1	1	seq
......................agaacugaauuccauggacGg...	1	1	seq
......................agaacugaauuccGuggacug...	1	1	seq
......................agaacugaauuccauggacug...	234	0	seq
......................agaacugaauuccauggacugg..	79	0	seq
......................agaacugaauuccauggacugC..	4	1	seq
......................agaacugaauuccauggacugU..	244	1	seq
......................agaacugaauuccauggacugA..	31	1	seq
......................agaacugaauuccauggaAugg..	1	1	seq
......................agaacugaauuccauggacuggA...	20	1	seq
......................agaacugaauuccauggacugAu...	1	1	seq
......................agaacugaauuccauggacuggu...	15	0	seq
......................agaacugaauuccauggacugUu...	13	1	seq
......................agaacugaauuccauggacugUuu..	2	1	seq
......................agaacugaauuccauggacugguu..	2	0	seq
......................agaacugaauuccauggacugguuA...	1	1	seq
.......................gaacugaauuccauggacu..	1	0	seq
.......................gaacugaauuccauggacug...	6	0	seq
.......................gaacugaauuccauggacugg..	10	0	seq
.......................gaacugaauuccauggacugU..	43	1	seq
.......................gaacugaauuccauggacugA..	3	1	seq
.......................gaacugaauuccauggacugUu...	6	1	seq
.......................gaacugaauuccauggacuggG...	1	1	seq
.......................gaacugaauuccauggacuggu...	1	0	seq
.......................gaacugaauuccauggacuggA...	5	1	seq
.......................gaacugaauuccauggacugguA..	1	1	seq
........................aacugaauuccauggacu..	16	0	seq
........................aacugaauuccauggacA..	1	1	seq
........................aacCgaauuccauggacug...	1	1	seq
........................aacugaauuAcauggacug...	1	1	seq
........................aacugaauuccauggacug...	369	0	seq
........................aacugaauuccauggacGg...	1	1	seq
........................Gacugaauuccauggacug...	1	1	seq
........................aacugaauuccauggacugg..	503	0	seq
........................aacugaauuccauggacugU..	922	1	seq
........................aacugaauuccauggacugC..	10	1	seq
........................aacugaauucUauggacugg..	1	1	seq
........................aacugaauuccauggacugA..	61	1	seq
........................aacugaauuccauggacuggA...	107	1	seq
........................aacugaauuccauggacuggu...	27	0	seq

Mature **Star**

```
accuuggcaguucccagcucugagaacugaauuccauggacugguuccaguuccaugcguucaguccaugguauucaguucucuagcuuggcugcaucgaacuucacaacua
........................aacugaauuccauggacugUu...................................................................    25    1    seq
........................aacugaauuccauggacugCu...................................................................    1     1    seq
........................aacugaauuccauggacuggG...................................................................    1     1    seq
........................aacugaauuccauggacugguG..................................................................    1     1    seq
........................aacugaauuccauggacugAuu..................................................................    1     1    seq
........................aacugaauuccauggacugUuu..................................................................    4     1    seq
........................aacugaauuccauggacugguA..................................................................    10    1    seq
........................aacugaauuccauggacuggAu..................................................................    2     1    seq
........................aacugaauuccauggacugguu..................................................................    65    0    seq
........................Gacugaauuccauggacugguuc.................................................................    1     1    seq
........................aacugaauuccauggacugguuc.................................................................    63    0    seq
........................aacugaauuccauggacugguuU.................................................................    9     1    seq
........................aacugaaAuccauggacugguuc.................................................................    1     1    seq
........................aacugaauuccauggacugguuA.................................................................    2     1    seq
........................aacugaauuccaAggacugguucc................................................................    1     1    seq
........................aacugaauuccauggacugguucU................................................................    11    1    seq
.........................acugaauuccauggacug.....................................................................    11    0    seq
.........................acugaauuccauggGcug.....................................................................    1     1    seq
.........................acugaauuccauggacugU....................................................................    17    1    seq
.........................acugaauuccauggacugA....................................................................    1     1    seq
.........................acugaauuccauggacugguuc.................................................................    4     0    seq
..........................cugaauuccauggacugA....................................................................    1     1    seq
..........................cugaauuccauggacugU....................................................................    2     1    seq
...........................ugaauuccauggacugguuc.................................................................    1     0    seq
...........................ugaauuccauggacugguucU................................................................    1     1    seq
.........................................................cguucaguccaugguauucag..................................    1     0    seq
............................................................ucaguccaugguauucaguuc...............................    3     0    seq
............................................................ucaguccaugguauucaguucu..............................    5     0    seq
............................................................Gcaguccaugguauucaguucu..............................    1     1    seq
............................................................ucaguccaugguauucaguucuc.............................    12    0    seq
............................................................ucaguccaugguauucaguuGuc.............................    1     1    seq
............................................................ucaguccaugguauucaguucucu............................    2     0    seq
..............................................................aguccaugguauucaguucuc.............................    22    0    seq
..............................................................aguccaugguauucaAuucuc.............................    1     1    seq
..............................................................aguccaugguauucaguucucu............................    34    0    seq
..............................................................aguccaugguauucaguucucua...........................    1     0    seq
..............................................................aguccaugguauucaguucucuaU..........................    1     1    seq
...............................................................guccaugguauucaguucu..............................    2     0    seq
...............................................................guccaugguauucaguucuc.............................    24    0    seq
...............................................................guccaugguauucaguucucu............................    123   0    seq
...............................................................Auccaugguauucaguucucu............................    1     1    seq
...............................................................guccaugguauucaguucucua...........................    40    0    seq
...............................................................guccaugguauucaguucucuaA..........................    1     1    seq
...............................................................guccaugguauucaguucucuaU..........................    11    1    seq
................................................................uccaugguauucaguucu..............................    2     0    seq
................................................................uccaugguauucaguucuc.............................    7     0    seq
................................................................uccaugUuauucaguucuc.............................    1     1    seq
................................................................Accaugguauucaguucuc.............................    1     1    seq
................................................................uccaugguauucaguucucu............................    79    0    seq
................................................................uccauggGauucaguucucu............................    1     1    seq
................................................................uccaugguauucaguucucua...........................    15    0    seq
................................................................uccaugguauucaguucucuU...........................    1     1    seq
................................................................uccaugguauucaguucucuaA..........................    12    1    seq
................................................................uccaugguauucaguucucuaC..........................    5     1    seq
................................................................uccaugguauucaguucucuaU..........................    44    1    seq
................................................................uccaugguauucaguucucuag..........................    1     0    seq
................................................................uccaugguauucaguucucuagA.........................    1     1    seq
...................................................................augguauucaguucucua...........................    1     0    seq
```

Provisional ID	:	mun-novel31
Score total	:	1.6
Score for star read(s)	:	-1.3
Score for read counts	:	0
Score for mfe	:	1.9
Score for randfold	:	1.6
Score for cons. seed	:	-0.6
Total read count	:	33
Mature read count	:	33
Loop read count	:	0
Star read count	:	0

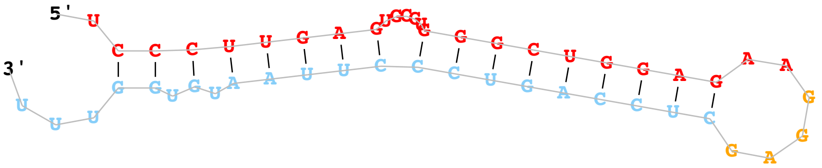

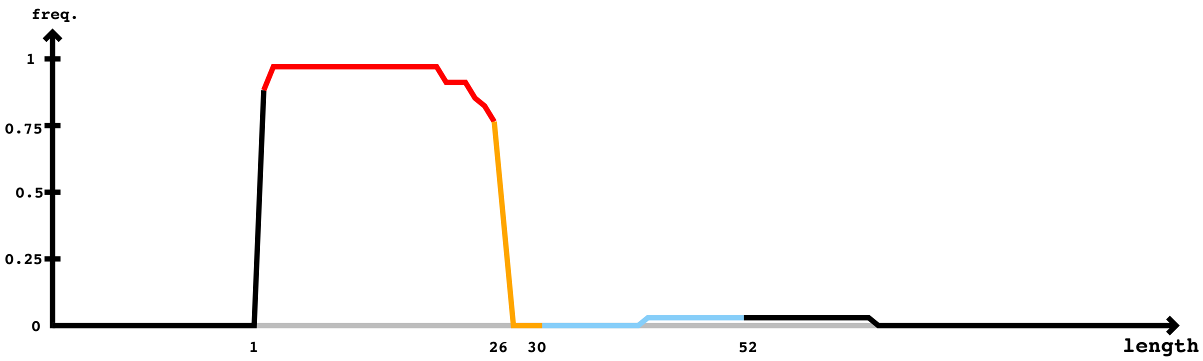

Mature **Star**

```
5'- aguaguggagauccaugggucccuugagugcgugggcuggagaaggagcuccagucccuuaaugugguuuccccaaauccucauuacuuggugucccccugugaggggagucau   -3'    exp
    (((((((..(((...((((..((((((((.....(((((((((......)))))))))))))).).))....)))).))).)))))))..(((.(((((.....)))).).)))   reads   mm   sample
    ....................ucccuugagugcgugggcu............................................................................   1       0    seq
    ....................ucccuugagGgcgugggcu............................................................................   1       1    seq
    ....................ucccuugagugcgugggcugga.........................................................................   2       0    seq
    ....................ucccuugagugcgugggcuggag........................................................................   1       0    seq
    ....................ucccuugagugcgugggcuggaga.......................................................................   2       0    seq
    ....................ucccuugagugcgugggcuggagGa......................................................................   1       1    seq
    ....................ucccuugagugcAugggcuggagaa......................................................................   2       1    seq
    ....................ucccuugagugcgugggcuggagaa......................................................................   19      0    seq
    ....................ucccuugagugcgugggcuggagaaA.....................................................................   1       1    seq
    .....................cccuugagugcAugggcuggagaa......................................................................   1       1    seq
    .....................cccuugagugcgugggcuggagaa......................................................................   2       0    seq
    ............................................................uaaugugguuuccccaaauccuca...............................   1       0    seq
```

Provisional ID	:	mun-novel11
Score total	:	758.1
Score for star read(s)	:	3.9
Score for read counts	:	755.2
Score for mfe	:	1.8
Score for randfold	:	-2.2
Score for cons. seed	:	-0.6
Total read count	:	1493
Mature read count	:	1489
Loop read count	:	3
Star read count	:	1

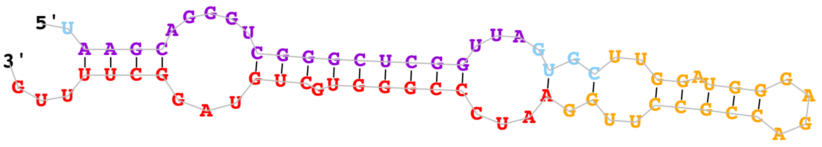

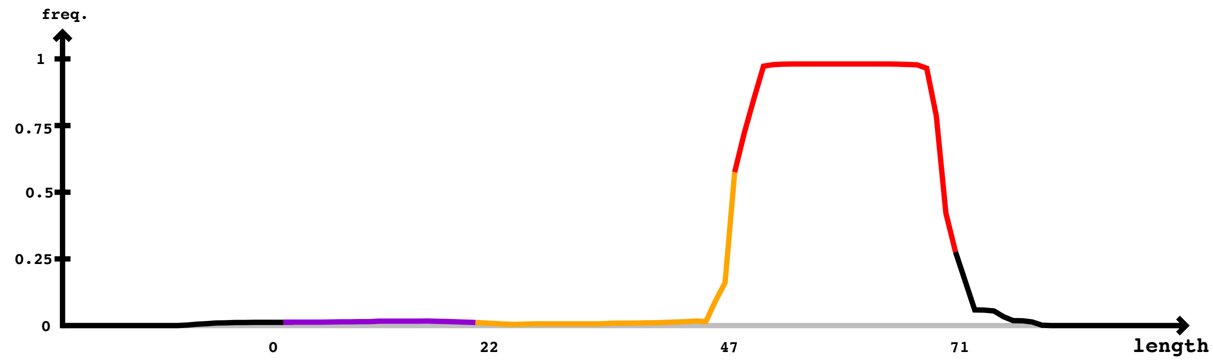

Star Mature

```
5'- aucucgucugcucucggaagcuaagcagggucgggcucgguuagugcuuggaugggagaccgccuuggaaucccgggugcuguaggcuuuugccagccgauaggggacccucuga  -3'   obs
    aucucgucugcucucggaagcuaagcagggucgggcucgguuagugcuuggaugggagaccgccuuggaaucccgggugcuguaggcuuuugccagccgauaggggacccucuga         exp
    .....((((.((.((((..((.((((.....(((((((((....(.(..((.(((....)))))..).)...)))))).)))...)))).))...)))).)).)))).......    reads   mm   sample
    ...........ucucggaagcuaagcagggucgggcucU............................................................................    1       1    seq
    ...........ucucggaagcuaagcagggucgggGucgg...........................................................................    1       1    seq
    ...........ucucggaagcuaagcagggucgggcucgguuagu......................................................................    1       0    seq
    ............cucggaagcuaagAagggucgggcu..............................................................................    1       1    seq
    ............Uucggaagcuaagcagggucgggcuc.............................................................................    1       1    seq
    ............cucggaagcuaagcagggucgggcucgguu.........................................................................    2       0    seq
    ............cucggaagcuaagcagggucgggcucggGu.........................................................................    1       1    seq
    .............ucggaagcuaagcagggucgggcucggu..........................................................................    1       0    seq
    .............ucggaagcuaagcagggucgggcucgguu.........................................................................    1       0    seq
    .............ucggaagcuaagcagggucgggcucgguuU........................................................................    1       1    seq
    ..............cggaagcuaagcagggucgggcucggu..........................................................................    1       0    seq
    ..............cggaagcuaagcagggucgggcucggGu.........................................................................    1       1    seq
    ..............cggaagcuaagcagggucgggcucgguu.........................................................................    1       0    seq
    ..............cggaagcuaagcagggucgggcucgguuagu......................................................................    1       0    seq
    ...............ggaagcuaagcagggAcgggcucgg...........................................................................    1       1    seq
    ................gaagcuaagcagggAcgggcu..............................................................................    1       1    seq
    ................gaagcuaagcagggucgggcucU............................................................................    1       1    seq
    ..................agcuaagcagggucgggcucggGu.........................................................................    1       1    seq
    ......................aagcagggucgggcucgguuU........................................................................    1       1    seq
    ..........................agggucgggcucgguuaguUcuuggaugggagaccgcc...................................................    1       1    seq
    ...........................gggucgggUucgguuagugcuuggaugggagaccgccu..................................................    1       1    seq
    .............................gucgggcucgguuagugcuuggaugggagaccgccu..................................................    1       0    seq
    ...............................cgggcucgguuagugcuuggaugggagaccgccu..................................................    2       0    seq
    ...............................cgggcucgguuagugcuuggaugggagaccgccuuggaau............................................    1       0    seq
    ....................................ucgguuagugcuuggaugggagaccgccu..................................................    1       0    seq
    ..............................................cuuggaugggagaccgccG..................................................    1       1    seq
    ...............................................uuggaugggagaccgccuuggaauc...........................................    2       0    seq
    .......................................................gagaccgccuAggaaucccgggugcuguaggcu...........................    1       1    seq
    .......................................................gagaccgccuuggaaucccgggugcuguaggcuuu.........................    1       0    seq
    .......................................................gagaccgccuuggaaucccgggugcuguaggcuuuuU.......................    1       1    seq
    ........................................................agaccgccuuggaaucccgggugcuguaggcuuuu........................    1       0    seq
    ..........................................................accgccuuggaaucccgggugcuguaggcu...........................    1       0    seq
    ...........................................................ccgccuuggaaucccgggugcuguaggcuuuu........................    1       0    seq
```

```
                          Star                                                    Mature
aucucgucugcucucggaagcuaagcagggucgggcucgguuagugcuuggaugggagaccgccuuggaaucccgggugcuguaggcuuuugccagccgauaggggacccucuga
............................................................cgccuuggaaucccgggugcuguaggcuuuu........................   1   0   seq
.............................................................gccuuggaaucccgggugcuguaggcuuu.........................   1   0   seq
.............................................................gccuuggaaucccgggugcuguaggcuuuu........................   1   0   seq
..............................................................ccuuggaaucccgggugcuguaggcuu..........................   1   0   seq
..............................................................ccuuggaaucccgggugcuguaggcuuu.........................   1   0   seq
...............................................................cuuggaaucccgggugcuguaggcuuuu........................   2   0   seq
................................................................uAggaaucccgggugcuguaggcuu..........................   1   1   seq
................................................................uuggaaucccgggugcuguaggcuuu.........................   1   0   seq
................................................................uuggaaucccgggugcuguaggcuuA.........................   3   1   seq
.................................................................Cggaaucccgggugcuguaggcuu..........................   1   1   seq
.................................................................uggaaucccgggugcuguaggcuuu.........................   1   0   seq
..................................................................ggaaucccgggugcuguaC..............................   1   1   seq
..................................................................ggaaucccgggugcuguaggcuC..........................   7   1   seq
..................................................................ggaaucccgggugcCguaggcuu..........................   1   1   seq
..................................................................Ugaaucccgggugcuguaggcuu..........................   1   1   seq
..................................................................ggaaucAcgggugcuguaggcuu..........................   1   1   seq
..................................................................ggaaucccgggugcuguaggcuG..........................   1   1   seq
..................................................................ggaaucccgggugcuguaUgcuu..........................   1   1   seq
..................................................................gAaaucccgggugcuguaggcuu..........................   1   1   seq
..................................................................ggaauAccgggugcuguaggcuu..........................   17  1   seq
..................................................................ggaaucccgggugcuguaggcuA..........................   19  1   seq
..................................................................Agaaucccgggugcuguaggcuuu.........................   1   1   seq
..................................................................ggaaucccgggugcuguaggcuCu.........................   3   1   seq
..................................................................ggaaucccCggugcuguaggcuuu.........................   1   1   seq
..................................................................ggaaucccggguUcuguaggcuuu.........................   1   1   seq
..................................................................ggaaucccgggugcuguaggcuAu.........................   1   1   seq
..................................................................ggaaucccgggugcuguaggUuuu.........................   1   1   seq
..................................................................ggaaucccgggugcuguaggcuuA.........................   45  1   seq
..................................................................ggaaucccgggugcuguaggcuuC.........................   1   1   seq
..................................................................ggaauAccgggugcuguaggcuuu.........................   3   1   seq
..................................................................ggaaucccgggugcuguaUgcuuu.........................   1   1   seq
..................................................................ggaaucccgggCgcuguaggcuuu.........................   1   1   seq
..................................................................gAaaucccgggugcuguaggcuuu.........................   1   1   seq
..................................................................ggaaucccgggugcuguaggcuuG.........................   1   1   seq
..................................................................ggaaucccgggugcuguaggcuAuu........................   3   1   seq
..................................................................ggaaucccgggugcuguaggcuuCu........................   1   1   seq
..................................................................ggaaucccgggugcuguaggcuuAu........................   1   1   seq
..................................................................ggaaucccgggugcuguaggcuuuuC.......................   1   1   seq
..................................................................ggaaucccgggugcuguaggcuuuuU.......................   4   1   seq
..................................................................ggaaucccgggugcuguaggcuuuuUcc.....................   1   1   seq
...................................................................gaaucccgggugcuguaggcA...........................   1   1   seq
...................................................................gaaucccgggugcugGaggcuu..........................   1   1   seq
...................................................................gaauAccgggugcuguaggcuu..........................   2   1   seq
...................................................................gaaucccgggugcuguaggcuC..........................   11  1   seq
...................................................................Aaaucccgggugcuguaggcuu..........................   1   1   seq
...................................................................gaaucccgggugcuguaggcuG..........................   2   1   seq
...................................................................gaaucccgggCgcuguaggcuu..........................   1   1   seq
...................................................................gaaucccgggugcuguaggcuA..........................   21  1   seq
...................................................................gaaucccgggugcuguagUcuu..........................   1   1   seq
...................................................................gaaucccgggugcuguaUgcuu..........................   1   1   seq
...................................................................gaaucccgggugcuguaggcuuC.........................   1   1   seq
...................................................................gaauAccgggugcuguaggcuuu.........................   7   1   seq
...................................................................gaaucccgggugcuguaggcuuA.........................   50  1   seq
...................................................................gaaucccgggugcuguaggcuAu.........................   1   1   seq
...................................................................gaaucccgggugcuguaggcuuAu........................   1   1   seq
...................................................................gaaucccgggugcuguaggcuAuu........................   1   1   seq
...................................................................gaaucccgggugcuguaggcuuuC........................   1   1   seq
...................................................................gaaucccgggugcuguaggcuuuuA.......................   1   1   seq
...................................................................gaaucccgggugcuguaggcuuuuU.......................   2   1   seq
....................................................................aaucccgggugcuguagU.............................   2   1   seq
....................................................................Caucccgggugcuguaggc............................   1   1   seq
....................................................................aaucccgggugcuguagAcu...........................   1   1   seq
....................................................................aaucccgggugcuguaggcG...........................   1   1   seq
....................................................................aaucccgggugcuguaggcA...........................   8   1   seq
....................................................................aaucccgggAgcuguaggcuu..........................   1   1   seq
....................................................................aaucccggUugcuguaggcuu..........................   1   1   seq
....................................................................aaucccgggCgcuguaggcuu..........................   1   1   seq
....................................................................aaucccgggugcuguaggcuC..........................   4   1   seq
....................................................................aauUccgggugcuguaggcuu..........................   1   1   seq
....................................................................aaucccgggugcuguaggcuA..........................   55  1   seq
```

Star Mature

```
aucucgucugcucucggaagcuaagcagggucgggcucgguuagugcuuggaugggagaccgccuuggaaucccgggugcuguaggcuuuugccagccgauaggggacccucuga
....................................................................aaucccgggGgcuguaggcuu..........................  1    1  seq
....................................................................aaucccgggugcuguaggcAu..........................  1    1  seq
....................................................................aaucccgggugcugCaggcuu..........................  1    1  seq
....................................................................aauAccgggugcuguaggcuuu.........................  2    1  seq
....................................................................aaucccgggugcuguaggcuuA.........................  147  1  seq
....................................................................aaucccgggugcuguaggcAuu.........................  2    1  seq
....................................................................aaucccgggGgcuguaggcuuu.........................  1    1  seq
....................................................................aaucccgggugcuguaggcuAu.........................  38   1  seq
....................................................................aaucccgggugcuguaggcuuG.........................  2    1  seq
....................................................................aaucccgggAgcuguaggcuuu.........................  2    1  seq
....................................................................Gaucccgggugcuguaggcuuu.........................  1    1  seq
....................................................................aaucccggguUcuguaggcuuu.........................  1    1  seq
....................................................................aaucccgggugcuguaggcuuC.........................  11   1  seq
....................................................................aaucccgggugcuguaggcuCu.........................  4    1  seq
....................................................................aaucccgggugcugCaggcuuu.........................  1    1  seq
....................................................................aaucccgggugcuguaggcuuuG........................  1    1  seq
....................................................................aaucccgggugcuguaggcuuAu........................  72   1  seq
....................................................................aaucccgggugUuguaggcuuuu........................  1    1  seq
....................................................................aaucccgggugcuguaggcuuCu........................  17   1  seq
....................................................................aaucccgggugcCguaggcuuuu........................  1    1  seq
....................................................................aaucccgggugcuguaggcuuuC........................  7    1  seq
....................................................................aauccAgggugcuguaggcuuuu........................  1    1  seq
....................................................................aaucccgCgugcuguaggcuuuu........................  1    1  seq
....................................................................aauAccgggugcuguaggcuuuu........................  1    1  seq
....................................................................aaucccgggugcuguaggcuCuu........................  1    1  seq
....................................................................aaucccgggAgcuguaggcuuuu........................  3    1  seq
....................................................................aaucccgggGgcuguaggcuuuu........................  1    1  seq
....................................................................aaucccgggugcuguaggcuAuu........................  56   1  seq
....................................................................aaucccgggugcuguaggcuuuAg.......................  1    1  seq
....................................................................aaucccgggugcuguaggcuuuuU.......................  179  1  seq
....................................................................aaucccgggugcuguaggcuuuuA.......................  10   1  seq
....................................................................aaucccgggugcuguaggcuuuuC.......................  3    1  seq
....................................................................aaucccgggugcuguaggcuuuuUc......................  1    1  seq
....................................................................aaucccgggugcuguaggcuuuugccaU...................  1    1  seq
....................................................................aaucccgggugcuguaggcuuuugccagccgU...............  1    1  seq
.....................................................................aucccgggugcuCuaggc............................  1    1  seq
.....................................................................aucccgggugcuguaggcG...........................  1    1  seq
.....................................................................aucccgggugcuguaggcA...........................  2    1  seq
.....................................................................aucccgggugUuguaggcuu..........................  1    1  seq
.....................................................................aCcccgggugcuguaggcuu..........................  1    1  seq
.....................................................................aucccgggugcuguaggcuA..........................  33   1  seq
.....................................................................auAccgggugcuguaggcuu..........................  2    1  seq
.....................................................................aucccgggugcugGaggcuu..........................  1    1  seq
.....................................................................aucAcgggugcuguaggcuu..........................  2    1  seq
.....................................................................aucccgggugcuguaggcuC..........................  1    1  seq
.....................................................................aucccgggugcuguaggcuG..........................  1    1  seq
.....................................................................Uucccgggugcuguaggcuu..........................  2    1  seq
.....................................................................auAccgggugcuguaggcuuu.........................  3    1  seq
.....................................................................aucccgggugcuguaggcuuA.........................  70   1  seq
.....................................................................aucccgggCgcuguaggcuuu.........................  1    1  seq
.....................................................................aucccgggugcuguaggcuuC.........................  1    1  seq
.....................................................................aucccgggugcuguaggcuAu.........................  5    1  seq
.....................................................................Cucccgggugcuguaggcuuuu........................  1    1  seq
.....................................................................aucccgggugcuguaggcuuCu........................  4    1  seq
.....................................................................auAccgggugcuguaggcuuuu........................  1    1  seq
.....................................................................aucccgggugcuguaggcuAuu........................  10   1  seq
.....................................................................aucccgggugcuguaggcuuuC........................  2    1  seq
.....................................................................aucccgggugcuguaggcuuAu........................  16   1  seq
.....................................................................aucccgggugcuguaggcuuuuU.......................  56   1  seq
.....................................................................aucccgggugcuguaggcuuuuA.......................  10   1  seq
.....................................................................aucccgggugcuguaggcuuuuC.......................  1    1  seq
.....................................................................aucccgggugcuguaggcuuuugccaU...................  1    1  seq
......................................................................ucccgggugcuguaggcA...........................  3    1  seq
......................................................................ucccgggugcuguaggcG...........................  1    1  seq
......................................................................ucccgggugcuguaggcuA..........................  27   1  seq
......................................................................ucccgggugcuguaggcuG..........................  1    1  seq
......................................................................uAccgggugcuguaggcuu..........................  1    1  seq
......................................................................ucccgggugcuguaggcuuA.........................  48   1  seq
......................................................................ucccgggugcuUuaggcuuu.........................  1    1  seq
......................................................................ucccggguUcuguaggcuuu.........................  1    1  seq
```

Star **Mature**

aucucgucugcucucggaagcuaagcagggucgggcucgguuagugcuuggaugggagaccgccuuggaaucccgggugcuguaggcuuuugccagccgauaggggacccucuga			
....uAccgggugcuguaggcuuu.........................	2	1	seq
....ucccgggugcuguaggcuCu.........................	1	1	seq
....ucccgggugcuguaggcuuuC........................	2	1	seq
....ucccgggCgcuguaggcuuuu........................	1	1	seq
....ucccgggugcuguaggcuuAu........................	2	1	seq
....ucccgggugcuguaggcuAuu........................	2	1	seq
....ucccgggugcuguaggcuuuuC.......................	1	1	seq
....ucccgggugcuguaggcuuuuU.......................	17	1	seq
....ucccgggugcuguaggcuuuuA.......................	5	1	seq
....ucccgggugcuguaggcuuuuUc......................	1	1	seq
....ucccgggGgcuguaggcuuuugcca....................	1	1	seq
....ucccgggugcuguaggcuuuugccU....................	1	1	seq
....ucccgggugcuguaggcuuuCgcca....................	2	1	seq
....ucccgggugcuguaggAuuuugcca....................	1	1	seq
....ucccgggugcuguaggcuuuugccaA...................	4	1	seq
....Acccgggugcuguaggcuuuugccag...................	1	1	seq
....ucccgggugcuguaggcuuuugccaU...................	24	1	seq
....ucccggUugcuguaggcuuuugccag...................	1	1	seq
....ucccgggGgcuguaggcuuuugccagc..................	3	1	seq
....ucccgggugcuguaggcuuuugccagA..................	1	1	seq
....ucccgggugcuguaggcuuuugcAagc..................	1	1	seq
....ucccgggugcuguaggcuuuugccagU..................	6	1	seq
....ucccgggugcuguaggcuuuuUccagc..................	1	1	seq
....ucccgggugcuUuaggcuuuugccagc..................	1	1	seq
....ucccgggugcuguaggcuuuCgccagc..................	8	1	seq
....ucccAggugcuguaggcuuuugccagc..................	1	1	seq
....ucccgggugcuguaggcuuuCgccagcc.................	1	1	seq
....ucccgggugcuguaggcuuuugccagccU................	3	1	seq
....ucccgggugcuguaggcuuuugccagccA................	1	1	seq
....ucccgggugcuguaggcuuuugcAagccg................	1	1	seq
....ucccgggugcuguaggcuuuCgccagccg................	2	1	seq
....ucccgggugcuguaggcuuuugccagccgU...............	16	1	seq
....ucccgggugcuguaggcuuuugccagccgC...............	1	1	seq
....ucccgggugcuguaggcuuuugccagccgaA..............	2	1	seq
....ccAgggugcuguaggcuu..........................	1	1	seq
....cccgggGgcuguaggcuu..........................	2	1	seq
....cccgggugcuguaggcuC..........................	2	1	seq
....Accgggugcuguaggcuu..........................	5	1	seq
....cccgggugcuguaggcuG..........................	1	1	seq
....cccgggugcuguaggcuA..........................	38	1	seq
....cccgggugcuguaggcuAu.........................	1	1	seq
....Accgggugcuguaggcuuu.........................	1	1	seq
....cccgggugcuguaggcuuA.........................	89	1	seq
....cccgggugcuguaggcuuAu........................	3	1	seq
....cccgggugAuguaggcuuuu........................	1	1	seq
....cccgggugcuguaggcuuGu........................	1	1	seq
....cccgggugcuguaggcuAuu........................	2	1	seq
....cccgggugcuguaggcuuuC........................	1	1	seq
....cccgggugcuguaggcuuuuU.......................	27	1	seq
....cccgggugcuguaggcuuuuA.......................	14	1	seq
....cccgggugcuguaggcuuuugccaU...................	2	1	seq
....cccgggugcuguaggcuuuugccagU..................	1	1	seq
....cccgggugcuguaggcuuuuAccagccga...............	1	1	seq
....ccgggCgcuguaggcuuu.........................	1	1	seq
....ccgggugcuguaggcuuA.........................	7	1	seq
....ccgggugcuguaggcuuuuU.......................	3	1	seq
....cgggugcuguaggcuuCu........................	1	1	seq
....cgggugcuguaggcuuuuU.......................	2	1	seq
....gggugcuguaggcuuuuU.......................	1	1	seq

Provisional ID	:	mun-miR-190
Score total	:	299.6
Score for star read(s)	:	3.9
Score for read counts	:	289.2
Score for mfe	:	1.9
Score for randfold	:	1.6
Score for cons. seed	:	3
Total read count	:	579
Mature read count	:	533
Loop read count	:	0
Star read count	:	46

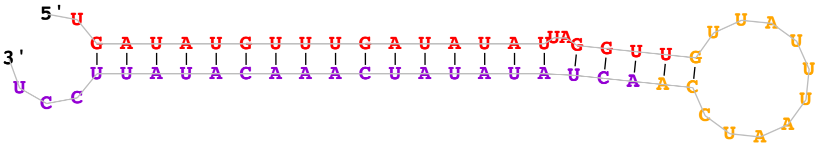

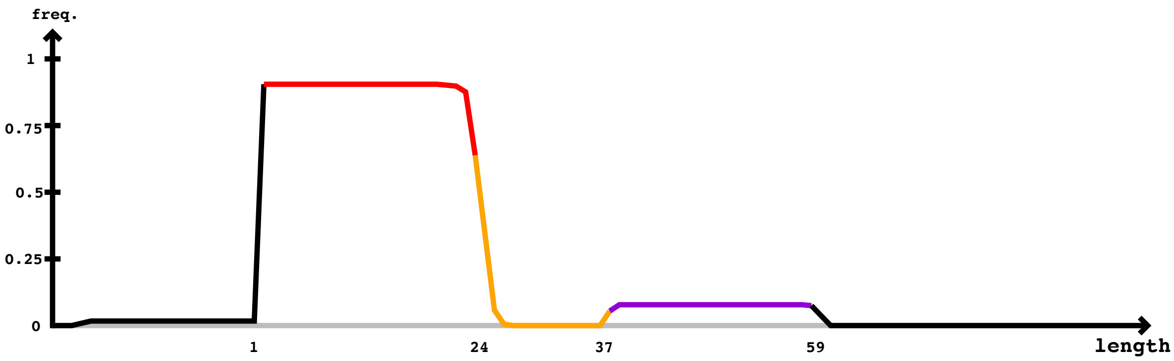

Mature Star

```
5'- uuuuucugcaggacucugugugauauguuugauauauuagguuguuauuuaauccaacuauauaucaaacauauuccuacaguguccugcccugucuccgggguuuuaaua   -3'   obs
    uuuuucugcaggacucugugugauauguuugauauauuagguuguuauuuaauccaacuauauaucaaacauauuccuacaguguccugcccugucuccgggguuuuaaua         exp
    ........((((((.(((((..(((((((((((((((..(((((..........))))))))))))))))))))..).)))).))))))(((((....)))))........   reads   mm   sample
    .uuuucugcaggacucugug...........................................................................................   5       0    seq
    ..uuucugcaggacucugug...........................................................................................   5       0    seq
    ....................ugauauguuugauauauua........................................................................   2       0    seq
    ....................ugauauguuugauauauuag.......................................................................   2       0    seq
    ....................ugauauguuugauauauuagg......................................................................   12      0    seq
    ....................ugauauguuugauauauuagU......................................................................   1       1    seq
    ....................ugauauguuugauauauuaggu.....................................................................   135     0    seq
    ....................ugauauguuugauauauuaggA.....................................................................   5       1    seq
    ....................Agauauguuugauauauuagguu....................................................................   1       1    seq
    ....................ugauauguuugauauauuaggGu....................................................................   1       1    seq
    ....................ugauauguuugauauauuagguA....................................................................   6       1    seq
    ....................ugauauguuugauauauuagguu....................................................................   239     0    seq
    ....................ugauauguuugauauauuagguG....................................................................   2       1    seq
    ....................ugauauguuugauauauuaggAu....................................................................   4       1    seq
    ....................ugauauguuugauauauuagguuU...................................................................   13      1    seq
    ....................ugauauguuugauauauuagguuA...................................................................   3       1    seq
    ....................ugauauguuugauauauuagguug...................................................................   73      0    seq
    ....................ugauauguuugauauauuagguuAu..................................................................   1       1    seq
    ....................ugauauguuugauauauuagguugu..................................................................   28      0    seq
    ....................ugauauguuugauauauuagguuUu..................................................................   2       1    seq
    ....................ugauauguuugauauauuagguuguu.................................................................   3       0    seq
    ........................................................acuauauaucaaacauauucc..................................   2       0    seq
    ........................................................acuauauaucaaacauauuccu.................................   30      0    seq
    .........................................................cuauauaucaaacauauuccu.................................   14      0    seq
```

Provisional ID	:	mun-miR-26
Score total	:	316243.7
Score for star read(s)	:	3.9
Score for read counts	:	316232.9
Score for mfe	:	2.3
Score for randfold	:	1.6
Score for cons. seed	:	3
Total read count	:	620289
Mature read count	:	619354
Loop read count	:	1
Star read count	:	934

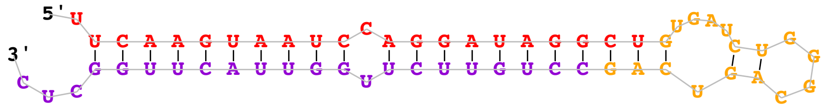

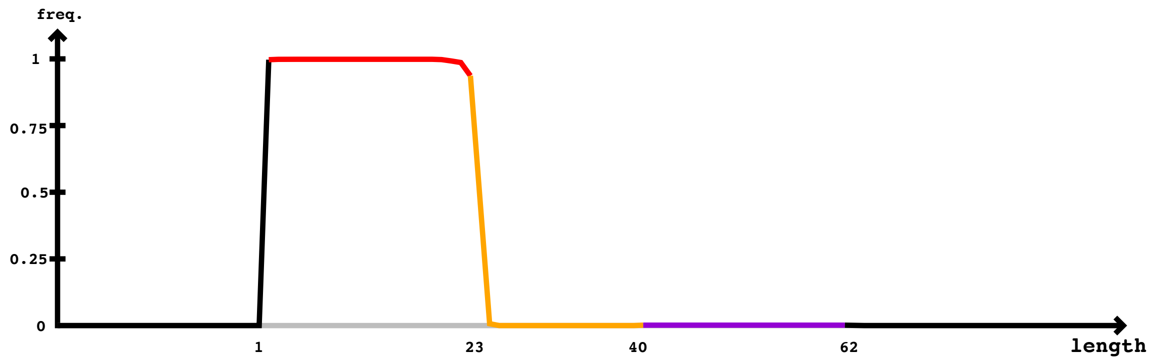


```
                        Mature                                  Star
5'- caguuguggcugguccccagguucaaguaauccaggauaggcugugaucugggcagucagccuguucuugguuacuuggcucuggagcuggccagaugcuucaccuggau  -3'   obs
    caguuguggcuggucccagguucaaguaauccaggauaggcugugaucugggcagucagccuguucuugguuacuuggcucuggagcuggccagaugcuucaccuggau        exp
    .((((.((((..(..(((((..((((((((.((((((((((....((....)).))))))))))).)))))))))..)))))..)..)))).).))).........   reads   mm   sample
    .............ucccUgguucaaguaauccaggauaggcu...................................................................   4       1    seq
    ...............ccGgguucaaguaauccaggauaggcu...................................................................   1       1    seq
    ................cGgguucaaguaauccaggauaggc.....................................................................   1       1    seq
    ...................Cuucaaguaauccaggauag......................................................................   1       1    seq
    ...................Cuucaaguaauccaggauaggc....................................................................   2       1    seq
    ...................Cuucaaguaauccaggauaggcu...................................................................   15      1    seq
    ...................Auucaaguaauccaggauaggcu...................................................................   10      1    seq
    ...................guucaaguaauccaggauaggcu...................................................................   1       0    seq
    ...................Uuucaaguaauccaggauaggcu...................................................................   1       1    seq
    ....................uucaaguaauccaggaua.......................................................................   313     0    seq
    ....................uucaaguaauccaggauG.......................................................................   2       1    seq
    ....................uucaaguaauccaggaAa.......................................................................   1       1    seq
    ....................uucaaguaaucUaggaua.......................................................................   1       1    seq
    ....................uucaGguaauccaggaua.......................................................................   1       1    seq
    ....................uucaaguaauccaggUua.......................................................................   1       1    seq
    ....................uucaaguaaucGaggaua.......................................................................   1       1    seq
    ....................uucaaguaauccaggauU.......................................................................   392     1    seq
    ....................uucaaguaauccaggauUg......................................................................   1       1    seq
    ....................uucaaguaaucUaggauag......................................................................   1       1    seq
    ....................uCcaaguaauccaggauag......................................................................   1       1    seq
    ....................Gucaaguaauccaggauag......................................................................   3       1    seq
    ....................uucaaguaauccGggauag......................................................................   1       1    seq
    ....................uucaaguaaCccaggauag......................................................................   1       1    seq
    ....................uucaaguaauccagAauag......................................................................   1       1    seq
    ....................Aucaaguaauccaggauag......................................................................   2       1    seq
    ....................uucaaguaauccaggauGg......................................................................   2       1    seq
    ....................uucaaguaauccaggauaU......................................................................   11      1    seq
    ....................uucaaguaauccaggauaA......................................................................   59      1    seq
    ....................uucaaguaauUcaggauag......................................................................   2       1    seq
    ....................uucaaguaauccaUgauag......................................................................   1       1    seq
    ....................uucaaguaauccUggauag......................................................................   1       1    seq
    ....................uucaaguaauccaggauag......................................................................   3115    0    seq
    ....................uucaagCaauccaggauag......................................................................   2       1    seq
```

Mature   Star

```
caguuguggcuggucccagguucaaguaauccaggauaggcugugaucugggcagucagccuguucuugguuacuuggcucuggagcuggccagaugcuucaccuggau
....................uucaaguUauccaggauag......................................................................    1      1     seq
....................uucaagAaauccaggauag......................................................................    3      1     seq
....................uuUaaguaauccaggauag......................................................................    1      1     seq
....................uucGaguaauccaggauag......................................................................    1      1     seq
....................uucaaguaauccGggauagg.....................................................................    1      1     seq
....................uucaaCuaauccaggauagg.....................................................................    2      1     seq
....................uucaaguaauAcaggauagg.....................................................................    1      1     seq
....................uucaaguaaAccaggauagg.....................................................................    1      1     seq
....................uucaaguaCuccaggauagg.....................................................................    1      1     seq
....................uucaaguaauccaggauagg.....................................................................    2382   0     seq
....................uucaagGaauccaggauagg.....................................................................    3      1     seq
....................uAcaaguaauccaggauagg.....................................................................    1      1     seq
....................uucaaguaauccaggauaAg.....................................................................    2      1     seq
....................uucaaguaauccaggCuagg.....................................................................    1      1     seq
....................uucaaguaauccaggauagC.....................................................................    4      1     seq
....................uucaaguaauccaggauagU.....................................................................    844    1     seq
....................Gucaaguaauccaggauagg.....................................................................    1      1     seq
....................uucaaguaauccaggGuagg.....................................................................    2      1     seq
....................uucaaguaaucAaggauagg.....................................................................    1      1     seq
....................uucaaguaauUcaggauagg.....................................................................    2      1     seq
....................uucaaguaauccaggauagA.....................................................................    366    1     seq
....................Cucaaguaauccaggauagg.....................................................................    3      1     seq
....................uucaagCaauccaggauagg.....................................................................    1      1     seq
....................uucaagAaauccaggauagg.....................................................................    2      1     seq
....................uucaaguaauccaggauCgg.....................................................................    1      1     seq
....................uucaaguaauccaggaGagg.....................................................................    1      1     seq
....................uCcaaguaauccaggauagg.....................................................................    1      1     seq
....................uucaaguaaucUaggauagg.....................................................................    1      1     seq
....................uucaaguaauccaggauaggA....................................................................    204    1     seq
....................uucaaguaauccagAauaggc....................................................................    2      1     seq
....................uucaaguaaGccaggauaggc....................................................................    2      1     seq
....................uucaaUuaauccaggauaggc....................................................................    3      1     seq
....................uucaaguaauccaggauagAc....................................................................    5      1     seq
....................uucaaguaauUcaggauaggc....................................................................    41     1     seq
....................uucaagAaauccaggauaggc....................................................................    3      1     seq
....................uucaaguaauccaUgauaggc....................................................................    3      1     seq
....................uucaaguaaAccaggauaggc....................................................................    1      1     seq
....................uucaaguaauAcaggauaggc....................................................................    4      1     seq
....................uucaaAuaauccaggauaggc....................................................................    3      1     seq
....................uucaagCaauccaggauaggc....................................................................    7      1     seq
....................uucaaguaauccaggauaAgc....................................................................    5      1     seq
....................uucaaguaCuccaggauaggc....................................................................    1      1     seq
....................uucaaguaauccaggaGaggc....................................................................    3      1     seq
....................uucaaguaauccaggauagCc....................................................................    1      1     seq
....................uucaaguaauccaggUuaggc....................................................................    1      1     seq
....................uucaaguaauccaggaCaggc....................................................................    2      1     seq
....................uucaaguaGuccaggauaggc....................................................................    2      1     seq
....................uucaGguaauccaggauaggc....................................................................    7      1     seq
....................uucaaguaauccaggauaCgc....................................................................    3      1     seq
....................uucaaguaauccaggauagUc....................................................................    3      1     seq
....................uucaaguaauccagCauaggc....................................................................    2      1     seq
....................uucaaguaaucGaggauaggc....................................................................    1      1     seq
....................uucaagGaauccaggauaggc....................................................................    11     1     seq
....................uucaaguUauccaggauaggc....................................................................    2      1     seq
....................uucaaguaauccaggGuaggc....................................................................    1      1     seq
....................uucGaguaauccaggauaggc....................................................................    6      1     seq
....................uucaaguaauccaggauaggc....................................................................    30074  0     seq
....................uucaaguaauccaggauaUgc....................................................................    6      1     seq
....................uucaaguaaucAaggauaggc....................................................................    11     1     seq
....................uucaaguaauGcaggauaggc....................................................................    2      1     seq
....................uAcaaguaauccaggauaggc....................................................................    1      1     seq
....................Aucaaguaauccaggauaggc....................................................................    21     1     seq
....................uuUaaguaauccaggauaggc....................................................................    1      1     seq
....................Cucaaguaauccaggauaggc....................................................................    12     1     seq
....................uucUaguaauccaggauaggc....................................................................    1      1     seq
....................Gucaaguaauccaggauaggc....................................................................    19     1     seq
....................uucaaguaauccagUauaggc....................................................................    1      1     seq
....................uCcaaguaauccaggauaggc....................................................................    2      1     seq
....................uucaaguaauccaggaAaggc....................................................................    3      1     seq
....................uucaaguaaucUaggauaggc....................................................................    56     1     seq
```

Mature   Star

```
caguuguggcuggucccagguucaaguaauccaggauaggcugugaucugggcagucagccuguucuugguuacuuggcucuggagcuggccagaugcuucaccuggau
....................uucaaguaauccaggauGggc....................................................................   2       1   seq
....................uuAaaguaauccaggauaggc....................................................................   2       1   seq
....................uucaaguaauccGggauaggc....................................................................   7       1   seq
....................uucaaguaauccaCgauaggc....................................................................   3       1   seq
....................uucaaguaauccaggauaggU....................................................................   242     1   seq
....................uuGaaguaauccaggauaggc....................................................................   1       1   seq
....................uucaaguGauccaggauaggc....................................................................   7       1   seq
....................uucaaguaauccaAgauaggc....................................................................   3       1   seq
....................uucaaguaauccaggauUggc....................................................................   2       1   seq
....................uucaaguaauccaggauaggG....................................................................   1       1   seq
....................uucaaguaaCccaggauaggc....................................................................   5       1   seq
....................uucaaguaaucGaggauaggcu...................................................................   33      1   seq
....................uucaaguaauccagUauaggcu...................................................................   77      1   seq
....................uucaaguaauccaggauagCcu...................................................................   14      1   seq
....................uucaaguaauccaggauagUcu...................................................................   72      1   seq
....................uucaaCuaauccaggauaggcu...................................................................   15      1   seq
....................uucaaguaauccaggUuaggcu...................................................................   42      1   seq
....................uucaaguaauccaggauaggcG...................................................................   4758    1   seq
....................uucaaguaaucAaggauaggcu...................................................................   103     1   seq
....................uucGaguaauccaggauaggcu...................................................................   66      1   seq
....................uucaCguaauccaggauaggcu...................................................................   7       1   seq
....................uucaaguaauGcaggauaggcu...................................................................   16      1   seq
....................uucaaguaauccaggauUggcu...................................................................   16      1   seq
....................uucaaguaauccaggauaggGu...................................................................   17      1   seq
....................uucaaguGauccaggauaggcu...................................................................   76      1   seq
....................uucaaguaaucUaggauaggcu...................................................................   954     1   seq
....................uAcaaguaauccaggauaggcu...................................................................   46      1   seq
....................uucaaguaauccaggauaCgcu...................................................................   14      1   seq
....................uucaaguaauccaggGuaggcu...................................................................   50      1   seq
....................uucaaguaauccaggauagAcu...................................................................   30      1   seq
....................uucaaguaauccaUgauaggcu...................................................................   76      1   seq
....................uucaUguaauccaggauaggcu...................................................................   18      1   seq
....................uucaaguaauccaCgauaggcu...................................................................   23      1   seq
....................uucaaguaUuccaggauaggcu...................................................................   9       1   seq
....................uucUaguaauccaggauaggcu...................................................................   26      1   seq
....................uucaaguaauccaAgauaggcu...................................................................   25      1   seq
....................uucaaguaCuccaggauaggcu...................................................................   4       1   seq
....................uucaagCaauccaggauaggcu...................................................................   89      1   seq
....................uucaGguaauccaggauaggcu...................................................................   89      1   seq
....................uCcaaguaauccaggauaggcu...................................................................   58      1   seq
....................uucaaguUauccaggauaggcu...................................................................   4       1   seq
....................uucaaguaauUcaggauaggcu...................................................................   321     1   seq
....................uucaaguaauccaggaCaggcu...................................................................   34      1   seq
....................uucaaguaauccaggauaggAu...................................................................   139     1   seq
....................uucCaguaauccaggauaggcu...................................................................   6       1   seq
....................uucaaguaauccaggauaggcu...................................................................   513040  0   seq
....................uucaaguaauccaggCuaggcu...................................................................   8       1   seq
....................uucaaguaauccaggauaggcC...................................................................   415     1   seq
....................uucaaguaauccaggauaggUu...................................................................   118     1   seq
....................uucaaguaaAccaggauaggcu...................................................................   30      1   seq
....................uucaaUuaauccaggauaggcu...................................................................   62      1   seq
....................uuAaaguaauccaggauaggcu...................................................................   60      1   seq
....................uucaaguaauccGggauaggcu...................................................................   65      1   seq
....................uucaaguaauccaggauGggcu...................................................................   42      1   seq
....................uucaaguaauccaggaGaggcu...................................................................   17      1   seq
....................uucaaguaauAcaggauaggcu...................................................................   67      1   seq
....................uucaagGaauccaggauaggcu...................................................................   80      1   seq
....................uGcaaguaauccaggauaggcu...................................................................   3       1   seq
....................uucaaguaauccaggaAaggcu...................................................................   21      1   seq
....................uucaagAaauccaggauaggcu...................................................................   58      1   seq
....................Gucaaguaauccaggauaggcu...................................................................   379     1   seq
....................uucaaguaauccagCauaggcu...................................................................   21      1   seq
....................uucaaguaaCccaggauaggcu...................................................................   46      1   seq
....................Aucaaguaauccaggauaggcu...................................................................   263     1   seq
....................uuUaaguaauccaggauaggcu...................................................................   46      1   seq
....................uucaaguaauccaggauCggcu...................................................................   8       1   seq
....................uucaaguaauccaggauaggcA...................................................................   10990   1   seq
....................uuGaaguaauccaggauaggcu...................................................................   11      1   seq
....................uucaaguaauccagAauaggcu...................................................................   24      1   seq
....................uucaaguaauccaggauaUgcu...................................................................   66      1   seq
```

```
                   Mature                                 Star
caguuguggcuggucccagguucaaguaaauccaggauaggcugugaucugggcagucagccuguucuugguuacuuggcucuggagcuggccagaugcuucaccuggau
....................uucaaguaaauccaggauaAgcu...................................................................  35     1  seq
....................uucaaguaGuccaggauaggcu...................................................................  28     1  seq
....................uucaaAuaaauccaggauaggcu...................................................................  26     1  seq
....................uucaaguCauccaggauaggcu...................................................................  5      1  seq
....................Cucaaguaaauccaggauaggcu...................................................................  157    1  seq
....................uucaaguaaGccaggauaggcu...................................................................  8      1  seq
....................uucaaguaaauccCggauaggcu...................................................................  15     1  seq
....................uucaaguaaauccUggauaggcu...................................................................  19     1  seq
....................uucaaguaaauccaggauaggcug..................................................................  1479   0  seq
....................uucaaguaaauccaggauaggcGg..................................................................  16     1  seq
....................uucaaguaaauccaggauaggcuC..................................................................  1182   1  seq
....................uucaaguaaauccaggauaggcAg..................................................................  14     1  seq
....................uAcaaguaaauccaggauaggcug..................................................................  1      1  seq
....................Aucaaguaaauccaggauaggcug..................................................................  1      1  seq
....................uucaaguaaaucUaggauaggcug..................................................................  1      1  seq
....................uucaaguaaauccaggauaggcCg..................................................................  1      1  seq
....................uucaaguaaauccaggauaggcuA..................................................................  2295   1  seq
....................uuAaaguaaauccaggauaggcug..................................................................  1      1  seq
....................uucaaguaaauccagUauaggcug..................................................................  1      1  seq
....................uucaaguaaauccaggauagAcug..................................................................  1      1  seq
....................uucGaguaaauccaggauaggcug..................................................................  1      1  seq
....................uucaagAaaauccaggauaggcug..................................................................  1      1  seq
....................Gucaaguaaauccaggauaggcug..................................................................  1      1  seq
....................uucaaguaaauccaggauaggcuU..................................................................  37129  1  seq
....................uucaaguaaauccaggauaggcuCu.................................................................  171    1  seq
....................uucaaguaaauccaggauaggcugu.................................................................  295    0  seq
....................uucaaguaaauccaggauaggcugG.................................................................  4      1  seq
....................uucaaguaaauccaggauaggcugA.................................................................  24     1  seq
....................uucaaguaaauccaggauaggcAgu.................................................................  1      1  seq
....................uucaaguaaauccaggauaggcugC.................................................................  5      1  seq
....................uucaaguaaauccaggauaggcuAu.................................................................  511    1  seq
....................uucaaCuaaauccaggauaggcugu.................................................................  1      1  seq
....................uucaaguaaauccaggaAaggcugu.................................................................  1      1  seq
....................uucGaguaaauccaggauaggcugu.................................................................  1      1  seq
....................uucaaguaaauccaggauaggcuUu.................................................................  3241   1  seq
....................uucaaguaaauccaggauaggcuguU................................................................  22     1  seq
....................uucaaguaaauccaggauaggcuUug................................................................  5      1  seq
....................uucaaguaaauccaggauaggcugGg................................................................  1      1  seq
....................uucaaguaaauccaggauaggcugugaucug..........................................................  1      0  seq
.....................ucaaguaaauccaggauag......................................................................  1      0  seq
.....................ucaaguaaauccaggauagU.....................................................................  1      1  seq
.....................ucaaguaaauccaggauaggU....................................................................  1      1  seq
.....................ucaaguaaauccaggauaggc....................................................................  42     0  seq
.....................Gcaaguaaauccaggauaggcu...................................................................  1      1  seq
.....................ucaGguaaauccaggauaggcu...................................................................  1      1  seq
.....................ucaaguaaaucUaggauaggcu...................................................................  2      1  seq
.....................ucaaguaaauccaggauaggcA...................................................................  7      1  seq
.....................ucaaguaaauccaggauaggcu...................................................................  567    0  seq
.....................ucaaguaaauccaggauaggcG...................................................................  10     1  seq
.....................uUaaguaaauccaggauaggcu...................................................................  2      1  seq
.....................Acaaguaaauccaggauaggcug..................................................................  1      1  seq
.....................ucaaguaaauccaggauaggcuU..................................................................  22     1  seq
.....................ucaaguaaauccaggauaggcug..................................................................  182    0  seq
.....................ucaaguaaauccaggauaggcuA..................................................................  3      1  seq
.....................ucaaguaaauccaggauaggcuC..................................................................  1      1  seq
.....................ucaaguaaauccaggauaggcGg..................................................................  3      1  seq
.....................ucaaguaaaucUaggauaggcug..................................................................  1      1  seq
.....................ucaaguaaauccaggauGggcug..................................................................  1      1  seq
.....................ucaaguaaauccaggauaggcuUu.................................................................  3      1  seq
.....................ucaaguaaauccaggauaggcugA.................................................................  8      1  seq
.....................ucaaguaaauccaggauaggcugu.................................................................  33     0  seq
.....................ucaaguaaauccaggauaggcuguU................................................................  1      1  seq
......................caaguaaauccaggauaggc....................................................................  1      0  seq
......................caaguaaauccaggauaggcA...................................................................  1      1  seq
......................caaguaaauccaggauaggcu...................................................................  46     0  seq
......................caaguaaauccaggauaggcuC..................................................................  1      1  seq
......................caaguaaauccaggauaggcugu.................................................................  1      0  seq
.......................aaguaaauccaggauaggcA...................................................................  1      1  seq
.......................aaguaaauccaggauaggcu...................................................................  26     0  seq
.......................aaguaaauccaggauaggcuU..................................................................  1      1  seq
```

```
                    Mature                                      Star
caguuguggcuggucccagguucaaguaauccaggauaggcugugaucugggcagucagccuguucuugguuacuuggcucuggagcuggccagaugcuucaccuggau
.......................aaguaauccaggauaggcuUu.................................................................    1     1    seq
........................aguaauccaggauaggcu...................................................................    21    0    seq
........................aguaauccaggauaggcuU..................................................................    1     1    seq
.........................................ugugaucugggcagucagc.................................................    1     0    seq
..........................................................gccuguucuugguuacuuggcuc............................    1     0    seq
...........................................................ccuguucuugguuacuuggc..............................    9     0    seq
...........................................................ccuguucuugguuacuuggcu.............................    43    0    seq
...........................................................ccuguucuugguuacuuggcuc............................    631   0    seq
...........................................................ccuguucuugUuuacuuggcuc............................    1     1    seq
...........................................................Acuguucuugguuacuuggcuc............................    1     1    seq
...........................................................ccuguucuugguuacuuggcuU............................    2     1    seq
...........................................................ccuguucuugguuacuuggcuA............................    1     1    seq
...........................................................Gcuguucuugguuacuuggcuc............................    1     1    seq
...........................................................ccuguucuugguuacuuggcuAu...........................    2     1    seq
...........................................................ccuguucuugguuacuugCcucu...........................    1     1    seq
...........................................................ccuguucuugguuacuuggcucu...........................    207   0    seq
...........................................................ccuguucuugguuacuuggcucA...........................    5     1    seq
...........................................................ccuguucuuggAuacuuggcucu...........................    1     1    seq
...........................................................ccuguucuugguuacuuggcucC...........................    4     1    seq
...........................................................ccuguucuugguuacuuggcucG...........................    1     1    seq
...........................................................ccuguucuugguuacuuggcucuA..........................    1     1    seq
...........................................................ccuguucuugguuacuuggcucuU..........................    9     1    seq
............................................................cuguucuugguuacuuggcuc............................    7     0    seq
............................................................cuguucuugguuacuuggcucu...........................    6     0    seq
```

Provisional ID	:	mun-miR-32
Score total	:	195.6
Score for star read(s)	:	3.9
Score for read counts	:	185.2
Score for mfe	:	1.9
Score for randfold	:	1.6
Score for cons. seed	:	3
Total read count	:	375
Mature read count	:	336
Loop read count	:	0
Star read count	:	39

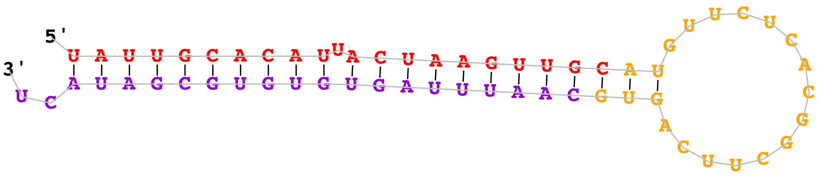

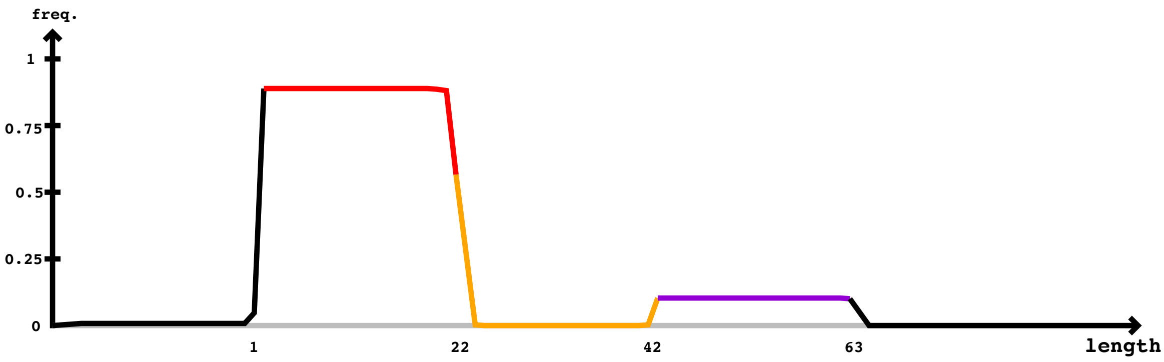


```
                         Mature                                  Star
5'- ucugcuugcucugguggagauauugcacauuacuaaguugcauguucucacggcuucagugcaauuuagugugugcgauacuuucacaugagugcaugcacacggguaugg  -3'  obs
    ucugcuugcucugguggagauauugcacauuacuaaguugcauguucucacggcuucagugcaauuuagugugugcgauacuuucacaugagugcaugcacacggguaugg       exp
    ..(((.(((.((.(((((((((((((((((.((((((((((((...............)))))))))))))))))))))).)))).))).)).))).)))...........  reads  mm  sample
    ucugcuugcucugguggaga...........................................................................................  2      0   seq
    .cugcuugcucugguggaga...........................................................................................  1      0   seq
    ...................auauugcacauuacuaaguug.......................................................................  7      0   seq
    ...................auauugcacauuacuaaguugA......................................................................  1      1   seq
    ...................auauugcacauuacuaaguugc......................................................................  7      0   seq
    ....................uauugcacauuacuaagu.........................................................................  1      0   seq
    ....................uauugcacauuacuaaguu........................................................................  2      0   seq
    ....................uauugcacauCacuaaguug.......................................................................  1      1   seq
    ....................uauugcacauuacuaaguug.......................................................................  111    0   seq
    ....................uauugcacauuacuaaguuAc......................................................................  1      1   seq
    ....................uauugcacauuacuaaguugc......................................................................  187    0   seq
    ....................uauugcacauuacuaaguugU......................................................................  1      1   seq
    ....................uauugcacauuacuaaguugca.....................................................................  16     0   seq
    ....................uauugcacauuacuaaguugcaA....................................................................  1      1   seq
    ............................................................gcaauuuagugugugcgauacu.............................  1      0   seq
    .............................................................caauuuagugugugcgauac..............................  1      0   seq
    .............................................................caauuuagugugugcgauacu.............................  37     0   seq
```

Provisional ID	:	mun-novel17
Score total	:	11.3
Score for star read(s)	:	3.9
Score for read counts	:	7.8
Score for mfe	:	2.4
Score for randfold	:	-2.2
Score for cons. seed	:	-0.6
Total read count	:	27
Mature read count	:	26
Loop read count	:	0
Star read count	:	1

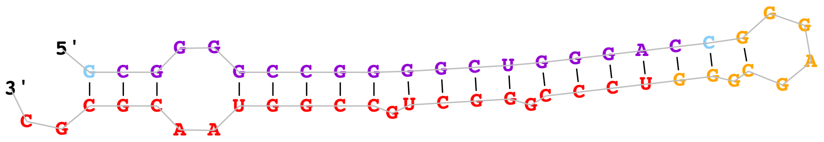

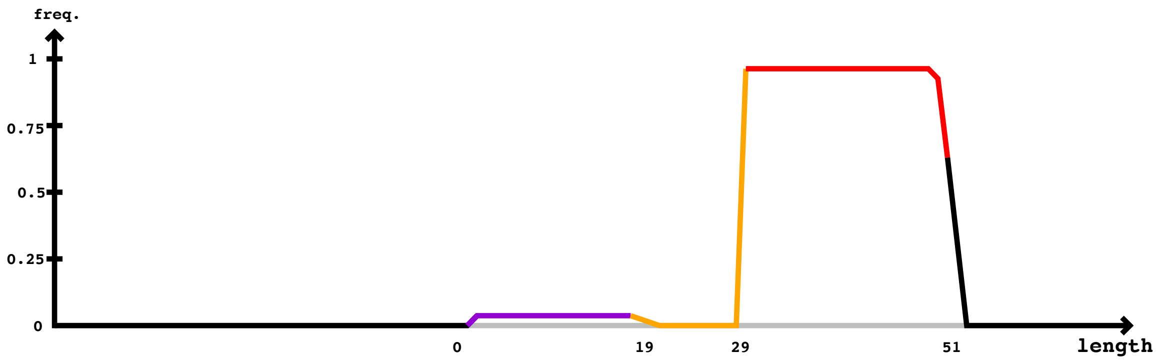

Star **Mature**

```
5'- acgcguggccgcagggcuucgggcagcucacgcaggugcgugcggggccggggcugggaccgggagcggggucccgggcugccgguaacgcgcggugccguugcaggugggg -3'   obs
    acgcguggccgcagggcuucgggcagcucacgcaggugcgugcggggccggggcugggaccgggagcggggucccgggcugccgguaacgcgcggugccguugcaggugggg        exp
    .(.((.((((....)))).)).)...((((((((((..((((((..((((((((((((((((....).))))))).)))).))))))..))))).)..))..)))..)))))   reads   mm   sample
    ..........................................cggggccggggcugggCc..................................................   1       1    seq
    ......................................................................ucccgggcugccgguaacgc....................   1       0    seq
    ......................................................................ucccgggcugccgguaacgcg...................   5       0    seq
    ......................................................................ucccgggcugccgguaacgcU...................   3       1    seq
    ......................................................................ucccgggcugccgguaacgcgA..................   5       1    seq
    ......................................................................ucccgggcugccgguaacgcgU..................   12      1    seq
```

Provisional ID	:	mun-novel62
Score total	:	1.3
Score for star read(s)	:	-1.3
Score for read counts	:	0
Score for mfe	:	1.6
Score for randfold	:	1.6
Score for cons. seed	:	-0.6
Total read count	:	22
Mature read count	:	22
Loop read count	:	0
Star read count	:	0

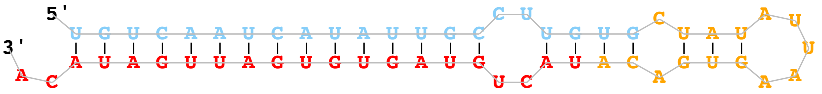

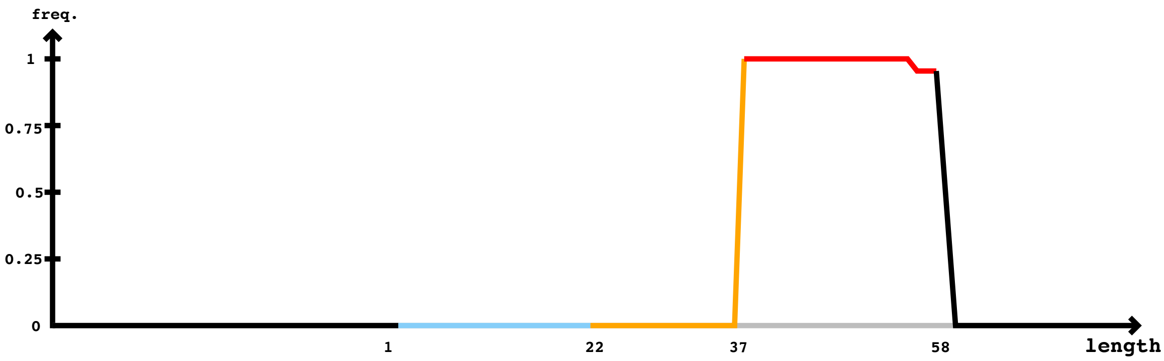

Star Mature

```
5'- gacguguuguguugugacaagucaucucagauugugucaaucauauugccuugugcuauauuaagugacauacuguagugugauugauacaacgugaaaugacacaucauu   -3'    exp
    ((.((((..(...((((....)))).(((..((((((((((((((((((..((((.(((.....))).))))..))))))))))))))))))..))).)..)))).))...    reads    mm    sample
    ......................................................................uacuguagugugauugau.......................    1        0     seq
    ......................................................................uacuguagugugauugauacU....................    1        1     seq
    ......................................................................uacuguagugugauugauaca....................    19       0     seq
    ......................................................................uacuguagugugauugauacaa...................    1        0     seq
```

Provisional ID	:	mun-novel63
Score total	:	5.1
Score for star read(s)	:	-1.3
Score for read counts	:	0
Score for mfe	:	1.8
Score for randfold	:	1.6
Score for cons. seed	:	3
Total read count	:	145
Mature read count	:	145
Loop read count	:	0
Star read count	:	0

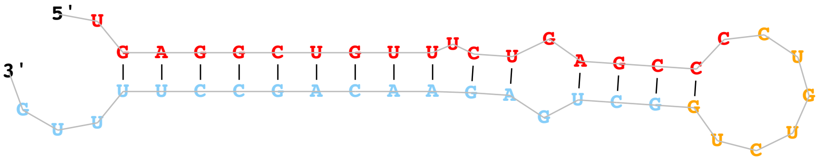

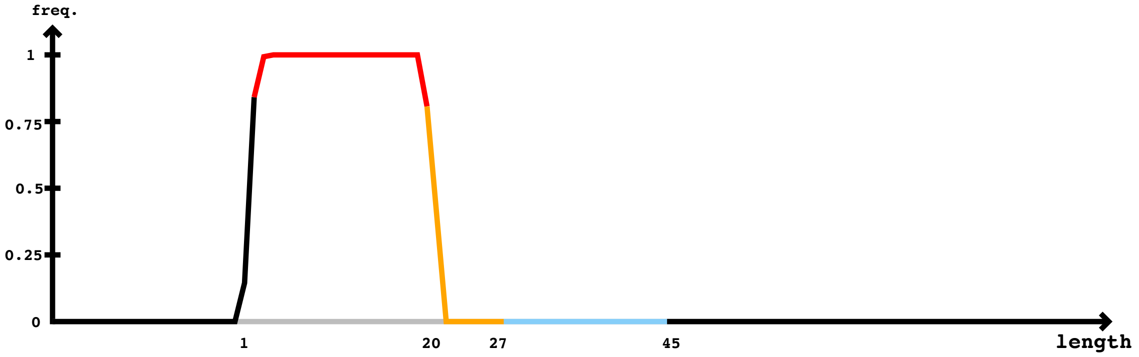

Mature Star

```
5'- uccagaaaaccaacgcugcugaggcuguuucugagccccugucuggcugagaacagccuuuugagcccaguccucaccauuguggccugggcagccccuaagccacua   -3'   exp
    ..............(((...(((((((((.((.((((.......)))).)))))))))))....((((((.((.((....)).)).))))))........))).....   reads   mm   sample
    ..................cAgaggcuguuucugagcc.......................................................................   18      1    seq
    ..................cAgaggcuguuucugagccc......................................................................   2       1    seq
    ..................cAgaggcuguuucugagcccc.....................................................................   1       1    seq
    ...................Agaggcuguuucugagcc.......................................................................   10      1    seq
    ...................Agaggcuguuucugagccc......................................................................   89      1    seq
    ...................Agaggcuguuucugagcccc.....................................................................   2       1    seq
    ....................gaggcuguuucugagcGc......................................................................   1       1    seq
    ....................gaggcuguuucugagccc......................................................................   15      0    seq
    ....................gaggcuguuucugagcccc.....................................................................   6       0    seq
    .....................aggcuguuucugagcccc.....................................................................   1       0    seq
```

Provisional ID	:	mun-novel50
Score total	:	347.8
Score for star read(s)	:	3.9
Score for read counts	:	337.1
Score for mfe	:	2.1
Score for randfold	:	1.6
Score for cons. seed	:	3
Total read count	:	673
Mature read count	:	303
Loop read count	:	2
Star read count	:	368

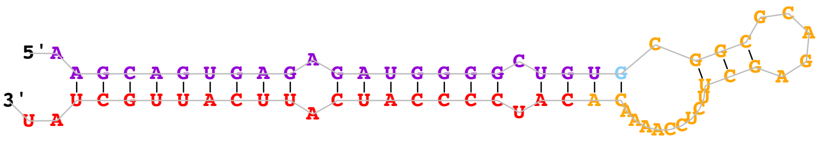

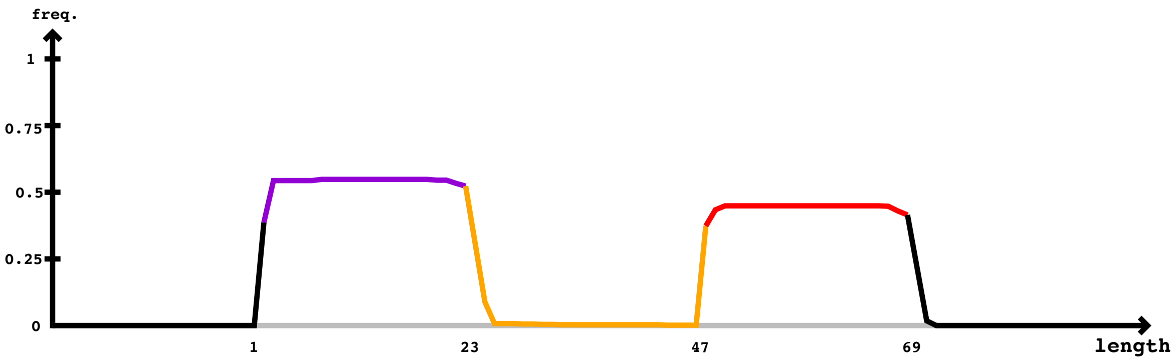


```
                         Star                                                Mature
5'- ugaggacgugccuccccggcaagcagugagagauggggcugugcggcgcagagcuucuccaaaacacauccccaucauucauugcuaucuggggcaccuccagcucuccgug   -3'    obs
    ugaggacgugccuccccggcaagcagugagagauggggcugugcggcgcagagcuucuccaaaacacauccccaucauucauugcuaucuggggcaccuccagcucuccgug          exp
    ...(((.(((((...((((..(((((((((.(((((((.((((.(((.....)))..........)))).))))))).)))))))))..))))))))).)))..........    reads    mm    sample
    ....................aagcagugagagaugggg..........................................................................    2        0     seq
    ....................aagcagugagagauggggcu........................................................................    8        0     seq
    ....................aagcagugagagauggggcug.......................................................................    6        0     seq
    ....................aGgcagugagagauggggcugu......................................................................    1        1     seq
    ....................aagcagugagagauggggcugG......................................................................    2        1     seq
    ....................aagcagugagagauggggcugu......................................................................    147      0     seq
    ....................aagcagugagagauggggcugA......................................................................    1        1     seq
    ....................aagcagugagagauggggcugug.....................................................................    1        0     seq
    ....................aagcagugagagauggggcuguU.....................................................................    77       1     seq
    ....................aagcagugagagauggggcuguA.....................................................................    12       1     seq
    ....................aagcagugagagauggggcugugU....................................................................    4        1     seq
    .....................agcagugagagauggggcug.......................................................................    3        0     seq
    .....................agcagugagagauggggcugu......................................................................    14       0     seq
    .....................agcagugagagauggggcuguU.....................................................................    18       1     seq
    .....................agcagugagagauggggcugug.....................................................................    17       0     seq
    .....................agcagugagagauggggcuguA.....................................................................    3        1     seq
    .....................agcagugagagauggggcugugA....................................................................    6        1     seq
    .....................agcagugagagauggggcugugU....................................................................    43       1     seq
    .....................agcagugagagauggggcugugG....................................................................    2        1     seq
    ..........................ugagagauggggcugugcggc.................................................................    1        0     seq
    ..........................ugagagauggggcugugcggcgU...............................................................    1        1     seq
    ..........................ugagagauggggcugugcggcgcag.............................................................    1        0     seq
    .........................................ugcggcgcagagcuucuccaa..................................................    1        0     seq
    .........................................ugcggcgcagagcuucuccaaaacac.............................................    1        0     seq
    ..................................................................cauccccaucauucauugc...........................    1        0     seq
    ..................................................................cauccccaucauucauugcu..........................    12       0     seq
    ..................................................................cauccccaucauucauugcuU.........................    1        1     seq
    ..................................................................cauccccaucauucauugcua.........................    7        0     seq
    ..................................................................cauccccaucauucauugcCau........................    1        1     seq
    ..................................................................cauccccaucauucauugcuGu........................    2        1     seq
    ..................................................................cauccccaucauucauugcuUu........................    1        1     seq
    ..................................................................cauccccaucauucauugcuau........................    206      0     seq
    ..................................................................cauccccaucauucauugcuauA.......................    1        1     seq
```

Star Mature

ugaggacgugccuccccggcaagcagugagagauggggcugugcggcgcagagcuucuccaaaacacauccccaucauucauugcuaucuggggcaccuccagcucuccgug			
....cauccccaucauucauugcuaCc.......................	2	1	seq
....cauccccaucauucauugcuauU.......................	17	1	seq
....auccccaucauucauugcua.........................	2	0	seq
....auccccaucauucauugcuau........................	7	0	seq
....auccccaucauucauugcuaCc.......................	1	1	seq
....auccccaucauucauugcuauc.......................	12	0	seq
....auccccaucauucauugcuauU.......................	15	1	seq
....auccccaucauucauugcuauA.......................	2	1	seq
....auccccaucauucauugcuaucu......................	1	0	seq
....auccccaucauucauugcuaucC......................	1	1	seq
....auccccaucauucauugcuauUu......................	1	1	seq
....uccccaucauucauugcuauU.......................	1	1	seq
....uccccaucauucauugcuauUu......................	5	1	seq
....uccccaucauucauugcuaucu......................	4	0	seq

Provisional ID	:	mun-miR-27
Score total	:	5.3
Score for star read(s)	:	-1.3
Score for read counts	:	0
Score for mfe	:	2
Score for randfold	:	1.6
Score for cons. seed	:	3
Total read count	:	46871
Mature read count	:	46836
Loop read count	:	0
Star read count	:	35

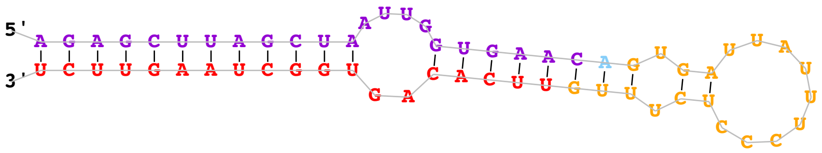

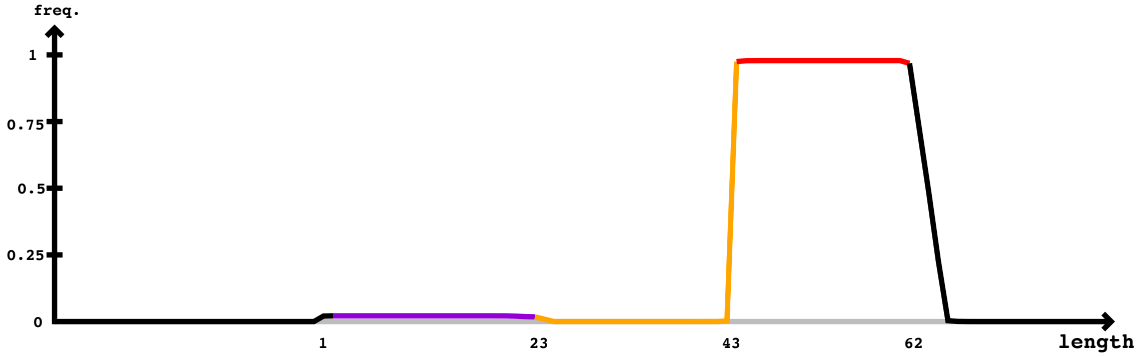

Star **Mature**

```
5'- aagacaggaaccucucuggcgaggugcagagcuuagcuaauuggugaacagugauuauuucccucuuuguucacaguggcuaaguucuacaccugaagagaaggguug  -3'   obs
    aagacaggaaccucucuggcgaggugcagagcuuagcuaauuggugaacagugauuauuucccucuuuguucacaguggcuaaguucuacaccugaagagaaggguug         exp
    ........((((((((..(.(((((.((((((((((((....((((((((.((.........)).))))))))..)))))))))))).)))))).)))))..)))).   reads   mm   sample
    ..........................cagagcuuagcuaauugg................................................................   4       0    seq
    ..........................cagagcuuagcuaauuggu...............................................................   2       0    seq
    ..........................cagagcuuagcuaauuggug..............................................................   49      0    seq
    ..........................cagagcuuagcuaauugguA..............................................................   1       1    seq
    ..........................cagagcuuagcuaauuggugga.............................................................   65      0    seq
    ..........................cagagcuuagcuaauuggugU.............................................................   3       1    seq
    ..........................cagagcuuagcuaauuggugG.............................................................   9       1    seq
    ..........................Aagagcuuagcuaauuggugga.............................................................   1       1    seq
    ..........................cagagcuuagcuaauuggugaa............................................................   41      0    seq
    ..........................cagagcuuagcuaauuggugaU............................................................   3       1    seq
    ..........................cagagcuuagcuaauuggGgaa............................................................   1       1    seq
    ..........................cagagcuuagcuaauuUgugaa............................................................   1       1    seq
    ..........................cagagcuuagcuaauuggugUac...........................................................   1       1    seq
    ..........................cagagcuuagcuaauuggugaac...........................................................   730     0    seq
    ..........................cagagcuuagcuaauuggugaaG...........................................................   3       1    seq
    ..........................cagagcuuagcuaauuggugaaA...........................................................   17      1    seq
    ..........................cagagcuuagcuaauuggugaaU...........................................................   3       1    seq
    ..........................cagagcuuagUuaauuggugaac...........................................................   2       1    seq
    ..........................cagagUuuagcuaauuggugaac...........................................................   1       1    seq
    ..........................cagagcuuagcuGauuggugaac...........................................................   1       1    seq
    ..........................cGgagcuuagcuaauuggugaac...........................................................   1       1    seq
    ..........................Aagagcuuagcuaauuggugaac...........................................................   1       1    seq
    ..........................cagagcuuagcuaauuggugaaca..........................................................   28      0    seq
    ..........................cagagcuuagcuaauuggugaacU..........................................................   24      1    seq
    ...........................agagcuuagcuaauuggugga.............................................................   2       0    seq
    ...........................agagcuuagcuaauuggugaac...........................................................   29      0    seq
    ...........................agagcuuagcuaauuggugaacU..........................................................   1       1    seq
    ...........................agagcuuagcuaauuggugaaca..........................................................   2       0    seq
    ...........................agagcuuagcuaauuggugaacagugau.....................................................   1       0    seq
    .................................................................uuuguucacaguggcuaaguucu....................   3       0    seq
    ....................................................................guucacaguggcuaaguuc.....................   1       0    seq
    ....................................................................Cuucacaguggcuaaguucu....................   1       1    seq
    ....................................................................guucacaguggcuaaguucu....................   38      0    seq
```

Star **Mature**

```
aagacaggaaccucucuggcgaggugcagagcuuagcuaauuggugaacagugauuauuucccucuuugguucacaguggcuaaguucuacaccugaagagaaggguug
....................................................................Auucacaguggcuaaguucu....................    1      1    seq
....................................................................guucacaguggcuaaguucC....................    3      1    seq
....................................................................guucacaguggcuaaguucua...................    10     0    seq
....................................................................guucacaguggcuaaguucuac..................    35     0    seq
....................................................................guucacaguggcuaaguucuaU..................    6      1    seq
....................................................................guucacaguggcuaaguucuGc..................    7      1    seq
....................................................................guucacaguggcuaaguucuacU.................    4      1    seq
....................................................................guucacaguggcuaaguucuaca.................    2      0    seq
....................................................................guucacaguggcuaaguucuacC.................    14     1    seq
....................................................................guucacaguggcuaaguucuacaA................    1      1    seq
.....................................................................uucacagCggcuaaguuc.....................    1      1    seq
.....................................................................Gucacaguggcuaaguuc.....................    1      1    seq
.....................................................................uucacaguUgcuaaguuc.....................    1      1    seq
.....................................................................uucacagugCcuaaguuc.....................    1      1    seq
.....................................................................uucacaguggcuaagGuc.....................    1      1    seq
.....................................................................uucacaguggcuaaguuU.....................    6      1    seq
.....................................................................uucacGguggcuaaguuc.....................    1      1    seq
.....................................................................uucacagAggcuaaguuc.....................    1      1    seq
.....................................................................uucacaguggcuaGguuc.....................    4      1    seq
.....................................................................uucacaguggcuaaUuuc.....................    1      1    seq
.....................................................................uucacaguggcuaaguuc.....................    473    0    seq
.....................................................................uucacagAggcuaaguucu....................    4      1    seq
.....................................................................uucacaguggcuaagGucu....................    3      1    seq
.....................................................................uucacaguggcuaaUuucu....................    1      1    seq
.....................................................................uucacaUuggcuaaguucu....................    2      1    seq
.....................................................................uucacaguggcCaaguucu....................    1      1    seq
.....................................................................uucacaguggcuaaguuAu....................    5      1    seq
.....................................................................uucacaguggcuaaguucA....................    22     1    seq
.....................................................................uAcacaguggcuaaguucu....................    2      1    seq
.....................................................................uucacaguggAuaaguucu....................    1      1    seq
.....................................................................uucacaguggcuaGguucu....................    1      1    seq
.....................................................................uucacagGggcuaaguucu....................    1      1    seq
.....................................................................uucacaguggcuaaguucG....................    10     1    seq
.....................................................................uucacagugAcuaaguucu....................    2      1    seq
.....................................................................uucacaguggcuCaguucu....................    2      1    seq
.....................................................................uucacaguggcuaaCuucu....................    1      1    seq
.....................................................................uucacagCggcuaaguucu....................    3      1    seq
.....................................................................uucacaguggcuaaguucu....................    7012   0    seq
.....................................................................Aucacaguggcuaaguucu....................    5      1    seq
.....................................................................uucacaguggUuaaguucu....................    5      1    seq
.....................................................................uuAacaguggcuaaguucu....................    1      1    seq
.....................................................................uucaAaguggcuaaguucu....................    2      1    seq
.....................................................................uucacaguggcuaUguucu....................    1      1    seq
.....................................................................uucacaguCgcuaaguucu....................    1      1    seq
.....................................................................Cucacaguggcuaaguucu....................    1      1    seq
.....................................................................uucacaguggcuGaguucu....................    1      1    seq
.....................................................................uucGcaguggcuaaguucu....................    3      1    seq
.....................................................................uucacagugUcuaaguucu....................    1      1    seq
.....................................................................uucacaguggcuaagAucu....................    4      1    seq
.....................................................................uucacaguUgcuaaguucu....................    2      1    seq
.....................................................................uucaGaguggcuaaguucu....................    1      1    seq
.....................................................................Gucacaguggcuaaguucu....................    4      1    seq
.....................................................................uucacaguggcuaaguuUu....................    2      1    seq
.....................................................................uucacaguggcuaaguucC....................    10095  1    seq
.....................................................................uucacaguggcuaagCucu....................    3      1    seq
.....................................................................uucacaguggcuaaguucua...................    1201   0    seq
.....................................................................uucaUaguggcuaaguucua...................    1      1    seq
.....................................................................uucacaguggcuaaguucAa...................    5      1    seq
.....................................................................uucacagugAcuaaguucua...................    2      1    seq
.....................................................................uucacaguggcuaagGucua...................    1      1    seq
.....................................................................uucacaguggcCaaguucua...................    1      1    seq
.....................................................................uucacaguggcuaaguucuU...................    190    1    seq
.....................................................................Aucacaguggcuaaguucua...................    1      1    seq
.....................................................................uucacaguAgcuaaguucua...................    1      1    seq
.....................................................................uucacagAggcuaaguucua...................    1      1    seq
.....................................................................Gucacaguggcuaaguucua...................    1      1    seq
.....................................................................uucacaguggcuaaguucCa...................    4327   1    seq
.....................................................................uucacaguCgcuaaguucua...................    1      1    seq
.....................................................................uucacCguggcuaaguucua...................    1      1    seq
.....................................................................uucacaguggcuaaguuGua...................    1      1    seq
```

Star **Mature**

```
aagacaggaaccucucuggcgaggugcagagcuuagcuaauuggugaacagugauuauuucccucuuuguucacaguggcuaaguucuacaccugaagagaaggguug
.....................................................................uucacaguggcuaaguucuC...................	5	1	seq
.....................................................................uucacaguggcuaaguucuG...................	105	1	seq
.....................................................................uCcacaguggcuaaguucuac..................	2	1	seq
.....................................................................uAcacaguggcuaaguucuac..................	1	1	seq
.....................................................................uucacaguAgcuaaguucuac..................	1	1	seq
.....................................................................uucacaguggcuaaguucuUc..................	2	1	seq
.....................................................................Gucacaguggcuaaguucuac..................	4	1	seq
.....................................................................uucacaguggcuaagAucuac..................	2	1	seq
.....................................................................uuAacaguggcuaaguucuac..................	1	1	seq
.....................................................................uucacagugAcuaaguucuac..................	1	1	seq
.....................................................................uucacaUggcuaaguucuac...................	1	1	seq
.....................................................................uucacaguggcuaaguuAuac..................	1	1	seq
.....................................................................uucacagugUcuaaguucuac..................	2	1	seq
.....................................................................uuUacaguggcuaaguucuac..................	3	1	seq
.....................................................................Cucacaguggcuaaguucuac..................	6	1	seq
.....................................................................uucacaguggcuaagGucuac..................	1	1	seq
.....................................................................uucacaguggcuGaguucuac..................	2	1	seq
.....................................................................uucacagAggcuaaguucuac..................	5	1	seq
.....................................................................uucaGaguggcuaaguucuac..................	1	1	seq
.....................................................................Aucacaguggcuaaguucuac..................	5	1	seq
.....................................................................uucaUaguggcuaaguucuac..................	2	1	seq
.....................................................................uucacaguggcuaaguucAac..................	1	1	seq
.....................................................................uucacagGggcuaaguucuac..................	6	1	seq
.....................................................................uucGcaguggcuaaguucuac..................	1	1	seq
.....................................................................uucacaguCgcuaaguucuac..................	1	1	seq
.....................................................................uucacaguggcuaaguGcuac..................	1	1	seq
.....................................................................uucacaguggcuaaguuUuac..................	5	1	seq
.....................................................................uucacaguggcuaaguucuac..................	9744	0	seq
.....................................................................uucacaguggcuaaguucuaA..................	37	1	seq
.....................................................................uucacaguggUuaaguucuac..................	1	1	seq
.....................................................................uuGacaguggcuaaguucuac..................	2	1	seq
.....................................................................uucacaguggcuaaguuGuac..................	2	1	seq
.....................................................................uucacaguggcuaaguucuaU..................	388	1	seq
.....................................................................uucacaguggcuaaUuucuac..................	2	1	seq
.....................................................................uucacaguggcuaaguucuGc..................	1207	1	seq
.....................................................................uucacagCggcuaaguucuac..................	1	1	seq
.....................................................................uucacagugCcuaaguucuac..................	1	1	seq
.....................................................................uucacGguggcuaaguucuac..................	3	1	seq
.....................................................................uucacaguggcuaaAuucuac..................	1	1	seq
.....................................................................uucacUguggcuaaguucuac..................	2	1	seq
.....................................................................uucacaguggAuaaguucuac..................	3	1	seq
.....................................................................uucacaguggcuaagCucuac..................	5	1	seq
.....................................................................uucacaguggcuaaguucCac..................	769	1	seq
.....................................................................uucacaguggcCaaguucuac..................	1	1	seq
.....................................................................uucacaguUgcuaaguucuac..................	4	1	seq
.....................................................................uucacaguggcuaaguucuaG..................	1	1	seq
.....................................................................uucGcaguggcuaaguucuaca.................	1	1	seq
.....................................................................uucacaguggcuaagAucuaca.................	2	1	seq
.....................................................................uucacagGggcuaaguucuaca.................	1	1	seq
.....................................................................uucacaguggcuaaguucuaca.................	3029	0	seq
.....................................................................uucaUaguggcuaaguucuaca.................	1	1	seq
.....................................................................Cucacaguggcuaaguucuaca.................	1	1	seq
.....................................................................uucacaguggcuaaguucuacG.................	17	1	seq
.....................................................................uucacaguggUuaaguucuaca.................	1	1	seq
.....................................................................uucacaguggcuaaguucuaUa.................	19	1	seq
.....................................................................uucacaguggcuaaguucuaAa.................	31	1	seq
.....................................................................uucacaguggcuaaguucuacC.................	3667	1	seq
.....................................................................Gucacaguggcuaaguucuaca.................	5	1	seq
.....................................................................uucacagAggcuaaguucuaca.................	2	1	seq
.....................................................................uucacagugAcuaaguucuaca.................	1	1	seq
.....................................................................uucacaguggcuaaguucuacU.................	3776	1	seq
.....................................................................uucacaguggcuaaguucCaca.................	20	1	seq
.....................................................................uucacaguUgcuaaguucuaca.................	1	1	seq
.....................................................................uucacGguggcuaaguucuaca.................	2	1	seq
.....................................................................uuAacaguggcuaaguucuaca.................	1	1	seq
.....................................................................uucacaguggcuaaguucuGca.................	52	1	seq
.....................................................................uucacaguggcuaaguuAuaca.................	1	1	seq
.....................................................................uucacaguggcuaagGucuaca.................	1	1	seq
.....................................................................uucacaguggcuaaguucuGcac................	1	1	seq
.....................................................................uucacaguggcuaaguucuacac................	7	0	seq
```

Star Mature

```
aagacaggaaccucucuggcgaggugcagagcuuagcuaauuggugaacagugauuauuucccucuuuguucacaguggcuaaguucuacaccugaagagaaggguug
.....................................................................uucacaguggcuaaguucuaAac................	1	1	seq
.....................................................................uucacaguggcuaaguucuacCc................	9	1	seq
.....................................................................uucacaguggcuaaguucuacaA................	100	1	seq
.....................................................................uucacaguggcuaaguucuacaU................	10	1	seq
.....................................................................uucacaguggcuaaguucuacacc...............	1	0	seq
.....................................................................uucacaguggcuaaguucuacacU...............	3	1	seq
.....................................................................uucacaguggcuaaguucCacacc...............	1	1	seq
......................................................................ucacaguggcuaaguucu....................	11	0	seq
......................................................................ucacaguggcuaaguucuU...................	1	1	seq
......................................................................ucacaguggcuaaguucua...................	1	0	seq
......................................................................ucacaguggcuaaguucuac..................	39	0	seq
......................................................................Ccacaguggcuaaguucuac..................	1	1	seq
......................................................................ucacaCuggcuaaguucuac..................	1	1	seq
......................................................................ucacaguggcuaaguucuacC.................	59	1	seq
......................................................................ucacaguggcuaaguucuaca.................	5	0	seq
......................................................................ucacaguggcuaaguucuacU.................	24	1	seq
......................................................................ucacaguggcuaaguucuacaA................	1	1	seq
......................................................................ucacaguggcuaaguucuacac................	2	0	seq
......................................................................ucacaguggcuaaguucuacCc................	2	1	seq
......................................................................ucacaguggcuaaguucuacacc...............	7	0	seq
......................................................................ucacaguggcuaaguucuacacU...............	2	1	seq
......................................................................ucacaguggcuaaguucuacaccu..............	1	0	seq
.......................................................................cacaguggcuaaguucua...................	1	0	seq
.......................................................................cacaguggcuaaguucuac..................	2	0	seq
.......................................................................cacaguggcuaaguucuacC.................	1	1	seq
.......................................................................cacaguggcuaaguucuaca.................	1	0	seq
.......................................................................cacaguggcuaaguucuacac................	1	0	seq
.......................................................................cacaguggcuaaguucuGcacc...............	2	1	seq
.......................................................................cacaguggcuaaguucuacacU...............	4	1	seq
.......................................................................cacaguggcuaaguucuacacc...............	2	0	seq
.......................................................................cacaguggcuaaguucuacaccu..............	1	0	seq
........................................................................acaguggcuaaguucuac..................	1	0	seq
........................................................................acaguggcuaaguucuacacc...............	2	0	seq
........................................................................acaguggcuaaguucuGcacc...............	2	1	seq
........................................................................acaguggcuaaguucuacaccu..............	2	0	seq
```

Provisional ID	:	mun-novel91
Score total	:	1972
Score for star read(s)	:	3.9
Score for read counts	:	1974.2
Score for mfe	:	-3.2
Score for randfold	:	-2.2
Score for cons. seed	:	-0.6
Total read count	:	3884
Mature read count	:	2009
Loop read count	:	1852
Star read count	:	23

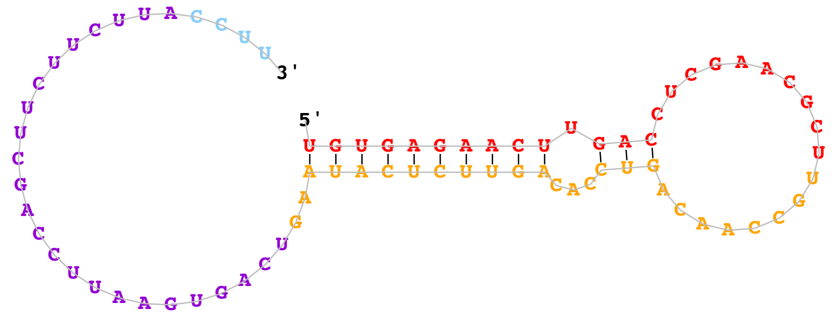

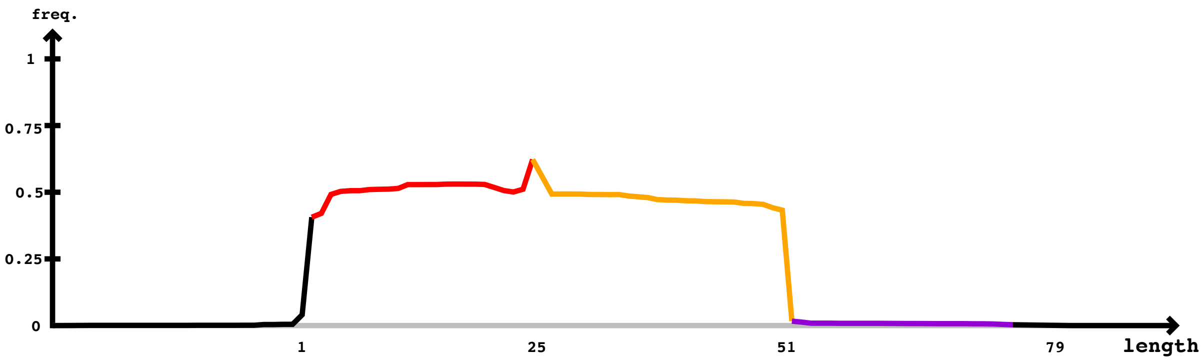

Mature Star

```
5'- uaacauauaauugguuaagcacaagugugagaacuugaccucgaacgcuugccaacaguccacaguucucauaagucagugaauuccagcuucuucuuaccuugcuugcuuaggc  -3'  obs
    uaacauauaauugguuaagcacaagugugagaacuugaccucgaacgcuugccaacaguccacaguucucauaagucagugaauuccagcuucuucuuaccuugcuugcuuaggc        exp
    ............(.(((((((((((.(((((((((.((..(((...(((((..(((........)))....)))))...)))..)).))....))))))))))))..)))))).)  reads  mm  sample
    uaacauaGaauugguuaagcaca..............................................................................................  1  1  seq
    .aacauauaauugguuaagc...............................................................................................  1  0  seq
    ..acauauaauugguuaagc...............................................................................................  1  0  seq
    .............guuaagcacaCgugugagaacuugaccucgaacgcu..................................................................  1  1  seq
    ..................gcacaCgugugagaacuugacc...........................................................................  1  1  seq
    ....................acaCgugugagaacuugaccu..........................................................................  1  1  seq
    ....................acaCgugugagaacuugaccucga.......................................................................  2  1  seq
    ....................acaagugugagaacuugaccucgaa......................................................................  1  0  seq
    ....................acaCgugugagaacuugaccucgaac.....................................................................  1  1  seq
    ....................acaagugugagaacuugaccucgaaA.....................................................................  4  1  seq
    ....................acaCgugugagaacuugaccucgaacgc...................................................................  1  1  seq
    ....................acaagugugagaacuugaccucgaacgcu..................................................................  1  0  seq
    ....................acaCgugugagaacuugaccucgaacgcu..................................................................  1  1  seq
    ....................acaagugugagaacuugaccucgaaAgcuugccaacagucc......................................................  1  1  seq
    ......................aagugugagaacuugaccucga.......................................................................  1  0  seq
    ......................aagugugagaacuugaccucgaaA.....................................................................  1  1  seq
    ......................aUgugugagaacuugaccucgaacgcu..................................................................  1  1  seq
    .......................Cgugugagaacuugaccucgaa......................................................................  1  1  seq
    ........................gugugagaacuugaccucg........................................................................  1  0  seq
    ........................gugugagaacuugaccucga.......................................................................  7  0  seq
    ........................gugugagaacuugaccucgaG......................................................................  1  1  seq
    ........................gugugagaacuugaccucgaa......................................................................  17  0  seq
    ........................gugugagaacuugaccucgaac.....................................................................  4  0  seq
    ........................gugugagaacuugaccucgaacg....................................................................  2  0  seq
    ........................gugugagaacuugaccucgaacgc...................................................................  15  0  seq
    ........................gugGgagaacuugaccucgaacgc...................................................................  1  1  seq
    ........................gugugagaaUuugaccucgaacgc...................................................................  1  1  seq
    ........................gugugagaacuugaccucgaacgcG..................................................................  1  1  seq
    ........................Cugugagaacuugaccucgaacgcu..................................................................  2  1  seq
    ........................gAgugagaacuugaccucgaacgcu..................................................................  1  1  seq
    ........................gugugagaacuugaccucgaacgcu..................................................................  88  0  seq
    ........................gugugagaacuugaccucgaacgcuu.................................................................  2  0  seq
    ........................gugugagaacuugaccucgaacgcuugcc..............................................................  1  0  seq
```

Mature **Star**

uaacauauaauugguuaagcacaagugugagaacuugaccucgaacgcuugccaacaguccacaguucucauaagucagugaauuccagcuucuucuuaccuugcuugcuuaggc			
........................gugugagaacuugaccucgaacgcuugccG...	1	1	seq
........................gugugagaacuugaccucgaacgcuugccaacaguccaca...	1	0	seq
.........................ugugagaacuugaccucg..	4	0	seq
.........................ugugagaacuugaccucCa...	1	1	seq
.........................ugugagaacuugaccucgG...	1	1	seq
.........................ugugagaacuugaccucga...	33	0	seq
.........................ugugagaacuugaccucgaa..	23	0	seq
.........................ugugagaacuugaccucgaG..	1	1	seq
.........................ugugagaacuugaccucgaac...	14	0	seq
.........................ugugagaacuugaccucgaacg..	19	0	seq
.........................ugugagaacuugaccucgUacgc...	1	1	seq
.........................ugugagaacuugaccucgaaGgc...	1	1	seq
.........................ugugagaacuugaccucgaacgU...	1	1	seq
.........................ugugagaacuugaccucgaacgc...	53	0	seq
.........................ugugagaaUuugaccucgaacgc...	1	1	seq
.........................Ggugagaacuugaccucgaacgcu..	2	1	seq
.........................ugugagGacuugaccucgaacgcu..	1	1	seq
.........................uAugagaacuugaccucgaacgcu..	1	1	seq
.........................ugugagaacuugaccucgaacgcA..	8	1	seq
.........................ugugagaacuugaccucgaGcgcu..	1	1	seq
.........................ugugagaacuugaccucgaacgcC..	1	1	seq
.........................ugugagaacuugaUcucgaacgcu..	1	1	seq
.........................ugugagaacuugaccucAaacgcu..	4	1	seq
.........................ugugagaacuugaccuAgaacgcu..	2	1	seq
.........................ugugagaGcuugaccucgaacgcu..	1	1	seq
.........................ugugagaacuugaccucgaacgcG..	14	1	seq
.........................ugugagaacuugacGucgaacgcu..	1	1	seq
.........................ugugagaacuugaGcucgaacgcu..	1	1	seq
.........................uguAagaacuugaccucgaacgcu..	2	1	seq
.........................ugugagaacuugaccucgaacgcu..	1237	0	seq
.........................ugCgagaacuugaccucgaacgcu..	2	1	seq
.........................ugugagaacuugaAcucgaacgcu..	1	1	seq
.........................ugugagaacuuUaccucgaacgcu..	1	1	seq
.........................ugugagaacuugaccucAaacgcuu...	1	1	seq
.........................ugugagaacuugaccucgaacgcuu...	13	0	seq
.........................ugugagaacuugaccucgaacgcuugc..	1	0	seq
.........................ugugagaacuugaccucgaacgcuugcc...	2	0	seq
.........................ugugagaacuugaccucgaacgAuugcc...	1	1	seq
.........................ugugagaacuugaccucgaacgcuugcca..	3	0	seq
.........................ugugagaacuugaccucgaacgcuugccaacag..	2	0	seq
.........................ugugagaacuugaccucgaacgcuugccaacagu...	1	0	seq
.........................ugugagaacuugaccucgaacgcuugccaacaguc..	2	0	seq
.........................ugugagaacuugaccucgaacgcuugccaacagucc...	16	0	seq
.........................ugugagaacuugaccucgaacgcuugccaacagucca..	4	0	seq
.........................ugugagaacuugaccucgaacgcuugccaacaguccac...	1	0	seq
.........................ugugagaacuugaccucgaaUgcuugccaacaguccaca...	2	1	seq
.........................ugugagaacuugaccucgaaUgcuugccaacaguccacagu...	2	1	seq
..........................gugagaacuugaccucga..	3	0	seq
..........................gugagaacuugaccucgaa...	8	0	seq
..........................gugagaacuugaccucgaacg...	3	0	seq
..........................gugagaacuugaccucgaacgc..	2	0	seq
..........................gugagaacuugaccucgaacgcu...	42	0	seq
..........................gugagaacuugaccucgaacgcuugcc..	1	0	seq
...........................ugagaacuugaccucgaa..	5	0	seq
...........................ugagaacuugaccucgaac...	2	0	seq
...........................ugagaacuugaccucgaacg..	3	0	seq
...........................ugagaacuugaccucgaacgc...	15	0	seq
...........................ugagaacuugaccucAaacgcu..	1	1	seq
...........................ugagaacuugaccucgaacUcu..	1	1	seq
...........................Agagaacuugaccucgaacgcu..	1	1	seq
...........................ugagaacuugaccucgaacgcA..	4	1	seq
...........................uAagaacuugaccucgaacgcu..	1	1	seq
...........................ugagaacuugaccucgaacgcG..	3	1	seq
...........................ugagaacuugaccucgaacgcC..	1	1	seq
...........................ugagaaUuugaccucgaacgcu..	1	1	seq
...........................ugagaacuugaccucgaacgcu..	234	0	seq
...........................ugagaacuugaccucgaacgcuA...	1	1	seq
...........................ugagaacuugaccucgaacgcuu...	4	0	seq
...........................ugagaacuugaccucgaacgcuugcc...	6	0	seq
...........................ugagaacuugaccucgaacgcuugcca..	1	0	seq

Mature **Star**

uaacauauaauugguuaagcacaagugugagaacuugaccucgaacgcuugccaacaguccacaguucucauaagucagugaauuccagcuucuucuuaccuugcuugcuuaggc			
...........................ugagaacuugaccucgaacgcuugccaacagu..	1	0	seq
...........................ugagaacuugaccucgaacgcuugccaacaguccaca...	1	0	seq
...........................ugagaacuugaccucgaacAcuugccaacaguccacagu...	1	1	seq
...........................ugagaacuugaccucgaaUgcuugccaacaguccacaguuc...	1	1	seq
............................gagaacuugaccucgaacgc...	3	0	seq
............................gagaacuugacUucgaacgcu..	1	1	seq
............................gagaacuugaccucgaacgcu..	30	0	seq
............................gagaacuugaccucgaacgcuugU...	1	1	seq
............................gagaacuugaccucgaacgcuugcc..	3	0	seq
............................gagaacuugaccucgaacgcuugccU...	1	1	seq
............................gagaacuugaccucgaacgcuugccaa..	1	0	seq
............................gagaacuugaccucgaacgcuugccaacag...	1	0	seq
............................gagaacuugaccucgaacgcuugccaacagucc..	1	0	seq
............................gagaacuugaccucgaacgcuugccaacagucca...	1	0	seq
............................gagaacuugaccucgaaUgcuugccaacaguccaca...	1	1	seq
............................gagaacuugaccucgaaUgcuugccaacaguccacag..	1	1	seq
............................gagaacuugaccucgaaUgcuugccaacaguccacaguucu..	1	1	seq
.............................agaacuugaccucgaacg..	2	0	seq
.............................agaacuugaccucgaacgc...	3	0	seq
.............................agaacuugaccucgaacgcu..	4	0	seq
.............................agaacuugaccucgaaUgcuugccaacaguccaca...	1	1	seq
.............................agaacuugaccucgaaUgcuugccaacaguccacagu...	1	1	seq
...............................aacuugaccucgaacgcu..	13	0	seq
...............................aacuugaccucgaacgcuu...	1	0	seq
...............................aacuugaccucgaacgcuugccaaA...	1	1	seq
...............................aacuugaccucgaacgcuugccaacagucc..	2	0	seq
................................acuugaccucgaacgcuugccaaca..	1	0	seq
................................acuugaccucgaacgcuugccaacagucc..	1	0	seq
................................acuugaccucgaacgcuugccaacaguccaca...	1	0	seq
................................acuugaccucgaacgcuugccaacaguccacag..	1	0	seq
.................................cuugaccucgaacgcuugccaacagucc..	3	0	seq
..................................uugaccucgaacgcuugccaacag...	3	0	seq
..................................uugaccucgaacgcuugccaacagu..	3	0	seq
..................................uugaccucgaacgcuugccaacagucc..	1	0	seq
..................................uugaccucgaacAcuugccaacaguccacagu...	1	1	seq
..................................uugaccucgaacAcuugccaacaguccacaguucucau...	1	1	seq
..................................uugaccucgaacgcuugccaacaguccacaguucucauaaC..	1	1	seq
...................................ugaccucgUacgcuugcc..	1	1	seq
...................................ugaccucgaacgcuugccU...	1	1	seq
...................................ugaccucgaacgcuugcca...	1	0	seq
...................................ugaccucgaacgcuugccaac...	1	0	seq
...................................ugaccucgaacgcuugccaaca..	2	0	seq
...................................ugaccucgaacgcuugccaacaU...	1	1	seq
...................................ugaccucgaacgcuugUcaacag...	1	1	seq
...................................ugaccucgaacgcuugccaacag...	14	0	seq
...................................ugaccucgaacgcuugccaGcagu..	1	1	seq
...................................ugaccucgaacgcuugccaacaUu..	1	1	seq
...................................ugaccucgaacgUuugccaacagu..	1	1	seq
...................................ugaccucgaacgcuugccaacagu..	3	0	seq
...................................ugaccucgaacgcuugUcaacagu..	1	1	seq
...................................ugaccucgaacgcuugccaacaguc...	9	0	seq
...................................ugaccucgaacgcuugccGacaguc...	1	1	seq
...................................ugaccucgaacgcuugccaacagucU..	2	1	seq
...................................ugCccucgaacgcuugccaacagucc..	1	1	seq
...................................ugaccucgaacgcuugccaacagCcc..	1	1	seq
...................................ugaccucgaacgcuugccaacagucca...	2	0	seq
...................................ugaccucgaacgcuugccaacaguccac..	1	0	seq
...................................ugaccucgaaUgcuugccaacaguccaca...	1	1	seq
...................................ugaccucgaacAcuugccaacaguccaca...	1	1	seq
...................................ugaccucgaacAcuugccaacaguccacagu...	3	1	seq
...................................ugaccucgaaUgcuugccaacaguccacagu...	1	1	seq
...................................ugaccucgaacgcuugccaacaguccacagu...	3	0	seq
...................................ugaccucgaacgcuugccaacaguccacaguuc...	1	0	seq
...................................ugaccucgaacAcuugccaacaguccacaguuc...	1	1	seq
...................................ugaccucgaacgcuugccaacaguccacaguucucauaaC..	1	1	seq
...................................ugaccucgaacgcuugccaacaguccacaguucucauaaCu.......................................	1	1	seq
.....................................accucgaacgcuugccaacagucc..	1	0	seq
....cucgaacgcuugccaacaguc...	1	0	seq
....cucgaacgcuugccaacaguccacaguucucauaaC..	5	1	seq
....ucgaacgcuugcUaacagucc..	1	1	seq

Mature Star

```
uaacauauaauugguuaagcacaagugugagaacuugaccucgaacgcuugccaacaguccacaguucucauaagucagugaauuccagcuucuucuuaccuugcuugcuuaggc
........................................ucgaaUgcuugccaacaguccacaguuc...............................................	1	1	seq
............................................acgcuugccaacaguccacaguucu..............................................	1	0	seq
............................................acgcuugccaacaguccacaguucucauaaC........................................	1	1	seq
.............................................cgcuugccaacaguccacagCucu..............................................	1	1	seq
.............................................Ugcuugccaacaguccacaguucuca............................................	1	1	seq
.............................................Ugcuugccaacaguccacaguucucau...........................................	1	1	seq
.............................................Ugcuugccaacaguccacaguucucaua..........................................	1	1	seq
.............................................cgcuugccaacaguccacaguucucauaaC........................................	6	1	seq
..............................................gcuugccaacaguccacaguucucaua..........................................	1	0	seq
..............................................gcuugccaacaguccacaguucucauaaC........................................	3	1	seq
...............................................cuugccaacaguccacaguucuc.............................................	1	0	seq
...............................................cuugccaacaguccacaguucuca............................................	1	0	seq
...............................................cuugccaacaguccacaguucucauU..........................................	1	1	seq
...............................................cuugccaacaguccacaguucucaua..........................................	2	0	seq
...............................................cuugccaacaguccacaguucucauaa.........................................	1	0	seq
...............................................cuugccaacaguccacaguucucauaaC........................................	65	1	seq
................................................uugccaacaguccacaguu................................................	1	0	seq
................................................uugccaacaguccacaguucuc.............................................	6	0	seq
................................................uugccaacagucAacaguucucau...........................................	1	1	seq
................................................uugccaacaguccacaguucucau...........................................	5	0	seq
................................................uugccaacagAccacaguucucau...........................................	1	1	seq
................................................uugccaacaguccacaguucuUaua..........................................	1	1	seq
................................................uugccaacaguccacaguucucaua..........................................	20	0	seq
................................................uugccaGcaguccacaguucucaua..........................................	1	1	seq
................................................uugccaacaguccacaguucucaAaa.........................................	1	1	seq
................................................uugccaacaguccacaguucucauaa.........................................	12	0	seq
................................................uugccaacaguccacaguucucauaaC........................................	493	1	seq
................................................uugccaacaguccacaguucucauaag........................................	2	0	seq
................................................uugccaacaguccacaguucucauaaUu.......................................	1	1	seq
................................................uugccaacaguccacaguucucauaaCu.......................................	3	1	seq
................................................uugccaacaguccacaguucucauaaCuc......................................	2	1	seq
................................................uugccaacagAccacaguucucauaagucagugaauucc............................	1	1	seq
.................................................ugccaacaguccacaguu................................................	1	0	seq
.................................................ugccaacaguccacaguucu..............................................	1	0	seq
.................................................ugccaacaguccacaguucuc.............................................	14	0	seq
.................................................ugccaacaguccacaguucucU............................................	1	1	seq
.................................................ugccaacaguccacaguucuca............................................	1	0	seq
.................................................ugccaacaguccacaguucucau...........................................	2	0	seq
.................................................ugccaacaguccacaguucucaua..........................................	22	0	seq
.................................................ugUcaacaguccacaguucucaua..........................................	1	1	seq
.................................................ugccaacaguccacaguuUucaua..........................................	2	1	seq
.................................................ugccaacaguccacaguucucauaa.........................................	22	0	seq
.................................................ugccaacaguccacaguucucauaaA........................................	2	1	seq
.................................................ugccaacaguccacaguucucauaag........................................	3	0	seq
.................................................ugccaacaguccacaguucucauaaC........................................	1091	1	seq
.................................................ugccaacaguccacaguucucauaaCu.......................................	9	1	seq
.................................................ugccaacaguccacaguucucauaaCuc......................................	14	1	seq
.................................................ugccaacaguccacaguucucauaaCucagu...................................	1	1	seq
.................................................ugccaacaguccacaguucucauaaCucagugaauu..............................	1	1	seq
.................................................ugccaacaguccacaguucucauaaCucagugaauuccag..........................	1	1	seq
..................................................gccaacaguccacaguucuc.............................................	1	0	seq
..................................................gccaacaguccacaguucucauaa.........................................	1	0	seq
..................................................gccaacaguccacaguucucauaaC........................................	18	1	seq
...................................................ccaacaguccacaguucucauaaC........................................	1	1	seq
....................................................caacaguccacaguucucauaaC........................................	1	1	seq
....................................................caacaguccacaguucucauaaCucagu...................................	1	1	seq
.....................................................aacaguccacaguucucauaaC........................................	13	1	seq
.....................................................aacaguccacaguucucauaaCuc......................................	1	1	seq
......................................................acaguccacaguucucauaaC........................................	2	1	seq
.......................................................caguccacaguucucauaaC........................................	1	1	seq
.........................................................guccacaguucucauaaC........................................	4	1	seq
............................................................cacaguucucauaagucagugaauuc.............................	1	0	seq
..................................................................ucucauaaCucagugaauuccagc.........................	1	1	seq
....................................................................ucauaaCucagugaauuccagcuucu.....................	1	1	seq
....................................................................ucauaaCucagugaauuccagcuucuuc...................	1	1	seq
....................................................................ucauaaCucagugaauuccagcuucuucu..................	1	1	seq
.....................................................................cauaagucagugaauuccagcuucuuc...................	1	0	seq
......................................................................auaaCucagugaauuccagcuucuuc...................	1	1	seq
.......................................................................uaaCucagugaauuccagcuucuucuu.................	1	1	seq
...........................................................................ucagugaaCuccagcuucu.....................	1	1	seq
```

```
                        Mature                                              Star
uaacauauaauugguuaagcacaagugugagaacuugaccucgaacgcuugccaacaguccacaguucucauaagucagugaauuccagcuucuucuuaccuugcuugcuuaggc
...........................................................................ucagugaauuccagcuucuucu..................   4   0   seq
...........................................................................ucGgugaauuccagcuucuucu..................   1   1   seq
...........................................................................ucagugaauuccagcuucuucuu.................   5   0   seq
...........................................................................ucagugaauCccagcuucuucuua................   1   1   seq
...........................................................................ucGgugaauuccagcuucuucuua................   1   1   seq
...........................................................................ucagugaauuccagcuucuucuua................   5   0   seq
...........................................................................ucagugaauuccagcCucuucuua................   1   1   seq
...........................................................................ucagugaauuccagcuucuucuuaA...............   2   1   seq
...........................................................................ucagugaauucUagcuucuucuuacc..............   1   1   seq
...........................................................................ucagugaauuccagcuucuucuuaccu.............   1   0   seq
```

Provisional ID	:	mun-miR-375
Score total	:	5.4
Score for star read(s)	:	-1.3
Score for read counts	:	0
Score for mfe	:	2.1
Score for randfold	:	1.6
Score for cons. seed	:	3
Total read count	:	4603
Mature read count	:	4603
Loop read count	:	0
Star read count	:	0

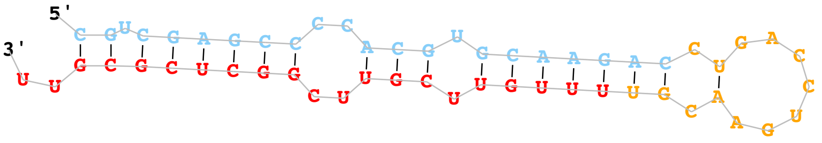

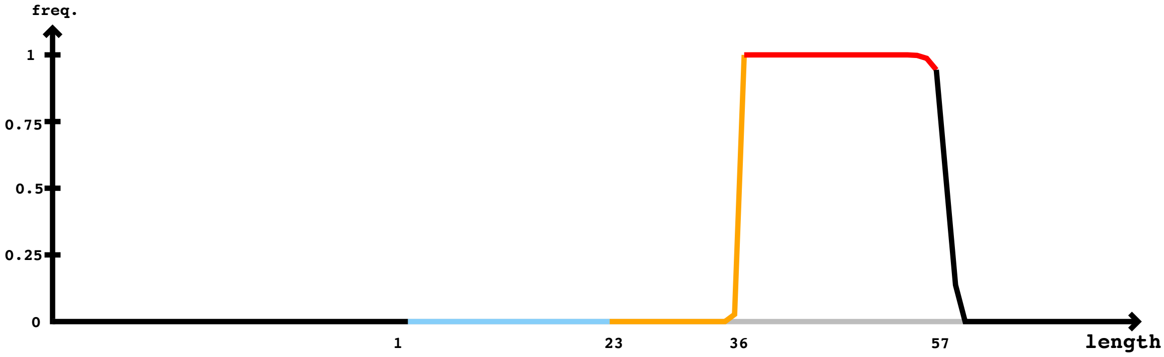


```
                                                   Star                   Mature
5'- cugucccagccuggagcaccucugcacuugccuggcgucgagccccacgugcaagaccugaccugaacguuuuguucguucggcucgcguuaggcagguccagcccaucug   -3'   exp
    .((..((.....))..))...(((.(((((((((((.((((((..(((.(((((((.(.......).))))))).)))..))))))))))))))))).)))........   reads   mm   sample
    ......................................................................uuuuguucguucggcucgc......................   1       0    seq
    ......................................................................uuuuguucguucggcucgcg.....................   6       0    seq
    ......................................................................uuuuguucguucggcucgcgu....................   16      0    seq
    ......................................................................uuuuguucguucggcucgcgA....................   1       1    seq
    ......................................................................uuuuguucguucggcucgcguu...................   77      0    seq
    ......................................................................uuuuguucguucggcucgcguuU..................   14      1    seq
    ......................................................................uuuuguucguucggcucgcguua..................   9       0    seq
    .......................................................................uuuguucguucggcucgc......................   7       0    seq
    .......................................................................uuuguucguucggcucgA......................   1       1    seq
    .......................................................................uuuguucguucggcucgcg.....................   43      0    seq
    .......................................................................uuuguucguucggcucgcU.....................   1       1    seq
    .......................................................................uCuguucguucggcucgcg.....................   1       1    seq
    .......................................................................uuuguucguuUggcucgcgu....................   1       1    seq
    .......................................................................uuuguucguucggcucgcgA....................   10      1    seq
    .......................................................................uuuguCcguucggcucgcgu....................   1       1    seq
    .......................................................................uuuguAcguucggcucgcgu....................   1       1    seq
    .......................................................................Guuguucguucggcucgcgu....................   1       1    seq
    .......................................................................uuuguucguucggcucgcgG....................   5       1    seq
    .......................................................................uuuguucguucggcucgcUu....................   1       1    seq
    .......................................................................uuuguucguucggcucgcgu....................   161     0    seq
    .......................................................................uuuguucguucggcucAcguu...................   1       1    seq
    .......................................................................uuuguucguucggcucUcguu...................   1       1    seq
    .......................................................................uuuguuAguucggcucgcguu...................   2       1    seq
    .......................................................................Guuguucguucggcucgcguu...................   2       1    seq
    .......................................................................uuuguucguucggUucgcguu...................   4       1    seq
    .......................................................................Auuguucguucggcucgcguu...................   2       1    seq
    .......................................................................uuuguucguucggcuUgcguu...................   3       1    seq
    .......................................................................uuuguucguucggcucgcguu...................   2025    0    seq
    .......................................................................uuuguucguucggcucgcguA...................   8       1    seq
    .......................................................................uuuguucguucggcucgcguG...................   3       1    seq
    .......................................................................uuCguucguucggcucgcguu...................   1       1    seq
    .......................................................................uuuguucguuAggcucgcguu...................   1       1    seq
    .......................................................................uuuguucguuUggcucgcguu...................   3       1    seq
    .......................................................................uuuguucUuucggcucgcguu...................   1       1    seq
```

```
                                               Star                                          Mature
cugucccagccuggagcaccucugcacuugccuggcgucgagccccacgugcaagaccugaccugaacguuuuguucguucggcucgcguuaggcagguccagcccaucug
......................................................................uuuguuGguucggcucgcguu....................   1      1      seq
......................................................................uuuguucguucggcucCcguu....................   1      1      seq
......................................................................uuuguucguucggcucgcUuu....................   1      1      seq
......................................................................uuuguucguucggcucgUguu....................   1      1      seq
......................................................................uuuguucguucggcucgcgGu....................   8      1      seq
......................................................................uuuguucguucggcucgcgCu....................   1      1      seq
......................................................................uuuguucguucggcucgcguC....................   2      1      seq
......................................................................uuuguuUguucggcucgcguu....................   2      1      seq
......................................................................uuuguucguucggcucgAguu....................   1      1      seq
......................................................................uuuguucguucUgcucgcguu....................   1      1      seq
......................................................................uuuguucguucggAucgcguu....................   3      1      seq
......................................................................uuuguucguucggcucgcguuU...................   253    1      seq
......................................................................uuuguucguucggcucgAguua...................   1      1      seq
......................................................................uuuguucguucggcucgcguAa...................   4      1      seq
......................................................................uuuguucguucggcucgcgCua...................   1      1      seq
......................................................................uuuguucguucUgcucgcguua...................   1      1      seq
......................................................................uuuguucguucggcucgcguua...................   1246   0      seq
......................................................................Guuguucguucggcucgcguua...................   3      1      seq
......................................................................uuuguucguucggcucgcguGa...................   2      1      seq
......................................................................uuuguucguucggcucgcguCa...................   6      1      seq
......................................................................uuuguucguuUggcucgcguua...................   3      1      seq
......................................................................uuuguucguucggcucgcguuG...................   2      1      seq
......................................................................uuuguucguucggcucgcguuC...................   3      1      seq
......................................................................uuuguucgGucggcucgcguua...................   1      1      seq
......................................................................Cuuguucguucggcucgcguua...................   2      1      seq
......................................................................uuuguucguucgCcucgcguua...................   1      1      seq
......................................................................uuuguucguCcggcucgcguua...................   1      1      seq
......................................................................uuuguucguucggcucgcgGua...................   4      1      seq
......................................................................uuuguucguucggcucgcguuaC..................   1      1      seq
......................................................................uuuguucguucggcucgcguuag..................   1      0      seq
......................................................................uuuguucguucggcucgcguuaA..................   82     1      seq
......................................................................uuuguucguucggcucgcguuaU..................   547    1      seq
.......................................................................uuguucguucggcucgcguua...................   1      0      seq
```

Provisional ID	:	mun-novel20
Score total	:	1.8
Score for star read(s)	:	-1.3
Score for read counts	:	0
Score for mfe	:	2.1
Score for randfold	:	1.6
Score for cons. seed	:	-0.6
Total read count	:	263
Mature read count	:	256
Loop read count	:	7
Star read count	:	0

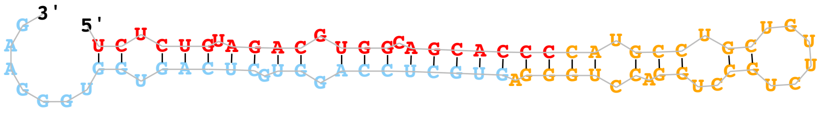

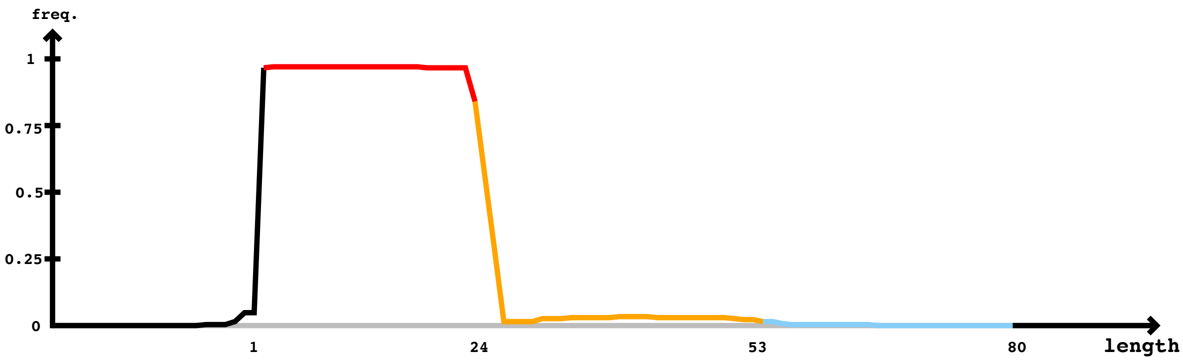

Mature Star

```
5'- augucucacccuguugcucuucucuguagacguggcagcaccccaugccugcuguucugccuggaccugggagugcuccaggugcucaguggugggaagggcuuggcugguga -3'   exp
    .....(((((..((.(((((((((((.((((.(((.(((((((((.(((.((......))..)).).)))).)))))))).)).))))).....))))))))...)).)))))   reads  mm  sample
    ..............ugcucCucucuguagacguggca............................................................................   1      1   seq
    .................ucCucucuguagacguggcagcacc.......................................................................   2      1   seq
    .................ucCucucuguagacguggcagcaccc......................................................................   1      1   seq
    ..................cCucucuguagacguggcagcacc.......................................................................   3      1   seq
    ..................cuucucugCagacguggcagcacc.......................................................................   1      1   seq
    ..................cCucucuguagacguggcagcaccc......................................................................   2      1   seq
    ..................cCucucuguagacguggcagcacccc.....................................................................   2      1   seq
    ..................cCucucuguagacguggcagcacccca....................................................................   1      1   seq
    ....................ucucugCagacguggcagcacc.......................................................................   28     1   seq
    ....................ucucugCagacguggcagcaccc......................................................................   80     1   seq
    ....................ucucuguagacguggcagcaccc......................................................................   5      0   seq
    ....................ucucuguagacguggcagcacccc.....................................................................   11     0   seq
    ....................ucucugCagacguggcagcacccc.....................................................................   46     1   seq
    ....................ucucugCagacguggcagcacccca....................................................................   51     1   seq
    ....................ucucuguagacguggcagcacccca....................................................................   25     0   seq
    ....................ucucuguagacguggcagcaccccaugccugcuguucugcc....................................................   1      0   seq
    .....................cucuguagacguggcagcacccca....................................................................   1      0   seq
    .............................................ugccugcuguucugccuggaccug............................................   1      0   seq
    .............................................ugccugcuguucugccuggaccugg...........................................   1      0   seq
    .............................................ugccugcuguucugccuggaccugggagu.......................................   1      0   seq
    .................................................ugcuguucugccuggaccuggga.........................................   2      0   seq
    .................................................ugcuguucugccuggaccugggagu.......................................   1      0   seq
    ....................................................uguucugccuggaccugggagug......................................   1      0   seq
    .........................................................ugccuggaccugggagugcuccaggug.............................   1      0   seq
```

Provisional ID	:	mun-miR-458a
Score total	:	5.5
Score for star read(s)	:	-1.3
Score for read counts	:	0
Score for mfe	:	2.2
Score for randfold	:	1.6
Score for cons. seed	:	3
Total read count	:	753
Mature read count	:	753
Loop read count	:	0
Star read count	:	0

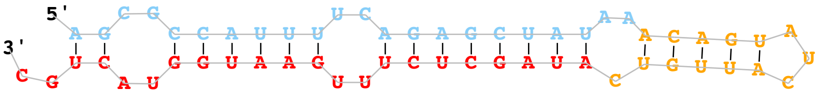

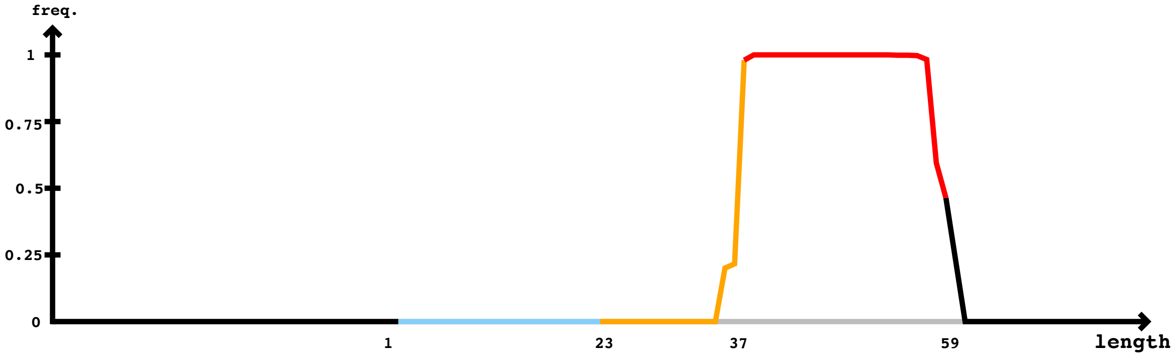

Star Mature

```
5'- gggacgagagccgcugcaccauggugcagauggcagcgccauuuucagagcuauaaacaguaucauugucauagcucuuugaaugguacugccauauguaccggaaaaccag  -3'   exp
    .((.(....))).(((.....(((((((.(((((((..((((((.((((((((..(((((...))))).))))))))..))))))..))))))).)))))))......)))    reads   mm   sample
    ....................................................................ucauagcucuuugaaugg..........................    1       0    seq
    ....................................................................ucauagcucuuugaaugguac.......................    7       0    seq
    ....................................................................ucauagcucuuugaaugguacu......................    118     0    seq
    ....................................................................ucauagcucuuugaaugguacA......................    1       1    seq
    ....................................................................ucauagcucCuugaaugguacu......................    1       1    seq
    ....................................................................Acauagcucuuugaaugguacu......................    1       1    seq
    ....................................................................Ccauagcucuuugaaugguacu......................    1       1    seq
    ....................................................................ucauGgcucuuugaaugguacu......................    1       1    seq
    ....................................................................uUauagcucuuugaaugguacu......................    1       1    seq
    ....................................................................ucauagcucuuugaaugguaUu......................    1       1    seq
    ....................................................................ucauagcucuuugaaugguacug.....................    15      0    seq
    ....................................................................ucauagcucuuugaaugguacuU.....................    1       1    seq
    ....................................................................ucauagcucuuugaaugguacugU....................    2       1    seq
    .....................................................................cauagcucuuugaaugguacu......................    8       0    seq
    .....................................................................cauagcucuuugaaugguacug.....................    4       0    seq
    ......................................................................auagcucuuugaauggua........................    1       0    seq
    ......................................................................auagcucuuugaaugguac.......................    4       0    seq
    ......................................................................auagcucuuugaaugguGcu......................    1       1    seq
    ......................................................................auGgcucuuugaaugguacu......................    1       1    seq
    ......................................................................auagcucuuugaaugguacG......................    1       1    seq
    ......................................................................auagcucuuugaaugguacu......................    156     0    seq
    ......................................................................auagcuUuuugaaugguacug.....................    1       1    seq
    ......................................................................auagcucuuugaaugguacug.....................    77      0    seq
    ......................................................................auagcucuuugaaugguacugc....................    266     0    seq
    ......................................................................auagcucuuugaaugguacugU....................    23      1    seq
    ......................................................................auagcucuuugaaugguacugA....................    1       1    seq
    ......................................................................auagcucuuugaaugguacugcU...................    38      1    seq
    ......................................................................auagcucuuugaaugguacugcA...................    5       1    seq
    .......................................................................uagcucuuugaaugguacug.....................    1       0    seq
    .......................................................................uagcucuuugaaugguacugc....................    8       0    seq
    .......................................................................uagcucuuugaaugguacugcc...................    2       0    seq
    .......................................................................uagcucuuugaaugguacugcU...................    4       1    seq
```

Provisional ID	:	mun-miR-19b
Score total	:	3380.8
Score for star read(s)	:	3.9
Score for read counts	:	3370.6
Score for mfe	:	1.7
Score for randfold	:	1.6
Score for cons. seed	:	3
Total read count	:	6623
Mature read count	:	6613
Loop read count	:	0
Star read count	:	10

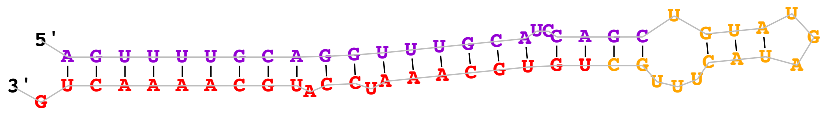

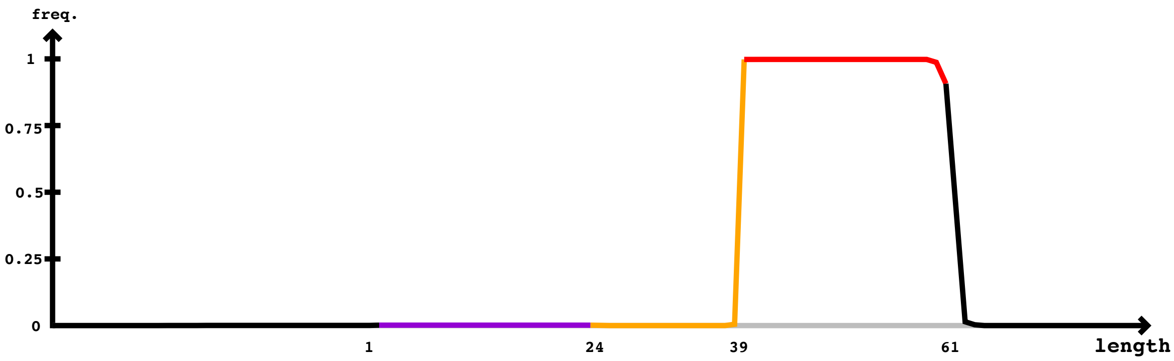


```
                                Star                                 Mature
5'- uuuauuauuuaauugaacacuguucucugguuaguuuugcagguuugcauccagcuguaugauacuuugcugugcaaauccaugcaaaacugacuguggcagugacaaguca   -3'   obs
    uuuauuauuuaauugaacacuguucucugguuaguuuugcagguuugcauccagcuguaugauacuuugcugugcaaauccaugcaaaacugacuguggcagugacaaguca         exp
    .................((((((...(((((((((((((((((((((((..((((.(((...)))...)))))))))).)).)))))))))))))).))))))).........   reads   mm   sample
    ..........aauugaacacuguucucugguu................................................................................   1       0    seq
    ..............gaacacuguucucugguu................................................................................   2       0    seq
    ................................aguuuugcagguuugcauccag..........................................................   1       0    seq
    ................................aguuuugcagguuugcauccagc.........................................................   7       0    seq
    ................................aguuuugcagguuugcauccagU.........................................................   1       1    seq
    ................................aguuuugcagguuugcauccagcu........................................................   1       0    seq
    .....................................................................cugugcaaauccaugcaaaacu.....................   14      0    seq
    .....................................................................cugugcaaauccaugcaaaacug....................   10      0    seq
    .....................................................................cugugcaaauccaugcaaaacuga...................   2       0    seq
    .....................................................................cugugcaaauccaugcaaaacugU...................   5       1    seq
    .....................................................................cugugcaaauccaugcaaaacugaU..................   1       1    seq
    ......................................................................ugugcaaauccaugcaaaa.......................   1       0    seq
    ......................................................................uguAcaaauccaugcaaaac......................   1       1    seq
    ......................................................................ugugcaaauccaugcaaaac......................   70      0    seq
    ......................................................................ugCgcaaauccaugcaaaac......................   1       1    seq
    ......................................................................ugugcaaauccaugcaaaacu.....................   476     0    seq
    ......................................................................Agugcaaauccaugcaaaacu.....................   1       1    seq
    ......................................................................ugCgcaaauccaugcaaaacu.....................   1       1    seq
    ......................................................................Ggugcaaauccaugcaaaacu.....................   3       1    seq
    ......................................................................ugugcaaauccaugUaaaacu.....................   31      1    seq
    ......................................................................ugugcaaauccauUcaaaacu.....................   1       1    seq
    ......................................................................ugugcaaauccauCcaaaacu.....................   1       1    seq
    ......................................................................ugugcaaauccaugcaaaacA.....................   1       1    seq
    ......................................................................uCugcaaauccaugcaaaacu.....................   1       1    seq
    ......................................................................ugugcaaauccaugcaaaacCg....................   1       1    seq
    ......................................................................ugugcaaauccaugcaaaacuU....................   2       1    seq
    ......................................................................ugugcaaaucAaugcaaaacug....................   1       1    seq
    ......................................................................uAugcaaauccaugcaaaacug....................   1       1    seq
    ......................................................................ugugcaaauccaugcaaaGcug....................   1       1    seq
    ......................................................................ugugcaaauccaugcaaaaAug....................   1       1    seq
    ......................................................................ugugcaaauccaugAaaaacug....................   1       1    seq
    ......................................................................Ggugcaaauccaugcaaaacug....................   3       1    seq
    ......................................................................Agugcaaauccaugcaaaacug....................   2       1    seq
```

Star Mature

```
uuuauuauuuaauugaacacuguucucugguuaguuuugcagguuugcauccagcuguaugauacuuugcugugcaaauccaugcaaaacugacuguggcagugacaaguca
......................................................................ugAgcaaauccaugcaaaacug....................  1     1  seq
......................................................................ugugcaaauccauCcaaaacug....................  2     1  seq
......................................................................ugugcaaauccaugcaaaacuA....................  3     1  seq
......................................................................uCugcaaauccaugcaaaacug....................  1     1  seq
......................................................................ugugcaaauccaugUaaaacug....................  20    1  seq
......................................................................ugugcaGauccaugcaaaacug....................  1     1  seq
......................................................................ugugcaaauccaugcaaaacGg....................  7     1  seq
......................................................................ugugcaaauccaugcaaaacug....................  3382  0  seq
......................................................................ugugcaaaCccaugcaaaacug....................  2     1  seq
......................................................................ugugAaaauccaugcaaaacug....................  1     1  seq
......................................................................ugugcUaauccaugcaaaacug....................  1     1  seq
......................................................................uguAcaaauccaugcaaaacug....................  2     1  seq
......................................................................uguUcaaauccaugcaaaacug....................  1     1  seq
......................................................................ugugcaaauccaCgcaaaacug....................  1     1  seq
......................................................................ugugcaaauccaCgcaaaacuga...................  1     1  seq
......................................................................ugugcaaauccaugcaaaacuUa...................  1     1  seq
......................................................................uUugcaaauccaugcaaaacuga...................  2     1  seq
......................................................................ugugcaaauccaugcaaaacugC...................  27    1  seq
......................................................................ugugcaaauccaugcaaaacuga...................  1617  0  seq
......................................................................ugugcaaauccaugcaaaacugU...................  784   1  seq
......................................................................Agugcaaauccaugcaaaacuga...................  1     1  seq
......................................................................ugugcaaauccaugAaaaacuga...................  1     1  seq
......................................................................ugugcaaauccauCcaaaacuga...................  2     1  seq
......................................................................ugugcaaauccauUcaaaacuga...................  1     1  seq
......................................................................ugugcaaauccaugUaaaacuga...................  1     1  seq
......................................................................ugugcGaauccaugcaaaacuga...................  1     1  seq
......................................................................ugugcaaGuccaugcaaaacuga...................  1     1  seq
......................................................................ugugcaaauccaugcaaaacugG...................  14    1  seq
......................................................................Ggugcaaauccaugcaaaacuga...................  5     1  seq
......................................................................ugugcaaaCccaugcaaaacuga...................  1     1  seq
......................................................................ugugcaaaucUaugcaaaacugac..................  7     1  seq
......................................................................ugugcaaauccaugcaaaacugac..................  19    0  seq
......................................................................ugugcaaauccaugcaaaacugaA..................  7     1  seq
......................................................................ugugcaaauccaugcaaaacugaU..................  40    1  seq
......................................................................ugugcaaauccaugcaaaacugacu.................  8     0  seq
......................................................................ugugcaaauccaugcaaaacugaAu.................  1     1  seq
......................................................................ugugcaaauccaugcaaaacugaUu.................  2     1  seq
......................................................................ugugcaaaucUaugcaaaacugacu.................  9     1  seq
........................................................................ugUaaauccaugcaaaacug....................  1     1  seq
```

Provisional ID : mun-miR-101-1
Score total : 4.9
Score for star read(s) : -1.3
Score for read counts : 0
Score for mfe : 1.6
Score for randfold : 1.6
Score for cons. seed : 3
Total read count : 49193
Mature read count : 49186
Loop read count : 0
Star read count : 7

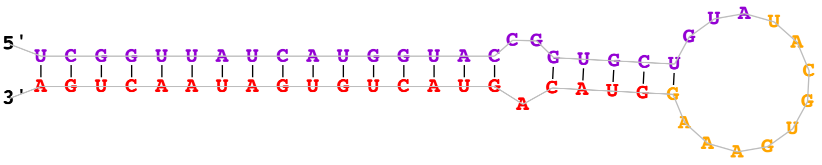

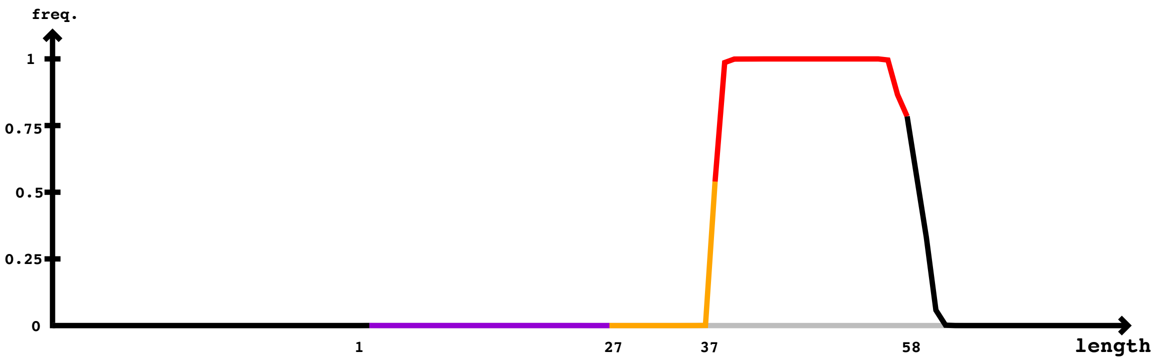

Star Mature

```
5'- augaaauaauaagacugugaacuguccuuuuucgguuaucaugguaccggugcuguauacgugaaagguacaguacugugauaacugaagaaugguggugccaucacuug   -3'    obs
    augaaauaauaagacugugaacuguccuuuuucgguuaucaugguaccggugcuguauacgugaaagguacaguacugugauaacugaagaaugguggugccaucacuug          exp
    ................(((((..((.(((((((((((((((((((((..(((((............))))).))))))))))))))))))))).))..))....))))...    reads    mm    sample
    augaaauaauaagacugugaacuguccuuuu...............................................................................    1    0    seq
    .............acugugaacuguccuuuuu..............................................................................    1    0    seq
    ...............................ucgguuaucaugguaccggug..........................................................    1    0    seq
    ...............................ucgguuaucaugguaccggugc.........................................................    1    0    seq
    ...............................ucgguuaucaugguaccggugA.........................................................    1    1    seq
    ...............................ucgguuaucaugguaccggugcu........................................................    2    0    seq
    ...............................ucgguuaucaugguaccggugcugua.....................................................    2    0    seq
    ...............................................................aaagguacaguacugugauaacu........................    1    0    seq
    ................................................................aagguacaguacugugauaacu........................    5    0    seq
    .................................................................agguacaguacugugauaacu........................    2    0    seq
    ..................................................................Uguacaguacugugauaacu........................    1    1    seq
    ..................................................................gguacaguacugugauaacu........................    5    0    seq
    ..................................................................Cguacaguacugugauaacu........................    2    1    seq
    ..................................................................gguacaguacugugauaacug.......................    5    0    seq
    ..................................................................Cguacaguacugugauaacuga......................    3    1    seq
    ..................................................................gguacaguacugugauaacuga......................    3    0    seq
    ...................................................................guacaguacugugauaac.........................    205    0    seq
    ...................................................................guacagAacugugauaacu........................    3    1    seq
    ...................................................................guacaguacugugauaaUu........................    1    1    seq
    ...................................................................guacaguacugAgauaacu........................    1    1    seq
    ...................................................................guacaguacAgugauaacu........................    1    1    seq
    ...................................................................guacGguacugugauaacu........................    2    1    seq
    ...................................................................guacaguacuCugauaacu........................    2    1    seq
    ...................................................................guacaguacCgugauaacu........................    1    1    seq
    ...................................................................gGacaguacugugauaacu........................    1    1    seq
    ...................................................................guaUaguacugugauaacu........................    1    1    seq
    ...................................................................guacaguCcugugauaacu........................    1    1    seq
    ...................................................................guacaguacugugauaGcu........................    1    1    seq
    ...................................................................guacaguacugugauaacG........................    11    1    seq
    ...................................................................guacaguacGgugauaacu........................    1    1    seq
    ...................................................................guacaUuacugugauaacu........................    3    1    seq
    ...................................................................guacaguacugCgauaacu........................    1    1    seq
    ...................................................................Auacaguacugugauaacu........................    3    1    seq
```

```
                               Star                                  Mature
augaaauaauaagacugugaacuguccuuuuucgguuaucaugguaccggugcuguauacgugaaagguacaguacugugauaacugaagaaugguggugccaucacuug
...................................................................Cuacaguacugugauaacu........................   2      1   seq
...................................................................guacaguacugugauaacu........................   6153   0   seq
...................................................................guacaguacuUugauaacu........................   1      1   seq
...................................................................guaAaguacugugauaacu........................   4      1   seq
...................................................................guacaguacuguAauaacu........................   1      1   seq
...................................................................guacagGacugugauaacu........................   1      1   seq
...................................................................guacaguacugugauaacC........................   1      1   seq
...................................................................Uuacaguacugugauaacu........................   2      1   seq
...................................................................guacaguacuguCauaacu........................   2      1   seq
...................................................................guacaguacugugauaacA........................   2      1   seq
...................................................................gCacaguacugugauaacu........................   3      1   seq
...................................................................guacaguacuUugauaacug.......................   1      1   seq
...................................................................guUcaguacugugauaacug.......................   1      1   seq
...................................................................guacaUuacugugauaacug.......................   2      1   seq
...................................................................Cuacaguacugugauaacug.......................   3      1   seq
...................................................................gCacaguacugugauaacug.......................   1      1   seq
...................................................................Uuacaguacugugauaacug.......................   2      1   seq
...................................................................guacaguacugugauaacuA.......................   9      1   seq
...................................................................guacagGacugugauaacug.......................   1      1   seq
...................................................................guacaguacugAgauaacug.......................   2      1   seq
...................................................................guacaguaGugugauaacug.......................   1      1   seq
...................................................................Auacaguacugugauaacug.......................   4      1   seq
...................................................................guacaguacuguCauaacug.......................   1      1   seq
...................................................................guacaguacugugauaacug.......................   3488   0   seq
...................................................................guacaguacugugauaaAug.......................   1      1   seq
...................................................................guacaguacugugauaacGg.......................   9      1   seq
...................................................................guacaguacugGgauaacug.......................   1      1   seq
...................................................................guacaguacugugGuaacug.......................   1      1   seq
...................................................................guacaguacAgugauaacug.......................   1      1   seq
...................................................................guacaguacugugauaacuU.......................   5      1   seq
...................................................................guacaguaUugugauaacug.......................   3      1   seq
...................................................................guacaguacugGgauaacuga......................   2      1   seq
...................................................................Cuacaguacugugauaacuga......................   2      1   seq
...................................................................guacaguacuUugauaacuga......................   2      1   seq
...................................................................guacaguaGugugauaacuga......................   1      1   seq
...................................................................guacaUuacugugauaacuga......................   2      1   seq
...................................................................guacaguacuAugauaacuga......................   1      1   seq
...................................................................guacaguacuguCauaacuga......................   2      1   seq
...................................................................guacaguacugugauaacugG......................   75     1   seq
...................................................................guacaguacugugGuaacuga......................   1      1   seq
...................................................................guacaguacugugauaaUuga......................   1      1   seq
...................................................................guacaguaAugugauaacuga......................   2      1   seq
...................................................................guacaguacugugauaGcuga......................   3      1   seq
...................................................................guacaguacuguAauaacuga......................   1      1   seq
...................................................................guacaguaUugugauaacuga......................   4      1   seq
...................................................................guacaguacugugauaUcuga......................   2      1   seq
...................................................................guacaguacugugauaacugU......................   581    1   seq
...................................................................guacaguacugugauaacuUa......................   1      1   seq
...................................................................Auacaguacugugauaacuga......................   5      1   seq
...................................................................guacaguacugCgauaacuga......................   1      1   seq
...................................................................gCacaguacugugauaacuga......................   1      1   seq
...................................................................guacaguacugAgauaacuga......................   4      1   seq
...................................................................guacagGacugugauaacuga......................   1      1   seq
...................................................................guacaguacugugauaacCga......................   1      1   seq
...................................................................guacaAuacugugauaacuga......................   1      1   seq
...................................................................guacagAacugugauaacuga......................   2      1   seq
...................................................................guacaguacugugaCaacuga......................   2      1   seq
...................................................................guacaguacugugauaacugC......................   5      1   seq
...................................................................guacaguacugugauaacuga......................   9581   0   seq
...................................................................guacaguacugugauaaAuga......................   2      1   seq
...................................................................guacaguacAgugauaacuga......................   2      1   seq
...................................................................guacaguacugugauaacAga......................   1      1   seq
...................................................................guacagCacugugauaacuga......................   5      1   seq
...................................................................Uuacaguacugugauaacuga......................   1      1   seq
...................................................................guacaguacCgugauaacuga......................   3      1   seq
...................................................................guacaguacugugUuaacuga......................   1      1   seq
...................................................................guacaguacugugauaacugaa.....................   3707   0   seq
...................................................................guacaguacugugauaacugaU.....................   444    1   seq
...................................................................guacaguacugGgauaacugaa.....................   1      1   seq
...................................................................guacaguacugugGuaacugaa.....................   1      1   seq
```

```
                                 Star                                Mature
augaaauaauaagacugugaacuguccuuuuucgguuaucaugguaccggugcuguauacgugaaagguacaguacugugauaacugaagaaugguggugccaucacuug
...................................................................Cuacaguacugugauaacugaa.....................	1	1	seq
...................................................................guacaAuacugugauaacugaa.....................	1	1	seq
...................................................................guacaguacugugauaacugaG.....................	1	1	seq
...................................................................guacaguacugugauaacugaC.....................	820	1	seq
...................................................................guacagCacugugauaacugaa.....................	1	1	seq
...................................................................guacaguGcugugauaacugaa.....................	1	1	seq
...................................................................guacaguacuguUauaacugaa.....................	1	1	seq
...................................................................guaAaguacugugauaacugaa.....................	2	1	seq
...................................................................guacaguacugugauaGcugaa.....................	1	1	seq
...................................................................guacaguacugugauaaUugaa.....................	1	1	seq
...................................................................guacagGacugugauaacugaa.....................	1	1	seq
...................................................................guGcaguacugugauaacugaa.....................	1	1	seq
...................................................................guacaguacugugaCaacugaa.....................	1	1	seq
...................................................................guacaUuacugugauaacugaa.....................	1	1	seq
...................................................................guacaguaAugugauaacugaa.....................	1	1	seq
...................................................................guacaguacugugauaacugUa.....................	2	1	seq
...................................................................gCacaguacugugauaacugaa.....................	2	1	seq
...................................................................Auacaguacugugauaacugaa.....................	3	1	seq
...................................................................guacaguacGgugauaacugaa.....................	1	1	seq
...................................................................Uuacaguacugugauaacugaa.....................	3	1	seq
...................................................................guacaguacugugauaacugaag....................	30	0	seq
...................................................................guacaguacugugauaacugaCg....................	1	1	seq
...................................................................guacaguacugugauaacugaaC....................	70	1	seq
...................................................................guacaguacugugauaacugaaU....................	293	1	seq
...................................................................guacaguacugugauaacugaaA....................	840	1	seq
...................................................................guacaguacugugauaacugaagU...................	1	1	seq
...................................................................guacaguacugugauaacugaaga...................	1	0	seq
...................................................................guacaguacugugauaacugaaUa...................	11	1	seq
...................................................................guacaguacugugauaacugaaAa...................	40	1	seq
...................................................................guacaguacugugauaacugaagaU..................	1	1	seq
...................................................................guacaguacugugauaacugaaAaa..................	2	1	seq
....................................................................uacaguacugugauaacG........................	1	1	seq
....................................................................uacagCacugugauaacu........................	1	1	seq
....................................................................uacaguacugugauaacu........................	164	0	seq
....................................................................uacaguacugugauaacug.......................	461	0	seq
....................................................................uacagCacugugauaacug.......................	1	1	seq
....................................................................uacagAacugugauaacug.......................	1	1	seq
....................................................................uacaguacugugUuaacuga......................	2	1	seq
....................................................................uacaguacugugauaGcuga......................	2	1	seq
....................................................................uacagGacugugauaacuga......................	1	1	seq
....................................................................uacaguacuAugauaacuga......................	1	1	seq
....................................................................uacaguaUugugauaacuga......................	1	1	seq
....................................................................uacaCuacugugauaacuga......................	1	1	seq
....................................................................uacGguacugugauaacuga......................	1	1	seq
....................................................................uacaguacugugauaacGga......................	1	1	seq
....................................................................uacaguacugugauaacuga......................	2504	0	seq
....................................................................Gacaguacugugauaacuga......................	1	1	seq
....................................................................Aacaguacugugauaacuga......................	3	1	seq
....................................................................uacaguacugugauaacugG......................	17	1	seq
....................................................................uacagAacugugauaacuga......................	2	1	seq
....................................................................uaAaguacugugauaacuga......................	1	1	seq
....................................................................uacaUuacugugauaacuga......................	1	1	seq
....................................................................uacaguacuUugauaacuga......................	2	1	seq
....................................................................uacaguacugugGuaacuga......................	1	1	seq
....................................................................uacaguacugugauaacugU......................	107	1	seq
....................................................................Cacaguacugugauaacuga......................	1	1	seq
....................................................................uacaguacugugauaacugC......................	1	1	seq
....................................................................uacaguacugugauaaAuga......................	2	1	seq
....................................................................uacaguacugugauaacuUa......................	1	1	seq
....................................................................uacaguacuguUauaacuga......................	2	1	seq
....................................................................uacaguacuguAauaacuga......................	1	1	seq
....................................................................uacaguacugugauaacugaC.....................	765	1	seq
....................................................................uacaguacugugGuaacugaa.....................	2	1	seq
....................................................................uacaguacugugauaacugaU.....................	167	1	seq
....................................................................uacGguacugugauaacugaa.....................	3	1	seq
....................................................................uacaguacugugauaacCgaa.....................	1	1	seq
....................................................................uacaguacugugauaacuAaa.....................	1	1	seq
....................................................................uacaguacuCugauaacugaa.....................	1	1	seq
....................................................................uacaguaAugugauaacugaa.....................	2	1	seq
....................................................................Cacaguacugugauaacugaa.....................	2	1	seq
```

```
                                  Star                                  Mature
augaaauaauaagacugugaacuguccuuuuucgguuaucaugguaccggugcuguauacgugaaagguacaguacugugauaacugaagaaugguggugccaucacuug
....................................................................uacaguacugugauaaGugaa.....................  1     1  seq
....................................................................uacaguacugugauaacGgaa.....................  1     1  seq
....................................................................uacaguacugugauaacugaa.....................  3295  0  seq
....................................................................uacagGacugugauaacugaa.....................  2     1  seq
....................................................................uacaguacugugauaacugaG.....................  1     1  seq
....................................................................uacaguacugCgauaacugaa.....................  3     1  seq
....................................................................Gacaguacugugauaacugaa.....................  4     1  seq
....................................................................uacaUuacugugauaacugaa.....................  2     1  seq
....................................................................Aacaguacugugauaacugaa.....................  2     1  seq
....................................................................uacaguacugugaCaacugaa.....................  2     1  seq
....................................................................uacaguUcugugauaacugaa.....................  1     1  seq
....................................................................uacaguacugugauaGcugaa.....................  2     1  seq
....................................................................uacagCacugugauaacugaa.....................  1     1  seq
....................................................................uacaguacuAugauaacugaa.....................  1     1  seq
....................................................................uacaguacuUugauaacugaa.....................  1     1  seq
....................................................................uacagAacugugauaacugaa.....................  1     1  seq
....................................................................uacaguacCgugauaacugaa.....................  1     1  seq
....................................................................uacaUuacugugauaacugaag....................  1     1  seq
....................................................................uacaguGcugugauaacugaag....................  1     1  seq
....................................................................uacagCacugugauaacugaag....................  1     1  seq
....................................................................uacaguacugugauaUcugaag....................  1     1  seq
....................................................................uacagGacugugauaacugaag....................  2     1  seq
....................................................................uGcaguacugugauaacugaag....................  1     1  seq
....................................................................uacaCuacugugauaacugaag....................  1     1  seq
....................................................................uacagAacugugauaacugaag....................  1     1  seq
....................................................................uacaguacugugauaacugaag....................  4132  0  seq
....................................................................uacaguaAugugauaacugaag....................  1     1  seq
....................................................................uacaguacugugauaacugaGg....................  1     1  seq
....................................................................uacaguacugugauaacuCaag....................  1     1  seq
....................................................................uacaguacugugauaaUugaag....................  1     1  seq
....................................................................uacGguacugugauaacugaag....................  1     1  seq
....................................................................Gacaguacugugauaacugaag....................  2     1  seq
....................................................................uacaguacugugauaacugaaU....................  4229  1  seq
....................................................................uacaguacugugauaacugaaA....................  1974  1  seq
....................................................................uacaguacugugauaacugaCg....................  10    1  seq
....................................................................Aacaguacugugauaacugaag....................  3     1  seq
....................................................................Cacaguacugugauaacugaag....................  4     1  seq
....................................................................uacaguacugugauaacugaaC....................  1650  1  seq
....................................................................uacaguacuUugauaacugaag....................  1     1  seq
....................................................................uacaguacugugauaacugaaga...................  507   0  seq
....................................................................uacaguacugugauaGcugaaga...................  1     1  seq
....................................................................uacaguacugugauaacugaagC...................  35    1  seq
....................................................................uacaguacugugauaacugaaUa...................  12    1  seq
....................................................................uacaguacGgugauaacugaaga...................  1     1  seq
....................................................................uacaguacugugauaacugaaCa...................  3     1  seq
....................................................................uacaguacugugauaacugaaAa...................  73    1  seq
....................................................................uacUguacugugauaacugaaga...................  1     1  seq
....................................................................uacaguacugugauaacugaagU...................  1710  1  seq
....................................................................uacaguacugugauaacugaagaa..................  22    0  seq
....................................................................uacaguacugugauaacugaagaU..................  4     1  seq
....................................................................uacaguacugugauaacugaagUa..................  11    1  seq
....................................................................uacaguacugugauaacugaaAaa..................  3     1  seq
....................................................................uacaguacugugauaacugaagUau.................  1     1  seq
.....................................................................acaguacugugauaacuga......................  13    0  seq
.....................................................................acaguacugugauaacugU......................  1     1  seq
.....................................................................acaguacugugauaacugaC.....................  2     1  seq
.....................................................................acagAacugugauaacugaa.....................  1     1  seq
.....................................................................acaguacugugauaacugaa.....................  36    0  seq
.....................................................................acaguacugugauaacugaag....................  131   0  seq
.....................................................................acaguacugugauaacugaaC....................  4     1  seq
.....................................................................acaguacugugauaacugaaU....................  42    1  seq
.....................................................................acaguacugugauaaUugaag....................  1     1  seq
.....................................................................acaguacugugauaacugaaA....................  26    1  seq
.....................................................................acaguacugugauaacugaagC...................  3     1  seq
.....................................................................acaguacugugauaacugaagU...................  359   1  seq
.....................................................................acaguacugugauaacugaaga...................  3     0  seq
.....................................................................acaguacugugauaacugaagUa..................  20    1  seq
.....................................................................acaguacugugauaacugaagUau.................  5     1  seq
.....................................................................acaguacugugauaacugaagaaG................  1     1  seq
......................................................................caguacugugauaacugaa.....................  1     0  seq
```

Star **Mature**

```
augaaauaauaagacugugaacuguccuuuuucgguuaucaugguaccggugcuguauacgugaaagguacaguacugugauaacugaagaaugguggugccaucacuug
......................................................................caguacugugauaacugaaU....................	1	1	seq
......................................................................caguacugugauaacugaaA....................	2	1	seq
......................................................................caguacugugauaacugaag....................	1	0	seq
......................................................................caguacugugauaacugaagU...................	3	1	seq
......................................................................caguacugugauaacugaaAaa..................	1	1	seq
.......................................................................aguacugugauaacugaa.....................	1	0	seq
.......................................................................aguacugugauaacugaagU...................	1	1	seq
........................................................................guacugugauaacugaaU....................	4	1	seq
........................................................................guacugugauaacugaaA....................	1	1	seq
........................................................................guacugugauaacugaag....................	3	0	seq
........................................................................guacugugauaacugaagU...................	4	1	seq
........................................................................guacugugauaacugaaga...................	1	0	seq
```

Provisional ID : mun-miR-215
Score total : 35512.5
Score for star read(s) : 3.9
Score for read counts : 35503.3
Score for mfe : 0.7
Score for randfold : 1.6
Score for cons. seed : 3
Total read count : 69650
Mature read count : 69646
Loop read count : 0
Star read count : 4

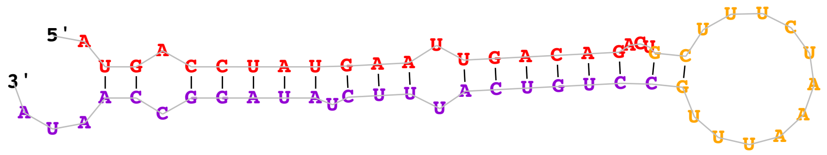

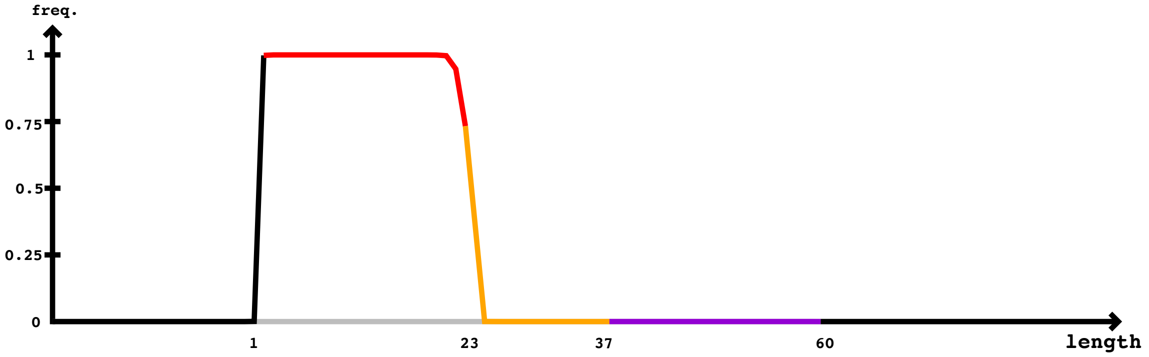

Mature **Star**

```
5'- aagaacugguguccaggaaaaugaccuaugaauugacagacugcuuucuaaaauuugccugucauuucuauaggccaauauucugugcacuuucccacucuggucacag  -3'   obs
    aagaacugguguccaggaaaaugaccuaugaauugacagacugcuuucuaaaauuugccugucauuucuauaggccaauauucugugcacuuucccacucuggucacag         exp
    ..(((..((((..(((((.(.((.((((((((.((((((...((...........))))))))).))).))))).)).).))))).)))).))).....(((....)))  reads  mm  sample
    ..................aaaugaccuaugaauugacagacC...................................................................  2      1   seq
    ...................Caugaccuaugaauugacagac....................................................................  4      1   seq
    ...................aaugaccuaugaauugacagac....................................................................  6      0   seq
    ...................aaugaccuaugaauugacagacu...................................................................  1      0   seq
    ...................Caugaccuaugaauugacagacu...................................................................  23     1   seq
    ...................aaugaccuaugaauugacagacC...................................................................  5      1   seq
    ...................aaugaccuaugaauugacagacA...................................................................  1      1   seq
    ....................augaccuaugaauugaca.......................................................................  28     0   seq
    ....................augaccuaugaauugacaA......................................................................  2      1   seq
    ....................augaccuaugaauuUacag......................................................................  1      1   seq
    ....................augaccuaugaauugacag......................................................................  179    0   seq
    ....................augaAcuaugaauugacaga.....................................................................  1      1   seq
    ....................augaccuaugaaAugacaga.....................................................................  1      1   seq
    ....................augaccuaugaauCgacaga.....................................................................  1      1   seq
    ....................augaccuaugaauugacaga.....................................................................  3454   0   seq
    ....................augaccuaugaauugacGga.....................................................................  1      1   seq
    ....................Uugaccuaugaauugacaga.....................................................................  2      1   seq
    ....................augaccuaugaauugacaAa.....................................................................  2      1   seq
    ....................augacAuaugaauugacaga.....................................................................  1      1   seq
    ....................augaccuaugaauugacagU.....................................................................  5      1   seq
    ....................Gugaccuaugaauugacaga.....................................................................  1      1   seq
    ....................augaccuaAgaauugacaga.....................................................................  1      1   seq
    ....................augaccuaugaauugCcaga.....................................................................  1      1   seq
    ....................augaccuaugaauuCacaga.....................................................................  1      1   seq
    ....................augaccAaugaauugacaga.....................................................................  1      1   seq
    ....................augaccuaugGauugacaga.....................................................................  2      1   seq
    ....................augaccuaugaauugGcaga.....................................................................  2      1   seq
    ....................augaGcuaugaauugacaga.....................................................................  1      1   seq
    ....................augaccuaugUauugacagac....................................................................  1      1   seq
    ....................augaccuauAaauugacagac....................................................................  3      1   seq
    ....................augaccuaugaauugacaAac....................................................................  1      1   seq
    ....................augaccuaugaauuAacagac....................................................................  3      1   seq
    ....................augaccuaugaUuugacagac....................................................................  1      1   seq
```

Mature Star

```
aagaacugguguccaggaaaaugaccuaugaauugacagacugcuuucuaaauuugccugucauuucuauaggccaauauucugugcacuuuucccacucuggucacag
....................augaAcuaugaauugacagac....................................................................   1       1   seq
....................augaccuaugaauugacagCc....................................................................   3       1   seq
....................augCccuaugaauugacagac....................................................................   1       1   seq
....................Uugaccuaugaauugacagac....................................................................   3       1   seq
....................augaccuaugaauugacagaA....................................................................   117     1   seq
....................Gugaccuaugaauugacagac....................................................................   11      1   seq
....................augaccuaugaauugacGgac....................................................................   1       1   seq
....................augGccuaugaauugacagac....................................................................   1       1   seq
....................augaccuaugaauuCacagac....................................................................   2       1   seq
....................Cugaccuaugaauugacagac....................................................................   4       1   seq
....................augaccuaugaauugacagGc....................................................................   3       1   seq
....................augaccuaugaauugacagaU....................................................................   5       1   seq
....................augaccuaAgaauugacagac....................................................................   1       1   seq
....................augaccuauUaauugacagac....................................................................   2       1   seq
....................aCgaccuaugaauugacagac....................................................................   2       1   seq
....................augaccuaugaauuUacagac....................................................................   1       1   seq
....................augaccuaugaauugaAagac....................................................................   3       1   seq
....................auCaccuaugaauugacagac....................................................................   1       1   seq
....................augaccuaugaauugacaUac....................................................................   1       1   seq
....................augaccuaugaauCgacagac....................................................................   4       1   seq
....................augaccuaCgaauugacagac....................................................................   2       1   seq
....................augaccuaugaauugacagUc....................................................................   1       1   seq
....................augaccuaGgaauugacagac....................................................................   1       1   seq
....................aAgaccuaugaauugacagac....................................................................   1       1   seq
....................augaccuaugaauugaGagac....................................................................   2       1   seq
....................augaccuaugaauugacagac....................................................................   14701   0   seq
....................augacAuaugaauugacagac....................................................................   5       1   seq
....................auCaccuaugaauugacagacu...................................................................   3       1   seq
....................augacUuaugaauugacagacu...................................................................   1       1   seq
....................augaUcuaugaauugacagacu...................................................................   3       1   seq
....................augaccuaugaauugacaUacu...................................................................   3       1   seq
....................augaccuaugaauugacagCcu...................................................................   1       1   seq
....................augaccuaugaauCgacagacu...................................................................   2       1   seq
....................augGccuaugaauugacagacu...................................................................   4       1   seq
....................augaccuauAaauugacagacu...................................................................   4       1   seq
....................augaccuaugaauugaAagacu...................................................................   15      1   seq
....................augaccuaugaauugacagGcu...................................................................   5       1   seq
....................augaccuaugaauugacaAacu...................................................................   5       1   seq
....................augaccuaugaauugacagaGu...................................................................   1       1   seq
....................augaccuaugaauuAacagacu...................................................................   8       1   seq
....................augaccuaugaauugacGgacu...................................................................   8       1   seq
....................Gugaccuaugaauugacagacu...................................................................   40      1   seq
....................augaccuauUaauugacagacu...................................................................   2       1   seq
....................augaccuaugaauAgacagacu...................................................................   3       1   seq
....................augUccuaugaauugacagacu...................................................................   1       1   seq
....................augCccuaugaauugacagacu...................................................................   1       1   seq
....................augaccGaugaauugacagacu...................................................................   1       1   seq
....................augaccuaugaGuugacagacu...................................................................   3       1   seq
....................augaccuaugaauugaUagacu...................................................................   8       1   seq
....................augaccuaugaauugacagacA..................................................................   4652    1   seq
....................augaccuaugaauugacagacu...................................................................   43228   0   seq
....................augaAcuaugaauugacagacu...................................................................   9       1   seq
....................augaccuaugaauugacaCacu...................................................................   2       1   seq
....................auUaccuaugaauugacagacu...................................................................   10      1   seq
....................augaccuaCgaauugacagacu...................................................................   3       1   seq
....................augaccuaugaCuugacagacu...................................................................   3       1   seq
....................augaccuaugaauugacagacC..................................................................   2353    1   seq
....................augaccuaugaauugacUgacu...................................................................   1       1   seq
....................Uugaccuaugaauugacagacu...................................................................   8       1   seq
....................augaccuaugaauugacCgacu...................................................................   1       1   seq
....................auAaccuaugaauugacagacu...................................................................   2       1   seq
....................augaccuGugaauugacagacu...................................................................   3       1   seq
....................augaccuauCaauugacagacu...................................................................   2       1   seq
....................augaccuaugaauugacagaUu...................................................................   5       1   seq
....................augaccuaugaauugacagacG..................................................................   227     1   seq
....................augaccuaugaauuUacagacu...................................................................   13      1   seq
....................augaccuaAgaauugacagacu...................................................................   4       1   seq
....................augaccuaugaauugaGagacu...................................................................   2       1   seq
....................augaGcuaugaauugacagacu...................................................................   3       1   seq
....................augaccuaugaauugGcagacu...................................................................   2       1   seq
```

Mature Star

```
aagaacugguguccaggaaaaugaccuaugaauugacagacugcuuucuaaaauuugccugucauuucuauaggccaauauucugugcacuuuucccacucuggucacag
....................augaccuaugaauugacagaAu....................................................................	8	1	seq
....................augaccuaugaauugUcagacu....................................................................	3	1	seq
....................aAgaccuaugaauugacagacu....................................................................	2	1	seq
....................augaccuaugaaAugacagacu....................................................................	2	1	seq
....................augaccAaugaauugacagacu....................................................................	4	1	seq
....................augaccuaugaaCugacagacu....................................................................	4	1	seq
....................Cugaccuaugaauugacagacu....................................................................	10	1	seq
....................augaccuaugaauugacagUcu....................................................................	1	1	seq
....................augaccuaugaauuCacagacu....................................................................	6	1	seq
....................augaccCaugaauugacagacu....................................................................	8	1	seq
....................augaccuaugCauugacagacu....................................................................	1	1	seq
....................augaccuaugGauugacagacu....................................................................	4	1	seq
....................aCgaccuaugaauugacagacu....................................................................	6	1	seq
....................augaccuaGgaauugacagacu....................................................................	1	1	seq
....................augaccuaugaauugacagacuA...................................................................	19	1	seq
....................augaccuaugaauugacagacuC...................................................................	1	1	seq
....................augaccuaugaauugacagacuU...................................................................	139	1	seq
....................augaccuaugaauugacagacug...................................................................	3	0	seq
....................augaccuaugaauugacagacCg...................................................................	1	1	seq
....................augaccuaugaauugacagacugG..................................................................	1	1	seq
....................augaccuaugaauugacagacugc..................................................................	10	0	seq
....................augaccuaugaauugacagacugU..................................................................	7	1	seq
....................augaccuaugaauugacagacugcu.................................................................	3	0	seq
....................augaccuaugaauugacagacugcCu................................................................	1	1	seq
.....................ugaccuaugaauugacaga......................................................................	2	0	seq
.....................ugaccuaugaauugacagac.....................................................................	16	0	seq
.....................Agaccuaugaauugacagacu....................................................................	1	1	seq
.....................ugaccuaugaauugacagacA....................................................................	5	1	seq
.....................ugaccuaugaauugacagacu....................................................................	77	0	seq
.....................ugaccuaugaauugacagacC....................................................................	23	1	seq
.....................ugaccuaugaauugacagacugc..................................................................	1	0	seq
.......................accuaugaauugacagac.....................................................................	1	0	seq
.......................accuaugaauugacagacu....................................................................	1	0	seq
.........................................................ccugucauuucuauaggccaau...............................	2	0	seq
.........................................................ccugucauuucuauaggccaaua..............................	2	0	seq
```

Provisional ID	:	mun-novel84
Score total	:	5.1
Score for star read(s)	:	-1.3
Score for read counts	:	0
Score for mfe	:	1.8
Score for randfold	:	1.6
Score for cons. seed	:	3
Total read count	:	38
Mature read count	:	36
Loop read count	:	0
Star read count	:	2

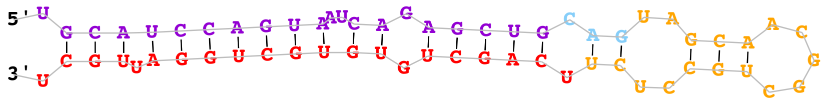

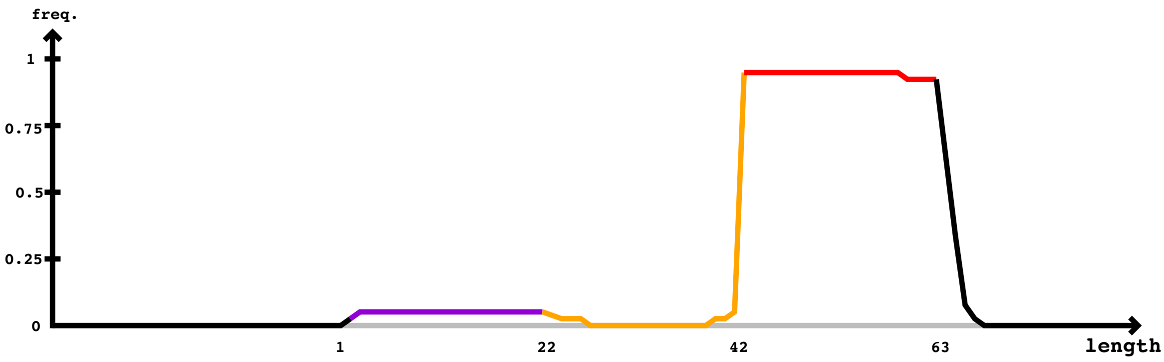

Star **Mature**

```
5'- ugucaagagaagcauugcaucgcuacaucugcauccaguaaucagagcugcaguagcaacggcugccucuucagcugugugcuggauugcugaauguggugaaugaaacac   -3'    obs
    ugucaagagaagcauugcaucgcuacaucugcauccaguaaucagagcugcaguagcaacggcugccucuucagcugugugcuggauugcugaauguggugaaugaaacac          exp
    .................((((((((((((.((((((((((..((.(((((.((..(((.....)))..)).))))).))))))))).))).).)))))))).)))......   reads    mm     sample
    .............................ugcauccaguaaucagagcug.............................................................   1        0      seq
    ..............................gcauccaguaaucagagcugcagu.........................................................   1        0      seq
    ...................................................................ucuucagcugugugcuggau........................   1        0      seq
    .....................................................................uucagcugugugcuggauugcu....................   1        0      seq
    ......................................................................ucagcugugugcuggauugcu....................   16       0      seq
    ......................................................................ucagcugugugcuggauugcuU...................   1        1      seq
    ......................................................................ucagcugugugcuggauugcug...................   5        0      seq
    ......................................................................ucagcugugugcuggauugcugU..................   4        1      seq
    ......................................................................ucagcugugugcuggauugcuga..................   6        0      seq
    ......................................................................ucagcugugugcuggauugcugaU.................   2        1      seq
    ......................................................................ucagcugugugcuggauugcugaUu................   1        1      seq
```

Provisional ID	:	mun-miR-100
Score total	:	79368.8
Score for star read(s)	:	3.9
Score for read counts	:	79359
Score for mfe	:	1.3
Score for randfold	:	1.6
Score for cons. seed	:	3
Total read count	:	155671
Mature read count	:	155589
Loop read count	:	0
Star read count	:	82

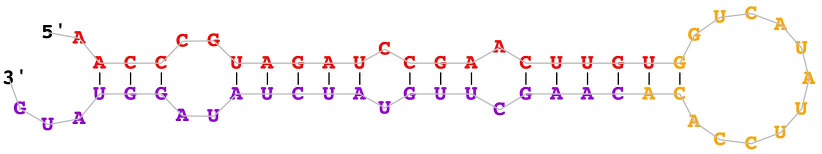

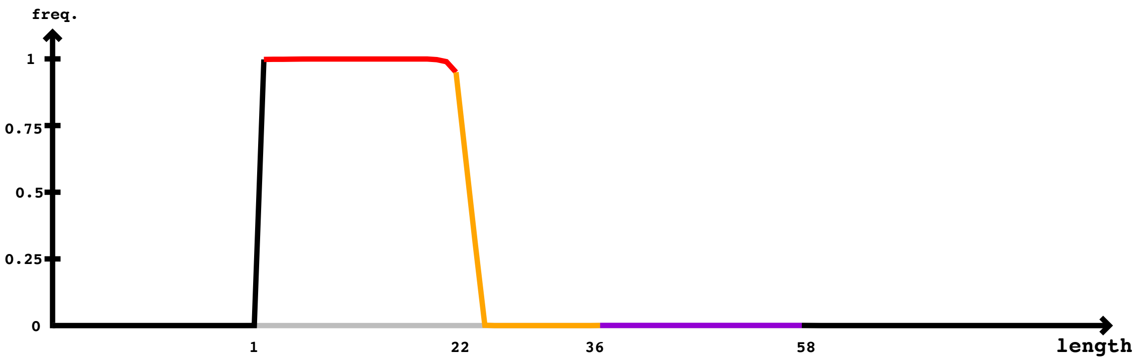

Mature Star

```
5'- gaccccugccuguugccgcaaacccguagauccgaacuuguggucauauuccacacaagcuuguaucuauagguaugugucugucuggcaagugcacaaaggguugug   -3'   obs
    gaccccugccuguugccgcaaacccguagauccgaacuuguggucauauuccacacaagcuuguaucuauagguaugugucugucuggcaagugcacaaaggguugug         exp
    ((((.((((.(..(.((((.(((..(((((.(((.((((((...........)))))).))).)))))..))).)))).)...).)))).(....)...)))))...    reads   mm   sample
    .................Aaaacccguagauccgaacuugu....................................................................    1       1    seq
    .................Aaaacccguagauccgaacuugug...................................................................    1       1    seq
    ..................aaacccguagauccgaacuug.....................................................................    1       0    seq
    ..................Caacccguagauccgaacuug.....................................................................    1       1    seq
    ..................aaacccguagauccgaacuugu....................................................................    2       0    seq
    ..................Caacccguagauccgaacuugu....................................................................    1       1    seq
    ..................Caacccguagauccgaacuugug...................................................................    8       1    seq
    ...................aacccguagauUcgaacu.......................................................................    1       1    seq
    ...................Gacccguagauccgaacu.......................................................................    1       1    seq
    ...................aacccgCagauccgaacu.......................................................................    2       1    seq
    ...................aacccguagauccgaacu.......................................................................    362     0    seq
    ...................aacccguagauccgaacA.......................................................................    1       1    seq
    ...................aacccguagaucGgaacu.......................................................................    1       1    seq
    ...................aacccgAagauccgaacuu......................................................................    1       1    seq
    ...................aacccguagauccgaacuA......................................................................    3       1    seq
    ...................aaUccguagauccgaacuu......................................................................    1       1    seq
    ...................aacccguagaucUgaacuu......................................................................    7       1    seq
    ...................aaAccguagauccgaacuu......................................................................    1       1    seq
    ...................aacccguagauccgaacuG......................................................................    2       1    seq
    ...................aacccguagaucAgaacuu......................................................................    1       1    seq
    ...................aacccguagauccgaaUuu......................................................................    1       1    seq
    ...................Gacccguagauccgaacuu......................................................................    1       1    seq
    ...................aacccguagauccgaaAuu......................................................................    2       1    seq
    ...................aacccguagauccUaacuu......................................................................    1       1    seq
    ...................aacccguagauccgaacuu......................................................................    1142    0    seq
    ...................aacccguagaCccgaacuu......................................................................    1       1    seq
    ...................aacccgGagauccgaacuu......................................................................    2       1    seq
    ...................aacccguagauccgCacuu......................................................................    1       1    seq
    ...................aacccguaUauccgaacuu......................................................................    1       1    seq
    ...................aacccUuagauccgaacuug.....................................................................    1       1    seq
    ...................aacccguagauccgaaUuug.....................................................................    4       1    seq
    ...................aaccUguagauccgaacuug.....................................................................    1       1    seq
    ...................aacccguagauccgaacuuA.....................................................................    9       1    seq
```

```
                    Mature                Star
gaccccugccuguugccgcaaacccguagauccgaacuuguggucauauuccacacaagcuuguaucuauagguaugugucugucuggcaagugcacaaaggguugug
....................aacccgAagauccgaacuug........................................................................................ 1 1 seq
....................aacccguagauUcgaacuug........................................................................................ 6 1 seq
....................aacccguagauccgaacuGg........................................................................................ 2 1 seq
....................aaccAguagauccgaacuug........................................................................................ 2 1 seq
....................aacccguaUauccgaacuug........................................................................................ 1 1 seq
....................aacccguagauccgaacuuU........................................................................................ 18 1 seq
....................aacccgGagauccgaacuug........................................................................................ 1 1 seq
....................Gacccguagauccgaacuug........................................................................................ 6 1 seq
....................aacccguagaAccgaacuug........................................................................................ 1 1 seq
....................aacccgCagauccgaacuug........................................................................................ 2 1 seq
....................aacGcguagauccgaacuug........................................................................................ 1 1 seq
....................aacAcguagauccgaacuug........................................................................................ 2 1 seq
....................Cacccguagauccgaacuug........................................................................................ 2 1 seq
....................aacccguagaucUgaacuug........................................................................................ 13 1 seq
....................aacccguagauccAaacuug........................................................................................ 1 1 seq
....................aacccguagaCccgaacuug........................................................................................ 2 1 seq
....................aacccguaAauccgaacuug........................................................................................ 1 1 seq
....................aacccAuagauccgaacuug........................................................................................ 1 1 seq
....................aacccguagauccgaacuug........................................................................................ 6101 0 seq
....................aacccguagaucAgaacuug........................................................................................ 2 1 seq
....................aacccguagaucAgaacuugu....................................................................................... 7 1 seq
....................aaUccguagauccgaacuugu....................................................................................... 10 1 seq
....................aacccguagauccgaacAugu....................................................................................... 1 1 seq
....................aacccguagauccgaacuuUu....................................................................................... 9 1 seq
....................aacccguagauccAaacuugu....................................................................................... 4 1 seq
....................aacccAuagauccgaacuugu....................................................................................... 5 1 seq
....................aacccguagauccgaGcuugu....................................................................................... 4 1 seq
....................aacccguagauccgGacuugu....................................................................................... 13 1 seq
....................aacccgCagauccgaacuugu....................................................................................... 11 1 seq
....................aacccguagauGcgaacuugu....................................................................................... 2 1 seq
....................aacccguagaAccgaacuugu....................................................................................... 2 1 seq
....................aacccguagauccgaaUuugu....................................................................................... 20 1 seq
....................aacccguaAauccgaacuugu....................................................................................... 2 1 seq
....................aacccguagauccgaCcuugu....................................................................................... 1 1 seq
....................aacccguagaCccgaacuugu....................................................................................... 6 1 seq
....................aGcccguagauccgaacuugu....................................................................................... 2 1 seq
....................aacccguagauccgaacuugG....................................................................................... 80 1 seq
....................aacccguagaucGgaacuugu....................................................................................... 3 1 seq
....................aacccguUgauccgaacuugu....................................................................................... 6 1 seq
....................aacccguagauccgUacuugu....................................................................................... 1 1 seq
....................aacccguaUauccgaacuugu....................................................................................... 9 1 seq
....................aaAccguagauccgaacuugu....................................................................................... 7 1 seq
....................aacccguagauccgaaAuugu....................................................................................... 11 1 seq
....................aacccguagauccgaacuCgu....................................................................................... 3 1 seq
....................aacccguagaGccgaacuugu....................................................................................... 1 1 seq
....................aaccAguagauccgaacuugu....................................................................................... 9 1 seq
....................aacccguagauccUaacuugu....................................................................................... 4 1 seq
....................aacccguagauccgaacuugC....................................................................................... 7 1 seq
....................aaccUguagauccgaacuugu....................................................................................... 15 1 seq
....................aacccguagauccgCacuugu....................................................................................... 5 1 seq
....................aUcccguagauccgaacuugu....................................................................................... 1 1 seq
....................aacccguagaucUgaacuugu....................................................................................... 260 1 seq
....................aaGccguagauccgaacuugu....................................................................................... 1 1 seq
....................aacccguaCauccgaacuugu....................................................................................... 3 1 seq
....................aacccguagGuccgaacuugu....................................................................................... 4 1 seq
....................Cacccguagauccgaacuugu....................................................................................... 9 1 seq
....................aacccguagauccgaaGuugu....................................................................................... 2 1 seq
....................aaccGguagauccgaacuugu....................................................................................... 4 1 seq
....................aacAcguagauccgaacuugu....................................................................................... 1 1 seq
....................aacccgGagauccgaacuugu....................................................................................... 21 1 seq
....................aacccguagauccgaacuuAu....................................................................................... 2 1 seq
....................aacccguagauccgaacuuCu....................................................................................... 2 1 seq
....................aacUcguagauccgaacuugu....................................................................................... 4 1 seq
....................Gacccguagauccgaacuugu....................................................................................... 44 1 seq
....................aacccguagauUcgaacuugu....................................................................................... 29 1 seq
....................aacccguagauccgaacuugA....................................................................................... 240 1 seq
....................aacccCuagauccgaacuugu....................................................................................... 2 1 seq
....................aacccguagauAcgaacuugu....................................................................................... 4 1 seq
....................aacccguagauccgaacuAgu....................................................................................... 3 1 seq
....................aacccguagauccgaacuugu....................................................................................... 49275 0 seq
```

Mature Star

```
gaccccugccuguugccgcaaacccguagauccgaacuuguggucauauuccacacacaagcuuguaucuauagguaugugucugucuggcaagugcacaaaggguugug
....................aacccgAagauccgaacuugu.....................................................................	6	1	seq
....................aacccguGgauccgaacuugu.....................................................................	1	1	seq
....................aacccguagUuccgaacuugu.....................................................................	3	1	seq
....................aacccguagauccCaacuugu.....................................................................	4	1	seq
....................Uacccguagauccgaacuugu.....................................................................	9	1	seq
....................aacccUuagauccgaacuugu.....................................................................	16	1	seq
....................aacccguagauccgCacuugug....................................................................	2	1	seq
....................aacccguagUuccgaacuugug....................................................................	5	1	seq
....................aacccgCagauccgaacuugug....................................................................	9	1	seq
....................aacccguagauccgaacuCgug....................................................................	5	1	seq
....................aacccguagauccgaacuugGg....................................................................	33	1	seq
....................Uacccguagauccgaacuugug....................................................................	15	1	seq
....................aacccguagauAcgaacuugug....................................................................	5	1	seq
....................aacccguagauccAaacuugug....................................................................	3	1	seq
....................aacccguagCuccgaacuugug....................................................................	3	1	seq
....................aacccguaUauccgaacuugug....................................................................	7	1	seq
....................aacccguGgauccgaacuugug....................................................................	6	1	seq
....................aacccguagauccgaCcuugug....................................................................	2	1	seq
....................aacccguagauccgaaAuugug....................................................................	36	1	seq
....................aaGccguagauccgaacuugug....................................................................	2	1	seq
....................aaccUguagauccgaacuugug....................................................................	4	1	seq
....................aacccguagauUcgaacuugug....................................................................	12	1	seq
....................aaAccguagauccgaacuugug....................................................................	8	1	seq
....................aacAcguagauccgaacuugug....................................................................	12	1	seq
....................aacccguaCauccgaacuugug....................................................................	1	1	seq
....................aacccguagauccgaacuugug....................................................................	48525	0	seq
....................aacGcguagauccgaacuugug....................................................................	1	1	seq
....................aaUccguagauccgaacuugug....................................................................	1	1	seq
....................aacccguagauccgaacuuAug....................................................................	2	1	seq
....................aacccguagauGcgaacuugug....................................................................	5	1	seq
....................aacccguagaCccgaacuugug....................................................................	3	1	seq
....................aaccGguagauccgaacuugug....................................................................	2	1	seq
....................aacccguagauccgaacuuCug....................................................................	4	1	seq
....................aacccgGagauccgaacuugug....................................................................	15	1	seq
....................aacccguagauccgaacuuguU....................................................................	128	1	seq
....................aacccguagauccgaacCugug....................................................................	4	1	seq
....................aacccguagauccUaacuugug....................................................................	10	1	seq
....................aacccUuagauccgaacuugug....................................................................	14	1	seq
....................aacccguagaucUgaacuugug....................................................................	35	1	seq
....................aacccgAagauccgaacuugug....................................................................	2	1	seq
....................aacccguagaGccgaacuugug....................................................................	1	1	seq
....................aacccguagauccgaacuugCg....................................................................	13	1	seq
....................aacUcguagauccgaacuugug....................................................................	2	1	seq
....................aacccguagauccgaacuuguA....................................................................	497	1	seq
....................aacccguagGuccgaacuugug....................................................................	11	1	seq
....................aacccCuagauccgaacuugug....................................................................	4	1	seq
....................Cacccguagauccgaacuugug....................................................................	11	1	seq
....................aacccguagauccgaacAugug....................................................................	3	1	seq
....................aacccguagauccgaacuugAg....................................................................	8	1	seq
....................aacccguagauccgaacuuguC....................................................................	7	1	seq
....................Gacccguagauccgaacuugug....................................................................	50	1	seq
....................aacccguagauccgGacuugug....................................................................	4	1	seq
....................aGcccguagauccgaacuugug....................................................................	3	1	seq
....................aacccguagauccgaacuuUug....................................................................	14	1	seq
....................aacccguagaucAgaacuugug....................................................................	6	1	seq
....................aacccguagauccgaacGugug....................................................................	3	1	seq
....................aaccAguagauccgaacuugug....................................................................	3	1	seq
....................aacccguaAauccgaacuugug....................................................................	2	1	seq
....................aacccguagauccCaacuugug....................................................................	2	1	seq
....................aacccAuagauccgaacuugug....................................................................	2	1	seq
....................aacccguagauccgaaUuugug....................................................................	13	1	seq
....................aacAcguagauccgaacuugugg...................................................................	1	1	seq
....................aacccgGagauccgaacuugugg...................................................................	2	1	seq
....................aacccguagauccgaacuugugg...................................................................	6726	0	seq
....................Uacccguagauccgaacuugugg...................................................................	1	1	seq
....................aacccguagaucAgaacuugugg...................................................................	2	1	seq
....................aacccguagauccgaacuugugC...................................................................	62	1	seq
....................aacccgCagauccgaacuugugg...................................................................	2	1	seq
....................aacccguagauGcgaacuugugg...................................................................	1	1	seq
....................aacccguagGuccgaacuugugg...................................................................	2	1	seq
```

Mature **Star**

```
gaccccugccuguugccgcaaacccguagauccgaacuuguggucauauuccacacaagcuuguaucuauagguaugugucugucuggcaagugcacaaaggguugug
....................aacccguagauccUaacuugugg.................................................................	1	1	seq
....................aacccguagauccgaUcuugugg.................................................................	624	1	seq
....................aacccguagauccgaacuuguAg.................................................................	3	1	seq
....................aacccguagauccgaacuugCgg.................................................................	2	1	seq
....................aacccguagauccgaacuugugA.................................................................	33967	1	seq
....................aacccguagauUcgaacuugugg.................................................................	5	1	seq
....................aaccUguagauccgaacuugugg.................................................................	1	1	seq
....................aGcccguagauccgaacuugugg.................................................................	1	1	seq
....................Gacccguagauccgaacuugugg.................................................................	6	1	seq
....................aacccguagaucGgaacuugugg.................................................................	1	1	seq
....................aaccAguagauccgaacuugugg.................................................................	2	1	seq
....................aacGcguagauccgaacuugugg.................................................................	1	1	seq
....................aacccguagaucUgaacuugugg.................................................................	8	1	seq
....................aacccguaCauccgaacuugugg.................................................................	1	1	seq
....................aacccguagauccCaacuugugg.................................................................	1	1	seq
....................aacccguagauccgaaUuugugg.................................................................	2	1	seq
....................aacccguagauccgaacuuguUg.................................................................	3	1	seq
....................aacccguagauccgaacuugugU.................................................................	6203	1	seq
....................aaAccguagauccgaacuugugg.................................................................	3	1	seq
....................aacccguagauccgaaAuugugg.................................................................	1	1	seq
....................aaGccguagauccgaacuugugg.................................................................	1	1	seq
....................aacccgAagauccgaacuugugg.................................................................	2	1	seq
....................aacccguagauccgaacuugGgg.................................................................	5	1	seq
....................aacUcguagauccgaacuugugg.................................................................	1	1	seq
....................aacccUuagauccgaacuugugg.................................................................	1	1	seq
....................aacccguagauccgaacuuguggu................................................................	50	0	seq
....................aacccguagauccgaacuuguggA................................................................	49	1	seq
....................aacccguagauccgaUcuuguggu................................................................	1	1	seq
....................aacccguagauccgaacuugugAu................................................................	73	1	seq
....................aacccguagauccgaacuugugUu................................................................	9	1	seq
....................aacccguagauccgaacuuguggucauauucU........................................................	1	1	seq
....................aacccguagauccgaacuuguggucauauuccacacU...................................................	3	1	seq
....................aacccguagauccgaacuuguggucauauuccacacaa..................................................	3	0	seq
.....................aAccguagauccgaacuu.....................................................................	1	1	seq
.....................acccguagauccgaacuug....................................................................	4	0	seq
.....................acccguagauccgaacuugu...................................................................	14	0	seq
.....................acccguagauccgaaUuugug..................................................................	1	1	seq
.....................acccguagauccgaacuugug..................................................................	19	0	seq
.....................acccguagauccgaacuugugg.................................................................	25	0	seq
.....................acccguagauccgaacuugugU.................................................................	3	1	seq
.....................acccguagauccgaacuugugA.................................................................	16	1	seq
.....................acccguagauccgaUcuugugg.................................................................	1	1	seq
......................cccguagauccgaacuugu...................................................................	1	0	seq
......................cccguagauccgaacuugug..................................................................	2	0	seq
......................cccguagauccgaacuugugg.................................................................	1	0	seq
.......................ccguagauccgaacuugu...................................................................	35	0	seq
.......................ccguagauccgaacuugG...................................................................	1	1	seq
.......................ccguagauccgaacuugug..................................................................	9	0	seq
.......................ccguagauccgaacuugugU.................................................................	5	1	seq
.......................ccguagauccgaacuugugA.................................................................	20	1	seq
.......................ccguagauccgaacuugugg.................................................................	4	0	seq
.......................ccguagauccgaUcuugugg.................................................................	1	1	seq
.......................ccguagauccgaacuugugC.................................................................	1	1	seq
........................cguagauccgaacuugug..................................................................	21	0	seq
........................cguagauccgaacuuguA..................................................................	1	1	seq
........................cguagauccgaacuugugU.................................................................	16	1	seq
........................cguagauccgaacuugugA.................................................................	28	1	seq
........................cguagauccgaacuugugg.................................................................	4	0	seq
.........................guagauccgaacuugugA.................................................................	2	1	seq
.........................guagauccgaacuugugU.................................................................	3	1	seq
.........................guagauccgaacuugugg.................................................................	1	0	seq
.......................................................caagcuuguaucuauaggua.................................	1	0	seq
.......................................................caagcuuguaucuauagguau................................	4	0	seq
.......................................................Gaagcuuguaucuauagguaug...............................	1	1	seq
.......................................................caagcuuguaucuauagguauU...............................	2	1	seq
.......................................................caagcuuguaucuauagguaug...............................	68	0	seq
.......................................................caagcuuguaucuauaggGaug...............................	1	1	seq
.......................................................caagcuuguaucuauagguaugu..............................	1	0	seq
.........................................................agcuuguaucuauagguaug...............................	1	0	seq
..........................................................gcuuguaucuauagguaug...............................	1	0	seq
```

```
                    Mature                             Star
gaccccugccuguugccgcaaacccguagauccgaacuuguggucauauuccacacaagcuuguaucuauagguaugugucugucuggcaagugcacaaaggguugug
...........................................................cuuguaucuauagguaug...............................    2    0    seq
```

Provisional ID	:	mun-miR-153-1
Score total	:	756.9
Score for star read(s)	:	3.9
Score for read counts	:	746.5
Score for mfe	:	1.9
Score for randfold	:	1.6
Score for cons. seed	:	3
Total read count	:	1476
Mature read count	:	1462
Loop read count	:	0
Star read count	:	14

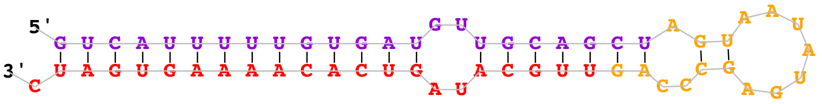

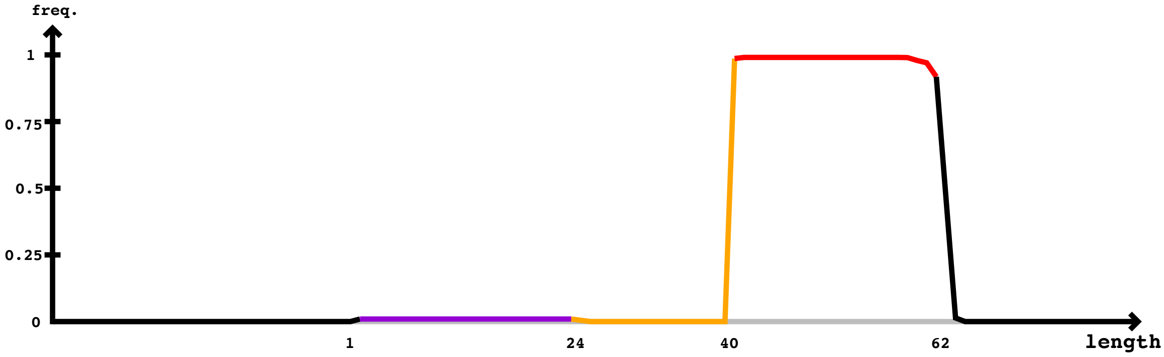

Star Mature

```
5'- uauuuugccagcuaauuagcgguugccagugucauuuuugugauguugcagcuaguaauaugagcccaguugcauagucacaaaagugaucauuggaaacugugacucugc -3'  obs
    uauuuugccagcuaauuagcgguugccagugucauuuuugugauguugcagcuaguaauaugagcccaguugcauagucacaaaagugaucauuggaaacugugacucugc       exp
    ..........((......((((((.((((((((((((((((((..(((((((.((.......))..)))))))..))))))))))))).))))))).))))))......))  reads  mm  sample
    ..............................Aucauuuuugugauguugcagcu..........................................................  1      1   seq
    ..............................gucauuuuugugauguugcagcu..........................................................  12     0   seq
    ..............................gucauuuuugugauguugcagcuU.........................................................  1      1   seq
    .....................................................................uugcauagucacaaaagu........................  1      0   seq
    .....................................................................uugcauagucacaaaagug.......................  15     0   seq
    .....................................................................uugcauagCcacaaaagug.......................  1      1   seq
    .....................................................................uugcauagucacaaaaguga......................  11     0   seq
    .....................................................................uugcauagucacaaaagugG......................  2      1   seq
    .....................................................................uugcauagucacaaaagugau.....................  76     0   seq
    .....................................................................uugcauagucacaaaagugauc....................  1141   0   seq
    .....................................................................uugcauaUucacaaaagugauc....................  1      1   seq
    .....................................................................uugcauagucaUaaaagugauc....................  6      1   seq
    .....................................................................uugcauagucacaaaGgugauc....................  1      1   seq
    .....................................................................uugcauagucacaaaagugauG....................  103    1   seq
    .....................................................................uugcauaguUacaaaagugauc....................  2      1   seq
    .....................................................................uugcauagucacaaaaguUauc....................  1      1   seq
    .....................................................................Augcauagucacaaaagugauc....................  2      1   seq
    .....................................................................uugcaGagucacaaaagugauc....................  1      1   seq
    .....................................................................uugcauagucacaaaagugauU....................  8      1   seq
    .....................................................................uugcGuagucacaaaagugauc....................  1      1   seq
    .....................................................................uugcauagucacaaaagGgauc....................  2      1   seq
    .....................................................................uugcauagucacaaaagugauA....................  40     1   seq
    .....................................................................uugcauagucacaaaagugauca...................  16     0   seq
    .....................................................................uugcauagucacaaaagugaucU...................  5      1   seq
    .....................................................................uugcauagucacaaaagugaucaG..................  1      1   seq
    .....................................................................uugcauagucacaaaagugaucau..................  19     0   seq
    ......................................................................ugcauagucacaaaagugau.....................  1      0   seq
    ......................................................................ugcauagucacaaaagugauc....................  5      0   seq
```

Provisional ID	:	mun-miR-19a
Score total	:	1545.3
Score for star read(s)	:	3.9
Score for read counts	:	1535.2
Score for mfe	:	1.6
Score for randfold	:	1.6
Score for cons. seed	:	3
Total read count	:	3023
Mature read count	:	3005
Loop read count	:	0
Star read count	:	18

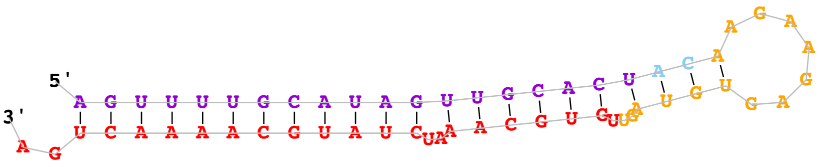

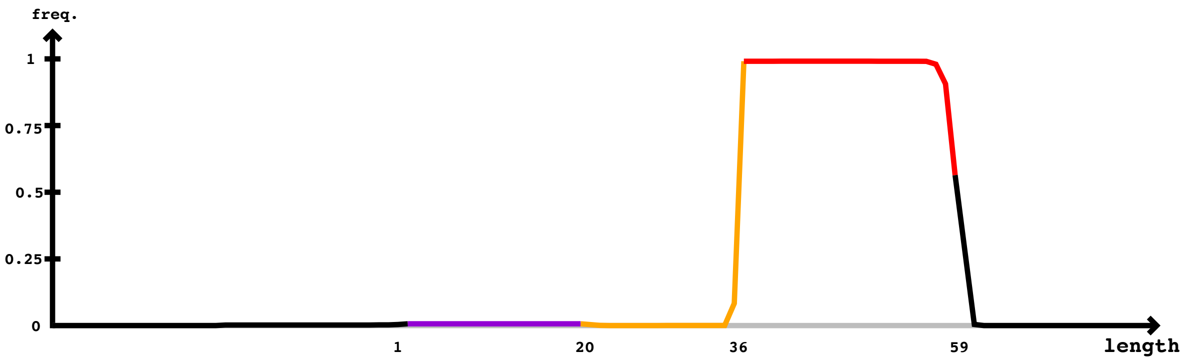

Star **Mature**

```
5'- uguuccaguagcuuucugcuuugcagucuucuguuaguuuugcauaguugcacuacaagaagaguguaguugugcaaaucuaugcaaaacugaugguggccuguuacaaccua  -3'  obs
    uguuccaguagcuuucugcuuugcagucuucuguuaguuuugcauaguugcacuacaagaagaguguaguugugcaaaucuaugcaaaacugaugguggccuguuacaaccua       exp
    (((..(((..(((..((((...))))....(((((((((((((((((((((((((((.......))))...))))))..))))))))))))))))).))))))..))).....  reads  mm  sample
    ................ugcuuugcagucuucuguu..............................................................................  6      0   seq
    ................................guuaguuuugcauaguugcacu...........................................................  2      0   seq
    ..................................uaguuuugcauaguugcacu...........................................................  2      0   seq
    ..................................uaguuuugcauaguugcacua..........................................................  2      0   seq
    ...................................aguuuugcauaguugcacu...........................................................  7      0   seq
    ...................................aguuuugcauaguugcacua..........................................................  5      0   seq
    ...................................aguuuugcauaguugcacuaU.........................................................  1      1   seq
    ...................................aguuuugcauaguugcacuac.........................................................  1      0   seq
    ..............................................................aguguaguugugcaaaucuaug.............................  1      0   seq
    .....................................................................uugugcaaaucuaugcaaaac.......................  3      0   seq
    .....................................................................uugugcaaaucuaugcaaaacu......................  62     0   seq
    .....................................................................uugugcaaaucuGugcaaaacu......................  1      1   seq
    .....................................................................uugugcaaaucuaugcaaaacA......................  1      1   seq
    .....................................................................uugugcaaaucuGugcaaaacug.....................  1      1   seq
    .....................................................................Gugugcaaaucuaugcaaaacug.....................  1      1   seq
    .....................................................................uugugcaaaucuaugcaaaacug.....................  74     0   seq
    .....................................................................uugugcaaaucuaugcaaaacugG....................  1      1   seq
    .....................................................................Gugugcaaaucuaugcaaaacuga....................  1      1   seq
    .....................................................................uugugcaaaucuaugcaaaacugC....................  1      1   seq
    .....................................................................uugugcaaaucuaugcaaaacugU....................  37     1   seq
    .....................................................................uugugcaaaucuaugcaaaacuga....................  62     0   seq
    .....................................................................uugugcaaaucuaugcaaaacugau...................  5      0   seq
    .....................................................................uugugcaaaucuaugcaaaacugauU..................  1      1   seq
    ......................................................................ugugcaaaucuaugcaaaa........................  1      0   seq
    ......................................................................ugugcaaaucuaugcaaaac.......................  26     0   seq
    ......................................................................uguCcaaaucuaugcaaaac.......................  1      1   seq
    ......................................................................ugugcaaaucuaugUaaaac.......................  1      1   seq
    ......................................................................ugugcaaCucuaugcaaaac.......................  1      1   seq
    ......................................................................ugugcaaaucuaugcaGaac.......................  1      1   seq
    ......................................................................ugugcaaaucuaugUaaaacu......................  7      1   seq
    ......................................................................ugugcaaaucuaugcaaaacu......................  147    0   seq
    ......................................................................ugugcaaaucuGugcaaaacu......................  1      1   seq
    ......................................................................ugugcaaaucuaugcaaaaAu......................  1      1   seq
```

Star Mature

```
uguuccaguagcuuucugcuuugcagucuucuguuaguuuugcauaguugcacuacaagaagaguguaguugugcaaaucuaugcaaaacugaugguggccuguuacaaccua
......................................................................ugugcaaaucuaugcaGaacu......................  1     1  seq
......................................................................ugugcaaaucuauCcaaaacu......................  1     1  seq
......................................................................ugugcaaaucuaugcaaaacA......................  1     1  seq
......................................................................ugugcaaaucuaugUaaaacug.....................  2     1  seq
......................................................................ugugcaaaucuaugcaGaacug.....................  2     1  seq
......................................................................ugugcaaaucAaugcaaaacug.....................  1     1  seq
......................................................................Cgugcaaaucuaugcaaaacug.....................  1     1  seq
......................................................................ugugcaaaucuaugcaaaacug.....................  950   0  seq
......................................................................Ggugcaaaucuaugcaaaacug.....................  1     1  seq
......................................................................ugCgcaaaucuaugcaaaacug.....................  1     1  seq
......................................................................ugugUaaaucuaugcaaaacug.....................  1     1  seq
......................................................................ugugcaaaucuGugcaaaacug.....................  3     1  seq
......................................................................ugugcaaaucuGugcaaaacuga....................  1     1  seq
......................................................................ugugcaaaucuaugcaaaacuga....................  1001  0  seq
......................................................................ugugcaaaucuaugUaaaacuga....................  2     1  seq
......................................................................ugugcaaaucuaugcaaaacugU....................  423   1  seq
......................................................................ugugcaaauAuaugcaaaacuga....................  1     1  seq
......................................................................ugugcaaaucuauUcaaaacuga....................  1     1  seq
......................................................................ugugcaaaucuaugcaGaacuga....................  3     1  seq
......................................................................ugugcaaaucuaugcaaaacugG....................  9     1  seq
......................................................................ugugcGaaucuaugcaaaacuga....................  1     1  seq
......................................................................ugugcaaaucuaugcaaaacugC....................  12    1  seq
......................................................................ugugcaaaucuaugcaaGacuga....................  3     1  seq
......................................................................ugugcaaaucuaugcaaaacugUu...................  30    1  seq
......................................................................ugugcaaaucuaugcaaaacugaC...................  7     1  seq
......................................................................ugugcaaaucuaugUaaaacugau...................  1     1  seq
......................................................................ugugcaaaucuaugcaaaacugau...................  53    0  seq
......................................................................ugugcaaaucCaugcaaaacugau...................  40    1  seq
......................................................................ugugcaaaucuaugcaaaacugaA...................  1     1  seq
......................................................................ugugcaaaucuaugcaaaacugauU..................  12    1  seq
..........................................................................caaaucuaugcaaaacuga....................  1     0  seq
```

Provisional ID	:	mun-miR-128-2
Score total	:	4784.7
Score for star read(s)	:	3.9
Score for read counts	:	4774.1
Score for mfe	:	2.1
Score for randfold	:	1.6
Score for cons. seed	:	3
Total read count	:	9376
Mature read count	:	9371
Loop read count	:	0
Star read count	:	5

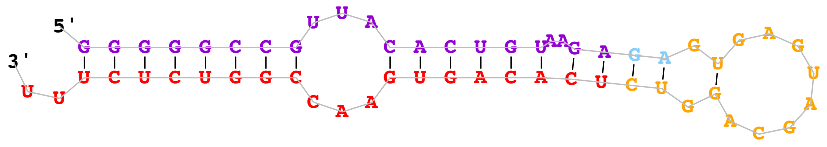

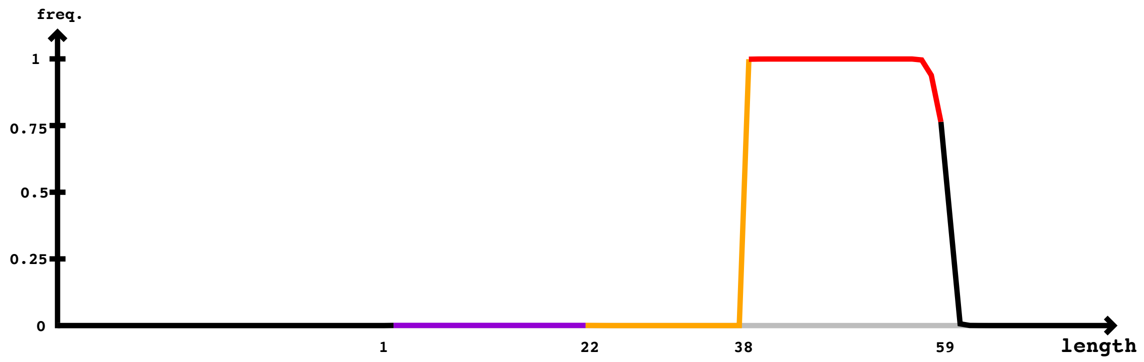

Star Mature

```
5'- caccagcucccugcagaagggugcagcuggaaggggggccguuacacuguaagagagugaguagcaggucucacagugaaccggucucuuuuccugcugugucaugau   -3'   obs
    caccagcucccugcagaagggugcagcuggaaggggggccguuacacuguaagagagugaguagcaggucucacagugaaccggucucuuuuccugcugugucaugau         exp
    .....((.....)).....((..((((.(((((((((((((...((((((..((((.(........).))))))))))...))))))))))))).)))).)).....   reads   mm   sample
    .................................gggggccguuacacuguaaga......................................................   4       0    seq
    .................................gggggccguuacacuguaagaga....................................................   1       0    seq
    .....................................................................cucacagugaaccggucucuuu.................   1       0    seq
    ......................................................................ucacagugaaccggGcuc....................   1       1    seq
    ......................................................................ucacagugaaccAgucuc....................   1       1    seq
    ......................................................................ucacagugaaccggucuc....................   31      0    seq
    ......................................................................ucacagugaaccggucucC...................   1       1    seq
    ......................................................................ucacagugaaccggucucu...................   514     0    seq
    ......................................................................ucacagugaaccggCcucu...................   1       1    seq
    ......................................................................ucacagugaaccggucuUu...................   6       1    seq
    ......................................................................ucacagugaaccggucucA...................   11      1    seq
    ......................................................................Acacagugaaccggucucu...................   1       1    seq
    ......................................................................ucaUagugaaccggucucu...................   1       1    seq
    ......................................................................Ccacagugaaccggucucu...................   1       1    seq
    ......................................................................ucacagugaaccgCucucu...................   1       1    seq
    ......................................................................ucacagugaaccggucAcu...................   1       1    seq
    ......................................................................ucacagugaaUcggucucuu..................   3       1    seq
    ......................................................................ucacagugaaccggAcucuu..................   1       1    seq
    ......................................................................ucacagCgaaccggucucuu..................   1       1    seq
    ......................................................................ucacagugaaccggucucuC..................   5       1    seq
    ......................................................................ucacagugaacUggucucuu..................   1       1    seq
    ......................................................................ucacagugaaccggGcucuu..................   2       1    seq
    ......................................................................ucCcagugaaccggucucuu..................   1       1    seq
    ......................................................................ucacagugaaccggucucAu..................   1       1    seq
    ......................................................................ucacagugaaccggucucuu..................   1603    0    seq
    ......................................................................ucacagugaaccggucuUuu..................   5       1    seq
    ......................................................................ucaAagugaaccggucucuu..................   1       1    seq
    ......................................................................ucacaUugaaccggucucuu..................   1       1    seq
    ......................................................................ucacaAugaaccggucucuu..................   1       1    seq
    ......................................................................ucacagugaaccggucucuG..................   6       1    seq
    ......................................................................Gcacagugaaccggucucuu..................   1       1    seq
    ......................................................................ucacagGgaaccggucucuu..................   1       1    seq
    ......................................................................ucacagugaaccUgucucuu..................   1       1    seq
```

Star **Mature**

```
caccagcucccugcagaagggugcagcuggaaggggggccguuacacuguaagagagugaguagcaggucucacagugaaccggucucuuuuccugcugugucaugau
......................................................................ucacagugaaccggucucuA..................   6      1   seq
......................................................................ucacagugaaccggCcucuuu.................   2      1   seq
......................................................................Ccacagugaaccggucucuuu.................   3      1   seq
......................................................................ucacagugaaccggucucuuC.................   33     1   seq
......................................................................ucaUagugaaccggucucuuu.................   1      1   seq
......................................................................ucacaguUaaccggucucuuu.................   1      1   seq
......................................................................ucacagugaaccggucucuuu.................   5401   0   seq
......................................................................ucUcagugaaccggucucuuu.................   2      1   seq
......................................................................ucacaCugaaccggucucuuu.................   1      1   seq
......................................................................ucacagugaaccggGcucuuu.................   6      1   seq
......................................................................ucacagugaaccggucucuuA.................   33     1   seq
......................................................................ucacagugaaccggucucuGu.................   1      1   seq
......................................................................ucacaguAaaccggucucuuu.................   1      1   seq
......................................................................ucacagugaaccgguGucuuu.................   1      1   seq
......................................................................ucacagugaaccggucuAuuu.................   1      1   seq
......................................................................ucacagugaaccggucucuuG.................   9      1   seq
......................................................................ucacagugaaccggucucuCu.................   6      1   seq
......................................................................ucacagugaaccggucuGuuu.................   2      1   seq
......................................................................ucacagugaaccAgucucuuu.................   1      1   seq
......................................................................Acacagugaaccggucucuuu.................   2      1   seq
......................................................................uUacagugaaccggucucuuu.................   2      1   seq
......................................................................ucacagugaaUcggucucuuu.................   4      1   seq
......................................................................ucacagugaaccgguAucuuu.................   1      1   seq
......................................................................ucacagugaaccggucucCuu.................   1      1   seq
......................................................................ucacagugGaccggucucuuu.................   4      1   seq
......................................................................ucacagugaaccggucucuAu.................   22     1   seq
......................................................................ucacagugaaccggucuUuuu.................   10     1   seq
......................................................................ucacagugaUccggucucuuu.................   1      1   seq
......................................................................ucacagCgaaccggucucuuu.................   1      1   seq
......................................................................Gcacagugaaccggucucuuu.................   1      1   seq
......................................................................ucacagGgaaccggucucuuu.................   2      1   seq
......................................................................uGacagugaaccggucucuuu.................   2      1   seq
......................................................................ucacaUugaaccggucucuuu.................   2      1   seq
......................................................................ucacagugaaccggAcucuuu.................   2      1   seq
......................................................................ucacaAugaaccggucucuuu.................   2      1   seq
......................................................................ucacagugaaccggucucuuuu................   1256   0   seq
......................................................................ucacagugaaccggucucuuAu................   41     1   seq
......................................................................uUacagugaaccggucucuuuu................   1      1   seq
......................................................................ucacagugaaccggucucuuuC................   46     1   seq
......................................................................uGacagugaaccggucucuuuu................   1      1   seq
......................................................................ucacagugaaccggucucuuuA................   142    1   seq
......................................................................ucacagugaaUcggucucuuuu................   2      1   seq
......................................................................Acacagugaaccggucucuuuu................   2      1   seq
......................................................................Ccacagugaaccggucucuuuu................   1      1   seq
......................................................................ucacagugaaccggGcucuuuu................   3      1   seq
......................................................................ucacagugaaccggucuUuuuu................   4      1   seq
......................................................................ucaAagugaaccggucucuuuu................   1      1   seq
......................................................................ucacagugaaccggucucuuuG................   11     1   seq
......................................................................ucacagCgaaccggucucuuuu................   2      1   seq
......................................................................ucacagugaaccggucucuuGu................   1      1   seq
......................................................................ucacagAgaaccggucucuuuu................   1      1   seq
......................................................................ucacagugaaccggucucuuCu................   10     1   seq
......................................................................uAacagugaaccggucucuuuu................   1      1   seq
......................................................................ucacagugaaccggucucuAuu................   2      1   seq
......................................................................ucacagugaaccggucucGuuu................   1      1   seq
......................................................................ucacagugaaccggucucuuuuA...............   18     1   seq
......................................................................ucacagugaaccggucucuuuuc...............   33     0   seq
......................................................................ucacagugaaccggucucuuuuG...............   3      1   seq
......................................................................ucacagugaaccggucucuuuucU..............   3      1   seq
......................................................................ucacagugaaccggucucuuuuAcu.............   1      1   seq
.......................................................................cacagugaaccggucucuuu.................   1      0   seq
.......................................................................cacagugaaUcggucucuuuu................   1      1   seq
.......................................................................cacagugaaccggucucuuuu................   1      0   seq
.......................................................................cacagugaaccggucucuuuuc...............   3      0   seq
........................................................................acagugaaccggucucuuuu................   1      0   seq
```

Provisional ID	:	mun-miR-15a
Score total	:	2138.8
Score for star read(s)	:	3.9
Score for read counts	:	2129.2
Score for mfe	:	1.1
Score for randfold	:	1.6
Score for cons. seed	:	3
Total read count	:	4188
Mature read count	:	4186
Loop read count	:	0
Star read count	:	2

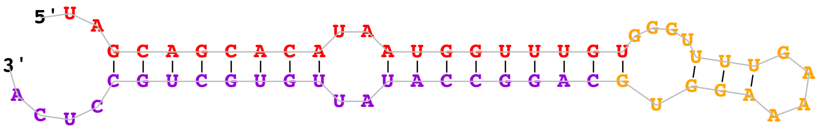

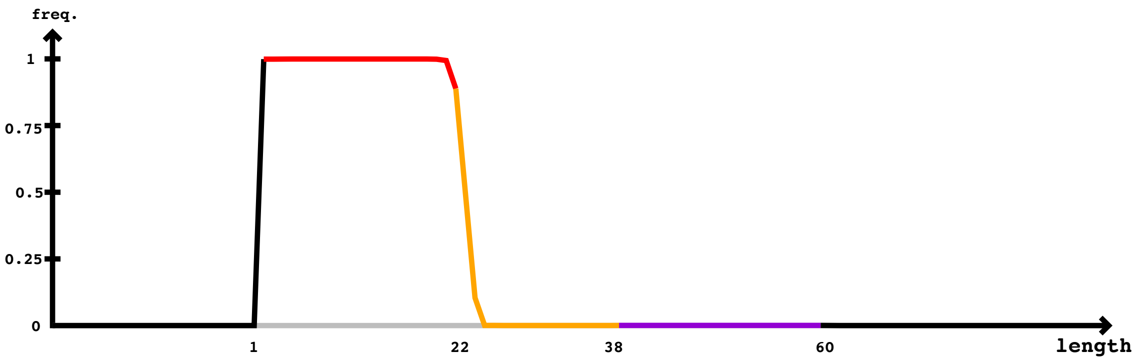

Mature **Star**

```
5'- guagacaccuuggcauaacguagcagcacauaaugguuugugggguuuugaaaaggugcaggccauauugugcugccucaaaaaaacaaggauuucaucauccugaaga   -3'   obs
    guagacaccuuggcauaacguagcagcacauaaugguuuguggguuuugaaaaggugcaggccauauugugcugccucaaaaaaacaaggauuucaucauccugaaga          exp
    .......(((((.......(..((((((((..(((((((((....(((....))).)))))))))..))))))))..).......))))).(((((......))))).   reads   mm   sample
    ....................uagcagcacauaaugguu......................................................................   3       0    seq
    ....................uagcagcacauaaugguuu.....................................................................   21      0    seq
    ....................uagcagcacauaaugguuug....................................................................   442     0    seq
    ....................uagcagcacauaaCgguuugu...................................................................   1       1    seq
    ....................uagcagcacauaaugguuugA...................................................................   1       1    seq
    ....................uagcagcacauUaugguuugu...................................................................   1       1    seq
    ....................uagcagcacauCaugguuugu...................................................................   798     1    seq
    ....................uagcagcacauaauggGuugu...................................................................   2       1    seq
    ....................uagcagcacauaaugguuugu...................................................................   1824    0    seq
    ....................uagcagAacauaaugguuugu...................................................................   1       1    seq
    ....................uagcagcacauaaugguuuAu...................................................................   1       1    seq
    ....................uagcagcacauaauggAuugu...................................................................   1       1    seq
    ....................Gagcagcacauaaugguuugu...................................................................   1       1    seq
    ....................uagcagcacauaaugguuugG...................................................................   3       1    seq
    ....................Cagcagcacauaaugguuugu...................................................................   2       1    seq
    ....................uagcagcacGuaaugguuugu...................................................................   1       1    seq
    ....................uagcagcacauaaugguuugug..................................................................   575     0    seq
    ....................uagcagcacauaauggAuugug..................................................................   1       1    seq
    ....................uagcagcacauaaugguuugGg..................................................................   1       1    seq
    ....................Aagcagcacauaaugguuugug..................................................................   1       1    seq
    ....................uagcagcacauaaugguuuguU..................................................................   25      1    seq
    ....................uagcagcacauaauggGuugug..................................................................   1       1    seq
    ....................uagcagcacauaaugguuuguA..................................................................   43      1    seq
    ....................uagcagcacauaaugguuugugA.................................................................   94      1    seq
    ....................uagcagcacauaaugguuugugU.................................................................   300     1    seq
    ....................uagcagcacauaaugguuugugC.................................................................   8       1    seq
    ....................uagcagcacauaaugguuugugg.................................................................   32      0    seq
    ....................uagcagcacauaaugguuugugggu...............................................................   1       0    seq
    ......................gcagcacauaaugguuuguggguuuugaaaaggug...................................................   1       0    seq
    .......................cagcacauaaugguuugu...................................................................   1       0    seq
    .........................................................caggccauauugugcugccuca.............................   2       0    seq
```

Provisional ID	:	mun-miR-425
Score total	:	5229.9
Score for star read(s)	:	3.9
Score for read counts	:	5219.2
Score for mfe	:	2.2
Score for randfold	:	1.6
Score for cons. seed	:	3
Total read count	:	10249
Mature read count	:	9660
Loop read count	:	0
Star read count	:	589

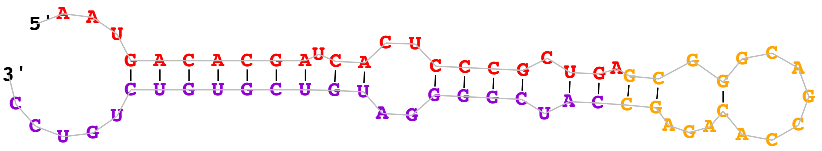

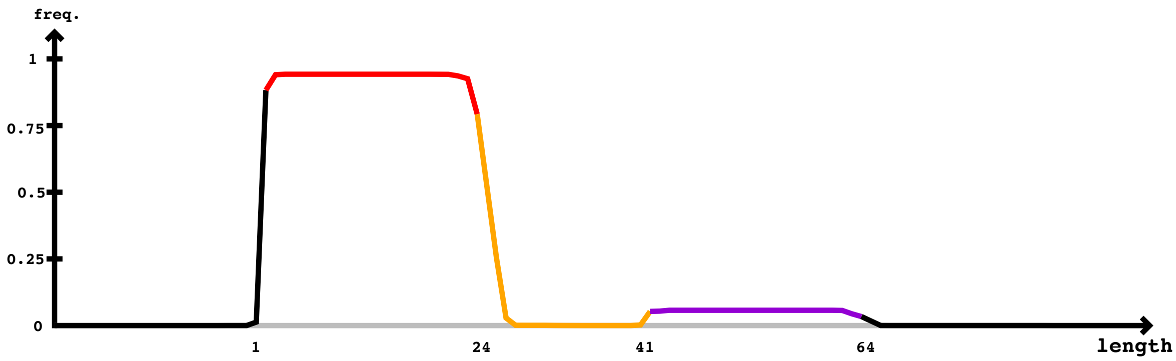

Mature **Star**

```
5'- ccggagagagacggcuuuggaaugacacgaucacucccgcugagcgggcagccacagagccaucggggaugucgugucuguccaaagcucuuucggcaccuccgagagcccc   -3'    obs
    ccggagagagacggcuuuggaaugacacgaucacucccgcugagcgggcagccacagagccaucggggaugucgugucuguccaaagcucuuucggcaccuccgagagcccc          exp
    .(((((.((((.(((((((((..(((((((.((..((((.((.((.(.......)...)))).))))..)))))))))..)))))))))).))))).....)))))........    reads      mm        sample
    ................Guggaaugacacgaucacucccgcuga.....................................................................    1          1         seq
    ...................gaaugacacgaucacucccgcu.......................................................................    2          0         seq
    ...................gaaugacacgaucacucccgcug......................................................................    65         0         seq
    ...................gaaugacacgaucacucccgcugU.....................................................................    57         1         seq
    ...................gaaugacacgaucacucccgcuga.....................................................................    2          0         seq
    ...................gaaugacacgaucacucccgcugagc...................................................................    5          0         seq
    ...................gaaugacacgaucacucccgcugagcgggcag.............................................................    1          0         seq
    ....................aaugacacgaucacuccc..........................................................................    2          0         seq
    ....................aaugacacgaucacucccA.........................................................................    1          1         seq
    ....................aaugacacgaucacucccg.........................................................................    2          0         seq
    ....................aaugacacgaucacucccgU........................................................................    1          1         seq
    ....................aaugacacgaucacucccgc........................................................................    56         0         seq
    ....................aaugacacgaucacucUcgcu.......................................................................    1          1         seq
    ....................aaugacacgaucacucccgcu.......................................................................    104        0         seq
    ....................aGugacacgaucacucccgcug......................................................................    1          1         seq
    ....................aaugacacgaucacucccgcGg......................................................................    3          1         seq
    ....................aaugacacgaucacucccgcuU......................................................................    2          1         seq
    ....................aaugacacAaucacucccgcug......................................................................    2          1         seq
    ....................aaugacacgaucacucccgUug......................................................................    2          1         seq
    ....................aaugacacUaucacucccgcug......................................................................    1          1         seq
    ....................aaugacGcgaucacucccgcug......................................................................    1          1         seq
    ....................aaugacacgaucacucccgcug......................................................................    1279       0         seq
    ....................aaugacacgaucacucccgcuA......................................................................    1          1         seq
    ....................aaugacacgaucacucccUcuga.....................................................................    1          1         seq
    ....................aGugacacgaucacucccgcuga.....................................................................    1          1         seq
    ....................aaugacacgaucacGcccgcuga.....................................................................    1          1         seq
    ....................aaugacacgaucaAucccgcuga.....................................................................    1          1         seq
    ....................aaugacacgaucacucccgcuga.....................................................................    1706       0         seq
    ....................aaugacacgaucacuUccgcuga.....................................................................    1          1         seq
    ....................Uaugacacgaucacucccgcuga.....................................................................    2          1         seq
    ....................aaugacacgauUacucccgcuga.....................................................................    1          1         seq
    ....................aaugacacgaucacuccUgcuga.....................................................................    1          1         seq
    ....................aaugacacgaucacucccgcugC.....................................................................    2          1         seq
```

```
                    Mature                                  Star
ccggagagagacggcuuuggaaugacacgaucacucccgcugagcgggcagccacagagccaucggggaugucgugucuguccaaagcucuuucggcaccuccgagagcccc
....................Gaugacacgaucacucccgcuga....................................................................	3	1	seq
....................aaugacacgaucacucccgcugU....................................................................	2939	1	seq
....................aauAacacgaucacucccgcuga....................................................................	1	1	seq
....................aaugacacgaucacucUcgcuga....................................................................	1	1	seq
....................aaugacacgaucacucccgcugG....................................................................	12	1	seq
....................aaugacacgaucacucccgcugaA...................................................................	35	1	seq
....................aauUacacgaucacucccgcugag...................................................................	1	1	seq
....................aaugacacgaucacucccgcugaC...................................................................	5	1	seq
....................aaugacacgaucacucccgGugag...................................................................	1	1	seq
....................aaugacacgaucacucccCcugag...................................................................	1	1	seq
....................aaugacacgaucacuccAgcugag...................................................................	1	1	seq
....................aaugacacgaucacucccgcuCag...................................................................	1	1	seq
....................aaugacacgaucacAcccgcugag...................................................................	1	1	seq
....................aGugacacgaucacucccgcugag...................................................................	1	1	seq
....................aaugacacgaucacucccgcugag...................................................................	413	0	seq
....................aaugacacgaucacucccgcugaU...................................................................	168	1	seq
....................aaugacacgaucacucccgcugUg...................................................................	1	1	seq
....................aaugacacgaucacuccAgcugagc..................................................................	1	1	seq
....................aauUacacgaucacucccgcugagc..................................................................	2	1	seq
....................aaugacacgaucacucccgcugagA..................................................................	57	1	seq
....................aaugacacgaucacucccgcGgagc..................................................................	1	1	seq
....................aaugacacgaucacucccAcugagc..................................................................	1	1	seq
....................aaugacacUaucacucccgcugagc..................................................................	1	1	seq
....................aaugacacgaucacucccgcugagU..................................................................	124	1	seq
....................aGugacacgaucacucccgcugagc..................................................................	2	1	seq
....................aaugacacgauAacucccgcugagc..................................................................	1	1	seq
....................aaugacacgaucacucccgcugaCc..................................................................	1	1	seq
....................aaugacacgaucacucccgcugGgc..................................................................	1	1	seq
....................aaugaGacgaucacucccgcugagc..................................................................	2	1	seq
....................aaugacacgaucacuccUgcugagc..................................................................	2	1	seq
....................aaugacacgaucacucccCcugagc..................................................................	2	1	seq
....................aaugacacgaucacucccgcugagc..................................................................	1742	0	seq
....................aaugacacgaucacucccUcugagc..................................................................	1	1	seq
....................aaugacacgaucacucccgcugaAc..................................................................	1	1	seq
....................aaugacacgaucacucccgUugagc..................................................................	1	1	seq
....................aaugacacgaucacucccgcugagG..................................................................	1	1	seq
....................aaugacacgaucacucccgcugagcC.................................................................	1	1	seq
....................aaugacacgaucacucccgcugagcA.................................................................	9	1	seq
....................aaugacacgaucacucccgcugagcU.................................................................	200	1	seq
....................aaugacacgaucacucccgcugagcgA................................................................	1	1	seq
....................aaugacacgaucacucccgcugagcgggcU.............................................................	4	1	seq
....................aaugacacgaucacucccgcugagcgggcag............................................................	1	0	seq
....................aaugacacgaucacucccgcugagcgggcagc...........................................................	1	0	seq
....................aaugacacgaucacucccgcugagcgggcagcc..........................................................	1	0	seq
....................aaugacacgaucacucccgcugagcgggcagccacagagc...................................................	1	0	seq
.....................augacacgaucacucccgc.......................................................................	4	0	seq
.....................augacacgaucacucccgcu......................................................................	1	0	seq
.....................augacacgaucacucccgcug.....................................................................	8	0	seq
.....................augacacgaucacucccgcugU....................................................................	16	1	seq
.....................augacacgaucacucccgcuga....................................................................	55	0	seq
.....................augacacgaucacucccgcugaU...................................................................	12	1	seq
.....................augacacgaucacucccgcugaA...................................................................	2	1	seq
.....................augacacgaucacucccgcugag...................................................................	71	0	seq
.....................augacacgaucacucccgcugaAc..................................................................	1	1	seq
.....................augacacgaucacucccgcugagc..................................................................	289	0	seq
.....................augacacgaucacucccgcugagA..................................................................	27	1	seq
.....................augacacgaucacuccUgcugagc..................................................................	1	1	seq
.....................augacacgaucacucccgcugagU..................................................................	33	1	seq
.....................augacacgaucacucccgAugagc..................................................................	1	1	seq
.....................augacacgaucacucccgcugagcA.................................................................	4	1	seq
.....................augacacgaucacucccgcugagcU.................................................................	68	1	seq
......................ugacacgaucacucccgcuga....................................................................	1	0	seq
......................ugacacgaucacucccgcugagc..................................................................	17	0	seq
......................ugacacgaucacucccgcugagU..................................................................	1	1	seq
......................ugacacgaucacucccgcugagA..................................................................	1	1	seq
...........................................................ccaucggggaugucgugucugu..............................	20	0	seq
...........................................................ccaucggggaugucgugGcugu..............................	1	1	seq
...........................................................ccaucggggaugucgugucugA..............................	2	1	seq
............................................................caucggggaugucgugucug...............................	7	0	seq
............................................................caucggggaugucgugucugG..............................	1	1	seq
```

```
                    Mature                                  Star
ccggagagagacggcuuuggaaugacacgaucacucccgcugagcgggcagccacagagccaucggggaugucgugucuguccaaagcucuuucggcaccuccgagagcccc
............................................................Aaucggggaugucgugucugu...............................   1     1   seq
............................................................caucggggaugucgugucugu...............................   103   0   seq
............................................................caucggggaugucgugucuguc..............................   89    0   seq
............................................................caucggggaugucgugucuUuc..............................   1     1   seq
............................................................caucggggaugucgugucuguU..............................   10    1   seq
............................................................caucggggaugucgugGcuguc..............................   1     1   seq
............................................................caucggggaugucgugucuguA..............................   1     1   seq
............................................................Gaucggggaugucgugucuguc..............................   1     1   seq
............................................................caucggggaugucgugucugucc.............................   61    0   seq
............................................................caucggggaugucgugucugucG.............................   1     1   seq
............................................................caucggggaugucgugucugucA.............................   82    1   seq
............................................................caucggggaugucgugucugucU.............................   145   1   seq
............................................................caucggggaugucgugucugucAa............................   1     1   seq
............................................................caucggggaugucgugucugucca............................   3     0   seq
............................................................caucggggaugucgugucuguccU............................   13    1   seq
............................................................caucggggaugucgugucuguccaa...........................   1     0   seq
.............................................................aucggggaugucgugucuguc..............................   2     0   seq
.............................................................aucggggaugucgugucugucA.............................   2     1   seq
.............................................................aucggggaugucgugucugucc.............................   5     0   seq
.............................................................aucggggaugucgugucugucca............................   1     0   seq
..............................................................ucggggaugucgugucugu...............................   2     0   seq
..............................................................ucggggaugucgugucugucc.............................   18    0   seq
..............................................................ucggggaugucgugucugucA.............................   4     1   seq
..............................................................ucggggaugucgugucugucU.............................   9     1   seq
..............................................................ucggggaugucgugucugucAa............................   1     1   seq
...................................................................................aaagcucuuucggcaccuc..........   1     0   seq
```

Provisional ID	:	mun-novel12
Score total	:	17.6
Score for star read(s)	:	3.9
Score for read counts	:	10.8
Score for mfe	:	1.9
Score for randfold	:	1.6
Score for cons. seed	:	-0.6
Total read count	:	33
Mature read count	:	29
Loop read count	:	0
Star read count	:	4

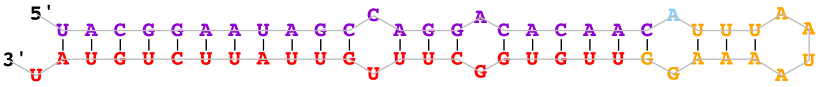

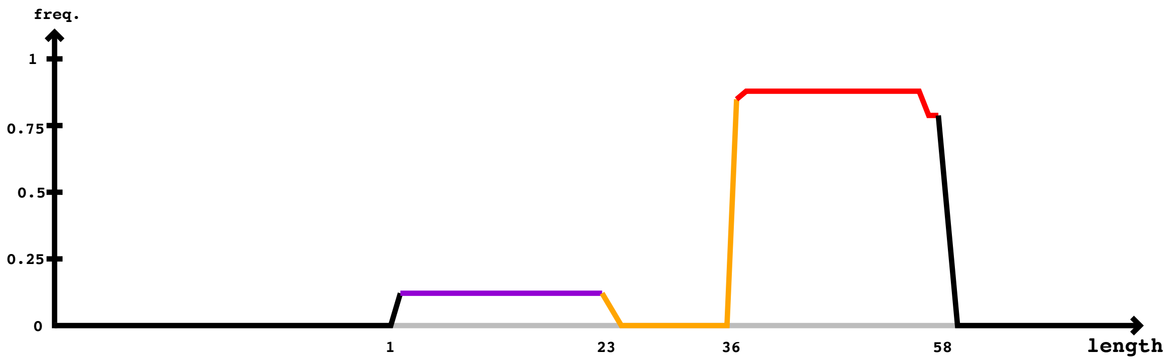

Star **Mature**

```
5'- guaggcucuuacagcuuuuuguaugugguugggguacggaauagccaggacacaacauuuaauaaaagguuguggcuuuguuauucuguauuucagcuauuuuauaaacuc   -3'    obs
    guaggcucuuacagcuuuuuguaugugguuggggguacggaauagccaggacacaacauuuaauaaaagguuguggcuuuguuauucuguauuucagcuauuuuauaaacuc          exp
    ..(((((.....)))))((((((.(((((((..((((((((((((.(((.((((((.(((....))).)))))).))).))))))))))))..)))))))..))))))...    reads   mm   sample
    ..................................uacggaauagccaggacacaaU.......................................................    1       1    seq
    ..................................uacggaauagccaggacacaac.......................................................    3       0    seq
    ....................................................................uuguggcuuuguuauucugu.......................    3       0    seq
    ....................................................................uuguggcuuuguuauucuguau.....................    20      0    seq
    ....................................................................uuguggcuuuguuauucuguaA.....................    3       1    seq
    ....................................................................uuguggcuuuguuauucuguUu.....................    1       1    seq
    ....................................................................uuguggcuuuguuauucuguauu....................    1       0    seq
    .....................................................................uguggcuuuguuauucuguauu....................    1       0    seq
```

Provisional ID	:	mun-miR-23
Score total	:	5.1
Score for star read(s)	:	-1.3
Score for read counts	:	0
Score for mfe	:	1.8
Score for randfold	:	1.6
Score for cons. seed	:	3
Total read count	:	13365
Mature read count	:	13365
Loop read count	:	0
Star read count	:	0

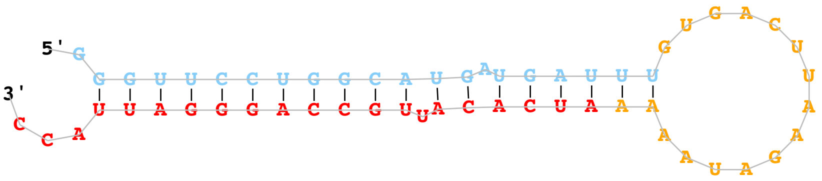

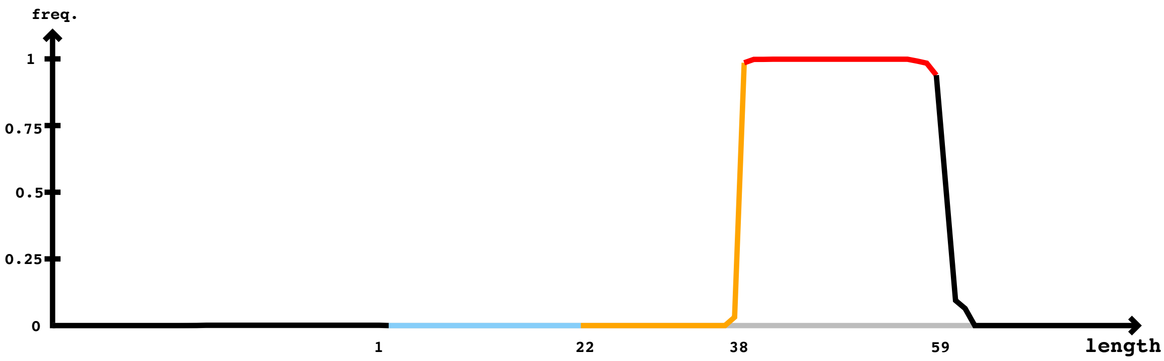


```
                                              Star                              Mature
5'- cugcacagcuuucuuuuaauguugugguuguuugggguuccuggcaugaugauuugugacuuaagauaaaaaucacauugccagggauuaccacacagccauaaccagcucc   -3'   exp
    ......((((..........((((((((((.((((((((((((((.((((((..................)))))))).))))))))))..))).))))))))).))))..   reads   mm   sample
    ............cuuuuaauguugugguuguuu..............................................................................   1       0    seq
    .............uuuuaauguugugguuguuu..............................................................................   3       0    seq
    ..............uuuaauguugugguuguuu..............................................................................   12      0    seq
    .....................................................................aaucacauugccagggau........................   2       0    seq
    .....................................................................aaucacauugccagggauG.......................   1       1    seq
    .....................................................................aaucacauugccagggauu.......................   6       0    seq
    .....................................................................aaucacauugccagggauua......................   1       0    seq
    .....................................................................aaucacauugccagggauuU......................   9       1    seq
    .....................................................................aaucacauugccagggauuUc.....................   26      1    seq
    .....................................................................aaucacauugccagggauuac.....................   1       0    seq
    .....................................................................aaucacauugccagggauuUcc....................   322     1    seq
    .....................................................................aaucacauugccagggauuacc....................   14      0    seq
    .....................................................................aaucacauugccagggauuUcca...................   38      1    seq
    .....................................................................aaucacauugccagggauuaccac..................   1       0    seq
    .....................................................................aaucacauugccagggauuaccacU.................   1       1    seq
    ......................................................................aucacauugccagggauG.......................   1       1    seq
    ......................................................................aucacauugccagggauu.......................   82      0    seq
    ......................................................................aucGcauugccagggauu.......................   1       1    seq
    ......................................................................aucacauugccagggauA.......................   1       1    seq
    ......................................................................aucacauugccagggauuU......................   69      1    seq
    ......................................................................aucacauugccagggauua......................   25      0    seq
    ......................................................................aucacauugcUagggauuac.....................   1       1    seq
    ......................................................................aucacauugUcagggauuac.....................   2       1    seq
    ......................................................................aucacauugccCgggauuac.....................   1       1    seq
    ......................................................................aucacauugccagggauuUc.....................   368     1    seq
    ......................................................................aucacauugccagggauuaU.....................   3       1    seq
    ......................................................................aucacauugccagggauuac.....................   186     0    seq
    ......................................................................aucacauugccagggauuaA.....................   3       1    seq
    ......................................................................aucacauugccagggauuCc.....................   2       1    seq
    ......................................................................aucacauugUcagggauuacc....................   60      1    seq
    ......................................................................aucacauugccaggUauuacc....................   1       1    seq
    ......................................................................aucacauugccagggauuacG....................   1       1    seq
    ......................................................................aucacauuAccagggauuacc....................   1       1    seq
    ......................................................................aucacauugcUagggauuacc....................   1       1    seq
```

Star Mature

```
cugcacagcuuucuuuuaauguugugguuguuuggguuccuggcaugaugauuugugacuuaagauaaaaaaucacauugccagggauuaccacacagccauaaccagcucc
.......................................................................aucacauugccagggauuacc....................	2114	0	seq
.......................................................................aucacauugccagggaGuacc....................	1	1	seq
.......................................................................aAcacauugccagggauuacc....................	1	1	seq
.......................................................................aucacauugAcagggauuacc....................	1	1	seq
.......................................................................aucacauuUccagggauuacc....................	2	1	seq
.......................................................................aucacauugccaAggauuacc....................	1	1	seq
.......................................................................Gucacauugccagggauuacc....................	2	1	seq
.......................................................................aucacauugccagggauuUcc....................	6268	1	seq
.......................................................................aucacauuCccagggauuacc....................	1	1	seq
.......................................................................aucacauugccagggauuacA....................	12	1	seq
.......................................................................aucacauugccagUgauuacc....................	1	1	seq
.......................................................................aucacGuugccagggauuacc....................	1	1	seq
.......................................................................auAacauugccagggauuacc....................	1	1	seq
.......................................................................aucacauugccagggauuacU....................	26	1	seq
.......................................................................aucacauugUcagggauuacca...................	2	1	seq
.......................................................................aucacauugccagggauuaccC...................	1	1	seq
.......................................................................aucacauugccagggauuacca...................	618	0	seq
.......................................................................aucacauugccagggauuaccG...................	2	1	seq
.......................................................................aucacauugccagggauuacAa...................	2	1	seq
.......................................................................aucacauugccagggauuaccU...................	51	1	seq
.......................................................................aAcacauugccagggauuacca...................	1	1	seq
.......................................................................aucacauugcUagggauuacca...................	1	1	seq
.......................................................................aucacauuAccagggauuacca...................	1	1	seq
.......................................................................aucacauugccagggauuUcca...................	1659	1	seq
.......................................................................aucacauugccagggauuGcca...................	1	1	seq
.......................................................................aucacauugccagggauuUccac..................	3	1	seq
.......................................................................aucacauugccagggauuaccac..................	327	0	seq
.......................................................................aucacauuAccagggauuaccac..................	1	1	seq
.......................................................................aucacauugccagggauuaccaU..................	57	1	seq
.......................................................................aucacauugccagggauuaccaA..................	2	1	seq
.......................................................................aucacauugccagggauuaccacG.................	4	1	seq
.......................................................................aucacauugccagggauuUccaca.................	1	1	seq
.......................................................................aucacauugccagggauuaccacC.................	23	1	seq
.......................................................................aucacauugccagggauuaccacU.................	760	1	seq
.......................................................................aucacauugccagggauuaccaca.................	5	0	seq
........................................................................ucacauugccagggauuU......................	2	1	seq
........................................................................ucacauugccagggauuac.....................	4	0	seq
........................................................................ucacauugccagggauuUc.....................	4	1	seq
........................................................................ucacauugccagggauuUcc....................	28	1	seq
........................................................................ucacauugccagggauuacc....................	33	0	seq
........................................................................ucacauugccagggauuacU....................	1	1	seq
........................................................................ucacauugUcagggauuacc....................	1	1	seq
........................................................................ucacauugccagggauuacA....................	1	1	seq
........................................................................ucacauugccagggauuaccU...................	1	1	seq
........................................................................ucacauugccagggauuacca...................	14	0	seq
........................................................................ucacauugccagggauuUcca...................	8	1	seq
........................................................................ucacauugccagggauuaccaU..................	3	1	seq
........................................................................ucacauugccagggauuaccac..................	10	0	seq
........................................................................ucacauugccagggauuaccaca.................	1	0	seq
........................................................................ucacauugccagggauuaccacU.................	60	1	seq
.........................................................................cacauugccagggauuaccacU.................	1	1	seq
..........................................................................acauugccagggauuacc....................	2	0	seq
..........................................................................acauugccagggauuUcc....................	5	1	seq
..........................................................................acauugccagggauuUcca...................	1	1	seq
```

Provisional ID	: mun-miR-30a
Score total	: 46287.8
Score for star read(s)	: 3.9
Score for read counts	: 46277
Score for mfe	: 2.4
Score for randfold	: 1.6
Score for cons. seed	: 3
Total read count	: 90782
Mature read count	: 84688
Loop read count	: 0
Star read count	: 6094

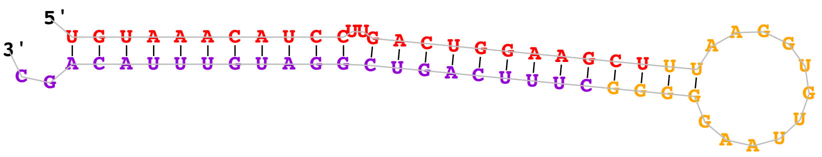

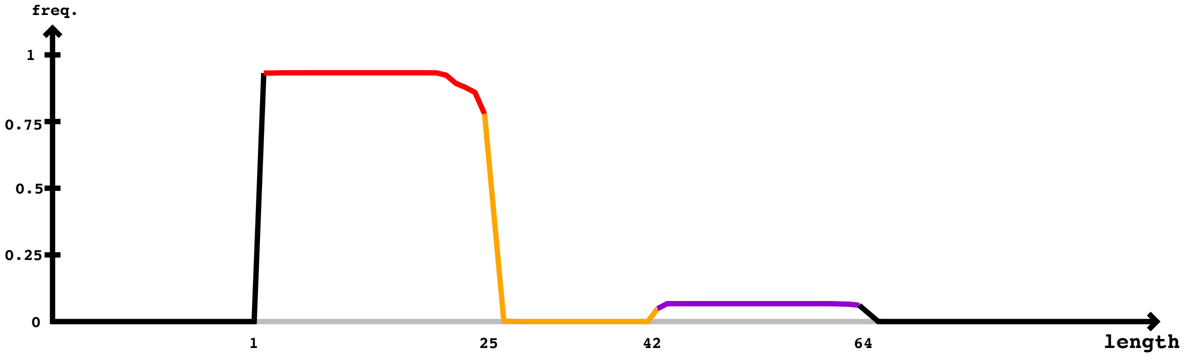

Mature **Star**

```
5'- uuuggggcaguccuggcuacuguaaacauccuugacuggaagcuuuaaggguguuaaggggggcuuucagucggauguuuacagcagcagguugccaugcucaucugagcacagg   -3'   obs
    uuuggggcaguccuggcuacuguaaacauccuugacuggaagcuuuaaggguguuaaggggggcuuucagucggauguuuacagcagcagguugccaugcucaucugagcacagg         exp
    .....(((((.((((.((.((((((((((((..(((((((((((((............))))))))))))))))))))))))).))))))))))).(((((....)))))....   reads   mm   sample
    ..................aAuguaaacauccuugacuggaagcu......................................................................   1       1    seq
    ...................cuguaaacauccuugacuggaag........................................................................   1       0    seq
    ...................cuguaaacauccuugacuggaagcu......................................................................   2       0    seq
    ...................Auguaaacauccuugacuggaagcu......................................................................   1       1    seq
    ....................uguaaacauccuugacug............................................................................   30      0    seq
    ....................uguaaacauccCugacugg...........................................................................   1       1    seq
    ....................Cguaaacauccuugacugg...........................................................................   1       1    seq
    ....................uguaaacaucUuugacugg...........................................................................   4       1    seq
    ....................uUuaaacauccuugacugg...........................................................................   1       1    seq
    ....................uguaaacauccuugacugg...........................................................................   727     0    seq
    ....................uguaaacauccuugacugU...........................................................................   22      1    seq
    ....................uguaaacauccuugacugA...........................................................................   22      1    seq
    ....................uguaaacauccuugaAugg...........................................................................   1       1    seq
    ....................Gguaaacauccuugacugg...........................................................................   1       1    seq
    ....................uguaaacauAcuugacugg...........................................................................   1       1    seq
    ....................uCuaaacauccuugacugg...........................................................................   1       1    seq
    ....................uguaaacauUcuugacugg...........................................................................   1       1    seq
    ....................uguaaacauccuugacugC...........................................................................   1       1    seq
    ....................uguaaacauccuugacugga..........................................................................   2402    0    seq
    ....................Gguaaacauccuugacugga..........................................................................   2       1    seq
    ....................uguaaacGuccuugacugga..........................................................................   1       1    seq
    ....................uguaaacauccuugacuAga..........................................................................   1       1    seq
    ....................uguaaacauccCugacugga..........................................................................   38      1    seq
    ....................uguaaacauUcuugacugga..........................................................................   59      1    seq
    ....................Cguaaacauccuugacugga..........................................................................   2       1    seq
    ....................uguaaacauccuugacCgga..........................................................................   1       1    seq
    ....................uguaaacauccuAgacugga..........................................................................   10      1    seq
    ....................uguaGacauccuugacugga..........................................................................   1       1    seq
    ....................uguaaacauccuugacuggG..........................................................................   8       1    seq
    ....................uguUaacauccuugacugga..........................................................................   1       1    seq
    ....................uguaaacauccuugacugCa..........................................................................   1       1    seq
    ....................Aguaaacauccuugacugga..........................................................................   4       1    seq
    ....................uguaaacauccuugGcugga..........................................................................   1       1    seq
```

```
                    Mature                                      Star
uuuggggcaguccuggcuacuguaaacauccuugacuggaagcuuuaagguguuaaggggcuuucagucggauguuuacagcagcagguugccaugcucaucugagcacagg
....................uguaaacaucAuugacugga.........................................................................   1      1   seq
....................uguaaacauccuugacuggU.........................................................................   194    1   seq
....................uguaaacauccuGgacugga.........................................................................   1      1   seq
....................uguaaacauccuugacuggC.........................................................................   3      1   seq
....................uguaaacauccuugaAugga.........................................................................   1      1   seq
....................uguaaacaucUuugacugga.........................................................................   40     1   seq
....................uguaaacauccuAgacuggaa........................................................................   1      1   seq
....................uguaaacaucUuugacuggaa........................................................................   6      1   seq
....................uguaaacauccuugacuggaa........................................................................   1246   0   seq
....................Gguaaacauccuugacuggaa........................................................................   2      1   seq
....................uguaaacauUcuugacuggaa........................................................................   19     1   seq
....................uguaaacauccuuAacuggaa........................................................................   1      1   seq
....................uguaaacauccuugacuUgaa........................................................................   2      1   seq
....................ugCaaacauccuugacuggaa........................................................................   1      1   seq
....................uguaaacauccuGgacuggaa........................................................................   1      1   seq
....................uguaaacauccuugacuggaC........................................................................   3      1   seq
....................uguaaacauccuugaUuggaa........................................................................   1      1   seq
....................uguaaacauccuugacuggaU........................................................................   86     1   seq
....................uguCaacauccuugacuggaa........................................................................   1      1   seq
....................uguaaacauccCugacuggaa........................................................................   15     1   seq
....................uguaaacauccuugacuggaG........................................................................   9      1   seq
....................ugGaaacauccuugacuggaa........................................................................   1      1   seq
....................ugCaaacauccuugacuggaag.......................................................................   2      1   seq
....................uguaaacaucUuugacuggaag.......................................................................   3      1   seq
....................ugGaaacauccuugacuggaag.......................................................................   1      1   seq
....................uguaaacauccuAgacuggaag.......................................................................   1      1   seq
....................uguaaacauccuugacugAaag.......................................................................   1      1   seq
....................uguaaacauccuugaUuggaag.......................................................................   1      1   seq
....................uguaaacauccCugacuggaag.......................................................................   1      1   seq
....................uguaaacauUcuugacuggaag.......................................................................   7      1   seq
....................uguaaacauccuugacuggaag.......................................................................   1079   0   seq
....................uguaaacaucAuugacuggaag.......................................................................   1      1   seq
....................uguaaacauccuugacuggaaA.......................................................................   548    1   seq
....................uguaaacauccuuUacuggaag.......................................................................   1      1   seq
....................uguaaacauccuugacuggaaU.......................................................................   49     1   seq
....................uguaaacauccuugacuggaaC.......................................................................   9      1   seq
....................Gguaaacauccuugacuggaag.......................................................................   1      1   seq
....................ugCaaacauccuugacuggaagc......................................................................   3      1   seq
....................uguaaacaCccuugacuggaagc......................................................................   1      1   seq
....................uguaaacauAcuugacuggaagc......................................................................   1      1   seq
....................uguaaacauUcuugacuggaagc......................................................................   16     1   seq
....................uguaaGcauccuugacuggaagc......................................................................   1      1   seq
....................uguaaacaucUuugacuggaagc......................................................................   28     1   seq
....................uguaaacauccuugacuggaagc......................................................................   6283   0   seq
....................uguaaacauccuugacuggaagU......................................................................   466    1   seq
....................uguaaacauccuugacugUaagc......................................................................   1      1   seq
....................uguaaacauccCugacuggaagc......................................................................   2      1   seq
....................uguaaacauccuGgacuggaagc......................................................................   1      1   seq
....................uguaaacCuccuugacuggaagc......................................................................   1      1   seq
....................ugAaaacauccuugacuggaagc......................................................................   1      1   seq
....................uguaaacauccuugacugCaagc......................................................................   2      1   seq
....................uguaaacauccuugacuggaaAc......................................................................   1      1   seq
....................uguaaacauccuugacuUgaagc......................................................................   3      1   seq
....................Cguaaacauccuugacuggaagc......................................................................   4      1   seq
....................Gguaaacauccuugacuggaagc......................................................................   4      1   seq
....................uguaaacauccuugacCggaagc......................................................................   4      1   seq
....................uguaGacauccuugacuggaagc......................................................................   1      1   seq
....................uguaaacauccuugacuCgaagc......................................................................   2      1   seq
....................uguaaacauccuugacuggaagG......................................................................   1      1   seq
....................uguaUacauccuugacuggaagc......................................................................   1      1   seq
....................uguaaacauccuugacAggaagc......................................................................   2      1   seq
....................uguaaacauccuugaUuggaagc......................................................................   1      1   seq
....................ugGaaacauccuugacuggaagc......................................................................   2      1   seq
....................uguaaacauccuuUacuggaagc......................................................................   1      1   seq
....................uguaaacauccuugacuggaaUc......................................................................   2      1   seq
....................uguGaacauccuugacuggaagc......................................................................   2      1   seq
....................uguaaacaAccuugacuggaagc......................................................................   1      1   seq
....................uAuaaacauccuugacuggaagc......................................................................   3      1   seq
....................uguaaacauccuugacuggaagA......................................................................   530    1   seq
....................uguaaacauccuugaGuggaagc......................................................................   1      1   seq
```

Mature **Star**

```
uuuggggcaguccuggcuacuguaaacauccuugacuggaagcuuuaagguguuaaggggggcuuucagucggauguuuacagcagcagguugccaugcucaucugagcacagg
....................uUuaaacauccuugacuggaagc......................................................................    3      1    seq
....................uguaaacauccuAgacuggaagc......................................................................    3      1    seq
....................Aguaaacauccuugacuggaagc......................................................................    1      1    seq
....................uguaaacauccuugacuggaGgcu.....................................................................    5      1    seq
....................uguaaaUauccuugacuggaagcu.....................................................................    14     1    seq
....................uguaaaAauccuugacuggaagcu.....................................................................    9      1    seq
....................uguaaacauccuugacuUgaagcu.....................................................................    14     1    seq
....................uguaaacauccuugacuAgaagcu.....................................................................    8      1    seq
....................uguaaacauAcuugacuggaagcu.....................................................................    6      1    seq
....................uguaaacauccuugacCggaagcu.....................................................................    9      1    seq
....................ugCaaacauccuugacuggaagcu.....................................................................    16     1    seq
....................uguaaacauccuuCacuggaagcu.....................................................................    4      1    seq
....................uguaaGcauccuugacuggaagcu.....................................................................    4      1    seq
....................uCuaaacauccuugacuggaagcu.....................................................................    3      1    seq
....................uguaGacauccuugacuggaagcu.....................................................................    10     1    seq
....................uguaaacauccuugaGuggaagcu.....................................................................    4      1    seq
....................uguaaacaGccuugacuggaagcu.....................................................................    2      1    seq
....................uguaaacauccuugacuggaaCcu.....................................................................    5      1    seq
....................uguaaacauccuugacuggaagcC.....................................................................    20     1    seq
....................uguaaacaAccuugacuggaagcu.....................................................................    3      1    seq
....................uguaaUcauccuugacuggaagcu.....................................................................    5      1    seq
....................uguaaacauccAugacuggaagcu.....................................................................    1      1    seq
....................uguaaacauccuugacugAaagcu.....................................................................    3      1    seq
....................uguaaacauccuugacuggaagcG.....................................................................    334    1    seq
....................uguaaacauccuugUcuggaagcu.....................................................................    2      1    seq
....................uguaaacauccuugaUuggaagcu.....................................................................    3      1    seq
....................uguaaacauccuugCcuggaagcu.....................................................................    1      1    seq
....................uguaaacaucAuugacuggaagcu.....................................................................    11     1    seq
....................uguaaacUuccuugacuggaagcu.....................................................................    1      1    seq
....................uguaaacauccuGgacuggaagcu.....................................................................    4      1    seq
....................uguaaacauccuuAacuggaagcu.....................................................................    5      1    seq
....................uguaaacauccuugacGggaagcu.....................................................................    3      1    seq
....................uguaaacauccuAgacuggaagcu.....................................................................    9      1    seq
....................uAuaaacauccuugacuggaagcu.....................................................................    2      1    seq
....................uguaaacGuccuugacuggaagcu.....................................................................    1      1    seq
....................uguaaacauccCugacuggaagcu.....................................................................    15     1    seq
....................uguaaacauUcuugacuggaagcu.....................................................................    31     1    seq
....................uguaaacauccuugacuCgaagcu.....................................................................    4      1    seq
....................Gguaaacauccuugacuggaagcu.....................................................................    57     1    seq
....................uguaaacauccuugacuggaagAu.....................................................................    8      1    seq
....................ugGaaacauccuugacuggaagcu.....................................................................    11     1    seq
....................uguaaacaCccuugacuggaagcu.....................................................................    5      1    seq
....................uguGaacauccuugacuggaagcu.....................................................................    5      1    seq
....................uguaaacauccuugacuggaaAcu.....................................................................    2      1    seq
....................uguaaacauccuugacuggaaUcu.....................................................................    9      1    seq
....................uguaaacauccuugacuggaagcA.....................................................................    202    1    seq
....................uguaaacauccuuUacuggaagcu.....................................................................    11     1    seq
....................uguaaacauccuugacugUaagcu.....................................................................    10     1    seq
....................uguaaacauccuugacuggGagcu.....................................................................    6      1    seq
....................uguaaacauccuugacuggaagGu.....................................................................    1      1    seq
....................uguaaacauccuugaAuggaagcu.....................................................................    10     1    seq
....................uguaaacauccuugacAggaagcu.....................................................................    9      1    seq
....................uguaaacauccuugacuggaagUu.....................................................................    6      1    seq
....................uguaaacauGcuugacuggaagcu.....................................................................    3      1    seq
....................uguaaacaucGuugacuggaagcu.....................................................................    5      1    seq
....................ugAaaacauccuugacuggaagcu.....................................................................    9      1    seq
....................uguaaCcauccuugacuggaagcu.....................................................................    1      1    seq
....................uguaUacauccuugacuggaagcu.....................................................................    2      1    seq
....................uguaaacauccuugacuggaagcu.....................................................................    65670  0    seq
....................uUuaaacauccuugacuggaagcu.....................................................................    16     1    seq
....................uguaaacaucUuugacuggaagcu.....................................................................    32     1    seq
....................Cguaaacauccuugacuggaagcu.....................................................................    30     1    seq
....................Aguaaacauccuugacuggaagcu.....................................................................    33     1    seq
....................uguaaacauccuugacugCaagcu.....................................................................    2      1    seq
....................uguUaacauccuugacuggaagcu.....................................................................    1      1    seq
....................uguaaacauccuugacuggCagcu.....................................................................    1      1    seq
....................uguaaacauccuugGcuggaagcu.....................................................................    4      1    seq
....................uguaaacauccuCgacuggaagcuu....................................................................    1159   1    seq
....................Aguaaacauccuugacuggaagcuu....................................................................    1      1    seq
....................uguaaacauccuugacuggGagcuu....................................................................    1      1    seq
```

```
                    Mature                                    Star
uuuggggcaguccuggcuacuguaaacauccuugacuggaagcuuuaaggguguuaagggggcuuucagucggauguuuacagcagcagguugccaugcucaucugagcacagg
....................uguaaacauccCugacuggaagcuu.....................................................................   1     1   seq
....................ugAaaacauccuugacuggaagcuu.....................................................................   1     1   seq
....................uguaaacauccuugacuggaagcuC.....................................................................   2     1   seq
....................uguaaaAauccuugacuggaagcuu.....................................................................   1     1   seq
....................uguaaacauccuugacuggaagcCu.....................................................................   7     1   seq
....................uguaaacauccuugacuggaagcuu.....................................................................   2402  0   seq
....................uguaaacUuccuugacuggaagcuu.....................................................................   1     1   seq
....................uguaaacauccuugacuggaagcuA.....................................................................   23    1   seq
....................uguaaacauccuugacuggaagcAu.....................................................................   1     1   seq
....................Gguaaacauccuugacuggaagcuu.....................................................................   3     1   seq
....................ugCaaacauccuugacuggaagcuu.....................................................................   1     1   seq
....................uCuaaacauccuugacuggaagcuu.....................................................................   1     1   seq
....................uguaaacauccuugacuggaaUcuu.....................................................................   1     1   seq
....................uguaaacauccuuUacuggaagcuu.....................................................................   2     1   seq
....................uguaaacauccuugacugUaagcuu.....................................................................   2     1   seq
....................ugCaaacauccuugacuggaagcuuu....................................................................   1     1   seq
....................uguaaacauccuugacuggaagcuuA....................................................................   33    1   seq
....................uguaaacauccuugacuggaagcuGu....................................................................   2     1   seq
....................uguaaacauccuugacuggaagcuuC....................................................................   1     1   seq
....................uguaaacauccuugacuggaagcuuu....................................................................   73    0   seq
....................uguaaacauccuugacuggaagcuAu....................................................................   2     1   seq
....................uguaaacauccuugacuggaagcuCu....................................................................   9     1   seq
....................uguaaacauccuugacuggaagcuuG....................................................................   1     1   seq
....................uguaaacauccuCgacuggaagcuuu....................................................................   39    1   seq
....................Gguaaacauccuugacuggaagcuuu....................................................................   1     1   seq
....................uguaaacauccuugacuggaagcuuuC...................................................................   1     1   seq
....................uguaaacauccuCgacuggaagcuuua...................................................................   4     1   seq
....................uguaaacauccuugacuggaagcuAua...................................................................   2     1   seq
....................uguaaacauccuugacuggaagcuuuU...................................................................   7     1   seq
....................uguaaacauccuugacuggaagcuuua...................................................................   5     0   seq
....................uguaaacauccuugacuggaagcuuuUa..................................................................   2     1   seq
....................uguaaacauccuugacuggaagcuuuaaggguguuaaggggg....................................................   1     0   seq
.....................guaaacauccuugacuggaagcu......................................................................   10    0   seq
.....................guaaacauccuCgacuggaagcuu.....................................................................   2     1   seq
.....................guaaacauccuugacuggaagcuu.....................................................................   2     0   seq
.....................guaaacauccuugacuggaagcuuu....................................................................   1     0   seq
......................uaaacauccuugacuggaagc.......................................................................   2     0   seq
......................uaaacauccuugacuggaagcu......................................................................   67    0   seq
......................uaaacauccuugacuggaagcG......................................................................   1     1   seq
......................uaaacauccuugacuggaagcuu.....................................................................   5     0   seq
.........................Ccauccuugacuggaagcu......................................................................   1     1   seq
.........................acauccuugacuggaagcu......................................................................   1     0   seq
............................................uuaaggguguuaagggggcuuucagucggauguuuacagcU.............................   1     1   seq
..............................................................cuuucagucggauguuuaU.................................   1     1   seq
..............................................................cuuucagucggaugAuuac.................................   1     1   seq
..............................................................cuuucagucggauguuuac.................................   91    0   seq
..............................................................cuuucagucggauguuuaA.................................   3     1   seq
..............................................................Guuucagucggauguuuaca................................   1     1   seq
..............................................................Auuucagucggauguuuaca................................   1     1   seq
..............................................................cuuucagucggauguuuaAa................................   1     1   seq
..............................................................cuuucagucggauguuuacU................................   4     1   seq
..............................................................cuuucagucggauguuuaca................................   74    0   seq
..............................................................cuuucagucggauUuuuaca................................   1     1   seq
..............................................................cuCucagucggauguuuacag...............................   1     1   seq
..............................................................cuuucagucggauguuuacaU...............................   1     1   seq
..............................................................cuuucagucggauguuuacag...............................   236   0   seq
..............................................................cuuucaUucggauguuuacag...............................   1     1   seq
..............................................................cuuucagucggauguuuacaA...............................   13    1   seq
..............................................................Guuucagucggauguuuacagc..............................   1     1   seq
..............................................................cuuucagucggauguuuacagG..............................   1     1   seq
..............................................................cuuucaguAggauguuuacagc..............................   1     1   seq
..............................................................cuuucagucggauguuuacagA..............................   7     1   seq
..............................................................cuuucagucAgauguuuacagc..............................   1     1   seq
..............................................................cuuucagucggauguuuacagU..............................   23    1   seq
..............................................................cuuucagucggGuguuuacagc..............................   1     1   seq
..............................................................cuuucagucggaugCuuacagc..............................   1     1   seq
..............................................................cuuucagucUgauguuuacagc..............................   2     1   seq
..............................................................cuuuAagucggauguuuacagc..............................   2     1   seq
..............................................................cuuucagCcggauguuuacagc..............................   2     1   seq
..............................................................cuuucagucggauUuuuacagc..............................   1     1   seq
```

Mature Star

uuuggggcaguccuggcuacuguaaacauccuugacuggaagcuuuaagguguuaagggggcuuucagucggauguuuacagcagcagguugccaugcucaucugagcacagg			
....cuuucagucggauguuuaAagc..............................	2	1	seq
....cuuucagucgAauguuuacagc..............................	1	1	seq
....cuuucagGcggauguuuacagc..............................	1	1	seq
....cuuucaguUggauguuuacagc..............................	4	1	seq
....cuuucagucggauguuuacagc..............................	3685	0	seq
....Auuucagucggauguuuacagc..............................	3	1	seq
....cuuGcagucggauguuuacagc..............................	1	1	seq
....cuuucagucggauguuCacagc..............................	1	1	seq
....Uuuucagucggauguuuacagc..............................	3	1	seq
....cuuucaUucggauguuuacagc..............................	2	1	seq
....cuuucagucggauguuuaUagc..............................	2	1	seq
....cuuucagucggauguuuUcagc..............................	1	1	seq
....cuuCcagucggauguuuacagc..............................	1	1	seq
....cuuucagucggauguuuacagcU.............................	125	1	seq
....cuuucagucggauguuuacagca.............................	39	0	seq
....cuuucagucggauguuuacagcC.............................	7	1	seq
....cuuucagucggauguuuacagUa.............................	1	1	seq
....cuuucagucggauguuuGcagca.............................	35	1	seq
....cuuucagucggauguuuacagAa.............................	1	1	seq
....uuucagucggauguuuac.................................	5	0	seq
....uuucagucggauguuuacU................................	1	1	seq
....uuucagucggauguuuaca................................	11	0	seq
....uuucagucggauguuuacaA...............................	4	1	seq
....uuucagucggauguuuacag...............................	82	0	seq
....uuucUgucggauguuuacagc..............................	1	1	seq
....uuucagucggaugCuuacagc..............................	1	1	seq
....uuucagucggaugAuuacagc..............................	1	1	seq
....uuucagucUgauguuuacagc..............................	1	1	seq
....uuucagucggauguuuacagU..............................	7	1	seq
....Cuucagucggauguuuacagc..............................	1	1	seq
....uuucagucggauguuuacagA..............................	22	1	seq
....uAucagucggauguuuacagc..............................	1	1	seq
....uuucagucggauguuuaAagc..............................	1	1	seq
....uuucagucggaugGuuacagc..............................	1	1	seq
....uuucagucggauguuuacagc..............................	1077	0	seq
....uuucagucggGuguuuacagc..............................	1	1	seq
....uuucaAucggauguuuacagc..............................	1	1	seq
....uuucagucggauguuuacagUa.............................	1	1	seq
....uuucagucggauguuuacagca.............................	9	0	seq
....uuucagucggauguuuacagcU.............................	392	1	seq
....uuucagucggauguuuacagcC.............................	57	1	seq
....uuucagucggauguuuacagAa.............................	4	1	seq
....uuucagucggauguuuGcagca.............................	2	1	seq
....uuucagucggauguuuacagcaU............................	9	1	seq
....uuucagucggauguuuacagcaA............................	2	1	seq
....uucagucggauguuuacagc..............................	7	0	seq
....uucagucggauguuuacagU..............................	1	1	seq
....uucagucggauguuuacagcU.............................	2	1	seq
....ucagucggauguuuacagc..............................	1	0	seq

Provisional ID	:	mun-miR-22
Score total	:	87488.3
Score for star read(s)	:	3.9
Score for read counts	:	87478
Score for mfe	:	1.9
Score for randfold	:	1.6
Score for cons. seed	:	3
Total read count	:	171596
Mature read count	:	171436
Loop read count	:	1
Star read count	:	159

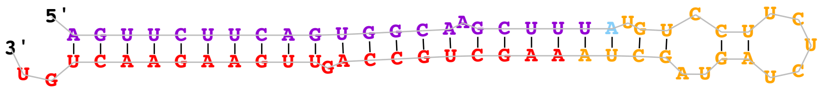

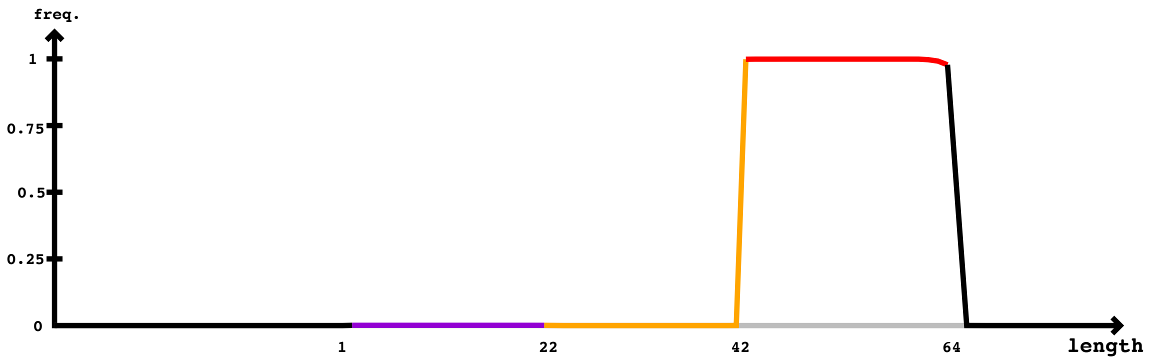


```
                                 Star                                        Mature
5'- cagcgccccgcuccuggcuaaccagcaccaguucuucaguggcaagcuuuauguccuucucuaguagcuaaagcugccaguugaagaacuguugaauguagccacacuc  -3'   obs
    cagcgccccgcuccuggcuaaccagcaccaguucuucaguggcaagcuuuauguccuucucuaguagcuaaagcugccaguugaagaacuguugaauguagccacacuc         exp
    .((((...))))..((((((.....((.((((((((((((((((.((((((.((.((.....))..))))))))))))).))))))))))).))....)))))).....   reads   mm   sample
    ............................caguucuucaguggcaagcuu............................................................   1       0    seq
    ............................caguucuucaguggcaagcuuu...........................................................   1       0    seq
    .............................aguucuucaguggcaagcu.............................................................   3       0    seq
    .............................aguucuucaguggcaagcuu............................................................   15      0    seq
    .............................aguucuucagCggcaagcuuu...........................................................   1       1    seq
    .............................aguucuucaguggcaagcuuu...........................................................   77      0    seq
    .............................aguucuucaguggcaagcuuuU..........................................................   2       1    seq
    .............................aguucuucaguggcaagcuuua..........................................................   56      0    seq
    .............................aguucuucaguggcaagcuuuaA.........................................................   2       1    seq
    .............................aguucuucaguggcaagcuuuau.........................................................   1       0    seq
    ...................................................uguccuucucuaguagcuaaagc...................................   1       0    seq
    ....................................................................uaGagcugccaguugaagaacugu.................   1       1    seq
    ....................................................................Aaaagcugccaguugaagaacugu.................   1       1    seq
    .....................................................................aaagcugccaguugaagaacu...................   1       0    seq
    .....................................................................aaagcugccaguugaagaacug..................   3       0    seq
    .....................................................................Caagcugccaguugaagaacugu.................   3       1    seq
    .....................................................................aaagcugccaguugaagaacugu.................   29      0    seq
    ......................................................................aagcugccaguugaagaa.....................   20      0    seq
    ......................................................................Gagcugccaguugaagaac....................   1       1    seq
    ......................................................................aagcugccaguugaagaac....................   354     0    seq
    ......................................................................aagcugccaguugaagaaU....................   1       1    seq
    ......................................................................aagcugccaguugaagaacu...................   872     0    seq
    ......................................................................aagcugccaguCgaagaacu...................   1       1    seq
    ......................................................................aagcugccaguugaaCaacu...................   1       1    seq
    ......................................................................aagcugAcaguugaagaacu...................   1       1    seq
    ......................................................................aagAugccaguugaagaacu...................   1       1    seq
    ......................................................................aagcugccaguugUagaacu...................   1       1    seq
    ......................................................................aagcugccaguugaagaacA...................   5       1    seq
    ......................................................................aagcugcUaguugaagaacu...................   1       1    seq
    ......................................................................aagcugccaguugaagaacG...................   1       1    seq
    ......................................................................aagcugccaguugaGgaacu...................   1       1    seq
    ......................................................................aagcugccagGugaagaacu...................   1       1    seq
    ......................................................................aagcugccaguugaagaGcu...................   1       1    seq
```

```
                              Star                                        Mature
cagcgccccgcuccuggcuaaccagcaccaguucuucaguggcaagcuuuauguccuucucuaguagcuaaagcugccaguugaagaacuguugaauguagccacacuc
......................................................................aagcugccaguugaagaaUu................... 1       1   seq
......................................................................Gagcugccaguugaagaacu................... 1       1   seq
......................................................................aagcuUccaguugaagaacug.................. 2       1   seq
......................................................................aagcugUcaguugaagaacug.................. 1       1   seq
......................................................................aagcugccagGugaagaacug.................. 2       1   seq
......................................................................aagcugccagCugaagaacug.................. 1       1   seq
......................................................................aagcugccaguuUaagaacug.................. 2       1   seq
......................................................................aaCcugccaguugaagaacug.................. 1       1   seq
......................................................................aagcugccaguugaagaacAg.................. 1       1   seq
......................................................................aagcugccaguugaagaacGg.................. 3       1   seq
......................................................................aagcugccaguugaagaaAug.................. 1       1   seq
......................................................................aagcugcUaguugaagaacug.................. 1       1   seq
......................................................................Cagcugccaguugaagaacug.................. 2       1   seq
......................................................................aagcugccaguugaagGacug.................. 1       1   seq
......................................................................aagcugccaguugaagaacuU.................. 1       1   seq
......................................................................aagcugAcaguugaagaacug.................. 1       1   seq
......................................................................aagcugccGguugaagaacug.................. 2       1   seq
......................................................................aagcugccaguuAaagaacug.................. 1       1   seq
......................................................................aagcugccaguugaagaacug.................. 2359    0   seq
......................................................................aagcugccaguugaagaacuA.................. 1       1   seq
......................................................................aagcugccaguCgaagaacug.................. 1       1   seq
......................................................................aagcugccaguugaaCaacug.................. 1       1   seq
......................................................................aagcugccaguugaagaacCg.................. 1       1   seq
......................................................................aagcugcGaguugaagaacugu................. 8       1   seq
......................................................................aagcugccaguugGagaacugu................. 16      1   seq
......................................................................aagcugccaguugCagaacugu................. 6       1   seq
......................................................................aagcugccaguugaagGacugu................. 20      1   seq
......................................................................aagcugccaguugaagaaGugu................. 4       1   seq
......................................................................aaUcugccaguugaagaacugu................. 31      1   seq
......................................................................aagcugccaguugaagUacugu................. 13      1   seq
......................................................................aagcugccaguugaGgaacugu................. 13      1   seq
......................................................................aagcugccaguugaCgaacugu................. 5       1   seq
......................................................................aagcugccUguugaagaacugu................. 7       1   seq
......................................................................aagcugccaguuUaagaacugu................. 34      1   seq
......................................................................aagcuCccaguugaagaacugu................. 5       1   seq
......................................................................aagcugccagAugaagaacugu................. 10      1   seq
......................................................................aagcugccaguugaagaacugu................. 165528  0   seq
......................................................................aagcugAcaguugaagaacugu................. 30      1   seq
......................................................................aagcugccaguugaaAaacugu................. 13      1   seq
......................................................................Cagcugccaguugaagaacugu................. 36      1   seq
......................................................................aagcugccaguGgaagaacugu................. 2       1   seq
......................................................................aagcugccaguugaagaGcugu................. 14      1   seq
......................................................................aagcugccaguugaagaacuUu................. 28      1   seq
......................................................................aagcugccaguAgaagaacugu................. 3       1   seq
......................................................................aagcugccaguugaagaacuCu................. 8       1   seq
......................................................................aagcugccaUuugaagaacugu................. 20      1   seq
......................................................................aagcugccaguugUagaacugu................. 5       1   seq
......................................................................aagcugccaguugaagaacCgu................. 8       1   seq
......................................................................aagcugccaguuAaagaacugu................. 19      1   seq
......................................................................aagcugccaguugaagaacAgu................. 4       1   seq
......................................................................aagcugccGguugaagaacugu................. 21      1   seq
......................................................................aagcugccagGugaagaacugu................. 14      1   seq
......................................................................aagcugccaguugaagaaAugu................. 25      1   seq
......................................................................aagcugccaAuugaagaacugu................. 11      1   seq
......................................................................aagcugUcaguugaagaacugu................. 40      1   seq
......................................................................aagcCgccaguugaagaacugu................. 28      1   seq
......................................................................aagcugccaguugaagaaUugu................. 7       1   seq
......................................................................aagcugccaguugaagCacugu................. 3       1   seq
......................................................................aagUugccaguugaagaacugu................. 4       1   seq
......................................................................aagcugccaguugaagaacugC................. 60      1   seq
......................................................................aagGugccaguugaagaacugu................. 2       1   seq
......................................................................aGgcugccaguugaagaacugu................. 19      1   seq
......................................................................aagcugccaguugaagaUcugu................. 6       1   seq
......................................................................aagAugccaguugaagaacugu................. 22      1   seq
......................................................................aagcuAccaguugaagaacugu................. 28      1   seq
......................................................................aagcugccaguugaagaacugG................. 371     1   seq
......................................................................aagcugccaguugaaUaacugu................. 40      1   seq
......................................................................aagcugccaguugaagaacGgu................. 5       1   seq
......................................................................aagcugccaCuugaagaacugu................. 4       1   seq
......................................................................aagcugccaguugaUgaacugu................. 14      1   seq
```

```
                             Star                                       Mature
cagcgccccgcuccuggcuaaccagcaccaguucuucaguggcaagcuuuauguccuucucuaguagcuaaagcugccaguugaagaacuguugaauguagccacacuc
......................................................................aagcuUccaguugaagaacugu.................   36    1    seq
......................................................................aaAcugccaguugaagaacugu.................   10    1    seq
......................................................................aagcugcAaguugaagaacugu.................   22    1    seq
......................................................................aaCcugccaguugaagaacugu.................   7     1    seq
......................................................................aagcugcUaguugaagaacugu.................   23    1    seq
......................................................................aagcAgccaguugaagaacugu.................   5     1    seq
......................................................................aagcugccaguCgaagaacugu.................   22    1    seq
......................................................................aagcugccaguugaaCaacugu.................   9     1    seq
......................................................................aagcGgccaguugaagaacugu.................   4     1    seq
......................................................................aagcugccCguugaagaacugu.................   5     1    seq
......................................................................Gagcugccaguugaagaacugu.................   132   1    seq
......................................................................aagcugccagCugaagaacugu.................   24    1    seq
......................................................................Uagcugccaguugaagaacugu.................   32    1    seq
......................................................................aagcugccaguugaagaacugA.................   47    1    seq
......................................................................aagcugccaguuCaagaacugu.................   12    1    seq
......................................................................aUgcugccaguugaagaacugu.................   2     1    seq
......................................................................aagcugGcaguugaagaacugu.................   9     1    seq
......................................................................aCgcugccaguugaagaacugu.................   4     1    seq
......................................................................aagcugccaguugaagaacuAu.................   8     1    seq
......................................................................aagcugccaguugaagaacuguu................   348   0    seq
......................................................................aagcugccaguugaaCaacuguu................   1     1    seq
......................................................................aagcugccaguugaagaacuguA................   235   1    seq
......................................................................aagcugccaguugaagaacuguC................   1     1    seq
......................................................................aagcugccaguugaagaacugCu................   7     1    seq
......................................................................Gagcugccaguugaagaacuguu................   2     1    seq
......................................................................aagcugccaguugaagaacuguug...............   2     0    seq
......................................................................aagcugccaguugaagaacuguAg...............   2     1    seq
......................................................................aagcugccaguugaagaacuguuA...............   10    1    seq
......................................................................aagcugccaguugaagaacuguuU...............   13    1    seq
......................................................................aagcugccaguugaagaacuguugG..............   11    1    seq
......................................................................aagcugccaguugaagaacuguuAa..............   1     1    seq
.......................................................................agcugccaguugaagaacugG.................   1     1    seq
.......................................................................agcugccaguugaagaacugu.................   78    0    seq
.......................................................................agcugccaguugaagaacuguA................   1     1    seq
.......................................................................agcugccaguugaaUaacuguu................   1     1    seq
.........................................................................cugccaguugaagaacugu.................   1     0    seq
..........................................................................ugccaguugaagaacugu.................   5     0    seq
```

Provisional ID	:	mun-novel76
Score total	:	94.6
Score for star read(s)	:	3.9
Score for read counts	:	91.9
Score for mfe	:	1.6
Score for randfold	:	-2.2
Score for cons. seed	:	-0.6
Total read count	:	192
Mature read count	:	188
Loop read count	:	0
Star read count	:	4

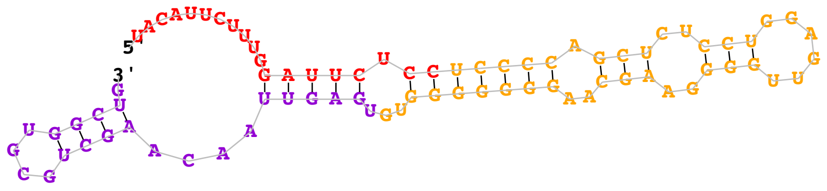

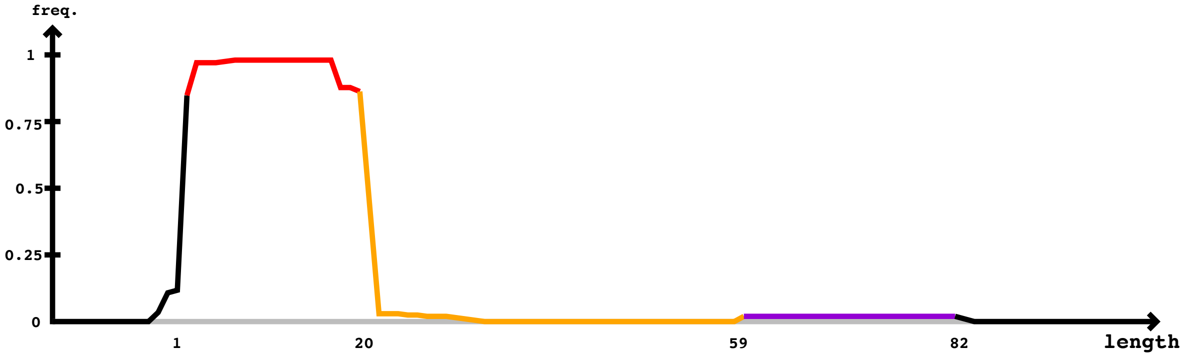

Mature **Star**

```
5'- acccaugggagcuacauucuuuggauucuccuccccagcucuccuggaguuggggaagcaaggggggggugugaguuaacaagcugcguggcugggguuuaaaccacgacaauug  -3'   obs
    acccaugggagcuacauucuuuggauucuccuccccagcucuccuggaguuggggaagcaaggggggggugugaguuaacaagcugcguggcuggguuuaaaccacgacaauug         exp
    ((((.....(((((...(((..(((((.(((((((.(((..(((......)))..)))..)))))))...)))))...)))....)))))))))....................    reads     mm      sample
    .........agGuacauucuuuggauuc......................................................................................    1         1       seq
    .........agcuGcauucuuuggauuc......................................................................................    1         1       seq
    .........agcuacauucuuuggauuc......................................................................................    3         0       seq
    .........agcuaUauucuuuggauuc......................................................................................    1         1       seq
    .........agcuacauGcuuuggauuc......................................................................................    1         1       seq
    ..........gAuacauucuuuggauuc......................................................................................    1         1       seq
    ..........gcuacauucuuuggauuc......................................................................................    13        0       seq
    ..........gcuacauucuuuggauucucc...................................................................................    1         0       seq
    ...........cuacauucuuuggauucucc...................................................................................    2         0       seq
    ............uacGuucuuuggauucuc....................................................................................    1         1       seq
    ............uacauucuuuggauucuc....................................................................................    2         0       seq
    ............uacauucuuuggauuGucc...................................................................................    1         1       seq
    ............uacauucuuuggauucucc...................................................................................    118       0       seq
    ............uacGuucuuuggauucucc...................................................................................    5         1       seq
    ............Cacauucuuuggauucucc...................................................................................    1         1       seq
    ............uacauucuuuggauucuccA..................................................................................    1         1       seq
    ............uGcauucuuuggauucuccu..................................................................................    1         1       seq
    ............uacauucuuuggauucuccu..................................................................................    15        0       seq
    ............uacCuucuuuggauucuccu..................................................................................    1         1       seq
    ............uacauucuuuggauucuccuccc...............................................................................    1         0       seq
    ............uacauucuuuggaCucuccuccccagcuc.........................................................................    1         1       seq
    ............uacauucuuuggaCucuccuccccagcucu........................................................................    1         1       seq
    .............acauucuuuggauucucc...................................................................................    5         0       seq
    .............acauucuuuggauucuccu..................................................................................    19        0       seq
    .............acauucuuuggauucuccucccca.............................................................................    1         0       seq
    ................uucuuuggauucuccuccccaAcu..........................................................................    1         1       seq
    .................ucuuuggauAcuccuccccagcucuc.......................................................................    1         1       seq
    .......................................................................ugaguuaacaagcugcguggcug....................    3         0       seq
    .......................................................................ugaguuaacaagcugcguggcugU...................    1         1       seq
```

Provisional ID : mun-miR-204
Score total : 14895.3
Score for star read(s) : 3.9
Score for read counts : 14885
Score for mfe : 1.8
Score for randfold : 1.6
Score for cons. seed : 3
Total read count : 29208
Mature read count : 29180
Loop read count : 0
Star read count : 28

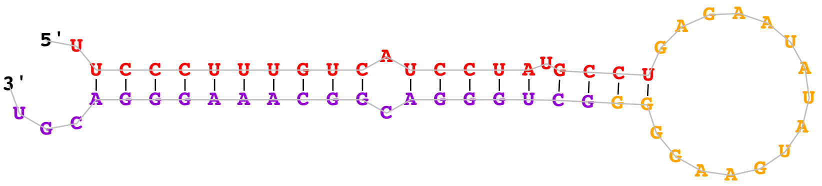

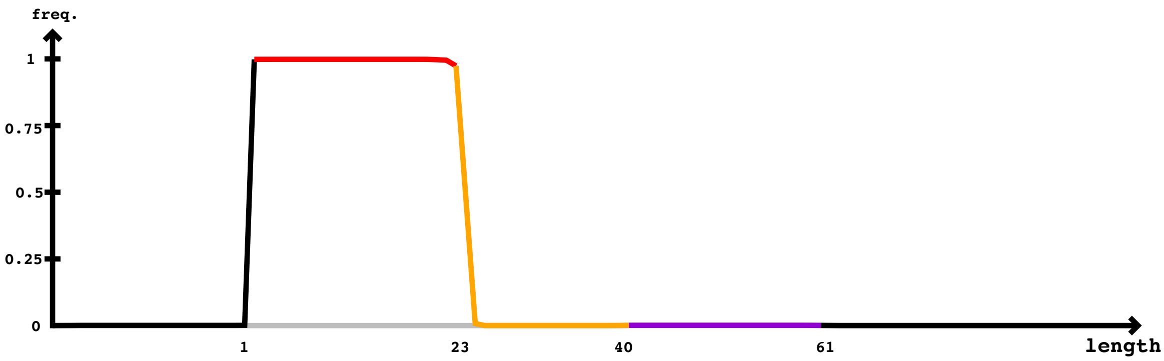

Mature Star

```
5'- guucaugugacccguggacuucccuuugucauccuaugccugagaauauaugaagggggcugggacggcaaagggacguucaacugucaucacuggcaucuucucugacca   -3'   obs
    guucaugugacccguggacuucccuuugucauccuaugccugagaauauaugaagggggcugggacggcaaagggacguucaacugucaucacuggcaucuucucugacca         exp
    (((..((((((...(((((.((((((((((.(((((.((((................)))))))))..))))))))))..)))))...)))).))..))).............   reads   mm   sample
    guucaugugacccguggac............................................................................................   10      0    seq
    .uucaugugacccguggac............................................................................................   8       0    seq
    ..................cuucccuuugucauccuaugccu......................................................................   5       0    seq
    ..................Auucccuuugucauccuaugccu......................................................................   1       1    seq
    ...................uucccuuugucauccuau..........................................................................   3       0    seq
    ...................uucccuuugGcauccuaug.........................................................................   1       1    seq
    ...................uucccuuugucauccuaug.........................................................................   37      0    seq
    ...................uucccuuugucauccuauU.........................................................................   1       1    seq
    ...................uucccuuugucauccuaugU........................................................................   2       1    seq
    ...................uucccuuugucauccuaugc........................................................................   57      0    seq
    ...................uucccuuuguUauccuaugcc.......................................................................   1       1    seq
    ...................uucccuuugucauccuaugcU.......................................................................   2       1    seq
    ...................uucccuuugGcauccuaugcc.......................................................................   1       1    seq
    ...................uucccuuugAcauccuaugcc.......................................................................   1       1    seq
    ...................uucccuuugucauccuaugcA.......................................................................   4       1    seq
    ...................Aucccuuugucauccuaugcc.......................................................................   1       1    seq
    ...................uucccuuugucauccuaugcc.......................................................................   611     0    seq
    ...................uAcccuuugucauccuaugccu......................................................................   2       1    seq
    ...................uucccuuugucauccuaugccG......................................................................   399     1    seq
    ...................uucccuuugucauccuaugGcu......................................................................   2       1    seq
    ...................uuUccuuugucauccuaugccu......................................................................   1       1    seq
    ...................uCccuuugucauccuaugccu.......................................................................   2       1    seq
    ...................uucccuuugucaCccuaugccu......................................................................   2       1    seq
    ...................uucccuCugucauccuaugccu......................................................................   3       1    seq
    ...................uucccCuugucauccuaugccu......................................................................   10      1    seq
    ...................uucAcuuugucauccuaugccu......................................................................   2       1    seq
    ...................uucccuuugucGuccuaugccu......................................................................   11      1    seq
    ...................uucccuuugucauccuaugcGu......................................................................   1       1    seq
    ...................uucccuuugucauccuaCgccu......................................................................   3       1    seq
    ...................uucccuuugucauccuaugAcu......................................................................   7       1    seq
    ...................uucccuuugucauccuauCccu......................................................................   5       1    seq
    ...................uucccuuugucauccuCugccu......................................................................   1       1    seq
    ...................uucccuuugucaucUuaugccu......................................................................   6       1    seq
```

Mature **Star**

```
guucaugugacccguggacuucccuuugucauccuaugccugagaauauaugaagggggcugggacggcaaagggacguucaacugucaucacuggcaucuucucugacca
...................uucccuuugucaucAuaugccu......................................................................  4      1  seq
...................uucccuuugucauccuaGgccu......................................................................  1      1  seq
...................uuGccuuugucauccuaugccu......................................................................  3      1  seq
...................uucccuuugucauccuaugcUu......................................................................  7      1  seq
...................uucccuuugucauAcuaugccu......................................................................  4      1  seq
...................uucccuuuguUauccuaugccu......................................................................  14     1  seq
...................uucccuuugucauccuauUccu......................................................................  4      1  seq
...................uucccuuugucUuccuaugccu......................................................................  1      1  seq
...................Gucccuuugucauccuaugccu......................................................................  18     1  seq
...................uucccuuCgucauccuaugccu......................................................................  3      1  seq
...................uucccuuuAucauccuaugccu......................................................................  2      1  seq
...................uucGcuuugucauccuaugccu......................................................................  2      1  seq
...................uuccUuuugucauccuaugccu......................................................................  2      1  seq
...................uucccuuugucauccuaugccC......................................................................  12     1  seq
...................uucccuuuguGauccuaugccu......................................................................  3      1  seq
...................uucccuuuUucauccuaugccu......................................................................  4      1  seq
...................uucccuuugucauGcuaugccu......................................................................  1      1  seq
...................uucccuuugucaAccuaugccu......................................................................  3      1  seq
...................uuccGuuugucauccuaugccu......................................................................  1      1  seq
...................uucccuuugucauUcuaugccu......................................................................  4      1  seq
...................uucccuuugucaucGuaugccu......................................................................  2      1  seq
...................uuccAuuugucauccuaugccu......................................................................  7      1  seq
...................uucccuuugCcauccuaugccu......................................................................  6      1  seq
...................uucccuuuCucauccuaugccu......................................................................  1      1  seq
...................uucccuuugucauccuaAgccu......................................................................  2      1  seq
...................uucccuuugucauccuGugccu......................................................................  2      1  seq
...................Aucccuuugucauccuaugccu......................................................................  14     1  seq
...................uucccuuugucauccuauAccu......................................................................  2      1  seq
...................Cucccuuugucauccuaugccu......................................................................  12     1  seq
...................uucccuuuguAauccuaugccu......................................................................  4      1  seq
...................uucccuuugucauccuaugcAu......................................................................  3      1  seq
...................uucccuuugGcauccuaugccu......................................................................  4      1  seq
...................uucccuuugucauccuaugUcu......................................................................  6      1  seq
...................uucccuuugucauccCaugccu......................................................................  5      1  seq
...................uucccuuugucauccuaugccu......................................................................  25059  0  seq
...................uuAccuuugucauccuaugccu......................................................................  6      1  seq
...................uucccuuugAcauccuaugccu......................................................................  3      1  seq
...................uucccuuugucauccuaugccA......................................................................  937    1  seq
...................uCcccuuugucauccuaugccug.....................................................................  1      1  seq
...................uucccuuugucauccuaugccuA.....................................................................  28     1  seq
...................Gucccuuugucauccuaugccug.....................................................................  1      1  seq
...................uucccuuugucauUcuaugccug.....................................................................  1      1  seq
...................uucccuuugucauccuaugccCg.....................................................................  2      1  seq
...................uucccCuugucauccuaugccug.....................................................................  1      1  seq
...................uucccuAugucauccuaugccug.....................................................................  1      1  seq
...................uucccuuugucauccuaugccug.....................................................................  952    0  seq
...................uucccuuugucauccuaugccGg.....................................................................  13     1  seq
...................uucccuuugucauccuaugccuU.....................................................................  586    1  seq
...................uucccuuugGcauccuaugccug.....................................................................  1      1  seq
...................uuUccuuugucauccuaugccug.....................................................................  1      1  seq
...................uucccuuugucauccuaugccuC.....................................................................  14     1  seq
...................Cucccuuugucauccuaugccug.....................................................................  1      1  seq
...................uucccuuugucauccuaugccAg.....................................................................  1      1  seq
...................uucccuuugucauccuaugccugU....................................................................  207    1  seq
...................uucccuuugucauccuaugccuUa....................................................................  2      1  seq
...................uucccuuugucauccuaugccuAa....................................................................  3      1  seq
...................uucccuuugucauccuaugccuga....................................................................  5      0  seq
...................uucccuuugucauccuaugccugagaau.............................................................  1      0  seq
....................ucccCuugucauccuaugccu......................................................................  1      1  seq
....................ucccuuugucauccuaugccu......................................................................  1      0  seq
......................ccuuugucauccuaugccu......................................................................  2      0  seq
.......................cuuugucauccuaugccu......................................................................  2      0  seq
.........................................................ggcugggacggcaaagggac..................................  1      0  seq
.........................................................ggcugggacggcaaagggacg.................................  1      0  seq
.........................................................ggcugggacggcaaagggacgu................................  2      0  seq
.........................................................ggcugggacggcaaagggacguu...............................  5      0  seq
..........................................................gcugggacggcaaagggacA.................................  1      1  seq
..........................................................gcugggacggcaaagggacg.................................  7      0  seq
..........................................................gcugggacggcaaagggacgu................................  7      0  seq
..........................................................gcugggacggcaaagggacgC................................  1      1  seq
```

```
                         Mature                                              Star
guucaugugacccguggacuucccuuugucauccuaugccugagaauauaugaagggggcugggacggcaaagggacguucaacugucaucacuggcaucuucucugacca
..........................................................gcugggacggcaaagggacguA...............................    1    1    seq
..........................................................gcugggacggcaaagggacguu...............................    1    0    seq
..........................................................gcugggacggcaaagggacguuU..............................    1    1    seq
```

Provisional ID	:	mun-miR-18a
Score total	:	271.9
Score for star read(s)	:	3.9
Score for read counts	:	262.7
Score for mfe	:	0.8
Score for randfold	:	1.6
Score for cons. seed	:	3
Total read count	:	527
Mature read count	:	492
Loop read count	:	0
Star read count	:	35

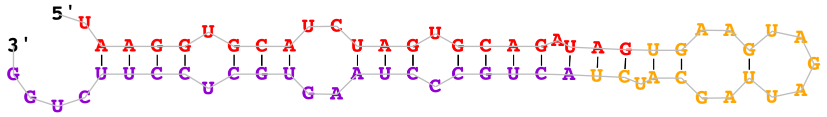

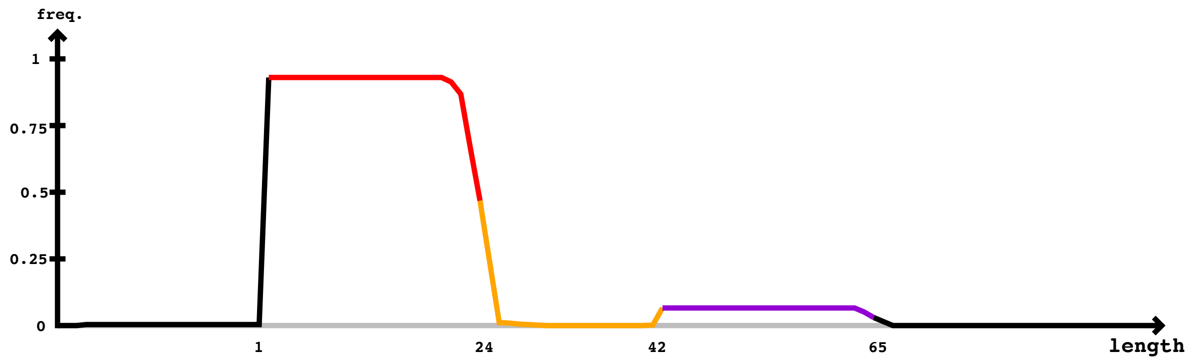

Mature **Star**

```
5'- gugucaagugcuuuuuguacuaaggugcaucuagugcagauagugaaguagauuagcaucuacugcccuaagugcuccuucuggcauaagaaguuaugucuucauccagucac  -3'  obs
    gugucaagugcuuuuuguacuaaggugcaucuagugcagauagugaaguagauuagcaucuacugcccuaagugcuccuucuggcauaagaaguuaugucuucauccagucac       exp
    ((((((.((((.....)))).((((.(((..(((.((((.(((((..(.....)..)).))))))).)))..))).)))).))))))..((((.....))))...........   reads   mm   sample
    .ugucaagugcuuuuuguac.............................................................................................   2       0    seq
    ....................uaaggugcaucuagugcag..........................................................................   8       0    seq
    ....................uaaggugcaucuaguUcag..........................................................................   1       1    seq
    ....................uaaggugcaucuagugcaga.........................................................................   24      0    seq
    ....................uaaggugcaucuagugcagaA........................................................................   1       1    seq
    ....................uaaggugcaucuagugcagau........................................................................   107     0    seq
    ....................uaaggugcaucuagugcagaAa.......................................................................   2       1    seq
    ....................uaaggugcaucuagugcagauU.......................................................................   8       1    seq
    ....................uaaggugcaucuagugcagauC.......................................................................   1       1    seq
    ....................uaaggugcaucuagugcagauG.......................................................................   45      1    seq
    ....................uaaggGgcaucuagugcagaua.......................................................................   1       1    seq
    ....................uaaggugcaucuagAgcagaua.......................................................................   1       1    seq
    ....................uaaggugcaucuagugcagaua.......................................................................   46      0    seq
    ....................uaaggugAaucuagugcagauag......................................................................   1       1    seq
    ....................uaaggugcaucuaAugcagauag......................................................................   1       1    seq
    ....................uaaggugcaucuagGgcagauag......................................................................   1       1    seq
    ....................uaaggugcaucuagugcagauaA......................................................................   3       1    seq
    ....................uaaggugcaucuagugcagauag......................................................................   162     0    seq
    ....................uaaggugcaucuagugcagauaU......................................................................   6       1    seq
    ....................uaaggugcaucuagugcagauagu.....................................................................   60      0    seq
    ....................uaaggugcaucuagugcaUauagu.....................................................................   1       1    seq
    ....................uaaggugcaucuagugcagauagA.....................................................................   7       1    seq
    ....................uaaggugcaucuagugcagauaguU....................................................................   1       1    seq
    ....................uaaggugcaucuagugcagauaguga...................................................................   2       0    seq
    ....................uaaggugcaucuagugcagauagugaa..................................................................   1       0    seq
    ....................uaaggugcaucuagugcagauagugaag.................................................................   1       0    seq
    ....................uaaggugcaucuagugcagauagugaagu................................................................   1       0    seq
    ...........................................................uacugcccuaagugcuccuucu................................   1       0    seq
    ............................................................acugcccuaagugcuccuucu................................   7       0    seq
    ............................................................acugcccuaagugcuccuucug...............................   11      0    seq
    ............................................................acugcccuaagugcuccuucugU..............................   5       1    seq
    ............................................................acugcccuaagugcuccuucugg..............................   11      0    seq
```

Provisional ID	:	mun-miR-458b
Score total	:	3.5
Score for star read(s)	:	-1.3
Score for read counts	:	-1.8
Score for mfe	:	2.1
Score for randfold	:	1.6
Score for cons. seed	:	3
Total read count	:	8
Mature read count	:	8
Loop read count	:	0
Star read count	:	0

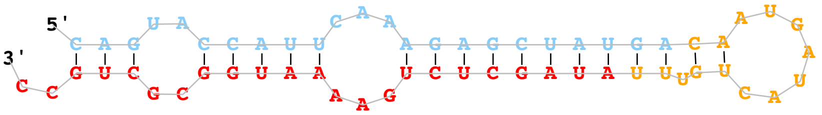

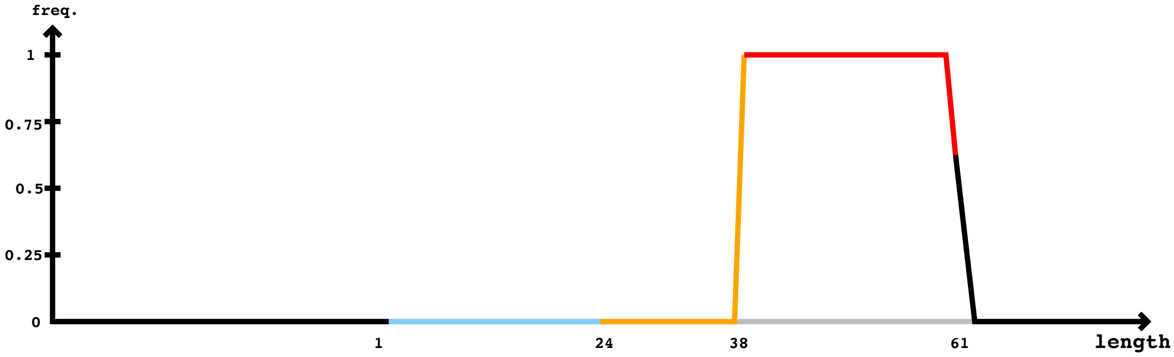

Star Mature

```
5'- caagcauaaaugcugguuuuccgguacauaugg caguaccauucaaagagcuauga caaugauacuguuu auagcucugaaaauggcgcugcc aucugcaccauggugcagc  -3'   exp
    ..........((((((....))))))...(((((((..(((((...((((((((((((.......)).))))))))))...)))))..)))))))((((((....)))))).   reads   mm   sample
    ......................................................................auagcucugaaaauggcgcugU....................   1       1    seq
    ......................................................................auagcucugaaaauggcgcugc....................   2       0    seq
    ......................................................................auagcucugaaaauggcgcugcU...................   5       1    seq
```

Provisional ID	:	mun-novel32
Score total	:	1.9
Score for star read(s)	:	-1.3
Score for read counts	:	0
Score for mfe	:	2.2
Score for randfold	:	1.6
Score for cons. seed	:	-0.6
Total read count	:	21
Mature read count	:	21
Loop read count	:	0
Star read count	:	0

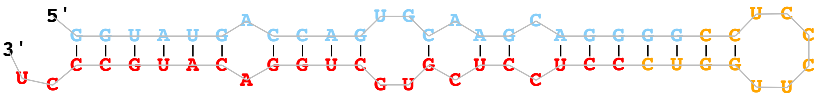

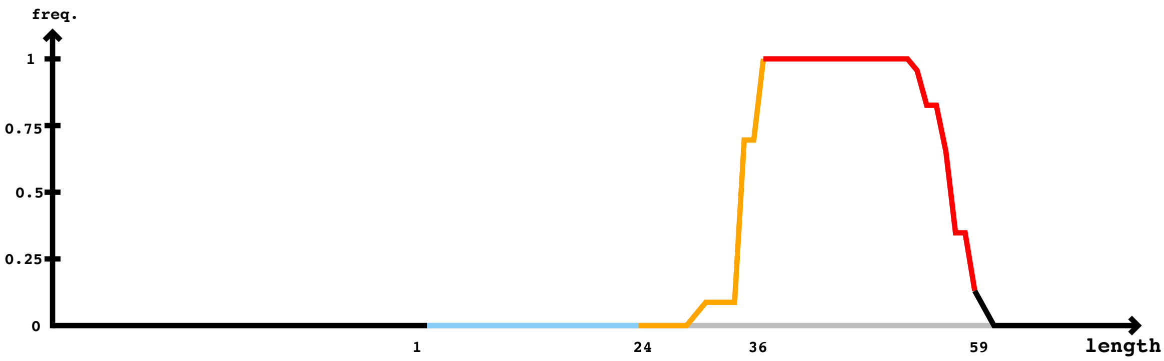

Star Mature

```
5'- ugcaagcauggaugccagcaccccggagccccugccagguaugaccagugcaagcaggggccucccuugguccccuccucgugcuggacaugcccucugcuccgcucugcag   -3'    exp
    ((((.((.(((...)))))....((((((........((((((.((((..(.((.(((((((......))))))).)).)..)))).))))))....))))))...)))).   reads     mm      sample
    ..............................................................cuuggucccuccucgugcuggac......................   1         0       seq
    ...............................................................uuggucccuccucgugcuggacU.....................   1         1       seq
    ...................................................................ucccuccucgugcuggaca.....................   1         0       seq
    ...................................................................ucccuccucgugcuggacU.....................   1         1       seq
    ...................................................................ucccuccucgugcuggacaug...................   2         0       seq
    ...................................................................ucccuccucgugcuggacauU...................   1         1       seq
    ...................................................................ucccuccucgugcuggacauC...................   1         1       seq
    ...................................................................ucccuccucgugcuggacaugU..................   3         1       seq
    ...................................................................ucccuccucgugcuggacaugA..................   3         1       seq
    ...................................................................ucccuccucgugcuggacaugccU................   2         1       seq
    .....................................................................ccuccucgugcuggacaugc..................   1         0       seq
    .....................................................................ccuccucgugcuggacaugccU................   3         1       seq
    .....................................................................ccuccucgugcuggacaugcccu...............   3         0       seq
```

Provisional ID	:	mun-novel6
Score total	:	5.6
Score for star read(s)	:	-1.3
Score for read counts	:	0
Score for mfe	:	2.3
Score for randfold	:	1.6
Score for cons. seed	:	3
Total read count	:	15
Mature read count	:	14
Loop read count	:	0
Star read count	:	1

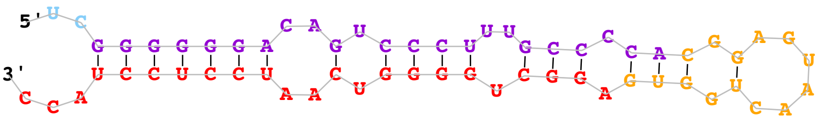

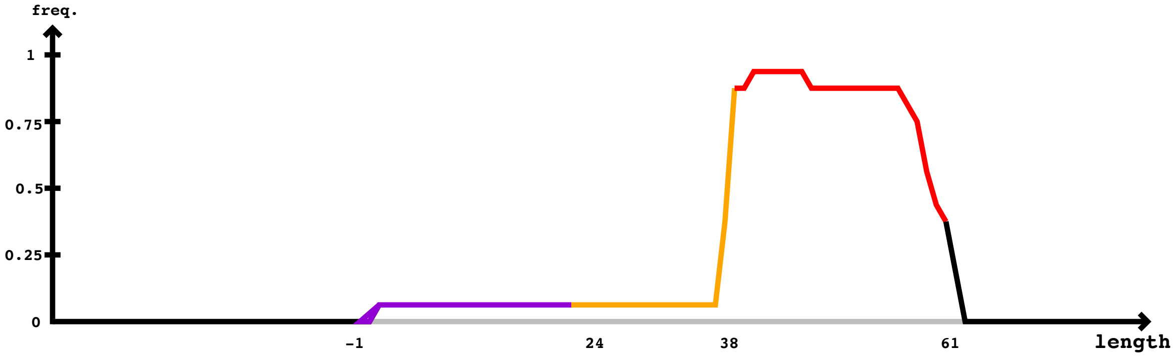

Star Mature

```
5'- accauaaggugacacugugucccuuuggggguc ggggggacagucccuuugcccca cggaguaacuggug aggcuggggucaauccuccuacc ccgugggggguccaucauccu  -3'   obs
    accauaaggugacacugugucccuuugggg ucggggggacagucccuuugcccca cggaguaacuggug aggcuggggucaauccuccuacc ccgugggggguccaucauccu        exp
    .......((((...((..(((((.((((((.(((((((..(.((((..(((.(((.(......).))).))).)))).)..)))))))))))))).))))).)))))))..   reads   mm   sample
    ..............................ggggggacagucccuuugcccca......................................................        1      0    seq
    ..................................................cacggaguaacuggugaggcuggg................................        1      0    seq
    ................................................................gaggcuggggucaauccuc.......................        1      0    seq
    ................................................................gaggcuggggucaauccucc......................        1      0    seq
    ................................................................gaggcuggggucaauccuccu.....................        2      0    seq
    ................................................................gaggcuggggucaauccuccua....................        1      0    seq
    .................................................................aggcuggggucaauccuccu.....................        1      0    seq
    .................................................................aggcuggggucaauccuccua....................        1      0    seq
    .................................................................aggcuggggucaauccuccuaU...................        1      1    seq
    .................................................................aggcuggggGcaauccuccuacc..................        1      1    seq
    .................................................................aggcuggggucaauccuccuacU..................        4      1    seq
    ...................................................................gcuggggucaauccuccuacc..................        1      0    seq
```

Provisional ID : mun-miR-10a-2
Score total : 361714.6
Score for star read(s) : 3.9
Score for read counts : 361704.7
Score for mfe : 1.3
Score for randfold : 1.6
Score for cons. seed : 3
Total read count : 709480
Mature read count : 709123
Loop read count : 0
Star read count : 357

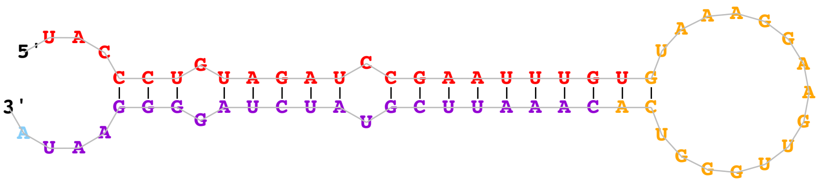

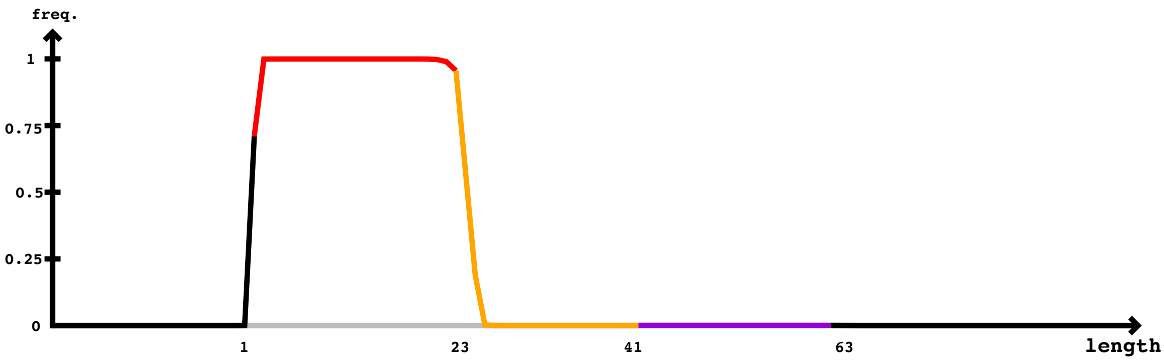

Mature **Star**

```
5'- ugcaucugucuucuauauguacccuguagauccgaauuuguguaaaggaaguugggucacaaauucguaucuaggggaauauguaguugacacaaacacuacaggucacac  -3'   obs
    ugcaucugucuucuauauguacccuguagauccgaauuuguguaaaggaaguugggucacaaauucguaucuaggggaauauguaguugacacaaacacuacaggucacac         exp
    ......((((..((((((((.((((.(((((.((((((((((...............)))))))))).))))))))).))))))))..))))...................   reads   mm   sample
    ugcaucugucuucuauaug............................................................................................   2       0    seq
    .................uCuacccuguagauccgaauuugu......................................................................   15      1    seq
    .................Gguacccuguagauccgaauuugu......................................................................   1       1    seq
    .................Cguacccuguagauccgaauuugu......................................................................   1       1    seq
    .................uCuacccuguagauccgaauuugug.....................................................................   2       1    seq
    .................uCuacccuguagauccgaauuugugu....................................................................   2       1    seq
    ..................Cuacccuguagauccgaauuu........................................................................   3       1    seq
    ..................guacccuguagauccgaauuug.......................................................................   1       0    seq
    ..................Cuacccuguagauccgaauuug.......................................................................   6       1    seq
    ..................Cuacccuguagauccgaauuugu......................................................................   154     1    seq
    ..................guacccuguagauccgaauuugu......................................................................   3       0    seq
    ..................Cuacccuguagauccgaauuugug.....................................................................   11      1    seq
    ..................Cuacccuguagauccgaauuugugu....................................................................   4       1    seq
    ...................uacccuguagauccgaaA..........................................................................   2       1    seq
    ...................uacccuguagauccgaUu..........................................................................   2       1    seq
    ...................uacccuguagaGccgaau..........................................................................   1       1    seq
    ...................uacccuguagauccgaau..........................................................................   108     0    seq
    ...................uacccuguagauccgaaG..........................................................................   1       1    seq
    ...................uGcccuguagauccgaauu.........................................................................   1       1    seq
    ...................uacAcuguagauccgaauu.........................................................................   2       1    seq
    ...................uacccugCagauccgaauu.........................................................................   2       1    seq
    ...................uacccuguagauccgaauu.........................................................................   870     0    seq
    ...................Gacccuguagauccgaauu.........................................................................   2       1    seq
    ...................uacccuguGgauccgaauu.........................................................................   1       1    seq
    ...................uacccugAagauccgaauu.........................................................................   1       1    seq
    ...................uacccuguagauUcgaauu.........................................................................   1       1    seq
    ...................uaAccuguagauccgaauu.........................................................................   1       1    seq
    ...................uaccAuguagauccgaauu.........................................................................   2       1    seq
    ...................uacccuguagauccgaauG.........................................................................   1       1    seq
    ...................uacccuguagauccgaauA.........................................................................   5       1    seq
    ...................uacccuguagauccgCauuu........................................................................   2       1    seq
    ...................uacccuguagUuccgaauuu........................................................................   2       1    seq
    ...................uacccuguagaucUgaauuu........................................................................   1       1    seq
```

```
                   Mature                                  Star
ugcaucugucuucuauauguacccuguagauccgaauuuguguaaaggaaguuggggucacaaauucguaucuaggggaauauguaguugacacaaacacuacaggucacac
...................uacccuguaAauccgaauuu.........................................................................    2       1    seq
...................uacccuguagauccgaauuu.........................................................................    4445    0    seq
...................uacccCguagauccgaauuu.........................................................................    1       1    seq
...................uacccuguagauccgaGuuu.........................................................................    1       1    seq
...................Cacccuguagauccgaauuu.........................................................................    3       1    seq
...................uacccuguagauUcgaauuu.........................................................................    4       1    seq
...................uaccGuguagauccgaauuu.........................................................................    1       1    seq
...................uacccuguagauccgaauAu.........................................................................    1       1    seq
...................uacccuguagauccgaauuA.........................................................................    42      1    seq
...................uacccuguagaCccgaauuu.........................................................................    1       1    seq
...................uacccuguagauAcgaauuu.........................................................................    1       1    seq
...................uacccuguagauccgaUuuu.........................................................................    1       1    seq
...................uacccuguagaGccgaauuu.........................................................................    1       1    seq
...................uacccuguagaucAgaauuu.........................................................................    1       1    seq
...................uacccuguagauccgaaAuu.........................................................................    1       1    seq
...................uacccuguagCuccgaauuu.........................................................................    1       1    seq
...................uacccuguagauccAaauuu.........................................................................    1       1    seq
...................uaccAuguagauccgaauuu.........................................................................    2       1    seq
...................Gacccuguagauccgaauuu.........................................................................    1       1    seq
...................uacccuguagauccgaauuG.........................................................................    3       1    seq
...................uacccuguaCauccgaauuu.........................................................................    1       1    seq
...................Aacccuguagauccgaauuu.........................................................................    2       1    seq
...................uacccuguagauccgaauGu.........................................................................    1       1    seq
...................uacccuUuagauccgaauuu.........................................................................    1       1    seq
...................uacccugAagauccgaauuu.........................................................................    2       1    seq
...................uacccuguagauccgaaAuug........................................................................    9       1    seq
...................uacccuCuagauccgaauuug........................................................................    1       1    seq
...................uacccuguUgauccgaauuug........................................................................    2       1    seq
...................uacccuguagauccgUauuug........................................................................    3       1    seq
...................uacccuguagaCccgaauuug........................................................................    1       1    seq
...................uacccuguagaGccgaauuug........................................................................    2       1    seq
...................uacccuAuagauccgaauuug........................................................................    2       1    seq
...................uacccuguagauUcgaauuug........................................................................    11      1    seq
...................uacccuguagauccgaauGug........................................................................    1       1    seq
...................uacccuguagauccUaauuug........................................................................    3       1    seq
...................uacccugAagauccgaauuug........................................................................    3       1    seq
...................uacccuguagaucUgaauuug........................................................................    2       1    seq
...................uacccugCagauccgaauuug........................................................................    5       1    seq
...................uacccuguaAauccgaauuug........................................................................    3       1    seq
...................uacccuguagGuccgaauuug........................................................................    1       1    seq
...................Gacccuguagauccgaauuug........................................................................    16      1    seq
...................uacccuguagauccgaUuuug........................................................................    1       1    seq
...................uacccuguagauccAaauuug........................................................................    1       1    seq
...................Aacccuguagauccgaauuug........................................................................    13      1    seq
...................uacccuguagauccgCauuug........................................................................    3       1    seq
...................uacccugGagauccgaauuug........................................................................    6       1    seq
...................uacccuguagaucAgaauuug........................................................................    6       1    seq
...................uaUccuguagauccgaauuug........................................................................    1       1    seq
...................uacccuguagauccgaCuuug........................................................................    3       1    seq
...................uacccuguagauccgaauCug........................................................................    2       1    seq
...................uacccuguagauccgaauuug........................................................................    18815   0    seq
...................uaccGuguagauccgaauuug........................................................................    1       1    seq
...................uacccCguagauccgaauuug........................................................................    4       1    seq
...................uaccUuguagauccgaauuug........................................................................    2       1    seq
...................uacccuguGgauccgaauuug........................................................................    2       1    seq
...................uacccuguagauccgaauuuA........................................................................    40      1    seq
...................uacAcuguagauccgaauuug........................................................................    6       1    seq
...................uaccAuguagauccgaauuug........................................................................    2       1    seq
...................uGcccuguagauccgaauuug........................................................................    2       1    seq
...................uacccGguagauccgaauuug........................................................................    1       1    seq
...................uacUcuguagauccgaauuug........................................................................    2       1    seq
...................Cacccuguagauccgaauuug........................................................................    16      1    seq
...................uaAccuguagauccgaauuug........................................................................    3       1    seq
...................uCccuguagauccgaauuug.........................................................................    1       1    seq
...................uacccuguagauAcgaauuug........................................................................    4       1    seq
...................uacccuguagauccgaaCuug........................................................................    3       1    seq
...................uacccuguagauccgaauuCg........................................................................    2       1    seq
...................uacccuguagauccgGauuug........................................................................    4       1    seq
...................uacccuguagaucGgaauuug........................................................................    1       1    seq
...................uacccuguaUauccgaauuug........................................................................    3       1    seq
```

Mature / Star			
ugcaucugucuucuauauguacccuguagauccgaauuuguguaaaggaaguugggucacaaauucguaucuaggggaauauguaguugacacaaacacuacaggucacac			
...................uacccuguagauccCaauuug...	3	1	seq
...................uacccuguagauccgaaGuug...	2	1	seq
...................uacccuguagauccgaauuuU...	55	1	seq
...................uacccuguagauccgaGuuug...	4	1	seq
...................uacccuguagauccgaauuugA..	3017	1	seq
...................uacccuCuagauccgaauuugu..	13	1	seq
...................uacccuguagauccgaaCuugu..	29	1	seq
...................uaAccuguagauccgaauuugu..	40	1	seq
...................uacGcuguagauccgaauuugu..	15	1	seq
...................uacccuguagaucUgaauuugu..	145	1	seq
...................uacccuguGgauccgaauuugu..	46	1	seq
...................uacAcuguagauccgaauuugu..	63	1	seq
...................uacccuguagauccgCauuugu..	26	1	seq
...................uacccuguagGuccgaauuugu..	42	1	seq
...................uacccuguagauccgaauAugu..	4	1	seq
...................uacccuguagauccgaGuuugu..	40	1	seq
...................uacccuguagauAcgaauuugu..	52	1	seq
...................uCccuguagauccgaauuugu..	1	1	seq
...................uacccuguagaGccgaauuugu..	33	1	seq
...................uaccGuguagauccgaauuugu..	6	1	seq
...................uacccuUuagauccgaauuugu..	69	1	seq
...................uacccuguagCuccgaauuugu..	15	1	seq
...................uaccAuguagauccgaauuugu..	52	1	seq
...................uacccGguagauccgaauuugu..	8	1	seq
...................uacccugAagauccgaauuugu..	49	1	seq
...................uacccuguagauccgaaAuugu..	145	1	seq
...................uacccuguagaucAgaauuugu..	97	1	seq
...................uacccuguagaucGgaauuugu..	11	1	seq
...................Cacccuguagauccgaauuugu..	133	1	seq
...................uacccuguagauccgGauuugu..	52	1	seq
...................uacccuguagauccgaCuuugu..	11	1	seq
...................uacccuAuagauccgaauuugu..	17	1	seq
...................uacccuguagUuccgaauuugu..	19	1	seq
...................uacccuguagauccgaauuugu..	355001	0	seq
...................uacccuguagauccgaauCugu..	19	1	seq
...................uacUcuguagauccgaauuugu..	33	1	seq
...................uacccuguagauccgaauuugC..	42	1	seq
...................uaGccuguagauccgaauuugu..	6	1	seq
...................uacccugCagauccgaauuugu..	83	1	seq
...................uacccugGagauccgaauuugu..	59	1	seq
...................uacccuguagauUcgaauuugu..	202	1	seq
...................uacccuguagauccgaauuuUu..	85	1	seq
...................uacccuguagauccgaauuuAu..	6	1	seq
...................uacccuguaAauccgaauuugu..	38	1	seq
...................uGcccuguagauccgaauuugu..	35	1	seq
...................uaUccuguagauccgaauuugu..	28	1	seq
...................uaccUuguagauccgaauuugu..	32	1	seq
...................uacccuguagauccgaaGuugu..	1	1	seq
...................uacccuguaUauccgaauuugu..	48	1	seq
...................uacccuguagauccgaauuuCu..	7	1	seq
...................uacccuguagauccgaauuGgu..	3	1	seq
...................uacccuguagaCccgaauuugu..	59	1	seq
...................uUcccuguagauccgaauuugu..	9	1	seq
...................uacccuguagauccCaauuugu..	30	1	seq
...................uacccuguCgauccgaauuugu..	6	1	seq
...................uacccAguagauccgaauuugu..	16	1	seq
...................uacccuguagauccgaauuugG..	208	1	seq
...................uacccuguagauccgUauuugu..	16	1	seq
...................uacccuguagauccgaUuuugu..	22	1	seq
...................uacccuguagauccgaauuCgu..	19	1	seq
...................uacccuguagauccAaauuugu..	44	1	seq
...................Gacccuguagauccgaauuugu..	240	1	seq
...................uacccuguaCauccgaauuugu..	10	1	seq
...................uacccuguagauccgaauGugu..	6	1	seq
...................uacccuguUgauccgaauuugu..	27	1	seq
...................uacccuguagauccUaauuugu..	56	1	seq
...................uacccCguagauccgaauuugu..	63	1	seq
...................Aacccuguagauccgaauuugu..	248	1	seq
...................uacccuguagauGcgaauuugu..	16	1	seq
...................uacccuguagauccgaauuAgu..	5	1	seq

```
                  Mature                   Star
ugcaucugucuucuauauguacccuguagauccgaauuuguguaaaggaaguugggucacaaauucguaucuaggggaauauguaguugacacaaacacuacaggucacac
...................uacccuguagauccgUauuugug.....................................................................    2      1    seq
...................uacccuguagUuccgaauuugug.....................................................................    9      1    seq
...................uacccuguagauccgaauuuguA.....................................................................    2435   1    seq
...................uacccuguagaucGgaauuugug.....................................................................    1      1    seq
...................uaAccuguagauccgaauuugug.....................................................................    7      1    seq
...................uacccuguagauccgCauuugug.....................................................................    4      1    seq
...................uacccugGagauccgaauuugug.....................................................................    16     1    seq
...................uacccuguagauGcgaauuugug.....................................................................    4      1    seq
...................uacccuguagauccgaUuuugug.....................................................................    4      1    seq
...................uacccuguagauccCaauuugug.....................................................................    5      1    seq
...................uaccGuguagauccgaauuugug.....................................................................    1      1    seq
...................uacccuguagauUcgaauuugug.....................................................................    11     1    seq
...................uacUcuguagauccgaauuugug.....................................................................    4      1    seq
...................uacccuguagauccUaauuugug.....................................................................    6      1    seq
...................uacccuguagauccgaauuugGg.....................................................................    42     1    seq
...................uacccAguagauccgaauuugug.....................................................................    1      1    seq
...................uacccuguGgauccgaauuugug.....................................................................    7      1    seq
...................uacccugAagauccgaauuugug.....................................................................    8      1    seq
...................uacccuguagauccgaauuCgug.....................................................................    6      1    seq
...................uacccuguagauccgaauuuUug.....................................................................    10     1    seq
...................uacccCguagauccgaauuugug.....................................................................    14     1    seq
...................uacccuguagGuccgaauuugug.....................................................................    8      1    seq
...................uacccuguagauccgaauuuguU.....................................................................    2069   1    seq
...................uacccuguagauccgaauuugug.....................................................................    58955  0    seq
...................uacccuguagaucAgaauuugug.....................................................................    18     1    seq
...................uacccuUuagauccgaauuugug.....................................................................    11     1    seq
...................uacccugCagauccgaauuugug.....................................................................    14     1    seq
...................uacAcuguagauccgaauuugug.....................................................................    21     1    seq
...................uacccuguagauAcgaauuugug.....................................................................    15     1    seq
...................Cacccuguagauccgaauuugug.....................................................................    33     1    seq
...................uacccuguagauccgaauCugug.....................................................................    4      1    seq
...................uacccuguagauccgGauuugug.....................................................................    5      1    seq
...................uacccuguagauccgaauuuguC.....................................................................    19     1    seq
...................uaccUuguagauccgaauuugug.....................................................................    4      1    seq
...................uacccuguagauccgaaAuugug.....................................................................    36     1    seq
...................uacGcuguagauccgaauuugug.....................................................................    1      1    seq
...................uacccuguagauccgaauuAgug.....................................................................    1      1    seq
...................uacccuguagauccgaauuugAg.....................................................................    6      1    seq
...................uacccuguagaGccgaauuugug.....................................................................    3      1    seq
...................uacccuguagaucUgaauuugug.....................................................................    7      1    seq
...................uacccuguagauccgaauuugCg.....................................................................    6      1    seq
...................uaccAuguagauccgaauuugug.....................................................................    9      1    seq
...................uacccuguaUauccgaauuugug.....................................................................    4      1    seq
...................uacccuguagauccgaauGugug.....................................................................    1      1    seq
...................uacccuguagauccgaGuuugug.....................................................................    7      1    seq
...................uacccuCuagauccgaauuugug.....................................................................    2      1    seq
...................Aacccuguagauccgaauuugug.....................................................................    39     1    seq
...................uacccuguagauccAaauuugug.....................................................................    13     1    seq
...................uGcccuguagauccgaauuugug.....................................................................    5      1    seq
...................uacccuguagauccgaauuuCug.....................................................................    1      1    seq
...................uUcccuguagauccgaauuugug.....................................................................    3      1    seq
...................uaUccuguagauccgaauuugug.....................................................................    3      1    seq
...................uaGccuguagauccgaauuugug.....................................................................    3      1    seq
...................uacccuguagCuccgaauuugug.....................................................................    1      1    seq
...................uacccuguagaCccgaauuugug.....................................................................    10     1    seq
...................Gacccuguagauccgaauuugug.....................................................................    42     1    seq
...................uacccuguaAauccgaauuugug.....................................................................    3      1    seq
...................uacccuguagauccgaauuuAug.....................................................................    14     1    seq
...................uacccuguagauccgaaGuugug.....................................................................    1      1    seq
...................uacccuAuagauccgaauuugug.....................................................................    3      1    seq
...................uacccuguUgauccgaauuugug.....................................................................    7      1    seq
...................uacccGguagauccgaauuugug.....................................................................    1      1    seq
...................uacccuguagauccgaaCuugug.....................................................................    7      1    seq
...................uacccuguagauccgCauuugugu....................................................................    1      1    seq
...................uacccuguagauccgaauuugugC....................................................................    148    1    seq
...................uacccuguUgauccgaauuugugu....................................................................    2      1    seq
...................uacccuguagauccgaauuugCgu....................................................................    8      1    seq
...................uacccuCuagauccgaauuugugu....................................................................    1      1    seq
...................uacccuguagauUcgaauuugugu....................................................................    7      1    seq
...................uacccuguagauccAaauuugugu....................................................................    1      1    seq
```

```
                   Mature                                 Star
ugcaucugucuucuauauguacccuguagauccgaauuuguguaaaggaaguuggguca caaauucguaucuaggggaauauguaguugacacaaacacuacaggucacac
...................uacccuguagauccgaauuugugA....................................................................   22293   1   seq
...................uacccuguagauccgaauuuguUu....................................................................   85      1   seq
...................uacccugGagauccgaauuugugu....................................................................   1       1   seq
...................uacccuguagauAcgaauuugugu....................................................................   6       1   seq
...................uacccuguagCuccgaauuugugu....................................................................   2       1   seq
...................uacccuguagauccgaauuugugu....................................................................   26075   0   seq
...................uaccAuguagauccgaauuugugu....................................................................   5       1   seq
...................uacccuguagauccgaUuuugugu....................................................................   2       1   seq
...................uacccuguagauccgaauuugugG....................................................................   4427    1   seq
...................uacccugAagauccgaauuugugu....................................................................   1       1   seq
...................uacccuguagauccgGauuugugu....................................................................   3       1   seq
...................uacAcuguagauccgaauuugugu....................................................................   6       1   seq
...................uacccugCagauccgaauuugugu....................................................................   6       1   seq
...................Cacccuguagauccgaauuugugu....................................................................   9       1   seq
...................uacccGguagauccgaauuugugu....................................................................   1       1   seq
...................uacccuguagauccgaaGuugugu....................................................................   2       1   seq
...................uacccuguagauGcgaauuugugu....................................................................   2       1   seq
...................uacccuguagauccgaauuugGgu....................................................................   12      1   seq
...................uacccuguGgauccgaauuugugu....................................................................   4       1   seq
...................Aacccuguagauccgaauuugugu....................................................................   17      1   seq
...................uaGccuguagauccgaauuugugu....................................................................   2       1   seq
...................uacccuguagauccgaCuuugugu....................................................................   1       1   seq
...................uacccCguagauccgaauuugugu....................................................................   3       1   seq
...................uacccuguagaCccgaauuugugu....................................................................   2       1   seq
...................uacccuguagaucAgaauuugugu....................................................................   6       1   seq
...................uacccuUuagauccgaauuugugu....................................................................   2       1   seq
...................uacccuguagauccgaauuuguAu....................................................................   159     1   seq
...................uacUcuguagauccgaauuugugu....................................................................   1       1   seq
...................uacccuguagaucUgaauuugugu....................................................................   6       1   seq
...................uacccuguagauccgaaAuugugu....................................................................   18      1   seq
...................uacccuguagauccgaauuCgugu....................................................................   2       1   seq
...................uacccuguagauccgaauuugAgu....................................................................   5       1   seq
...................uacccuguaUauccgaauuugugu....................................................................   3       1   seq
...................uacccuguagauccgaauuAgugu....................................................................   1       1   seq
...................uacccuguagauccgUauuugugu....................................................................   1       1   seq
...................uacccuguaCauccgaauuugugu....................................................................   1       1   seq
...................uacccuguagauccgaauuuguCu....................................................................   5       1   seq
...................uacccuguagauccgaauuuUugu....................................................................   8       1   seq
...................uacccuguagauccUaauuugugu....................................................................   2       1   seq
...................Gacccuguagauccgaauuugugu....................................................................   14      1   seq
...................uGcccuguagauccgaauuugugu....................................................................   2       1   seq
...................uacccuguagauccgaauCugugu....................................................................   1       1   seq
...................uacccuguagauccgaauGugugu....................................................................   1       1   seq
...................uaAccuguagauccgaauuugugu....................................................................   1       1   seq
...................uacccuguagauccgaaCuugugu....................................................................   3       1   seq
...................uacccuguagauccgaauuuguguG...................................................................   2       1   seq
...................uacccuguagaAccgaauuugugua...................................................................   39      1   seq
...................uacccuguagauccgaauuugugAa...................................................................   385     1   seq
...................uacccuguagauccgaauuugugua...................................................................   67      0   seq
...................uacccuguagauccgaauuugugCa...................................................................   3       1   seq
...................uacccuguagauccgaauuugugGa...................................................................   3       1   seq
...................uacccuguagauccgaauuuguguC...................................................................   2       1   seq
...................uacccuguagauccgaauuuguguU...................................................................   101     1   seq
...................uacccuguagaucAgaauuugugua...................................................................   1       1   seq
...................uacccuguagauccgaauuuguguaU..................................................................   7       1   seq
...................uacccuguagauccgaauuuguguUa..................................................................   3       1   seq
...................uacccuguagauccgaauuugugAaa..................................................................   38      1   seq
...................uacccuguagauccgaauuuguguaa..................................................................   15      0   seq
...................uacccuguagaAccgaauuuguguaa..................................................................   5       1   seq
...................uacccuguagauccgaauuuguguaaa.................................................................   3       0   seq
...................uacccuguagauccgaauuuguguaaag................................................................   3       0   seq
...................uacccuguagauccgaauuuguguaaagg...............................................................   8       0   seq
...................uacccuguagauccgaauuuguguaaagA...............................................................   1       1   seq
...................uacccuguagauccgaauuuguguaaaggU..............................................................   5       1   seq
...................uacccuguagauccgaauuuguguaaagga..............................................................   3       0   seq
...................uacccuguagauccgaauuuguguaaaggaa.............................................................   2       0   seq
...................uacccuguagauccgaauuuguguaaaggaU.............................................................   4       1   seq
...................uacccuguagauccgaauuuguguaaaggaag............................................................   2       0   seq
...................uacccuguagauccgaauuuguguaaaggaagu...........................................................   3       0   seq
...................uacccuguagauccgaauuuguguaaaggaaguugU........................................................   1       1   seq
```

```
                   Mature                                      Star
ugcaucugucuucuauaugacccuguagauccgaauuuguguaaaggaaguuggguca caaauucguaucuaggggaauauguaguugacacaaacacuacaggucacac
```

```
ugcaucugucuucuauauguacccuguagauccgaauuuguguaaaggaaguugggucacaaauucguaucuaggggaauauguaguugacacaaacacuacaggucacac
....................acccuguagauccgaauu.........................................................................  247     0   seq
....................acccuguagauUcgaauu.........................................................................  1       1   seq
....................acccuguagauccgaauA.........................................................................  1       1   seq
....................Gcccuguagauccgaauuu........................................................................  1       1   seq
....................acccuguagauUcgaauuu........................................................................  2       1   seq
....................acccuguagUuccgaauuu........................................................................  1       1   seq
....................acccuguagauccgaauuu........................................................................  1380    0   seq
....................acccuguagauccgaauuA........................................................................  21      1   seq
....................accUuguagauccgaauuu........................................................................  1       1   seq
....................acccugAagauccgaauuu........................................................................  1       1   seq
....................Ccccuguagauccgaauuu........................................................................  1       1   seq
....................acccuguaUauccgaauuu........................................................................  1       1   seq
....................acccugGagauccgaauuu........................................................................  1       1   seq
....................acAcuguagauccgaauuu........................................................................  1       1   seq
....................acccuguagaucUgaauuu........................................................................  2       1   seq
....................acccuguagauccgaGuuu........................................................................  1       1   seq
....................acccuguagGuccgaauuug.......................................................................  1       1   seq
....................acccuguagauccgaauuug.......................................................................  4827    0   seq
....................acccuguagauccgaauuuC.......................................................................  1       1   seq
....................acccuguagauccgaUuuug.......................................................................  1       1   seq
....................acccuguagauccgaaCuug.......................................................................  1       1   seq
....................acccuguagauccgaauCug.......................................................................  2       1   seq
....................acccuAuagauccgaauuug.......................................................................  1       1   seq
....................acccuguagauccgaauuuU.......................................................................  13      1   seq
....................acccuguagauUcgaauuug.......................................................................  12      1   seq
....................aAccuguagauccgaauuug.......................................................................  3       1   seq
....................Gcccuguagauccgaauuug.......................................................................  3       1   seq
....................acccuguagaucUgaauuug.......................................................................  4       1   seq
....................acccuguagauccgaauuuA.......................................................................  14      1   seq
....................acccuguagauccgGauuug.......................................................................  2       1   seq
....................acccugAagauccgaauuug.......................................................................  1       1   seq
....................acUcuguagauccgaauuug.......................................................................  1       1   seq
....................acccugCagauccgaauuug.......................................................................  4       1   seq
....................Ucccuguagauccgaauuug.......................................................................  2       1   seq
....................acccugGagauccgaauuug.......................................................................  2       1   seq
....................acccuguagauccgUauuug.......................................................................  1       1   seq
....................acccAguagauccgaauuug.......................................................................  1       1   seq
....................acccuguaUauccgaauuug.......................................................................  1       1   seq
....................acccuguagaucAgaauuug.......................................................................  4       1   seq
....................acAcuguagauccgaauuug.......................................................................  1       1   seq
....................acccCguagauccgaauuug.......................................................................  1       1   seq
....................acccuguagauccgUauuugu......................................................................  10      1   seq
....................acccuguagaucGgaauuugu......................................................................  7       1   seq
....................acccuCuagauccgaauuugu......................................................................  2       1   seq
....................acccCguagauccgaauuugu......................................................................  20      1   seq
....................acccuguaAauccgaauuugu......................................................................  3       1   seq
....................Gcccuguagauccgaauuugu......................................................................  50      1   seq
....................acccuguagauccgaauCugu......................................................................  4       1   seq
....................acccugCagauccgaauuugu......................................................................  11      1   seq
....................acccuguagauccUaauuugu......................................................................  6       1   seq
....................acccuguagauccgaauuugu......................................................................  64468   0   seq
....................Ccccuguagauccgaauuugu......................................................................  10      1   seq
....................acccuguagauccgaauAugu......................................................................  3       1   seq
....................acccuguGgauccgaauuugu......................................................................  8       1   seq
....................acccuguagauccgaauuCgu......................................................................  7       1   seq
....................acccuguagauccgaauuuUu......................................................................  17      1   seq
....................acccuguagauccgCauuugu......................................................................  8       1   seq
....................acccuguagauccgaGuuugu......................................................................  7       1   seq
....................aAccuguagauccgaauuugu......................................................................  8       1   seq
....................acccuguagauccgaauuugC......................................................................  12      1   seq
....................acccuguagauGcgaauuugu......................................................................  1       1   seq
....................acccuAuagauccgaauuugu......................................................................  6       1   seq
....................acccuguagauccgaauuugA......................................................................  1068    1   seq
....................acAcuguagauccgaauuugu......................................................................  8       1   seq
....................acccuguagaucUgaauuugu......................................................................  58      1   seq
....................acccuguagauUcgaauuugu......................................................................  112     1   seq
....................accUuguagauccgaauuugu......................................................................  3       1   seq
....................acccuguagauccgaaCuugu......................................................................  7       1   seq
....................acccuUuagauccgaauuugu......................................................................  10      1   seq
....................acccuguagauccgaauGugu......................................................................  1       1   seq
```

```
                   Mature                 Star
ugcaucugucuucuauauguacccuguagauccgaauuuguguaaaggaaguuggguca caaauucguaucuaggggaauauguaguugacacaaacacuacaggucacac
....................acccuguagauccgGauuugu......................................................................   14      1   seq
....................aGccuguagauccgaauuugu......................................................................   2       1   seq
....................acccuguUgauccgaauuugu......................................................................   5       1   seq
....................acccuguaCauccgaauuugu......................................................................   3       1   seq
....................acccuguagaGccgaauuugu......................................................................   2       1   seq
....................acccuguagauccgaUuuugu......................................................................   4       1   seq
....................aUccuguagauccgaauuugu......................................................................   23      1   seq
....................acccAguagauccgaauuugu......................................................................   5       1   seq
....................acccuguagauccgaCuuugu......................................................................   1       1   seq
....................Ucccuguagauccgaauuugu......................................................................   6       1   seq
....................acccuguaUauccgaauuugu......................................................................   7       1   seq
....................acccuguagauccCaauuugu......................................................................   2       1   seq
....................acccuguagauccgaaAuugu......................................................................   10      1   seq
....................acccuguagauccgaauuugG......................................................................   64      1   seq
....................acccuguagauccgaaGuugu......................................................................   1       1   seq
....................accAuguagauccgaauuugu......................................................................   16      1   seq
....................acccuguagaCccgaauuugu......................................................................   6       1   seq
....................acccuguagauccgaauuuCu......................................................................   2       1   seq
....................acccuguagGuccgaauuugu......................................................................   6       1   seq
....................acccugAagauccgaauuugu......................................................................   11      1   seq
....................acccGguagauccgaauuugu......................................................................   1       1   seq
....................acccuguagauccAaauuugu......................................................................   4       1   seq
....................acccuguagUuccgaauuugu......................................................................   2       1   seq
....................acccugGagauccgaauuugu......................................................................   13      1   seq
....................acccuguagCuccgaauuugu......................................................................   1       1   seq
....................acUcuguagauccgaauuugu......................................................................   14      1   seq
....................acccuguagauAcgaauuugu......................................................................   7       1   seq
....................acccuguagauccgaauuuAu......................................................................   5       1   seq
....................acccuguagauccgaauuGgu......................................................................   2       1   seq
....................acccuguagaucAgaauuugu......................................................................   21      1   seq
....................acccugCagauccgaauuugug.....................................................................   12      1   seq
....................acccuguagauccgaaGuugug.....................................................................   2       1   seq
....................acccuguaCauccgaauuugug.....................................................................   1       1   seq
....................Gcccuguagauccgaauuugug.....................................................................   39      1   seq
....................acccuguagauccgaGuuugug.....................................................................   5       1   seq
....................acccuguUgauccgaauuugug.....................................................................   7       1   seq
....................acccuguagauccgaauGugug.....................................................................   3       1   seq
....................acccuguagaCccgaauuugug.....................................................................   6       1   seq
....................acccuguaAauccgaauuugug.....................................................................   1       1   seq
....................acccuguagauccgGauuugug.....................................................................   7       1   seq
....................acccuguagauccgaaAuugug.....................................................................   27      1   seq
....................acccuUuagauccgaauuugug.....................................................................   7       1   seq
....................acccuguagauGcgaauuugug.....................................................................   2       1   seq
....................Ucccuguagauccgaauuugug.....................................................................   8       1   seq
....................acccuguagauccgaauuCgug.....................................................................   4       1   seq
....................acccCguagauccgaauuugug.....................................................................   11      1   seq
....................acccuguGgauccgaauuugug.....................................................................   8       1   seq
....................acccuguagauccUaauuugug.....................................................................   6       1   seq
....................acccuguagauccAaauuugug.....................................................................   4       1   seq
....................accGuguagauccgaauuugug.....................................................................   1       1   seq
....................acccugAagauccgaauuugug.....................................................................   5       1   seq
....................acccuguagaGccgaauuugug.....................................................................   1       1   seq
....................acccAguagauccgaauuugug.....................................................................   2       1   seq
....................accUuguagauccgaauuugug.....................................................................   3       1   seq
....................acccuguagauccgaauuugAg.....................................................................   7       1   seq
....................aAccuguagauccgaauuugug.....................................................................   7       1   seq
....................acccuguagaucUgaauuugug.....................................................................   8       1   seq
....................acccuguagaucAgaauuugug.....................................................................   13      1   seq
....................acAcuguagauccgaauuugug.....................................................................   8       1   seq
....................acccuguagauccgaaCuugug.....................................................................   6       1   seq
....................acccuguagUuccgaauuugug.....................................................................   2       1   seq
....................acccuguagauccgaauuuUug.....................................................................   13      1   seq
....................acccuguagauccgaauuuAug.....................................................................   3       1   seq
....................acccuguagauccgaauuuguU.....................................................................   213     1   seq
....................acccuguagauccgaauuugCg.....................................................................   8       1   seq
....................acccuguagauccgaauuuguA.....................................................................   789     1   seq
....................acccuguagGuccgaauuugug.....................................................................   3       1   seq
....................acccGguagauccgaauuugug.....................................................................   1       1   seq
....................acccuguagauccgaauuuguC.....................................................................   3       1   seq
....................acccuguagauccgaauuugug.....................................................................   50486   0   seq
```

Mature Star

ugcaucugucuucuauauguacccuguagauccgaauuuguguaaaggaaguugggucacaaauucguaucuaggggaauauguaguugacacaaacacuacaggucacac			
....................acccuguagauccgaUuuugug...	6	1	seq
....................acccuguaUauccgaauuugug...	9	1	seq
....................acccuguagauccgaauuAgug...	1	1	seq
....................acccuguagauccCaauuugug...	3	1	seq
....................acGcuguagauccgaauuugug...	1	1	seq
....................acccuguagauccgCauuugug...	2	1	seq
....................acccuguagauccgUauuugug...	2	1	seq
....................acccuAuagauccgaauuugug...	8	1	seq
....................acccuguagauAcgaauuugug...	10	1	seq
....................acccugGagauccgaauuugug...	14	1	seq
....................acccuguagauUcgaauuugug...	27	1	seq
....................aUccuguagauccgaauuugug...	1	1	seq
....................acccuguagauccgaauuugGg...	32	1	seq
....................accAuguagauccgaauuugug...	6	1	seq
....................acccuguagauccgaauAugug...	2	1	seq
....................Ccccuguagauccgaauuugug...	3	1	seq
....................acccuguagauccgaauCugug...	6	1	seq
....................acccuguagauccgaauuuCug...	2	1	seq
....................accUuguagauccgaauuugugu..	1	1	seq
....................acccuguagauccgaauuugugA..	52446	1	seq
....................acccuguagauccUaauuugugu..	1	1	seq
....................acccuguagauccgaauuuguCu..	1	1	seq
....................acccuguagaucAgaauuugugu..	3	1	seq
....................acccuguagauccgaauuuguUu..	7	1	seq
....................acccuguagauccgaGuuugugu..	1	1	seq
....................acccuguagauccgGauuugugu..	1	1	seq
....................acccuguagauccgaauuugugC..	103	1	seq
....................acccuguagauccgaauuuUugu..	5	1	seq
....................acccuguagaCccgaauuugugu..	3	1	seq
....................Ccccuguagauccgaauuugugu..	2	1	seq
....................accGuguagauccgaauuugugu..	3	1	seq
....................acccugGagauccgaauuugugu..	2	1	seq
....................acccuguagauccgaauuugugG..	9122	1	seq
....................acccuguagauccgCauuugugu..	2	1	seq
....................acccuguagauGcgaauuugugu..	1	1	seq
....................acccuguagaGccgaauuugugu..	1	1	seq
....................acccuguaUauccgaauuugugu..	2	1	seq
....................acccuguagauccAaauuugugu..	1	1	seq
....................acccuguagauccgaaAuugugu..	7	1	seq
....................acccuCuagauccgaauuugugu..	1	1	seq
....................aAccuguagauccgaauuugugu..	3	1	seq
....................acccuUuagauccgaauuugugu..	1	1	seq
....................acccuguaCauccgaauuugugu..	2	1	seq
....................Gcccuguagauccgaauuugugu..	11	1	seq
....................acGcuguagauccgaauuugugu..	1	1	seq
....................acccuguagauccCaauuugugu..	1	1	seq
....................acccuguagauccgaaGuugugu..	1	1	seq
....................acccuguagauccgaUuuugugu..	1	1	seq
....................acccuguagauccgaauuugugu..	17120	0	seq
....................acccugCagauccgaauuugugu..	2	1	seq
....................Ucccuguagauccgaauuugugu..	2	1	seq
....................acccuguagauUcgaauuugugu..	20	1	seq
....................acccuguagauAcgaauuugugu..	3	1	seq
....................acccuguagauccgaauCugugu..	2	1	seq
....................acccuguagauccgaauuugCgu..	3	1	seq
....................aUccuguagauccgaauuugugu..	5	1	seq
....................acccuguaAauccgaauuugugu..	2	1	seq
....................acccuguagauccgaauuugAgu..	3	1	seq
....................acccuguagauccgaauuuAugu..	1	1	seq
....................acccuguagUuccgaauuugugu..	1	1	seq
....................acccuguagaucUgaauuugugu..	9	1	seq
....................acccuguagauccgaauuugGgu..	10	1	seq
....................acccuguagauccgaauuuguAu..	50	1	seq
....................accAuguagauccgaauuugugu..	2	1	seq
....................acccuguagauccgaauuugugCa...	2	1	seq
....................acccuguagauccgaauuuguguG...	3	1	seq
....................acccuguagauccgaauuugugua...	57	0	seq
....................acccuguagauccgaauuugugAa...	1241	1	seq
....................acccuguagauccgaauuuguguC...	2	1	seq
....................acccuguagauccgaauuuguCua...	1	1	seq

Mature **Star**

```
ugcaucugucuucuauaugcuacccuguagauccgaauuuguguaaaggaaguuggguca caaauucguaucuaggggaauauguaguugacacaaacacuacaggucacac
```

```
ugcaucugucuucuauaug uacccuguagauccgaauuugu guaaaggaaguuggguca caaauucguaucuaggggaau auguaguugacacaaacacuacaggucacac
```

```
....................acccuguagauccgaauuuguguU...................................................................  27  1  seq
....................acccuguagauccgaauuugugGa...................................................................  23  1  seq
....................acccuguagaAccgaauuugugua...................................................................  13  1  seq
....................acccuguagauccgaauuuguguaa..................................................................  5  0  seq
....................acccuguagauccgaauuugugAaa..................................................................  69  1  seq
....................acccuguagauccgaauuugugGaa..................................................................  1  1  seq
....................acccuguagaAccgaauuuguguaa..................................................................  2  1  seq
....................acccuguagauccgaauuuguguaU..................................................................  4  1  seq
....................acccuguagauccgaauuuguguaaag................................................................  1  0  seq
.....................Accuguagauccgaauuugu......................................................................  1  1  seq
.....................cccuguagauccgaauuugu......................................................................  23  0  seq
.....................cccuguagauccgaauCugug.....................................................................  1  1  seq
.....................cccuguagauccgaauuugug.....................................................................  9  0  seq
.....................Accuguagauccgaauuugugu....................................................................  1  1  seq
.....................cccuguagauccgaauuugugu....................................................................  14  0  seq
.....................cccuguagauccgaauuugugA....................................................................  12  1  seq
.....................cccuguagauccgaauuugugG....................................................................  5  1  seq
.....................cccuguagauccgaauuugugAa...................................................................  1  1  seq
......................ccuguagauccgaauuugu......................................................................  15  0  seq
......................ccuguagauccgaauuugug.....................................................................  6  0  seq
......................ccuguagauccgaauuugugA....................................................................  4  1  seq
......................ccuguagauccgaauuugugu....................................................................  3  0  seq
.......................cuguagauccgaauuugu......................................................................  2  0  seq
.......................cuguagauccgaauuugug.....................................................................  1  0  seq
.......................cuguagauccgaauuugugu....................................................................  1  0  seq
........................uguagauccgaauuugug.....................................................................  4  0  seq
........................uguagauccgaauuugugG....................................................................  2  1  seq
........................uguagauccgaauuugugu....................................................................  1  0  seq
........................uguagauccgaauuugugA....................................................................  1  1  seq
.........................guagauccgaaCuugugu....................................................................  3  1  seq
..........................................................acaaauucguaucuagggga.................................  1  0  seq
..........................................................acaaauucguaucuaggggaUu...............................  1  1  seq
..........................................................acaaauucguaucuaggggaau...............................  3  0  seq
..........................................................acaaauucguaucuaggggaauU..............................  1  1  seq
...........................................................caaauucguaucuagggA..................................  1  1  seq
...........................................................caaauucguaucuagggga.................................  12  0  seq
...........................................................caaauucguaucuaggggaU................................  4  1  seq
...........................................................caaauucguaucuaggggaa................................  12  0  seq
...........................................................caaauucguaucuaggggaC................................  1  1  seq
...........................................................caaauucguaucuaggggaCu...............................  2  1  seq
...........................................................caaauucUuaucuaggggaau...............................  1  1  seq
...........................................................caaauucguaucuaggggaaA...............................  15  1  seq
...........................................................caaauucguaucuaggggaUu...............................  1  1  seq
...........................................................caaauucguaucuaggggaau...............................  196  0  seq
...........................................................caaauucguaucuaggggaauU..............................  64  1  seq
...........................................................caaauucguaucuaggggaaAa..............................  2  1  seq
...........................................................caaauucguaucuaggggaaua..............................  3  0  seq
...........................................................caaauucguaucuaggggaauau.............................  1  0  seq
...........................................................caaauucguaucuaggggaauUu.............................  4  1  seq
............................................................aaauucguaucuaggggaa................................  1  0  seq
............................................................aaauucguaucuaggggaau...............................  24  0  seq
............................................................aaauucguaucuaggggaaua..............................  3  0  seq
............................................................aaauucguaucuaggggaauU..............................  1  1  seq
............................................................aaauucguaucuaggggaauUu.............................  1  1  seq
.............................................................aauucguaucuaggggaau...............................  1  0  seq
.............................................................aauucguaucuaggggaauUu.............................  1  1  seq
```

Provisional ID	:	mun-let-7a-4
Score total	:	1074.4
Score for star read(s)	:	3.9
Score for read counts	:	1063.6
Score for mfe	:	2.2
Score for randfold	:	1.6
Score for cons. seed	:	3
Total read count	:	2098
Mature read count	:	1962
Loop read count	:	0
Star read count	:	136

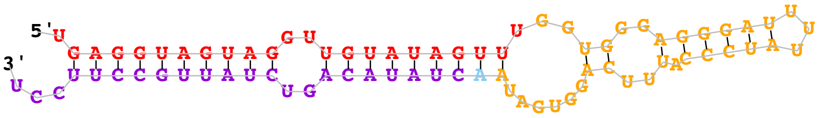

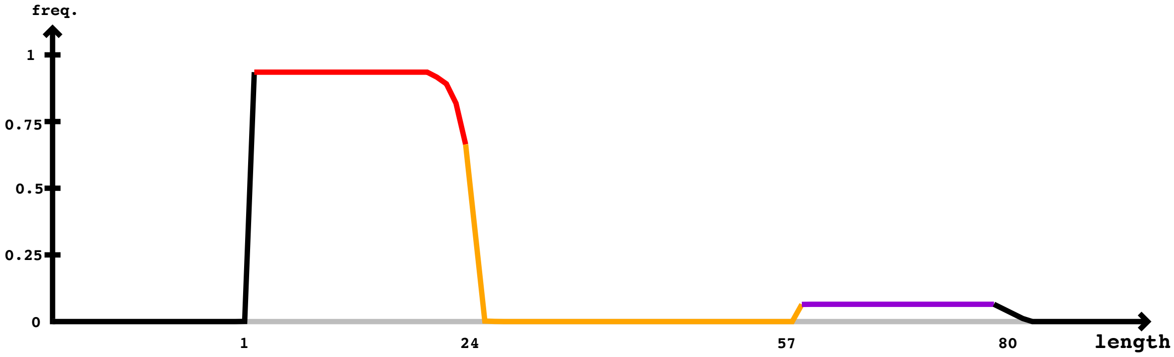

Mature **Star**

```
5'- cuguacugcucuguggaggugagguaguagguuguauaguuuggugggagggauuuuaucccauuucaggugauaacuauacagucuauugccuuccuuaaagagcagcaaa  -3'   obs
    cuguacugcucuguggaggugagguaguagguuguauaguuuggugggagggauuuuaucccauuucaggugauaacuauacagucuauugccuuccuuaaagagcagcaaa         exp
    .....(((((((...((((.((((((((((..(((((((((...((..((((((...))))).)..))......)))))))))..))))))))))))))..)))))))....   reads   mm   sample
    ..................gugagguaguagguugAauagu........................................................................   1   1   seq
    ...................ugaggGaguagguuguaua..........................................................................   1   1   seq
    ...................ugagguaguagguuguauU..........................................................................   37   1   seq
    ...................ugagguaguagguuguaAa..........................................................................   1   1   seq
    ...................ugagguaguagguuguauaA.........................................................................   27   1   seq
    ...................ugagguaguagguAguauag.........................................................................   1   1   seq
    ...................ugagguagGagguuguauag.........................................................................   1   1   seq
    ...................ugaggGaguagguuguauag.........................................................................   3   1   seq
    ...................ugagguaguagguuguauaU.........................................................................   17   1   seq
    ...................ugagguGguagguuguauag.........................................................................   1   1   seq
    ...................ugagguaguagguuguauUg.........................................................................   1   1   seq
    ...................ugaggAaguagguuguauag.........................................................................   2   1   seq
    ...................ugagguaguaggAuguauagu........................................................................   1   1   seq
    ...................ugagguaguaggGuguauagu........................................................................   2   1   seq
    ...................ugaggCaguagguuguauagu........................................................................   8   1   seq
    ...................ugagguagGagguuguauagu........................................................................   1   1   seq
    ...................ugagguaguagguuguauagA........................................................................   62   1   seq
    ...................ugaggAaguagguuguauagu........................................................................   9   1   seq
    ...................ugagguagAagguuguauagu........................................................................   1   1   seq
    ...................ugaggGaguagguuguauagu........................................................................   14   1   seq
    ...................ugagguaguagguuguaCagu........................................................................   1   1   seq
    ...................ugagguUguagguuguauagu........................................................................   1   1   seq
    ...................ugagguaguagguuguauaUu........................................................................   8   1   seq
    ...................ugagguaCuagguuguauagu........................................................................   1   1   seq
    ...................ugagguaguagguugAauagu........................................................................   2   1   seq
    ...................ugagguaguagguuguauaAu........................................................................   4   1   seq
    ...................Ggagguaguagguuguauagu........................................................................   6   1   seq
    ...................ugagguaguagguAguauagu........................................................................   1   1   seq
    ...................ugagguaguaAguuguauagu........................................................................   1   1   seq
    ...................uCagguaguagguuguauagu........................................................................   1   1   seq
    ...................ugagguaguagguuguGuagu........................................................................   2   1   seq
    ...................Cgagguaguagguuguauagu........................................................................   2   1   seq
    ...................ugagguaguagguuCuauagu........................................................................   2   1   seq
```

Mature **Star**

```
cuguacugcucuguggaggugagguaguagguuguauaguuugguggggagggauuuuaucccauuucaggugauaacuauacagucuauugccuuccuuaaagagcagcaaa
...................ugagUuaguagguuguauagu.........................................................................	1	1	seq
...................ugagguaguaUguuguauagu.........................................................................	3	1	seq
...................uUagguaguagguuguauagu.........................................................................	3	1	seq
...................ugaUguaguagguuguauagu.........................................................................	1	1	seq
...................ugagguaguaggCuguauagu.........................................................................	1	1	seq
...................ugagguaguagguuguaAagu.........................................................................	1	1	seq
...................ugUgguaguagguuguauagu.........................................................................	1	1	seq
...................ugGgguaguagguuguauagu.........................................................................	2	1	seq
...................ugagguaguagguuguauagC.........................................................................	2	1	seq
...................ugagguaguagguuUuauagu.........................................................................	1	1	seq
...................Agagguaguagguuguauagu.........................................................................	1	1	seq
...................ugagguagCagguuguauagu.........................................................................	2	1	seq
...................ugagguaguagguuguauagG.........................................................................	2	1	seq
...................ugagguaguUgguuguauagu.........................................................................	1	1	seq
...................ugagguaguGgguuguauaguu........................................................................	6	1	seq
...................ugagguaguagguuguaAaguu........................................................................	2	1	seq
...................uUagguaguagguuguauaguu........................................................................	5	1	seq
...................ugagCuaguagguuguauaguu........................................................................	1	1	seq
...................ugagguaguagguuguauagGu........................................................................	1	1	seq
...................ugaCguaguagguuguauaguu........................................................................	1	1	seq
...................ugCgguaguagguuguauaguu........................................................................	3	1	seq
...................ugagUuaguagguuguauaguu........................................................................	4	1	seq
...................ugagguaguagguuguauaAuu........................................................................	1	1	seq
...................ugagguagAagguuguauaguu........................................................................	3	1	seq
...................ugagguaguagguuguaCaguu........................................................................	11	1	seq
...................ugagguaguagguuguGuaguu........................................................................	2	1	seq
...................ugagguaguaggGuguauaguu........................................................................	14	1	seq
...................ugagguaguagguuguaGaguu........................................................................	1	1	seq
...................ugagguaguagguuUuauaguu........................................................................	5	1	seq
...................ugagguaCuagguuguauaguu........................................................................	1	1	seq
...................ugagguaguCgguuguauaguu........................................................................	1	1	seq
...................ugagguaguagguuAuauaguu........................................................................	2	1	seq
...................ugaggGaguagguuguauaguu........................................................................	43	1	seq
...................ugagguagCagguuguauaguu........................................................................	6	1	seq
...................ugagguaguagguCguauaguu........................................................................	3	1	seq
...................ugagguaguagguuguauaCuu........................................................................	2	1	seq
...................ugagguaguagguuguUuaguu........................................................................	2	1	seq
...................ugagguagGagguuguauaguu........................................................................	9	1	seq
...................Ggagguaguagguuguauaguu........................................................................	17	1	seq
...................ugaUguaguagguuguauaguu........................................................................	5	1	seq
...................ugagguaguagguuguauagAu........................................................................	3	1	seq
...................ugagguaguUgguuguauaguu........................................................................	2	1	seq
...................ugagguaguagguuguauCguu........................................................................	1	1	seq
...................uAagguaguagguuguauaguu........................................................................	2	1	seq
...................ugagguaguagguuCuauaguu........................................................................	3	1	seq
...................Agagguaguagguuguauaguu........................................................................	18	1	seq
...................ugagguaguagguugCauaguu........................................................................	5	1	seq
...................ugagguaguagCuuguauaguu........................................................................	6	1	seq
...................ugagguaguagguugGauaguu........................................................................	3	1	seq
...................ugagguaguaggCuguauaguu........................................................................	11	1	seq
...................ugagguaguaUguuguauaguu........................................................................	2	1	seq
...................ugaggCaguagguuguauaguu........................................................................	23	1	seq
...................ugagguaguagguuguauaguC........................................................................	3	1	seq
...................ugagguaguaggAuguauaguu........................................................................	4	1	seq
...................ugaggAaguagguuguauaguu........................................................................	32	1	seq
...................ugGgguaguagguuguauaguu........................................................................	5	1	seq
...................ugagguGguagguuguauaguu........................................................................	10	1	seq
...................ugagguUguagguuguauaguu........................................................................	1	1	seq
...................ugagguaguagguuguauaUuu........................................................................	7	1	seq
...................ugagguaguagguuguauagCu........................................................................	4	1	seq
...................ugagguaUuagguuguauaguu........................................................................	3	1	seq
...................Cgagguaguagguuguauaguu........................................................................	13	1	seq
...................ugagguaguagguugAauaguu........................................................................	1	1	seq
...................ugagguaguaCguuguauaguu........................................................................	3	1	seq
...................ugUgguaguagguuguauaguu........................................................................	2	1	seq
...................ugagguaguagguuguauaguG........................................................................	5	1	seq
...................ugagguaguagguuguauaguAu.......................................................................	28	1	seq
...................ugagguaguagguuguauaguCu.......................................................................	5	1	seq
...................ugagguaguagguuguauaguuG.......................................................................	79	1	seq
...................ugGgguaguagguuguauaguuu.......................................................................	1	1	seq
```

Mature **Star**

```
cuguacugcucuguggaggugagguaguagguuguauaguuuggugggagggauuuuaucccauuucaggugauaacuauacagucuauugccuuccuuaaagagcagcaaa
...................ugagguaguagguuguauagAuu......................................................................    2     1   seq
...................uAagguaguagguuguauaguuu......................................................................    1     1   seq
...................ugaAguaguagguuguauaguuu......................................................................    1     1   seq
...................Agagguaguagguuguauaguuu......................................................................    3     1   seq
...................ugagguaguagguugCauaguuu......................................................................    1     1   seq
...................Cgagguaguagguuguauaguuu......................................................................    1     1   seq
...................ugagguaguaggGuguauaguuu......................................................................    4     1   seq
...................ugagguagAagguuguauaguuu......................................................................    2     1   seq
...................ugagguaguagguuguauUguuu......................................................................    1     1   seq
...................ugaggGaguagguuguauaguuu......................................................................    14    1   seq
...................ugagguagCagguuguauaguuu......................................................................    2     1   seq
...................ugagguaguGgguuguauaguuu......................................................................    2     1   seq
...................ugagguaguagguuguauagCuu......................................................................    3     1   seq
...................ugaUguaguagguuguauaguuu......................................................................    5     1   seq
...................ugagguaguagguuguauaguuC......................................................................    50    1   seq
...................ugagguaguagguuUuauaguuu......................................................................    2     1   seq
...................ugagguaguagguCguauaguuu......................................................................    1     1   seq
...................ugagguaguagguuguauaguGu......................................................................    3     1   seq
...................ugagguagGagguuguauaguuu......................................................................    1     1   seq
...................ugagguGguagguuguauaguuu......................................................................    4     1   seq
...................ugaggAaguagguuguauaguuu......................................................................    10    1   seq
...................ugagguaguaUguuguauaguuu......................................................................    2     1   seq
...................ugagguaguagguuCuauaguuu......................................................................    3     1   seq
...................ugagUuaguagguuguauaguuu......................................................................    1     1   seq
...................ugaggCaguagguuguauaguuu......................................................................    6     1   seq
...................ugagguaguagguuguauaguuA......................................................................    1139  1   seq
...................ugagguaguagguGguauaguuu......................................................................    1     1   seq
...................ugagguaguagguuguauaUuuu......................................................................    1     1   seq
...................ugagguaguagguuguauCguuu......................................................................    2     1   seq
...................Ggagguaguagguuguauaguuu......................................................................    3     1   seq
...................ugagguaguagAuuguauaguuug.....................................................................    1     1   seq
...................ugagguaguagguuguauaguuuC.....................................................................    4     1   seq
...................ugagguaguagguuguauGguuug.....................................................................    1     1   seq
...................ugagguaguagAuuguauaguuugg....................................................................    3     1   seq
...................ugagguaguagguuguauaguuAggu...................................................................    1     1   seq
............................................................................cuauacagucuauuCccuucc...............    1     1   seq
............................................................................cuauacagucuauugccuucU...............    1     1   seq
............................................................................Uuauacagucuauugccuuccu..............    1     1   seq
............................................................................cuauacagucuauugccuucUu..............    4     1   seq
............................................................................cuauacaAucuauugccuuccu..............    87    1   seq
............................................................................cuauacagucuauugccuuccA..............    4     1   seq
............................................................................cuauacagucuauugccuucUuu.............    1     1   seq
............................................................................cuauacaAucuauugccuuccuu.............    5     1   seq
............................................................................cuauacagucuauugccuuccuA.............    5     1   seq
............................................................................cuauacagucuauugccuuccAu.............    2     1   seq
............................................................................cuauacagucuauugccuuccuuU............    24    1   seq
.............................................................................uauacaAucuauugccuuccu..............    1     1   seq
```

Provisional ID	:	mun-novel78
Score total	:	1.8
Score for star read(s)	:	-1.3
Score for read counts	:	0
Score for mfe	:	2.1
Score for randfold	:	1.6
Score for cons. seed	:	-0.6
Total read count	:	35
Mature read count	:	35
Loop read count	:	0
Star read count	:	0

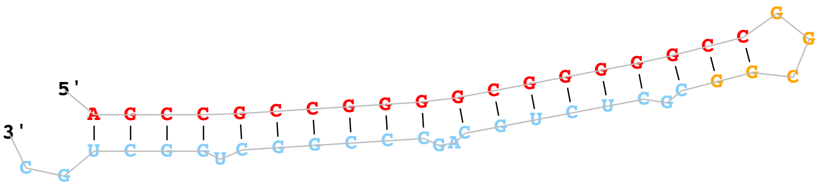

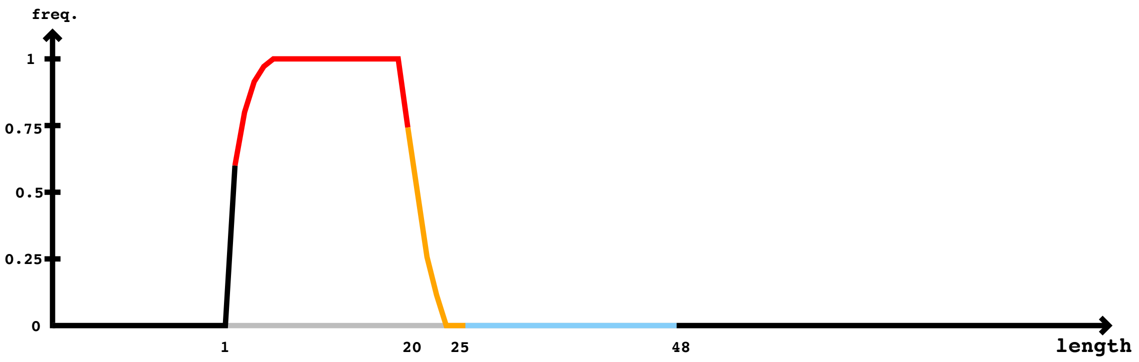

Mature Star

```
5'- gccuuucccucccucgcagccgccggggcgggggccggcggcgcucugcagcccggcuggcugcggcuuccugcgguaaccucucuccgccggnnnnnnnnnnnnnnnn   -3'    exp
    ............((((((((((((((((((((((...))).)))))))..))))))).))))))))...((.((((..........)))).))...............   reads    mm    sample
    .................Cgccgccggggcgggggc..........................................................................   9        1     seq
    .................Cgccgccggggcgggggcc.........................................................................   10       1     seq
    .................Cgccgccggggcgggggccgg.......................................................................   2        1     seq
    ..................gccgccggggcgggggcc.........................................................................   6        0     seq
    ..................gccgccggggcgggggccA........................................................................   1        1     seq
    ...................ccgccggggcgggggccgg.......................................................................   2        0     seq
    ...................ccgccggggcgggggccggG......................................................................   2        1     seq
    ....................cgccggggcgggggccgg.......................................................................   1        0     seq
    ....................cgccggggcgggggccggG......................................................................   1        1     seq
    .....................gccggggcgggggccggG......................................................................   1        1     seq
```

Provisional ID	:	mun-miR-138-2
Score total	:	430.1
Score for star read(s)	:	3.9
Score for read counts	:	419.7
Score for mfe	:	1.9
Score for randfold	:	1.6
Score for cons. seed	:	3
Total read count	:	835
Mature read count	:	826
Loop read count	:	1
Star read count	:	8

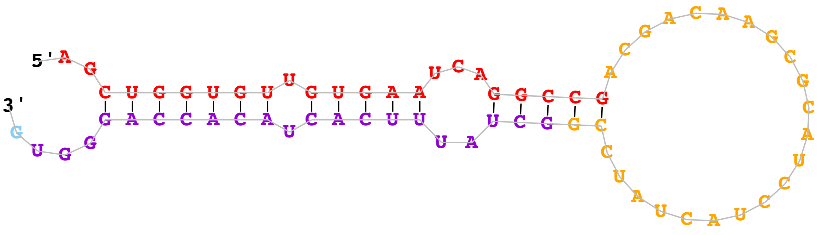

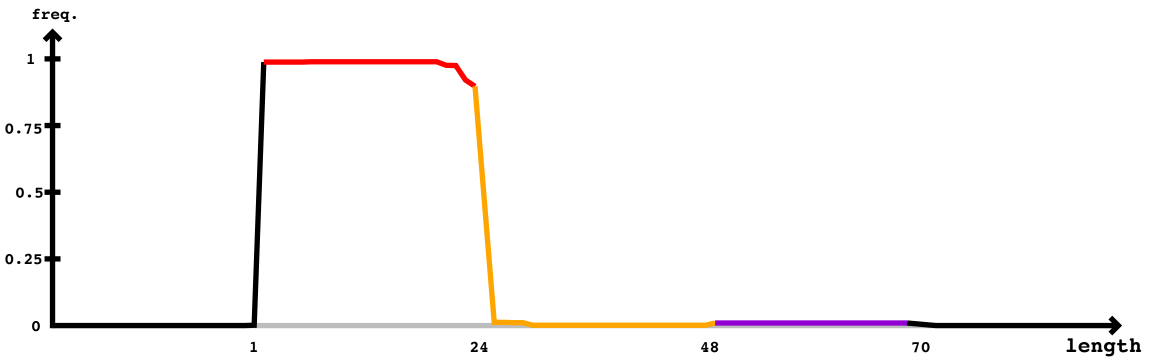

Mature **Star**

```
5'- ggaccugguauuggugcugcagcugguguugugaaucaggccgacgacaagcgcauccuacuauccggcuauuucacuacaccagggguggcaucauaccauuugcuuca  -3'   obs
    ggaccugguauuggugcugcagcugguguugugaaucaggccgacgacaagcgcauccuacuauccggcuauuucacuacaccagggguggcaucauaccauuugcuuca        exp
    .....((((((.(((((..(..(((((((.(((((...(((((.......................))))).)..))))).))))))).)..)))))))))))........   reads   mm   sample
    ...................cagcugguguugugaaucaggcc...................................................................   1       0    seq
    ....................agcuggGguugugaaucag......................................................................   1       1    seq
    ....................agcugguguugugaaucag......................................................................   10      0    seq
    ....................agcugguguugugaaucagg.....................................................................   1       0    seq
    ....................agcuggCguugugaaucaggc....................................................................   1       1    seq
    ....................agcugCuguugugaaucaggc....................................................................   1       1    seq
    ....................agcugguguugugaaucaggc....................................................................   41      0    seq
    ....................agcugguguugugaaucaggU....................................................................   2       1    seq
    ....................agcugguguugugaaucaggcc...................................................................   17      0    seq
    ....................agcugguguugugaaucaggcU...................................................................   2       1    seq
    ....................agcugguguugugaaucaggccA..................................................................   27      1    seq
    ....................agcugguguugugaaucaggccU..................................................................   4       1    seq
    ....................agcugguguugugaaucaggccg..................................................................   705     0    seq
    ....................agcugguguugugaaucaUgccg..................................................................   1       1    seq
    ....................agcugguguugugaaucaggccgG.................................................................   2       1    seq
    ....................agcugguguugugaaucaggccgacg..............................................................   1       0    seq
    ....................agcugguguugugaaucaggccgacgac............................................................   8       0    seq
    .........................guguugugaaucaggccg..................................................................   1       0    seq
    ...........................................acgacaagcgcauccuacuauccg..........................................   1       0    seq
    ...................................................................gcuauuucacuacaccagggu.....................   5       0    seq
    ...................................................................gcuauuucacuacaccaggguA....................   2       1    seq
    ...................................................................gcuauuucacuacaccaggguU....................   1       1    seq
```

Provisional ID	:	mun-novel37
Score total	:	994.3
Score for star read(s)	:	3.9
Score for read counts	:	983.6
Score for mfe	:	2.2
Score for randfold	:	1.6
Score for cons. seed	:	3
Total read count	:	1941
Mature read count	:	1860
Loop read count	:	0
Star read count	:	81

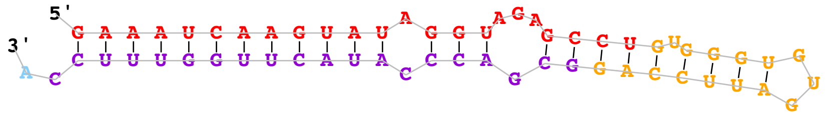

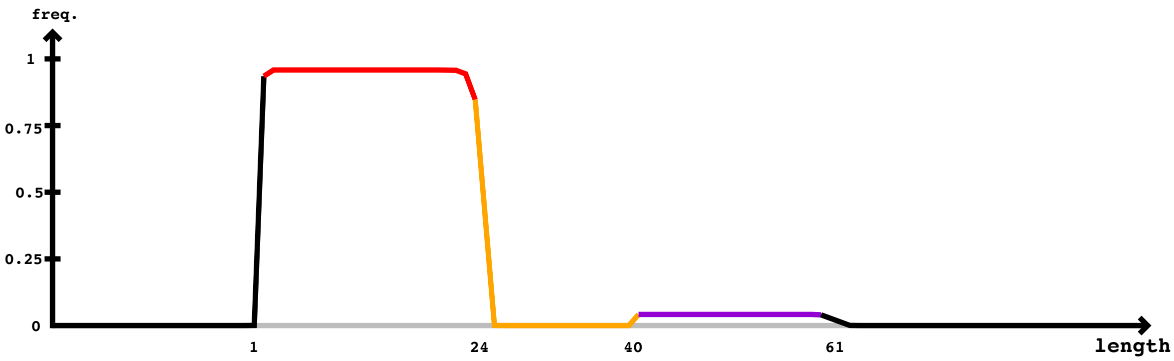

Mature   Star

```
5'- guuugcugugcgugaccuuggaaaucaaguauagguagagccugugggugugauuccaggcgacccauacuugguuuccagggugugcagggaaucuacagugggaagcaag  -3'    obs
    guuugcugugcgugaccuuggaaaucaaguauagguagagccugugggugugauuccaggcgacccauacuugguuuccagggugugcagggaaucuacagugggaagcaag         exp
    ((((.(((..(...((((((((((((((((((.(((...(((((.((((...))))))))).))).))))))))))))))))))..))).)))).................  reads   mm   sample
    ...................Cgaaaucaaguauagguagagccu.....................................................................  1       1    seq
    ....................gaaaucaaguauagguaga.........................................................................  1       0    seq
    ....................gaaaucaaguauagguagag........................................................................  1       0    seq
    ....................gaaaucaaguauagguagagU.......................................................................  1       1    seq
    ....................gaaaucaaguauagguagagc.......................................................................  25      0    seq
    ....................gaaaucaaguauagguagagcc......................................................................  186     0    seq
    ....................gaaaCcaaguauagguagagcc......................................................................  1       1    seq
    ....................gaaaucaaguauaggGagagccu.....................................................................  1       1    seq
    ....................gaaaucaaguauaggCagagccu.....................................................................  4       1    seq
    ....................gaaaucaaguauagguagagccA.....................................................................  88      1    seq
    ....................gaaaucaaguauagguagagccC.....................................................................  1       1    seq
    ....................gaaaucaaguauaggAagagccu.....................................................................  2       1    seq
    ....................gaaaucaagAauagguagagccu.....................................................................  1       1    seq
    ....................gaaaucaaguauagguagagccG.....................................................................  27      1    seq
    ....................gaaauUaaguauagguagagccu.....................................................................  1       1    seq
    ....................gaaaucaaguauagguagagccu.....................................................................  1469    0    seq
    ....................gaaaucaagGauagguagagccu.....................................................................  1       1    seq
    ....................gaaaucaaguauagguagagccuU....................................................................  4       1    seq
    .....................aaaucaaguauagguagagcc......................................................................  1       0    seq
    .....................aaaucaaguauagguagagccA.....................................................................  1       1    seq
    .....................aaaucaaguauagguagagccu.....................................................................  42      0    seq
    .....................aaaucaaguauagguagagccuUu...................................................................  1       1    seq
    ...........................................................gcgacccauacuugguuuc..................................  3       0    seq
    ...........................................................gcgacccauacuugguuucc.................................  47      0    seq
    ...........................................................gcgacccauacuugguuucca................................  24      0    seq
    ...........................................................gcgaccAauacuugguuucca................................  1       1    seq
    ...........................................................gcgacccauacuugguuuccC................................  2       1    seq
    ...........................................................gcgacccauacuugguuuccU................................  1       1    seq
    ...........................................................gcgacccauacuugguuuccaU...............................  1       1    seq
    ...........................................................gcgacccauacuugguuuccaA...............................  1       1    seq
    ...........................................................gcgacccauacuugguuuccagA..............................  1       1    seq
```

Provisional ID	:	mun-miR-1451
Score total	:	2197.9
Score for star read(s)	:	3.9
Score for read counts	:	2186.8
Score for mfe	:	2.7
Score for randfold	:	1.6
Score for cons. seed	:	3
Total read count	:	4301
Mature read count	:	4297
Loop read count	:	0
Star read count	:	4

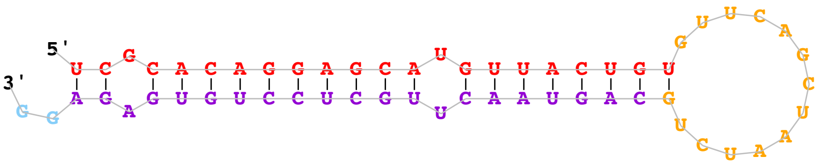

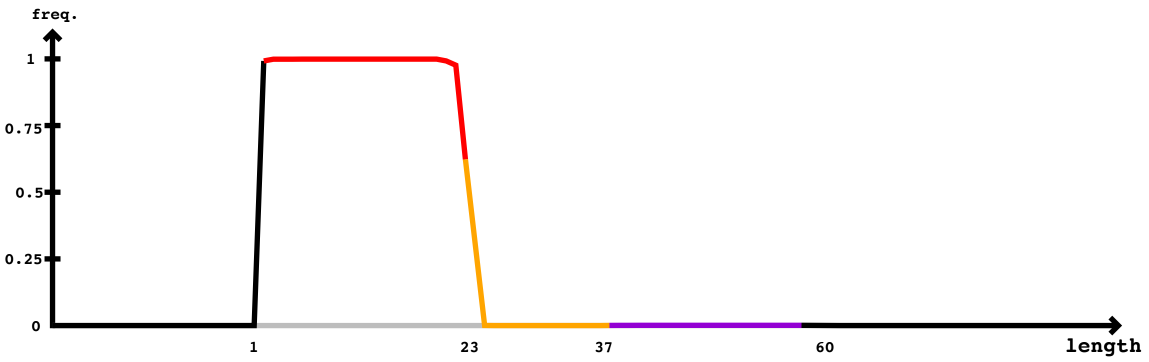

Mature **Star**

```
5'- caagcacaggagcugcugccucgcacaggagcauguuacuguguucagcuaaucugcaguaacuugcuccugugagaggguggcggcuccuggcuccaaccugcuucuuu -3'   obs
    caagcacaggagcugcugccucgcacaggagcauguuacuguguucagcuaaucugcaguaacuugcuccugugagagguggcggcuccuggcuccaaccugcuucuuu        exp
    ..(((.((((((((((..((((.(((((((((.((((((((.............)))))))).))))))))).))))..)))))))))))))..................   reads   mm   sample
    ....................ucgcacaggagcauguuac......................................................................   29      0    seq
    ....................ucgcGcaggagcauguuacu.....................................................................   1       1    seq
    ....................ucgcacaggagcauguuacu.....................................................................   67      0    seq
    ....................ucgcacaggagUauguuacu.....................................................................   1       1    seq
    ....................Ccgcacaggagcauguuacug....................................................................   1       1    seq
    ....................ucgcacaggagUauguuacug....................................................................   1       1    seq
    ....................ucgGacaggagcauguuacug....................................................................   1       1    seq
    ....................ucgcacaggagcauguuacuC....................................................................   2       1    seq
    ....................ucgcacaggagcauguuGcug....................................................................   1       1    seq
    ....................ucgcacaggagcauguuaAug....................................................................   1       1    seq
    ....................ucgcacaggagcauguGacug....................................................................   1       1    seq
    ....................ucgcacaggagcauguuacug....................................................................   1494    0    seq
    ....................ucgcacaggagcauguuacuU....................................................................   2       1    seq
    ....................Gcgcacaggagcauguuacug....................................................................   3       1    seq
    ....................ucgcacaggagcauguuacGg....................................................................   4       1    seq
    ....................ucUcacaggagcauguuacug....................................................................   1       1    seq
    ....................ucgcacaggaCcauguuacug....................................................................   1       1    seq
    ....................Acgcacaggagcauguuacugu...................................................................   1       1    seq
    ....................ucgAacaggagcauguuacugu...................................................................   2       1    seq
    ....................uAgcacaggagcauguuacugu...................................................................   1       1    seq
    ....................ucgcacaggagcaCguuacugu...................................................................   1       1    seq
    ....................ucgcacaggagcauguuacugG...................................................................   6       1    seq
    ....................ucgUacaggagcauguuacugu...................................................................   1       1    seq
    ....................ucgcacaggagcauguuacugA...................................................................   9       1    seq
    ....................ucgcacaggagcauguuacugu...................................................................   2499    0    seq
    ....................ucgcacaggGgcauguuacugu...................................................................   1       1    seq
    ....................ucgcacagUagcauguuacugu...................................................................   1       1    seq
    ....................Gcgcacaggagcauguuacugu...................................................................   1       1    seq
    ....................ucgcacaggagcaugAuacugu...................................................................   1       1    seq
    ....................ucgcacaggagcauguuacugC...................................................................   2       1    seq
    ....................ucgcacaggagUauguuacugu...................................................................   2       1    seq
    ....................ucAcacaggagcauguuacugu...................................................................   1       1    seq
    ....................ucgcacaggagAauguuacugu...................................................................   1       1    seq
```

```
                    Mature                             Star
caagcacaggagcugcugccucgcacaggagcauguuacuguguucagcuaaaucugcaguaacuugcuccugugagagguggcggcuccuggcuccaaccugcuucuuu
....................ucgcacaggaCcauguuacugu....................................................................    4    1    seq
....................ucgcGcaggagcauguuacugu....................................................................    1    1    seq
....................ucgcacaggagcauAuuacugu....................................................................    1    1    seq
....................ucgcacaggagcauguuacuguU...................................................................    65   1    seq
....................ucgcacaggagcauguuacuguA...................................................................    54   1    seq
....................ucgcacaggagcauguuacuguAu..................................................................    1    1    seq
....................ucgcacaggagcauguuacuguUu..................................................................    1    1    seq
.....................cgcacaggagUauguuacug.....................................................................    1    1    seq
.....................cgcacaggagcauguuacug.....................................................................    4    0    seq
.....................cgcacaggagcauguuacugu....................................................................    21   0    seq
.....................cgcacaggagcauguuacugug...................................................................    1    0    seq
.....................cgcacaggagcauguuacuguU...................................................................    1    1    seq
........................acaggagcauguuacugu....................................................................    1    0    seq
.........................................................caguaacuugcuccugugaga................................    2    0    seq
..........................................................aguaacuugcuccugugagaggu.............................    1    0    seq
............................................................uaacuugcuccugugagagg..............................    1    0    seq
```

Provisional ID	:	mun-novel7
Score total	:	0.8
Score for star read(s)	:	-1.3
Score for read counts	:	-0.8
Score for mfe	:	2
Score for randfold	:	1.6
Score for cons. seed	:	-0.6
Total read count	:	10
Mature read count	:	10
Loop read count	:	0
Star read count	:	0

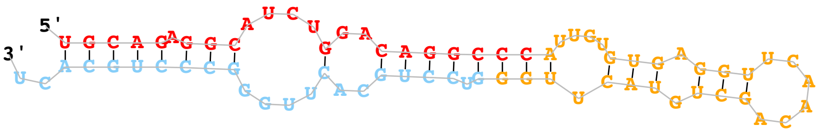

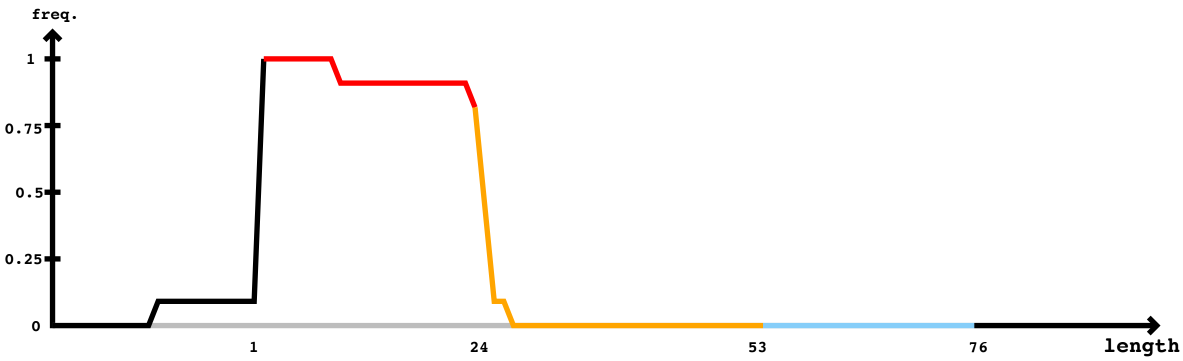


```
                    Mature                                              Star
5'- gcuggaggguaggaaggcucugcagaggcaucuggacaggcccauugugugagguucaacagcuguacuuggguccugcacuugggcccugcacuugggccacaacaaccuca  -3'   exp
    ....((((.......((((((((((.(((....(..((((((((....(((.(((......))).))).)))).))))..)....))))))))...))))).......)))).   reads      mm        sample
    .........uaggaaggcucugcagagg.....................................................................................   1          0         seq
    ....................ugcagaggcaucuggacaggcc.......................................................................   1          0         seq
    ....................ugcagaggcaucuggacaggccc......................................................................   8          0         seq
    ....................ugcagaggcaucuggacaggcccauu...................................................................   1          0         seq
```

Provisional ID	:	mun-novel72
Score total	:	27.4
Score for star read(s)	:	3.9
Score for read counts	:	24.6
Score for mfe	:	1.7
Score for randfold	:	-2.2
Score for cons. seed	:	-0.6
Total read count	:	60
Mature read count	:	49
Loop read count	:	0
Star read count	:	11

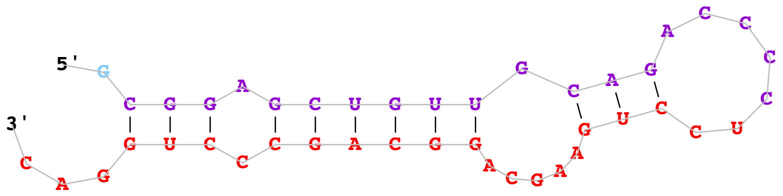

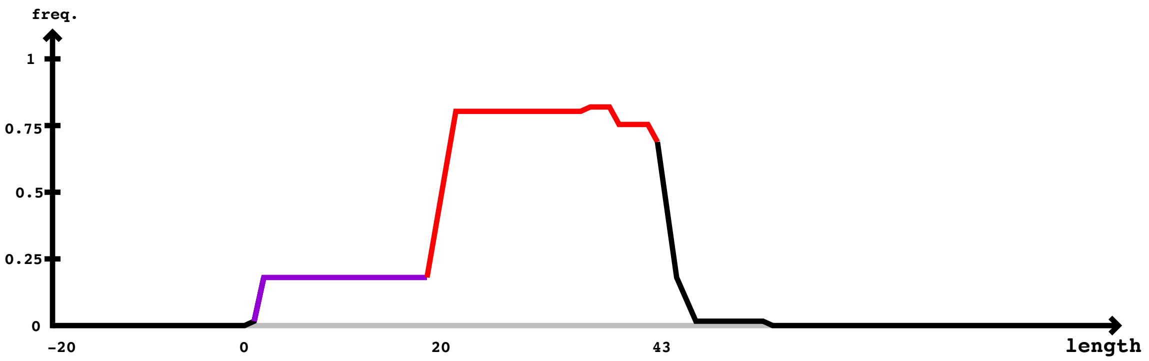

Star Mature

```
5'- ccuggagcgaguucaagguggcggagcuguugcagaccccuccugaagcaggcagcccuggacacaugcugcuggcaccacaucuugugccccgugaugggcacagcuga   -3'   obs
    ccuggagcgaguucaaggugcggagcuguugcagaccccuccugaagcaggcagcccuggacacaugcugcuggcaccacaucuugugccccgugaugggcacagcuga         exp
    ..(((.((.(((.((.(((.(((.((((((.(((.......))).....)))))).))).)).).))..))).)).)))....((((((((......))))))))....   reads   mm   sample
    ...................gcggagcuguugcagacccc.......................................................................   1       0    seq
    ....................cAgagcuguugcagacccc.......................................................................   1       1    seq
    ....................cggagcuguugcagacccc.......................................................................   9       0    seq
    .......................................uccugaagcaggcagccc....................................................   4       0    seq
    .......................................uccugaagcaggcagcccugga................................................   4       0    seq
    .......................................uccugaagcaggcagcccuggac...............................................   15      0    seq
    .......................................uccugaagcaggcagcccuggaA...............................................   1       1    seq
    .......................................uccugaagcaggcagcccuggCc...............................................   5       1    seq
    .......................................uccugaagcaggcagcccCggac...............................................   3       1    seq
    .......................................uccugaagcaggcagcccuggacU..............................................   2       1    seq
    .......................................uccugaagcaggcagcccuggaca..............................................   3       0    seq
    .......................................uccugaagcaggcagcccuggaAa..............................................   1       1    seq
    .......................................uccugaagcaggcagcccCggaca..............................................   1       1    seq
    .......................................uccugaagcaggcagcccuggacac.............................................   2       0    seq
    .......................................uccugaagcaggcagcccuggacaU.............................................   1       1    seq
    .......................................uccugaagcagAcagcccuggacac.............................................   1       1    seq
    .......................................uccugaagcaggcagcccCggacac.............................................   1       1    seq
    .......................................uccugaagcaggcagcccuggacacU............................................   1       1    seq
    .......................................uccugaagcaggcagcccuggacaca............................................   4       0    seq
    ......................................................cccuggacacaugcugcug....................................   1       0    seq
```

Provisional ID	:	mun-miR-1467
Score total	:	1272.7
Score for star read(s)	:	3.9
Score for read counts	:	1262.5
Score for mfe	:	1.7
Score for randfold	:	1.6
Score for cons. seed	:	3
Total read count	:	2488
Mature read count	:	2418
Loop read count	:	1
Star read count	:	69

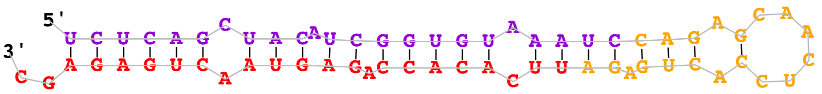

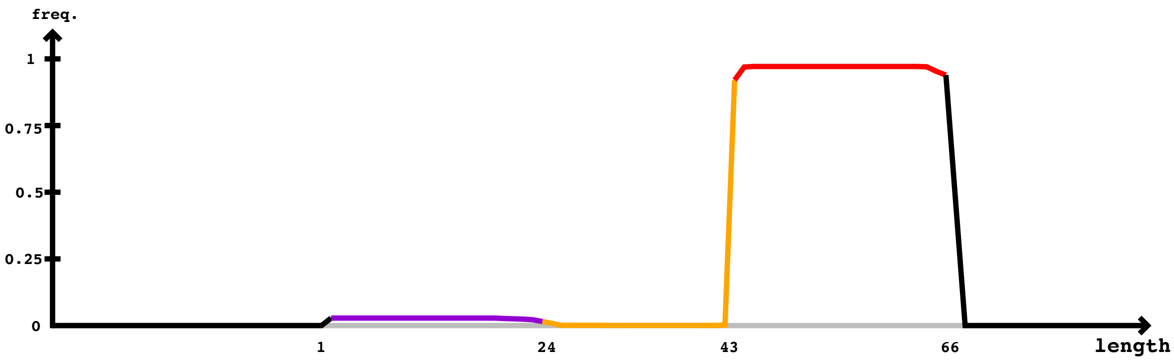

Star Mature

```
5'- aaggagagcugacagggcagguuuuuuucucagcuacaucgguguaaaauccagagcaacuccacugagauucacaccagaguaacugagagcagaaucuggccaacaucauc  -3'  obs
    aaggagagcugacagggcagguuuuuuucucagcuacaucgguguaaaauccagagcaacuccacugagauucacaccagaguaacugagagcagaaucuggccaacaucauc       exp
    ..............((.((((((((.(((((((.(((.(((((((.(((((((.(......).))).)))).))))).))))).))))))).))))))))).))..........   reads  mm  sample
    ...........................ucucagcuacaucggugu....................................................................   4      0   seq
    ...........................ucucagcuacaucgguguа...................................................................   3      0   seq
    ...........................ucucagcuacaucgguguaa..................................................................   3      0   seq
    ...........................ucucagcuacaucgguguaaa.................................................................   6      0   seq
    ...........................ucucagcuacaucgguguaaaau...............................................................   16     0   seq
    ...........................ucAcagcuacaucgguguaaaauc..............................................................   1      1   seq
    ...........................ucucagcuacaucgguguaaaauc..............................................................   30     0   seq
    ...........................ucucagcuacaucgguguaaaaucU.............................................................   6      1   seq
    ...........................ucucagcuacaucgguguaaaauccagagc........................................................   1      0   seq
    ...................................................cagagcaacuccacugaga...........................................   1      0   seq
    .....................................................................auucacaccagaguaacugagU......................   1      1   seq
    .....................................................................Cuucacaccagaguaacugagagc....................   2      1   seq
    ......................................................................uucacaccagaguaacugag.......................   2      0   seq
    ......................................................................uucacaccagaguaacugaA.......................   1      1   seq
    ......................................................................uucacaccagaguaaGugaga......................   1      1   seq
    ......................................................................uucacaccagaguaacugaAa......................   2      1   seq
    ......................................................................uucacaccGgaguaacugaga......................   1      1   seq
    ......................................................................uucacaccagaguaacugaga......................   36     0   seq
    ......................................................................uucacaccagaguaacugagaA.....................   1      1   seq
    ......................................................................uucacaccagaguaacugagag.....................   32     0   seq
    ......................................................................uucacaccagagGaacugagag.....................   1      1   seq
    ......................................................................uucacaccagagCaacugagagc....................   1      1   seq
    ......................................................................uucacaccagaguaacCgagagc....................   2      1   seq
    ......................................................................Gucacaccagaguaacugagagc....................   1      1   seq
    ......................................................................uucacaccGgaguaacugagagc....................   14     1   seq
    ......................................................................uucacacAagaguaacugagagc....................   1      1   seq
    ......................................................................uucacaccagaguaacugagaAc....................   3      1   seq
    ......................................................................uucacaccagaguaacugagagc....................   1794   0   seq
    ......................................................................uucacaccagaguaacugagagA....................   15     1   seq
    ......................................................................uucacaccagaguaaAugagagc....................   1      1   seq
    ......................................................................uucacaccagaguaacugagagU....................   42     1   seq
    ......................................................................uucacaccagagAaacugagagc....................   2      1   seq
    ......................................................................uucacaccagaguaacuUagagc....................   2      1   seq
```

Star Mature

```
aaggagagcugacagggcagguuuuuuucucagcuacaucgguguaaauccagagcaacuccacugagauucacaccagaguaacugagagcagaaucuggccaacaucauc
.....................................................................uucacaccagagGaacugagagc....................	1	1	seq
.....................................................................uucacaccagaguaacugagagG....................	1	1	seq
.....................................................................uucacGccagaguaacugagagc....................	1	1	seq
.....................................................................uucacaccagaguaGcugagagc....................	4	1	seq
.....................................................................uuUacaccagaguaacugagagc....................	1	1	seq
.....................................................................uucacaccagaguaacugagagcC...................	8	1	seq
.....................................................................uucacaccagaguaacugagagcU...................	314	1	seq
.....................................................................uucacaccagaguaacugagagca...................	3	0	seq
......................................................................ucacaccGgaguaacugaga......................	1	1	seq
......................................................................ucacaccagaguaacugagag.....................	1	0	seq
......................................................................ucacaccGgaguaacugagagc....................	10	1	seq
......................................................................ucacaccagaguaacugagagc....................	58	0	seq
......................................................................ucacaccagaguaacugagagU....................	1	1	seq
......................................................................ucacaccagaguaacugagagcC...................	4	1	seq
......................................................................ucacaccagaguaacugagagcU...................	47	1	seq
.......................................................................cacaccagaguaacugagagc....................	2	0	seq
.......................................................................cacaccagaguaacugagagcU...................	3	1	seq
```

Provisional ID	:	mun-miR-2954
Score total	:	1.4
Score for star read(s)	:	-1.3
Score for read counts	:	0
Score for mfe	:	1.9
Score for randfold	:	-2.2
Score for cons. seed	:	3
Total read count	:	1770
Mature read count	:	1770
Loop read count	:	0
Star read count	:	0

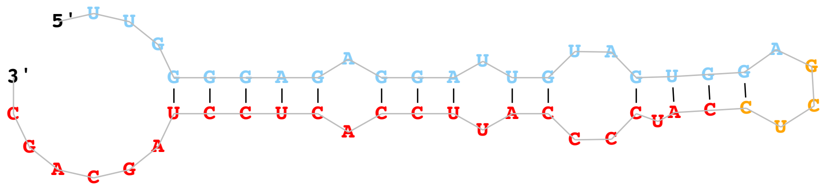

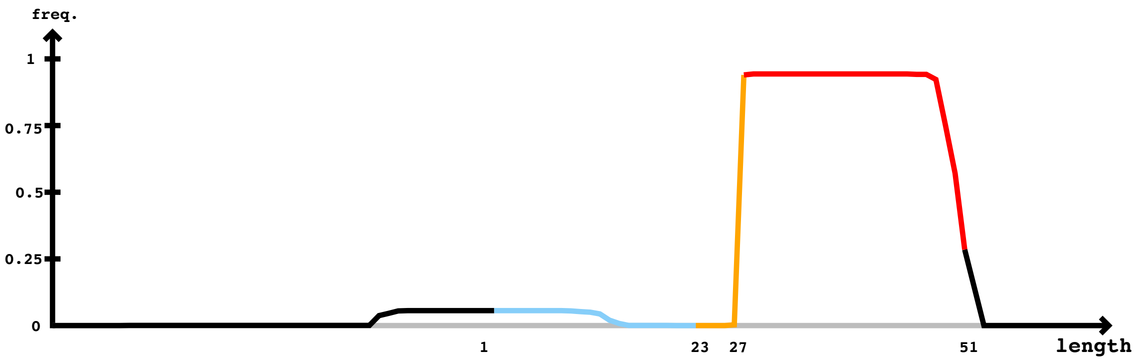

Star Mature

```
5'- ccggcagugaaaugagucugggcuuggagcagugcugagagggcuuggggagaggauuguaguggagcuccauccccauuccacuccuagcagcucucuggccauccu   -3'   exp
    .(((((.((...(((((....)))))...)).))))).(((((((..(((((.(((.((..((((....))).)..)).))).)))))...)))))))..........   reads   mm   sample
    ......gugaaaugagucugggcuuggagcagu...........................................................................   1   0   seq
    ................................ugcugagagggcuuggggag........................................................   2   0   seq
    ................................ugcugagagggcuuggggaAa.......................................................   2   1   seq
    ................................ugcugagagggcuuggggaga.......................................................   3   0   seq
    ................................ugcugagagggcuuggggagaU......................................................   1   1   seq
    ................................ugcugagagggcuuggggagaA......................................................   2   1   seq
    ................................ugcugagagggcuuggggagag......................................................   1   0   seq
    ................................ugcugagagggcuuggggagagA.....................................................   1   1   seq
    ................................ugcugagagggcuuggggagagg.....................................................   6   0   seq
    ................................ugcugagagggcuuggggagagU.....................................................   2   1   seq
    ................................ugcugagagggcuuggggagaggU....................................................   1   1   seq
    ................................ugcugagagggcuuggggagagga....................................................   27   0   seq
    ................................ugcugagagggcuuggggagaggau...................................................   15   0   seq
    ................................ugcugagagggcuuggggagaggaAu..................................................   2   1   seq
    ................................ugcugagagggcuuggggagaggauu..................................................   4   0   seq
    .................................gcugagagggcuuggggagagg.....................................................   1   0   seq
    .................................gcugagagggcuuggggagagga....................................................   13   0   seq
    .................................gcugagagggcuuggggagaggaAu..................................................   1   1   seq
    .................................gcugagagggcuuggggagaggauu..................................................   2   0   seq
    ..................................cugagagggcuuggggagagg.....................................................   2   0   seq
    ..................................cugagagggcuuggggagagga....................................................   3   0   seq
    ..................................cugagagggcuuggggagaggau...................................................   7   0   seq
    ..................................cugagagggcuuggggagaggauu..................................................   3   0   seq
    ..................................cugagagggcuuggggagaggaAu..................................................   1   1   seq
    ..................................cugagagggcuuggggagaggauuguagu.............................................   1   0   seq
    ...................................ugagagggcuuggggagaggau...................................................   1   0   seq
    ...................................ugagagggcuuggggagaggauu..................................................   1   0   seq
    .....................................................................ccauccccauuccacuccuagc.................   4   0   seq
    .....................................................................ccauccccauuccacuccuagcU................   1   1   seq
    ......................................................................cauccccauuccacuccu....................   3   0   seq
    ......................................................................cauccccauuccacuccuag..................   36   0   seq
    ......................................................................cauccccauuccacuccuagU.................   1   1   seq
    ......................................................................cauccccauuccacuccuagc.................   305   0   seq
    ......................................................................cauccccauuccacuccuagA.................   12   1   seq
```

Star Mature

```
ccggcagugaaaugagucugggcuuggagcagugcugagagggcuuggggagaggauuguaguggagcuccauccccauuccacuccuagcagcucucuggccauccu
......................................................................Uauccccauuccacuccuagc.................	1	1	seq
......................................................................cauccccauuccacuccuagAa................	19	1	seq
......................................................................cauccccauuccacuccuagca................	288	0	seq
......................................................................cauccccauuccacuccuagcU................	21	1	seq
......................................................................cauccccauuccacuccuagcG................	5	1	seq
......................................................................cauccccauuccacuccuagcaU...............	51	1	seq
......................................................................cauccccauuccacuccuagcag...............	376	0	seq
......................................................................cauccccauuccacuccuagcaC...............	43	1	seq
......................................................................cauccccauuccacuccuagcaA...............	64	1	seq
......................................................................cauccccauuccacuccuagcUg...............	1	1	seq
......................................................................caCcccauuccacuccuagcag...............	1	1	seq
......................................................................cauccccauuccacuccuagcagG..............	1	1	seq
......................................................................cauccccauuccacuccuagcaCc..............	17	1	seq
......................................................................cauccccauuccacuccuagcagU..............	440	1	seq
......................................................................cauccccauuccacuccuagcagA..............	24	1	seq
......................................................................cauccccauuccacuccuagcagc..............	7	0	seq
......................................................................cauccccauuccacuccuagcagUu.............	39	1	seq
......................................................................cauccccauuccacuccuagcaCcu.............	1	1	seq
......................................................................cauccccauuccacuccuagcagcu.............	1	0	seq
......................................................................cauccccauuccacuccuagcagAu.............	1	1	seq
.......................................................................auccccauuccacuccuagc.................	1	0	seq
.......................................................................auccccauuccacuccuagca................	3	0	seq
.......................................................................auccccauuccacuccuagcagU..............	3	1	seq
```

Provisional ID	:	mun-novel16
Score total	:	31.4
Score for star read(s)	:	3.9
Score for read counts	:	23.6
Score for mfe	:	2.9
Score for randfold	:	1.6
Score for cons. seed	:	-0.6
Total read count	:	58
Mature read count	:	55
Loop read count	:	0
Star read count	:	3

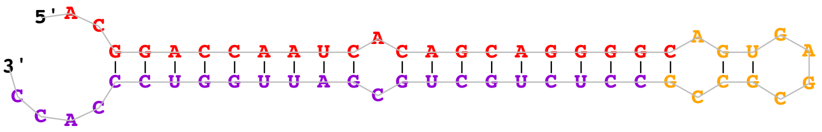

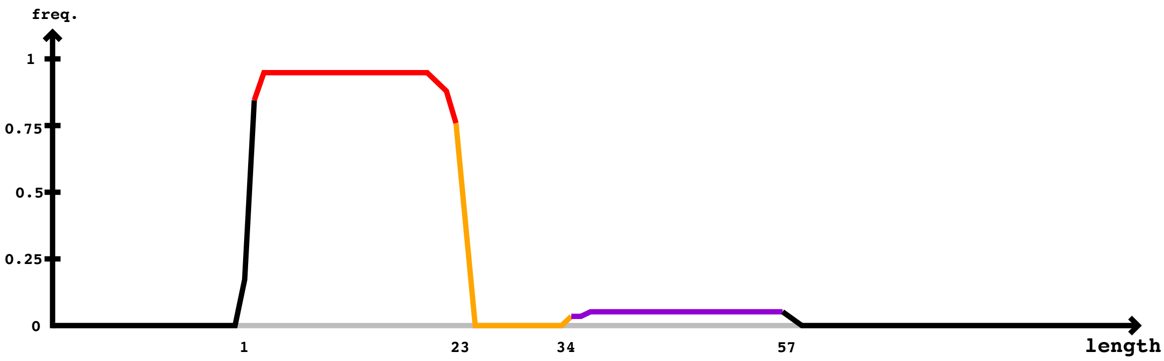

Mature **Star**

```
5'- ucgcugccccucccuccggacggaccaaucacagcaggggcagugagcgccgccucugcugcgauuggucccaccggagagaggggaggggcgccuuagccccgccucccc   -3'   obs
    ucgcugccccucccuccggacggaccaaucacagcaggggcagugagcgccgccucugcugcgauuggucccaccggagagaggggaggggcgccuuagccccgccucccc         exp
    ......((((((.((((((..(((((((((.((((((((((.((....)).)))))))))).)))))))))..)))))).)))))).(((((......)))))........   reads   mm   sample
    ..................gacggaccaaucacagcagU.........................................................................   1   1   seq
    ..................gacggaccaaucacagcaggA........................................................................   2   1   seq
    ..................gacggaccaaucacagcaggAg.......................................................................   3   1   seq
    ..................gacggaccaaucacagcaggAgc......................................................................   3   1   seq
    ..................gacggaccaaucacagcaggggU......................................................................   1   1   seq
    ...................acggaccaaucacagcagU.........................................................................   1   1   seq
    ...................acggaccaaucacagcagggg.......................................................................   1   0   seq
    ...................acggaccaaucacagcaggAg.......................................................................   2   1   seq
    ...................acggaccaaucacagcaggAgc......................................................................   31  1   seq
    ...................acggaccaaucacagcaggggc......................................................................   1   0   seq
    ...................acggaccaaucacagcaggggU......................................................................   3   1   seq
    ....................cggaccaaucacagcaggAg.......................................................................   1   1   seq
    ....................cggaccaaucacagcaggggU......................................................................   1   1   seq
    ....................cggaccaaucacagcaggAgc......................................................................   4   1   seq
    ....................................................ccucugcugcgauuggucccacU....................................   1   1   seq
    ....................................................ccucugcugcgauuggucccacc....................................   1   0   seq
    ......................................................ucugcugcgauuggucccaccU...................................   1   1   seq
```

Provisional ID : mun-novel13
Score total : 1.6
Score for star read(s) : -1.3
Score for read counts : 0
Score for mfe : 1.9
Score for randfold : 1.6
Score for cons. seed : -0.6
Total read count : 17
Mature read count : 17
Loop read count : 0
Star read count : 0

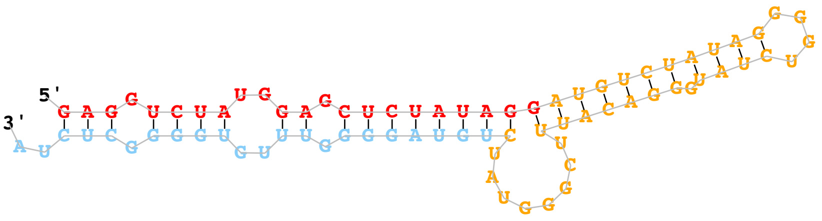

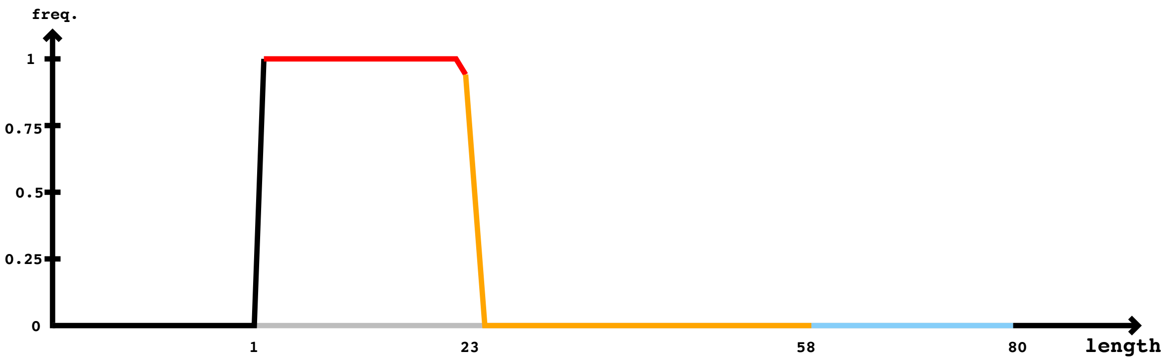


```
                        Mature                                          Star
5'- gggucugugggguaucuauagaggucuauggagcucuauaggaugucuauaggggucuaugggacauuucggguaucuguagggguuuguggggcucuauaggaucuaucg   -3'    exp
    .........((((.((((((((.((((..((.((((((((((((((((((....)))).)))))))........)))))))).))..)))).)))))))))))))....    reads    mm    sample
    ....................gaggucuauggagcucuauag.........................................................................    1        0     seq
    ....................gaggucuauggagcucuauagA........................................................................    8        1     seq
    ....................gaggucuauggagcucuauagU........................................................................    7        1     seq
    ....................gaggucuauggagcucuauagga.......................................................................    1        0     seq
```

Provisional ID : mun-let-7g-1
Score total : 12557.3
Score for star read(s) : 3.9
Score for read counts : 12546.4
Score for mfe : 2.4
Score for randfold : 1.6
Score for cons. seed : 3
Total read count : 24621
Mature read count : 24519
Loop read count : 1
Star read count : 101

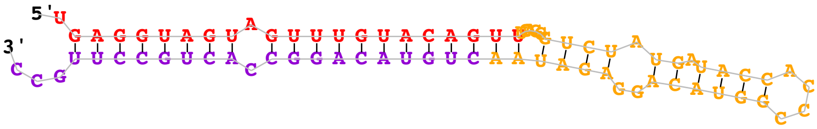

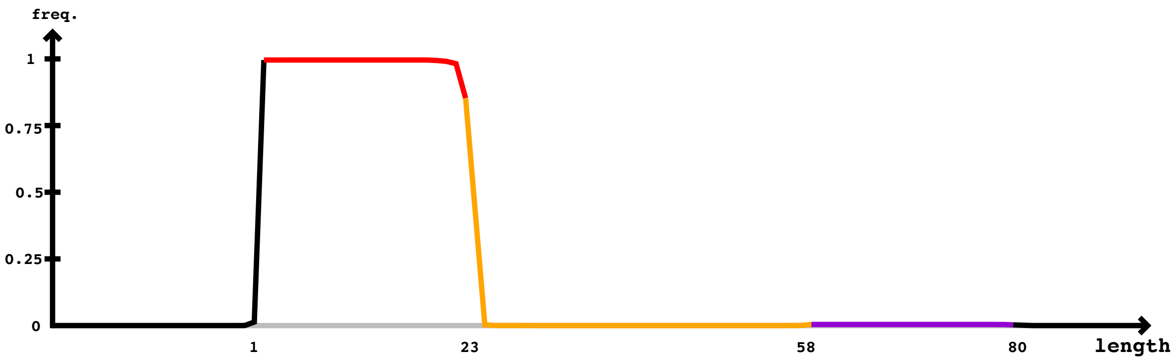

Mature Star

```
5'- ccucuugccugauuccaggcugagguaguaguuuguacaguuugagggucuaugauaccacccgguacaggagauaacuguacaggccacugccuugccugggacagagcgc  -3'   obs
    ccucuugccugauuccaggcugagguaguaguuuguacaguuugagggucuaugauaccacccgguacaggagauaacuguacaggccacugccuugccugggacagagcgc         exp
    ......((((.((((((((.(((((((.(((((((((((.....((((.((.((((....))))))..))))))))))))))).)))))))))))))))))))).))..   reads   mm   sample
    .cucuugccugauuccaggc............................................................................................   1   0   seq
    ..................Ucugagguaguaguuuguacaguu......................................................................   1   1   seq
    ...................cugagguaguaguuugua...........................................................................   2   0   seq
    ...................cugagguaguaguuuguac..........................................................................   4   0   seq
    ...................cugagguaguaguuuguaca.........................................................................   4   0   seq
    ...................cugagguaguaguuuguacag........................................................................   5   0   seq
    ...................cugagguaguaguuuguacagu.......................................................................   82   0   seq
    ...................Augagguaguaguuuguacaguu......................................................................   1   1   seq
    ...................cugaUguaguaguuuguacaguu......................................................................   1   1   seq
    ...................cugagguaguaguuuguacaguu......................................................................   203   0   seq
    ...................cugagguaguaguuuUuacaguu......................................................................   1   1   seq
    ...................cCgagguaguaguuuguacaguu......................................................................   1   1   seq
    ...................cugagguaguaguuugCacaguu......................................................................   1   1   seq
    ...................cugagguaguaguuuguacaguuu.....................................................................   15   0   seq
    ...................cugagguaguaguuuguacaguuA.....................................................................   3   1   seq
    ....................ugagguaguaguuuguac..........................................................................   40   0   seq
    ....................ugagguaguaguuuguacU.........................................................................   1   1   seq
    ....................ugagguaguaguuuguaca.........................................................................   74   0   seq
    ....................ugagguaguaguuuguacG.........................................................................   1   1   seq
    ....................ugagguaguaguuuguacaA........................................................................   3   1   seq
    ....................ugagguagAaguuuguacag........................................................................   1   1   seq
    ....................ugaggGaguaguuuguacag........................................................................   1   1   seq
    ....................ugagguaguaCuuuguacag........................................................................   1   1   seq
    ....................ugagguaguaguuuguacag........................................................................   198   0   seq
    ....................ugagguaguaguuuguacaU........................................................................   3   1   seq
    ....................ugagguaguaguuuUuacagu.......................................................................   1   1   seq
    ....................ugaAguaguaguuuguacagu.......................................................................   1   1   seq
    ....................ugagguaguGguuuguacagu.......................................................................   1   1   seq
    ....................ugagguaguagGuuguacagu.......................................................................   1   1   seq
    ....................ugaggGaguaguuuguacagu.......................................................................   3   1   seq
    ....................ugagguaguaUuuuguacagu.......................................................................   1   1   seq
    ....................ugaggCaguaguuuguacagu.......................................................................   3   1   seq
    ....................ugagguaguaguuugAacagu.......................................................................   1   1   seq
```

Mature Star

```
ccucuugccugauuccaggcugagguaguaguuuguacaguuugagggucuaugauaccacccgguacaggagauaacuguacaggccacugccuugccugggacagagcgc
....................ugagguaguaguuGguacagu.......................................................................   1       1   seq
....................ugagguaCuaguuuguacagu.......................................................................   1       1   seq
....................ugaUguaguaguuuguacagu.......................................................................   1       1   seq
....................ugaggAaguaguuuguacagu.......................................................................   4       1   seq
....................ugagguaguaguuuguacagA.......................................................................   8       1   seq
....................uUagguaguaguuuguacagu.......................................................................   1       1   seq
....................ugagguaguaguuuguacagu.......................................................................   3068    0   seq
....................Ggagguaguaguuuguacagu.......................................................................   3       1   seq
....................ugagguaguaguuuguaAagu.......................................................................   1       1   seq
....................ugagguaguaguuuAuacagu.......................................................................   1       1   seq
....................ugagguaguaguCuguacagu.......................................................................   1       1   seq
....................ugagguaguagCuuguacagu.......................................................................   1       1   seq
....................ugagguaguaguuuguacagC.......................................................................   1       1   seq
....................ugagguagCaguuuguacagu.......................................................................   2       1   seq
....................ugaUguaguaguuuguacaguu......................................................................   2       1   seq
....................ugagguaguagGuuguacaguu......................................................................   11      1   seq
....................ugagguaguaguuUuacaguu......................................................................   2       1   seq
....................ugagguaguaguuuguGcaguu......................................................................   2       1   seq
....................ugaAguaguaguuuguacaguu......................................................................   2       1   seq
....................ugagguaguaguuCguacaguu......................................................................   2       1   seq
....................ugagguaguaguuuguacaCuu......................................................................   1       1   seq
....................ugagguaguaguuuguacGguu......................................................................   2       1   seq
....................Ggagguaguaguuuguacaguu......................................................................   11      1   seq
....................Agagguaguaguuuguacaguu......................................................................   7       1   seq
....................ugagguaguaguuuguUcaguu......................................................................   1       1   seq
....................ugagguaguCguuuguacaguu......................................................................   1       1   seq
....................ugagguaguaguuuguacCguu......................................................................   1       1   seq
....................ugagguaguaguuuguacUguu......................................................................   3       1   seq
....................ugagguagGaguuuguacaguu......................................................................   3       1   seq
....................ugagguaguaguuugGacaguu......................................................................   1       1   seq
....................Cgagguaguaguuuguacaguu......................................................................   4       1   seq
....................ugagguaguaguuuCuacaguu......................................................................   2       1   seq
....................ugaCguaguaguuuguacaguu......................................................................   1       1   seq
....................ugagguaguaguuuAuacaguu......................................................................   1       1   seq
....................ugagguaguaguuugCacaguu......................................................................   2       1   seq
....................uCagguaguaguuuguacaguu......................................................................   2       1   seq
....................ugaggAaguaguuuguacaguu......................................................................   14      1   seq
....................ugagAuaguaguuuguacaguu......................................................................   1       1   seq
....................ugagguagAaguuuguacaguu......................................................................   1       1   seq
....................ugagguaguaguuuguacaguG......................................................................   12      1   seq
....................ugagguaguaguuuguacaguu......................................................................   17933   0   seq
....................ugUgguaguaguuuguacaguu......................................................................   1       1   seq
....................ugagCuaguaguuuguacaguu......................................................................   1       1   seq
....................ugagguaguaguuuguacaAuu......................................................................   2       1   seq
....................ugagguaguaUuuuguacaguu......................................................................   6       1   seq
....................ugaggGaguaguuuguacaguu......................................................................   15      1   seq
....................uAagguaguaguuuguacaguu......................................................................   1       1   seq
....................ugagguaguaguAuguacaguu......................................................................   1       1   seq
....................ugagguaCuaguuuguacaguu......................................................................   1       1   seq
....................ugagguagCaguuuguacaguu......................................................................   5       1   seq
....................ugagguaguaguuuguaAaguu......................................................................   3       1   seq
....................ugagguGguaguuuguacaguu......................................................................   4       1   seq
....................ugagguaguaAuuuguacaguu......................................................................   1       1   seq
....................ugagguaguaguCuguacaguu......................................................................   1       1   seq
....................ugagguaUuaguuuguacaguu......................................................................   2       1   seq
....................ugagguaguaCuuuguacaguu......................................................................   3       1   seq
....................ugagguaguaguuuguacaguA......................................................................   29      1   seq
....................uUagguaguaguuuguacaguu......................................................................   3       1   seq
....................ugagguaguaguuuguacaUuu......................................................................   6       1   seq
....................ugagguaguaguuuguaGaguu......................................................................   1       1   seq
....................ugagguaguaguuuguacaguC......................................................................   2       1   seq
....................ugagguaguaguuuguacagGu......................................................................   1       1   seq
....................ugagguaguGguuuguacaguu......................................................................   3       1   seq
....................ugaggCaguaguuuguacaguu......................................................................   8       1   seq
....................ugagguaguagAuuguacaguu......................................................................   3       1   seq
....................ugaggAaguaguuuguacaguuu.....................................................................   3       1   seq
....................ugGgguaguaguuuguacaguuu.....................................................................   1       1   seq
....................ugagguaguGguuuguacaguuu.....................................................................   2       1   seq
....................ugagguaguaguuuguacaguuA.....................................................................   669     1   seq
....................ugagguaguaguuuguacaguAu.....................................................................   4       1   seq
```

Mature **Star**

```
ccucuugccugauuccaggcugagguaguaguuuguacaguuugagggucuaugauaccacccgguacaggagauaacuguacaggccacugccuugccugggacagagcgc
....................ugagguaguaguuuguacaguuC.....................................................................	16	1	seq
....................ugagguaguaguuuguaAaguuu.....................................................................	2	1	seq
....................ugagguaguaguuuguacGguuu.....................................................................	1	1	seq
....................ugagguaguaUuuuguacaguuu.....................................................................	1	1	seq
....................ugagguaguaguuuguacaguuu.....................................................................	1840	0	seq
....................ugaggCaguaguuuguacaguuu.....................................................................	1	1	seq
....................ugagguaguaguuuguacaguuG.....................................................................	28	1	seq
....................ugCgguaguaguuuguacaguuu.....................................................................	1	1	seq
....................ugagguaUuaguuuguacaguuu.....................................................................	1	1	seq
....................Cgagguaguaguuuguacaguuu.....................................................................	1	1	seq
....................ugaggGaguaguuuguacaguuu.....................................................................	2	1	seq
....................ugagguagAaguuuguacaguuu.....................................................................	1	1	seq
....................ugagguaguaguuuguacaguuuU....................................................................	46	1	seq
....................ugagguaguaguuuguacaguuug....................................................................	1	0	seq
....................ugagguaguaguuuguacaguuuA....................................................................	5	1	seq
....................ugaggAaguaguuuguacaguuug....................................................................	1	1	seq
....................ugagguaguaguuuguacaguuuC....................................................................	1	1	seq
....................ugagguaguaguuuguacaguuAg....................................................................	1	1	seq
....................ugagguaguaguuuguacaguuugU...................................................................	1	1	seq
....................ugagguaguaguuuguacaguuugG...................................................................	1	1	seq
....................ugagguaguaguuuguacaguuugaA..................................................................	1	1	seq
....................ugagguaguaguuuguacaguuugagg.................................................................	3	0	seq
....................ugagguaguaguuuguacaguuugaggg................................................................	1	0	seq
....................ugagguaguaguuuguacaguuugagggu...............................................................	1	0	seq
.....................gagguaguaguuuguacaguu......................................................................	1	0	seq
.....................gagguaguaguuuguacaguuu.....................................................................	1	0	seq
.......................gguaguaguuuguacaguu......................................................................	1	0	seq
.........................uaguaguuuguacaguuugaggA................................................................	1	1	seq
..........................................ugagggucuaugauaccacccggu..............................................	1	0	seq
...........................................................................aacuguacaggccacugccu.................	1	0	seq
...........................................................................aacuguacaggccacugccuug...............	1	0	seq
...........................................................................aacuguacaggccacugccuugc..............	5	0	seq
............................................................................acuguacaggccacugccuug...............	1	0	seq
............................................................................acuguacaggccacugccuugc..............	2	0	seq
.............................................................................cuguacaggccacugccuug...............	1	0	seq
.............................................................................cuguacaggccacugccuugc..............	27	0	seq
.............................................................................cuguacaggccacugccuugcc.............	11	0	seq
.............................................................................cuguacaggccacugccuugcA.............	2	1	seq
.............................................................................cuguacaggccacugccuugcU.............	37	1	seq
.............................................................................cuguacaggccacugccuugccu............	3	0	seq
.............................................................................cuguacaggccacugccuugcUu............	2	1	seq
.............................................................................cuguacaggccacugccuugccA............	8	1	seq
```

Provisional ID	:	mun-novel42
Score total	:	1
Score for star read(s)	:	-1.3
Score for read counts	:	-0.8
Score for mfe	:	2.1
Score for randfold	:	1.6
Score for cons. seed	:	-0.6
Total read count	:	10
Mature read count	:	10
Loop read count	:	0
Star read count	:	0

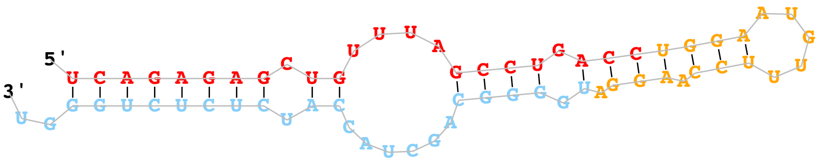

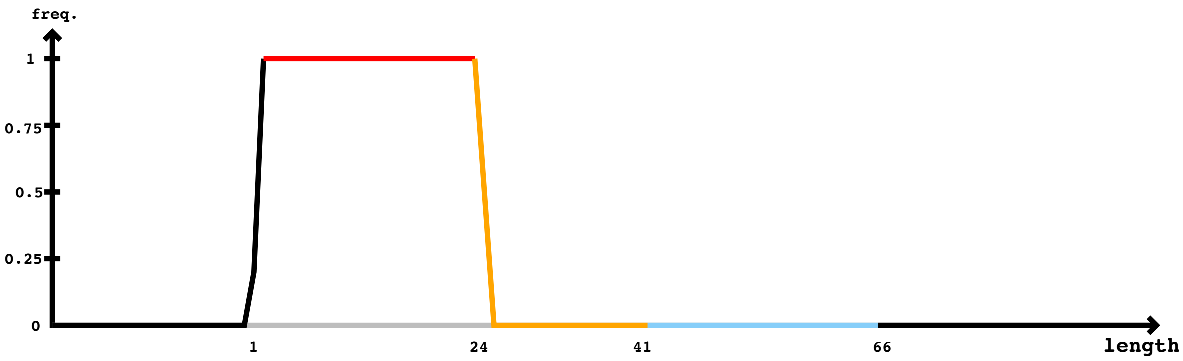

Mature Star

```
5'- cuuccauuggaucagguuguucagagagcuguuuagccugaccuggaauguuuccaaggauggggcagcuaccaucucucuggguaaccuguuccaguguuucaccacucuca   -3'    exp
    ....(((((((.(((((((..(((((((.((....((((.(((((((.....))).))).).))))......)).)))))))..))))))).)))))))..............    reads      mm        sample
    ..................uucagagagcuguuuagccugacc.......................................................................    2          0         seq
    ...................ucagagagcuguuuagccugacc.......................................................................    7          0         seq
    ...................ucagagagcuguuuagccugaccu......................................................................    1          0         seq
```

Provisional ID	:	mun-miR-34b
Score total	:	3690.2
Score for star read(s)	:	3.9
Score for read counts	:	3680
Score for mfe	:	1.7
Score for randfold	:	1.6
Score for cons. seed	:	3
Total read count	:	7230
Mature read count	:	7106
Loop read count	:	0
Star read count	:	124

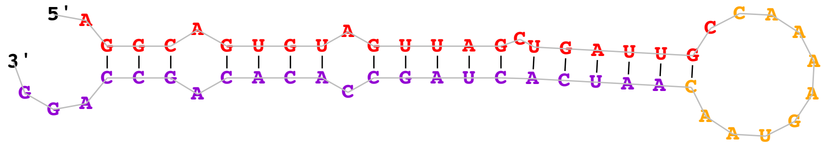

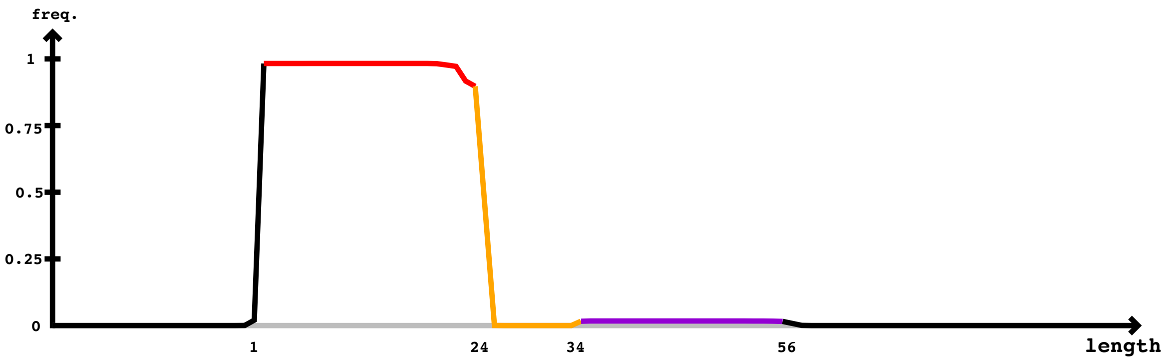

Mature Star

```
5'- cuucuaucagccuaguuacuaggcaguguaguuagcugauugccaaaaguaacaaucacuagccacacagccagguaaaaagguuuggaagauaccucaguggaaaccaga  -3'  obs
    cuucuaucagccuaguuacuaggcaguguaguuagcugauugccaaaaguaacaaucacuagccacacagccagguaaaaagguuuggaagauaccucaguggaaaccaga       exp
    ((((((..(((((..(((((.(((.((((.(((((.((((((..........))))))))))).)))).))).))))).))..)))))))))))...((.....))........  reads  mm  sample
    ..................cuaggcaguguaguuagcugau......................................................................  1     0   seq
    ..................cuaggcaguguaguuagcugauu.....................................................................  1     0   seq
    ...................uaggcaguguaguuagcug........................................................................  1     0   seq
    ...................uaggcaguguaguuagcuga.......................................................................  8     0   seq
    ...................uaggcaguguaguuagcugau......................................................................  11    0   seq
    ...................uaggcaguguaguuagcugauA.....................................................................  3     1   seq
    ...................uaggcagugCaguuagcugauu.....................................................................  1     1   seq
    ...................Caggcaguguaguuagcugauu.....................................................................  1     1   seq
    ...................uaggcaguguaguuagcugauu.....................................................................  85    0   seq
    ...................uaggcaguguaguuagcugauuA....................................................................  1     1   seq
    ...................uaggcaguguaguuagcugauuU....................................................................  3     1   seq
    ...................uaggcaguguaguuagcugauug....................................................................  10    0   seq
    ...................uaggcaguguaguuagcugauugA...................................................................  9     1   seq
    ...................uaggcaguguaguuagcugauugc...................................................................  2     0   seq
    ...................Caggcaguguaguuagcugauugc...................................................................  10    1   seq
    ....................aggcaguguaguuagcug........................................................................  5     0   seq
    ....................aggcaguguaguuagcugU.......................................................................  1     1   seq
    ....................aggcaguguaguuagcuga.......................................................................  26    0   seq
    ....................aggcaguUuaguuagcugau......................................................................  1     1   seq
    ....................aggcaguguaguuagcugau......................................................................  25    0   seq
    ....................aggcaguguaguuagcugauu.....................................................................  301   0   seq
    ....................aggcaguguaguuagcugauA.....................................................................  6     1   seq
    ....................aggcagGguaguuagcugauug....................................................................  1     1   seq
    ....................aggcaguguaguuagcugauuA....................................................................  1     1   seq
    ....................aggcaguguaguuagcugauuU....................................................................  4     1   seq
    ....................aggcaguguaguuagcugauug....................................................................  123   0   seq
    ....................aggcaguguaguuagcugauugc...................................................................  5535  0   seq
    ....................aggcagugGaguuagcugauugc...................................................................  2     1   seq
    ....................aggcaguguaguuagAugauugc...................................................................  2     1   seq
    ....................Uggcaguguaguuagcugauugc...................................................................  3     1   seq
    ....................aggcagAguaguuagcugauugc...................................................................  1     1   seq
    ....................aggcaguguaguuaUcugauugc...................................................................  1     1   seq
    ....................aCgcaguguaguuagcugauugc...................................................................  2     1   seq
```

Mature **Star**

cuucuaucagccuaguuacuaggcaguguaguuagcugauugccaaaaguaacaaucacuagccacacagccagguaaaaagguuuggaagauaccucaguggaaaccaga			
....................aggcaguguaguuagcuUauugc..	3	1	seq
....................aggcaguguaguuGgcugauugc..	1	1	seq
....................agUcaguguaguuagcugauugc..	1	1	seq
....................aggGaguguaguuagcugauugc..	1	1	seq
....................aggcagugAaguuagcugauugc..	3	1	seq
....................aggcaguguaguuagcugauugA..	43	1	seq
....................aggcaguguaguuagcugauuUc..	1	1	seq
....................aggcaUuguaguuagcugauugc..	1	1	seq
....................aggcaguguaguuagcugauuAc..	1	1	seq
....................aAgcaguguaguuagcugauugc..	1	1	seq
....................aggcaguguGguuagcugauugc..	1	1	seq
....................Gggcaguguaguuagcugauugc..	6	1	seq
....................aggcaguguaguuagcugUuugc..	1	1	seq
....................Cggcaguguaguuagcugauugc..	3	1	seq
....................aggcaguguaguuagUugauugc..	1	1	seq
....................aggcaguguaguuAcugauugc..	1	1	seq
....................aggcaguUuaguuagcugauugc..	1	1	seq
....................aggcagCguaguuagcugauugc..	4	1	seq
....................agAcaguguaguuagcugauugc..	1	1	seq
....................aggcGguguaguuagcugauugc..	1	1	seq
....................aggcaguguaguuagGugauugc..	1	1	seq
....................aggcagGguaguuagcugauugc..	3	1	seq
....................aggcagugCaguuagcugauugc..	2	1	seq
....................aggcaguguaguuagcugauugcG...	8	1	seq
....................aggcaguguaguuagcugauugcU...	755	1	seq
....................aggcaguguaguuagcugauugcA...	30	1	seq
....................aggcaguguaguuagcugauugcc...	39	0	seq
....................aggcaguguaguuagcugauugUc...	1	1	seq
....................aggcaguguaguuagcugauugccU..	3	1	seq
.........................guguaguuagcugauugc..	1	0	seq
....aaucacuagccacacagc..	1	0	seq
....aaucacuagccacacagccU......................................	1	1	seq
....aaucacuagccacacagcca......................................	2	0	seq
....aaucacuagccacacagccag.....................................	6	0	seq
....aaucacuagccacacagccagA....................................	1	1	seq
....aaucacuUgccacacagccagg....................................	1	1	seq
....aaucacuagccacacagccagg....................................	80	0	seq
....aaucacuagccacacagccagU....................................	3	1	seq
....aaucacuagccacacagccaggA...................................	12	1	seq
....aaucacuagccacacagccaggu...................................	7	0	seq
....aaucacuagccacacagccaggua..................................	1	0	seq
....aaucacuagccacacagccagguU..................................	2	1	seq
....aucacuagccacacagccag.....................................	1	0	seq
....aucacuagccacacagccagg....................................	1	0	seq
....aucacuagccacacagccaggA...................................	1	1	seq
....aucacuagccacacagccaggu...................................	3	0	seq
....aucacuagccacacagccagguU..................................	1	1	seq

Provisional ID : mun-novel10
Score total : 1.9
Score for star read(s) : -1.3
Score for read counts : 0
Score for mfe : 2.2
Score for randfold : 1.6
Score for cons. seed : -0.6
Total read count : 1076
Mature read count : 1076
Loop read count : 0
Star read count : 0

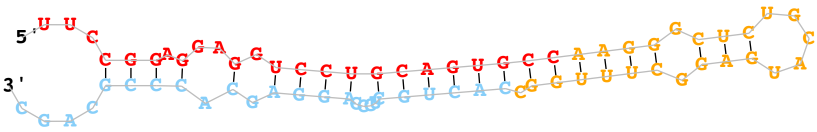

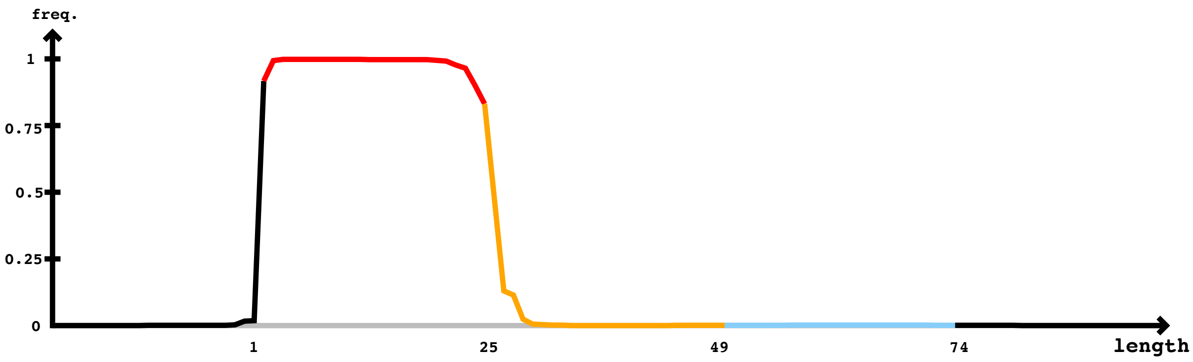

Mature **Star**

5'- cgaggaguuuggaggggaucuuccggaggagguccugcagugccaagggcucugcaugaggcuuuggccacugcucccaggagcacccgcagcauccccaacccucuggcuugu -3'	exp		
.((((.(((....((((((((..(((.(..(.((((((((((((((((.(((....))).)))))).))))))....)))).).)))).)).)))))))))))))........	reads	mm	sample
........uuggaggggaucuuccggaggag...	1	0	seq
.................aucuuccggaggagguccugcagugcc..	2	0	seq
..................ucuuccggaggagguccugcagug..	1	0	seq
..................ucuuccggaggagguccugcagugcc..	4	0	seq
..................ucuuUcggaggagguccugcagugcc..	1	1	seq
..................ucuuccggaggagguccugcagugccG...	1	1	seq
..................ucuuUcggaggagguccugcagugcca...	1	1	seq
..................ucuuccggaggagguccugcagugccU...	1	1	seq
..................ucuuccggaggagguccugcagugcca...	4	0	seq
..................ucuuUcggaggagguccugcagugccaa..	1	1	seq
..................ucuuccggaggagguccugcagugccaagg..	1	0	seq
...................cuuccggaggagguccugcagugcc..	1	0	seq
...................cuuccggaggagguccugcagugcca...	1	0	seq
....................uuccggaggagguccugc..	3	0	seq
....................uuUcggaggagguccugca...	1	1	seq
....................uuccggaggagguccugca...	2	0	seq
....................uuUcggaggagguccugcag..	1	1	seq
....................uuccggaggagguccugcaU..	2	1	seq
....................uuccggaggagguccugcaC..	1	1	seq
....................uuccggaggagguccugcaA..	2	1	seq
....................uuccggaggagguccugcag..	9	0	seq
....................uuccggaggagguccugcagu...	12	0	seq
....................uuccggaggaAguccugcagug..	1	1	seq
....................uuccggaggagguccugcagug..	66	0	seq
....................uuUcggaggagguccugcagug..	1	1	seq
....................uuccggaggagguccugcagugA...	2	1	seq
....................uuccggaggagguccugcagugU...	10	1	seq
....................uuUcggaggagguccugcagugc...	2	1	seq
....................uuccggaggagguccuUcagugc...	1	1	seq
....................uuccggaggaAguccugcagugc...	1	1	seq
....................uuccggaggagguccugcagugc...	59	0	seq
....................uuUcggaggagguccugcagugcc..	22	1	seq
....................uuccAgaggagguccugcagugcc..	4	1	seq
....................uuccggaggaggCccugcagugcc..	1	1	seq

```
                    Mature                                            Star
cgaggaguuuggaggggaucuuccggaggagguccugcagugccaagggcucugcaugaggcuuuggccacugcucccaggagcacccgcagcauccccaacccucuggcuugu
....................uuccggaggagguccugcagugcA......................................................................  3    1  seq
....................uuccggaggaAguccugcagugcc......................................................................  11   1  seq
....................uuccggaggagguccugcagugcc......................................................................  315  0  seq
....................uuccggaAgagguccugcagugcc......................................................................  1    1  seq
....................uuccggaggagguccugcagCgcc......................................................................  1    1  seq
....................uuccggaggaAguccugcagugcca.....................................................................  7    1  seq
....................uuccAgaggagguccugcagugcca.....................................................................  5    1  seq
....................uuccggaggagguccugcagugccG.....................................................................  9    1  seq
....................uuUcggaggagguccugcagugcca.....................................................................  19   1  seq
....................uuccggaggagguccugcagugcAa.....................................................................  2    1  seq
....................uuccggaggagguccugcagugccU.....................................................................  64   1  seq
....................uuccggaggagguccugcagAgcca.....................................................................  1    1  seq
....................uuccggaggagguccugcagugcca.....................................................................  221  0  seq
....................uuUcggaggagguccugcagugccaa....................................................................  3    1  seq
....................uuccggaggagguccugcagugccaC....................................................................  1    1  seq
....................uuccggaggagguccugcagugccaa....................................................................  5    0  seq
....................uuccggaggagguccugcagugccaU....................................................................  3    1  seq
....................uuccAgaggagguccugcagugccaag...................................................................  1    1  seq
....................uuccggaggagguccugcagugccaag...................................................................  76   0  seq
....................uuccggaggagguccugcagugccaaC...................................................................  1    1  seq
....................uuUcggaggagguccugcagugccaag...................................................................  2    1  seq
....................uuccggaggagguccugcaguUccaag...................................................................  1    1  seq
....................uuccggaggagguccugcagugccaaA...................................................................  1    1  seq
....................uuccggaggagguccugcagugccaagg..................................................................  6    0  seq
....................uuccggaggagguccugcagugccaagU..................................................................  6    1  seq
....................uuccggaggaAguccugcagugccaagg..................................................................  1    1  seq
....................uuccAgaggagguccugcagugccaagg..................................................................  1    1  seq
....................uuccggaggagguccugcagugccaaggg.................................................................  1    0  seq
....................uuUcggaggagguccugcagugccaagggc................................................................  2    1  seq
....................uuccggaggagguccugcagugccaagggcuc..............................................................  2    0  seq
.....................uccggaggagguccugcag..........................................................................  1    0  seq
.....................uccggaggagguccugcagu.........................................................................  1    0  seq
.....................uccggaggagguccugcagug........................................................................  1    0  seq
.....................uUcggaggagguccugcagugcc......................................................................  2    1  seq
.....................uccggaggagguccugcagugcc......................................................................  5    0  seq
.....................uccggaggagguccugcagugccU.....................................................................  7    1  seq
.....................uccggaggagguccugcagugccG.....................................................................  3    1  seq
.....................uccAgaggagguccugcagugcca.....................................................................  1    1  seq
.....................uUcggaggagguccugcagugcca.....................................................................  1    1  seq
.....................uccggaggagguccugcagugcca.....................................................................  38   0  seq
.....................uccggaggagguccugcagugccaU....................................................................  2    1  seq
.....................uccggaggagguccugcagugccaa....................................................................  2    0  seq
.....................uccggaggagguccugcagugccaaC...................................................................  1    1  seq
.....................uccggaggagguccugcagugccaag...................................................................  12   0  seq
.....................uccggaggaAguccugcagugccaag...................................................................  1    1  seq
.....................uccggaggagguccugcagugccaaU...................................................................  1    1  seq
.....................uccggaggagguccugcagugccaagg..................................................................  2    0  seq
.....................uccggaggagguccugcagugccaagU..................................................................  2    1  seq
......................ccggaggagguccugcagugcca.....................................................................  2    0  seq
......................ccggaggagguccugcagugccaag...................................................................  1    0  seq
......................ccggaggagguccugcagugccaagg..................................................................  1    0  seq
......................ccggaggagguccugcagugccaaggg.................................................................  1    0  seq
...............................................................uuggccacugcucccaggagcacccgc........................  1    0  seq
...........................................................................cccaggagcaGccgcagcauccccc..............  1    1  seq
```

Provisional ID	:	mun-miR-2188
Score total	:	5301.7
Score for star read(s)	:	3.9
Score for read counts	:	5291.1
Score for mfe	:	2.1
Score for randfold	:	1.6
Score for cons. seed	:	3
Total read count	:	10390
Mature read count	:	10042
Loop read count	:	1
Star read count	:	347

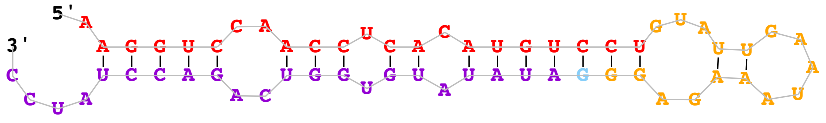

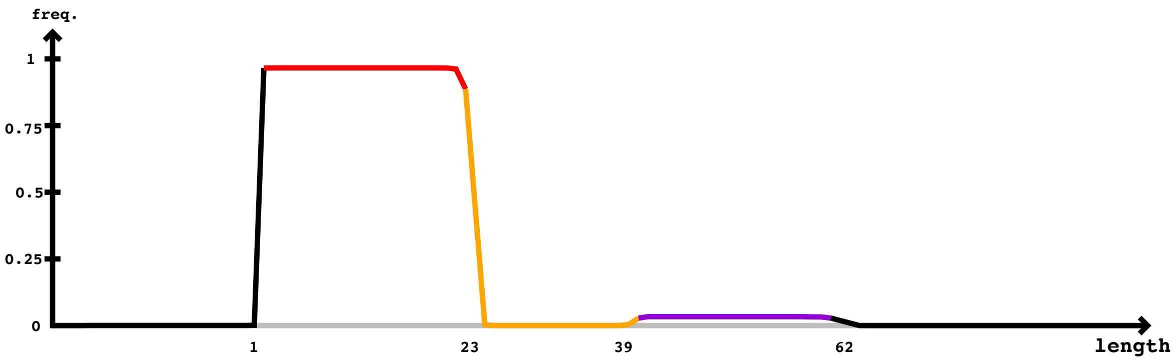


```
                Mature                                   Star
5'- ggaauaucagaccugugggaaagguccaaccucacauguccuguauugaauaaaagagggauauauguggucagaccuaucccacaggcccuguauucuuacucaagugcaga   -3'   obs
    ggaauaucagaccugugggaaagguccaaccucacauguccuguauugaauaaaagagggauauauguggucagaccuaucccacaggcccuguauucuuacucaagugcaga         exp
    ((((((.(((.((((((((.(((((..(((.((.(((((((...((.....))..))))))).)).))).))))).))))))))..)))))))))).................   reads   mm   sample
    ..aauaucagaccuguggga.............................................................................................   3       0    seq
    ...................aaagguccaaccucacauguc.........................................................................   2       0    seq
    ...................aaagguccaaccucacaugucc........................................................................   3       0    seq
    ...................Caagguccaaccucacauguccu.......................................................................   1       1    seq
    ....................aagguccaaccucacaugu..........................................................................   4       0    seq
    ....................aagguccaaccucacGuguc.........................................................................   1       1    seq
    ....................aagguccGaccucacauguc.........................................................................   1       1    seq
    ....................aagguccaaccucacauguc.........................................................................   36      0    seq
    ....................aGgguccaaccucacaugucc........................................................................   3       1    seq
    ....................Gagguccaaccucacaugucc........................................................................   6       1    seq
    ....................aagguccGaccucacaugucc........................................................................   5       1    seq
    ....................aagguccaaccucacauCucc........................................................................   1       1    seq
    ....................aagguccaaccucacaugucc........................................................................   750     0    seq
    ....................aaggGccaaccucacaugucc........................................................................   2       1    seq
    ....................aagguccaacUucacaugucc........................................................................   1       1    seq
    ....................aagguccaaccucacaugucA........................................................................   3       1    seq
    ....................aagguccaaccuGacaugucc........................................................................   1       1    seq
    ....................aagguccaaccucacaugucU........................................................................   2       1    seq
    ....................aaggAccaaccucacaugucc........................................................................   1       1    seq
    ....................Cagguccaaccucacaugucc........................................................................   1       1    seq
    ....................aagguccaaUcucacaugucc........................................................................   1       1    seq
    ....................aagguccaaccucacauguAc........................................................................   1       1    seq
    ....................aagguccaaccucacaugAcc........................................................................   1       1    seq
    ....................aaggGccaaccucacauguccu.......................................................................   12      1    seq
    ....................aagUuccaaccucacauguccu.......................................................................   2       1    seq
    ....................aagguccaaccucacauguccA.......................................................................   116     1    seq
    ....................aagguccaUccucacauguccu.......................................................................   1       1    seq
    ....................aagguGcaaccucacauguccu.......................................................................   1       1    seq
    ....................Cagguccaaccucacauguccu.......................................................................   2       1    seq
    ....................aagguccaaccuAacauguccu.......................................................................   2       1    seq
    ....................aagguccaaccucacauguAcu.......................................................................   2       1    seq
    ....................aagguccaacAucacauguccu.......................................................................   2       1    seq
    ....................aagguccaaccucacauguccC.......................................................................   3       1    seq
```

Mature **Star**

ggaauaucagaccugugggaaagguccaaccucacauguccuguauugaauaaaagagggauauauguggucagaccuaucccacaggcccuguauucuuacucaagugcaga			
....................aagguccaaccucaUauguccu...	3	1	seq
....................aagguccaaccAcacauguccu...	3	1	seq
....................aagAuccaaccucacauguccu...	2	1	seq
....................aagguccaaccucacauAuccu...	3	1	seq
....................aaggAccaaccucacauguccu...	3	1	seq
....................aagguccaaccuUacauguccu...	1	1	seq
....................aagguccaGccucacauguccu...	2	1	seq
....................aagguccaaccucacauguccu...	8643	0	seq
....................aagguccaaccucacaugucGu...	1	1	seq
....................aGgguccaaccucacauguccu...	35	1	seq
....................aagguccaaccucacauguccG...	43	1	seq
....................aagguccaaUcucacauguccu...	7	1	seq
....................aaggucGaaccucacauguccu...	2	1	seq
....................Gagguccaaccucacauguccu...	37	1	seq
....................aagguccaaAcucacauguccu...	1	1	seq
....................aagguAcaaccucacauguccu...	1	1	seq
....................aaggCccaaccucacauguccu...	2	1	seq
....................aagguccaaccucGcauguccu...	1	1	seq
....................aagguccaaccucaAauguccu...	2	1	seq
....................aagguccaaccucacaCguccu...	1	1	seq
....................aagguccaaccucacauUuccu...	1	1	seq
....................aagguccaaccCcacauguccu...	1	1	seq
....................aaggucAaaccucacauguccu...	3	1	seq
....................aagguccaaccucacauguUcu...	1	1	seq
....................aagguccGaccucacauguccu...	38	1	seq
....................aagguccCaccucacauguccu...	1	1	seq
....................aagguccaaccucacauCuccu...	1	1	seq
....................aagguccaacUucacauguccu...	1	1	seq
....................aagguccaaccucUcauguccu...	1	1	seq
....................aagguccaaccucacGuguccu...	2	1	seq
....................aaUguccaaccucacauguccu...	1	1	seq
....................aagguccaaccucacauguccug..	13	0	seq
....................aagguccaaccucacauguccuA..	2	1	seq
....................aagguccaaccucacauguccuU..	177	1	seq
....................aagguccaaccucacauguccuC..	4	1	seq
....................aagguccaaccucacauguccuCu...	3	1	seq
....................aagguccaaccucacauguccuAu...	4	1	seq
....................aagguccaaccucacauguccuUu...	19	1	seq
.....................agguccaaccucacauguccu...	3	0	seq
.....................Ggguccaaccucacauguccu...	1	1	seq
.....................agguccaaccucacauguccug..	1	0	seq
.....................agguccaaccucacauguccugu...	1	0	seq
....cuguauCgaauaaaagaggga..	1	1	seq
....gauauGuguggucagacc....................................	1	1	seq
....gauauGuguggucagaccu...................................	1	1	seq
....gauauauguggucagaccu...................................	5	0	seq
....gauauauguggucagaccua..................................	1	0	seq
....gauauauguggucagaccuau.................................	8	0	seq
....gauauGuguggucagaccuau.................................	3	1	seq
....gauauauguggucagaccuauU................................	2	1	seq
....gauauauguggucGgaccuauc................................	2	1	seq
....gauauGuguggucagaccuauc................................	7	1	seq
....gauauauguggucagaccuauc................................	12	0	seq
....auauauguggucagaccu...................................	1	0	seq
....auauauguggucagaccua..................................	2	0	seq
....auauGuguggucagaccuau.................................	13	1	seq
....auauauguggucagaccuau.................................	18	0	seq
....auauauguggucagaccuauc................................	15	0	seq
....auauauguggucagaccuauU................................	10	1	seq
....auauGuguggucagaccuauc................................	39	1	seq
....auauauguggucagaccuaucc...............................	2	0	seq
....auauauguggucagaccuaucA...............................	1	1	seq
....auauGuguggucagaccuaucc...............................	1	1	seq
....auauauguggucagaccuaucU...............................	146	1	seq
....uauauguggucagaccuauc................................	2	0	seq
....uauGuguggucagaccuauc................................	1	1	seq
....uauGuguggucagaccuaucc...............................	3	1	seq
....uauauguggucagaccuaucU...............................	42	1	seq
....uauauguggucagaccuaucc...............................	3	0	seq
....uauauguggucagaccuauccU..............................	1	1	seq

```
                           Mature                                             Star
ggaauaucagaccugugggaaagguccaaccucacauguccuguauugaauaaagagggauauauguggucagaccuaucccacaggcccuguauucuuacucaagugcaga
............................................................uauauguggucagaccuauccc..............................    2    0    seq
............................................................uauGuguggucagaccuauccc..............................    2    1    seq
............................................................uauauguggucagaccuaucccU.............................    1    1    seq
```

Provisional ID	:	mun-miR-199
Score total	:	36710.3
Score for star read(s)	:	3.9
Score for read counts	:	36699.9
Score for mfe	:	1.9
Score for randfold	:	1.6
Score for cons. seed	:	3
Total read count	:	71997
Mature read count	:	57702
Loop read count	:	4
Star read count	:	14291

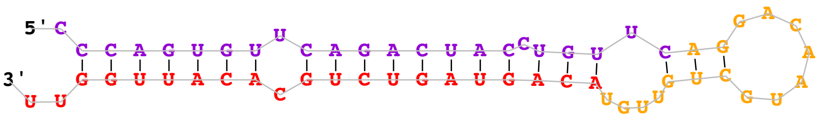

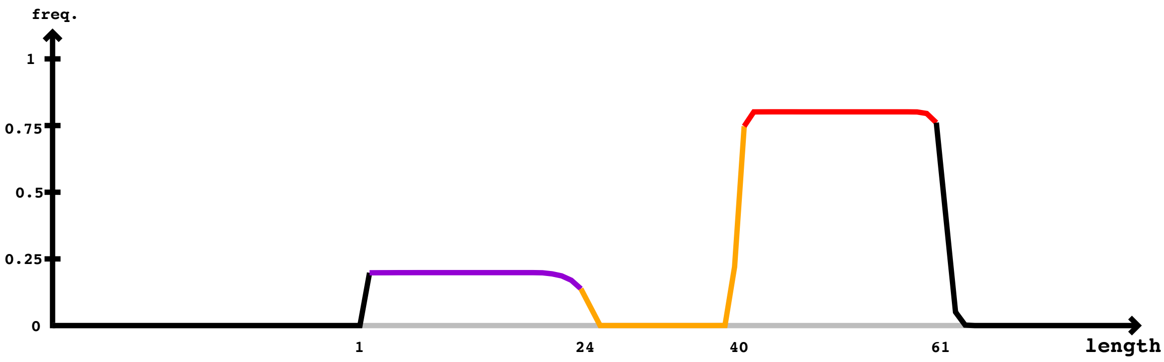

Star Mature

```
5'- auagacgcguccggagauccugcuccgucgccccaguguucagacuaccuguucaggacaaugcuguuguacaguagucugcacauugguuagacugggcaagggaaagca  -3'   obs
    auagacgcguccggagauccugcuccgucgccccaguguucagacuaccuguucaggacaaugcuguuguacaguagucugcacauugguuagacugggcaagggaaagca         exp
    ......((.(((((....)).((((.(((...(((((((.((((((((.(((.(((.......)))....))))))))))).)))))))...))).))))..)))...)).   reads   mm   sample
    ...............................cccaguguucagacuacc..............................................................   42      0    seq
    ...............................cccaguguucagacuaccu.............................................................   289     0    seq
    ...............................cccagugGucagacuaccu.............................................................   1       1    seq
    ...............................cccaguguuUagacuaccu.............................................................   1       1    seq
    ...............................cccaguguucagacuaccGg............................................................   1       1    seq
    ...............................cccaguguuUagacuaccug............................................................   1       1    seq
    ...............................cccaguguucagacuaccuU............................................................   16      1    seq
    ...............................cccagCguucagacuaccug............................................................   1       1    seq
    ...............................cccaguguucagacuaccuC............................................................   1       1    seq
    ...............................cccaguguucagacuaccug............................................................   521     0    seq
    ...............................cccagAguucagacuaccug............................................................   1       1    seq
    ...............................cccagugCucagacuaccug............................................................   1       1    seq
    ...............................cccagGguucagacuaccug............................................................   1       1    seq
    ...............................cccaguguucagacuCccug............................................................   1       1    seq
    ...............................cccagugAucagacuaccugu...........................................................   1       1    seq
    ...............................Uccaguguucagacuaccugu...........................................................   1       1    seq
    ...............................cccaguguucagacuacUugu...........................................................   1       1    seq
    ...............................cccaguCuucagacuaccugu...........................................................   1       1    seq
    ...............................cccaguguucagacCaccugu...........................................................   1       1    seq
    ...............................cccaguguucagacuaccugu...........................................................   1135    0    seq
    ...............................cccaguguucagacuaccugA...........................................................   21      1    seq
    ...............................Accaguguucagacuaccugu...........................................................   2       1    seq
    ...............................cccaguguucagacuaccugG...........................................................   1       1    seq
    ...............................Gccaguguucagacuaccugu...........................................................   2       1    seq
    ...............................cccaguguucagacuaccuAu...........................................................   1       1    seq
    ...............................cccagGguucagacuaccugu...........................................................   1       1    seq
    ...............................cccaguguucagacuaAcugu...........................................................   1       1    seq
    ...............................cccaguguuAagacuaccuguu..........................................................   1       1    seq
    ...............................Accaguguucagacuaccuguu..........................................................   1       1    seq
    ...............................cccaguguucagGcuaccuguu..........................................................   1       1    seq
    ...............................cccaguguucaCacuaccuguu..........................................................   1       1    seq
    ...............................Uccaguguucagacuaccuguu..........................................................   3       1    seq
    ...............................cccagAguucagacuaccuguu..........................................................   2       1    seq
```

```
                              Star                                   Mature
auagacgcguccggagauccugcuccgucgccccaguguucagacuaccuguucaggacaaugcuguuguacaguagucugcacauugguuagacugggcaagggaaagca
...............................cUcaguguucagacuaccuguu..........................................................  2     1  seq
...............................cccagGguucagacuaccuguu..........................................................  1     1  seq
...............................cccaguguucagacuaccuguu..........................................................  2248  0  seq
...............................cccaguguucGgacuaccuguu..........................................................  1     1  seq
...............................Gccaguguucagacuaccuguu..........................................................  3     1  seq
...............................cccagugCucagacuaccuguu..........................................................  1     1  seq
...............................cccaguguucagacuaccuguG..........................................................  2     1  seq
...............................cccaguguucagacuaccuguA..........................................................  39    1  seq
...............................cccaguguucagacuaAcuguu..........................................................  2     1  seq
...............................ccUaguguucagacuaccuguuc.........................................................  1     1  seq
...............................cccaguguucGgacuaccuguuc.........................................................  2     1  seq
...............................cccaUuguucagacuaccuguuc.........................................................  2     1  seq
...............................cccaguguucagaUuaccuguuc.........................................................  1     1  seq
...............................cccaguguucagacuaccuguuU.........................................................  1046  1  seq
...............................cccaguguucagaAuaccuguuc.........................................................  1     1  seq
...............................cccaguguucaUacuaccuguuc.........................................................  4     1  seq
...............................Accaguguucagacuaccuguuc.........................................................  7     1  seq
...............................cccagugCucagacuaccuguuc.........................................................  2     1  seq
...............................cccaguAuucagacuaccuguuc.........................................................  1     1  seq
...............................cccaguguucagacuaccugAuc.........................................................  1     1  seq
...............................cAcaguguucagacuaccuguuc.........................................................  1     1  seq
...............................cccaguguucagacuaccuguuc.........................................................  5265  0  seq
...............................cccaguguucagacAaccuguuc.........................................................  1     1  seq
...............................cccagugAucagacuaccuguuc.........................................................  1     1  seq
...............................Uccaguguucagacuaccuguuc.........................................................  5     1  seq
...............................cccaguguucagacuGccuguuc.........................................................  1     1  seq
...............................cccaguguucagGcuaccuguuc.........................................................  1     1  seq
...............................cccaguguucagacuaccuguuG.........................................................  41    1  seq
...............................cccagGguucagacuaccuguuc.........................................................  3     1  seq
...............................ccAaguguucagacuaccuguuc.........................................................  1     1  seq
...............................cccaguguucagacuaccCguuc.........................................................  1     1  seq
...............................cccaCuguucagacuaccuguuc.........................................................  1     1  seq
...............................cccagugGucagacuaccuguuc.........................................................  1     1  seq
...............................Gccaguguucagacuaccuguuc.........................................................  4     1  seq
...............................cccaguguuAagacuaccuguuc.........................................................  1     1  seq
...............................cccaguguucagacuaccuguuA.........................................................  842   1  seq
...............................cccaguguuGagacuaccuguuca........................................................  1     1  seq
...............................cccaguguucagacuaccuguuca........................................................  96    0  seq
...............................cccaguguucagacuaccuguucC........................................................  25    1  seq
...............................Uccaguguucagacuaccuguuca........................................................  1     1  seq
...............................cccaguguucagaAuaccuguuca........................................................  1     1  seq
...............................cccaguguucagacuaccuguuAa........................................................  5     1  seq
...............................cccaguguucagacuaccuguucU........................................................  2526  1  seq
...............................cccaguguucagacuaccuguucG........................................................  1     1  seq
...............................cccaguguucagacuaccuguucaA.......................................................  1     1  seq
................................ccaguguucagacuaccuguu..........................................................  2     0  seq
................................ccaguguucagacuaccuguuc.........................................................  9     0  seq
................................ccaguguucagacuaccuguuA.........................................................  1     1  seq
................................ccaguguucagacuaccuguuU.........................................................  2     1  seq
................................ccaguguucagacuaccuguuca........................................................  1     0  seq
................................ccaguguucagacuaccuguucU........................................................  2     1  seq
.................................caguguucagacuaccuguuc.........................................................  5     0  seq
.................................caguguucagacuaccuguucU........................................................  2     1  seq
..................................aguguucagacuaccugu...........................................................  1     0  seq
..................................aguguucagacuaccuguu..........................................................  4     0  seq
..................................aguguucagacuaccuguuc.........................................................  9     0  seq
....................................uguucagacCaccuguuc.........................................................  1     1  seq
....................................uguucagacuaccuguuc.........................................................  1     0  seq
....................................uguucaUacuaccuguuc.........................................................  1     1  seq
...................................................uucaggacaaugcuguuguacaguagu.................................  1     0  seq
.....................................................caggacaaugcuguuguac.......................................  1     0  seq
.....................................................caggacaaugcuguuguacagu....................................  3     0  seq
.....................................................caggacaaugcuguuguacagua...................................  1     0  seq
......................................................aggacaaugcuguuguacagua...................................  1     0  seq
...................................................................uCuacaguagucugcacauugguu....................  3     1  seq
...................................................................ugGacaguagucugcacauugguu....................  1     1  seq
....................................................................gGacaguagucugcacauugguu....................  5     1  seq
....................................................................Cuacaguagucugcacauugguu....................  4     1  seq
....................................................................guacaguagucugcacauugguu....................  2     0  seq
....................................................................gGacaguagucugcacauugguua...................  3     1  seq
```

```
                              Star                                 Mature
auagacgcguccggagauccugcuccgucgccccaguguucagacuaccuguucaggacaaugcuguuguacaguagucugcacauugguuagacugggcaagggaaagca
.....................................................................uacaguagucugcacauu........................   3       0   seq
.....................................................................uacaguagGcugcacauug.......................   1       1   seq
.....................................................................uacaguagucugcacauGg.......................   1       1   seq
.....................................................................uacaguagucugcacauug.......................   10      0   seq
.....................................................................uacaguagucugcacauugg......................   230     0   seq
.....................................................................Gacaguagucugcacauugg......................   1       1   seq
.....................................................................uacaguagucugcacauugA......................   1       1   seq
.....................................................................uacaUuagucugcacauugg......................   1       1   seq
.....................................................................uacagCagucugcacauugg......................   1       1   seq
.....................................................................uacaguagucugcacauugU......................   7       1   seq
.....................................................................uacaguagucugcacauuggu.....................   1042    0   seq
.....................................................................uacaguagucugcacauuUgu.....................   1       1   seq
.....................................................................uacaguagucugcacauugUu.....................   1       1   seq
.....................................................................uacaguagucugcacauuggC.....................   10      1   seq
.....................................................................Cacaguagucugcacauuggu.....................   2       1   seq
.....................................................................uacagAagucugcacauuggu.....................   1       1   seq
.....................................................................uacaguagucugcacauuggA.....................   17      1   seq
.....................................................................Gacaguagucugcacauuggu.....................   1       1   seq
.....................................................................uacGguagucugcacauuggu.....................   1       1   seq
.....................................................................uacagGagucugcacauuggu.....................   1       1   seq
.....................................................................uacaguagucCgcacauuggu.....................   1       1   seq
.....................................................................uacaguUgucugcacauuggu.....................   2       1   seq
.....................................................................uacaguaguUugcacauuggu.....................   1       1   seq
.....................................................................uacaguagucugcacauuggG.....................   4       1   seq
.....................................................................uacaguagucugcacauugCu.....................   2       1   seq
.....................................................................uacaguagucuCcacauuggu.....................   1       1   seq
.....................................................................uacaguaCucugcacauugguu....................   1       1   seq
.....................................................................uacaguagucugGacauugguu....................   2       1   seq
.....................................................................uacaguaguAugcacauugguu....................   1       1   seq
.....................................................................uacaguCgucugcacauugguu....................   2       1   seq
.....................................................................uacagAagucugcacauugguu....................   5       1   seq
.....................................................................uacaguagucugcacauugUuu....................   4       1   seq
.....................................................................uacaguagucugcacauugguA....................   1252    1   seq
.....................................................................uacaguagGcugcacauugguu....................   4       1   seq
.....................................................................uacaUuagucugcacauugguu....................   1       1   seq
.....................................................................uacaguagucugcUcauugguu....................   1       1   seq
.....................................................................uacaguagucuCcacauugguu....................   1       1   seq
.....................................................................uacaguagucugUacauugguu....................   1       1   seq
.....................................................................uacaguagCcugcacauugguu....................   2       1   seq
.....................................................................uacaguagucCgcacauugguu....................   1       1   seq
.....................................................................uacaguagucugcacauuggAu....................   2       1   seq
.....................................................................uacaAuagucugcacauugguu....................   1       1   seq
.....................................................................uacaguagucugcacauuUguu....................   5       1   seq
.....................................................................uacaguagucugcacauugCuu....................   5       1   seq
.....................................................................uacaguagucuUcacauugguu....................   2       1   seq
.....................................................................uaAaguagucugcacauugguu....................   1       1   seq
.....................................................................uGcaguagucugcacauugguu....................   1       1   seq
.....................................................................uacaguaUucugcacauugguu....................   1       1   seq
.....................................................................Cacaguagucugcacauugguu....................   29      1   seq
.....................................................................uacaguagucugcacauugguG....................   13      1   seq
.....................................................................uacaguaguGugcacauugguu....................   1       1   seq
.....................................................................uacaguagucugAacauugguu....................   2       1   seq
.....................................................................uacagCagucugcacauugguu....................   1       1   seq
.....................................................................Gacaguagucugcacauugguu....................   5       1   seq
.....................................................................uacaguagucugcacaCugguu....................   1       1   seq
.....................................................................Aacaguagucugcacauugguu....................   4       1   seq
.....................................................................uacaguagucuAcacauugguu....................   2       1   seq
.....................................................................uacaguagucugcacauuggGu....................   10      1   seq
.....................................................................uacaguagucugcacauuggCu....................   3       1   seq
.....................................................................uacaguagucugcacauugguu....................   11353   0   seq
.....................................................................uacagGagucugcacauugguu....................   1       1   seq
.....................................................................uacaguagucugcacauugguC....................   119     1   seq
.....................................................................Aacaguagucugcacauugguua...................   1       1   seq
.....................................................................uacaguagucugcacauugguua...................   637     0   seq
.....................................................................uacaguagucugAacauugguua...................   2       1   seq
.....................................................................uacaguagucugcacaGugguua...................   1       1   seq
.....................................................................uacaguagucugcacauugguuG...................   30      1   seq
.....................................................................Cacaguagucugcacauugguua...................   2       1   seq
.....................................................................uacaguagucugcacauuggAua...................   1       1   seq
.....................................................................uacaguagucugcacauugguAa...................   126     1   seq
```

Star **Mature**

```
auagacgcguccggagauccugcuccgucgccccaguguucagacuaccuguucaggacaaugcuguuguacaguagucugcacauugguuagacugggcaagggaaagca
.....................................................................uacaguagucugcacauAgguua....................	1	1	seq
.....................................................................uacaguagucugcacauugguuC....................	24	1	seq
.....................................................................uacaguagucugcacauugguCa....................	3	1	seq
.....................................................................uacagGagucugcacauugguua....................	1	1	seq
.....................................................................uacaguagucugcacauugguuU....................	579	1	seq
.....................................................................uacaguagucugcacauugguGa....................	13	1	seq
.....................................................................uacaguagucugcacaCugguua....................	1	1	seq
.....................................................................uacaguagucugcacauugAuua....................	1	1	seq
.....................................................................uacaguagucugcacauugguuag...................	3	0	seq
.....................................................................uacaguagucugcacauugguuaU...................	164	1	seq
.....................................................................uacaguagucugcacauugguuaC...................	6	1	seq
.....................................................................uacaguagucugcacauugguuaA...................	65	1	seq
.....................................................................uacaguagucugcacauugguuaAa..................	3	1	seq
.....................................................................uacaguagucugcacauugguuaUa..................	2	1	seq
......................................................................acaguagucugcacauug.......................	16	0	seq
......................................................................acaguagucugcacauugU......................	13	1	seq
......................................................................Ucaguagucugcacauugg......................	1	1	seq
......................................................................acaguagucugcaAauugg......................	1	1	seq
......................................................................acaguagucugcacauugg......................	152	0	seq
......................................................................acaguagucugcacauugA......................	1	1	seq
......................................................................acaguagucugcacauuggC.....................	2	1	seq
......................................................................acaguagucGgcacauuggu.....................	1	1	seq
......................................................................acagGagucugcacauuggu.....................	1	1	seq
......................................................................acaguagucugcacauuggA.....................	13	1	seq
......................................................................acaguaguAugcacauuggu.....................	1	1	seq
......................................................................acaguagAcugcacauuggu.....................	1	1	seq
......................................................................acaguagucugcacauuggG.....................	3	1	seq
......................................................................acaguagucugcacauGggu.....................	1	1	seq
......................................................................Ucaguagucugcacauuggu.....................	1	1	seq
......................................................................acaguagucugcacauuggu.....................	1171	0	seq
......................................................................acaguagucuUcacauuggu.....................	1	1	seq
......................................................................acaguagucugcacUuuggu.....................	1	1	seq
......................................................................acaguagucugcacauuggGu....................	10	1	seq
......................................................................acagAagucugcacauugguu....................	7	1	seq
......................................................................acagGagucugcacauugguu....................	11	1	seq
......................................................................acaguagucCgcacauugguu....................	4	1	seq
......................................................................acaguagucugcacauuggAu....................	2	1	seq
......................................................................acaguagucugcacCuugguu....................	1	1	seq
......................................................................acaguagucuUcacauugguu....................	5	1	seq
......................................................................acaguagucAgcacauugguu....................	1	1	seq
......................................................................acaguagucugcacaAugguu....................	1	1	seq
......................................................................acaguagucugcacauuCguu....................	1	1	seq
......................................................................acaguagucugcacauugguG....................	32	1	seq
......................................................................Ccaguagucugcacauugguu....................	8	1	seq
......................................................................acaguagucugcacUuugguu....................	4	1	seq
......................................................................acaguagucuCcacauugguu....................	2	1	seq
......................................................................acaguagucugcaGauugguu....................	2	1	seq
......................................................................acaguaUucugcacauugguu....................	3	1	seq
......................................................................acGguagucugcacauugguu....................	1	1	seq
......................................................................acaguaguAugcacauugguu....................	4	1	seq
......................................................................acaguagucugcacauuUguu....................	3	1	seq
......................................................................acaguagucugcacauugUuu....................	2	1	seq
......................................................................Ucaguagucugcacauugguu....................	5	1	seq
......................................................................acagCagucugcacauugguu....................	3	1	seq
......................................................................acaguagucugcacauugguu....................	24532	0	seq
......................................................................acaguagucugcacaCugguu....................	1	1	seq
......................................................................acaguagucugcacauugguC....................	13	1	seq
......................................................................acaguagucugcCcauugguu....................	1	1	seq
......................................................................acaguagucugcacauugCuu....................	16	1	seq
......................................................................acaguagucugcGcauugguu....................	1	1	seq
......................................................................aAaguagucugcacauugguu....................	3	1	seq
......................................................................acaguagucugcaUauugguu....................	2	1	seq
......................................................................acaguaguUugcacauugguu....................	1	1	seq
......................................................................acaguagGcugcacauugguu....................	2	1	seq
......................................................................acaguagucugcUcauugguu....................	1	1	seq
......................................................................acaguagucugcacauuggCu....................	7	1	seq
......................................................................acaguagucugcaAauugguu....................	6	1	seq
......................................................................acaguagucugcacauCgguu....................	6	1	seq
......................................................................acaguagucuAcacauugguu....................	10	1	seq
......................................................................aUaguagucugcacauugguu....................	5	1	seq
```

```
                                             Star                                     Mature
auagacgcguccggagauccugcuccgucgccccaguguucagacuaccuguucaggacaaugcuguuguacaguagucugcacauugguuagacugggcaagggaaagca
......................................................................acaCuagucugcacauugguu....................	5	1	seq
......................................................................acaguaAucugcacauugguu....................	3	1	seq
......................................................................acaguagAcugcacauugguu....................	5	1	seq
......................................................................acaUuagucugcacauugguu....................	5	1	seq
......................................................................acaguagucugcacGuugguu....................	4	1	seq
......................................................................Gcaguagucugcacauugguu....................	20	1	seq
......................................................................acaguagucugcacauuAguu....................	3	1	seq
......................................................................acaguagucugcacauAgguu....................	2	1	seq
......................................................................acaguGgucugcacauugguu....................	4	1	seq
......................................................................acaguaguGugcacauugguu....................	1	1	seq
......................................................................acaguagCcugcacauugguu....................	2	1	seq
......................................................................acaguagucugcacauugAuu....................	1	1	seq
......................................................................acaguagucugcacauugguA....................	1153	1	seq
......................................................................acaguagucugUacauugguu....................	6	1	seq
......................................................................acaAuagucugcacauugguu....................	2	1	seq
......................................................................acaguagucugUacauugguua...................	2	1	seq
......................................................................acaguUgucugcacauugguua...................	2	1	seq
......................................................................Gcaguagucugcacauugguua...................	4	1	seq
......................................................................acaguaUucugcacauugguua...................	2	1	seq
......................................................................acaguagGcugcacauugguua...................	3	1	seq
......................................................................acaguagucugcacauuggCua...................	2	1	seq
......................................................................acaguagucuCcacauugguua...................	1	1	seq
......................................................................acagGagucugcacauugguua...................	2	1	seq
......................................................................acaguagucugcacauugguuC...................	47	1	seq
......................................................................acaguagucuAcacauugguua...................	2	1	seq
......................................................................acaguagucugcacauuCguua...................	2	1	seq
......................................................................acaguGgucugcacauugguua...................	1	1	seq
......................................................................acaguagucugcacauugUuua...................	1	1	seq
......................................................................acaguaAucugcacauugguua...................	1	1	seq
......................................................................acaguagucugcacauugguCa...................	16	1	seq
......................................................................acaguagucugcacauugguuG...................	214	1	seq
......................................................................aUaguagucugcacauugguua...................	1	1	seq
......................................................................aAaguagucugcacauugguua...................	2	1	seq
......................................................................acagAagucugcacauugguua...................	1	1	seq
......................................................................acaAuagucugcacauugguua...................	1	1	seq
......................................................................acaguagucugAacauugguua...................	1	1	seq
......................................................................acaguagCcugcacauugguua...................	2	1	seq
......................................................................acaguagucuUcacauugguua...................	3	1	seq
......................................................................acaguagucugcacauuggAua...................	1	1	seq
......................................................................acagCagucugcacauugguua...................	1	1	seq
......................................................................acaguagucugcaUauugguua...................	1	1	seq
......................................................................acaguagucugGacauugguua...................	1	1	seq
......................................................................acaguagucCgcacauugguua...................	2	1	seq
......................................................................acaguagucugcacauugguAa...................	337	1	seq
......................................................................acaguagucugcacauugguGa...................	22	1	seq
......................................................................acaUuagucugcacauugguua...................	1	1	seq
......................................................................acaguagucugcacauuggGua...................	1	1	seq
......................................................................acaguagucugcaGauugguua...................	2	1	seq
......................................................................acaguagucugcacaCugguua...................	1	1	seq
......................................................................acaguagucugcacauugguuU...................	692	1	seq
......................................................................Ccaguagucugcacauugguua...................	5	1	seq
......................................................................acaguagucugcacauugguua...................	6330	0	seq
......................................................................acaguagucugcacGuugguua...................	1	1	seq
......................................................................acaguagucugcacauugguuaA..................	1741	1	seq
......................................................................acaguagucugcacauugguuUg..................	3	1	seq
......................................................................acaguagucugcacauugguuaU..................	1067	1	seq
......................................................................acaguagucugcacauugguuag..................	6	0	seq
......................................................................acaguagucugcacauugguuaC..................	9	1	seq
......................................................................acaguagucugcacauugguuaCa.................	1	1	seq
......................................................................acaguagucugcacauugguuaUa.................	16	1	seq
......................................................................acaguagucugcacauugguuaAa.................	66	1	seq
......................................................................acaguagucugcacauugguuaga.................	2	0	seq
.......................................................................caguagucugcacauugg......................	6	0	seq
.......................................................................caguagucugcacauuggA.....................	2	1	seq
.......................................................................caguagucugcaAauuggu.....................	1	1	seq
.......................................................................caguagucugcacauuggu.....................	188	0	seq
.......................................................................Gaguagucugcacauuggu.....................	1	1	seq
.......................................................................caguagucugUacauuggu.....................	1	1	seq
.......................................................................caAuagucugcacauugguu....................	1	1	seq
.......................................................................caguagucugAacauugguu....................	1	1	seq
```

Star Mature

```
auagacgcguccggagauccugcuccgucgccccaguguucagacuaccuguucaggacaaugcuguuguacaguagucugcacauugguuagacugggcaagggaaagca
.......................................................................Gaguagucugcacauugguu.................... 4 1 seq
.......................................................................caguagucuUcacauugguu.................... 1 1 seq
.......................................................................caguaCucugcacauugguu.................... 1 1 seq
.......................................................................caguagucugcacauugguu.................... 2275 0 seq
.......................................................................Aaguagucugcacauugguu.................... 1 1 seq
.......................................................................caguagAcugcacauugguu.................... 1 1 seq
.......................................................................caguagucugcacauugguG.................... 5 1 seq
.......................................................................caguagucugcacauCgguu.................... 1 1 seq
.......................................................................caguagucugcacauuggCu.................... 1 1 seq
.......................................................................caguagucugcacauuUguu.................... 1 1 seq
.......................................................................caguagucuAcacauugguu.................... 1 1 seq
.......................................................................Uaguagucugcacauugguu.................... 4 1 seq
.......................................................................caguagucugcacauugguA.................... 90 1 seq
.......................................................................caguagucugcacauuggCua................... 1 1 seq
.......................................................................caguagucugcacauugguua................... 689 0 seq
.......................................................................caguagucugcacauugguAa................... 25 1 seq
.......................................................................Uaguagucugcacauugguua................... 2 1 seq
.......................................................................Aaguagucugcacauugguua................... 1 1 seq
.......................................................................caguagucugcacauugguuC................... 4 1 seq
.......................................................................caguagucugcacauugguCa................... 3 1 seq
.......................................................................caguagucugcacauugguuG................... 1 1 seq
.......................................................................caguagucugcacauugguuU................... 79 1 seq
.......................................................................caguagGcugcacauugguua................... 1 1 seq
.......................................................................caguagucugcacauugguuaA.................. 281 1 seq
.......................................................................caguagucugcacauugguuaC.................. 15 1 seq
.......................................................................caguagucugcacauugguuaU.................. 151 1 seq
.......................................................................caguagucugcacauugguuaUa................. 9 1 seq
.......................................................................caguagucugcacauugguuaCa................. 1 1 seq
.......................................................................caguagucugcacauugguuaAa................. 29 1 seq
........................................................................aguagucugcacauugguu.................... 3 0 seq
........................................................................aguagucugcacauugguua................... 4 0 seq
........................................................................aguagucugcacauugguuag.................. 2 0 seq
........................................................................aguagucugcacauugguuaU.................. 1 1 seq
.........................................................................guagucugcacauugguu.................... 7 0 seq
.........................................................................guagucugcacauugguua................... 3 0 seq
.........................................................................guagucugcacauugguuaA.................. 1 1 seq
```

Provisional ID	:	mun-let-7a-2
Score total	:	1049.3
Score for star read(s)	:	3.9
Score for read counts	:	1039.2
Score for mfe	:	1.6
Score for randfold	:	1.6
Score for cons. seed	:	3
Total read count	:	2050
Mature read count	:	2036
Loop read count	:	0
Star read count	:	14

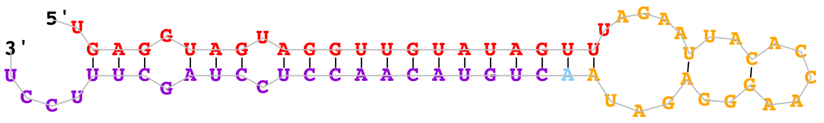

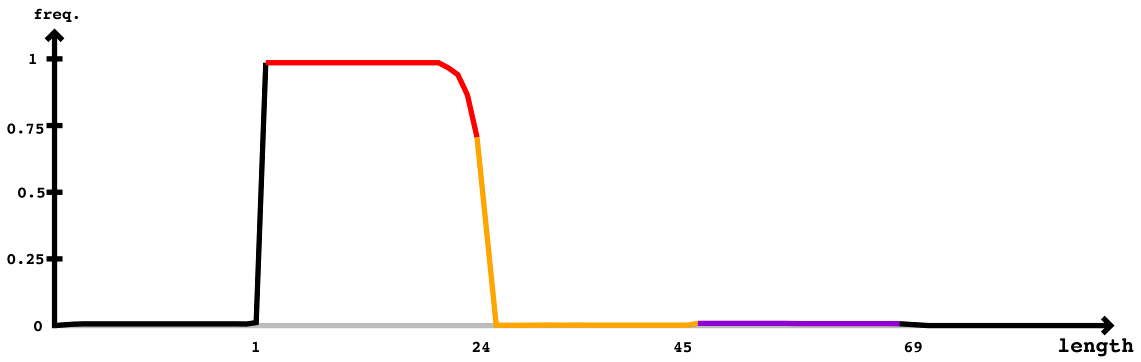

Mature **Star**

```
5'- uugacugcaugcauccagguugagguaguagguuguauaguuuagaauuacaccaagggagauaacuguacaaccuccuagcuuuccuugggucuugcacagaacggc  -3'   obs
    uugacugcaugcauccagguugagguaguagguuguauaguuuagaauuacaccaagggagauaacuguacaaccuccuagcuuuccuugggucuugcacagaacggc         exp
    .....((((.(.(((((((..(((.(((.((((((((((((.....(..(.....)..)...)))))))))))).))).)))..)))))))).)))).........   reads   mm   sample
    uugacugcaugcauccag..........................................................................................   1       0    seq
    uugacugcaugcauccaggu........................................................................................   10      0    seq
    .ugacugcaugcauccaggu........................................................................................   2       0    seq
    ...................uugagguaguagAuuguauagu...................................................................   6       1    seq
    ...................uugagguaguagAuuguauaguu..................................................................   5       1    seq
    ....................ugagguaguagguuguauU.....................................................................   37      1    seq
    ....................ugagguaguagguuguaAa.....................................................................   1       1    seq
    ....................ugaggGaguagguuguaua.....................................................................   1       1    seq
    ....................ugagguagGagguuguauag....................................................................   1       1    seq
    ....................ugagguaguagguAguauag....................................................................   1       1    seq
    ....................ugagguGguagguuguauag....................................................................   1       1    seq
    ....................ugaggAaguagguuguauag....................................................................   2       1    seq
    ....................ugaggGaguagguuguauag....................................................................   3       1    seq
    ....................ugagguaguagguuguauaA....................................................................   27      1    seq
    ....................ugagguaguagguuguauaU....................................................................   17      1    seq
    ....................ugagguaguagguuguauUg....................................................................   1       1    seq
    ....................ugagguagAagguuguauagu...................................................................   1       1    seq
    ....................Agagguaguagguuguauagu...................................................................   1       1    seq
    ....................ugagguaguaggCuguauagu...................................................................   1       1    seq
    ....................ugagguaCuagguuguauagu...................................................................   1       1    seq
    ....................ugagguaguagguuguauaUu...................................................................   8       1    seq
    ....................ugGgguaguagguuguauagu...................................................................   2       1    seq
    ....................ugaggCaguagguuguauagu...................................................................   8       1    seq
    ....................ugUgguaguagguuguauagu...................................................................   1       1    seq
    ....................ugagguUguagguuguauagu...................................................................   1       1    seq
    ....................ugagguaguaggGuguauagu...................................................................   2       1    seq
    ....................ugagguaguagguuguGuagu...................................................................   2       1    seq
    ....................ugagUuaguagguuguauagu...................................................................   1       1    seq
    ....................ugagguaguagguuguaAagu...................................................................   1       1    seq
    ....................ugagguaguagguuCuauagu...................................................................   2       1    seq
    ....................ugagguaguagguuUuauagu...................................................................   1       1    seq
    ....................ugagguaguaUguuguauagu...................................................................   3       1    seq
    ....................ugagguaguagguugAauagu...................................................................   2       1    seq
```

```
                    Mature                                        Star
uugacugcaugcauccagguugagguaguagguuguauaguuagaauuacaccaagggagauaacuguacaaccuccuagcuuuccuugggucuugcacagaacggc
....................uCagguaguagguuguauagu.................................................................. 1 1 seq
....................ugagguaguaggAuguauagu.................................................................. 1 1 seq
....................ugagguaguagguuguauagG.................................................................. 2 1 seq
....................ugagguaguagguAguauagu.................................................................. 1 1 seq
....................Ggagguaguagguuguauagu.................................................................. 6 1 seq
....................ugaggGaguagguuguauagu.................................................................. 14 1 seq
....................ugagguaguagguuguauagA.................................................................. 62 1 seq
....................ugagguaguagguuguauagC.................................................................. 2 1 seq
....................ugagguaguagguuguauaAu.................................................................. 4 1 seq
....................ugaUguaguagguuguauagu.................................................................. 1 1 seq
....................Cgagguaguagguuguauagu.................................................................. 2 1 seq
....................ugagguaguUgguuguauagu.................................................................. 1 1 seq
....................ugagguagGagguuguauagu.................................................................. 1 1 seq
....................ugagguaguaAguuguauagu.................................................................. 1 1 seq
....................uUagguaguagguuguauagu.................................................................. 3 1 seq
....................ugaggAaguagguuguauagu.................................................................. 9 1 seq
....................ugagguaguagguuguaCagu.................................................................. 1 1 seq
....................ugagguagCagguuguauagu.................................................................. 2 1 seq
....................ugagguagAagguuguauaguu................................................................. 3 1 seq
....................uUagguaguagguuguauaguu................................................................. 5 1 seq
....................ugagguaUuagguuguauaguu................................................................. 3 1 seq
....................ugagguaguUgguuguauaguu................................................................. 2 1 seq
....................ugaCguaguagguuguauaguu................................................................. 1 1 seq
....................ugagguaguaggCuguauaguu................................................................. 11 1 seq
....................ugaUguaguagguuguauaguu................................................................. 5 1 seq
....................ugagguaguagguuguauaAuu................................................................. 1 1 seq
....................Ggagguaguagguuguauaguu................................................................. 17 1 seq
....................ugagguaguagguuguUuaguu................................................................. 2 1 seq
....................ugagguaguagguuguaCaguu................................................................. 11 1 seq
....................Agagguaguagguuguauaguu................................................................. 18 1 seq
....................ugCgguaguagguuguauaguu................................................................. 3 1 seq
....................ugagguaguaggAuguauaguu................................................................. 4 1 seq
....................ugagguaguaUguuguauaguu................................................................. 2 1 seq
....................ugagguaCuagguuguauaguu................................................................. 1 1 seq
....................ugagguaguaCguuguauaguu................................................................. 3 1 seq
....................ugGgguaguagguuguauaguu................................................................. 5 1 seq
....................ugagguaguagguuCuauaguu................................................................. 3 1 seq
....................ugagguaguCgguuguauaguu................................................................. 1 1 seq
....................ugagguaguGgguuguauaguu................................................................. 6 1 seq
....................ugagguaguagguCguauaguu................................................................. 3 1 seq
....................uAagguaguagguuguauaguu................................................................. 2 1 seq
....................ugagguagCagguuguauaguu................................................................. 6 1 seq
....................ugagguGguagguuguauaguu................................................................. 10 1 seq
....................ugagguaguagguuguauagGu................................................................. 1 1 seq
....................ugagguaguagguuguauaguC................................................................. 3 1 seq
....................ugagCuaguagguuguauaguu................................................................. 1 1 seq
....................ugagguaguagguuguaGaguu................................................................. 1 1 seq
....................ugagguaguagguuguauagCu................................................................. 4 1 seq
....................ugagguaguagguugAauaguu................................................................. 1 1 seq
....................ugagguaguagguuAuauaguu................................................................. 2 1 seq
....................ugaggGaguagguuguauaguu................................................................. 43 1 seq
....................ugagguaguagCuuguauaguu................................................................. 6 1 seq
....................ugagguaguaggGuguauaguu................................................................. 14 1 seq
....................ugUgguaguagguuguauaguu................................................................. 2 1 seq
....................ugagguaguagguuguauaCuu................................................................. 2 1 seq
....................ugaggAaguagguuguauaguu................................................................. 32 1 seq
....................ugagguaguagguuguauagAu................................................................. 3 1 seq
....................ugagguaguagguuguauaUuu................................................................. 7 1 seq
....................ugagguUguagguuguauaguu................................................................. 1 1 seq
....................ugagguaguagguugGauaguu................................................................. 3 1 seq
....................ugagguaguagguugCauaguu................................................................. 5 1 seq
....................ugagguaguagguuguauaguG................................................................. 5 1 seq
....................ugagguaguagguuguauCguu................................................................. 1 1 seq
....................ugagguagGagguuguauaguu................................................................. 9 1 seq
....................ugagguaguagguuguGuaguu................................................................. 2 1 seq
....................ugagguaguagguuguaAaguu................................................................. 2 1 seq
....................ugaggCaguagguuguauaguu................................................................. 23 1 seq
....................ugagguaguagguuUuauaguu................................................................. 5 1 seq
....................Cgagguaguagguuguauaguu................................................................. 13 1 seq
....................ugagUuaguagguuguauaguu................................................................. 4 1 seq
```

Mature Star

```
uugacugcaugcauccagguugagguaguagguuguauaguuuagaauuacaccaagggagauaacuguacaaccuccuagcuuuccuugggucuugcacagaacggc
....................ugagguaguGgguuguauaguuu.................................................................	2	1	seq
....................ugaAguaguagguuguauaguuu.................................................................	1	1	seq
....................Agagguaguagguuguauaguuu.................................................................	3	1	seq
....................ugagguagGagguuguauaguuu.................................................................	1	1	seq
....................ugagguaguagguCguauaguuu.................................................................	1	1	seq
....................ugagguGguagguuguauaguuu.................................................................	4	1	seq
....................ugagguaguagguuguauaUuuu.................................................................	1	1	seq
....................ugagguaguagguuguauUguuu.................................................................	1	1	seq
....................ugagguaguagguuguauagCuu.................................................................	3	1	seq
....................ugagguaguagguuguauaguAu.................................................................	28	1	seq
....................ugagguagCagguuguauaguuu.................................................................	2	1	seq
....................ugagguaguagguuguauaguuA.................................................................	1139	1	seq
....................ugagguaguagguuguauCguuu.................................................................	2	1	seq
....................ugagguaguagguuguauaguuC.................................................................	50	1	seq
....................ugagguaguaggGuguauaguuu.................................................................	4	1	seq
....................Cgagguaguagguuguauaguuu.................................................................	1	1	seq
....................ugGgguaguagguuguauaguuu.................................................................	1	1	seq
....................ugagguaguagguuUuauaguuu.................................................................	2	1	seq
....................ugagguaguagguGguauaguuu.................................................................	1	1	seq
....................ugagguagAagguuguauaguuu.................................................................	2	1	seq
....................ugagguaguagguuguauaguCu.................................................................	5	1	seq
....................ugagguaguagguugCauaguuu.................................................................	1	1	seq
....................ugagguaguagguuguauaguuG.................................................................	79	1	seq
....................uAagguaguagguuguauaguuu.................................................................	1	1	seq
....................ugaggGaguagguuguauaguuu.................................................................	14	1	seq
....................Ggagguaguagguuguauaguuu.................................................................	3	1	seq
....................ugagguaguagguuguauaguGu.................................................................	3	1	seq
....................ugaUguaguagguuguauaguuu.................................................................	5	1	seq
....................ugagguaguagguuCuauaguuu.................................................................	3	1	seq
....................ugagguaguaUguuguauaguuu.................................................................	2	1	seq
....................ugaggCaguagguuguauaguuu.................................................................	6	1	seq
....................ugagguaguagguuguauagAuu.................................................................	2	1	seq
....................ugagUuaguagguuguauaguuu.................................................................	1	1	seq
....................ugaggAaguagguuguauaguuu.................................................................	10	1	seq
....................ugagguaguagguuguauaguAua................................................................	1	1	seq
....................ugagguaguagAuuguauaguuua................................................................	34	1	seq
....................ugagguaguagguuguauaguuuC................................................................	4	1	seq
....................ugagguaguagguuguauaguuAa................................................................	32	1	seq
....................ugagguaguagguuguauaguuGa................................................................	2	1	seq
....................ugaggCaguagguuguauaguuuag...............................................................	1	1	seq
....................ugagguaguagguuguauaguuuagaauuac.........................................................	1	0	seq
....................ugagguaguagguuguauaguuuagaauuacacU......................................................	1	1	seq
................................................uacaccaagggagauaacuguacaaccuccuagcuuucc.....................	1	0	seq
.................................................acaccaagggagauaacuguacaacc.................................	1	0	seq
....................................................ccaagggagauaacuguacaaccu................................	1	0	seq
................................................................acuguacaaccuccuagcuuuU......................	1	1	seq
.................................................................cuguacaaccuccuagcuuucU.....................	4	1	seq
.................................................................cuguacaaccuccuagcuuuccu....................	8	0	seq
.................................................................cuguacaaccuccuagcuuuccA....................	1	1	seq
```

Provisional ID	:	mun-miR-383
Score total	:	54.4
Score for star read(s)	:	3.9
Score for read counts	:	44
Score for mfe	:	1.9
Score for randfold	:	1.6
Score for cons. seed	:	3
Total read count	:	98
Mature read count	:	94
Loop read count	:	0
Star read count	:	4

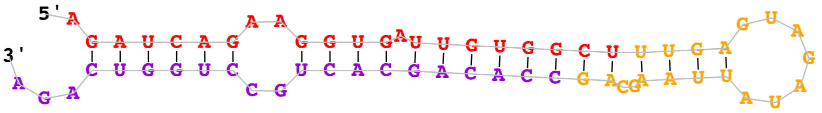

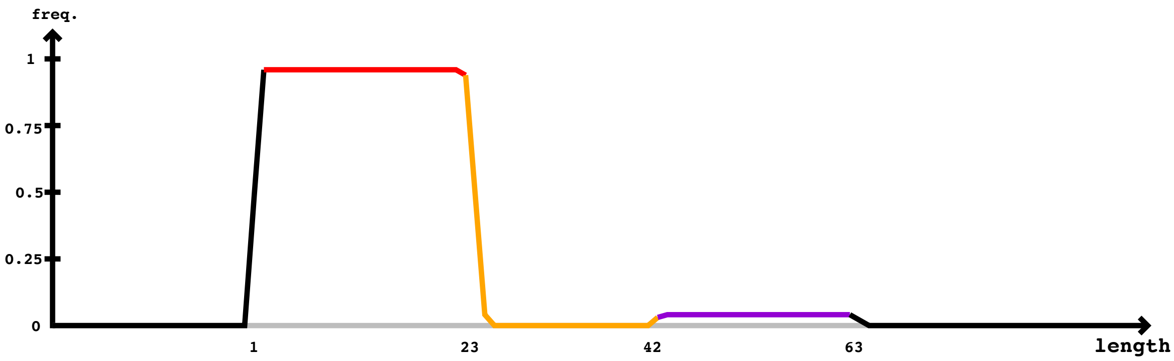

Mature Star

```
5'- ccucaagucacuugcuccucagaucagaaggugauuguggcuuugaguagauauuaagcagccacagcacugccuggucagaaagagcaaguguacuagccuuuuacuucuu   -3'    obs
    ccucaagucacuugcuccucagaucagaaggugauuguggcuuugaguagauauuaagcagccacagcacugccuggucagaaagagcaaguguacuagccuuuuacuucuu          exp
    .....(((((((((((.((.((((((..((((.((((((((((((.......))))..))))))))))))..)))))).))..)))))))).))).................   reads   mm   sample
    ...................cagaucagaaggugauuguggc.......................................................................   1   0   seq
    ...................cagaucagaaggGgauuguggc.......................................................................   1   1   seq
    ...................cagaucagaaggugauuguggcA......................................................................   1   1   seq
    ...................cagaucagaaggugauuguggcu......................................................................   32   0   seq
    ...................cagaucagaaggugauuguggcG......................................................................   2   1   seq
    ...................cagaucagaaggugauuguggcuu.....................................................................   10   0   seq
    ...................cagaucagaaggugauuguggcuCu....................................................................   1   1   seq
    ....................agaucagaaggugauuguggcu......................................................................   35   0   seq
    ....................agaucagaaggugauuguggcuu.....................................................................   6   0   seq
    ....................agaucagaaggugauuguggcuA.....................................................................   2   1   seq
    ....................agaucagaaggugauuguggcAuu....................................................................   1   1   seq
    ....................agaucagaaggugauuguggcuuu....................................................................   1   0   seq
    ....................agaucagaaggugauuguggcuAu....................................................................   1   1   seq
    .............................................................ccacagcacugUcuggucaga..............................   1   1   seq
    .............................................................ccacagcacugccuggucaga..............................   1   0   seq
    .............................................................ccacagcacugccuggucagaU.............................   1   1   seq
    ..............................................................cacagcacugccuggucagaU.............................   1   1   seq
```

Provisional ID	:	mun-novel1
Score total	:	16
Score for star read(s)	:	3.9
Score for read counts	:	6.2
Score for mfe	:	1.3
Score for randfold	:	1.6
Score for cons. seed	:	3
Total read count	:	24
Mature read count	:	21
Loop read count	:	0
Star read count	:	3

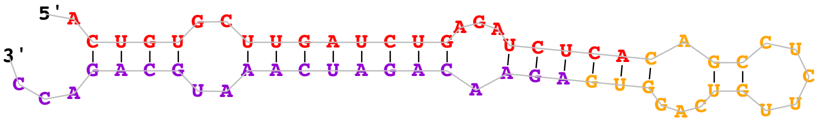

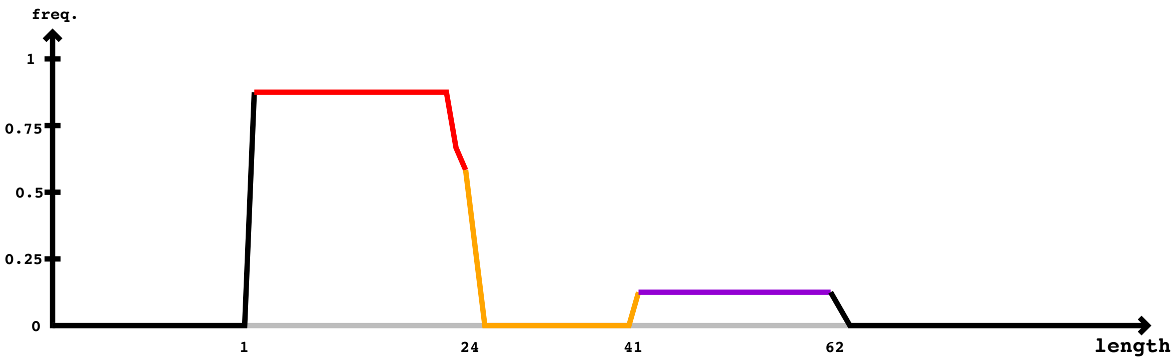

Mature **Star**

```
5'- acuuugagguccucuaaggacugugcuugaucugagaucucacagccucuugucaggugagaacagaucaaaugcagaccccugacaccucuuacacccuuggcagcagcug   -3'    obs
    acuuugagguccucuaaggacugugcuugaucugagaucucacagccucuugucaggugagaacagaucaaaugcagaccccugacaccucuuacacccuuggcagcagcug          exp
    .....(((((..((...((.((((..((((((((...((((((.((.....))...)))))).))))))))..)))).))...)).))))))..........(((....))).   reads   mm   sample
    ...................acugugcuugaucugagaucG........................................................................   1       1    seq
    ...................acugugcuugaucugagaucu........................................................................   4       0    seq
    ...................acugugcuugaucugagaucuc.......................................................................   2       0    seq
    ...................acugugcuugaucugagaucuca......................................................................   9       0    seq
    ...................acugugcuugaucugagaucucU......................................................................   5       1    seq
    ...........................................................agaacagaucaaaugcagacc................................   3       0    seq
```

Provisional ID : mun-novel46
Score total : 0.6
Score for star read(s) : -1.3
Score for read counts : -0.8
Score for mfe : 1.8
Score for randfold : 1.6
Score for cons. seed : -0.6
Total read count : 10
Mature read count : 10
Loop read count : 0
Star read count : 0

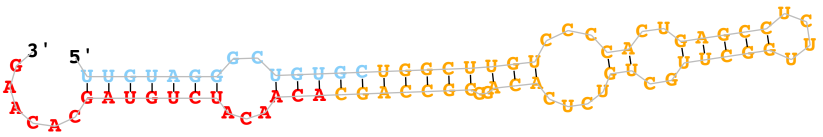

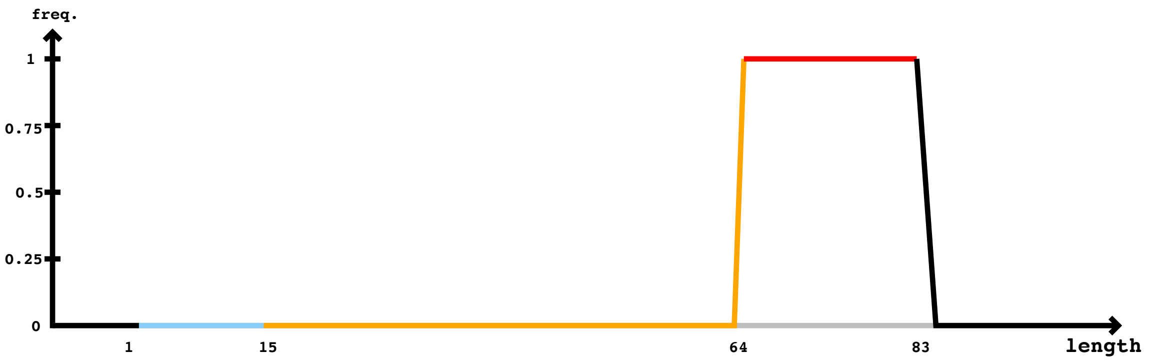

Star Mature

```
5'- uaggguuuuguagggcugugcuggcuuguccccacugagccucuuggcuugcugucucacaggggccagcacaacaucuguagcacaagcccagugugauguacucacg   -3'    exp
    ..((((((((((((..(((((((((((((...((..(((((....)))))..))....)))..))))))))))...)))...))).))))))...((((.....)))).    reads    mm    sample
    ......................................................................acaacaucuguagcacaag....................    10       0     seq
```

Provisional ID	:	mun-miR-181a
Score total	:	90079.4
Score for star read(s)	:	3.9
Score for read counts	:	90069.9
Score for mfe	:	1
Score for randfold	:	1.6
Score for cons. seed	:	3
Total read count	:	176680
Mature read count	:	175570
Loop read count	:	0
Star read count	:	1110

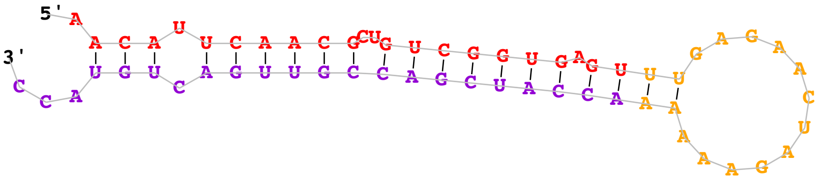

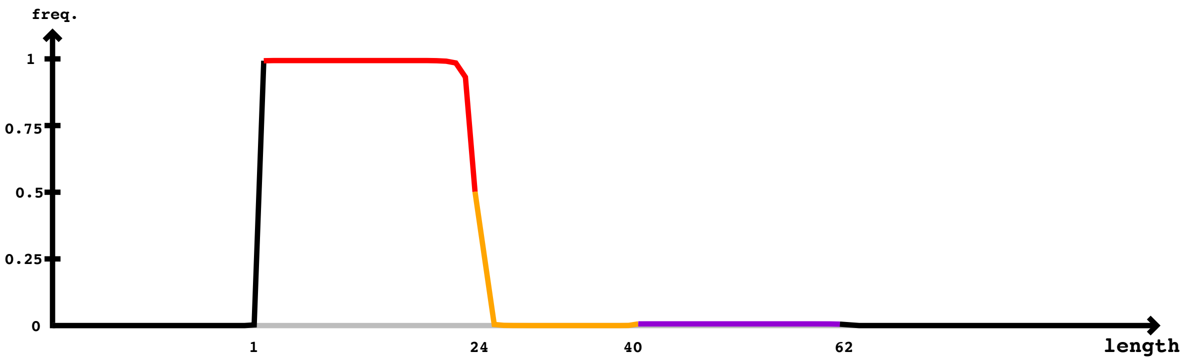

Mature **Star**

```
5'- aucuuuggauagcuucagugaacauucaacgcugucggugaguuugagaacuagaaaaaaccaucgaccguugacuguaccuugagguuuaucacaaccccucuguuaaaugg   -3'   obs
    aucuuuggauagcuucagugaacauucaacgcugucggugaguuugagaacuagaaaaaaccaucgaccguugacuguaccuugagguuuaucacaaccccucuguuaaaugg          exp
    ....((((((((((((((.(.(((.((((((..(((((((.((((............))))))))))))))))).))).).)))))).))))).)))................   reads   mm   sample
    ..................uCaacauucaacgcugucggugag.......................................................................   1       1    seq
    ..................Ggaacauucaacgcugucggugagu......................................................................   1       1    seq
    ..................uCaacauucaacgcugucggugagu......................................................................   1       1    seq
    ...................gaacauucaacgcugucg............................................................................   1       0    seq
    ...................gaacauucaacgcugucggu..........................................................................   1       0    seq
    ...................Caacauucaacgcugucggug.........................................................................   1       1    seq
    ...................gaacauucaacgcugucggug.........................................................................   10      0    seq
    ...................gaacaCucaacgcugucgguga........................................................................   1       1    seq
    ...................gaacauucaacgcugucggugU........................................................................   12      1    seq
    ...................gaacauucaacgcugucgguga........................................................................   85      0    seq
    ...................gaacauucaacgcugucggugG........................................................................   17      1    seq
    ...................gaacauucaacgcugucggugaU.......................................................................   6       1    seq
    ...................Caacauucaacgcugucggugag.......................................................................   6       1    seq
    ...................gaacauucaacgcugucggugag.......................................................................   230     0    seq
    ...................gaacaCucaacgcugucggugag.......................................................................   1       1    seq
    ...................gaacauucaacgcugucggugaA.......................................................................   2       1    seq
    ...................gaacauucaacgcugucggGgag.......................................................................   1       1    seq
    ...................gaacauucaacgcugucggAgag.......................................................................   1       1    seq
    ...................gaacauucaacgcugucggugagu......................................................................   42      0    seq
    ...................Caacauucaacgcugucggugagu......................................................................   5       1    seq
    ...................gaacauucaacgcugucggugaguuuU...................................................................   1       1    seq
    ....................aacauucaacgcugucgA...........................................................................   1       1    seq
    ....................aacauucaacgcugucgg...........................................................................   109     0    seq
    ....................aacauucaacgcugucgU...........................................................................   1       1    seq
    ....................aacauAcaacgcugucggu..........................................................................   1       1    seq
    ....................aacauucaacgAugucggu..........................................................................   1       1    seq
    ....................aacauucaacgcugGcggu..........................................................................   1       1    seq
    ....................aacauucaacgcugucgCu..........................................................................   2       1    seq
    ....................aacauucaaUgcugucggu..........................................................................   1       1    seq
    ....................aacauucaacgcugucggA..........................................................................   2       1    seq
    ....................aacauucaacgcugucggG..........................................................................   3       1    seq
    ....................aacauucaacgcugucggu..........................................................................   286     0    seq
    ....................aacauuUaacgcugucggu..........................................................................   1       1    seq
```

```
                    Mature                                      Star
aucuuuggauagcuucagugaacauucaacgcugucggugaguuugagaacuagaaaaaaccaucgaccguugacuguaccuugagguuuaucacaaccccucuguuaaaugg
....................aacauucaacCcugucggug.........................................................................   1      1   seq
....................aacauucaacgcugucgguA.........................................................................   14     1   seq
....................aacauucaacgcugucgguU.........................................................................   7      1   seq
....................aacauucaacgAugucggug.........................................................................   1      1   seq
....................aacauucaacgcuCucggug.........................................................................   1      1   seq
....................aaAauucaacgcugucggug.........................................................................   1      1   seq
....................aacCuucaacgcugucggug.........................................................................   1      1   seq
....................aacauucaacgUugucggug.........................................................................   3      1   seq
....................aacauucaacgcugucgCug.........................................................................   1      1   seq
....................aacauucaacgcuUucggug.........................................................................   1      1   seq
....................aacauucaacgcugucggGg.........................................................................   9      1   seq
....................aacauucaacgcugucggug.........................................................................   1125   0   seq
....................aacauucaacgcugucggAg.........................................................................   2      1   seq
....................aacauucaacgcugAcggug.........................................................................   1      1   seq
....................aacauucaacgcugucggCg.........................................................................   5      1   seq
....................aacauucaacgcugGcggug.........................................................................   2      1   seq
....................aacGuucaacgcugucggug.........................................................................   1      1   seq
....................aacauucGacgcugucggug.........................................................................   1      1   seq
....................aacauucaacgGugucgguga........................................................................   1      1   seq
....................aaAauucaacgcugucgguga........................................................................   1      1   seq
....................aacauucaaAgcugucgguga........................................................................   1      1   seq
....................aacauucaacgcugucgUuga........................................................................   2      1   seq
....................aaUauucaacgcugucgguga........................................................................   1      1   seq
....................aacauucaaUgcugucgguga........................................................................   2      1   seq
....................aacauucaacgUugucgguga........................................................................   15     1   seq
....................aacauCcaacgcugucgguga........................................................................   1      1   seq
....................aacauucaacgcugucggAga........................................................................   6      1   seq
....................aacauucaacgcugucgguga........................................................................   6698   0   seq
....................aacauucaacgcuguUgguga........................................................................   2      1   seq
....................Cacauucaacgcugucgguga........................................................................   1      1   seq
....................aacauucaacgcugucAguga........................................................................   2      1   seq
....................aacauucaacgcugCcgguga........................................................................   1      1   seq
....................aacauucaacgcugucggGga........................................................................   28     1   seq
....................aacauucaacgcugucUguga........................................................................   2      1   seq
....................Gacauucaacgcugucgguga........................................................................   5      1   seq
....................aacauucaacgcugucgguUa........................................................................   1      1   seq
....................aacauucaacAcugucgguga........................................................................   2      1   seq
....................aacauucaacgcCgucgguga........................................................................   1      1   seq
....................aacauucaacgcugucggugG........................................................................   1035   1   seq
....................Uacauucaacgcugucgguga........................................................................   1      1   seq
....................aacauucaacCcugucgguga........................................................................   2      1   seq
....................aacauucaacgcugAcgguga........................................................................   2      1   seq
....................aacauucaCcgcugucgguga........................................................................   1      1   seq
....................aacauucaacgcugucgCuga........................................................................   1      1   seq
....................aacauucaacgcugucggugU........................................................................   1406   1   seq
....................aacauuUaacgcugucgguga........................................................................   2      1   seq
....................aacauucaacUcugucgguga........................................................................   2      1   seq
....................aacauucaacgcuguAgguga........................................................................   3      1   seq
....................aacauucaacgcugucgguAa........................................................................   20     1   seq
....................aacGuucaacgcugucgguga........................................................................   2      1   seq
....................aacauucaacgcugucggCga........................................................................   6      1   seq
....................aacauucaacgcugGcgguga........................................................................   2      1   seq
....................aacaCucaacgcugucgguga........................................................................   2      1   seq
....................aacauucaacgcugucgAuga........................................................................   3      1   seq
....................aacauucaacgcugucgguAag.......................................................................   4      1   seq
....................aUcauucaacgcugucggugag.......................................................................   8      1   seq
....................aacauucaacgcugucggugaA.......................................................................   1260   1   seq
....................aacauucaacgcuguUggugag.......................................................................   14     1   seq
....................aacauucaacgcugucgguUag.......................................................................   12     1   seq
....................aacauucaacgcugucgCugag.......................................................................   2      1   seq
....................aacauucaacgcuguGggugag.......................................................................   4      1   seq
....................aacaGucaacgcugucggugag.......................................................................   1      1   seq
....................aacauucaacgcugCcggugag.......................................................................   4      1   seq
....................aacauucaacAcugucggugag.......................................................................   8      1   seq
....................aacauucaaUgcugucggugag.......................................................................   9      1   seq
....................aacauucaCcgcugucggugag.......................................................................   2      1   seq
....................aacauucaacgcCgucggugag.......................................................................   8      1   seq
....................aacauucaacgcugucgAugag.......................................................................   4      1   seq
....................aaGauucaacgcugucggugag.......................................................................   2      1   seq
....................aacauucaacgcugucggugGg.......................................................................   72     1   seq
```

Mature Star

aucuuuggauagcuucagugaacauucaacgcugucggugaguuugagaacuagaaaaaaccaucgaccguugacuguaccuugagguuuaucacaaccccucuguuaaaugg			
....................aacauucaacgAugucggugag...	13	1	seq
....................aacauucaacgcugucUgugag...	9	1	seq
....................aacaAucaacgcugucggugag...	2	1	seq
....................aacauucCacgcugucggugag...	1	1	seq
....................Gacauucaacgcugucggugag...	66	1	seq
....................Cacauucaacgcugucggugag...	13	1	seq
....................aacUuucaacgcugucggugag...	3	1	seq
....................aacauucaacgcugucgguCag...	3	1	seq
....................aacauucaacgcugucggugag...	72685	0	seq
....................aacauucaaGgcugucggugag...	4	1	seq
....................aacauucaacgcuAucggugag...	8	1	seq
....................aacauucaacgcuguAggugag...	9	1	seq
....................aacauucaUcgcugucggugag...	1	1	seq
....................aacaCucaacgcugucggugag...	8	1	seq
....................aacauucaacgcugucAgugag...	10	1	seq
....................aaUauucaacgcugucggugag...	4	1	seq
....................aacauucaacgcuCucggugag...	9	1	seq
....................aacauucaacgcugucgUugag...	19	1	seq
....................aacauucaacgcugucggugaC...	24	1	seq
....................aacauucaaAgcugucggugag...	20	1	seq
....................aacauuUaacgcugucggugag...	7	1	seq
....................aacauucaacgcugucggGgag...	118	1	seq
....................aacauucaacgcuUucggugag...	16	1	seq
....................aacauuAaacgcugucggugag...	9	1	seq
....................aacauucaacUcugucggugag...	9	1	seq
....................aacauucaacgcugucggAgag...	24	1	seq
....................aacauucaacCcugucggugag...	6	1	seq
....................aacauucaacgcugucggugaU...	986	1	seq
....................Uacauucaacgcugucggugag...	15	1	seq
....................aacauCcaacgcugucggugag...	4	1	seq
....................aacauucUacgcugucggugag...	1	1	seq
....................aacauucaacgcugGcggugag...	6	1	seq
....................aacauucaacgUugucggugag...	60	1	seq
....................aacauucGacgcugucggugag...	14	1	seq
....................aacauucaacgcugucCgugag...	3	1	seq
....................aacauucaacgcugucggCgag...	38	1	seq
....................aacauucaacgcAgucggugag...	1	1	seq
....................aacauuGaacgcugucggugag...	4	1	seq
....................aacauAcaacgcugucggugag...	3	1	seq
....................aacauucaacgcGgucggugag...	1	1	seq
....................aGcauucaacgcugucggugag...	5	1	seq
....................aacauucaGcgcugucggugag...	4	1	seq
....................aacGuucaacgcugucggugag...	10	1	seq
....................aaAauucaacgcugucggugag...	8	1	seq
....................aacauucaacgcugAcggugag...	5	1	seq
....................aacauucaacgcugucggugUg...	8	1	seq
....................aacauucaacgcugucggugaAu..	79	1	seq
....................aacauuUaacgcugucggugagu..	9	1	seq
....................aaUauucaacgcugucggugagu..	7	1	seq
....................aacauucaacgcugucgguAagu..	4	1	seq
....................aacauucaacgcugucgguUagu..	12	1	seq
....................aacauucaacgcugucggugCgu..	2	1	seq
....................aacauucaacgcugucAgugagu..	8	1	seq
....................aCcauucaacgcugucggugagu..	1	1	seq
....................Uacauucaacgcugucggugagu..	19	1	seq
....................aacauucaacgcuguAggugagu..	7	1	seq
....................aacauucaGcgcugucggugagu..	9	1	seq
....................aUcauucaacgcugucggugagu..	4	1	seq
....................aaGauucaacgcugucggugagu..	5	1	seq
....................aacauCcaacgcugucggugagu..	4	1	seq
....................aacauuAaacgcugucggugagu..	12	1	seq
....................aacauuGaacgcugucggugagu..	6	1	seq
....................aacauucaacgcuguUggugagu..	16	1	seq
....................aacauucUacgcugucggugagu..	1	1	seq
....................aacauucaacAcugucggugagu..	17	1	seq
....................aacauucaacgcugucggugagC..	22	1	seq
....................aacauucaacUcugucggugagu..	11	1	seq
....................aacauucaacgcCgucggugagu..	8	1	seq
....................aacauucaacgcugucggAgagu..	31	1	seq
....................aacauucaacgcugucggugaCu..	3	1	seq

Mature **Star**

```
aucuuuggauagcuucagugaacauucaacgcugucggugaguuugagaacuagaaaaaaccaucgaccguugacuguaccuugagguuuaucacaaccccucuguuaaaugg
....................aacauucaacgcugucggugUgu......................................................................	6	1	seq
....................aacauucaaUgcugucggugagu......................................................................	15	1	seq
....................Gacauucaacgcugucggugagu......................................................................	74	1	seq
....................aacGuucaacgcugucggugagu......................................................................	4	1	seq
....................aacauucaacgcugucCgugagu......................................................................	4	1	seq
....................aacauucaacgGugucggugagu......................................................................	1	1	seq
....................aacauucaacgcugucggugagA......................................................................	3159	1	seq
....................aacauucaacgcugAcggugagu......................................................................	9	1	seq
....................aacauucaaAgcugucggugagu......................................................................	10	1	seq
....................aacaCucaacgcugucggugagu......................................................................	8	1	seq
....................aacauucaacgcugucUgugagu......................................................................	18	1	seq
....................aacauucaacgcugucggugagG......................................................................	126	1	seq
....................aacauucaacgcugucggugagu......................................................................	80846	0	seq
....................aacauucaacgcugGcggugagu......................................................................	8	1	seq
....................aaAauucaacgcugucggugagu......................................................................	7	1	seq
....................aacauucaacgAugucggugagu......................................................................	11	1	seq
....................aacauucGacgcugucggugagu......................................................................	14	1	seq
....................aacauucaacgcuUucggugagu......................................................................	14	1	seq
....................aacauucaacgcGgucggugagu......................................................................	2	1	seq
....................aacauAcaacgcugucggugagu......................................................................	6	1	seq
....................aacaGucaacgcugucggugagu......................................................................	1	1	seq
....................aacCuucaacgcugucggugagu......................................................................	1	1	seq
....................aacauucaacgcugucgCugagu......................................................................	5	1	seq
....................aacauucaacgcugCcggugagu......................................................................	10	1	seq
....................aacauucaacgcAgucggugagu......................................................................	5	1	seq
....................aacauucaacgUugucggugagu......................................................................	86	1	seq
....................aacauucaacgcugucgAugagu......................................................................	3	1	seq
....................aacauucaacgcuguGggugagu......................................................................	4	1	seq
....................aacauucaacgcugucggugGgu......................................................................	11	1	seq
....................aacauucaacgcugucggugaUu......................................................................	25	1	seq
....................aacauucaUcgcugucggugagu......................................................................	3	1	seq
....................aacUuucaacgcugucggugagu......................................................................	3	1	seq
....................aacauucaacgcugucggGgagu......................................................................	120	1	seq
....................aacauucaacCcugucggugagu......................................................................	5	1	seq
....................aacauucaacgcuCucggugagu......................................................................	7	1	seq
....................aacauucaacgcugucgUugagu......................................................................	11	1	seq
....................aacauucaacgcugucggCgagu......................................................................	41	1	seq
....................Cacauucaacgcugucggugagu......................................................................	18	1	seq
....................aGcauucaacgcugucggugagu......................................................................	6	1	seq
....................aacauucaacgcuAucggugagu......................................................................	5	1	seq
....................aacauucaacgcuCucggugaguu.....................................................................	1	1	seq
....................aacauucaacgcugucggugGguu.....................................................................	1	1	seq
....................aacauucaacgcugucggugUguu.....................................................................	1	1	seq
....................aacauucaacgcugucggugaguu.....................................................................	2659	0	seq
....................aacauucaacgcugucggugagCu.....................................................................	1	1	seq
....................aacauucaacgcugucggugaguA.....................................................................	46	1	seq
....................aacauucaacUcugucggugaguu.....................................................................	1	1	seq
....................aacauucCacgcugucggugaguu.....................................................................	1	1	seq
....................aacauucaacAcugucggugaguu.....................................................................	1	1	seq
....................Gacauucaacgcugucggugaguu.....................................................................	2	1	seq
....................aaUauucaacgcugucggugaguu.....................................................................	1	1	seq
....................aacauucaacgcugucggugaguG.....................................................................	7	1	seq
....................aacGuucaacgcugucggugaguu.....................................................................	1	1	seq
....................aacauucaacgUugucggugaguu.....................................................................	1	1	seq
....................aacauucaacgcugucgguCaguu.....................................................................	1	1	seq
....................aacauucaacgcugCcggugaguu.....................................................................	1	1	seq
....................aacauucaacgcugucggGgaguu.....................................................................	4	1	seq
....................aacauucaacgcuguAggugaguu.....................................................................	1	1	seq
....................aacauucaacgcugucggCgaguu.....................................................................	2	1	seq
....................aacauucaacgcugucggugagGu.....................................................................	4	1	seq
....................aacauucaaAgcugucggugaguu.....................................................................	1	1	seq
....................aacauucaacgcugucgCugaguu.....................................................................	1	1	seq
....................aacauucaacgcugucggugCguu.....................................................................	1	1	seq
....................aacauucaacgcugucggugaguC.....................................................................	2	1	seq
....................Cacauucaacgcugucggugaguu.....................................................................	1	1	seq
....................aacauucaacgcugucggugaAuu.....................................................................	1	1	seq
....................aacauucaacgcuguUggugaguu.....................................................................	1	1	seq
....................aacauucaacgcugucggugagAu.....................................................................	11	1	seq
....................aacauucaacgcugucggugagAuu....................................................................	1	1	seq
....................aacauucaacgcugucggGgaguuu....................................................................	1	1	seq
```

```
                    Mature                                        Star
aucuuuggauagcuucagugaacauucaacgcugucggugaguuugagaacuagaaaaaaccaucgaccguugacuguaccuugagguuuaucacaaccccucuguuaaaugg
....................aacauucaacgcugucggCgaguuu....................................................................	1	1	seq
....................aacauucaacgcuAucggugaguuu....................................................................	1	1	seq
....................Gacauucaacgcugucggugaguuu....................................................................	1	1	seq
....................Cacauucaacgcugucggugaguuu....................................................................	1	1	seq
....................aacauucaacgcugucggugaguuA....................................................................	5	1	seq
....................aacauucaacgcugucggugaguAu....................................................................	3	1	seq
....................aacGuucaacgcugucggugaguuu....................................................................	1	1	seq
....................aacauucaacgcugucggugaguuu....................................................................	576	0	seq
....................aacauucaacgcugucggugaguuG....................................................................	2	1	seq
....................aacauucaacgcugucggugaguuuA...................................................................	1	1	seq
....................aacauucaacgcugucggugaguuGg...................................................................	4	1	seq
....................aacauucaacgcugucggugaguuug...................................................................	1	0	seq
....................aacauucaacgcugucggugaguuuU...................................................................	97	1	seq
....................aacauucaacgcugucggugaguuuAa..................................................................	1	1	seq
....................aacauucaacgcugucggugaguuuga..................................................................	3	0	seq
....................aacauucaacgcugucggugaguuugU..................................................................	5	1	seq
....................aacauucaacgcugucggugaguuugag.................................................................	12	0	seq
....................aacauucaacgcugucggugaguuugagU................................................................	5	1	seq
.....................acauucaacgcugucgguga........................................................................	2	0	seq
.....................acauucaacgcugucgUugag.......................................................................	1	1	seq
.....................acauucaacgcugucggugag.......................................................................	19	0	seq
.....................acauucaacgcugucggugagA......................................................................	5	1	seq
.....................acauucaacgcugucggugagu......................................................................	73	0	seq
.....................acauucaacgcugucggugagG......................................................................	1	1	seq
.....................acauucaacgcugucggugaguu.....................................................................	9	0	seq
.....................acauucaacgcugucggugaguuu....................................................................	4	0	seq
......................cauucaacgcugucgguga........................................................................	1	0	seq
......................cauucaacgcugucggugagu......................................................................	1	0	seq
......................cauucaacgcugucggugaguu.....................................................................	1	0	seq
.......................auucaacgcugucggugagu......................................................................	1	0	seq
........................uucaacgcugucggugaU.......................................................................	1	1	seq
........................uucaacgcugucggugagu......................................................................	2	0	seq
.........................ucaacgcugucggugagu......................................................................	1	0	seq
..........................caacgcugucggugaguu.....................................................................	1	0	seq
...........................................uugagaacuagaaaaaaccaucgaccguugacuguacc................................	1	0	seq
................................................aacuagaaaaaaccaucgaccguugacuguacc................................	1	0	seq
..........................................................aaccaucgaccguugacugu...................................	4	0	seq
..........................................................aaccaucgaccguugacugua..................................	3	0	seq
..........................................................aaccaucgaccguugacuguaA.................................	14	1	seq
..........................................................aaccaucgaccguugacuguac.................................	13	0	seq
..........................................................aaccaucgaccguugacuguaU.................................	7	1	seq
..........................................................aaccaucgaccguugacuguacc................................	13	0	seq
..........................................................aaccaucgaccguugacuguacU................................	3	1	seq
..........................................................aaccaucgaccguugacuguaccu...............................	4	0	seq
...........................................................accaucgaccgCugacug....................................	1	1	seq
...........................................................accaucgaccguugacugA...................................	1	1	seq
...........................................................accaucgaccguugacugu...................................	10	0	seq
...........................................................accaucgaccguugacugua..................................	14	0	seq
...........................................................accaucgaccguugacuguac.................................	58	0	seq
...........................................................accauUgaccguugacuguac.................................	1	1	seq
...........................................................accaucgaccguugacuguaA.................................	46	1	seq
...........................................................accaucgaccguugacuguacA................................	3	1	seq
...........................................................accaucgaccguugacuUuacc................................	1	1	seq
...........................................................accaucgaccguugacuguacU................................	71	1	seq
...........................................................accaucgaccAuugacuguacc................................	1	1	seq
...........................................................accaucgaccguugacuguacc................................	562	0	seq
...........................................................accaucgaccguugaUuguaccu...............................	123	1	seq
...........................................................Uccaucgaccguugacuguaccu...............................	1	1	seq
...........................................................accaucgaccguugacuguaccG...............................	1	1	seq
...........................................................accUucgaccguugacuguaccu...............................	1	1	seq
...........................................................accaucgaccguugacuguaccC...............................	1	1	seq
...........................................................accaucgaccguugacuguaccu...............................	142	0	seq
...........................................................accaucgaccguugacuguaccuu..............................	1	0	seq
...........................................................accaucgaccguugacuguaccAu..............................	1	1	seq
...........................................................accaucgaccguugaUuguaccuu..............................	1	1	seq
............................................................ccaucgaccguugacuguacc................................	2	0	seq
............................................................ccaucgaccguugacuguaccu...............................	6	0	seq
```

Provisional ID	:	mun-novel27
Score total	:	0.1
Score for star read(s)	:	-1.3
Score for read counts	:	0
Score for mfe	:	0.4
Score for randfold	:	1.6
Score for cons. seed	:	-0.6
Total read count	:	103
Mature read count	:	102
Loop read count	:	0
Star read count	:	1

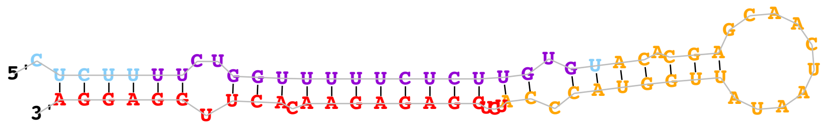

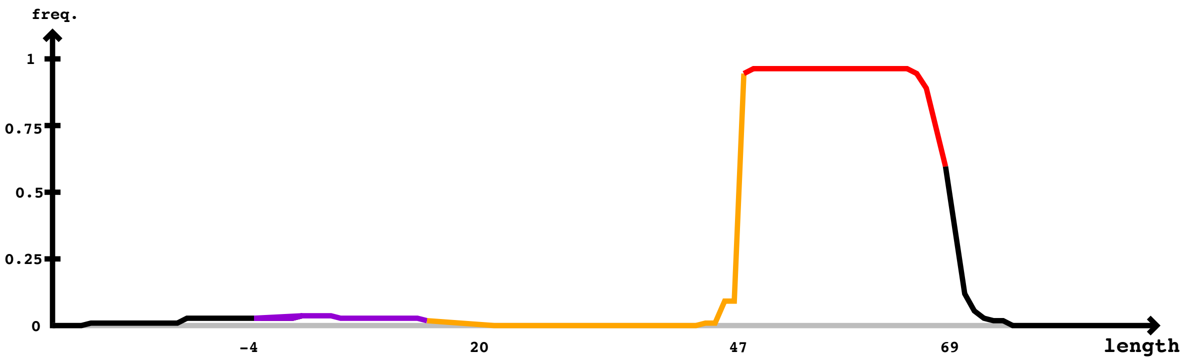


```
                                          Star                                           Mature
5'- ccugccucuuccuucuugccucuuuucugguuuuucucuuguguacacgagcaacuaauauugguacccaucuggagagaacacuuggaggauauaagaccacccaagaagga  -3'   obs
    ccugccucuuccuucuugccucuuuucugguuuuucucuuguguacacgagcaacuaauauugguacccaucuggagagaacacuuggaggauauaagaccacccaagaagga        exp
    .........((((((((((((((.....(((((((((((((.((((.(((..........))))))).))...)))))))).))).))))).............)))))))))   reads   mm   sample
    ..ugccucuuccuucuugccucCuuucu.....................................................................................   1       1    seq
    ............uucuugccucCuuucugguuuuucu............................................................................   1       1    seq
    ............uucuugccucCuuucugguuuuucuc...........................................................................   1       1    seq
    ........................uucugguuuCucucuugug......................................................................   1       1    seq
    ..................................................................Accaucuggagagaacacuuggagga.....................   1       1    seq
    .....................................................................caucuggagagaacacuGggag......................   3       1    seq
    .....................................................................caucuggagagaacacuGggagg.....................   3       1    seq
    .....................................................................caucuggagagaacacuGggagga....................   1       1    seq
    .....................................................................caucuggagagaacacuGggaggaua..................   1       1    seq
    .....................................................................caucuggagagaacacuuggaggauaG.................   1       1    seq
    ......................................................................ucuggagagaacacuGgg.........................   2       1    seq
    ......................................................................ucuggagagaacacuGgga........................   6       1    seq
    ......................................................................ucuggagagaacacuGggag.......................   12      1    seq
    ......................................................................ucuggagagaacacuuggagg......................   1       0    seq
    ......................................................................ucuggagagaacacuGggagg......................   12      1    seq
    ......................................................................ucuggagagaacacuGggagga.....................   31      1    seq
    ......................................................................ucuggagagaacacuuggagga.....................   1       0    seq
    ......................................................................ucuggagagaacacuGggaggau....................   15      1    seq
    ......................................................................ucuggagagaacacuuggaggau....................   3       0    seq
    ......................................................................ucuggagagaacacuGggaggaua...................   4       1    seq
    ......................................................................ucuggagagaacacuuggaggaua...................   1       0    seq
    ......................................................................ucuggagagaacacuuggaggauaG..................   2       1    seq
    ......................................................................ucuggagagaacacuuggaggauaGa.................   1       1    seq
    ......................................................................ucuggagagaacacuuggaggauaGaag...............   2       1    seq
    .......................................................................cuggagagaacacuGggag.......................   1       1    seq
    .......................................................................cuggagagaacacuGggaggaua...................   1       1    seq
```

Provisional ID	:	mun-novel69
Score total	:	4.1
Score for star read(s)	:	-1.3
Score for read counts	:	-1.3
Score for mfe	:	2.2
Score for randfold	:	1.6
Score for cons. seed	:	3
Total read count	:	9
Mature read count	:	9
Loop read count	:	0
Star read count	:	0

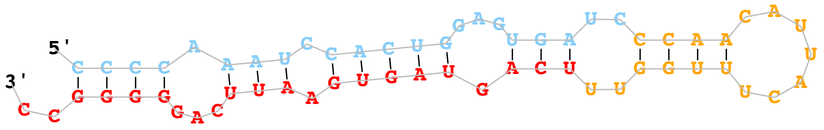

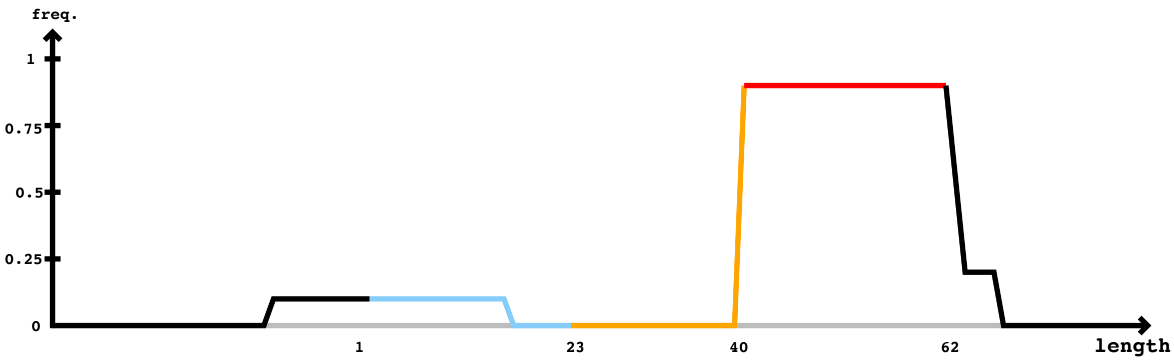

Star Mature

```
5'- ugcccugggggucccccaagaugcucuggggccccaaauccacuggagugaucccaacauuacuuugguuucaguagugaauucagggggccagagaggguggaucccccaa   -3'    exp
    .....((((((((((((......((((..((((((.(((.(((((...(((..((((.......))))..))).))))).)))...)))))))))).))).))).)))))).     reads      mm         sample
    .....................ugcucugggAccccaaauccacugg..................................................................     1          1          seq
    ......................................................................ucaguagugaauucagggggcc....................     4          0          seq
    ......................................................................ucaguagugaauucagggggccG...................     2          1          seq
    ......................................................................ucaguagugaauucagggggccU...................     1          1          seq
    ......................................................................ucaguagugaauucagggggccagaga...............     2          0          seq
```

Provisional ID	:	mun-novel64
Score total	:	0.5
Score for star read(s)	:	-1.3
Score for read counts	:	-1.3
Score for mfe	:	2.2
Score for randfold	:	1.6
Score for cons. seed	:	-0.6
Total read count	:	9
Mature read count	:	9
Loop read count	:	0
Star read count	:	0

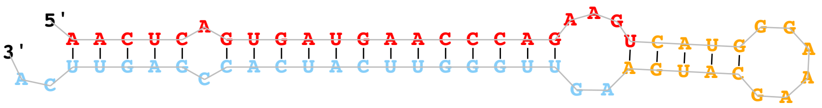

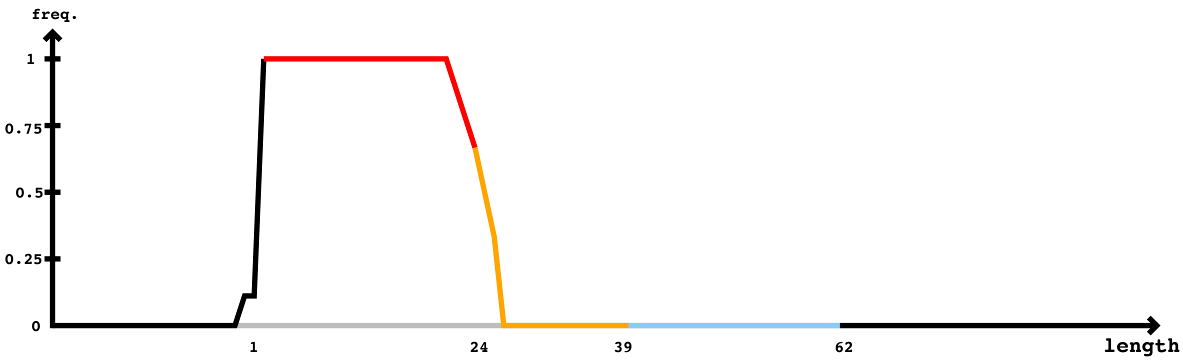

Mature **Star**

```
5'- caugaaagcugaccuuaaugaacucagugaugaacccagaagucaugggaaagcaugaaguuggguucaucaccgaguucaguaaggccagugcuugauccugguugcucugc   -3'    exp
    ((.(...((((.(((((.(((((((.(((((((((((((...(((((......)))))..))))))))))))).))))))).))))).)))).).))................    reads    mm    sample
    ..................ugaacucagugaugaacccaga.........................................................................    1        0     seq
    ....................aacucagugaugaacccagaa........................................................................    1        0     seq
    ....................aacucagugaugaacccagaaA.......................................................................    1        1     seq
    ....................aacucagugaugaacccagaagu......................................................................    3        0     seq
    ....................aacucagugaugaacccagaagucU....................................................................    2        1     seq
    ....................aacucagugaugaacccagaaguca....................................................................    1        0     seq
```

Provisional ID	:	mun-miR-107
Score total	:	1675.1
Score for star read(s)	:	3.9
Score for read counts	:	1665.2
Score for mfe	:	1.4
Score for randfold	:	1.6
Score for cons. seed	:	3
Total read count	:	3278
Mature read count	:	3274
Loop read count	:	0
Star read count	:	4

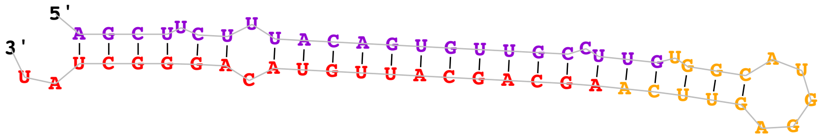

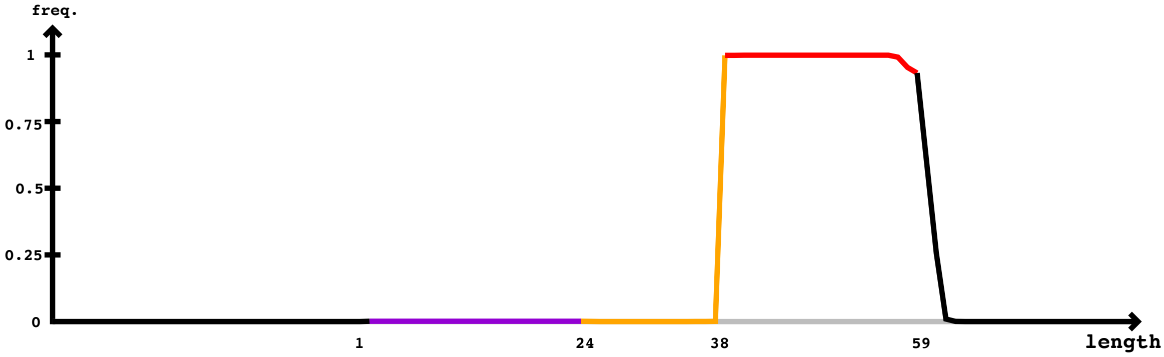


```
                                             Star                                          Mature
5'- augauucucuuucaguguucucuuugcuuucagcuucuuuacaguguugccuuguggcauggaguucaagcagcauuguacagggcuaucaaagcauggagaguugcagca   -3'   obs
    augauucucuuucaguguucucuuugcuuucagcuucuuuacaguguugccuuguggcauggaguucaagcagcauuguacagggcuaucaaagcauggagaguugcagca         exp
    .(((.......)))...((((((.((((((.((((.((.(((((((((((.(((.(((.....))))))))))))))))).))))))...)))))).))))))........   reads   mm   sample
    ...............................agcuucuuuacaguguugccuug.........................................................   4       0    seq
    .................................................................ucaagcagcauuguacagggcu........................   1       0    seq
    ...................................................................aagcagcauuguacagggcuau......................   1       0    seq
    ...................................................................aagcagcauuguacagggcuauc.....................   1       0    seq
    ....................................................................agcagcauuguacagggc.........................   24      0    seq
    ....................................................................agcagcauuguUcagggcu........................   1       1    seq
    ....................................................................agcagcauuguacagggcG........................   1       1    seq
    ....................................................................agcagcauuguacagggcu........................   118     0    seq
    ....................................................................agcagcauuguacagggUu........................   1       1    seq
    ....................................................................agcagcauuguacagggcC........................   1       1    seq
    ....................................................................agcagcauuguacagggcA........................   3       1    seq
    ....................................................................agcagcauuguacagggcua.......................   54      0    seq
    ....................................................................agcagcauuguacagggcuU.......................   12      1    seq
    ....................................................................agcagcauuguacagggcCa.......................   2       1    seq
    ....................................................................agcagcauuguacagggcuG.......................   1       1    seq
    ....................................................................aUcagcauuguacagggcuau......................   1       1    seq
    ....................................................................agcagcauuguacagggcuUu......................   3       1    seq
    ....................................................................agcagcauuguacagggGuau......................   1       1    seq
    ....................................................................agcagcauuguacaUggcuau......................   1       1    seq
    ....................................................................agcagcauuguacagggcuau......................   1146    0    seq
    ....................................................................agcagcauuguacagggcuaC......................   2       1    seq
    ....................................................................agcagcauuguacaggCcuau......................   1       1    seq
    ....................................................................agcagcauuguacagggcuaA......................   5       1    seq
    ....................................................................agcagcauuguaUagggcuauc.....................   2       1    seq
    ....................................................................agcagcGuuguacagggcuauc.....................   1       1    seq
    ....................................................................agcagcauuguacagAgcuauc.....................   1       1    seq
    ....................................................................agcagcauuguacagggcuaCc.....................   1       1    seq
    ....................................................................agcagcauuguacaggUcuauc.....................   1       1    seq
    ....................................................................agcagcauuguacagggcuauA.....................   40      1    seq
    ....................................................................agcagcauuguacagggcuauU.....................   258     1    seq
    ....................................................................agcagcauuguacagggcuauc.....................   745     0    seq
    ....................................................................agcagcauugCacagggcuauc.....................   1       1    seq
    ....................................................................agGagcauuguacagggcuauc.....................   1       1    seq
```

```
                              Star                                 Mature
augauucucuuucaguguucucuuugcuuucagcuucuuuacaguguugccuuguggcauggaguucaagcagcauuguacagggcuaucaaagcauggagaguugcagca
....................................................................agcagcauuguacagggcuauAa....................   14    1    seq
....................................................................agcagcauuguacagggcuauca....................   187   0    seq
....................................................................agcagcauuguacagggcuaucU....................   603   1    seq
....................................................................agcagcauuguacagggcuUuca....................   1     1    seq
....................................................................agcagcauuguacagggcuaucC....................   1     1    seq
....................................................................agcagcauuguacagggcuauUa....................   3     1    seq
....................................................................agcagcaCuguacagggcuauca....................   1     1    seq
....................................................................agcagcauuguacagggcuaucaa...................   13    0    seq
....................................................................agcagcauuguacagggcuaucaU...................   16    1    seq
....................................................................agcagcauuguacagggcuaucaaU..................   2     1    seq
......................................................................cagcauuguacagggcuau......................   2     0    seq
```

Provisional ID	:	mun-novel40
Score total	:	2.5
Score for star read(s)	:	3.9
Score for read counts	:	-3.3
Score for mfe	:	1.1
Score for randfold	:	1.6
Score for cons. seed	:	-0.6
Total read count	:	5
Mature read count	:	4
Loop read count	:	0
Star read count	:	1

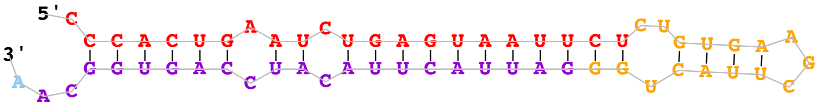

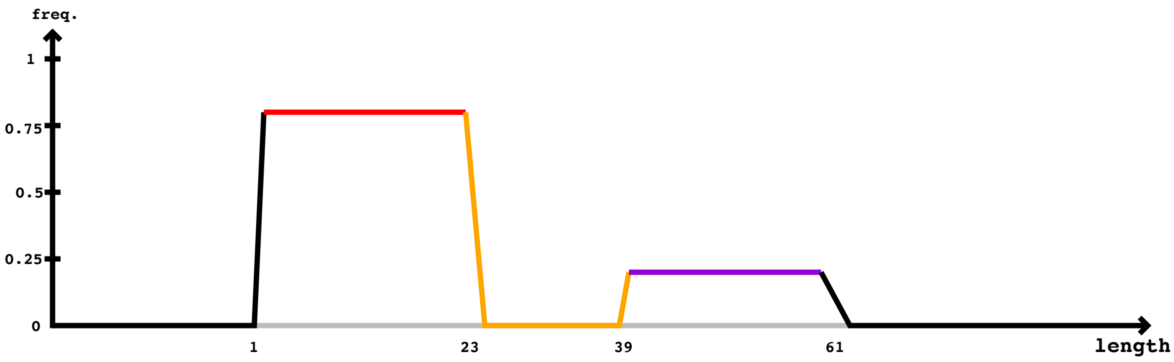

Mature Star

```
5'- cccacugaucacauucuuuacccacugaaucugaguaauucucugugaagcuuacugggauuacuuacauccaguggcaagagaauugauuauuaggcuacaagauuugugc   -3'    obs
    cccacugaucacauucuuuacccacugaaucugaguaauucucugugaagcuuacugggauuacuuacauccaguggcaagagaauugauuauuaggcuacaagauuugugc          exp
    .((..((((((.((((((..((((((.((.((((((((((((..((((...)))).)))))))))))).)).))))))..)))))))))))))...))................    reads      mm        sample
    ....................cccacugaaucugaguaauucu......................................................................    4          0         seq
    ..........................................................gauuacuuacauccaguggca.................................    1          0         seq
```

Provisional ID	:	mun-miR-499
Score total	:	117
Score for star read(s)	:	3.9
Score for read counts	:	106.7
Score for mfe	:	1.9
Score for randfold	:	1.6
Score for cons. seed	:	3
Total read count	:	221
Mature read count	:	204
Loop read count	:	0
Star read count	:	17

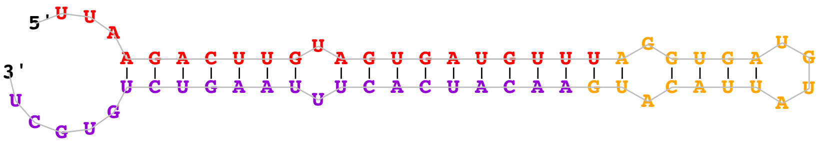

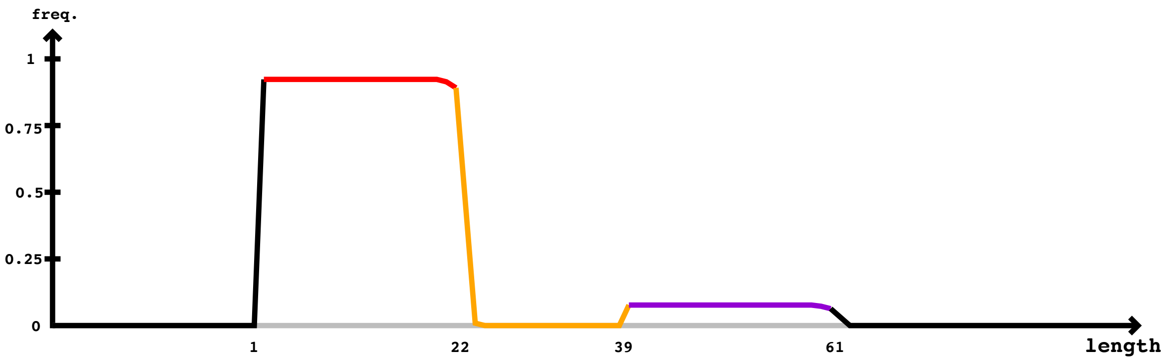

Mature　　Star

```
5'- cgccuuugagagagcggcaguuaagacuuguagugauguuuaggugauguauuacaugaacaucacuuuaagucugugcuacuucucuccucauucuggucagcgggaagg   -3'   obs
    cgccuuugagagagcggcaguuaagacuuguagugauguuuaggugauguauuacaugaacaucacuuuaagucugugcuacuucucuccucauucuggucagcgggaagg         exp
    .......((((((..(((....(((((((.((((((((((((.((((....)))).)))))))))))).))))))).)))..).))))))....((((.(....).))))..    reads   mm   sample
    ....................uuaagacuuguagugaugu........................................................................    2       0    seq
    ....................uuaagacuuguagugauguu.......................................................................    5       0    seq
    ....................uuaagacuuguaguAauguuu......................................................................    1       1    seq
    ....................uuaagacuuguagugauguuu......................................................................    160     0    seq
    ....................uuaagacuuguagugauguuua.....................................................................    32      0    seq
    ....................uuaagacuuguagugauguuuU.....................................................................    2       1    seq
    ....................uuaagacuuguagugauguuuaA....................................................................    2       1    seq
    .........................................................aacaucacuuuaagucugug..................................    1       0    seq
    .........................................................aacaucacuuuaagucugugc.................................    2       0    seq
    .........................................................aacaucacuuuaagucugugcu................................    13      0    seq
    .........................................................aacaucacuuuaagucugugcuG...............................    1       1    seq
```

Provisional ID	:	mun-novel49
Score total	:	37.9
Score for star read(s)	:	3.9
Score for read counts	:	30.7
Score for mfe	:	2.3
Score for randfold	:	1.6
Score for cons. seed	:	-0.6
Total read count	:	72
Mature read count	:	71
Loop read count	:	0
Star read count	:	1

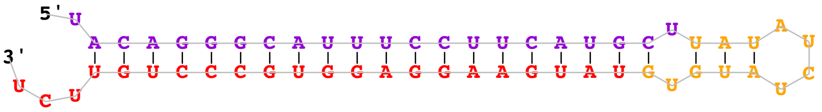

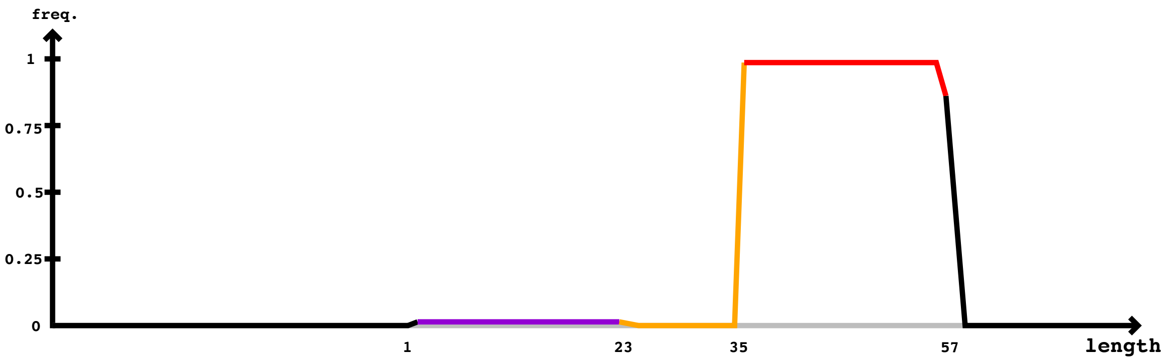

Star Mature

```
5'- aauagcaauuuuacccauuucuaucuaucuacacaguacagggcauuuccuucaugcuuauaucuauguguaugaaggaggugcccuguucugugugga uguccaaggacu   -3'   obs
    aauagcaauuuuacccauuucuaucuaucuacacaguacagggcauuuccuucaugcuuauaucuauguguaugaaggaggugcccuguucuguguggauguccaaggacu         exp
    ............................(((((((((.((((((((((((((((((((.(((....))).)))))))))))))))))))).)))))))))(((....))).   reads   mm   sample
    ....................................uacagggcauuuccuucaugcu.....................................................   1       0    seq
    ......................................................................uaugaaggaggugcccuguuc....................   9       0    seq
    ......................................................................uaugaaggaggCgcccuguucu...................   1       1    seq
    ......................................................................uaugaaggaggugcccuguucu...................   60      0    seq
    ......................................................................uaugaaggaggugcccuguucuU..................   1       1    seq
```

Provisional ID	:	mun-miR-130a
Score total	:	4.8
Score for star read(s)	:	-1.3
Score for read counts	:	0
Score for mfe	:	1.5
Score for randfold	:	1.6
Score for cons. seed	:	3
Total read count	:	503
Mature read count	:	473
Loop read count	:	0
Star read count	:	30

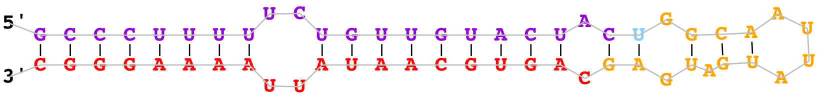

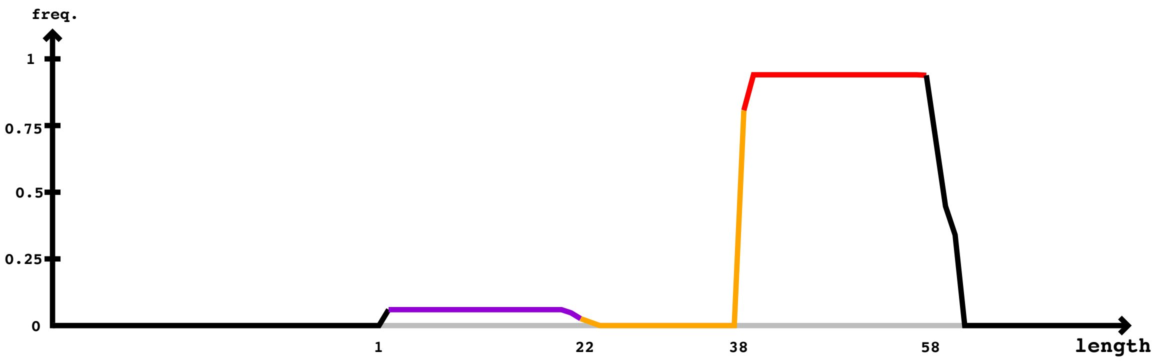

Star **Mature**

```
5'- uuggaaagucucugaaguaugcuguuguccagagcccuuuuucuguuguacuacuggcaauuaugaugagcagugcaauauuaaaagggcauuggcuggcagaaacauga  -3'  obs
    uuggaaagucucugaaguaugcuguuguccagagcccuuuuucuguuguacuacuggcaauuaugaugagcagugcaauauuaaaagggcauuggcuggcagaaacauga       exp
    (..((.....))..).((...((((((.((((.((((((((..(((((((((.((.(((....)).).)).)))))))))..)))))))).)))).))))))..))....   reads  mm  sample
    .................................gcccuuuuucuguuguacu..........................................................   6      0   seq
    .................................gcccuuuuucuguuguacua.........................................................   11     0   seq
    .................................gcccuuuuucuguuguacuac........................................................   12     0   seq
    .................................gcccuuuuucuguuguacuacu.......................................................   1      0   seq
    ......................................................................cagugcaauauuaaaaggg.....................   1      0   seq
    ......................................................................cagugcaauauuaaaagggc....................   131    0   seq
    ......................................................................cagugcaauauuaCaagggc....................   1      1   seq
    ......................................................................caUugcaauauuaaaagggc....................   1      1   seq
    ......................................................................cagugcaauauuaaaagggca...................   105    0   seq
    ......................................................................cagugcaauauuaaaagggcU...................   2      1   seq
    ......................................................................cagugcaauauuaaaagggcaA..................   4      1   seq
    ......................................................................cagugcaauauuaaaagggcau..................   44     0   seq
    ......................................................................cagugcaauauuaaaagggcaC..................   1      1   seq
    ......................................................................cagugcaauauuaaaagggUau..................   2      1   seq
    ......................................................................cagugcaauauuaaaagggcauu.................   83     0   seq
    ......................................................................cagugcaauauuaaaagggcauG.................   4      1   seq
    ......................................................................cagugcaauauuaaaagggcauA.................   24     1   seq
    ......................................................................cagugcCauauuaaaagggcauu.................   1      1   seq
    ......................................................................cGgugcaauauuaaaagggcauu.................   1      1   seq
    ......................................................................cagugcaauauuaaaagggcaAu.................   1      1   seq
    .......................................................................agugcaauauuaaaagggc....................   4      0   seq
    .......................................................................agugcaauauuaaaagggca...................   3      0   seq
    .......................................................................agugcaauauuaaaagggcau..................   3      0   seq
    .......................................................................agugcaauauuaaaagggcauG.................   1      1   seq
    .......................................................................agugcaauauuaaaagggcauu.................   50     0   seq
    .......................................................................agugcaauauuaaaagggcauA.................   5      1   seq
    .......................................................................agugcaauCuuaaaagggcauu.................   1      1   seq
```

Provisional ID	:	mun-miR-106
Score total	:	358.9
Score for star read(s)	:	3.9
Score for read counts	:	348.3
Score for mfe	:	2
Score for randfold	:	1.6
Score for cons. seed	:	3
Total read count	:	695
Mature read count	:	499
Loop read count	:	0
Star read count	:	196

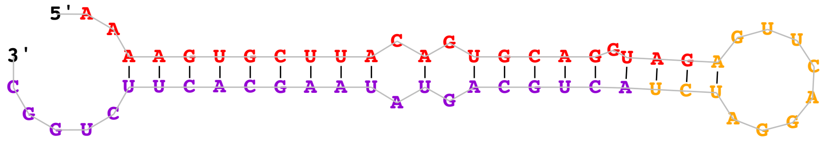

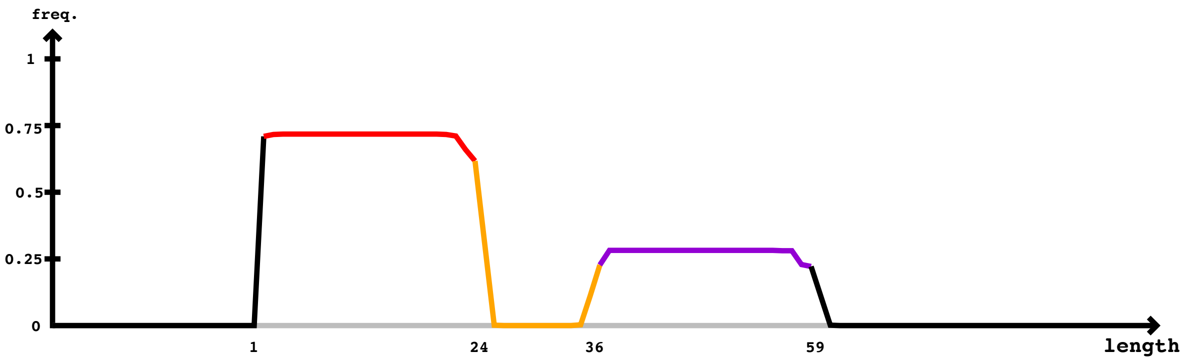


```
                    Mature                              Star
5'- ugaugggccuugaguugugcaaaagugcuuacagugcagguagaguucaggaucuacugcaguauaagcacuucuggcauuacuguggcgaugcggugacgguagcagggcau  -3'    obs
    ugaugggccuugaguugugcaaaagugcuuacagugcagguagaguucaggaucuacugcaguauaagcacuucuggcauuacuguggcgaugcggugacgguagcagggcau          exp
    ......(((((....((((..(((((((((.(.(((((.((((........)))))))).).)))))))))...))))((((((.((......)).)))))).))))).. reads    mm    sample
    ....................aaaagugcuuacagugcag.......................................................................... 1    0    seq
    ....................aaaagugcuuacagugcagg......................................................................... 4    0    seq
    ....................aaaagugcuuacagugcaggC........................................................................ 1    1    seq
    ....................aGaagugcuuacagugcaggu........................................................................ 1    1    seq
    ....................aaaagugcuuacagugcaggu........................................................................ 33   0    seq
    ....................aaaagugcuuacagugcaggua....................................................................... 27   0    seq
    ....................aaaagugcuuacagugcagguU....................................................................... 2    1    seq
    ....................Gaaagugcuuacagugcagguag...................................................................... 1    1    seq
    ....................aaaagugcuuacagugcagguUg...................................................................... 1    1    seq
    ....................aaaagugcuuacagugcagguaA...................................................................... 13   1    seq
    ....................Uaaagugcuuacagugcagguag...................................................................... 1    1    seq
    ....................aaaagugcuuaUagugcagguag...................................................................... 1    1    seq
    ....................aaaagugcuuacagugcagguag...................................................................... 231  0    seq
    ....................aaaagugcuuacagugcagguaU...................................................................... 22   1    seq
    ....................aaaagugcuuacagugcagguaga..................................................................... 8    0    seq
    ....................Caaagugcuuacagugcagguaga..................................................................... 63   1    seq
    ....................aaaagugcuuacagugcagguagU..................................................................... 82   1    seq
    ....................aaaagugcuuacagugcagguagaU.................................................................... 1    1    seq
    .....................aaagugcuuacagugcaggua....................................................................... 1    0    seq
    .....................aaagugcuuacagugcagguag...................................................................... 4    0    seq
    ......................aagugcuuacagugcagguaU...................................................................... 1    1    seq
    .....................................................cuacugcaguauaagcacuuc....................................... 1    0    seq
    .....................................................cuacugcaguauaagcacuucug..................................... 1    0    seq
    ......................................................uacugcaguauaagcacuucug..................................... 29   0    seq
    ......................................................uacugcaguauaagcacuucugA.................................... 1    1    seq
    ......................................................uacugcaguauaagcacuucugg.................................... 3    0    seq
    ......................................................uacugcaguauaagcacuucuggU................................... 1    1    seq
    ......................................................uacugcaguauaagcacuucuggc................................... 36   0    seq
    ......................................................uacugcaguauaagcacuucuggcU.................................. 6    1    seq
    .......................................................acugcaguauaagcacuucug..................................... 6    0    seq
    .......................................................acugcaguauaagcacuucugg.................................... 1    0    seq
    .......................................................acugcaguauaagcacuucuggA................................... 1    1    seq
    .......................................................acugcaguauaagcacuucuggc................................... 51   0    seq
```

Mature **Star**

```
ugaugggccuugaguugugcaaaagugcuuacagugcagguagaguucaggaucuacugcaguauaagcacuucuggcauuacuguggcgaugcggugacgguagcagggcau
.......................................................acugcaguauaagcacuucuggU...................................	1	1	seq
.......................................................acugcaguauaagcacuucuggca..................................	1	0	seq
.......................................................acugcaguauaagcacuucuggcU..................................	19	1	seq
........................................................cugcaguauaagcacuucuggc...................................	21	0	seq
........................................................cugcaguauaagcacuucuggA...................................	1	1	seq
........................................................cugcaguauaagcacuucuggcU..................................	15	1	seq
........................................................cugcaguauaagcacuucuggcau.................................	1	0	seq
```

Provisional ID	:	mun-miR-551-1
Score total	:	5.2
Score for star read(s)	:	-1.3
Score for read counts	:	0
Score for mfe	:	1.9
Score for randfold	:	1.6
Score for cons. seed	:	3
Total read count	:	38
Mature read count	:	36
Loop read count	:	0
Star read count	:	2

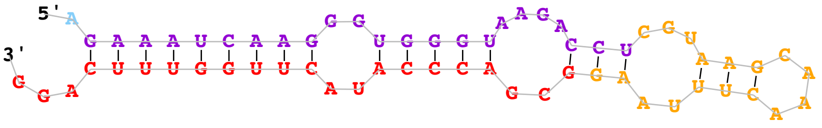

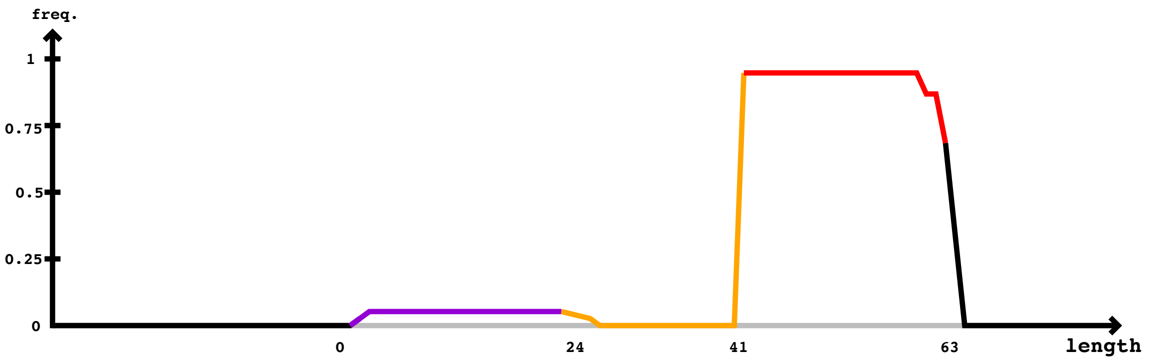


```
                            Star                                        Mature
5'- cacucacccaaagcugcccccauggcuccagaaaucaaggguggguaagaccucguaagcaaacuuuaaggcgacccauacuugguuucaggggcuguguggcuaucuu   -3'   obs
    cacucacccaaagcugcccccauggcuccagaaaucaaggguggguaagaccucguaagcaaacuuuaaggcgacccauacuugguuucaggggcuguguggcuaucuu         exp
    ..............(((..((((((((.((((((((..(((((....(((...(((....)))..)))..)))))..)))))))))).))))))))).)))......   reads   mm   sample
    ..............................gaaaucaaggguggguaagaccu........................................................   1       0    seq
    ...............................aaaucaaggguggguaagaccucg.....................................................   1       0    seq
    ......................................................................gcgacccauacuugguuuc....................   3       0    seq
    ......................................................................gcgacccUuacuugguuucag..................   1       1    seq
    ......................................................................gcgacccauacuugguuucaC..................   1       1    seq
    ......................................................................gcgacccauacuugguuucag..................   5       0    seq
    ......................................................................gcgacccauacuugguuucagU.................   26      1    seq
```

Provisional ID	:	mun-let-7f
Score total	:	1086
Score for star read(s)	:	3.9
Score for read counts	:	1075.3
Score for mfe	:	2.1
Score for randfold	:	1.6
Score for cons. seed	:	3
Total read count	:	2121
Mature read count	:	2025
Loop read count	:	0
Star read count	:	96

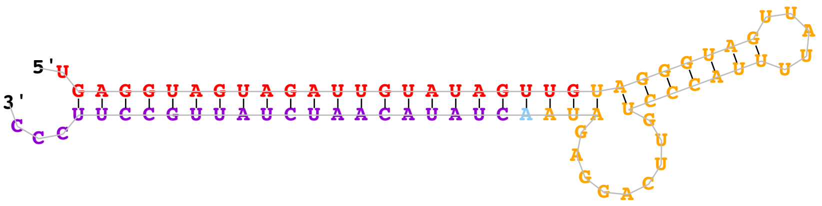

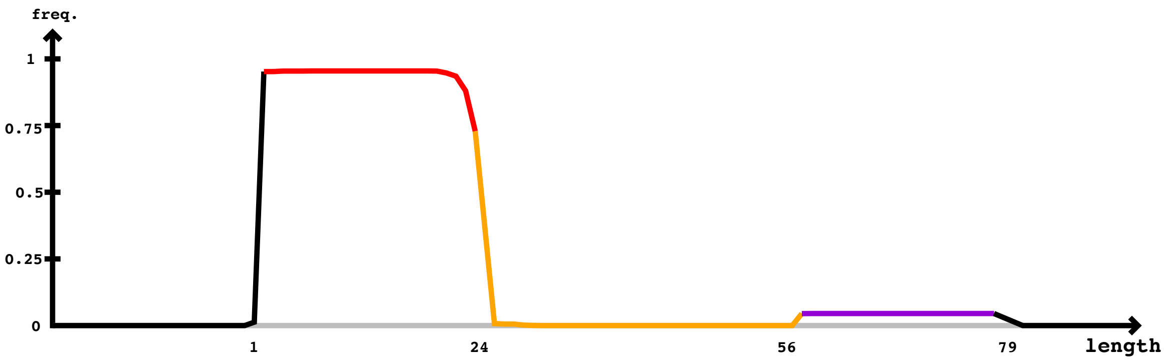


```
                         Mature                                         Star
5'- uguaugcuucucugucagagugagguaguagauuguauaguuguaggguaguuauuuuacccuguucaggagauaacuauacaaucuauugccuucccugaggaguaaaac  -3'   obs
    uguaugcuucucugucagagugagguaguagauuguauaguuguaggguaguuauuuuacccuguucaggagauaacuauacaaucuauugccuucccugaggaguaaaac        exp
    ....(((((((....((.(..((((((((((((((((((((((((((((((.....))))))).........)))))))))))))))))))))))..))))))))))....   reads   mm   sample
    ...................Uugagguaguagauuguauagu......................................................................   6       1    seq
    ...................Cugagguaguagauuguauagu......................................................................   2       1    seq
    ...................Cugagguaguagauuguauaguu.....................................................................   14      1    seq
    ...................Uugagguaguagauuguauaguu.....................................................................   5       1    seq
    ....................ugagguaguagauuguaA.........................................................................   1       1    seq
    ....................ugagguaguagauuguauU........................................................................   14      1    seq
    ....................ugagguagAagauuguaua........................................................................   1       1    seq
    ....................ugagguagAagauuguauag.......................................................................   1       1    seq
    ....................Ggagguaguagauuguauag.......................................................................   1       1    seq
    ....................ugaggCaguagauuguauag.......................................................................   1       1    seq
    ....................Agagguaguagauuguauag.......................................................................   1       1    seq
    ....................ugagguaguagauuguauaU.......................................................................   6       1    seq
    ....................ugaggGaguagauuguauag.......................................................................   1       1    seq
    ....................ugagguaguagauuguauaA.......................................................................   15      1    seq
    ....................ugagguaguagauuguauUgu......................................................................   1       1    seq
    ....................ugagguaguagauuguauGgu......................................................................   1       1    seq
    ....................ugagguaAuagauuguauagu......................................................................   2       1    seq
    ....................ugagguaguagauuguauagA......................................................................   51      1    seq
    ....................Agagguaguagauuguauagu......................................................................   6       1    seq
    ....................ugagguaguagauuguauaAu......................................................................   2       1    seq
    ....................uAagguaguagauuguauagu......................................................................   1       1    seq
    ....................ugaggAaguagauuguauagu......................................................................   4       1    seq
    ....................ugagguaguagauugGauagu......................................................................   1       1    seq
    ....................ugagguGguagauuguauagu......................................................................   2       1    seq
    ....................Ggagguaguagauuguauagu......................................................................   5       1    seq
    ....................ugagguagCagauuguauagu......................................................................   1       1    seq
    ....................ugagguaguagauuguGuagu......................................................................   2       1    seq
    ....................ugagguaguagauuUuauagu......................................................................   4       1    seq
    ....................ugagguaguagaCuguauagu......................................................................   1       1    seq
    ....................ugaAguaguagauuguauagu......................................................................   1       1    seq
    ....................ugagguagAagauuguauagu......................................................................   2       1    seq
    ....................uUagguaguagauuguauagu......................................................................   1       1    seq
    ....................Cgagguaguagauuguauagu......................................................................   1       1    seq
```

Mature   Star

```
uguaugcuucucugucagagugagguaguagauuguauaguuguaggguaguuauuuuacccuguucaggagauaacuauacaaucuauugccuucccugaggaguaaaac
....................ugagguaguagauuCuauagu......................................................................   1     1   seq
....................ugagguaguagauuguauaUu......................................................................   4     1   seq
....................ugagguaguagauuguUuagu......................................................................   1     1   seq
....................ugaggGaguagauuguauagu......................................................................   6     1   seq
....................ugagguaguagauAguauagu......................................................................   1     1   seq
....................ugagguaguagauuguauagC......................................................................   1     1   seq
....................ugagguaguaUauuguauagu......................................................................   2     1   seq
....................ugaggCaguagauuguauagu......................................................................   2     1   seq
....................ugagguaguagauuAuauagu......................................................................   1     1   seq
....................ugagguaguagauuguauagGu.....................................................................   5     1   seq
....................uCagguaguagauuguauaguu.....................................................................   1     1   seq
....................ugagguaguagaAuguauaguu.....................................................................   1     1   seq
....................ugagguaguaUauuguauaguu.....................................................................   3     1   seq
....................ugagguaguagauCguauaguu.....................................................................   2     1   seq
....................ugagguaguagauuguauGguu.....................................................................   2     1   seq
....................ugagguaguagauuguaCaguu.....................................................................   3     1   seq
....................ugagguaguagauugCauaguu.....................................................................   1     1   seq
....................ugagguaguagauuguauaAuu.....................................................................   3     1   seq
....................ugagguaguagauuguauaguA.....................................................................   57    1   seq
....................uAagguaguagauuguauaguu.....................................................................   2     1   seq
....................ugagguaguagauuguauaUuu.....................................................................   5     1   seq
....................ugaggAaguagauuguauaguu.....................................................................   21    1   seq
....................ugaggCaguagauuguauaguu.....................................................................   12    1   seq
....................ugagguaguagauuguauaguG.....................................................................   4     1   seq
....................ugagguaguagauuguauaguC.....................................................................   9     1   seq
....................ugagguaguagauuCuauaguu.....................................................................   3     1   seq
....................ugagguaguagauuAuauaguu.....................................................................   1     1   seq
....................ugagUuaguagauuguauaguu.....................................................................   4     1   seq
....................ugagguagGagauuguauaguu.....................................................................   4     1   seq
....................Ggagguaguagauuguauaguu.....................................................................   25    1   seq
....................ugGgguaguagauuguauaguu.....................................................................   4     1   seq
....................ugagguaguagauuguauagCu.....................................................................   4     1   seq
....................ugagguaguagauuUuauaguu.....................................................................   4     1   seq
....................ugagguaguagauAguauaguu.....................................................................   1     1   seq
....................uUagguaguagauuguauaguu.....................................................................   2     1   seq
....................Agagguaguagauuguauaguu.....................................................................   19    1   seq
....................ugaAguaguagauuguauaguu.....................................................................   1     1   seq
....................Cgagguaguagauuguauaguu.....................................................................   16    1   seq
....................ugagguaguagCuuguauaguu.....................................................................   6     1   seq
....................ugagguaguGgauuguauaguu.....................................................................   2     1   seq
....................ugagguagCagauuguauaguu.....................................................................   3     1   seq
....................ugagguaguagauuguauUguu.....................................................................   1     1   seq
....................ugCgguaguagauuguauaguu.....................................................................   1     1   seq
....................ugagguaUuagauuguauaguu.....................................................................   2     1   seq
....................ugUgguaguagauuguauaguu.....................................................................   2     1   seq
....................ugagguGguagauuguauaguu.....................................................................   3     1   seq
....................ugagCuaguagauuguauaguu.....................................................................   1     1   seq
....................ugagAuaguagauuguauaguu.....................................................................   1     1   seq
....................ugagguCguagauuguauaguu.....................................................................   1     1   seq
....................ugaUguaguagauuguauaguu.....................................................................   4     1   seq
....................ugaCguaguagauuguauaguu.....................................................................   1     1   seq
....................ugaggGaguagauuguauaguu.....................................................................   35    1   seq
....................ugagguagAagauuguauaguu.....................................................................   4     1   seq
....................ugagguaguagauugGauaguu.....................................................................   11    1   seq
....................ugagguaguagauuguaAaguu.....................................................................   2     1   seq
....................ugagguaguagauuguGuaguu.....................................................................   4     1   seq
....................ugagguaguagauuguauaguAg....................................................................   1     1   seq
....................ugaggGaguagauuguauaguug....................................................................   1     1   seq
....................ugagguaguagGuuguauaguug....................................................................   79    1   seq
....................ugagguaguagauuguauaguuA....................................................................   1240  1   seq
....................ugagguaguagauuguauaguug....................................................................   82    0   seq
....................uUagguaguagauuguauaguug....................................................................   1     1   seq
....................ugagguaguagauuguauaguuC....................................................................   46    1   seq
....................ugagguaguagauuguauaguugA...................................................................   1     1   seq
....................ugagguaguagauuguauaguugu...................................................................   7     0   seq
....................ugagguaguagauuguauaguuCu...................................................................   1     1   seq
....................ugagguaguagauuguauaguugG...................................................................   1     1   seq
....................ugagguaguagGuuguauaguugu...................................................................   20    1   seq
....................ugagguaguagauuguauaguuAu...................................................................   45    1   seq
....................ugagguaguagauuguauaguugua..................................................................   4     0   seq
```

```
                    Mature                                                     Star
uguaugcuucucugucagagugagguaguagauuguauaguuguaggguaguuauuuuacccuguucaggagauaacuauacaaucuauugccuucccugaggaguaaaac
....................ugagguaguagauuguauaguuguagg................................................................	6	0	seq
....................ugagguaguagauuguauaguugGagg................................................................	1	1	seq
....................ugagguaguagauuguauaguuguagU................................................................	1	1	seq
....................ugagguaguagauuguauaguuguaggU...............................................................	1	1	seq
....................ugagguaguagauuguauaguuguaggg...............................................................	1	0	seq
....................ugagguaguagauuguauaguugGaggg...............................................................	1	1	seq
....................ugagguaguagauuguauaguuguagggA..............................................................	1	1	seq
......................agguaguagauuguauaguugu...................................................................	4	0	seq
.........................uaguagauuguauaguuA....................................................................	1	1	seq
............................................................................cuauacaaucuauugccuucU..............	1	1	seq
............................................................................cuauacaaucuauugccuuccA.............	1	1	seq
............................................................................cuauacaaucuauugccuuccU.............	87	1	seq
............................................................................cuauacaaucuauugccuuccG.............	1	1	seq
............................................................................cuauacaaucuauugccuuccUu............	5	1	seq
.............................................................................uauacaaucuauugccuuccU.............	1	1	seq
```

Provisional ID	:	mun-miR-219b
Score total	:	5.3
Score for star read(s)	:	-1.3
Score for read counts	:	0
Score for mfe	:	2
Score for randfold	:	1.6
Score for cons. seed	:	3
Total read count	:	53
Mature read count	:	53
Loop read count	:	0
Star read count	:	0

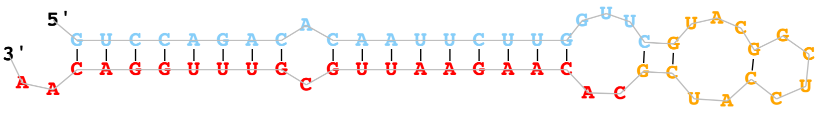

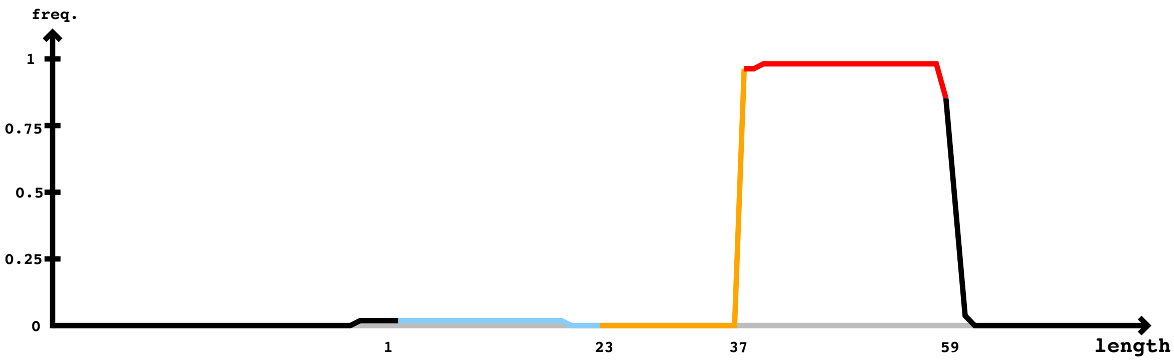

Star Mature

```
5'- cccggccgguggagucggaaccucugcuacagauguccagacacaauucuugguucguacggcuccaucgcacaagaauugcguuuggacaaucaggagcggagauucccau  -3'   exp
    .((((((....).)))))...((((((...((((((((((.(((((((((...((...(....)..))..))))))))).))))))))).)))...)))))))........   reads   mm   sample
    ..............................agauguccagacacaauucuug............................................................   1       0    seq
    ......................................................................cacaagaauugcguuuggaca.....................   3       0    seq
    ......................................................................cacaagaauugcguuuggaAa.....................   4       1    seq
    ......................................................................cacaagaauugcguuuggacaa....................   25      0    seq
    ......................................................................cacaagaauugcguuuggacaaA...................   1       1    seq
    ......................................................................cacaagaauugcguuuggacaau...................   17      0    seq
    ......................................................................cacaagaauugcguuuggacaauU..................   2       1    seq
    ........................................................................caagaauugcguuuggacaau...................   1       0    seq
```

Provisional ID	:	mun-let-7a
Score total	:	1299.8
Score for star read(s)	:	3.9
Score for read counts	:	1289.5
Score for mfe	:	1.9
Score for randfold	:	1.6
Score for cons. seed	:	3
Total read count	:	2541
Mature read count	:	2532
Loop read count	:	1
Star read count	:	8

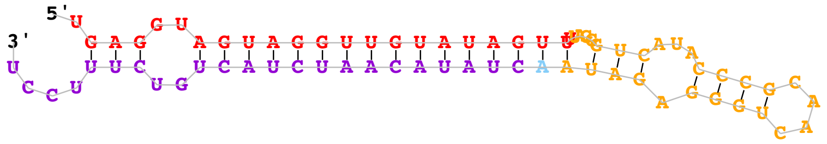

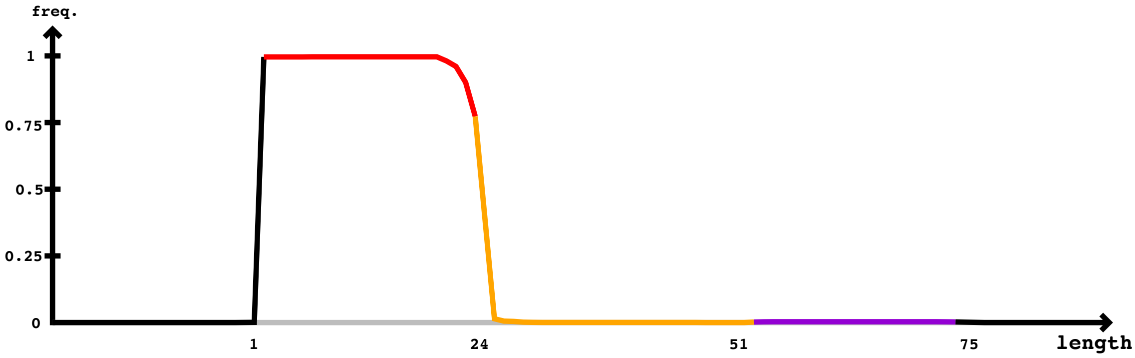


```
                      Mature                                    Star
5'- aagaugccugcacugugggaugagguaguagguuguauaguuuuagggucauacccgcaacugggagauaacuauacaaucuacugucuuuccuaaagcagcagaaaa  -3'  obs
    aagaugccugcacugugggaugagguaguagguuguauaguuuuagggucauacccgcaacugggagauaacuauacaaucuacugucuuuccuaaagcagcagaaaa       exp
    ....(((.(((....(((((.(((..(((((((((((((((.....(((...((((....)))).))))))))))))))))))..))))))))..))))))..... reads  mm  sample
    ..................Aaugagguaguagguuguauaguu..................................................................  1      1   seq
    ...................augagguaguagguuguauGguu..................................................................  1      1   seq
    ....................ugagguaguagguuguauU.....................................................................  37     1   seq
    ....................ugagguaguagguuguaAa.....................................................................  1      1   seq
    ....................ugaggGaguagguuguaua.....................................................................  1      1   seq
    ....................ugaggAaguagguuguauag....................................................................  2      1   seq
    ....................ugagguaguagguuguauUg....................................................................  1      1   seq
    ....................ugagguGguagguuguauag....................................................................  1      1   seq
    ....................ugagguagGagguuguauag....................................................................  1      1   seq
    ....................ugagguaguagguAguauag....................................................................  1      1   seq
    ....................ugaggGaguagguuguauag....................................................................  3      1   seq
    ....................ugagguaguagguuguauaA....................................................................  27     1   seq
    ....................ugagguaguagguuguauaU....................................................................  17     1   seq
    ....................ugagguaguagguuguauaUu...................................................................  8      1   seq
    ....................ugagguaCuagguuguauagu...................................................................  1      1   seq
    ....................ugaggCaguagguuguauagu...................................................................  8      1   seq
    ....................Agagguaguagguuguauagu...................................................................  1      1   seq
    ....................ugagguaguaUguuguauagu...................................................................  3      1   seq
    ....................ugagguaguagguuguGuagu...................................................................  2      1   seq
    ....................ugagguaguaggGuguauagu...................................................................  2      1   seq
    ....................ugagguUguagguuguauagu...................................................................  1      1   seq
    ....................ugagguaguagguuguaAagu...................................................................  1      1   seq
    ....................ugagguaguUgguuguauagu...................................................................  1      1   seq
    ....................ugagguaguagguuguaCagu...................................................................  1      1   seq
    ....................ugagguaguagguuguauagC...................................................................  2      1   seq
    ....................Ggagguaguagguuguauagu...................................................................  6      1   seq
    ....................ugagguaguagguuguauagA...................................................................  62     1   seq
    ....................ugagUuaguagguuguauagu...................................................................  1      1   seq
    ....................ugagguaguaAguuguauagu...................................................................  1      1   seq
    ....................Cgagguaguagguuguauagu...................................................................  2      1   seq
    ....................ugagguaguagguuguauaAu...................................................................  4      1   seq
    ....................ugagguagGagguuguauagu...................................................................  1      1   seq
    ....................ugagguaguagguAguauagu...................................................................  1      1   seq
```

```
                     Mature                                     Star
aagaugccugcacugugggaugagguaguagguuguauaguuuuagggucauacccgcaacugggagauaacuauacaaucuacugucuuuccuaaagcagcagaaaa
....................ugagguagCagguuguauagu.................................................................... 2 1 seq
....................ugaggGaguagguuguauagu.................................................................... 14 1 seq
....................ugagguaguaggCuguauagu.................................................................... 1 1 seq
....................ugagguaguagguuguauagG.................................................................... 2 1 seq
....................ugagguaguagguuUuauagu.................................................................... 1 1 seq
....................ugagguagAagguuguauagu.................................................................... 1 1 seq
....................ugaUguaguagguuguauagu.................................................................... 1 1 seq
....................ugaggAaguagguuguauagu.................................................................... 9 1 seq
....................ugGgguaguagguuguauagu.................................................................... 2 1 seq
....................uUagguaguagguuguauagu.................................................................... 3 1 seq
....................ugUgguaguagguuguauagu.................................................................... 1 1 seq
....................uCagguaguagguuguauagu.................................................................... 1 1 seq
....................ugagguaguagguugAauagu.................................................................... 2 1 seq
....................ugagguaguaggAuguauagu.................................................................... 1 1 seq
....................ugagguaguagguuCuauagu.................................................................... 2 1 seq
....................ugGgguaguagguuguauaguu................................................................... 5 1 seq
....................ugagguaguGgguuguauaguu................................................................... 6 1 seq
....................ugagguGguagguuguauaguu................................................................... 10 1 seq
....................ugagguaguagguuUuauaguu................................................................... 5 1 seq
....................ugaCguaguagguuguauaguu................................................................... 1 1 seq
....................ugCgguaguagguuguauaguu................................................................... 3 1 seq
....................ugagguaguagguuguaAaguu................................................................... 2 1 seq
....................Agagguaguagguuguauaguu................................................................... 18 1 seq
....................ugagguagGagguuguauaguu................................................................... 9 1 seq
....................ugagguaguagguuguauagAu................................................................... 3 1 seq
....................ugagguaguaggCuguauaguu................................................................... 11 1 seq
....................ugagguaguagguuguauaguG................................................................... 5 1 seq
....................ugaUguaguagguuguauaguu................................................................... 5 1 seq
....................ugagguaguagguuguauagGu................................................................... 1 1 seq
....................ugagguaguagguuguaCaguu................................................................... 11 1 seq
....................ugagguaguaggAuguauaguu................................................................... 4 1 seq
....................uUagguaguagguuguauaguu................................................................... 5 1 seq
....................ugagguaguagguuguauagCu................................................................... 4 1 seq
....................ugagguaguaUguuguauaguu................................................................... 2 1 seq
....................ugagguaguagguuguauCguu................................................................... 1 1 seq
....................ugagguaguagguCguauaguu................................................................... 3 1 seq
....................ugaggAaguagguuguauaguu................................................................... 32 1 seq
....................Ggagguaguagguuguauaguu................................................................... 17 1 seq
....................ugagguaguagguuguGuaguu................................................................... 2 1 seq
....................ugagguagCagguuguauaguu................................................................... 6 1 seq
....................ugagCuaguagguuguauaguu................................................................... 1 1 seq
....................ugagguaguagguuguauaUuu................................................................... 7 1 seq
....................ugagguaCuagguuguauaguu................................................................... 1 1 seq
....................ugagUuaguagguuguauaguu................................................................... 4 1 seq
....................ugagguUguagguuguauaguu................................................................... 1 1 seq
....................ugagguaguagguuguaGaguu................................................................... 1 1 seq
....................uAagguaguagguuguauaguu................................................................... 2 1 seq
....................ugaggGaguagguuguauaguu................................................................... 43 1 seq
....................ugagguaguagguuguauaguC................................................................... 3 1 seq
....................ugagguaguCgguuguauaguu................................................................... 1 1 seq
....................ugagguaguagguuguauaAuu................................................................... 1 1 seq
....................ugagguaguUgguuguauaguu................................................................... 2 1 seq
....................ugagguaguagguugAauaguu................................................................... 1 1 seq
....................ugagguaguagguuguauaCuu................................................................... 2 1 seq
....................ugagguaguagguugGauaguu................................................................... 3 1 seq
....................ugagguaguagguuguUuaguu................................................................... 2 1 seq
....................ugUgguaguagguuguauaguu................................................................... 2 1 seq
....................ugaggCaguagguuguauaguu................................................................... 23 1 seq
....................ugagguagAagguuguauaguu................................................................... 3 1 seq
....................ugagguaguagguugCauaguu................................................................... 5 1 seq
....................ugagguaUuagguuguauaguu................................................................... 3 1 seq
....................ugagguaguaCguuguauaguu................................................................... 3 1 seq
....................Cgagguaguagguuguauaguu................................................................... 13 1 seq
....................ugagguaguagguuCuauaguu................................................................... 3 1 seq
....................ugagguaguagCuuguauaguu................................................................... 6 1 seq
....................ugagguaguagguuAuauaguu................................................................... 2 1 seq
....................ugagguaguaggGuguauaguu................................................................... 14 1 seq
....................ugaggGaguagguuguauaguuu.................................................................. 14 1 seq
....................ugagguaguaggGuguauaguuu.................................................................. 4 1 seq
....................ugagguagAagguuguauaguuu.................................................................. 2 1 seq
```

Mature Star

```
aagaugccugcacugugggaugagguaguagguuguauaguuuuagggucauacccgcaacugggagauaacuauacaaucuacugucuuuccuaaagcagcagaaaa
....................ugagguaguagguuguauaguCu.................................................................	5	1	seq
....................ugagguaguagguuguauagAuu.................................................................	2	1	seq
....................Agagguaguagguuguauaguuu.................................................................	3	1	seq
....................ugagguaguGgguuguauaguuu.................................................................	2	1	seq
....................ugagguaguagguuguauaguuC.................................................................	50	1	seq
....................ugagguGguagguuguauaguuu.................................................................	4	1	seq
....................uAagguaguagguuguauaguuu.................................................................	1	1	seq
....................ugagguaguagguuguauaguAu.................................................................	28	1	seq
....................Cgagguaguagguuguauaguuu.................................................................	1	1	seq
....................ugagguaguaUguuguauaguuu.................................................................	2	1	seq
....................ugagUuaguagguuguauaguuu.................................................................	1	1	seq
....................ugagguaguagguCguauaguuu.................................................................	1	1	seq
....................ugagguaguagguuguauaguGu.................................................................	3	1	seq
....................ugagguaguagguuUuauaguuu.................................................................	2	1	seq
....................ugagguaguagguuguauCguuu.................................................................	2	1	seq
....................ugagguaguagguuCuauaguuu.................................................................	3	1	seq
....................ugagguagGagguuguauaguuu.................................................................	1	1	seq
....................ugagguaguagguuguauaguuG.................................................................	79	1	seq
....................ugagguaguagguGguauaguuu.................................................................	1	1	seq
....................ugagguaguagguuguauaUuuu.................................................................	1	1	seq
....................ugaggCaguagguuguauaguuu.................................................................	6	1	seq
....................ugagguaguagguuguauaguuA.................................................................	1139	1	seq
....................ugaUguaguagguuguauaguuu.................................................................	5	1	seq
....................ugagguaguagguuguauUguuu.................................................................	1	1	seq
....................ugagguaguagguuguauagCuu.................................................................	3	1	seq
....................ugaggAaguagguuguauaguuu.................................................................	10	1	seq
....................ugagguagCagguuguauaguuu.................................................................	2	1	seq
....................ugagguaguagguugCauaguuu.................................................................	1	1	seq
....................ugaAguaguagguuguauaguuu.................................................................	1	1	seq
....................ugGgguaguagguuguauaguuu.................................................................	1	1	seq
....................Ggagguaguagguuguauaguuu.................................................................	3	1	seq
....................ugagguaguagguuguauaguAuu................................................................	1	1	seq
....................ugagguaguagguuguauGguuuu................................................................	64	1	seq
....................Cgagguaguagguuguauaguuuu................................................................	1	1	seq
....................ugagguaguagguuguauaguuuC................................................................	4	1	seq
....................ugagguaguagguuguauaguuuu................................................................	352	0	seq
....................ugagguagGagguuguauaguuuu................................................................	1	1	seq
....................ugagguaguagUuuguauaguuuu................................................................	1	1	seq
....................ugagguaguagguuguauaguuGu................................................................	20	1	seq
....................ugagguaguagguuguauaguuCu................................................................	7	1	seq
....................ugagguaguagguuguaCaguuuu................................................................	1	1	seq
....................ugagguaguagguuguauaguuAu................................................................	93	1	seq
....................ugagguaguagguuguauaguuuua...............................................................	2	0	seq
....................ugagguaguagguuguauaguuuuU...............................................................	20	1	seq
....................ugagguaguagguuguauaguuuuGg..............................................................	1	1	seq
....................ugagguaguagguuguauaguuuuag..............................................................	2	0	seq
....................ugagguaguagguuguauaguuuuagA.............................................................	1	1	seq
....................ugagguaguagguuguauaguuuuagg.............................................................	6	0	seq
....................ugagguaguagguuguauaguuuuUggg............................................................	1	1	seq
....................ugagguaguagguuguauaguuuuaggU............................................................	1	1	seq
.........................uaguagguuguauaguuuuaggUu...........................................................	1	1	seq
..........................................uuagggucauacccgcaacuggga..........................................	1	0	seq
.......................................................................cuauacaGucuacugucuuu.................	1	1	seq
.......................................................................cCauacaaucuacugucuuuc................	1	1	seq
.......................................................................cuauacaaucuacugucuuuccA..............	3	1	seq
........................................................................uauacaaucuacugucuuuccC..............	1	1	seq
........................................................................uauacaaucuacugucuuuccuU.............	1	1	seq
.........................................................................auacaaucuacugucuuuccuU.............	1	1	seq
```

Provisional ID	:	mun-novel86
Score total	:	0
Score for star read(s)	:	-1.3
Score for read counts	:	0
Score for mfe	:	0.2
Score for randfold	:	1.6
Score for cons. seed	:	-0.6
Total read count	:	17
Mature read count	:	17
Loop read count	:	0
Star read count	:	0

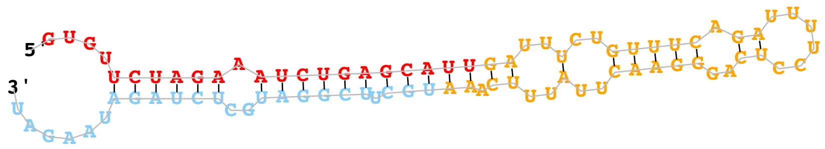

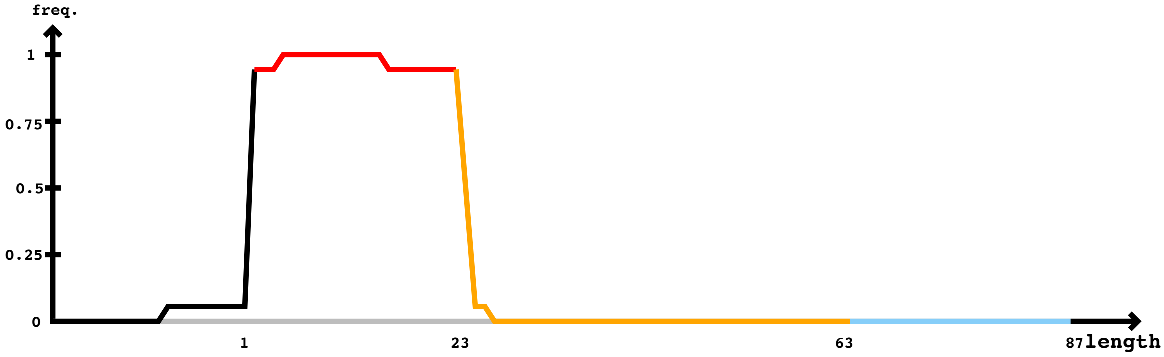

Mature **Star**

```
5'- augcuagaaaucuuugugugguguucuagaaaucugagcauugauuucuguuucagauuuuccucagggaacuuauuucaaaugcuucggaugcucuagauaagauacgguu   -3'    exp
    ............((((((.(..((((((.((((((((((((((..(..(((((.((......))..)))))..)..)).))))).))))))..))))))..).))))))..   reads    mm    sample
    ..........ucuuuguguguguucuagaaauc..............................................................................   1        0     seq
    ...................guguucuagaaaucugagcauu......................................................................   13       0     seq
    ...................guguucuagaaaucugagcauA......................................................................   3        1     seq
    ......................uucuagaaaucugagcauugUu...................................................................   1        1     seq
```

Provisional ID	:	mun-miR-203
Score total	:	662.3
Score for star read(s)	:	3.9
Score for read counts	:	655.3
Score for mfe	:	2.1
Score for randfold	:	1.6
Score for cons. seed	:	-0.6
Total read count	:	1297
Mature read count	:	1278
Loop read count	:	0
Star read count	:	19

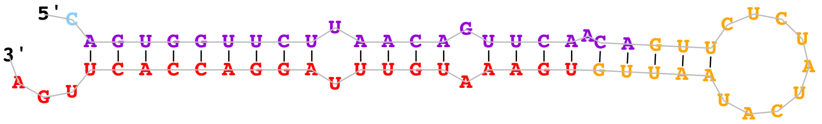

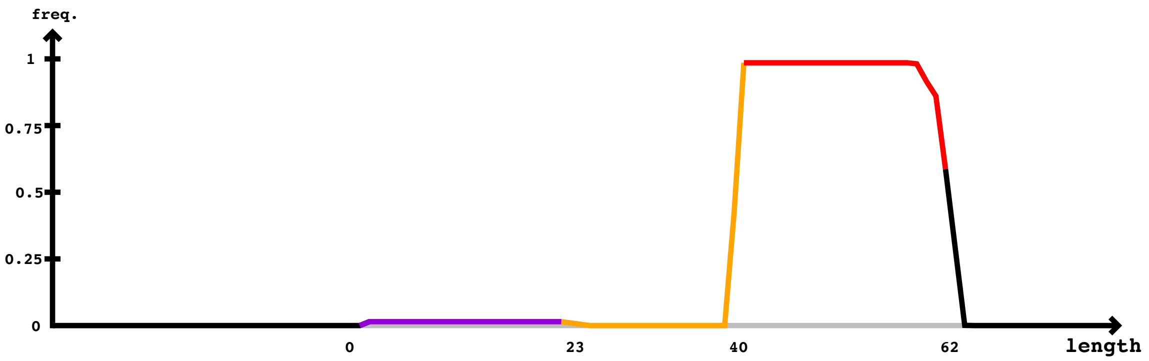

Star **Mature**

```
5'- acggaccgcggcuccucagccuccuuggugcagugguucuuaacaguucaacaguucucuaucauaaauugugaaauguuuaggaccacuugaccagggaggccccggca  -3'   obs
    acggaccgcggcuccucagccuccuuggugcagugguucuuaacaguucaacaguucucuaucauaaauugugaaauguuuaggaccacuugaccagggaggccccggca         exp
    ...(.(((.((.......(((((((((((..(((((((((.((((.((((.(((((..........))))))))).)))).)))))))))..))))))))))))))))).    reads   mm   sample
    ...............................agugguucuuaacaguucaac..........................................................    8       0    seq
    ...............................agugguucuuaacaguucaaca.........................................................    8       0    seq
    ...............................agugguucuuaacaguucaacU.........................................................    1       1    seq
    ...............................agugguucuuaacaguucaacag........................................................    2       0    seq
    ......................................................................gugaaauguuuaggaccac.....................    3       0    seq
    ......................................................................gugaaauguuuaggaccacA....................    1       1    seq
    ......................................................................gugaaauguuuaCgaccacu....................    1       1    seq
    ......................................................................gugaaauguuuaggaccacu....................    64      0    seq
    ......................................................................gugaaauguuuaggaccacuu...................    51      0    seq
    ......................................................................Uugaaauguuuaggaccacuug..................    1       1    seq
    ......................................................................gugaaauguuuaggaccacuuU..................    20      1    seq
    ......................................................................gugaaauguuuaggaccacuug..................    162     0    seq
    ......................................................................gugaaaugGuuaggaccacuug..................    1       1    seq
    ......................................................................gugaaauguuuaggaccacuuUa.................    1       1    seq
    ......................................................................gugaaauguuuaggaccacuuga.................    47      0    seq
    ......................................................................gugaaauguuuaggaccacuuAa.................    1       1    seq
    ......................................................................gugaaauguuuaggaccacuugG.................    1       1    seq
    ......................................................................gugaaauguuuaggaccacuugU.................    184     1    seq
    ......................................................................gugaaauguuuaggaccacuugac................    1       0    seq
    ......................................................................gugaaauguuuaggaccacuugaU................    4       1    seq
    ......................................................................gugaaauguuuaggaccacuugaA................    16      1    seq
    .......................................................................ugaaauguuuaggaccac.....................    2       0    seq
    .......................................................................ugaaauguuuaggaccacu....................    18      0    seq
    .......................................................................ugaaauguuuaggaccacuu...................    22      0    seq
    .......................................................................ugaaauguuuaggaccacuug..................    148     0    seq
    .......................................................................ugaaauguuuaggaccacuuU..................    17      1    seq
    .......................................................................Agaaauguuuaggaccacuug..................    1       1    seq
    .......................................................................ugGaauguuuaggaccacuug..................    1       1    seq
    .......................................................................ugaaauguuuaggaccacuuA..................    3       1    seq
    .......................................................................ugaaauguuuaggaccacuuC..................    1       1    seq
    .......................................................................ugaaauguuuaggaccacuugU.................    399     1    seq
    .......................................................................ugaaauguuuaggaccacuugG.................    1       1    seq
    .......................................................................ugaaauguuuaggaccacuuUa.................    2       1    seq
```

```
                              Star                     Mature
acggaccgcggcuccucagccuccuuggugcagugguucuuaacaguucaacaguucucuaucauaauugugaaauguuuaggaccacuugaccagggaggccccggca
......................................................................ugaaauguuuaggaccacuuga.................   72   0   seq
......................................................................ugaaauguuuaggaccacuuAa.................   1    1   seq
......................................................................ugaaauguuuaggaccacuugC.................   4    1   seq
......................................................................ugaaauguuuaggaccacuugaA................   13   1   seq
......................................................................ugaaauguuuaggaccacuugaU................   12   1   seq
......................................................................ugaaauguuuaggaccacuugac................   1    0   seq
......................................................................ugaaauguuuaggaccacuugacA...............   1    1   seq
```

Provisional ID	:	mun-novel33
Score total	:	14.2
Score for star read(s)	:	3.9
Score for read counts	:	7.3
Score for mfe	:	2
Score for randfold	:	1.6
Score for cons. seed	:	-0.6
Total read count	:	26
Mature read count	:	20
Loop read count	:	0
Star read count	:	6

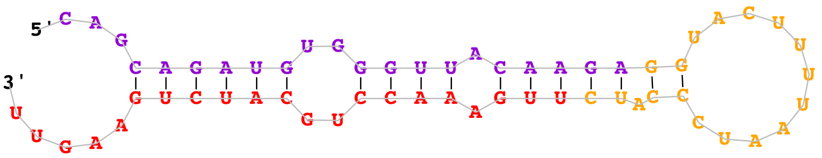

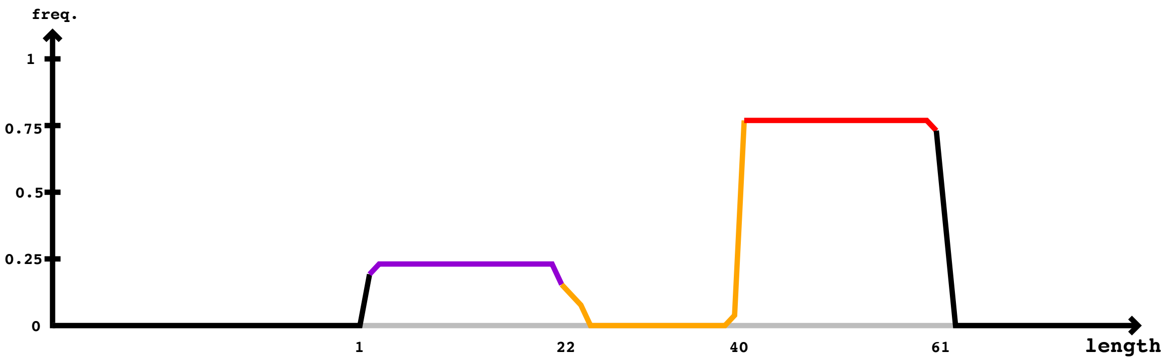

Star Mature

```
5'- uccuuagcaccuuguauucuguggaaggugg cagcagauguggguuacaaga gguacuuuuaaucccauc uugaaaccugcaucugaaguu aucuucugcuucagcugugc   -3'   obs
    uccuuagcaccuuguauucuguggaagguggcagcagauguggguuacaagagguacuuuuaaucccaucuugaaaccugcaucugaaguuaucuucugcuucagcugugc          exp
    ......((((...((.....(..(((((((((..((((((..((((.(((((((..........)).))))).))))..))))))..)))))))))..)....)).))))   reads   mm   sample
    ..............................cagcagauguggguuacaag............................................................   2       0    seq
    ..............................cagcagauguggguuacaaga...........................................................   2       0    seq
    ..............................cagcagauguggguuacaagagA.........................................................   1       1    seq
    ...............................agcagauguggguuacaagagg.........................................................   1       0    seq
    ....................................................................cuugaaaccugcaucugaaguu....................   1       0    seq
    .....................................................................uugaaaccugcaucugaagu.....................   1       0    seq
    .....................................................................uugaaaccugcaucugaaguu....................   18      0    seq
```

Provisional ID	:	mun-miR-15b
Score total	:	1559.2
Score for star read(s)	:	3.9
Score for read counts	:	1549.5
Score for mfe	:	1.2
Score for randfold	:	1.6
Score for cons. seed	:	3
Total read count	:	3051
Mature read count	:	3046
Loop read count	:	0
Star read count	:	5

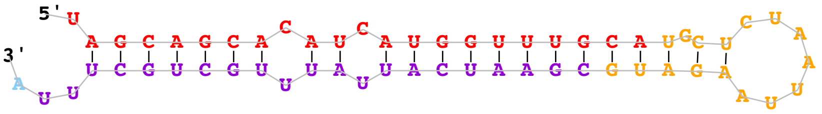

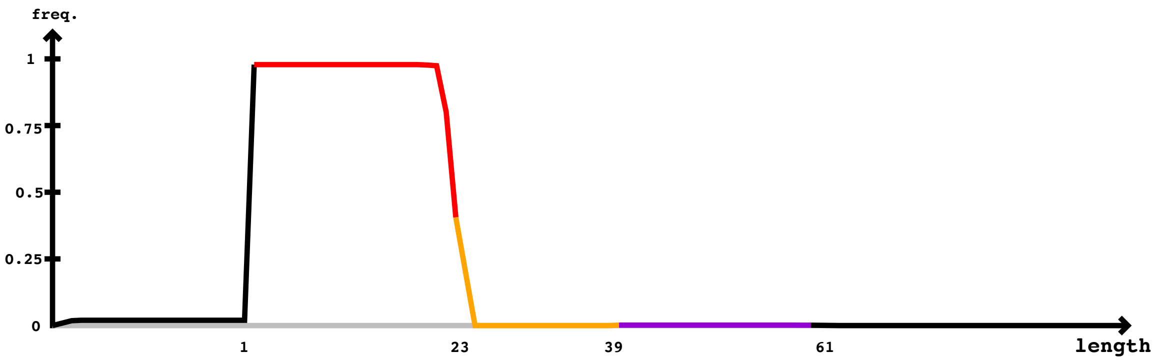

Mature **Star**

```
5'- uugaggccuuaaaguacucuagcagcacaucaugguuugcaugcucuaauuaagaugcgaaucauuauuugcugcuuuagaaauuuaaggaagagaaacaguugaagaua  -3'   obs
    uugaggccuuaaaguacucuagcagcacaucaugguuugcaugcucuaauuaagaugcgaaucauuauuugcugcuuuagaaauuuaaggaagagaaacaguugaagaua         exp
    ......(((((((...((..(((((((.((.(((((((((((.((.......))))))))))))).)).)))))))..))...)))))))....................  reads   mm   sample
    uugaggccuuaGaguacuc...........................................................................................  1       1    seq
    uugaggccuuaaaguacuc...........................................................................................  56      0    seq
    .ugaggccuuaaaguacuc...........................................................................................  5       0    seq
    ...................uagcagcacaucaugguu.........................................................................  5       0    seq
    ...................uagcagcacaucaugguuu........................................................................  8       0    seq
    ...................uagcaCcacaucaugguuug.......................................................................  1       1    seq
    ...................uagcagcacaucaugCuuug.......................................................................  1       1    seq
    ...................uagcagcacaucauggGuug.......................................................................  1       1    seq
    ...................uGgcagcacaucaugguuug.......................................................................  1       1    seq
    ...................uagcagcacaucaugguAug.......................................................................  1       1    seq
    ...................Cagcagcacaucaugguuug.......................................................................  1       1    seq
    ...................uagcagcacaucaugguuug.......................................................................  537     0    seq
    ...................uagcaAcacaucaugguuugc......................................................................  1       1    seq
    ...................uagcagcacaucaugguuugG......................................................................  1       1    seq
    ...................uagcagcacaucaugUuuugc......................................................................  1       1    seq
    ...................uagcagcacaucaugguuugU......................................................................  798     1    seq
    ...................Cagcagcacaucaugguuugc......................................................................  1       1    seq
    ...................uagcagcacaucaugguGugc......................................................................  1       1    seq
    ...................uagcagcacaucaugguuugc......................................................................  419     0    seq
    ...................uagcagcacaucaugguuugA......................................................................  5       1    seq
    ...................uagcagcacaucaugguuugUa.....................................................................  1180    1    seq
    ...................uagcagcacaucaugguuugca.....................................................................  64      0    seq
    ...................uagcagcacaucaugguuugcau....................................................................  3       0    seq
    ...................uagcagcacaucaugguuugcaA....................................................................  1       1    seq
    ...................uagcagcacaucaugguuugUau....................................................................  15      1    seq
    .........................................................cgaaucauuauuugcugcu..................................  1       0    seq
    .........................................................cgaaucauuauuugcugcuuu................................  3       0    seq
    .........................................................cgaaucauuauuugcugcuuuU...............................  1       1    seq
```

Provisional ID	:	mun-miR-301a
Score total	:	4.5
Score for star read(s)	:	-1.3
Score for read counts	:	0
Score for mfe	:	1.2
Score for randfold	:	1.6
Score for cons. seed	:	3
Total read count	:	403
Mature read count	:	403
Loop read count	:	0
Star read count	:	0

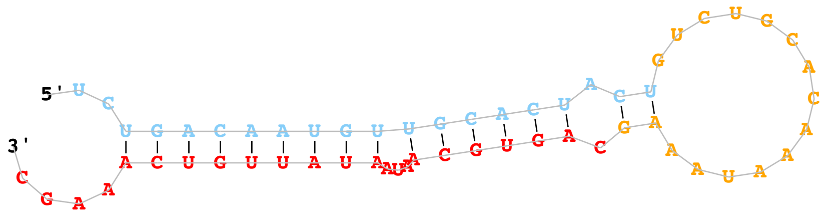

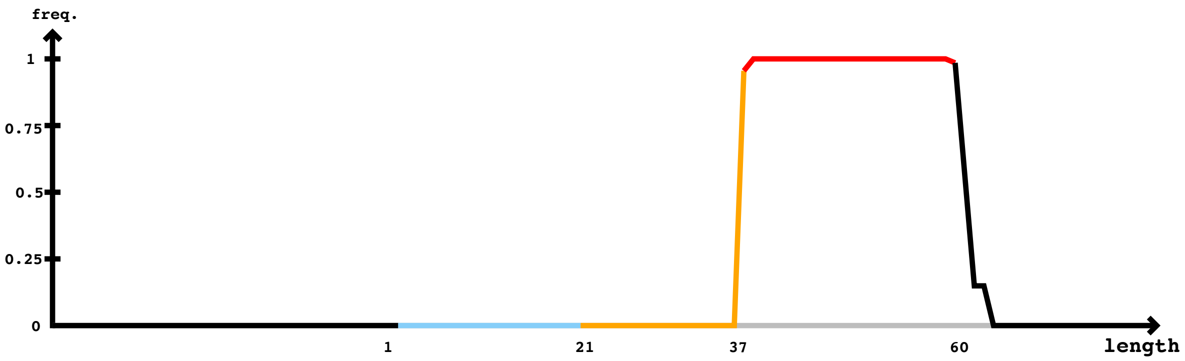

Star Mature

```
5'- gcagaacgugacaguggcugcugguaucgcuggcucugacaauguugcacuacugucugcacaaauaaagcagugcaauaauauugucaaagcauuugguuccaguccuaaua   -3'    exp
    ((...((......)).)).(((((.((((...(((.(((((((((((((((.((.............)).))))))...))))))))).)))...)))).))))).......    reads     mm        sample
    ......................................................................cagugcaauaauauugucaaag.....................    6         0         seq
    ......................................................................cagugcaauaauauuCucaaagc....................    1         1         seq
    ......................................................................cagugcaauaauauugucaaagc....................    306       0         seq
    ......................................................................cagugcaauaauauugucUaagc....................    1         1         seq
    ......................................................................cagugcaauaUuauugucaaagc....................    1         1         seq
    ......................................................................cagugcaauaauauugucaaagcU...................    13        1         seq
    ......................................................................cagugcaauaauauugucaaagcauu.................    2         0         seq
    ......................................................................cagugcaauaGuauugucaaagcauu.................    55        1         seq
    .......................................................................agugcaauaauauugucaaagc....................    13        0         seq
    .......................................................................agugcaauaauauugucaaagcU...................    2         1         seq
    .......................................................................agugcaauaGuauugucaaagcauu.................    3         1         seq
```

Provisional ID	:	mun-novel74
Score total	:	5.3
Score for star read(s)	:	-1.3
Score for read counts	:	0
Score for mfe	:	2
Score for randfold	:	1.6
Score for cons. seed	:	3
Total read count	:	244
Mature read count	:	244
Loop read count	:	0
Star read count	:	0

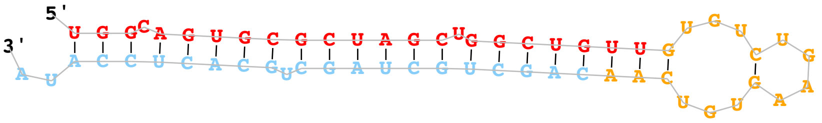

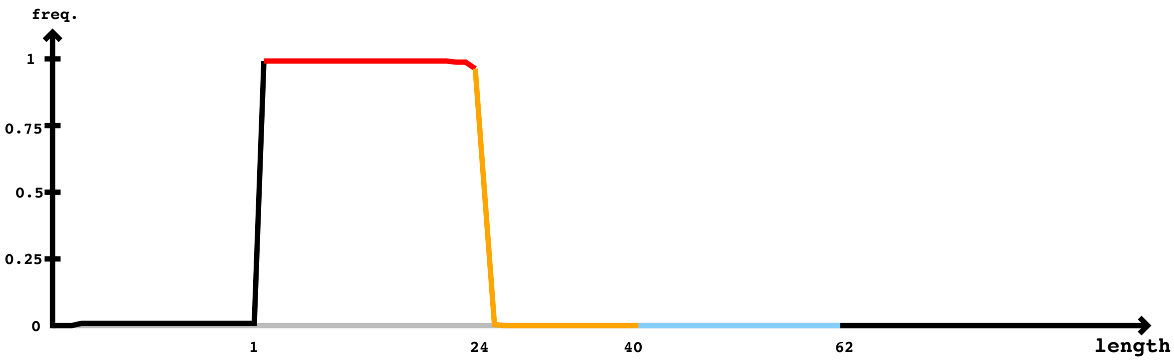

Mature Star

```
5'- cucaagaggauguaacagucuggcagugcgcuagcuggcuguugugucugaagugucaacagcugcuagcugcacuccauauuguuucauucaaaagcaucguggaaaacuc   -3'    exp
    .......(((((.((((((.(((.(((((((((((.((((((((...(....)...)))))))))))))).)))))))).)))))).))))).....................    reads    mm    sample
    .ucaagaggauguaacaguc............................................................................................    2    0    seq
    ....................uggcagugcgcuagcuggcu........................................................................    1    0    seq
    ....................uggcagugcgcuagcuggcugu......................................................................    6    0    seq
    ....................uggcagugcgcuagcuggUuguu.....................................................................    1    1    seq
    ....................uggcagugcgcuagcuggcuguu.....................................................................    230    0    seq
    ....................uggcagugcgAuagcuggcuguu.....................................................................    1    1    seq
    ....................uggcagugcgcuagcuggcuguA.....................................................................    1    1    seq
    ....................uggcagugcgcuagcuggcuguug....................................................................    1    0    seq
    ....................uggcagugcgcuagcuggcuguuU....................................................................    2    1    seq
    ....................uggcagugcgcuagcuggcuguugu...................................................................    1    0    seq
```

Provisional ID	:	mun-miR-129
Score total	:	225.4
Score for star read(s)	:	3.9
Score for read counts	:	214.8
Score for mfe	:	2.2
Score for randfold	:	1.6
Score for cons. seed	:	3
Total read count	:	433
Mature read count	:	326
Loop read count	:	0
Star read count	:	107

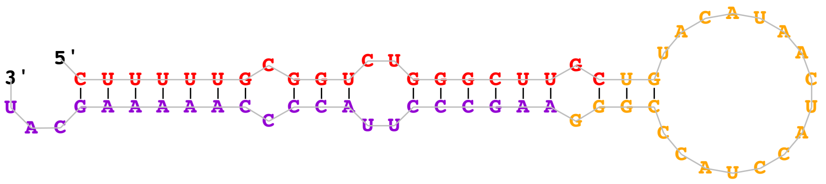

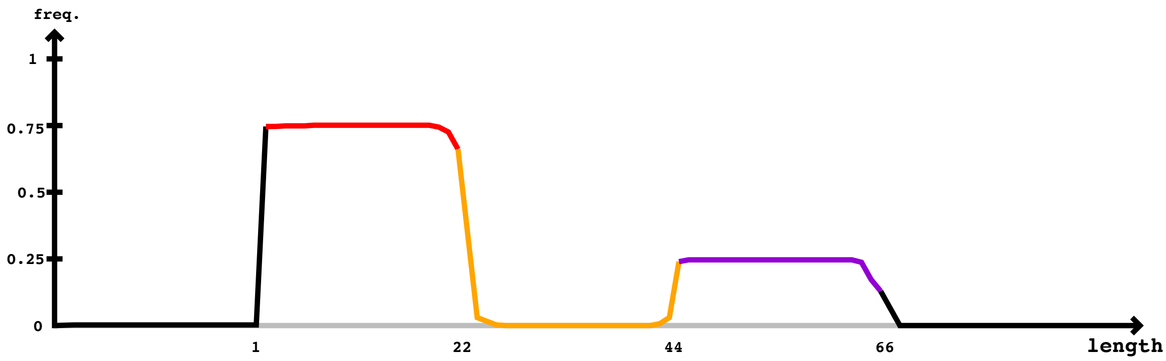

Mature **Star**

```
5'- agacuuuguccuucgcgaaucuuuuugcggucugggcuugcuguacauaacuaccuacccgggaagcccuuaccccaaaaagcauucgcggagggcgcauucgccgaagga  -3'  obs
    agacuuuguccuucgcgaaucuuuuugcggucugggcuugcuguacauaacuaccuacccgggaagcccuuaccccaaaaagcauucgcggagggcgcauucgccgaagga       exp
    .((.(.(((((((((((((((((((((.(((..((((((.(((................))).))))))..))).))))))).)))))))))))))).).)).........    reads  mm  sample
    agacuuuguccuucgcgaau...........................................................................................    1      0   seq
    ....................cuuuuugcggucugggcu.........................................................................    3      0   seq
    ....................cuuuuugcggucugggcuu........................................................................    8      0   seq
    ....................cuuuuugcggucugggcuuU.......................................................................    2      1   seq
    ....................cuuuuugcggucugggcuug.......................................................................    25     0   seq
    ....................Auuuuugcggucugggcuug.......................................................................    1      1   seq
    ....................cuuuuugcggucugggcuugA......................................................................    2      1   seq
    ....................cuuuuugcggucugggcuugG......................................................................    1      1   seq
    ....................cuuuuugcggucugggcuugc......................................................................    226    0   seq
    ....................cuuuuugcggucugggAuugc......................................................................    1      1   seq
    ....................cuuuuugcggucCgggcuugc......................................................................    2      1   seq
    ....................cuuuuugcggucugggcuugU......................................................................    13     1   seq
    ....................cuuuuugcggCcugggcuugcu.....................................................................    1      1   seq
    ....................cuuuuugcggucugggcuugcA.....................................................................    6      1   seq
    ....................cuuuuugcggucugggcuugcu.....................................................................    21     0   seq
    ....................cuuuuugcggucugggcuugcC.....................................................................    1      1   seq
    ....................cuuuuugcggucugggcuugcuU....................................................................    5      1   seq
    ....................cuuuuugcggucugggcuugcuA....................................................................    1      1   seq
    ....................cuuuuugcggucugggcuugcuUu...................................................................    1      1   seq
    ....................cuuuuugcggucugggcuugcugu...................................................................    3      0   seq
    ....................cuuuuugcggucugggcuugcugC...................................................................    1      1   seq
    ......................uuuugcggucugggcuugcugua..................................................................    1      0   seq
    .........................ugcggucugggcuugcugu...................................................................    1      0   seq
    .............................................................ggaagcccuuaccccaaaaagc............................    3      0   seq
    ..............................................................gaagcccuuaccccaaaaag.............................    1      0   seq
    ..............................................................gaagcccuuaccccaaaaagc............................    8      0   seq
    ..............................................................gaagcccuuaccccaaaaagcC...........................    1      1   seq
    ...............................................................aagcccuuaccccaaaaag.............................    3      0   seq
    ...............................................................aagcccuuaccccaaaaagc............................    17     0   seq
    ...............................................................aagcccuuaccccaaaaagcC...........................    6      1   seq
    ...............................................................aagcccuuaccccaaaaagca...........................    11     0   seq
    ...............................................................aagcccuuaccccGaaaagca...........................    1      1   seq
    ...............................................................aagcccuuaccccaaaaagcau..........................    47     0   seq
```

Mature **Star**

```
agacuuuguccuucgcgaaucuuuuugcggucugggcuugcuguacauaaacuaccuacccgggaagcccuuaccccaaaaagcauucgcggagggcgcauucgccgaagga
................................................................aagcccuuaccccaaaaagcaC..........................    1    1    seq
................................................................aagcccuuaccccaaaaagcCu..........................    4    1    seq
................................................................aagcccuuaccccaaaaagcauu.........................    1    0    seq
.................................................................agcccuuaccccaaaaagcCu..........................    1    1    seq
.................................................................agcccuuaccccaaaaagcauu.........................    2    0    seq
```

Provisional ID	:	mun-novel47
Score total	:	1.5
Score for star read(s)	:	-1.3
Score for read counts	:	0
Score for mfe	:	2
Score for randfold	:	-2.2
Score for cons. seed	:	3
Total read count	:	30
Mature read count	:	30
Loop read count	:	0
Star read count	:	0

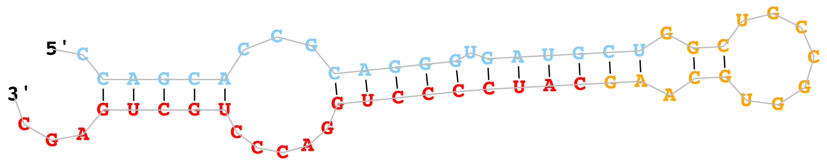

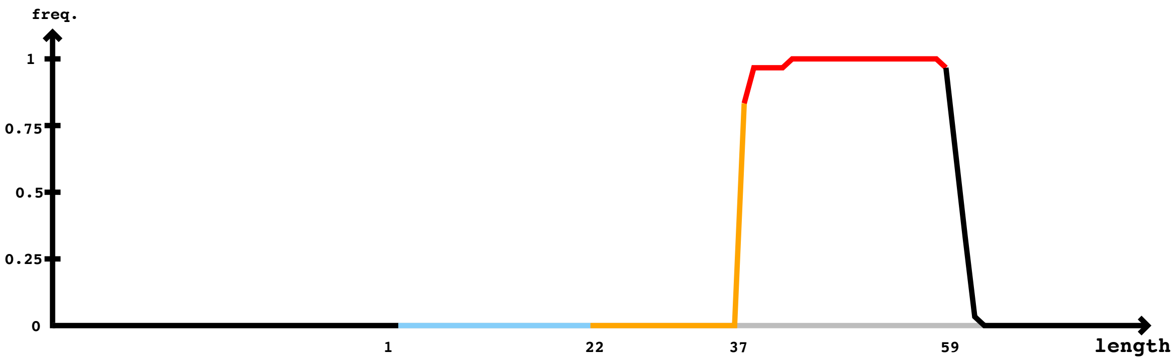

Star Mature

```
5'- ggaacgacacggagccgcugugccgaggucagugccagcaccgcagggugaugcuggcugccggugcaagcauccccuggacccugcugagcccugccugccucuugcucau   -3'    exp
    ...........((((....(.((..(((.(((.(((((((...(((((.((((((.((.......)).))))))))))).....))))).)).)))))))))....))))..     reads    mm    sample
    ......................................................................cauccccuggacccugcugag.....................     1        0     seq
    ......................................................................Aauccccuggacccugcugagc....................     1        1     seq
    ......................................................................cauccccuggacccugcugagc....................     8        0     seq
    ......................................................................cauccccuggacccugcugagcc...................     7        0     seq
    ......................................................................cauccccuggacccugcugagccU..................     6        1     seq
    ......................................................................cauccccuggacccugcugagccc..................     2        0     seq
    .......................................................................auccccuggacccugcugagc....................     2        0     seq
    .......................................................................auccccuggacccugcugagccA..................     1        1     seq
    .......................................................................auccccuggacccugcugagcccu.................     1        0     seq
    ...........................................................................ccuggacccugcugagcc...................     1        0     seq
```

Provisional ID		mun-miR-33
Score total	:	345.7
Score for star read(s)	:	3.9
Score for read counts	:	335.1
Score for mfe	:	2.1
Score for randfold	:	1.6
Score for cons. seed	:	3
Total read count	:	669
Mature read count	:	487
Loop read count	:	0
Star read count	:	182

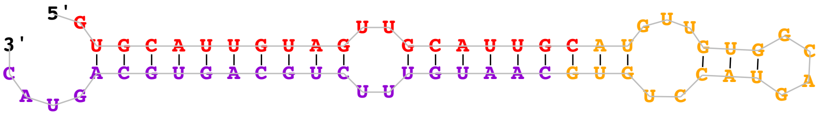

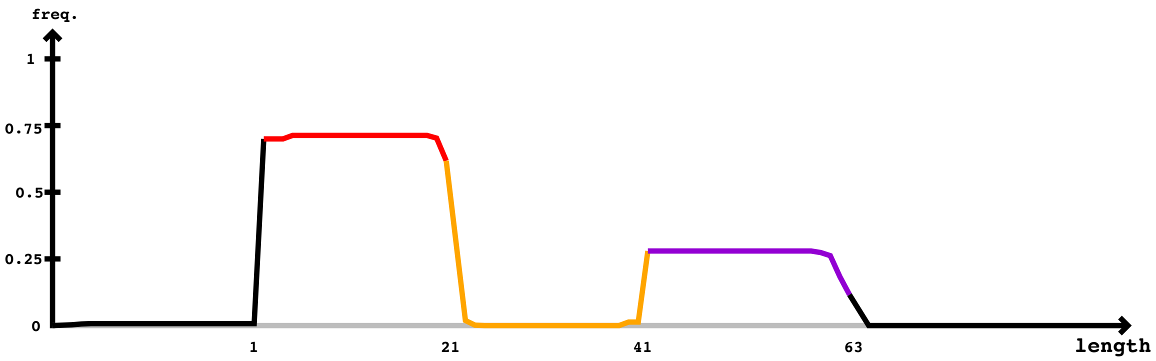

Mature Star

```
5'- ccuccuggguggcagcuguagugcauuguaguugcauugcauguuguggcaguaccugugcaauguuucugcagugcaguacagaggcacucugcuucugcagugugagu   -3'   obs
    ccuccuggguggcagcuguagugcauuguaguugcauugcauguuguggcaguaccugugcaauguuucugcagugcaguacagaggcacucugcuucugcagugugagu         exp
    .(((...((((.(..(((((.((((((((((..(((((((((...(((....)))..)))))))))..)))))))))).)))))..)))))((((....))))...))).    reads   mm   sample
    ccuccuggguggcagcugua..........................................................................................    2       0    seq
    .cuccuggguggcagcugua..........................................................................................    2       0    seq
    ..uccuggguggcagcugua..........................................................................................    1       0    seq
    ....................gugcauuguaguugcauu........................................................................    7       0    seq
    ....................guUcauuguaguugcauug.......................................................................    1       1    seq
    ....................gugcauuguaguuUcauug.......................................................................    1       1    seq
    ....................gugcauuguaguugcauug.......................................................................    56      0    seq
    ....................gugcauuguaguuUcauugc......................................................................    1       1    seq
    ....................gugcauuguaguugcauugc......................................................................    186     0    seq
    ....................gugcauuguaguugUauugc......................................................................    1       1    seq
    ....................gugcauuguaguugcauugU......................................................................    13      1    seq
    ....................gugcauuguaguuAcauugc......................................................................    2       1    seq
    ....................gugcauuguaguugcauugcC.....................................................................    1       1    seq
    ....................Cugcauuguaguugcauugca.....................................................................    1       1    seq
    ....................gugcauuguaUuugcauugca.....................................................................    1       1    seq
    ....................gugUauuguaguugcauugca.....................................................................    1       1    seq
    ....................gugcauuguaguugcauugcU.....................................................................    8       1    seq
    ....................gugcauuguaguugcauuUca.....................................................................    1       1    seq
    ....................gugcauuguaguugcauugUa.....................................................................    1       1    seq
    ....................gugGauuguaguugcauugca.....................................................................    1       1    seq
    ....................gugcauuguaguugUauugca.....................................................................    5       1    seq
    ....................gugcauuguaguugcauugca.....................................................................    177     0    seq
    ....................gugcauuguaguugcauugcau....................................................................    1       0    seq
    ....................gugcauuguaguugcauugcaA....................................................................    11      1    seq
    ....................gugcauuguaguugcauugcauU...................................................................    1       1    seq
    .......................cauuguaguugcauugca......................................................................    9       0    seq
    ..........................................................ugcaauguuucugcagugca................................    1       0    seq
    ..........................................................ugcaauguuucugcagugcagu..............................    8       0    seq
    ............................................................caauguuucugcagugca................................    3       0    seq
    ............................................................caauguuucugcagugcag...............................    6       0    seq
    ............................................................caauguuucugcagugcaU...............................    2       1    seq
    ............................................................caauguuucugcagugcagu..............................    46      0    seq
    ............................................................caauguuucugcagugcagua.............................    43      0    seq
```

```
                Mature                          Star
ccuccuggguggcagcuguagugcauuguaguugcauugcauguuguggcaguaccugugcaauguuucugcagugcaguacagaggcacucugcuucugcagugugagu
............................................................caauguuucugcagugcaguU.............................   3    1    seq
............................................................caauguuucugcagugcaguaA............................   13   1    seq
............................................................caauguuucugcagugcaguaU............................   65   1    seq
............................................................caauguuucugcagugcaguaAa...........................   1    1    seq
```

Provisional ID	:	mun-miR-140
Score total	:	10549
Score for star read(s)	:	3.9
Score for read counts	:	10538.2
Score for mfe	:	2.3
Score for randfold	:	1.6
Score for cons. seed	:	3
Total read count	:	20682
Mature read count	:	19789
Loop read count	:	1
Star read count	:	892

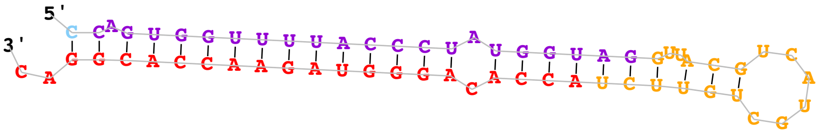

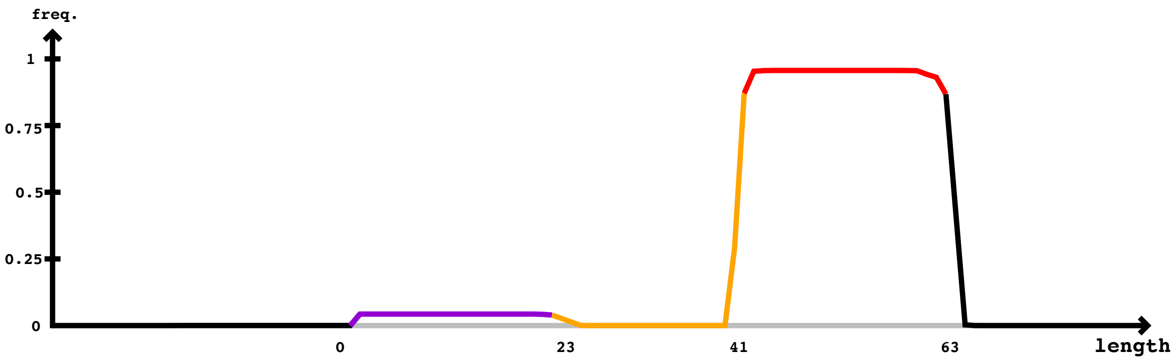

Star Mature

```
5'- gcuccugcucuccaccucucuguguccugccagugguuuuacccuaugguagguuacgucaugcuguucuaccacaggguagaaccacggacaggaugccggggcugccucu   -3'   obs
    gcuccugcucuccaccucucuguguccugccagugguuuuacccuaugguagguuacgucaugcuguucuaccacaggguagaaccacggacaggaugccggggcugccucu         exp
    (((((.((....(((......)))(((((((.(((((((((((((.(((((((..(((......))))))))))).))))))))))))))).))))).)).))))).......   reads   mm   sample
    ...........ccaccucucuguguccugc..................................................................................   3   0   seq
    ............caccucucuguguccugc..................................................................................   2   0   seq
    ..............................cagugguuuuacccuaug................................................................   1   0   seq
    ..............................cagugguuuuacccuaugg...............................................................   20   0   seq
    ..............................cagugguuuuacccuauggu..............................................................   51   0   seq
    ..............................cagugguuuuacccuaugguU.............................................................   2   1   seq
    ..............................cagugguuuuacccuauggua.............................................................   56   0   seq
    ..............................cagugguuuuacccuaugguC.............................................................   2   1   seq
    ..............................cagCgguuuuacccuaugguag............................................................   1   1   seq
    ..............................cagugguuuuacccuaugguaU............................................................   2   1   seq
    ..............................cagugguuuuacccuaugguaA............................................................   18   1   seq
    ..............................cagugguuuuacccuaugguaC............................................................   25   1   seq
    ..............................cagGgguuuuacccuaugguag............................................................   1   1   seq
    ..............................cagugguuuuacccuaugguag............................................................   573   0   seq
    ..............................cagugguuuuacccuauUguag............................................................   1   1   seq
    ..............................cagugguuuuacccuGugguag............................................................   2   1   seq
    ..............................cagugguuuuacccuaugguagA...........................................................   8   1   seq
    ..............................cagugguuuuacccuaugguagU...........................................................   111   1   seq
    ..............................cagugguuuuacccuaugguagg...........................................................   12   0   seq
    ..............................cagugguuuuacccuaugguagC...........................................................   1   1   seq
    ..............................cagugguuuuacccuaugguaggu..........................................................   4   0   seq
    ..............................cagugguuuuacccuaugguaggAu.........................................................   1   1   seq
    ....................................................guuacgucaugcuguucu..........................................   1   0   seq
    .....................................................................uaccacaggguagaaccU.........................   2   1   seq
    .....................................................................uaccacaggguagaacca.........................   4   0   seq
    .....................................................................uaccacaggguagaaccac........................   9   0   seq
    .....................................................................uaccacaggguagaaccacg.......................   156   0   seq
    .....................................................................uaccacagUguagaaccacg.......................   1   1   seq
    .....................................................................uaccacaggguagaaccacU.......................   10   1   seq
    .....................................................................uaccacaggguagaaccacA.......................   2   1   seq
    .....................................................................uGccacaggguagaaccacg.......................   7   1   seq
    .....................................................................uaccacagCguagaaccacg.......................   1   1   seq
    .....................................................................uaccacGggguagaaccacg.......................   1   1   seq
```

```
                              Star                                    Mature
gcuccugcucuccaccucucuguguccugccagugguuuuacccuaugguagguuacgucaugcuguucuaccacaggguagaaccacggacaggaugccggggcugccucu
.....................................................................uaccacaggguagaaccacC.......................    1      1     seq
.....................................................................uaccacaggguagaaccaUg.......................    2      1     seq
.....................................................................Gaccacaggguagaaccacg.......................    1      1     seq
.....................................................................uaccacagggCagaaccacg.......................    1      1     seq
.....................................................................uaccacagggGagaaccacg.......................    1      1     seq
.....................................................................uaccacaggguagaaccacgA......................    4      1     seq
.....................................................................uaccacaggguGgaaccacgg......................    1      1     seq
.....................................................................uaccacaggguagaaccacgg......................    161    0     seq
.....................................................................uGccacaggguagaaccacgg......................    1      1     seq
.....................................................................uaccacGggguagaaccacgg......................    1      1     seq
.....................................................................uaccacaggguagaaccacgU......................    6      1     seq
.....................................................................uaccacaggguagaaUcacgga.....................    2      1     seq
.....................................................................uaccacaggguagaaccaAgga.....................    1      1     seq
.....................................................................uaccacaggguagaaccacgga.....................    911    0     seq
.....................................................................Aaccacaggguagaaccacgga.....................    4      1     seq
.....................................................................uaccacaggguagaaccacggU.....................    14     1     seq
.....................................................................uaccacaggguagaaccacggG.....................    3      1     seq
.....................................................................uaccacagggGagaaccacgga.....................    1      1     seq
.....................................................................uaccacaAgguagaaccacgga.....................    1      1     seq
.....................................................................uaccacaggguagaaccaUgga.....................    1      1     seq
.....................................................................uaccacagggCagaaccacgga.....................    1      1     seq
.....................................................................uGccacaggguagaaccacgga.....................    13     1     seq
.....................................................................uaccacGggguagaaccacgga.....................    5      1     seq
.....................................................................Caccacaggguagaaccacgga.....................    1      1     seq
.....................................................................uaccacagggAagaaccacggac....................    1      1     seq
.....................................................................uaccacaggguagaaccaAggac....................    1      1     seq
.....................................................................uaccacaggguagaaccacggaA....................    1033   1     seq
.....................................................................uaccacaUgguagaaccacggac....................    1      1     seq
.....................................................................uaccacaggguagaaccacggaU....................    93     1     seq
.....................................................................uaccacaggguaUaaccacggac....................    1      1     seq
.....................................................................uaccacaggguagaaccCcggac....................    1      1     seq
.....................................................................uaccaAaggguagaaccacggac....................    1      1     seq
.....................................................................uaccacaggguagaaccacggac....................    1679   0     seq
.....................................................................uaccacaggguagaaccaUggac....................    1      1     seq
.....................................................................uGccacaggguagaaccacggac....................    5      1     seq
.....................................................................Caccacaggguagaaccacggac....................    1      1     seq
.....................................................................uaccacagggGagaaccacggac....................    2      1     seq
.....................................................................uaccacaggguagaaccacggaG....................    3      1     seq
.....................................................................uaccacaggguGgaaccacggac....................    3      1     seq
.....................................................................uacAacaggguagaaccacggac....................    1      1     seq
.....................................................................uaccacGggguagaaccacggac....................    10     1     seq
.....................................................................uaccacagggCagaaccacggac....................    1      1     seq
.....................................................................uaccacaggguagaaccacggaGa...................    14     1     seq
.....................................................................uaccaUaggguagaaccacggaca...................    1      1     seq
.....................................................................uaccacaggguagaaccaUggaca...................    1      1     seq
.....................................................................uaccacaggguagaaccacggacU...................    1649   1     seq
.....................................................................uaccacaggguagaaccacggaAa...................    49     1     seq
.....................................................................uGccacaggguagaaccacggaca...................    2      1     seq
.....................................................................uaccacaggguagaaccacggaca...................    117    0     seq
.....................................................................uaccacaggguagaaccacggacC...................    18     1     seq
.....................................................................uaccacaggguagaaccacggacG...................    8      1     seq
.....................................................................uaccacaggguagaaccacggacaU..................    4      1     seq
.....................................................................uaccacaggguagaaccacggacUg..................    1      1     seq
......................................................................Gccacaggguagaaccac........................    1      1     seq
......................................................................accacaggguagaaccac........................    4      0     seq
......................................................................accacaggguagaaccacg.......................    93     0     seq
......................................................................accacGggguagaaccacg.......................    1      1     seq
......................................................................accacaggguaUaaccacgg......................    1      1     seq
......................................................................accacaggguagaaccacgg......................    50     0     seq
......................................................................accacaggguagaaccacgU......................    4      1     seq
......................................................................accacaggguagaaUcacgga.....................    1      1     seq
......................................................................accacaggguagaaccacggU.....................    2      1     seq
......................................................................accacaggguagaaccacgga.....................    331    0     seq
......................................................................accacaggguagaaccacUga.....................    1      1     seq
......................................................................accacagggAagaaccacgga.....................    2      1     seq
......................................................................accacagggGagaaccacgga.....................    1      1     seq
......................................................................accacGggguagaaccacgga.....................    2      1     seq
......................................................................accacaggguagaaccacgUa.....................    1      1     seq
......................................................................accacaggguagaaccacUgac....................    2      1     seq
......................................................................accacaggguagaacAacggac....................    3      1     seq
```

Star Mature

```
gcuccugcucuccaccucucuguguccugccagugguuuuacccuaugguagguuacgucaugcuguucuaccacaggguagaaccacggacaggaugccggggcugccucu
......................................................................accacaggguagaaccacggGc....................  2     1  seq
......................................................................accacaggguagaaccacggaG....................  3     1  seq
......................................................................acAacaggguagaaccacggac....................  1     1  seq
......................................................................accacaggguagaaccacgCac....................  1     1  seq
......................................................................accacaCgguagaaccacggac....................  1     1  seq
......................................................................accacagUguagaaccacggac....................  2     1  seq
......................................................................accacaggguagaaccacggac....................  5757  0  seq
......................................................................accacGggguagaaccacggac....................  15    1  seq
......................................................................acGacaggguagaaccacggac....................  1     1  seq
......................................................................accacaggguagaaccacgAac....................  2     1  seq
......................................................................accacaggguGgaaccacggac....................  18    1  seq
......................................................................accacagAguagaaccacggac....................  1     1  seq
......................................................................accacaggguagUaccacggac....................  1     1  seq
......................................................................Gccacaggguagaaccacggac....................  5     1  seq
......................................................................accacaggguagaaUcacggac....................  1     1  seq
......................................................................accacagggCagaaccacggac....................  3     1  seq
......................................................................accacaUgguagaaccacggac....................  2     1  seq
......................................................................accacagggAagaaccacggac....................  5     1  seq
......................................................................accacaAgguagaaccacggac....................  1     1  seq
......................................................................accacagggGagaaccacggac....................  5     1  seq
......................................................................aAcacaggguagaaccacggac....................  2     1  seq
......................................................................accacaggguagaaccacggaA....................  263   1  seq
......................................................................accacaggguagaaccacAgac....................  1     1  seq
......................................................................accacaggguagaaccacggaU....................  82    1  seq
......................................................................accaAaggguagaaccacggac....................  1     1  seq
......................................................................accacaggguagaaccaAggac....................  1     1  seq
......................................................................accGcaggguagaaccacggac....................  2     1  seq
......................................................................Cccacaggguagaaccacggac....................  2     1  seq
......................................................................accacaggguagaaccaUggac....................  1     1  seq
......................................................................accacaggguagaGccacggaca...................  1     1  seq
......................................................................accacaggguagaaccacggCca...................  1     1  seq
......................................................................accacGggguagaaccacggaca...................  4     1  seq
......................................................................accacaggguagaaccacggacG...................  67    1  seq
......................................................................accacaggguagaaccacggaca...................  529   0  seq
......................................................................accacaggguagaaccacggaUa...................  4     1  seq
......................................................................accacagggCagaaccacggaca...................  1     1  seq
......................................................................accacaggguagaaccacggacU...................  4341  1  seq
......................................................................Gccacaggguagaaccacggaca...................  1     1  seq
......................................................................accacaggguGgaaccacggaca...................  4     1  seq
......................................................................accacaggguagaaccacggaAa...................  160   1  seq
......................................................................accacaggguagaaccacggaGa...................  5     1  seq
......................................................................accacaggguagaaccacggacC...................  132   1  seq
......................................................................accacaggguagaaccacggacUg..................  4     1  seq
......................................................................accacaggguagaaccacggacaA..................  11    1  seq
......................................................................accacaggguagaaccacggacaC..................  1     1  seq
......................................................................accacaggguagaaccacggacaU..................  25    1  seq
......................................................................accacaggguagaaccacggaAag..................  1     1  seq
......................................................................accacaggguagaaccacggacag..................  3     0  seq
......................................................................accacaggguagaaccacggaUag..................  1     1  seq
......................................................................accacaggguagaaccacggacagU.................  2     1  seq
.......................................................................ccacGggguagaaccacg.......................  1     1  seq
.......................................................................ccacaggguagaaccacg.......................  1     0  seq
.......................................................................ccacaggguagaaccacgga.....................  13    0  seq
.......................................................................ccacaggguagaaccacggac....................  143   0  seq
.......................................................................ccacaggguagaaccacggaG....................  1     1  seq
.......................................................................ccacGggguagaaccacggac....................  1     1  seq
.......................................................................ccacaggguGgaaccacggac....................  2     1  seq
.......................................................................ccacaggguagaaccacggaU....................  2     1  seq
.......................................................................ccacaggguagaaccacggaA....................  10    1  seq
.......................................................................ccacaggguagaacAacggaca...................  1     1  seq
.......................................................................ccacaggguagaaccacggacG...................  7     1  seq
.......................................................................ccacaggguagaaccacggacU...................  1478  1  seq
.......................................................................ccacaggguagaaccacggaca...................  21    0  seq
.......................................................................ccacaggguagaaccacggacC...................  27    1  seq
.......................................................................ccacaggguagaaccacggaAa...................  2     1  seq
.......................................................................ccacaggguagaaccacggacUg..................  1     1  seq
........................................................................cacaggguagaaccacgga.....................  2     0  seq
........................................................................cacaggguagaaccacggac....................  1     0  seq
........................................................................cacaggguagaaccacggacU...................  45    1  seq
........................................................................cacaggguagaaccacggaca...................  1     0  seq
```

```
                             Star                        Mature
gcuccugcucuccaccucucuguguccugccagugguuuuacccuaugguagguuacgucaugcuguucuaccacaggguagaaccacggacaggaugccggggcugccucu
........................................................................cacaggguagaaccacggacC...................	2	1	seq
........................................................................cacaggguagaaccacggacag..................	1	0	seq
.........................................................................acaggguagaaccacgga.....................	1	0	seq
.........................................................................acaggguagaaccacggaA....................	1	1	seq
.........................................................................acaggguagaaccacggac....................	3	0	seq
.........................................................................acaggguagaaccacggacU...................	7	1	seq
.........................................................................acaggguagaaccacggaca...................	1	0	seq
```

Provisional ID	:	mun-novel61
Score total	:	1.9
Score for star read(s)	:	-1.3
Score for read counts	:	0
Score for mfe	:	2.2
Score for randfold	:	1.6
Score for cons. seed	:	-0.6
Total read count	:	15
Mature read count	:	15
Loop read count	:	0
Star read count	:	0

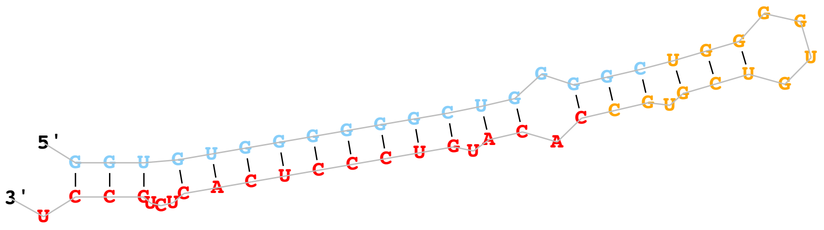

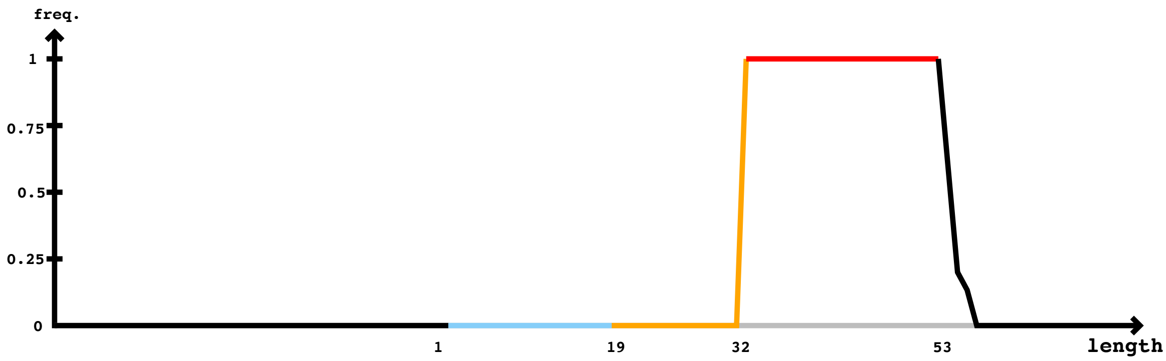

Star Mature

```
5'- gaaaugaaggcagugcaugagaaccuggcaggugaggagggugugggggggcuggggcuggggugucgugccacaugucccucacucugccuccugccugcagguguccgga   -3'    exp
    .................((.(((((((.(((((.((((((..(((((((((((.((((((....))).))).)).)))))))))....)))))))))))))))).))))..    reads    mm    sample
    ......................................................................cacaugucccucacucugccA....................    2        1     seq
    ......................................................................cacaugucccucacucugccu....................    5        0     seq
    ......................................................................cacaugucccucCcucugccu....................    5        1     seq
    ......................................................................cacaugucccucacucugccucc..................    1        0     seq
    ......................................................................cacaugucccucacucugccuccu.................    2        0     seq
```

Provisional ID	:	mun-miR-128
Score total	:	5029.4
Score for star read(s)	:	3.9
Score for read counts	:	5019.4
Score for mfe	:	1.6
Score for randfold	:	1.6
Score for cons. seed	:	3
Total read count	:	9857
Mature read count	:	9756
Loop read count	:	0
Star read count	:	101

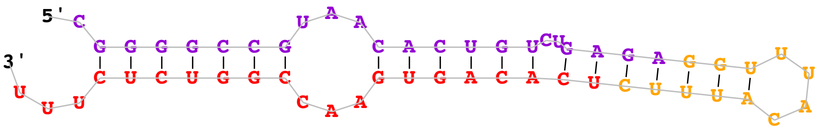

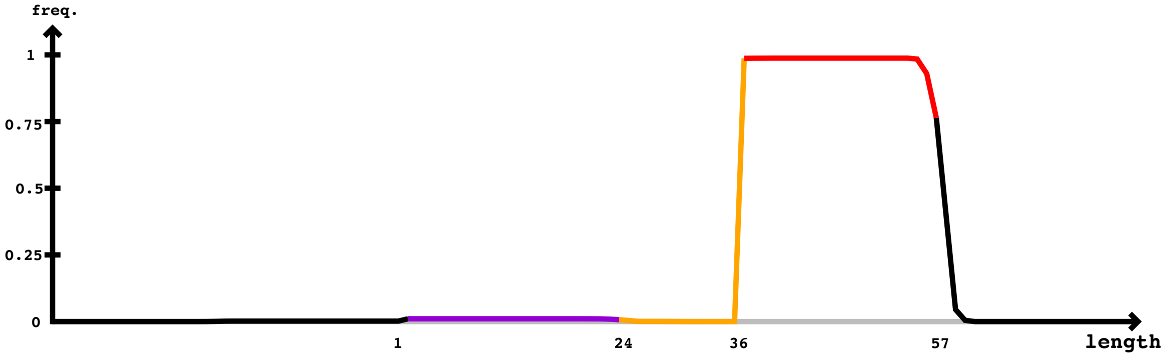

Star   Mature

```
5'- ggacuggccugaguuuauuuaugagcuguuggauucggggccguaacacugucugagagguuuacauuucucacagugaaccggucucuuuuucagcugcuuccuggcuuc  -3'   obs
    ggacuggccugaguuuauuuaugagcuguuggauucggggccguaacacugucugagagguuuacauuucucacagugaaccggucucuuuuucagcugcuuccuggcuuc         exp
    .....((((.............((((.((((((...(((((((...((((((..(((((((....)))))))))))))...)))))))...)))))).))))...))))..   reads   mm   sample
    ..............uuauuuaugagcuguuggauu............................................................................   1       0    seq
    ...............uauuuaugagcuguuggauu............................................................................   6       0    seq
    ................auuuaugagcuguuggauu............................................................................   9       0    seq
    .................uuuaugagcuguuggauu............................................................................   2       0    seq
    ...................................cggggccguaacacugucu.........................................................   1       0    seq
    ...................................cggggccguaacacugucug........................................................   2       0    seq
    ...................................cggggccguaacacugucuga.......................................................   10      0    seq
    ...................................cggggccguaacacugucugag......................................................   21      0    seq
    ...................................cggggccguaacacugucugagU.....................................................   1       1    seq
    ...................................cggggccguaacacugucugaga.....................................................   57      0    seq
    ...................................cggggccguaacacugucugagaA....................................................   3       1    seq
    ...................................cggggccguaacacugucugagag....................................................   1       0    seq
    ...................................cggggccguaacacugucugagagguu.................................................   2       0    seq
    ...................................cggggccguaacacugucugagagguuu................................................   3       0    seq
    ...................................cggggccguaacacugucugagagguuua...............................................   1       0    seq
    ...................................cggggccguaacacugucugagGgguuua...............................................   1       1    seq
    ....................................................................uucucacagugaaccggucucuuu...................   1       0    seq
    .....................................................................cucacagugaaccggucucuuu...................   1       0    seq
    ......................................................................ucacagugaaccggGcuc.......................   1       1    seq
    ......................................................................ucacagugaaccggucuc.......................   31      0    seq
    ......................................................................ucacagugaaccAgucuc.......................   1       1    seq
    ......................................................................Ccacagugaaccggucucu......................   1       1    seq
    ......................................................................Acacagugaaccggucucu......................   1       1    seq
    ......................................................................ucacagugaaccggucucA......................   11      1    seq
    ......................................................................ucacagugaaccggucucu......................   514     0    seq
    ......................................................................ucacagugaaccggCcucu......................   1       1    seq
    ......................................................................ucacagugaaccggucucC......................   1       1    seq
    ......................................................................ucacagugaaccggucuUu......................   6       1    seq
    ......................................................................ucacagugaaccgCucucu......................   1       1    seq
    ......................................................................ucaUagugaaccggucucu......................   1       1    seq
    ......................................................................ucacagugaaccggucAcu......................   1       1    seq
    ......................................................................ucacagugaaccUgucucuu.....................   1       1    seq
    ......................................................................ucCcagugaaccggucucuu.....................   1       1    seq
```

Star Mature

```
ggacuggccugaguuuauuuaugagcuguuggauucggggccguaacacugucugagaggguuuacauuucucacagugaaccggucucuuuucagcugcuuccuggcuc
......................................................................ucacagCgaaccggucucuu....................	1	1	seq
......................................................................ucacagugaaccggGcucuu....................	2	1	seq
......................................................................ucacagugaaccggucucuA....................	6	1	seq
......................................................................ucacagGgaaccggucucuu....................	1	1	seq
......................................................................ucacagugaaccggucucuC....................	5	1	seq
......................................................................ucaAagugaaccggucucuu....................	1	1	seq
......................................................................ucacagugaaccggAcucuu....................	1	1	seq
......................................................................ucacaAugaaccggucucuu....................	1	1	seq
......................................................................ucacaUugaaccggucucuu....................	1	1	seq
......................................................................ucacagugaaccggucuUuu....................	5	1	seq
......................................................................ucacagugaaccggucucAu....................	1	1	seq
......................................................................ucacagugaaUcggucucuu....................	3	1	seq
......................................................................ucacagugaaccggucucuG....................	6	1	seq
......................................................................Gcacagugaaccggucucuu....................	1	1	seq
......................................................................ucacagugaacUggucucuu....................	1	1	seq
......................................................................ucacagugaaccggucucuu....................	1603	0	seq
......................................................................ucacagugaUccggucucuuu...................	1	1	seq
......................................................................ucacagugaaUcggucucuuu...................	4	1	seq
......................................................................ucacagugaaccggCcucuuu...................	2	1	seq
......................................................................ucacaguAaaccggucucuuu...................	1	1	seq
......................................................................ucaUagugaaccggucucuuu...................	1	1	seq
......................................................................ucacagugaaccggucucuuu...................	5401	0	seq
......................................................................ucacagugaaccgguAucuuu...................	1	1	seq
......................................................................ucacaCugaaccggucucuuu...................	1	1	seq
......................................................................ucacagugaaccggucuUuuu...................	10	1	seq
......................................................................ucacagugaaccAgucucuuu...................	1	1	seq
......................................................................ucacagugaaccggucuAuuu...................	1	1	seq
......................................................................ucacaguUaaccggucucuuu...................	1	1	seq
......................................................................ucacagugaaccggucucCuu...................	1	1	seq
......................................................................Ccacagugaaccggucucuuu...................	3	1	seq
......................................................................ucacaAugaaccggucucuuu...................	2	1	seq
......................................................................ucacaUugaaccggucucuuu...................	2	1	seq
......................................................................ucacagugaaccggucucuAu...................	22	1	seq
......................................................................ucacagugaaccggucucuuA...................	33	1	seq
......................................................................ucacagugaaccggAcucuuu...................	2	1	seq
......................................................................ucacagugaaccggucucuuG...................	9	1	seq
......................................................................ucacagugaaccggucucuGu...................	1	1	seq
......................................................................ucacagugaaccggucuGuuu...................	2	1	seq
......................................................................ucacagugaaccgguGucuuu...................	1	1	seq
......................................................................Gcacagugaaccggucucuuu...................	1	1	seq
......................................................................ucacagugaaccggucucuCu...................	6	1	seq
......................................................................ucUcagugaaccggucucuuu...................	2	1	seq
......................................................................ucacagugaaccggucucuuC...................	33	1	seq
......................................................................ucacagugGaccggucucuuu...................	4	1	seq
......................................................................ucacagCgaaccggucucuuu...................	1	1	seq
......................................................................ucacagugaaccggGcucuuu...................	6	1	seq
......................................................................uUacagugaaccggucucuuu...................	2	1	seq
......................................................................Acacagugaaccggucucuuu...................	2	1	seq
......................................................................ucacagGgaaccggucucuuu...................	2	1	seq
......................................................................uGacagugaaccggucucuuu...................	2	1	seq
......................................................................ucacagugaaccggucucuuAu..................	41	1	seq
......................................................................ucacagAgaaccggucucuuuu..................	1	1	seq
......................................................................Acacagugaaccggucucuuuu..................	2	1	seq
......................................................................ucacagugaaccggucucuAuu..................	2	1	seq
......................................................................ucacagugaaccggucucGuuu..................	1	1	seq
......................................................................uAacagugaaccggucucuuuu..................	1	1	seq
......................................................................ucacagugaaccggucucuuuu..................	1256	0	seq
......................................................................uUacagugaaccggucucuuuu..................	1	1	seq
......................................................................uGacagugaaccggucucuuuu..................	1	1	seq
......................................................................ucacagugaaccggucucuuuC..................	46	1	seq
......................................................................Ccacagugaaccggucucuuuu..................	1	1	seq
......................................................................ucacagugaaUcggucucuuuu..................	2	1	seq
......................................................................ucacagugaaccggGcucuuuu..................	3	1	seq
......................................................................ucacagugaaccggucucuuGu..................	1	1	seq
......................................................................ucaAagugaaccggucucuuuu..................	1	1	seq
......................................................................ucacagugaaccggucucuuuG..................	11	1	seq
......................................................................ucacagugaaccggucuUuuuu..................	4	1	seq
......................................................................ucacagCgaaccggucucuuuu..................	2	1	seq
......................................................................ucacagugaaccggucucuuCu..................	10	1	seq
......................................................................ucacagugaaccggucucuuuA..................	142	1	seq
```

```
                                        Star                                    Mature
ggacuggccugaguuuauuuaugagcuguuggauucggggccguaacacugucugagagguuuacauuucucacagugaaccggucucuuuuucagcugcuuccuggcuuc
......................................................................ucacagugaaccggucuUuuuuu..................   1     1   seq
......................................................................ucacagugaaccggucucuuuGu..................   7     1   seq
......................................................................ucacagugaaccggucucuAuuu..................   1     1   seq
......................................................................ucacagugaaccggucucuuuAu..................   161   1   seq
......................................................................ucacagugaacUggucucuuuuu..................   1     1   seq
......................................................................ucacagugaaccggucucuuuuu..................   199   0   seq
......................................................................ucacagugaaccggucucuuAuu..................   1     1   seq
......................................................................ucacagugaaccggucucuuuuG..................   3     1   seq
......................................................................ucacagugaaccggucucuuuCu..................   8     1   seq
......................................................................ucacagugaaccggucucuuuuA..................   18    1   seq
......................................................................ucacagugaaccggucucuuuuuU.................   40    1   seq
......................................................................ucacagugaaccggucucuuuuuc.................   4     0   seq
.......................................................................cacagugaaccggucucuuu....................   1     0   seq
.......................................................................cacagugaaUcggucucuuuu...................   1     1   seq
.......................................................................cacagugaaccggucucuuuu...................   1     0   seq
.......................................................................cacagugaaccggucucuuuuuU.................   1     1   seq
........................................................................acagugaaccggucucuuuu...................   1     0   seq
.........................................................................cagugaaccggucucuuuAu..................   1     1   seq
```

Provisional ID	:	mun-novel75
Score total	:	0.2
Score for star read(s)	:	-1.3
Score for read counts	:	-1.3
Score for mfe	:	1.9
Score for randfold	:	1.6
Score for cons. seed	:	-0.6
Total read count	:	9
Mature read count	:	9
Loop read count	:	0
Star read count	:	0

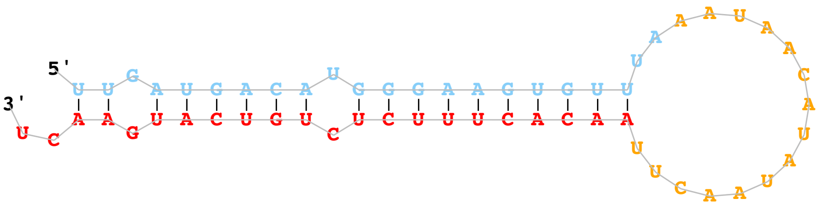

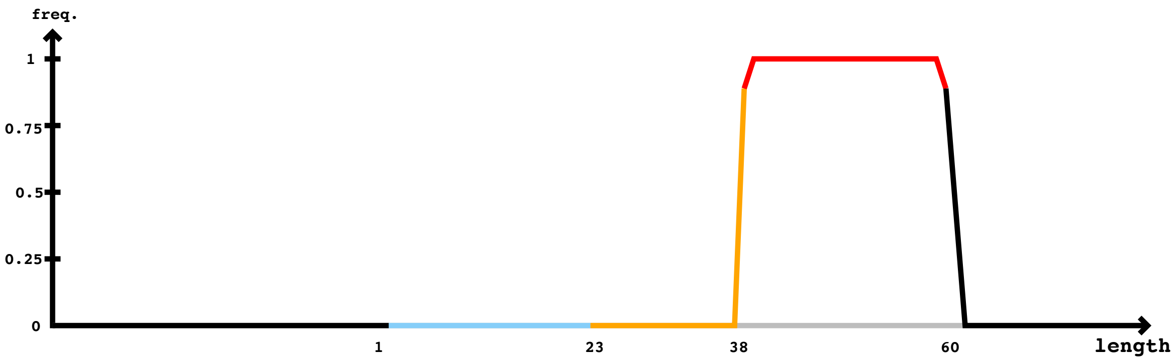

Star Mature

```
5'- cuacuauagcauaugguauguugagauagcagguugaugacaugggaaguguuuaaauaacauauaacuuaacacuuucucugucaugaacuuguuauuccaacacuucaga  -3'   exp
    ..............(((..(((((..(((((((((.((((((.((((((((((.................)))))))))).)))))).)))))))))..)))))..)))...   reads   mm   sample
    ......................................................................aacacuuucucugucaugaac.....................   1       0    seq
    ......................................................................aacacuuucucugucaugaacu....................   5       0    seq
    ......................................................................aacacuuucucugucaugaacuu...................   2       0    seq
    .......................................................................acacuuucucugucaugaacu....................   1       0    seq
```

Provisional ID	:	mun-novel54
Score total	:	4.1
Score for star read(s)	:	3.9
Score for read counts	:	-2.8
Score for mfe	:	2.1
Score for randfold	:	1.6
Score for cons. seed	:	-0.6
Total read count	:	6
Mature read count	:	4
Loop read count	:	0
Star read count	:	2

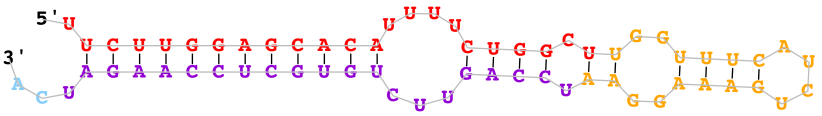

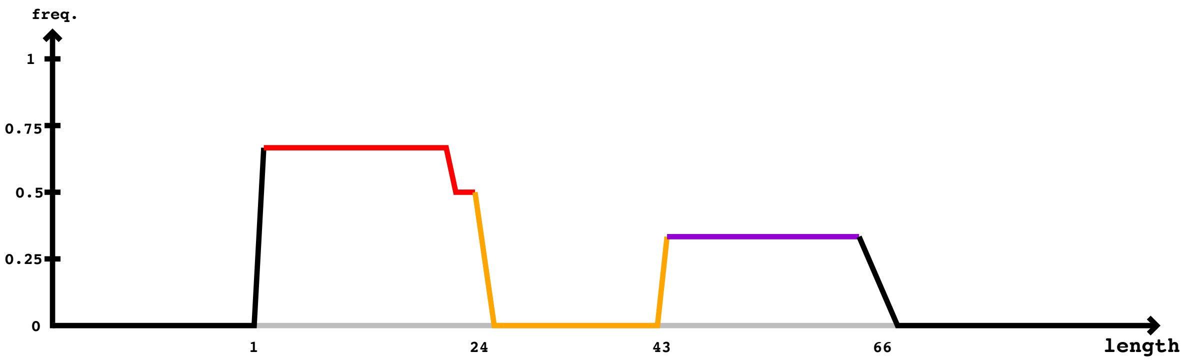


```
                    Mature                                        Star
5'- gaugcagcacauuagaugugouucuuggagcacauuuucuggcuugguuucaucugaaaggaauccaguucugugcuccaagaucacaacaugaugugaaccacugcaaaguaa   -3'    obs
    gaugcagcacauuagauguguucuuggagcacauuuucuggcuugguuucaucugaaaggaauccaguucugugcuccaagaucacaacaugaugugaaccacugcaaaguaa          exp
    ..((((((((((((..((((.((((((((((((....((((.((..((((....))))..)).))))...)))))))))))).))))...))))))).....)))))......   reads   mm   sample
    ....................uucuuggagcacauuuucug.........................................................................   1       0    seq
    ....................uucuuggagcacauuuucuggcu......................................................................   3       0    seq
    ..............................................................uccaguucugugcuccaagau..............................   2       0    seq
```

Provisional ID	:	mun-let-7i
Score total	:	4793
Score for star read(s)	:	3.9
Score for read counts	:	4782.3
Score for mfe	:	2.3
Score for randfold	:	1.6
Score for cons. seed	:	3
Total read count	:	9392
Mature read count	:	9366
Loop read count	:	1
Star read count	:	25

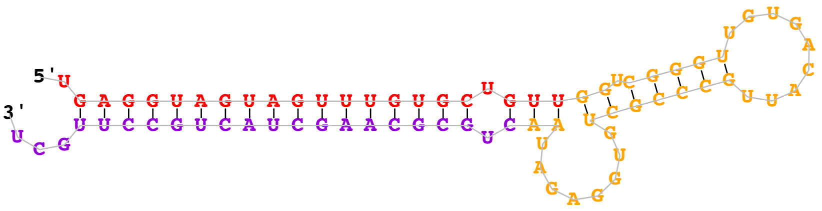

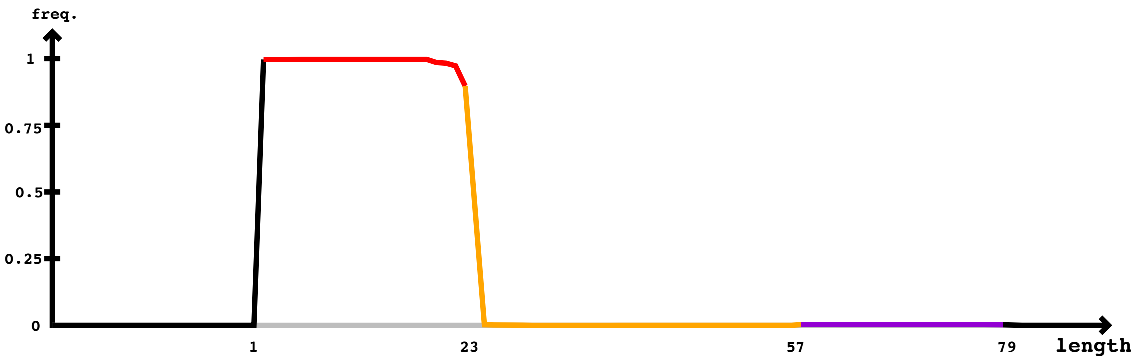

Mature Star

```
5'- gggugcaccagggcccuggcugagguaguaguuugugcuguuggucggguugugacauugcccgcuguggagauaacugcgcaagcuacugccuugcuagugcuggug  -3'   obs
    gggugcaccagggcccuggcugagguaguaguuugugcuguuggucggguugugacauugcccgcuguggagauaacugcgcaagcuacugccuugcuagugcuggug        exp
    .....((((((.((..((((.(((((((((((((((((.(((((.(((((.........)))))))........))).))))))))))))))))))))))).))))))  reads  mm  sample
    ....................ugaggGaguaguuugugc......................................................................  1      1   seq
    ....................ugagguaguaguuugugc......................................................................  108    0   seq
    ....................ugaggAaguaguuugugc......................................................................  1      1   seq
    ....................ugagguaguaguuuguAcu.....................................................................  1      1   seq
    ....................ugagguaguaguuugugcu.....................................................................  24     0   seq
    ....................ugagguaguaguuugugcug....................................................................  84     0   seq
    ....................Agagguaguaguuugugcug....................................................................  1      1   seq
    ....................ugagguaguaguuugugcuA....................................................................  3      1   seq
    ....................ugagguaguaguuugugcuU....................................................................  5      1   seq
    ....................ugagguaguaguuugugcGg....................................................................  1      1   seq
    ....................Ggagguaguaguuugugcugu...................................................................  1      1   seq
    ....................ugagguaguaguuuCugcugu...................................................................  1      1   seq
    ....................ugagguaguaguuugugcugu...................................................................  697    0   seq
    ....................ugagguaguaguuugugcuUu...................................................................  1      1   seq
    ....................ugagguaguaguuugCgcugu...................................................................  1      1   seq
    ....................ugagguaguaguuugugcugA...................................................................  3      1   seq
    ....................ugagguaguaguuugugcugC...................................................................  1      1   seq
    ....................ugagguaguagGuugugcugu...................................................................  1      1   seq
    ....................ugagguaguaguuugGgcugu...................................................................  1      1   seq
    ....................ugagguaguaguuuUugcugu...................................................................  1      1   seq
    ....................ugagguaguaguuuguUcugu...................................................................  1      1   seq
    ....................ugagguaguaguuugugcugG...................................................................  2      1   seq
    ....................ugagguaguagAuugugcuguu..................................................................  1      1   seq
    ....................ugagguaguaguuugugcuUuu..................................................................  1      1   seq
    ....................ugagguaguaguuuAugcuguu..................................................................  1      1   seq
    ....................ugagguaguaguuugugcuguG..................................................................  4      1   seq
    ....................ugagguaguagCuugugcuguu..................................................................  1      1   seq
    ....................ugaAguaguaguuugugcuguu..................................................................  1      1   seq
    ....................ugaUguaguaguuugugcuguu..................................................................  2      1   seq
    ....................ugagguaguaguuCgugcuguu..................................................................  1      1   seq
    ....................ugagguaguaguuugugcAguu..................................................................  2      1   seq
    ....................ugagguaguaguuuguCcuguu..................................................................  2      1   seq
    ....................uUagguaguaguuugugcuguu..................................................................  1      1   seq
```

Mature Star

```
gggugcaccagggcccuggcugagguaguaguuugugcuguuggucggguugugacauugcccgcuguggagauaacugcgcaagcuacugccuugcuagugcuggug
....................ugagguaguaguuugAgcuguu..................................................................  1     1  seq
....................Cgagguaguaguuugugcuguu..................................................................  2     1  seq
....................Ggagguaguaguuugugcuguu..................................................................  1     1  seq
....................ugaggCaguaguuugugcuguu..................................................................  2     1  seq
....................ugagguaguaguuuCugcuguu..................................................................  1     1  seq
....................ugGgguaguaguuugugcuguu..................................................................  2     1  seq
....................ugagguaguaguuugugcuguA..................................................................  8     1  seq
....................Agagguaguaguuugugcuguu..................................................................  2     1  seq
....................ugagguaguaguuuguUcuguu..................................................................  2     1  seq
....................ugagguaguaguAugugcuguu..................................................................  1     1  seq
....................ugaggAaguaguuugugcuguu..................................................................  9     1  seq
....................ugagguagGaguuugugcuguu..................................................................  1     1  seq
....................ugagguaguaguuugugcuguC..................................................................  1     1  seq
....................ugaggGaguaguuugugcuguu..................................................................  13    1  seq
....................ugagguaguaguuugugcuguu..................................................................  5618  0  seq
....................ugagguaguaguuuUugcuguu..................................................................  1     1  seq
....................ugagguaguaguuuguAcuguu..................................................................  3     1  seq
....................ugagguaguaUuuugugcuguu..................................................................  2     1  seq
....................ugagguaguaguuugugcuguug.................................................................  18    0  seq
....................ugagguaguaguuugugcuguuC.................................................................  14    1  seq
....................ugagguaguaguuugugcuguuA.................................................................  912   1  seq
....................ugagguaguaguuugugcuguAg.................................................................  1     1  seq
....................ugagguaguaguuugugcuguuU.................................................................  1777  1  seq
....................ugagguaguaguuugugcuguugU................................................................  8     1  seq
....................ugagguaguaguuugugcuguuggu...............................................................  2     0  seq
....................ugagguaguaguuugugcuguugguc..............................................................  2     0  seq
....................ugagguaguaguuugugcuguuggucg.............................................................  2     0  seq
....................ugagguaguaguuugugcuguuggucgg............................................................  5     0  seq
....................ugagguaguaguuugugcuguuggucgU............................................................  1     1  seq
......................agguaguaguuugugcuguuA.................................................................  1     1  seq
.......................gguaguaguuugugcuguu..................................................................  1     0  seq
........................guaguaguuugugcuguu..................................................................  1     0  seq
.........................................................uugcccgcuguggagauaacugc............................  1     0  seq
............................................................................cugcgcaagcuacugccuug............  2     0  seq
............................................................................cugcgcaagcuacugccuugc...........  3     0  seq
............................................................................cugcgcaagcuacugccuugcu..........  18    0  seq
............................................................................cugcgcaagcuacugccuugcC..........  1     1  seq
............................................................................cugcgcaagcuacugccuuAcu..........  1     1  seq
```

Provisional ID :mun-novel8
Score total : 0.3
Score for star read(s) : -1.3
Score for read counts : 0
Score for mfe : 0.8
Score for randfold : -2.2
Score for cons. seed : 3
Total read count : 92
Mature read count : 89
Loop read count : 3
Star read count : 0

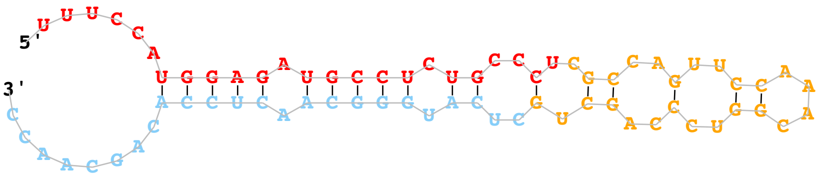

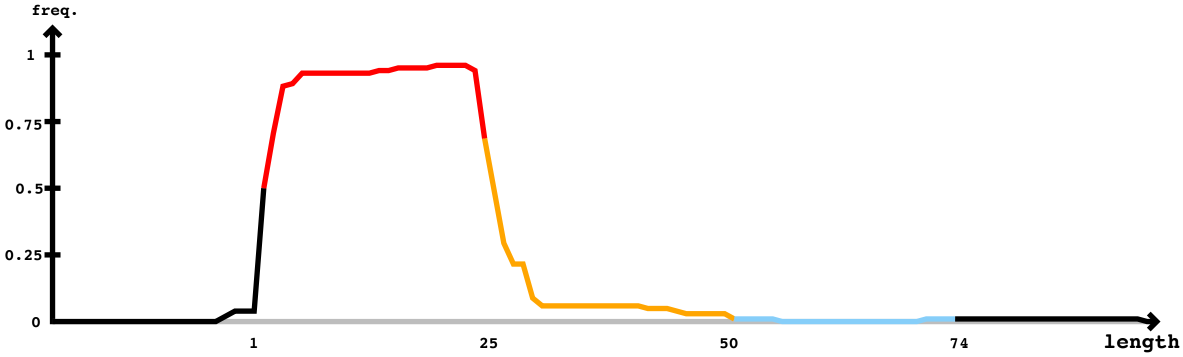

Mature Star

```
5'- acgcaacaaugcccccgugguuuccauggagaugccucugcccucgccaguuccaaacggucccagcugcucaugggcaacuccacagcaaccccugaagcuuugugauggug  -3'   exp
    .(((.((((.((..(.(.((((.(..(((((.(((((.((..(..((..(..((....))..)..)).)..)).))))).)))))..).)))).).)..)).))))....)))   reads   mm   sample
    ................guUguuuccauggagaugccucugccc......................................................................   1   1   seq
    ................gugguuuccauggagaugccucugcccu.....................................................................   1   0   seq
    .................ugguuuccauggagaugccucugcccu.....................................................................   1   0   seq
    .................uUguuuccauggagaugccucugcccu.....................................................................   1   1   seq
    ....................uuuccauggagaugccucugcc.......................................................................   1   0   seq
    ....................uuuccauggagaugccucugccc......................................................................   15   0   seq
    ....................uuuccauggagaugccucugcccu.....................................................................   20   0   seq
    ....................uuuccauggagaugccucugcccA.....................................................................   1   1   seq
    ....................uuuccauggagaugccucugcccuc....................................................................   1   0   seq
    ....................uuuccauggagaugccucugcccucU...................................................................   2   1   seq
    ....................uuuccauggagaugccucugcccucgcc.................................................................   3   0   seq
    ....................uuuccauggagaugccucugcccucgcca................................................................   1   0   seq
    ....................uuuccauggagaugccucugcccucgccU................................................................   1   1   seq
    ....................uuuccauggagaugccucugcccucgccag...............................................................   1   0   seq
    ....................uuuccauggagaugccuUugcccucgccagu..............................................................   1   1   seq
    .....................uuccauggagaugccucugccc......................................................................   5   0   seq
    .....................uuccauggagaugccucugcccu.....................................................................   12   0   seq
    .....................uuccauggagaugccucugcccucgcc.................................................................   4   0   seq
    ......................uccauggagaugccucugcc.......................................................................   1   0   seq
    ......................uccauggagaugccucugccc......................................................................   3   0   seq
    ......................uccauggagaugccucugcccu.....................................................................   2   0   seq
    ......................uccauggagaugccucugcccuc....................................................................   1   0   seq
    ......................uccauggagaugccucugcccucg...................................................................   4   0   seq
    ......................uccauggagaugccuUugcccucg...................................................................   1   1   seq
    ......................uccauggagaugccucugcccucgcc.................................................................   6   0   seq
    .......................ccauggagaugccucugcccucg...................................................................   1   0   seq
    ........................caCggagaugccucugccc......................................................................   1   1   seq
    ........................cauggagaugccucugccc......................................................................   2   0   seq
    ........................cauggagaugccucugcccucgccU................................................................   1   1   seq
    ................................ugccucugcccucgccaguuccaaacgg.....................................................   1   0   seq
    ..................................ccucugcccuUgccaguuccaaacggucc..................................................   1   1   seq
    ......................................ugcccucgccaguuccaaacgguccc.................................................   1   0   seq
    ...........................................ucgccaguuccaaacggucccagcug............................................   1   0   seq
    ..................................................uuccaaacggucccagcug............................................   1   0   seq
```

Mature Star

```
acgcaacaaugcccccgugguuuccauggagaugccucugcccucgccaguuccaaacggucccagcugcucaugggcaacuccacagcaaccccugaagcuuugugauggug
...................................................uccaaacgguUccagcugcucau.......................................	1	1	seq
.........................................................................................aaccccugaagcuuugugauggA.	1	1	seq
```

Provisional ID	:	mun-miR-429
Score total	:	967.3
Score for star read(s)	:	3.9
Score for read counts	:	957.1
Score for mfe	:	1.8
Score for randfold	:	1.6
Score for cons. seed	:	3
Total read count	:	1889
Mature read count	:	1884
Loop read count	:	0
Star read count	:	5

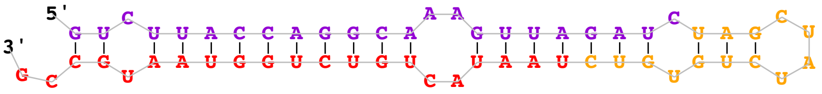

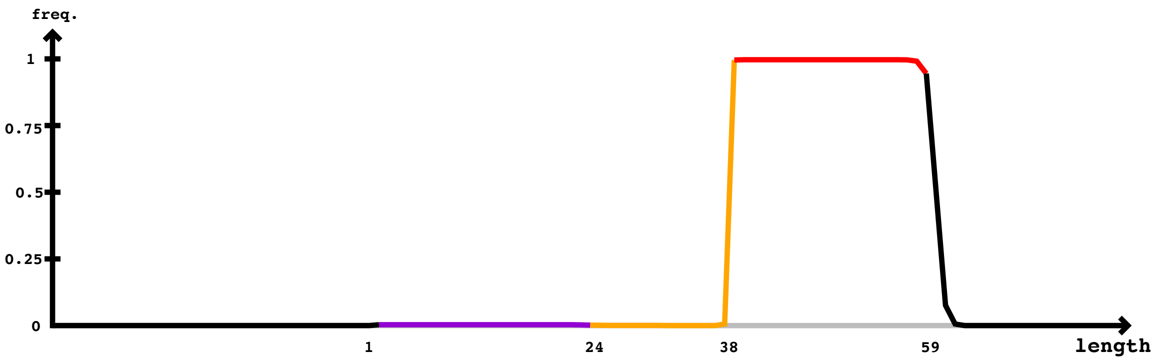

Star   Mature

```
5'- ccuuuagccugagugagugccugccgauugcugucuuaccaggcaaaguuagaucuagcuaucugugucuaauacugucugguaaugccgucaaucgcaucggcaaaagg  -3'    obs
    ccuuuagccugagugagugccugccgauugcugucuuaccaggcaaaguuagaucuagcuaucugugucuaauacugucugguaaugccgucaaucgcaucggcaaaagg         exp
    (((((.(((...((....)).(((.((((((.((.((((((((((..(((((((.(((....))).)))))))..)))))))))).)).).))))))))..))).)))))   reads   mm   sample
    ................................gucuuaccaggcaaaguuaga.........................................................   1       0    seq
    ................................gucuuaccaggcaaaguuagau........................................................   2       0    seq
    ................................gucuuaccaggcaaaguuagauG.......................................................   2       1    seq
    ................................gucuuaccaggcaaaguuagaucuagcuau................................................   1       0    seq
    ....................................................................cuaauacugucugguaaugcc.....................   1       0    seq
    ....................................................................cuaauacugucugguaaugccg....................   5       0    seq
    ....................................................................cuaauacugucugguaaugccA....................   2       1    seq
    ....................................................................cuaauacugucugguaaugccU....................   1       1    seq
    ....................................................................cuaauacugucugguaaugccgu...................   1       0    seq
    ....................................................................cuaauacugucugguaaugccguU..................   1       1    seq
    .....................................................................uaauacugucugguaaug.......................   1       0    seq
    .....................................................................uaauacugucugguaaugc......................   9       0    seq
    .....................................................................uaauacugucugguaaugcU.....................   5       1    seq
    .....................................................................uaauacugucugguaaugcc.....................   81      0    seq
    .....................................................................uaauacugucugguaaugcA.....................   1       1    seq
    .....................................................................Gaauacugucugguaaugccg....................   3       1    seq
    .....................................................................uaaCacugucugguaaugccg....................   1       1    seq
    .....................................................................uaauacugucCgguaaugccg....................   1       1    seq
    .....................................................................uaauacugucugguaaugccA....................   58      1    seq
    .....................................................................uaauacugucugguaaugccU....................   109     1    seq
    .....................................................................uaauacugucugguaaugccC....................   9       1    seq
    .....................................................................uaauacugucugguaaugccg....................   930     0    seq
    .....................................................................Aaauacugucugguaaugccg....................   1       1    seq
    .....................................................................Caauacugucugguaaugccg....................   2       1    seq
    .....................................................................uaauacugucuggGaaugccgu...................   1       1    seq
    .....................................................................uaauacugucugguaaugccUu...................   3       1    seq
    .....................................................................uaauacugucugguaaugUcgu...................   4       1    seq
    .....................................................................Gaauacugucugguaaugccgu...................   1       1    seq
    .....................................................................uaauacugucugguaaugccgA...................   10      1    seq
    .....................................................................uaauacugucugguaauUccgu...................   1       1    seq
    .....................................................................uaauacugucuAguaaugccgu...................   1       1    seq
    .....................................................................uaauacugucugguaaugccCu...................   1       1    seq
    .....................................................................uaauacugucuggAaaugccgu...................   1       1    seq
```

Star **Mature**

```
ccuuuagccugagugagugccugccgauugcugucuuaccaggcaaaguuagaucuagcuaucugugucuaauacugucugguaaugccgucaaucgcaucggcaaaagg
.....................................................................uaauacugucugguaaugccgC...................   1     1   seq
.....................................................................uaauacugucugguaaugccgu...................   495   0   seq
.....................................................................uaauacugucugguaaugccguA..................   9     1   seq
.....................................................................uaauacugucugguaaugccguG..................   1     1   seq
.....................................................................uaauacugucugguaaugccguU..................   113   1   seq
.....................................................................uaauacugucugguaaugccguc..................   7     0   seq
.....................................................................uaauacugucugguaaugccguUa.................   1     1   seq
.....................................................................uaauacugucugguaaugccguca.................   1     0   seq
.....................................................................uaauacugucugguaaugccguAa.................   2     1   seq
.....................................................................uaauacugucugguaaugccgucU.................   6     1   seq
.....................................................................uaauacugucugguaaugccguGa.................   1     1   seq
......................................................................aauacugucugguaaugccgu...................   1     0   seq
......................................................................aauacugucugguaaugccguU..................   1     1   seq
```

Provisional ID	:	mun-miR-1729
Score total	:	403.4
Score for star read(s)	:	3.9
Score for read counts	:	392.7
Score for mfe	:	2.2
Score for randfold	:	1.6
Score for cons. seed	:	3
Total read count	:	782
Mature read count	:	391
Loop read count	:	0
Star read count	:	391

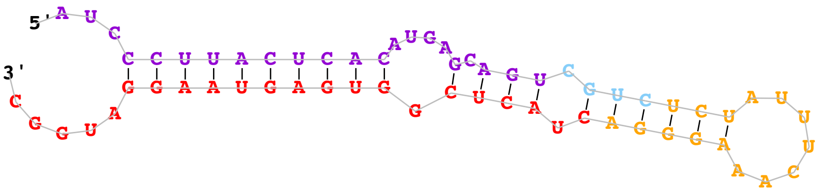

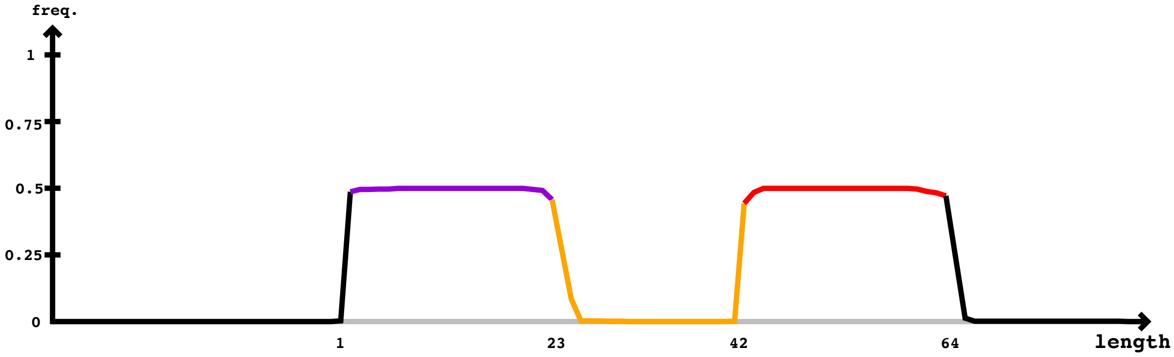


```
                                     Star                                     Mature
5'- uccucugugguagacaggccugauccugcaucccuuacucacaugagcagucgucucuauuucaaagggacuacucggugaguaaggauggcaggacugggaccacuaccua  -3'   obs
    uccucugugguagacaggccugauccugcaucccuuacucacaugagcagucgucucuauuucaaagggacuacucggugaguaaggauggcaggacugggaccacuaccua         exp
    ........(((((...(((((..((((((...((((((((((....(.(((.((((((.......)))))).)))).))))))))))...))))))..))).)).)))))..   reads   mm   sample
    ............................caucccuuacucacaugagcagu.............................................................   2       0    seq
    .............................aucccuuacucacaugagc................................................................   3       0    seq
    .............................aucccuuacucacaugagca...............................................................   3       0    seq
    .............................aucccuuacucacaugagcaA..............................................................   3       1    seq
    .............................aucccuuacucacaugagcag..............................................................   23      0    seq
    .............................Gucccuuacucacaugagcagu.............................................................   1       1    seq
    .............................aucAcuuacucacaugagcagu.............................................................   1       1    seq
    .............................aucccuuacucacaugagcagu.............................................................   168     0    seq
    .............................aucccuuacucacaugagcagG.............................................................   1       1    seq
    .............................aucccuuacucacaugagcaguc............................................................   112     0    seq
    .............................aucccuuacucacaugagcaguA............................................................   2       1    seq
    .............................aucccuuacucacaugagcagucA...........................................................   5       1    seq
    .............................aucccuuacucacaugagcagucg...........................................................   1       0    seq
    .............................aucccuuacucacaugagcagucC...........................................................   2       1    seq
    .............................aucccuuacucacaugagcagucU...........................................................   54      1    seq
    ..............................ucccuuacucacaugagcaguc............................................................   5       0    seq
    ..............................ucccuuacucacaugagcagucU...........................................................   2       1    seq
    ................................ccuuacucacaugagcaA..............................................................   1       1    seq
    ..................................uuacucacaugagcagucgucu........................................................   1       0    seq
    ..................................uuacucacaugagcagucgucucu......................................................   1       0    seq
    .....................................................................acuacucggugaguaaggauggcU...................   1       1    seq
    ......................................................................cuacucggugaguaagga........................   2       0    seq
    ......................................................................cuacucggugaguaaggau.......................   7       0    seq
    ......................................................................cuacucggugaguaaggaug......................   2       0    seq
    ......................................................................cuacucggugaguaaggauA......................   1       1    seq
    ......................................................................cuacucggugaguaaggaugg.....................   7       0    seq
    ......................................................................cuGcucggugaguaaggauggc....................   3       1    seq
    ......................................................................cuacucggugaguaaggauggc....................   186     0    seq
    ......................................................................cuacucggugaguaaggauggU....................   17      1    seq
    ......................................................................cuacucggugaguaaggauggA....................   16      1    seq
    ......................................................................cuacucggugaguaaggauggcG...................   1       1    seq
    ......................................................................cuacucggugaguaaggauggca...................   13      0    seq
    ......................................................................cuacucggugaguaaggauggUa...................   4       1    seq
```

Star Mature

uccucugugguagacaggccugauccugcaucccuuacucacaugagcagucgucucuauuucaaagggacuacucggugaguaaggauggcaggacugggaccacuaccua			
....cuacucggugaguaaggauggcU...................	79	1	seq
....cuacucggugaguaaggauggAa...................	2	1	seq
....cuacucggugaguaaggauggcaU.................	6	1	seq
....uacucggugaguaaggaug......................	1	0	seq
....uacucggugaguaaggaugg.....................	1	0	seq
....uacucggugaguaaggauggc....................	11	0	seq
....uacucggugaguaaggauggcU...................	3	1	seq
....uacucggugaguaaggauggcC...................	1	1	seq
....uacucggugaguaaggauggca...................	12	0	seq
....uacucggugaguaaggauggcaU..................	3	1	seq
....acucggugaguaaggaugg.....................	1	0	seq
....acucggugaguaaggauggA....................	1	1	seq
....acucggugaguaaggauggc....................	1	0	seq
....acucggugaguaaggauggca...................	7	0	seq
....acucggugaguaaggauggcU...................	2	1	seq
....aggacugggaccacuacc..	1	0	seq

Provisional ID	:	mun-novel87
Score total	:	1.8
Score for star read(s)	:	-1.3
Score for read counts	:	0
Score for mfe	:	2.1
Score for randfold	:	1.6
Score for cons. seed	:	-0.6
Total read count	:	48
Mature read count	:	48
Loop read count	:	0
Star read count	:	0

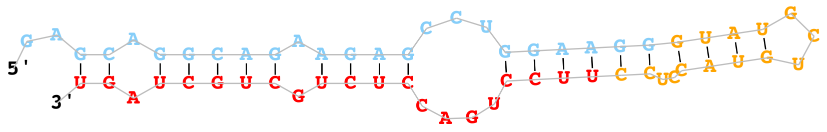

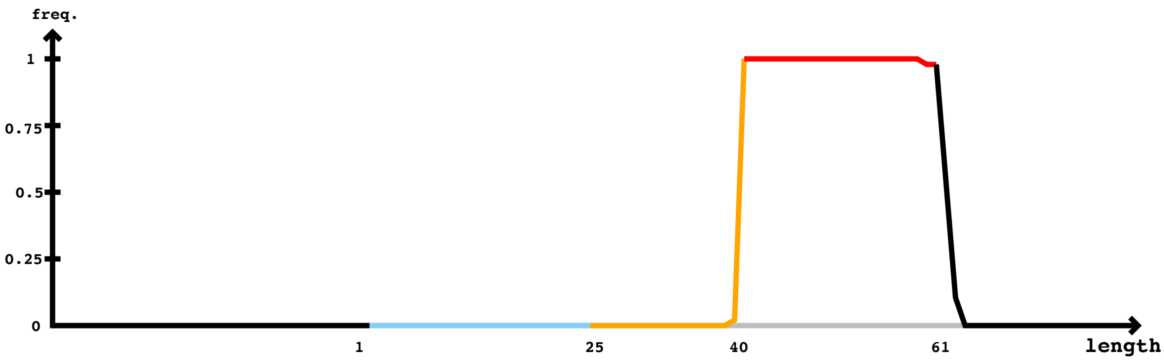

Star Mature

```
5'- aagguggaaguaucucucagugcuggcugaagagcaggcagaagagccuggaaggguaugcuguaccuccuuccugaccucugcugcuagucagcugccuggggaugaccu   -3'    exp
    .((((.....(((((.(((.((.((((((..(((((.((((.(..(..((((((((((...))))..)))))).)..))))))))))..))))))))))))))))))))    reads     mm      sample
    .....................................................................cuuccugaccucugcugcuagu....................    1         0       seq
    ......................................................................uuccugaccucugcugcua......................    1         0       seq
    ......................................................................uuccugaccucugcugcuagu....................    27        0       seq
    ......................................................................uuccugaccucugcugcuagA....................    1         1       seq
    ......................................................................uuccugaccucugcugcuaguc...................    11        0       seq
    ......................................................................uuccugaccucugcugcuaguU...................    2         1       seq
    ......................................................................uuccugaccucugcugcuagucU..................    5         1       seq
```

Provisional ID	:	mun-novel66
Score total	:	0.5
Score for star read(s)	:	-1.3
Score for read counts	:	0
Score for mfe	:	0.8
Score for randfold	:	1.6
Score for cons. seed	:	-0.6
Total read count	:	62
Mature read count	:	62
Loop read count	:	0
Star read count	:	0

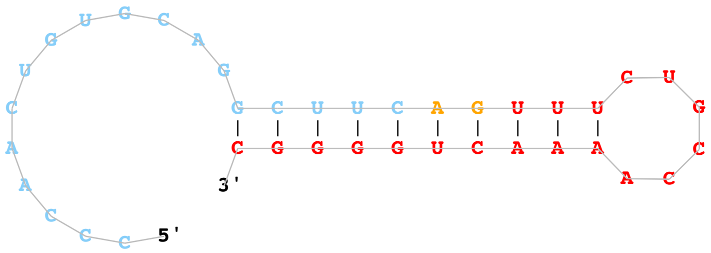

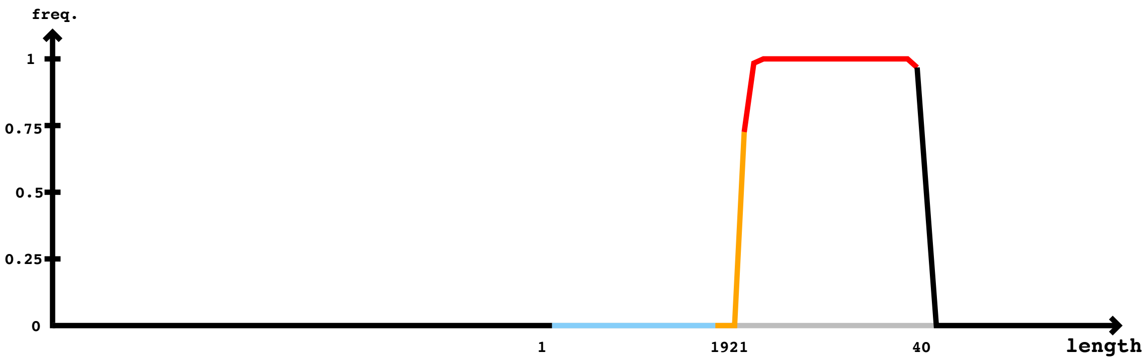

Star Mature

```
5'- ccacauagcugcucucuucagcaucuguccaggaugagcuuuucccucaccccaacugugcaggcuucaguuucugccaaaacuggggcucauggagaagucauaucag   -3'    exp
    ....((((.((((......)))).))))....((((.((((((((....(((((..((.(((((........)))))))....))))).....))))))))..))))..    reads    mm    sample
    .....................................................................uuucugccaaaaUugggg......................    2        1     seq
    .....................................................................uuucugccaaaaUuggggc.....................    38       1     seq
    .....................................................................uuucugccaaaacuggggc.....................    3        0     seq
    .....................................................................uuucugccaaaaUuggggcu....................    2        1     seq
    ......................................................................uucugccaaaaUuggggc.....................    14       1     seq
    ......................................................................uucugccaaaacuggggc.....................    2        0     seq
    .......................................................................ucugccaaaaUuggggcu....................    1        1     seq
```

Provisional ID	:	mun-novel88
Score total	:	0.2
Score for star read(s)	:	-1.3
Score for read counts	:	-1.3
Score for mfe	:	1.9
Score for randfold	:	1.6
Score for cons. seed	:	-0.6
Total read count	:	9
Mature read count	:	9
Loop read count	:	0
Star read count	:	0

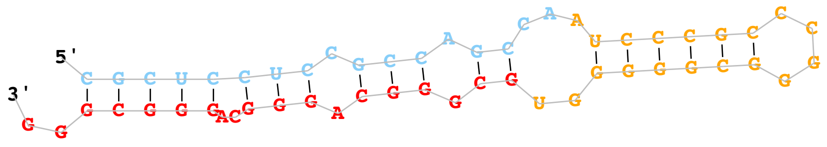

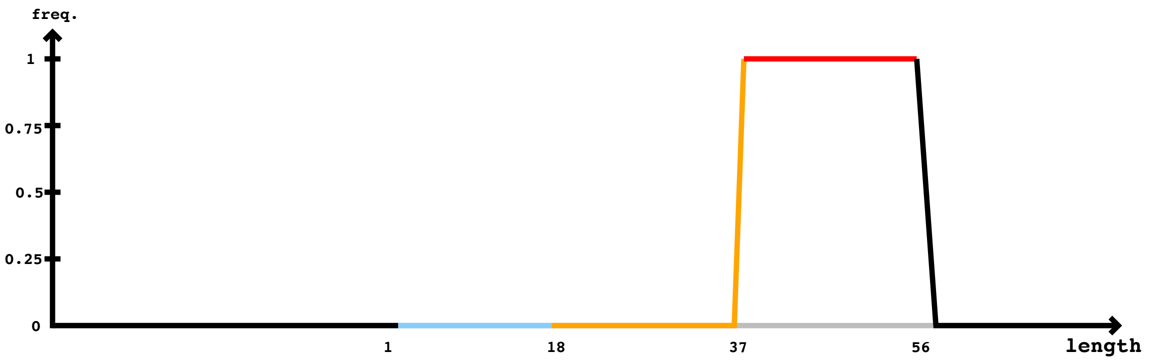

Star Mature

```
5'- nnnnnnnnnnnnnnnnnnnncgguccucacccaccgcuccuccgccagccaaucccgcccgggcgggggugcgggcagggcagggcgggacaggacaggacgagccuuc   -3'   exp
    .......................(((((..((..((((((((.(((.((...((((((....))))))..)).))).)))..))))))....))..))))).........  reads   mm   sample
    ......................................................................gcgggcagggcagggcggg....................  6       0    seq
    ......................................................................gcgggcagggcagggcggga...................  3       0    seq
```

Provisional ID	:	mun-miR-181b
Score total	:	4485.8
Score for star read(s)	:	3.9
Score for read counts	:	4475.9
Score for mfe	:	1.4
Score for randfold	:	1.6
Score for cons. seed	:	3
Total read count	:	8791
Mature read count	:	8774
Loop read count	:	0
Star read count	:	17

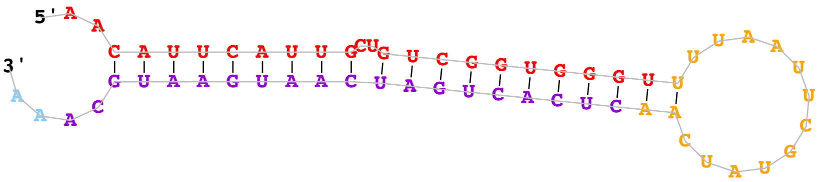

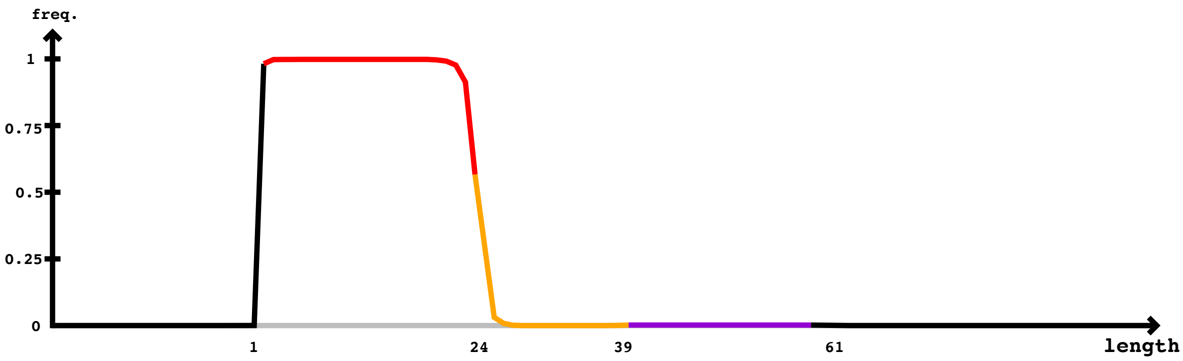

Mature Star

```
5'- ugcaucuaauggcugcaaucaacauucauugcugucggugggguuuuaauucguaucaacucacugaucaaugaaugcaaacugcggaccagcuaccagagagacugcgcacuc  -3'   obs
    ugcaucuaauggcugcaaucaacauucauugcugucggugggguuuuaauucguaucaacucacugaucaaugaaugcaaacugcggaccagcuaccagagagacugcgcacuc        exp
    .((((((..((((((((.....((((((((((..((((((((((............))))))))))))))))))))).....))))).)))..........))).)))......   reads   mm   sample
    .................aucaacauucauugcugucggugggu......................................................................   1       0    seq
    ..................caacauucauugcugucggugggu.......................................................................   1       0    seq
    ....................aacauucauugcugucgg...........................................................................   16      0    seq
    ....................aacauucaAugcugucggu..........................................................................   1       1    seq
    ....................aacauucauugcugucggu..........................................................................   39      0    seq
    ....................aacauucauugcugucggGg.........................................................................   1       1    seq
    ....................aacauucauugcuguUggug.........................................................................   1       1    seq
    ....................aacauucauugcugucgguU.........................................................................   2       1    seq
    ....................aacauucauugcugucgguA.........................................................................   8       1    seq
    ....................aacauucauugcugucggug.........................................................................   118     0    seq
    ....................Uacauucauugcugucggugg........................................................................   1       1    seq
    ....................aacauucauugcugucggGgg........................................................................   8       1    seq
    ....................aacauucauugcugucggugU........................................................................   65      1    seq
    ....................aacauucauugcugucggugg........................................................................   185     0    seq
    ....................aacauucauuCcugucggugg........................................................................   1       1    seq
    ....................aacauucauugcugucggugA........................................................................   298     1    seq
    ....................aacauucauugcugucggCgg........................................................................   1       1    seq
    ....................aacauucauugcugucggCggg.......................................................................   1       1    seq
    ....................aacauucauugcugucgguggU.......................................................................   37      1    seq
    ....................aacauucauugcugAcgguggg.......................................................................   1       1    seq
    ....................Gacauucauugcugucgguggg.......................................................................   1       1    seq
    ....................aacauucauugcugucUguggg.......................................................................   3       1    seq
    ....................aacauucauugcuguAgguggg.......................................................................   1       1    seq
    ....................aacauucauugcugucggugAg.......................................................................   2       1    seq
    ....................aacauucauugcugucggAggg.......................................................................   1       1    seq
    ....................aacauAcauugcugucgguggg.......................................................................   1       1    seq
    ....................aacauucauugGugucgguggg.......................................................................   1       1    seq
    ....................aacauucauugcugucAguggg.......................................................................   1       1    seq
    ....................aacauucauugcugucgguggA.......................................................................   780     1    seq
    ....................aacauucCuugcugucgguggg.......................................................................   1       1    seq
    ....................aGcauucauugcugucgguggg.......................................................................   1       1    seq
    ....................aacauucauugcCgucgguggg.......................................................................   1       1    seq
    ....................aacauucauugcAgucgguggg.......................................................................   1       1    seq
```

```
                    Mature                                 Star
ugcaucuaauggcugcaaucaacauucauugcugucggugggguuuaauucguaucaacucacugaucaaugaaugcaaacugcggaccagcuaccagagagacugcgcacuc
....................aacauucauugcuUucgguggg.......................................................................	1	1	seq
....................aacauucaAugcugucgguggg.......................................................................	1	1	seq
....................aacauucauugcugucgguggg.......................................................................	2179	0	seq
....................aacauucauCgcugucgguggg.......................................................................	1	1	seq
....................aacauucauugcugucgguUgg.......................................................................	1	1	seq
....................aacauucGuugcugucgguggg.......................................................................	1	1	seq
....................aacauucauugcugucggGggg.......................................................................	6	1	seq
....................aacauucauugcugucgguggC.......................................................................	1	1	seq
....................aacauAcauugcugucggugggu......................................................................	1	1	seq
....................aacauucauugcugucggugggC......................................................................	4	1	seq
....................aacauucauugcugucUgugggu......................................................................	1	1	seq
....................aacauucauugcugucggugggG......................................................................	104	1	seq
....................aacauucauugcugucggCgggu......................................................................	2	1	seq
....................aacauuAauugcugucggugggu......................................................................	1	1	seq
....................aacauucauugcugucgguggUu......................................................................	7	1	seq
....................aacauucauugUugucggugggu......................................................................	1	1	seq
....................aacauucauugcugucggAgggu......................................................................	2	1	seq
....................aacauucaCugcugucggugggu......................................................................	1	1	seq
....................aacauucauCgcugucggugggu......................................................................	1	1	seq
....................aacauucaAugcugucggugggu......................................................................	1	1	seq
....................aacauucauugcugGcggugggu......................................................................	2	1	seq
....................Gacauucauugcugucggugggu......................................................................	2	1	seq
....................aacauucauugcuguAggugggu......................................................................	1	1	seq
....................aacauucauugcuguUggugggu......................................................................	2	1	seq
....................aacauucauugcugucgguggCu......................................................................	3	1	seq
....................aacauuUauugcugucggugggu......................................................................	1	1	seq
....................aacauucauugcugucggugggu......................................................................	2613	0	seq
....................aacauucauugcugucggugggA......................................................................	417	1	seq
....................aaUauucauugcugucggugggu......................................................................	1	1	seq
....................aacauucUuugcugucggugggu......................................................................	1	1	seq
....................aacauucauugcugucggGgggu......................................................................	1	1	seq
....................aacauucauugcuUucggugggu......................................................................	1	1	seq
....................aacauucauugcugucggugUgu......................................................................	1	1	seq
....................aacauucauugcugucgguggAu......................................................................	40	1	seq
....................aacauucauugcuguAgguggguu.....................................................................	1	1	seq
....................aacauucauugcugucggugggGu.....................................................................	4	1	seq
....................aacauucauugcugucgguggguG.....................................................................	10	1	seq
....................aacauucauugcugucggugggAu.....................................................................	28	1	seq
....................aacauucauugcugucgguggCuu.....................................................................	1	1	seq
....................aacauucauugcugucggGggguu.....................................................................	2	1	seq
....................Gacauucauugcugucgguggguu.....................................................................	1	1	seq
....................aacauucaAugcugucgguggguu.....................................................................	1	1	seq
....................aacauucauugcugucggAggguu.....................................................................	1	1	seq
....................aacauGcauugcugucgguggguu.....................................................................	1	1	seq
....................aacauucauugcugucgguggguA.....................................................................	20	1	seq
....................aacauucauugcugucgguggguu.....................................................................	1327	0	seq
....................aacauucauugcugucgguggguCu....................................................................	1	1	seq
....................aacauucauugcugucggugggGuu....................................................................	1	1	seq
....................aacauucauugcugucggugggCuu....................................................................	1	1	seq
....................aacauucauugcugucggCggguuu....................................................................	1	1	seq
....................aacauucauugcugucgguggguuG....................................................................	2	1	seq
....................aacauucauugcugucgguggguAu....................................................................	11	1	seq
....................aacauucauuUcugucgguggguuu....................................................................	1	1	seq
....................aacauucauugcugucgguggguuC....................................................................	4	1	seq
....................aacauucauugcugucgguggguuA....................................................................	5	1	seq
....................aacauucauugcugucgguggguuu....................................................................	153	0	seq
....................aacauucauugcugucgguggguuCu...................................................................	6	1	seq
....................aacauucauugcugucgguggguuGu...................................................................	3	1	seq
....................aacauucauugcugucgguggguuuC...................................................................	1	1	seq
....................aacauucauugcugucggugggGuuu...................................................................	1	1	seq
....................aacauucauugcugucgguggguAuu...................................................................	1	1	seq
....................aacauucauugcugucgguggguuuu...................................................................	49	0	seq
....................aacauucauugcugucgguggguuuuU..................................................................	7	1	seq
....................aacauucauugcugucgguggguuuua..................................................................	1	0	seq
.....................acauucauugcugucggug.........................................................................	1	0	seq
.....................acauucauugcugucggugA........................................................................	1	1	seq
.....................acauucauugcugucggugg........................................................................	2	0	seq
.....................acauucauugcugucgguggA.......................................................................	6	1	seq
.....................acauucauugcugucgguggg.......................................................................	14	0	seq
.....................acauucauugcugucggGggg.......................................................................	1	1	seq
```

Mature Star

```
ugcaucuaauggcugcaaucaacauucauugcugucgguggguuuuaauucguaucaacucacugaucaaugaaugcaaacugcggaccagcuaccagagagacugcgcacuc
.....................acauucauugcugucggugggA......................................................................   2    1    seq
.....................acauucauugcugucggugggG......................................................................   3    1    seq
.....................acauucauugcugucggugggu......................................................................   35   0    seq
.....................acauucauugcugucgguggguu.....................................................................   52   0    seq
.....................acauucauugcugucgguggguA.....................................................................   1    1    seq
.....................acauucauugcugucgguggguCu....................................................................   1    1    seq
.....................acauucauugcugucgguggguuu....................................................................   18   0    seq
.....................acauucauugcugucgguggguuuuU..................................................................   1    1    seq
......................cauucauugcugucgguggguu.....................................................................   3    0    seq
......................cauucauugcugucgguggguuu....................................................................   2    0    seq
........................uucauugcugucgguggg.......................................................................   1    0    seq
........................uucauugcugucggugggu......................................................................   1    0    seq
.........................................................acucacugaucaaugaaugca...................................   5    0    seq
..........................................................cucacugaucaaugaaugc....................................   1    0    seq
..........................................................cucacugaucaaugaaugca...................................   7    0    seq
..........................................................cucacugaucaaugaaugcaU..................................   1    1    seq
..........................................................cucacugaucaaugaaugcaa..................................   3    0    seq
```

Provisional ID	:	mun-miR-138
Score total	:	461.4
Score for star read(s)	:	3.9
Score for read counts	:	450.8
Score for mfe	:	2.1
Score for randfold	:	1.6
Score for cons. seed	:	3
Total read count	:	896
Mature read count	:	889
Loop read count	:	0
Star read count	:	7

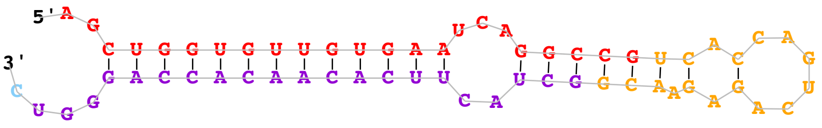

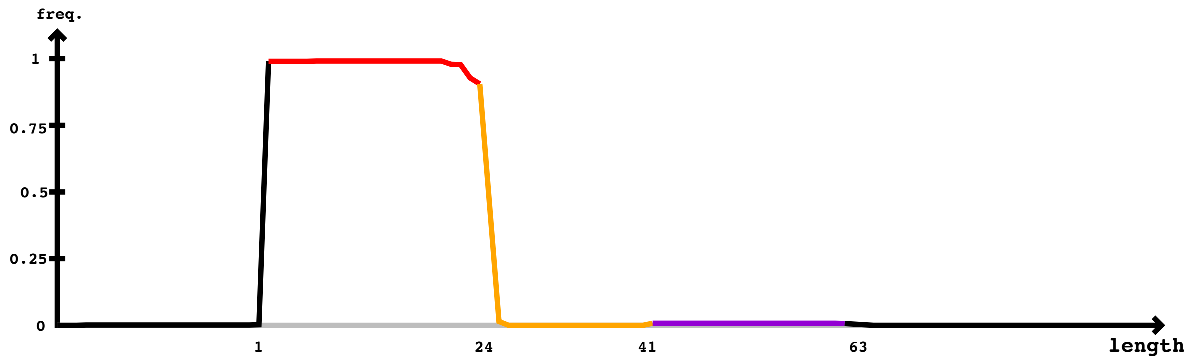

Mature **Star**

```
5'- ccccucaggugucgugcagcagcugguguugugaaucaggccgucaccagucagagaacggcuacuucacaacaccagggucgcaccgcaccacaguucaacugccagccaga   -3'   obs
    ccccucaggugucgugcagcagcugguguugugaaucaggccgucaccagucagagaacggcuacuucacaacaccagggucgcaccgcaccacaguucaacugccagccaga         exp
    .......(((((.((((.((..(((((((((((((...(((((((.(......).).))))))..))))))))))))).)).)))).))))).(((.....))).........    reads   mm   sample
    .cccucaggugucgugcagc.............................................................................................    1       0    seq
    ...................cagcugguguugugaaucaggcc.......................................................................    1       0    seq
    ....................agcugguguugugaaucag..........................................................................    10      0    seq
    ....................agcuggGguugugaaucag..........................................................................    1       1    seq
    ....................agcugguguugugaaucagg.........................................................................    1       0    seq
    ....................agcuggCguugugaaucaggc........................................................................    1       1    seq
    ....................agcugguguugugaaucaggc........................................................................    41      0    seq
    ....................agcugguguugugaaucaggU........................................................................    2       1    seq
    ....................agcugCuguugugaaucaggc........................................................................    1       1    seq
    ....................agcugguguugugaaucaggcU.......................................................................    2       1    seq
    ....................agcugguguugugaaucaggcc.......................................................................    17      0    seq
    ....................agcugguguugugaaucaggccg......................................................................    705     0    seq
    ....................agcugguguugugaaucaggccA......................................................................    27      1    seq
    ....................agcugguguugugaaucaUgccg......................................................................    1       1    seq
    ....................agcugguguugugaaucaggccU......................................................................    4       1    seq
    ....................agcugguguugugaaucaggccgu.....................................................................    57      0    seq
    ....................agcugguguugugaaucaggccgG.....................................................................    2       1    seq
    ....................agcugguguugugaaucaggccAu.....................................................................    1       1    seq
    ....................agcugguguugugaaucaggccUu.....................................................................    1       1    seq
    ....................agcugguguugugaaucaggccguA....................................................................    2       1    seq
    ....................agcugguguugugaaucaggccguU....................................................................    11      1    seq
    ........................guguugugaaucaggccg.......................................................................    1       0    seq
    ............................................................gcuacuucacaacaccaggg.................................    1       0    seq
    ............................................................gcuacuucacaacaccagggu................................    6       0    seq
```

Provisional ID	:	mun-novel35
Score total	:	1.3
Score for star read(s)	:	-1.3
Score for read counts	:	0
Score for mfe	:	1.6
Score for randfold	:	1.6
Score for cons. seed	:	-0.6
Total read count	:	18
Mature read count	:	18
Loop read count	:	0
Star read count	:	0

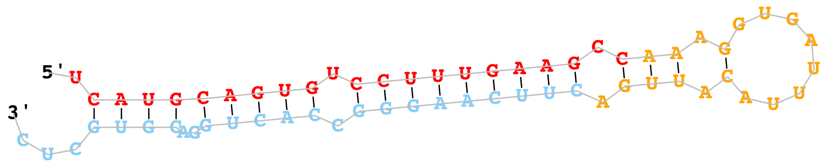

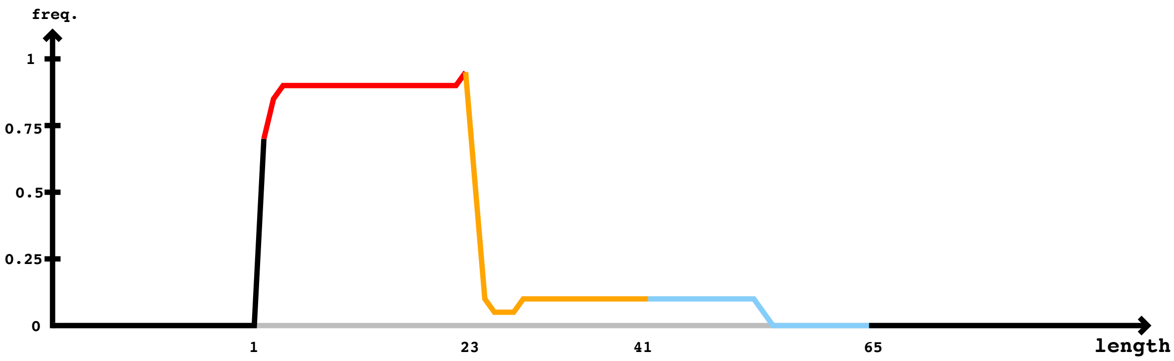

Mature **Star**

```
5'- uucuggagcuuguucaggccucaugcaguguccuuugaagccaaaggugauuuacauugacuucaagggccacuggacgugcuccugcaagaucaaguucugcugccguaac -3'   exp
    ....(((((((..((((...((((((((.((((((((.(((.(........).))).))))))))).)))))..))))..))))......)))))))..........   reads   mm   sample
    ....................ucaugcaguguccuuugaagcc......................................................................   13   0   seq
    ....................ucaugcaguguccuuugaagccU.....................................................................   1    1   seq
    .....................caugcaguguccuuugaagcc......................................................................   2    0   seq
    .....................caugcaguguccuuugaagccU.....................................................................   1    1   seq
    ......................augcaguguccuuugaagccaU....................................................................   1    1   seq
    .........................................caaaggugauuuacauugacuucaagggccac.......................................   1    0   seq
    ...............................................ugauuuacauugacuucaagggcca........................................   1    0   seq
```

Provisional ID	:	mun-miR-1587
Score total	:	5.6
Score for star read(s)	:	-1.3
Score for read counts	:	0
Score for mfe	:	2.3
Score for randfold	:	1.6
Score for cons. seed	:	3
Total read count	:	41
Mature read count	:	41
Loop read count	:	0
Star read count	:	0

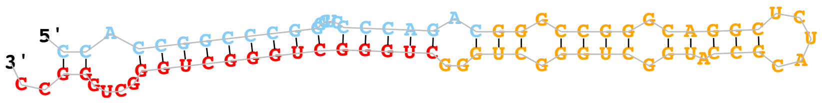

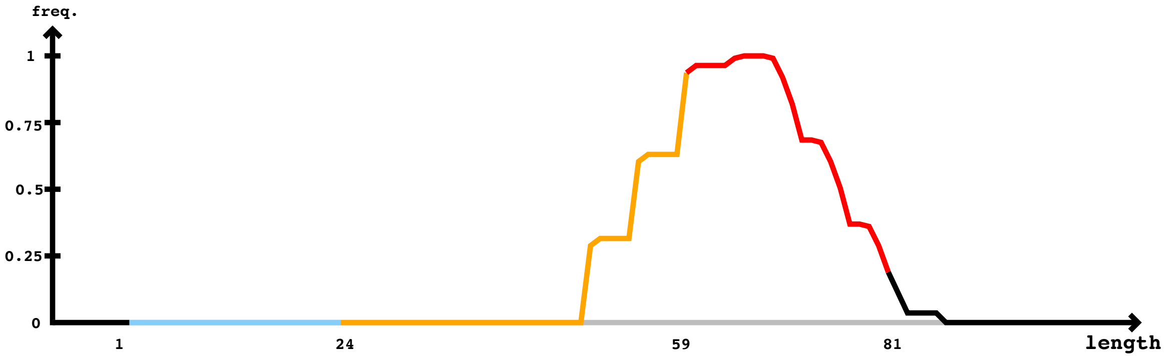

Star
Mature

```
5'- ccgucgccaccggcccgggguccccagacgggccgggcaggcucuacgccauggcugggcugggcugggcugggcugggcugggccgggccnnnnnnnnnnnnnnnnnnnn  -3'   exp
    (((..(((.(((((((((....(((((.(((.((((.(((((.....))).)).)))).)))..))))))))))))))....)))))).......................   reads   mm   sample
    ......................................................cugggcugggcugggcuggg.....................................   18      0    seq
    ......................................................Uugggcugggcugggcuggg.....................................   3       1    seq
    ......................................................cugggcugggcugggcugggc....................................   30      0    seq
    ......................................................cugggcugggcugggcugggcA...................................   45      1    seq
    .......................................................ugggcuAggcugggcugg......................................   3       1    seq
    .......................................................ugggcugggcugggcuggg.....................................   3       0    seq
    .......................................................ugggcugggcCgggcugggc....................................   3       1    seq
    ...........................................................cugggcugggcugggcuggg................................   18      0    seq
    ...........................................................Uugggcugggcugggcuggg................................   3       1    seq
    ...........................................................cugggcugggcugggcugggc...............................   30      0    seq
    ...........................................................cugggcugggcugggcugggcA..............................   45      1    seq
    ............................................................ugggcuAggcugggcugg.................................   3       1    seq
    ............................................................ugggcugggcugggcuggg................................   3       0    seq
    ............................................................ugggcugggcCgggcugggc...............................   3       1    seq
    ................................................................cugggcugggcugggcuggg...........................   18      0    seq
    ................................................................Uugggcugggcugggcuggg...........................   3       1    seq
    ................................................................cugggcugggcugggcugggc..........................   30      0    seq
    ................................................................cugggcugggcugggcugggcA.........................   45      1    seq
    ................................................................cugggcugggcugggcugggcc.........................   2       0    seq
    .................................................................ugggcuAggcugggcugg............................   3       1    seq
    .................................................................ugggcugggcugggcuggg...........................   3       0    seq
    .................................................................ugggcugggcCgggcugggc..........................   3       1    seq
    .....................................................................cugggcugggcAgggccgggcc....................   1       1    seq
    .....................................................................cugggcugggcugggccgggcA....................   2       1    seq
    ......................................................................ugggcugggcugggccgggcc....................   1       0    seq
```

Provisional ID	:	mun-miR-455
Score total	:	5.2
Score for star read(s)	:	-1.3
Score for read counts	:	0
Score for mfe	:	1.9
Score for randfold	:	1.6
Score for cons. seed	:	3
Total read count	:	6707
Mature read count	:	5126
Loop read count	:	1
Star read count	:	1580

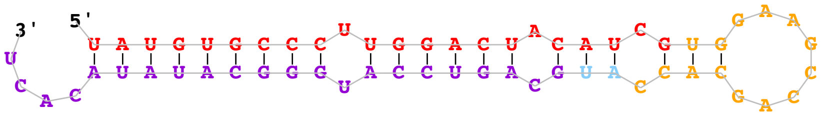

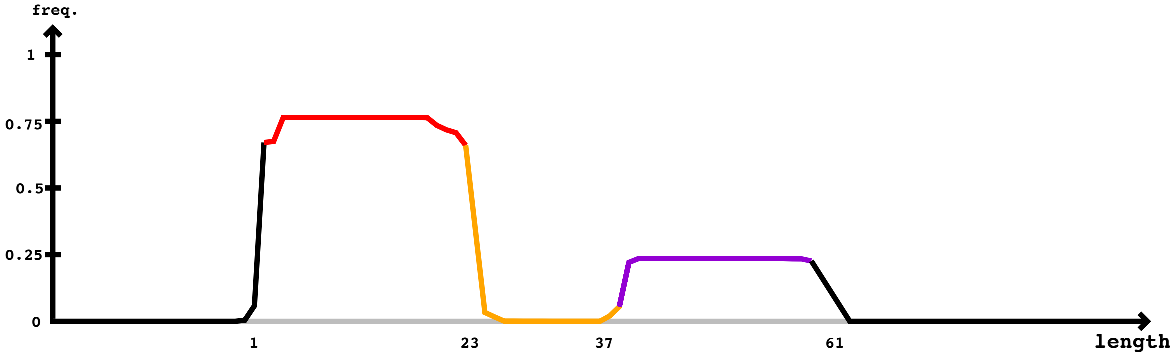

Mature **Star**

```
5'- caugaucccuggugugaggguaugugcccuuggacuacaucguggaagccagcaccaugcaguccaugggcauauacacuugccucaagguuuauuucaucacggcugccau  -3'   obs
    caugaucccuggugugaggguaugugcccuuggacuacaucguggaagccagcaccaugcaguccaugggcauauacacuugccucaagguuuauuucaucacggcugccau        exp
    .((((.((.(((.(..((.(((((((((.((((((.(((.(((........))).))).)))))).))))))))).))..).))).)).)))).................... reads  mm  sample
    ..................gguaugugcccuuggacua...........................................................................  3     0   seq
    ..................gguaugugcccuuggacuac..........................................................................  13    0   seq
    ..................gguaugugcccuuggacuGca.........................................................................  1     1   seq
    ..................gguaugugcccuuggacuaca.........................................................................  8     0   seq
    ..................gguaugugcccuuggacuacau........................................................................  4     0   seq
    ...................guaugugcccuuggacua...........................................................................  3     0   seq
    ...................guaugugcccuuggacuac..........................................................................  169   0   seq
    ...................guaugugcccuuggacuaU..........................................................................  4     1   seq
    ...................guaugugcccuuggacuaA..........................................................................  2     1   seq
    ...................guaugugcccuuggacuGc..........................................................................  3     1   seq
    ...................guaugugcccuuggacuGca.........................................................................  1     1   seq
    ...................guaugugcccuuggacuacU.........................................................................  11    1   seq
    ...................guaugugcccuuggacuaca.........................................................................  73    0   seq
    ...................guaugugcccuuggacuaAa.........................................................................  2     1   seq
    ...................guaugugcccuuggacuacau........................................................................  19    0   seq
    ...................guaugugcccuuggGcuacau........................................................................  1     1   seq
    ...................guGugugcccuuggacuacau........................................................................  1     1   seq
    ...................guaugugcccuuggacuGcau........................................................................  10    1   seq
    ...................guaugugcccuuggacuacauc.......................................................................  29    0   seq
    ...................guaugugcccuuggacuGcauc.......................................................................  2     1   seq
    ...................guaugugcccuuggacuacauU.......................................................................  2     1   seq
    ...................guaugugcccuuggacuacauA.......................................................................  7     1   seq
    ...................guaugugcccuuggacuacaucU......................................................................  5     1   seq
    ...................guaugugcccuuggacuacaucg......................................................................  14    0   seq
    ...................guaugugcccuuggacuacaucgu.....................................................................  1     0   seq
    ...................guaugugcccuuggacuacaucguggaagcc..............................................................  1     0   seq
    ...................guaugugcccuuggacuacaucguggaagccagcacc........................................................  2     0   seq
    ....................uaugugcccuuggacuac..........................................................................  3     0   seq
    ....................uaugugcccuuggacuaca.........................................................................  15    0   seq
    ....................uaugugcccuuggacuacau........................................................................  37    0   seq
    ....................uaugugcccuuggacuacaA........................................................................  3     1   seq
    ....................uaugugcccuuggacuGcauc.......................................................................  2     1   seq
    ....................uaugugcccuuggacuacauA.......................................................................  1     1   seq
```

Mature **Star**

```
caugaucccuggugugagggUAUGUGCCCUUGGACUACAUCGuggaagccagcaccauGCAGUCCAUGGGCAUAUACACUugccucaagguuuauuucaucacggcugccau
```

```
caugaucccuggugugaggguaugugcccuuggacuacaucguggaagccagcaccaugcaguccaugggcauauacacuugccucaagguuuauuucaucacggcugccau
....................uaugugcccuuggacuacauc.......................................................................  274   0  seq
....................uaugugcccuuggacuacauU.......................................................................  1     1  seq
....................uaugugcccuuggacuacauUg......................................................................  1     1  seq
....................uaugugcccuuggacuacaucU......................................................................  16    1  seq
....................uaugugcccAuggacuacaucg......................................................................  1     1  seq
....................uaugugcccuuggacuacauGg......................................................................  2     1  seq
....................uaugugcccuuggacuacUucg......................................................................  1     1  seq
....................uaugugGccuuggacuacaucg......................................................................  1     1  seq
....................uaugugcccuuggacuacaucg......................................................................  3242  0  seq
....................Aaugugcccuuggacuacaucg......................................................................  1     1  seq
....................uauAugcccuuggacuacaucg......................................................................  1     1  seq
....................uaugugcccuuggacuacaucA......................................................................  4     1  seq
....................uaCgugcccuuggacuacaucg......................................................................  1     1  seq
....................uaugugcccuuggacuacGucg......................................................................  1     1  seq
....................uauUugcccuuggacuacaucg......................................................................  1     1  seq
....................Gaugugcccuuggacuacaucg......................................................................  4     1  seq
....................uaugugcccuuggaUuacaucg......................................................................  1     1  seq
....................uaugGgcccuuggacuacaucg......................................................................  1     1  seq
....................uGugugcccuuggacuacaucg......................................................................  5     1  seq
....................uaugugcccuuUgacuacaucg......................................................................  1     1  seq
....................Caugugcccuuggacuacaucg......................................................................  1     1  seq
....................uaugugcccuuggacuaGaucg......................................................................  2     1  seq
....................uaugugcccuuggacuGcaucg......................................................................  18    1  seq
....................uaugugcccuuggacuacaucgG.....................................................................  2     1  seq
....................uaugugcccuuggacuacaucgA.....................................................................  29    1  seq
....................Gaugugcccuuggacuacaucgu.....................................................................  1     1  seq
....................uaugugcccuuggacuacaucgu.....................................................................  417   0  seq
....................uaugAgcccuuggacuacaucgu.....................................................................  1     1  seq
....................uaugugcccuugAacuacaucgu.....................................................................  1     1  seq
....................uaugugcccuuggGcuacaucgu.....................................................................  1     1  seq
....................uaugugcccuuggacuGcaucgu.....................................................................  2     1  seq
....................uaugugcccuuggacuacauAgu.....................................................................  1     1  seq
....................uaugugcccuuggacuacaucgug....................................................................  3     0  seq
....................uaugugcccuuggacuacaucgugU...................................................................  4     1  seq
....................uaugugcccuuggacuacaucgugg...................................................................  4     0  seq
.....................augugcccuuggacuacaucgu.....................................................................  28    0  seq
......................ugugcccuuggacuacaucg......................................................................  25    0  seq
......................ugugcccuuggacuacaucgA.....................................................................  1     1  seq
......................ugugcccuuggacCacaucgu.....................................................................  1     1  seq
......................ugugcccuuggacuacaucgu.....................................................................  367   0  seq
......................ugugcccuuggacuacaucguA....................................................................  1     1  seq
......................ugugcccuuggacuGcaucgug....................................................................  2     1  seq
......................ugugcccuuggacuacaucgug....................................................................  105   0  seq
......................ugugcccuuggacuacaucgugg...................................................................  10    0  seq
......................ugugcccuuggacuacaucgugC...................................................................  1     1  seq
......................ugugcccuuggacuacaucgugA...................................................................  42    1  seq
......................ugugcccuuggacuacaucgugU...................................................................  45    1  seq
......................ugugcccuuggacuacaucgugAaa.................................................................  2     1  seq
........................................cguggaagccagcaccauA.....................................................  1     1  seq
........................................................augcaguccaugggcaua......................................  1     0  seq
........................................................augcaguccaugggcauau.....................................  6     0  seq
........................................................augcaguccaugggcauauac...................................  21    0  seq
........................................................augcaguccaugggcauauacU..................................  17    1  seq
........................................................augcaguccaugggcauauaca..................................  32    0  seq
........................................................augcaguccaugggcauauacac.................................  35    0  seq
........................................................augcaguccaugggcauauacacC................................  3     1  seq
........................................................augcaguccaugggcauauacacu................................  19    0  seq
.........................................................ugcaguccaugggcauau.....................................  1     0  seq
.........................................................ugcaguccaugggcauaua....................................  1     0  seq
.........................................................ugcaguccaugggcauauU....................................  1     1  seq
.........................................................ugcaguccaugggcauaGac...................................  1     1  seq
.........................................................ugcaguccaugggcauauaA...................................  1     1  seq
.........................................................ugcaguccaugggcauauac...................................  27    0  seq
.........................................................ugcaguccaugggcauauaca..................................  38    0  seq
.........................................................ugcaguccaugggcauauacac.................................  106   0  seq
.........................................................ugcaguccaugggcauauacaA.................................  2     1  seq
.........................................................ugcaguccaugggcauauacaU.................................  2     1  seq
.........................................................Cgcaguccaugggcauauacac.................................  1     1  seq
.........................................................ugcGguccaugggcauauacac.................................  1     1  seq
.........................................................ugcaguccaugggcauauacacC................................  21    1  seq
```

```
                    Mature                                 Star
caugaucccuggugugagggauaugugcccuuggacuacaucguggaagccagcaccaugcaguccaugggcauauacacuugccucaagguuuauuucaucacggcugccau
.........................................................ugcaguccaugggcauauacacA................................	4	1	seq
.........................................................ugcaguUcaugggcauauacacu................................	1	1	seq
.........................................................Ggcaguccaugggcauauacacu................................	1	1	seq
.........................................................ugcaguccaugggcauauacacu................................	23	0	seq
.........................................................ugUaguccaugggcauauacacu................................	2	1	seq
..........................................................gcaguccaugggcauauac...................................	2	0	seq
..........................................................gcaguccaugggcauauaca..................................	28	0	seq
..........................................................gcaguccaugggcauauacU..................................	1	1	seq
..........................................................gUaguccaugggcauauaca..................................	1	1	seq
..........................................................gcaguccaugggcauauacac.................................	73	0	seq
..........................................................gcaguccaugggcauauacacA................................	8	1	seq
..........................................................gcaguccaugggcauauacacu................................	125	0	seq
..........................................................gcaguccaugggcauauacacC................................	858	1	seq
..........................................................gcaguccaugggcauauacaAu................................	1	1	seq
..........................................................gcaguccaugggcauauacacuu...............................	2	0	seq
..........................................................gcaguccaugggcauauacacCu...............................	14	1	seq
...........................................................caguccaugggcauauacU..................................	2	1	seq
...........................................................caguccaugggcauauaca..................................	1	0	seq
...........................................................caguccaugggcauauacaU.................................	1	1	seq
...........................................................caguccaugggcauauacac.................................	10	0	seq
...........................................................caguccaugggcauauacaA.................................	1	1	seq
...........................................................caguccauggUcauauacacu................................	1	1	seq
...........................................................caguccaugggcauauacacC................................	10	1	seq
...........................................................caguccaugggcauauacacu................................	13	0	seq
...........................................................Uaguccaugggcauauacacu................................	1	1	seq
...........................................................caguccaugggcauauacacCu...............................	48	1	seq
...........................................................caguccaugggcauauacacuu...............................	10	0	seq
............................................................aguccaugggcauauacU..................................	1	1	seq
```

Provisional ID	:	mun-novel93
Score total	:	43.2
Score for star read(s)	:	3.9
Score for read counts	:	36.8
Score for mfe	:	1.4
Score for randfold	:	1.6
Score for cons. seed	:	-0.6
Total read count	:	84
Mature read count	:	73
Loop read count	:	0
Star read count	:	11

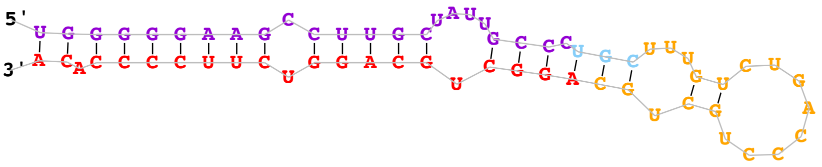

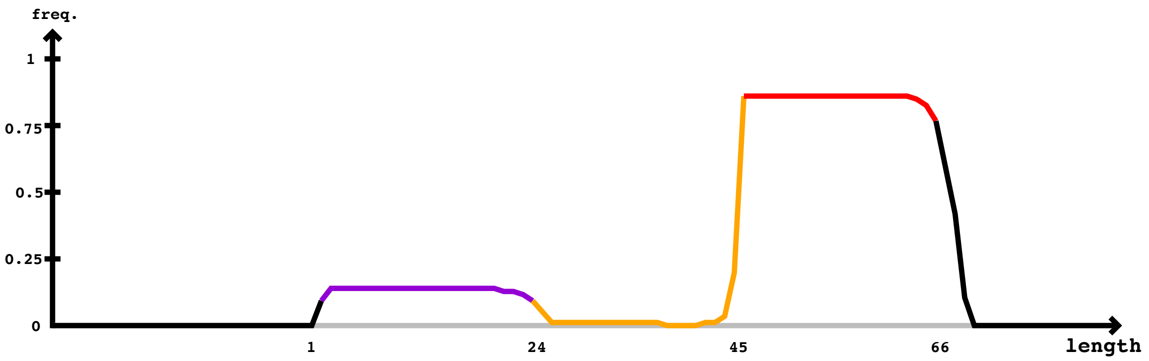


```
                            Star                                         Mature
5'- agagggagucagucugcuaaaauuaaaugggggaagccuugcuauugcccugcuuugucugacccugcugcaggcugcaggucuuccccacaccagcuuccccacccagc  -3'   obs
    agagggagucagucugcuaaaauuaaaugggggaagccuugcuauugcccugcuuugucugacccugcugcaggcugcaggucuuccccacaccagcuuccccacccagc        exp
    .(.((((((.((....)).........((((((((.(((((....(((.(((...((.........)).)))))).))))).))))))).))...)))))).).......   reads    mm    sample
    ...........................ugggggaagccuugcuauu................................................................   1        0     seq
    ...........................ugggggaagUcuugcuauugc..............................................................   1        1     seq
    ...........................ugggggaagccuugcuauugcc.............................................................   1        0     seq
    ...........................ugggggaagccuugcuauugccc............................................................   3        0     seq
    ...........................ugggggaagccuugcuauugccA............................................................   1        1     seq
    ...........................ugggggaagccuugcuauugcccu...........................................................   1        0     seq
    ............................gggggaagccuugcuauugcc.............................................................   1        0     seq
    ............................gggggaagUcuugcuauugccc............................................................   1        1     seq
    ............................gggggaagccuugcuauugccc............................................................   1        0     seq
    ............................gggggaagccuugcuauugcccugcuuugucugac...............................................   1        0     seq
    ...................................................................cugcaggcugcaggucuuccccacacca...............   1        0     seq
    .....................................................................gcaggcugcaggucuuccccacacc................   2        0     seq
    ......................................................................caggcugcaggucuuccccaU...................   1        1     seq
    ......................................................................caggcugcaggucuuccccacaA.................   1        1     seq
    ......................................................................caggcugcaggucuuccccacacc................   5        0     seq
    ......................................................................caggcugcaggucuuccccacacca...............   7        0     seq
    .......................................................................aggcugcaggucuucccU.....................   1        1     seq
    .......................................................................aggcugcaggucuucccca....................   2        0     seq
    .......................................................................aggcugcaggucuuccccaU...................   1        1     seq
    .......................................................................aggcugcaggucuuccccac...................   3        0     seq
    .......................................................................aggcugcaggucuuccccacU..................   10       1     seq
    .......................................................................aggcugcaggucuuccccaca..................   15       0     seq
    .......................................................................aggcugcaggucuuccccacC..................   3        1     seq
    .......................................................................aggcugcaggucuuccccacac.................   1        0     seq
    .......................................................................aggcugcaggucuuccccacacU................   6        1     seq
    .......................................................................aggcugcaggucuuccccacacc................   14       0     seq
    .......................................................................aggcugcaggucuuccccacaUca...............   1        1     seq
```

Provisional ID : mun-novel55
Score total : 1.5
Score for star read(s) : -1.3
Score for read counts : 0
Score for mfe : 1.8
Score for randfold : 1.6
Score for cons. seed : -0.6
Total read count : 33
Mature read count : 22
Loop read count : 11
Star read count : 0

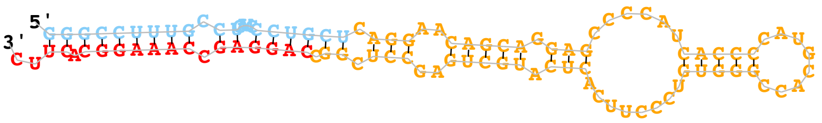

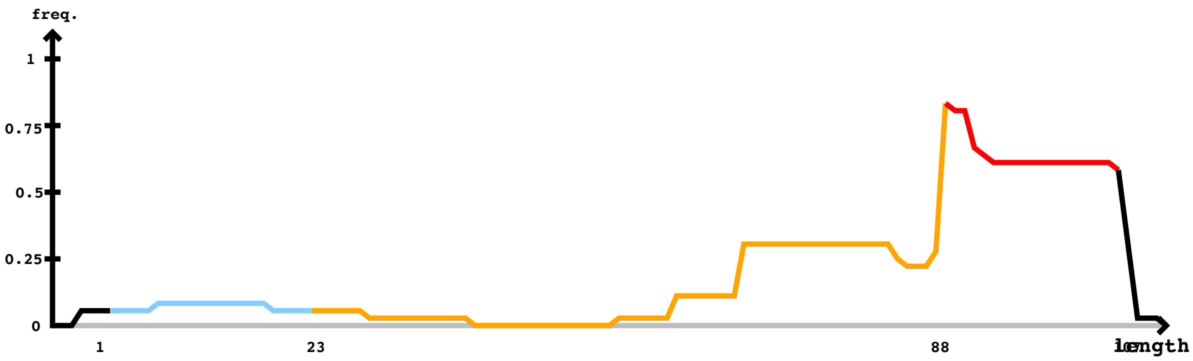

Star   Mature

```
5'- gcuggggccuuugccugaucccugcucaggaacagcaggagccccaucaccccaugcaccggguguccc uucacucaugcugagccucggccaggagccaaaggcacuucucac   -3'   exp
    (..((((((((((.((....((((((.(((..(((((.(((......(((((........)))))........))).)))))..))).)).)))))).))))))).)))..)..   reads   mm   sample
    .cuggggccuuugccugaCcc.............................................................................................   1   1   seq
    .cuggggccuuugccugaCcccugcucagga...................................................................................   1   1   seq
    .........uuugccugaCcccugcucaggaacagcaggagc.........................................................................   1   1   seq
    .........................................................accgggugucccuucacucaugcugagccu...........................   1   0   seq
    ...............................................................ugucccuucacucaugcugagcc............................   2   0   seq
    ...............................................................ugucccuucacucaugcugagccucggcc......................   1   0   seq
    ......................................................................ucacucaugcugagccucggccag....................   5   0   seq
    ......................................................................ucacucaugcugagccucggccagg...................   1   0   seq
    ......................................................................ucacucaugcugagccucggccagga..................   1   0   seq
    ..........................................................................................ccaggagccaaaggcacuu.....   1   0   seq
    ..........................................................................................ccaggagccaaaggcacuucucac   1   0   seq
    ...........................................................................................caggagccaaagAcacuuc....   17   1   seq
    ...........................................................................................caggagccaaaggcacuuc....   3   0   seq
```

Provisional ID	:	mun-novel22
Score total	:	34.8
Score for star read(s)	:	3.9
Score for read counts	:	27.7
Score for mfe	:	2.3
Score for randfold	:	1.6
Score for cons. seed	:	-0.6
Total read count	:	66
Mature read count	:	65
Loop read count	:	0
Star read count	:	1

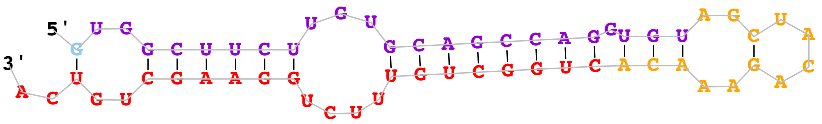

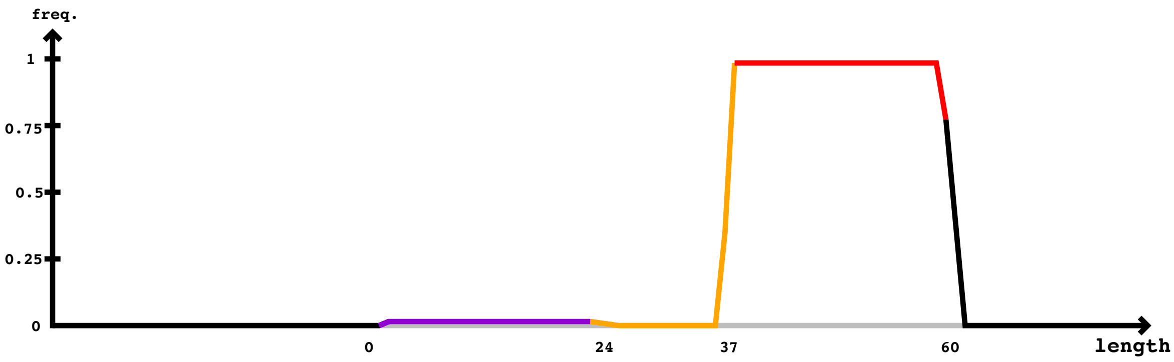

Star Mature

```
5'- ggggagagacaugggcaugcaggagggccgguguggcuucuugugcagccaggugu agcuacagaaacacuggcuguuucuggaagcugucauuagcccugccuucuccuga   -3'   obs
    ggggagagacaugggcaugcaggagggccgguguggcuucuugugcagccaggugu agcuacagaaacacuggcuguuucuggaagcugucauuagcccugccuucuccuga         exp
    .((((((..(((....))).((((((((..(((((((((((...((((((((.(((..(....)..)))))))))))....))))))))).)))..))))).))))))))..   reads   mm   sample
    .................................uggcuucuugugcagccaggugu........................................................   1       0    seq
    ....................................................................acuggcuguuucuggaagcuguc.....................   6       0    seq
    ....................................................................acuggcuguuucuggaagcuguca....................   3       0    seq
    ....................................................................acuggcuguuucuggaagcugucU....................   14      1    seq
    .....................................................................cuggcuguuucuggaagcuguc.....................   8       0    seq
    .....................................................................cuggcuguuucuggaagcugucU....................   23      1    seq
    .....................................................................cuggcuguuucuggaagcuguca....................   9       0    seq
    .....................................................................cuggcuguuucuggaagcugucau...................   2       0    seq
```

Provisional ID	:	mun-novel100
Score total	:	1.9
Score for star read(s)	:	-1.3
Score for read counts	:	0
Score for mfe	:	2.2
Score for randfold	:	1.6
Score for cons. seed	:	-0.6
Total read count	:	1770
Mature read count	:	1766
Loop read count	:	0
Star read count	:	4

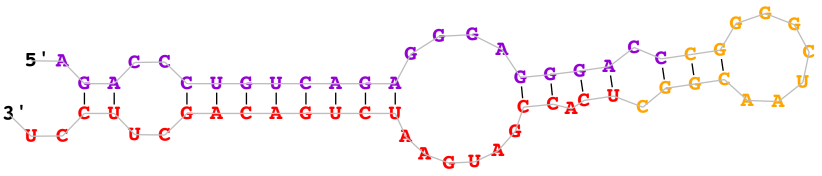

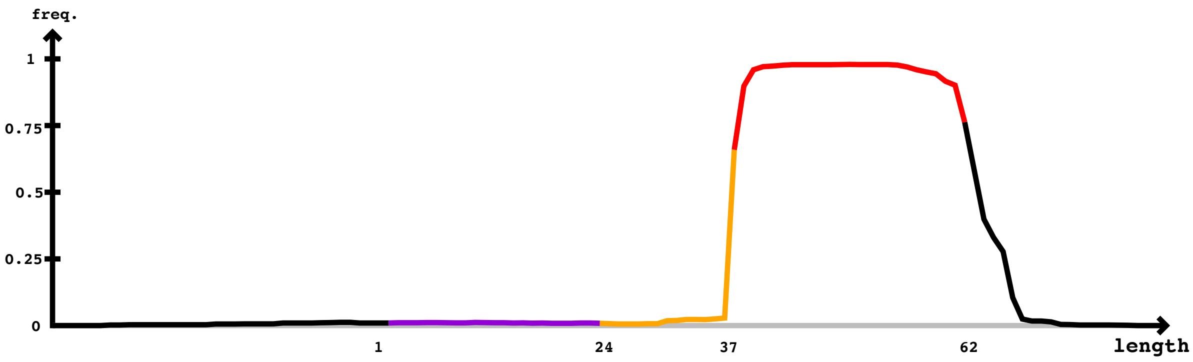

Star Mature

```
5'- gggaugcuccacagguccugagcugggaugcagagacccugucagagggagggaccggggcuaacggcucaccgaugaaucugacagcuuccugccucaggaucuccuguggg   -3'   obs
    gggaugcuccacagguccugagcugggaugcagagacccugucagagggagggaccggggcuaacggcucaccgaugaaucugacagcuuccugccucaggaucuccuguggg         exp
    ........((((((((((((((.......((((.((..((((((((....((((.(((.......))).)).))......))))))))..)))))))))))))...))))))).   reads   mm   sample
    ....ugcuccacagguccugagcugggaug....................................................................................   4   0   seq
    ......cuccacagguccugagcugggauA....................................................................................   1   1   seq
    ......cuccacagguccugagcugggaugcag.................................................................................   1   0   seq
    ...............uccugagcugggaugcagagaccc...........................................................................   2   0   seq
    ...............uccugagcugggaugcagagacccu..........................................................................   2   0   seq
    ...............uccugagcugggaugcagagacccug.........................................................................   1   0   seq
    ..................ugagcugggaugcagagacccuguc.......................................................................   1   0   seq
    ......................cugggaugcagagacccuguca......................................................................   1   0   seq
    ......................cugggaugcagagacccugucaga....................................................................   2   0   seq
    ......................cugggaugcagagacccugucagagU..................................................................   1   1   seq
    ......................cugggaugcagagacccugucagaggga................................................................   2   0   seq
    ..........................gaugcagagacccugucagagggagggCc...........................................................   1   1   seq
    ..........................gaugcagagacccugucagagggagggacc..........................................................   1   0   seq
    ...........................augcagagacccugucagagg..................................................................   1   0   seq
    ............................ugcagagacccugucagagggagggac...........................................................   1   0   seq
    ............................ugcagagacccugucagagggagggaccc.........................................................   1   0   seq
    .................................agacccugucagagggagggacc..........................................................   1   0   seq
    ..................................gacccugucagagggagggacU..........................................................   1   1   seq
    ..................................gacccugucagagggagggacccggggcuaacggcA............................................   1   1   seq
    .....................................ccugucagagggagggaccc.........................................................   1   0   seq
    .......................................ugucagagggagggacccgggg.....................................................   1   0   seq
    ........................................gucagagggagggacccggggcuaacggcu............................................   1   0   seq
    .........................................ucagagggagggacccggggcuaacggcuc...........................................   1   0   seq
    ..........................................cagagggagggacccggggcuaacU...............................................   1   1   seq
    ..........................................cagagggagggacccggggcuaacgg..............................................   1   0   seq
    ..........................................cagagggagggacccggggcuaacggc.............................................   1   0   seq
    ...............................................ggagggacccggggcuaac................................................   1   0   seq
    .................................................agggacccggggcuaacggcucaccga......................................   1   0   seq
    .....................................................acccggggcuaacggcuc...........................................   1   0   seq
    .....................................................acccUgggcuaacggcucaccga......................................   1   1   seq
    ............................................................gcuaacggcucaccgaugaaucugacagc.........................   2   0   seq
    .............................................................cuaacggcucaccgaugaaucugacagcuu.......................   1   0   seq
    ..............................................................uaacggcucaccgaugaaucugac............................   2   0   seq
```

Star **Mature**

```
gggaugcuccacagguccugagcugggaugcagagacccugucagagggagggacccggggcuaacggcucaccgaugaaucugacagcuuccugccucaggaucuccugugg g
..............................................................uaacggcucaccgaugaaucugaca...........................    5    0    seq
..............................................................uaacggcucaccgaugaaucugacag..........................    4    0    seq
..............................................................uaacggcucaccgaugaauAugacagc.........................    1    1    seq
..............................................................uaacggcucaccgaugaaucugacagc.........................    2    0    seq
..............................................................uaacggcCcaccgaugaaucugacagc.........................    1    1    seq
..............................................................uaacggcCcaccgaugaaucugacagcuu.......................    1    1    seq
..............................................................uaacggcucaccgaugaaucugacagcuu.......................    4    0    seq
..............................................................uaacggcucaccgaugaaucugacagcuucc.....................    1    0    seq
...............................................................aacggcucaccgaugaauc................................    1    0    seq
...............................................................aacggcucaccgaugaaucugacag..........................    1    0    seq
................................................................acggcucaccgaugaaucugac............................    1    0    seq
................................................................acggcucaccgaugaaucugaca...........................    2    0    seq
................................................................acggcucaccgaugaaucugacagc.........................    1    0    seq
................................................................acggcucaccgaugaaucugacagcuu.......................    1    0    seq
................................................................acggcucaccgaugaaucugacagcuucc.....................    2    0    seq
...................................................................gcucaccgaugaaucugac............................    1    0    seq
...................................................................gcucaccgaugaaucugacagcu........................    1    0    seq
...................................................................gcucaccgaugaaucugacagcuu.......................    3    0    seq
...................................................................Ucucaccgaugaaucugacagcuuccu....................    1    1    seq
....................................................................cucaccgaugaaucugaca...........................    1    0    seq
....................................................................cucaccgaugaaucugacagc.........................    1    0    seq
....................................................................cucaccgaugaaucugacagcuuc......................    2    0    seq
....................................................................cucaccgaugaaucugacagcuucc.....................    1    0    seq
....................................................................cucaccgaugaaucugacagcuuccu....................    2    0    seq
.....................................................................ucaccgaugaaucugaca...........................    6    0    seq
.....................................................................ucaccgaugaaucugacag..........................    11   0    seq
.....................................................................ucaccgaugaaucugacagc.........................    6    0    seq
.....................................................................ucaccgaugaaucugacagcu........................    8    0    seq
.....................................................................ucaccgaugaaucugacagcuu.......................    36   0    seq
.....................................................................ucaccgaugaaucugacagcuuc......................    25   0    seq
.....................................................................ucaccUaugaaucugacagcuucc.....................    1    1    seq
.....................................................................ucaccgaugaaucugacagcuucc.....................    215  0    seq
.....................................................................Ccaccgaugaaucugacagcuucc.....................    1    1    seq
.....................................................................ucaccgaugaaucugacagcuucU.....................    1    1    seq
.....................................................................ucaccgaugaaucugacagcuuccA....................    1    1    seq
.....................................................................ucaccgaugaaucugacaAcuuccu....................    1    1    seq
.....................................................................ucaccgaugaaucugacagcuuccC....................    1    1    seq
.....................................................................ucaccgaugaaucugacagcuuccG....................    1    1    seq
.....................................................................Ccaccgaugaaucugacagcuuccu....................    5    1    seq
.....................................................................ucaccgaugaaucugacagcuuccu....................    254  0    seq
.....................................................................ucaccgaugaaucugacagcuuccug...................    55   0    seq
.....................................................................Ccaccgaugaaucugacagcuuccug...................    3    1    seq
.....................................................................ucaccgaugaaucugacagcuuccuU...................    3    1    seq
.....................................................................ucaccgaugaaucugacagcuuccuA...................    2    1    seq
.....................................................................ucaccgaugaaucugacagcuuccugU..................    2    1    seq
.....................................................................ucaccgaugaaucugacagcuuccugc..................    84   0    seq
.....................................................................ucaccgaugaaucugacagcuuccugA..................    2    1    seq
.....................................................................Ccaccgaugaaucugacagcuuccugc..................    1    1    seq
.....................................................................ucaccgaugaaucugacagAuuccugcc.................    1    1    seq
.....................................................................Ccaccgaugaaucugacagcuuccugcc.................    1    1    seq
.....................................................................ucaccgaugaaucugacagcuuccugcc.................    82   0    seq
.....................................................................ucaccgaugaaucugacagcuucAugcc.................    1    1    seq
.....................................................................ucaccgaugaaucugacagcuuccugccu................    230  0    seq
.....................................................................ucaccgaugaaucugacagcuuccugccG................    1    1    seq
.....................................................................Ccaccgaugaaucugacagcuuccugccu................    2    1    seq
.....................................................................ucaccgaugaaucugacagcuuccugccA................    9    1    seq
.....................................................................ucaccgaugaaucugacagcuuccugccC................    3    1    seq
.....................................................................ucaccgaugaaucUacagcuuccugccu................    1    1    seq
.....................................................................Acaccgaugaaucugacagcuuccugccu................    1    1    seq
.....................................................................ucaccgaugaaucugacagcuucGugccu................    1    1    seq
.....................................................................ucaccgaugaaucugacagcuuccugccuA...............    4    1    seq
.....................................................................ucaccgaugaaucugacagcuuccugccuc...............    100  0    seq
.....................................................................Ccaccgaugaaucugacagcuuccugccuc...............    3    1    seq
.....................................................................ucaccgaugaaucugacagcuuccugccuU...............    3    1    seq
.....................................................................ucaccgaugaaucugacagcuuccugccuca..............    4    0    seq
.....................................................................ucaccgaugaaucugacagcuuccugccucC..............    1    1    seq
.....................................................................ucaccgaugaaucugacagcuuccugccucU..............    1    1    seq
.....................................................................ucaccgaugaaucugacagcuuccugccuAa..............    2    1    seq
.....................................................................ucaccgaugaaucugacagcuuccugccucagg............    3    0    seq
.....................................................................ucaccgaugaaucugacagcuuccugccucagga...........    8    0    seq
```

```
                                 Star                              Mature
gggaugcuccacagguccugagcugggaugcagagacccugucagagggagggacccggggcuaacggcucaccgaugaaucugacagcuuccugccucaggaucuccuguggg
.....................................................................ucaccgaugaaucugacagcuuccugccucaggauc.........	1	0	seq
.....................................................................ucaccgaugaaucugacagcuuccugccucaggaucuccu.....	1	0	seq
......................................................................caccgaugaaucugacaA..........................	1	1	seq
......................................................................caccgaugaaucugacag..........................	3	0	seq
......................................................................caccgaugaaucugacagc.........................	1	0	seq
......................................................................caccgaugaaucugacagcu........................	1	0	seq
......................................................................caccgaugaaucugacagcuu.......................	4	0	seq
......................................................................caccgaugaaucugacagcuuc......................	1	0	seq
......................................................................caccgaugaaucugacagcuucc.....................	36	0	seq
......................................................................caccgaugaaucugacagcuuccG....................	2	1	seq
......................................................................caccgaugaaucugacagcuuccC....................	1	1	seq
......................................................................caccgaugaaucugacagcuuccu....................	246	0	seq
......................................................................caccgaugaaucugacaCcuuccu....................	1	1	seq
......................................................................caccgaugaaucugacagcuuccuA...................	3	1	seq
......................................................................caccgaugaaucugacagcuuccuU...................	5	1	seq
......................................................................caccgaugaaucugacGgcuuccug...................	1	1	seq
......................................................................caccgaugaaucugacagcuuccug...................	12	0	seq
......................................................................Uaccgaugaaucugacagcuuccug...................	1	1	seq
......................................................................caccgaugaaucugacagcuuccugU..................	2	1	seq
......................................................................caccgaugaaucugacagcuuccugA..................	1	1	seq
......................................................................caccgaugaaucugacagcuuccugc..................	20	0	seq
......................................................................caccgaugaaucugacagcuuccugcc.................	11	0	seq
......................................................................caccgaugaaucugacagcuuccugccu................	48	0	seq
......................................................................caccgaugaaucugacagcuuccugccC................	1	1	seq
......................................................................caccgaugaaucugacagcuuccugccA................	3	1	seq
......................................................................caccgaugaaucugacagcuuccugccuU...............	1	1	seq
......................................................................caccgaugaaucugacagcuuccugccuc...............	32	0	seq
......................................................................caccgaugaaucugacagcuuccugccuca..............	2	0	seq
......................................................................caccgaugaaucugacagcuuccugccucagg............	2	0	seq
......................................................................caccgaugaaucugacagcuuccugccucagga...........	8	0	seq
......................................................................caccgaugaaucugacagcuuccugccucaggaA..........	1	1	seq
.......................................................................accgaugaaucugacagcu........................	2	0	seq
.......................................................................accgaugaaucugacagcuu.......................	3	0	seq
.......................................................................accgaugaaucugacagcuuc......................	1	0	seq
.......................................................................accgaugaaucugacagcuucc.....................	3	0	seq
.......................................................................accgaugaaucugaAagcuuccu....................	1	1	seq
.......................................................................accgaugaaucugacagcuuccu....................	69	0	seq
.......................................................................accgaugaaucugacagcuuccA....................	1	1	seq
.......................................................................accgaugaaucuUacagcuuccu....................	1	1	seq
.......................................................................accgaugaaucugacagcuuccug...................	6	0	seq
.......................................................................accgaugaaucugacagcuuccugc..................	13	0	seq
.......................................................................accgaugaaucugacagcuuccugcc.................	1	0	seq
.......................................................................accgaugaaucugacagcuuccugccu................	8	0	seq
.......................................................................accgaugaaucugacagcuuccugccuc...............	5	0	seq
.......................................................................accgaugaaucugacagcuuccugccuca..............	1	0	seq
.......................................................................accgaugaaucugacagcuuccugccucagg............	1	0	seq
.......................................................................accgaugaaucugacagcuuccugccucagga...........	1	0	seq
........................................................................ccgaugaaucugacagcu........................	1	0	seq
........................................................................ccgaugaaucugacagcuuccu....................	1	0	seq
........................................................................ccgaugaaucugacagcuuccugc..................	2	0	seq
........................................................................ccgaugaaucugacagcuuccugccA................	2	1	seq
........................................................................ccgaugaaucugacagcuuccugccu...............	12	0	seq
........................................................................ccgaugaaucugacagcuuccugccuc..............	1	0	seq
........................................................................ccgaugaaucugacagcuuccugccuca.............	2	0	seq
........................................................................ccgaugaaucugacagcuuccugccucagga..........	1	0	seq
.........................................................................cgaugaaucugacagcuuccug...................	1	0	seq
.........................................................................cgaugaaucugacagcuuccugc..................	2	0	seq
.........................................................................cgaugaaucugacagcuuccugcc.................	1	0	seq
..........................................................................gaugaaucugacagcuuccu....................	1	0	seq
..........................................................................gaugaaucugacagcuuccug...................	1	0	seq
..........................................................................gaugaaucugacagcuuccugcc.................	1	0	seq
..........................................................................gaugaaucugacagcuuccugccu................	2	0	seq
..........................................................................gaugaaucugacagcuuccugccuc...............	1	0	seq
...........................................................................augaaucugacagcuuccugcc.................	1	0	seq
...........................................................................augaaucugacagcuuccugccuc...............	1	0	seq
...........................................................................augaaucugacagcuuccugccucaggauc.........	1	0	seq
...........................................................................augaaucugacagcuuccugccucaggauU.........	1	1	seq
............................................................................ugaaucugacagcuuccug...................	1	0	seq
............................................................................ugaaucugacagcuuccugccu................	1	0	seq
................................................................................ucugacagcuuccugccucaggaucuccugu...	1	0	seq
```

```
                                           Star                                         Mature
gggaugcuccacagguccugagcugggaugcagagacccugucagagggagggacccggggcuaacggcucaccgaugaaucugacagcuuccugccucaggaucuccuguggg
.................................................................................cugacagcuuccugccuca..............    1    0    seq
.......................................................................................gcuuccugccucaggaucuccug....    1    0    seq
............................................................................................cugccucaggaucuccugu...    1    0    seq
```

Provisional ID	:	mun-miR-1784
Score total	:	3.3
Score for star read(s)	:	-1.3
Score for read counts	:	-1.8
Score for mfe	:	1.9
Score for randfold	:	1.6
Score for cons. seed	:	3
Total read count	:	8
Mature read count	:	8
Loop read count	:	0
Star read count	:	0

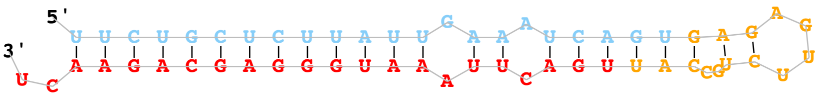

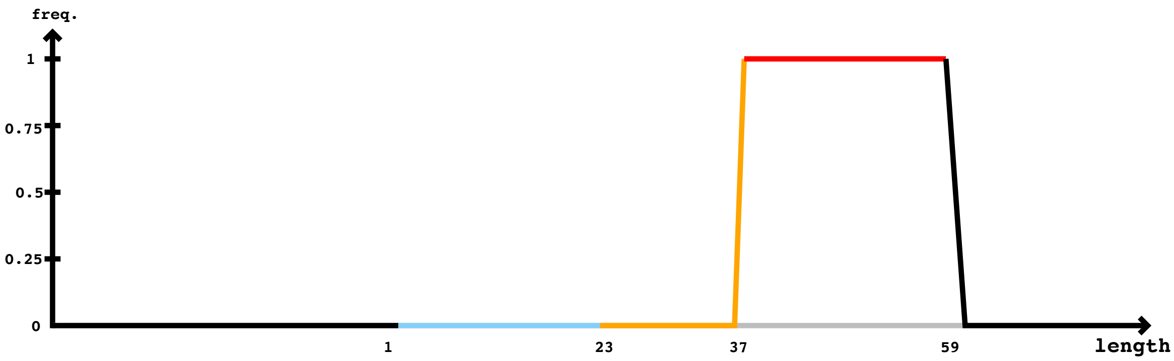

Star Mature

```
5'- aguuacuacugugggccaauuucuuggggcccaauucugcucuuauugaaaucagugagaguucugccauugacuuaaaugggagcagaacugggaccuaagaagacauuuc   -3'   exp
    ..((((....)))).....(((((((((.((((.(((((((((((((.((.((((((((....))..)))))).)).))))))))))))).)))).)))))))))).......   reads   mm   sample
    .....................................................................ugacuuaaaugggagcagaacu....................   8       0    seq
```

Provisional ID : mun-miR-16b
Score total : 3115.8
Score for star read(s) : 3.9
Score for read counts : 3106
Score for mfe : 1.3
Score for randfold : 1.6
Score for cons. seed : 3
Total read count : 6104
Mature read count : 6054
Loop read count : 0
Star read count : 50

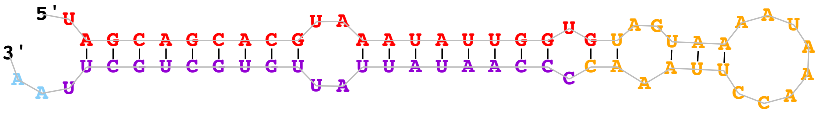

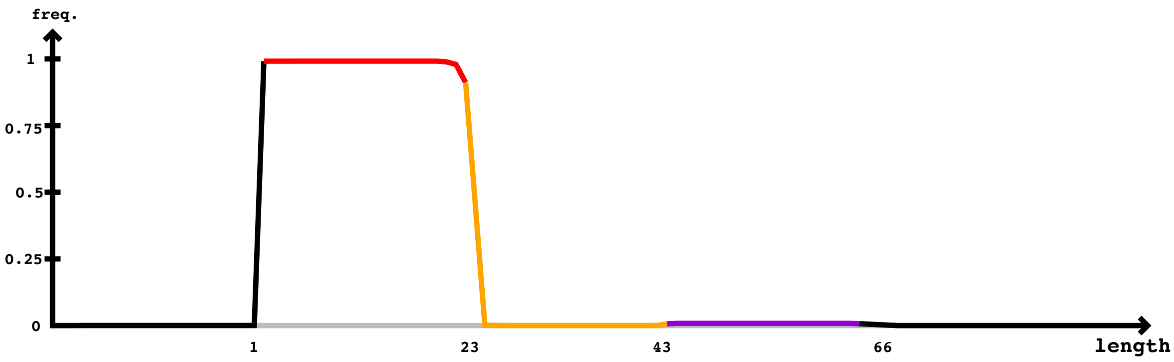


```
                                 Mature                                  Star
5'- cugucauacuuguuccgcccuagcagcacguaaauauugguguaguaaaauaaaccuuaaaccccaauauuauugugcugcuuaagcguggcagagguucagcaacuuguuu   -3'   obs
    cugucauacuuguuccgcccuagcagcacguaaauauugguguaguaaaauaaaccuuaaaccccaauauuauugugcugcuuaagcguggcagagguucagcaacuuguuu         exp
    .(((...((((((..(((...((((((((..((((((((.((..(((........))).)).))))))))..))))))))...)))..)))..)))...)))........   reads   mm   sample
    .ugucauacuuguuccgccc............................................................................................   1   0   seq
    ...................Guagcagcacguaaauauuggug......................................................................   1   1   seq
    ....................uagcagcacguaaauauu..........................................................................   1   0   seq
    ....................uagcagcacguaaauauug.........................................................................   15   0   seq
    ....................uagcagcacguaaauauuU.........................................................................   1   1   seq
    ....................uagcagcacguaaauauugU........................................................................   2   1   seq
    ....................uagcagcacguaaauauugg........................................................................   56   0   seq
    ....................uagcagcacgAaaauauugg........................................................................   1   1   seq
    ....................uagcagcacUuaaauauuggu.......................................................................   1   1   seq
    ....................uagcagcacguaaauauuggA.......................................................................   6   1   seq
    ....................uagcagcacguaaauauuggu.......................................................................   408   0   seq
    ....................uagcagcacAuaaauauuggu.......................................................................   1   1   seq
    ....................uagcagcacguaGauauuggug......................................................................   1   1   seq
    ....................uagcagcacguaaauUuuggug......................................................................   1   1   seq
    ....................uagcagcacguaaauauugguU......................................................................   12   1   seq
    ....................Gagcagcacguaaauauuggug......................................................................   2   1   seq
    ....................uagcagcacguaaauauugguC......................................................................   3   1   seq
    ....................uagcagcacguaaauauuggAg......................................................................   2   1   seq
    ....................uagcagcaUguaaauauuggug......................................................................   5   1   seq
    ....................Aagcagcacguaaauauuggug......................................................................   2   1   seq
    ....................uagcagcacgGaaauauuggug......................................................................   1   1   seq
    ....................uagcagcacguaaauaCuggug......................................................................   1   1   seq
    ....................uagcagcacCuaaauauuggug......................................................................   1   1   seq
    ....................uagcagcacguaaauauugUug......................................................................   1   1   seq
    ....................Cagcagcacguaaauauuggug......................................................................   1   1   seq
    ....................uagcagcacguaaauaGuggug......................................................................   1   1   seq
    ....................uagcagcacguaaauauuAgug......................................................................   2   1   seq
    ....................uagcagUacguaaauauuggug......................................................................   1   1   seq
    ....................uagcagcacguaaauauuggug......................................................................   4114   0   seq
    ....................uagcagcacguaaauauuggCg......................................................................   5   1   seq
    ....................uagcagcacguaaauauugCug......................................................................   1   1   seq
    ....................uaUcagcacguaaauauuggug......................................................................   1   1   seq
    ....................uagcagcacgCaaauauuggug......................................................................   2   1   seq
```

```
                    Mature                                        Star
cugucauacuuguuccgcccuagcagcacguaaauauugguguaguaaaauaaaccuuaaaccccaauauuauugugcugcuuaagcguggcagagguucagcaacuuguuu
....................uUgcagcacguaaauauuggug......................................................................    1     1    seq
....................uagcagcacguGaauauuggug......................................................................    1     1    seq
....................uagcagcGcguaaauauuggug......................................................................    1     1    seq
....................uagcagcacguaaauauuggGg......................................................................    4     1    seq
....................uagcagcacUuaaauauuggug......................................................................    1     1    seq
....................uagcagcacguaaauauugguA......................................................................    68    1    seq
....................uagcagcacguaaauauugguAu.....................................................................    8     1    seq
....................uagcagcacguaaauauuggugC.....................................................................    3     1    seq
....................uagcagcacguaaauauuCgugu.....................................................................    1     1    seq
....................uagcagcaAguaaauauuggugu.....................................................................    1     1    seq
....................uagcagcacguaaauauuggugG.....................................................................    74    1    seq
....................Cagcagcacguaaauauuggugu.....................................................................    1     1    seq
....................uagcagcacguaaauauuggugu.....................................................................    653   0    seq
....................uagcagcacguaaauauugguUu.....................................................................    1     1    seq
....................uagcagcacguaaauauuggugA.....................................................................    571   1    seq
....................uaUcagcacguaaauauuggugu.....................................................................    1     1    seq
....................uagcagcacguaaauauuggugua....................................................................    3     0    seq
....................uagcagcacguaaauauuggugAa....................................................................    4     1    seq
....................uagcagcacguaaauauuggugGa....................................................................    1     1    seq
....................uagcagcacguaaauauugguguagu..................................................................    1     0    seq
.....................agcagcacguaaauauuggugu.....................................................................    1     0    seq
........................agcacguaaauauuggug......................................................................    1     0    seq
.............................................................ccccaauauuauugugcugcuu.............................    1     0    seq
..............................................................cccaauauuauugugcugcu..............................    8     0    seq
..............................................................cccaauauuauugugcugcuu.............................    25    0    seq
..............................................................cccaauauuauugugcugcuua............................    1     0    seq
..............................................................cccaauauuauugugcugcuuU............................    4     1    seq
..............................................................cccaauauuauugugcugcuuaU...........................    1     1    seq
...............................................................ccaauauuauugugcugcuu.............................    5     0    seq
...............................................................ccaauauuauugugcugcuuU............................    1     1    seq
...............................................................ccaauauuauugugcugcuua............................    3     0    seq
...............................................................ccaauauuauugugcugcuuaU...........................    1     1    seq
```

Provisional ID	:	mun-miR-30c
Score total	:	12620.6
Score for star read(s)	:	3.9
Score for read counts	:	12610.1
Score for mfe	:	1.9
Score for randfold	:	1.6
Score for cons. seed	:	3
Total read count	:	24746
Mature read count	:	24323
Loop read count	:	0
Star read count	:	423

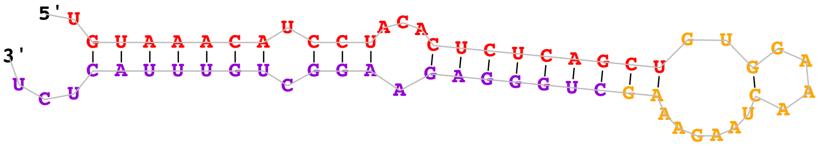

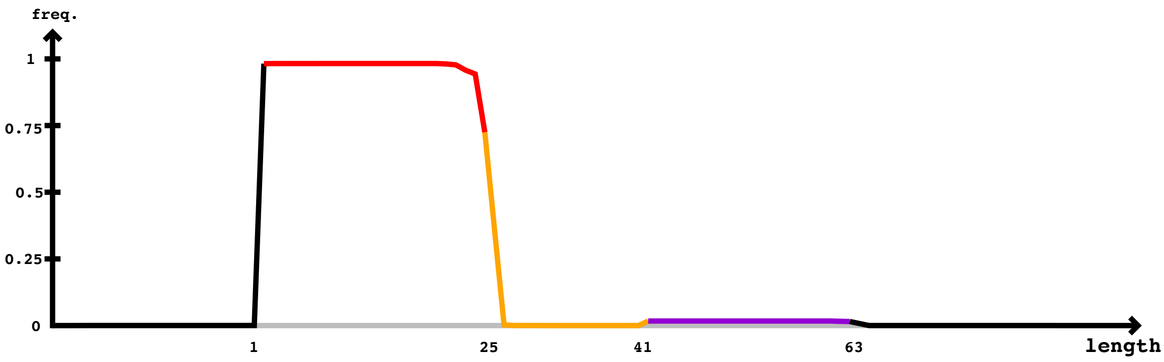

Mature Star

```
5'- gagcucugagugacagguaguguaaacauccuacacucucagcuguggaaacuaagaaagcugggagaaggcuguuuacucucccugccuuggaaaucaucuggagaacaa -3'  obs
    gagcucugagugacagguaguguaaacauccuacacucucagcuguggaaacuaagaaagcugggagaaggcuguuuacucucccugccuuggaaaucaucuggagaacaa       exp
    ((..((..((.(.((((.((.(((((((.(((...((((((((..(....)......)))))))).))).))))))).)).)))))))..))..))...............  reads  mm  sample
    .agcucugagugacagguag...........................................................................................  3  0  seq
    ..gcucugagugacagguag...........................................................................................  2  0  seq
    ..................aAuguaaacauccuacacucucagc....................................................................  1  1  seq
    ....................uguaaacauccuacacuc.........................................................................  1  0  seq
    ....................uguaaacauccuacacucu........................................................................  40  0  seq
    ....................Aguaaacauccuacacucu........................................................................  1  1  seq
    ....................uguaaacauccuacacucuc.......................................................................  85  0  seq
    ....................uguaaacauccuacacucuca......................................................................  478  0  seq
    ....................uguaaacauccuacacucucU......................................................................  12  1  seq
    ....................Gguaaacauccuacacucuca......................................................................  1  1  seq
    ....................uguaaacauccuacacucucaA.....................................................................  11  1  seq
    ....................uguaaacauccuacacucucaU.....................................................................  8  1  seq
    ....................uguaaacauccuacacucucag.....................................................................  330  0  seq
    ....................uguaaacauccuacGcucucag.....................................................................  1  1  seq
    ....................uguaaacauUcuacacucucag.....................................................................  1  1  seq
    ....................uguaaacauccuacacucCcag.....................................................................  1  1  seq
    ....................uUuaaacauccuacacucucagc....................................................................  1  1  seq
    ....................uguaaacauAcuacacucucagc....................................................................  2  1  seq
    ....................uguaaacauccuacacucuUagc....................................................................  1  1  seq
    ....................uguaaaAauccuacacucucagc....................................................................  2  1  seq
    ....................uguaaacauccuacacucucagA....................................................................  37  1  seq
    ....................Gguaaacauccuacacucucagc....................................................................  3  1  seq
    ....................uAuaaacauccuacacucucagc....................................................................  2  1  seq
    ....................uguaaacUuccuacacucucagc....................................................................  1  1  seq
    ....................ugGaaacauccuacacucucagc....................................................................  2  1  seq
    ....................uguaaacauUcuacacucucagc....................................................................  3  1  seq
    ....................uguaaacGuccuacacucucagc....................................................................  3  1  seq
    ....................uguaaacauccuacacuGucagc....................................................................  1  1  seq
    ....................Aguaaacauccuacacucucagc....................................................................  3  1  seq
    ....................uguaaacauccuacacucucUgc....................................................................  1  1  seq
    ....................ugCaaacauccuacacucucagc....................................................................  2  1  seq
    ....................uguaaacauccuacacAcucagc....................................................................  2  1  seq
    ....................uguaaacauccuaUacucucagc....................................................................  4  1  seq
```

```
                    Mature                                  Star
gagcucugagugacagguaguguaaacauccuacacucucagcuguggaaacuaagaaagcugggagaaggcuguuuacucucccugccuuggaaaucaucuggagaacaa
....................uguaaacauccuacacucucagc....................................................................   5155    0   seq
....................uguaaacauccuaGacucucagc....................................................................   1       1   seq
....................uguaaacauccuaAacucucagc....................................................................   1       1   seq
....................ugAaaacauccuacacucucagc....................................................................   2       1   seq
....................uguaaacauccuacacuUucagc....................................................................   3       1   seq
....................uguaaacauccuacacucucagU....................................................................   182     1   seq
....................uguaaacauccAacacucucagc....................................................................   1       1   seq
....................uguaaacauccuacacucuAagc....................................................................   1       1   seq
....................uguaaacaucUuacacucucagc....................................................................   1       1   seq
....................uguaaacaGccuacacucucagcu...................................................................   1       1   seq
....................uguaaacauccuacacucucagAu...................................................................   3       1   seq
....................uguaaacauccuacacucucaUcu...................................................................   2       1   seq
....................uguaaacauccuacacCcucagcu...................................................................   2       1   seq
....................Aguaaacauccuacacucucagcu...................................................................   11      1   seq
....................uguaaacauccuacaAucucagcu...................................................................   2       1   seq
....................uguaaacauccuacacucGcagcu...................................................................   1       1   seq
....................uguaaacauccuGcacucucagcu...................................................................   1       1   seq
....................uguaaacauccuacacucucagcA...................................................................   163     1   seq
....................uguaGacauccuacacucucagcu...................................................................   2       1   seq
....................uAuaaacauccuacacucucagcu...................................................................   3       1   seq
....................uguaaacauccuacGcucucagcu...................................................................   3       1   seq
....................uguaaacauccuacacucucagcC...................................................................   10      1   seq
....................uguaaacauccuacacucuUagcu...................................................................   4       1   seq
....................Cguaaacauccuacacucucagcu...................................................................   7       1   seq
....................uguaaacaucGuacacucucagcu...................................................................   1       1   seq
....................uguaaacauccuacacAcucagcu...................................................................   5       1   seq
....................ugCaaacauccuacacucucagcu...................................................................   3       1   seq
....................uguaaacauccuacUcucucagcu...................................................................   1       1   seq
....................uguaaacauccuacacucucagcu...................................................................   17216   0   seq
....................uguaaacauGcuacacucucagcu...................................................................   1       1   seq
....................uguaaacauAcuacacucucagcu...................................................................   2       1   seq
....................uguaaaAauccuacacucucagcu...................................................................   3       1   seq
....................ugAaaacauccuacacucucagcu...................................................................   1       1   seq
....................uguaaacCuccuacacucucagcu...................................................................   1       1   seq
....................uCuaaacauccuacacucucagcu...................................................................   5       1   seq
....................Gguaaacauccuacacucucagcu...................................................................   8       1   seq
....................uguaaacauccuacaGucucagcu...................................................................   1       1   seq
....................uguaaacauccuaAacucucagcu...................................................................   2       1   seq
....................uguaaacauccuacacuGucagcu...................................................................   1       1   seq
....................uguaaacauccCacacucucagcu...................................................................   2       1   seq
....................uguaaacauccuacacuUucagcu...................................................................   3       1   seq
....................uguaaacauccuaGacucucagcu...................................................................   1       1   seq
....................uguaaacauccuacacucucagUu...................................................................   4       1   seq
....................uguaaacauUcuacacucucagcu...................................................................   7       1   seq
....................uguaaacaucUuacacucucagcu...................................................................   2       1   seq
....................uguaaacauccuacacuAucagcu...................................................................   2       1   seq
....................uguaaacauccuaUacucucagcu...................................................................   6       1   seq
....................uguaaacauccuacacucucagcG...................................................................   125     1   seq
....................uguaaacauccuacacucucGgcu...................................................................   3       1   seq
....................uguaaacauccuacacucucaCcu...................................................................   1       1   seq
....................uguaaacaucAuacacucucagcu...................................................................   2       1   seq
....................uguaaaUauccuacacucucagcu...................................................................   3       1   seq
....................uguaaacauccuacacucuAagcu...................................................................   3       1   seq
....................uguaaacauccuacacucAcagcu...................................................................   1       1   seq
....................uUuaaacauccuacacucucagcu...................................................................   1       1   seq
....................uguaaacauccuacacucucagcug..................................................................   3       0   seq
....................uguaaacauccuacacucucagcuC..................................................................   3       1   seq
....................uguaaacauccuacacucucagcuU..................................................................   222     1   seq
....................uguaaacauccuacacucucagcuA..................................................................   7       1   seq
....................uguaaacauccuacacucucagcuAu.................................................................   6       1   seq
....................uguaaacauccuacacAcucagcugu.................................................................   1       1   seq
....................uguaaacauccuacacucucagcuUu.................................................................   24      1   seq
....................uguaaacauccuacacucucagcugu.................................................................   22      0   seq
....................uguaaacauccuacacucucagcugA.................................................................   1       1   seq
....................uguaaacauccuacacucucagcuCu.................................................................   1       1   seq
....................uguaaacauccuacacucucagcugug................................................................   2       0   seq
....................uguaaacauccuacacucucagcugugga..............................................................   1       0   seq
....................uguaaacauccuacacucucagcuguggaa.............................................................   3       0   seq
.....................guaaacauccuacacucucagc....................................................................   1       0   seq
.....................guaaacauccuacacucucagcu...................................................................   5       0   seq
```

```
                    Mature                                      Star
gagcucugagugacagguaguguaaacauccuacacucucagcuguggaaacuaagaaagcugggagaaggcuguuuacucucccugccuuggaaaucaucuggagaacaa
......................uaaacauccuacacucuca......................................................................  1      0      seq
......................uaaacauccuacacucucagcu...................................................................  2      0      seq
..........................cauccuacacucucagcu...................................................................  1      0      seq
.........................................................aagcugggagaaggcuguuuacu...............................  1      0      seq
............................................................cugggagaaggcuguuuac................................  1      0      seq
............................................................cugggagaaggcuguuuacA...............................  1      1      seq
............................................................Uugggagaaggcuguuuacu...............................  1      1      seq
............................................................cugggagaaggcuguuuacu...............................  23     0      seq
............................................................cugggagaaggcuguuuacuc..............................  24     0      seq
............................................................cugggagaaggGuguuuacucu.............................  1      1      seq
............................................................cugggagaagUcuguuuacucu.............................  1      1      seq
............................................................cugggagaaggcuguuuacucC.............................  3      1      seq
............................................................cugggagaaggAuguuuacucu.............................  1      1      seq
............................................................cugggagaaggcuguuuacucu.............................  343    0      seq
............................................................cugggagaaggcugAuuacucu.............................  1      1      seq
............................................................cugggagaaggcuguuuacucA.............................  3      1      seq
............................................................cugCgagaaggcuguuuacucu.............................  1      1      seq
............................................................cugggagaaggcuguuuacucuA............................  1      1      seq
............................................................cugggagaaggcuguuuacucuU............................  12     1      seq
.............................................................ugggagaaggcuguuuacucu.............................  5      0      seq
.............................................................ugggagaaggcuguuuacucuU............................  1      1      seq
..................................................................................cccugccuuggaaaucaucug........  2      0      seq
..................................................................................cccugccuuggaaaucaucuggagaac..  1      0      seq
```

Provisional ID	:	mun-miR-454
Score total	:	5.2
Score for star read(s)	:	-1.3
Score for read counts	:	0
Score for mfe	:	1.9
Score for randfold	:	1.6
Score for cons. seed	:	3
Total read count	:	1268
Mature read count	:	1266
Loop read count	:	0
Star read count	:	2

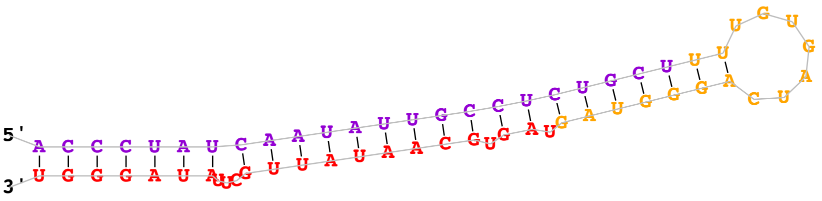

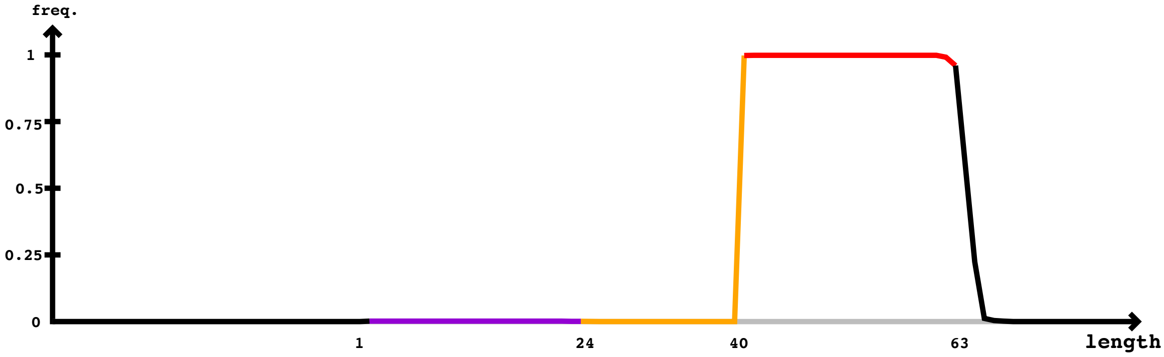


```
                                              Star                                  Mature
5'- cauuauccagcuuccuaaccuuaaggaugagacccuaucaauauugccucugcuuuugugaucaggguaguagugcaauauugcuuauagggucuuuucuuuugaggguua  -3'   obs
    cauuauccagcuuccuaaccuuaaggaugagacccuaucaauauugccucugcuuuugugaucaggguaguagugcaauauugcuuauagggucuuuucuuuugaggguua         exp
    ...............(((((((((((.(((((((((((((((((((((((((((((.......))))))).)).)))))))))...)))))))))).))))).)))))))  reads  mm  sample
    ...............................acccuaucaauauugccucug...........................................................  1      0   seq
    ...............................acccuaucaauauugccucugcu.........................................................  1      0   seq
    .....................................................................uagugcaauauugcuuauagg.....................  9      0   seq
    .....................................................................uagugcaauauugcuuauaggA....................  4      1   seq
    .....................................................................uagugcaauauugcuuauaggg....................  34     0   seq
    .....................................................................uagugcaauauugcuuauaggU....................  2      1   seq
    .....................................................................uagugcaauauugcuuauagggG...................  4      1   seq
    .....................................................................uagugcaauauugcuuauagggu...................  689    0   seq
    .....................................................................uagugcaauauugcuuauagggA...................  16     1   seq
    .....................................................................Cagugcaauauugcuuauagggu...................  1      1   seq
    .....................................................................uagugcaauauugcuuauagggC...................  2      1   seq
    .....................................................................uagugcaauauugcuuauaggguc..................  189    0   seq
    .....................................................................uagugcaauauCgcuuauaggguc..................  1      1   seq
    .....................................................................uagugcaauauugcuuauaggguG..................  2      1   seq
    .....................................................................uagugcaauauugcuuauagggGc..................  1      1   seq
    .....................................................................uagugcaauauugcuuauaggguU..................  21     1   seq
    .....................................................................uagugcaauauugcuuauaggguA..................  5      1   seq
    .....................................................................Aagugcaauauugcuuauaggguc..................  1      1   seq
    .....................................................................uagugcaauauugcuuauagggucu.................  256    0   seq
    .....................................................................uagugcaauauugcuuauaggguUu.................  3      1   seq
    .....................................................................uagugcaauauugcuuauagggucA.................  3      1   seq
    .....................................................................uagugcaauauugcuuauaggguAu.................  2      1   seq
    .....................................................................uagCgcaauauugcuuauagggucu.................  1      1   seq
    .....................................................................uagugcaauauugcuuauagggucG.................  2      1   seq
    .....................................................................uagugcaauauugcuuauagggucC.................  2      1   seq
    .....................................................................uagugcaauauugcuuauagggucAu................  3      1   seq
    .....................................................................uagugcaauauugcuuauagggucuu................  7      0   seq
    .....................................................................uagugcaauauugcuuauagggucuuu...............  3      0   seq
    .....................................................................uagugcaauauugcuuauagggucuuuu..............  2      0   seq
    ......................................................................agugcaauauugcuuauaggguc..................  1      0   seq
```

Provisional ID : mun-miR-202
Score total : 13766.7
Score for star read(s) : 3.9
Score for read counts : 13759.8
Score for mfe : 2
Score for randfold : 1.6
Score for cons. seed : -0.6
Total read count : 27001
Mature read count : 26695
Loop read count : 0
Star read count : 306

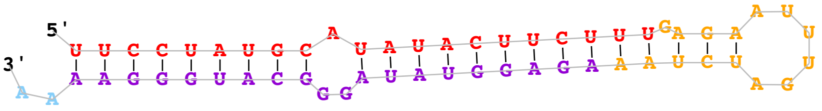

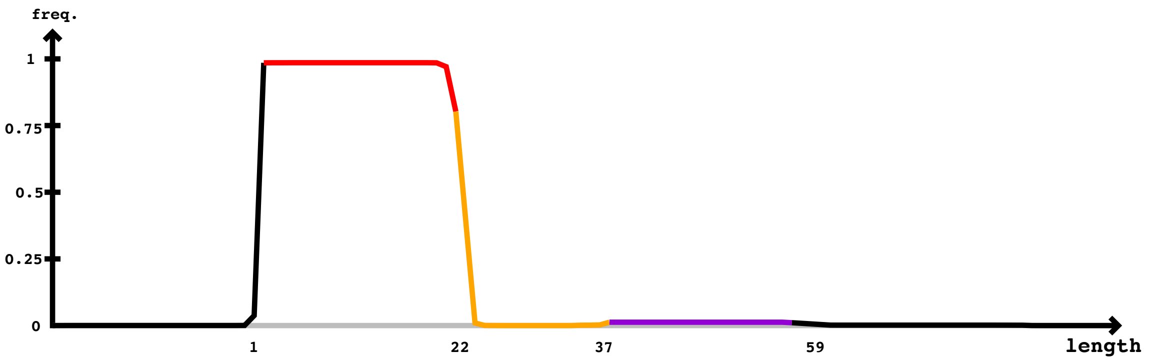

Mature Star

```
5'- aucuggcucguuguuccuuuuuccuaugcauauacuucuuugagaauuugaucuaaagagguauagggcaugggaaaauggggcaacugagguacuucaccauucauuu  -3'  obs
    aucuggcucguuguuccuuuuuccuaugcauauacuucuuugagaauuugaucuaaagagguauagggcaugggaaaauggggcaacugagguacuucaccauucauuu       exp
    ...((.(((((((((((.(((((((((((.(((((((((((.(((......))))))))))))))..))))))))))).)))))))).))).))...............  reads  mm  sample
    aucuggcucguuguuccuu..........................................................................................  2      0   seq
    aucuggcucguuguuccuuu.........................................................................................  3      0   seq
    ..................uuuuccuaugcauauacuucuu.....................................................................  2      0   seq
    ..................Guuuccuaugcauauacuucuuu....................................................................  1      1   seq
    ..................uuuuccuaugcauauacuucuuu....................................................................  1      0   seq
    ..................Cuuuccuaugcauauacuucuuu....................................................................  1      1   seq
    ...................uuuccuaugcauauaUuucu......................................................................  1      1   seq
    ...................uuuccuaugUauauacuucu......................................................................  1      1   seq
    ...................uuuccuaugcauauacuucu......................................................................  16     0   seq
    ...................uuuccuaugcauauacuucuu.....................................................................  275    0   seq
    ...................uuuccuaugUauauacuucuuu....................................................................  1      1   seq
    ...................uAuccuaugcauauacuucuuu....................................................................  1      1   seq
    ...................uuuccuaugcauauacuucuuC....................................................................  1      1   seq
    ...................Auuccuaugcauauacuucuuu....................................................................  2      1   seq
    ...................Guuccuaugcauauacuucuuu....................................................................  1      1   seq
    ...................Cuuccuaugcauauacuucuuu....................................................................  3      1   seq
    ...................uuuccuaugcauauacuucuuu....................................................................  685    0   seq
    ...................uuuccuaugcaCauacuucuuu....................................................................  1      1   seq
    ...................uuuccuaugcauauaAuucuuu....................................................................  1      1   seq
    ...................uuuccuaugcauauacuucuuug...................................................................  2      0   seq
    ...................uuuccuaugcauauacuucuuuA...................................................................  5      1   seq
    ...................uuuccuaugcauauacuucuuuU...................................................................  9      1   seq
    ...................uuuccuaugcauauacuucuuuCa..................................................................  1      1   seq
    ...................uuuccuaugcauauacuucuuuga..................................................................  1      0   seq
    ....................uuccuaugcaCauacuuc.......................................................................  1      1   seq
    ....................uuccuaugcauauacuuc.......................................................................  10     0   seq
    ....................uuccuaugcauauacuucu......................................................................  345    0   seq
    ....................uuccuaugUauauacuucu......................................................................  31     1   seq
    ....................Cuccuaugcauauacuucu......................................................................  1      1   seq
    ....................uuccuaugcauauacuucG......................................................................  1      1   seq
    ....................uuccuaugUauauacuucuu.....................................................................  60     1   seq
    ....................uuccuaugcauauacuAcuu.....................................................................  1      1   seq
    ....................uuccuaugcauauacuCcuu.....................................................................  1      1   seq
```

```
                    Mature                          Star
aucuggcucguuguuccuuuuuccuaugcauauacuucuuugagaauuugaucuaaagagguauagggcaugggaaaauggggcaacugagguacuucaccauucauuu
....................uuccCaugcauauacuucuu.....................................................................    3       1    seq
....................uuccuauUcauauacuucuu.....................................................................    2       1    seq
....................uuccuaugcauauacuucuu.....................................................................    4171    0    seq
....................uucGuaugcauauacuucuu.....................................................................    1       1    seq
....................uucAuaugcauauacuucuu.....................................................................    2       1    seq
....................uuccuaugcauauaAuucuu.....................................................................    2       1    seq
....................uuccuaugcauauacuucCu.....................................................................    2       1    seq
....................uAccuaugcauauacuucuu.....................................................................    1       1    seq
....................Auccuaugcauauacuucuu.....................................................................    2       1    seq
....................uuccuaugcauauacuucuG.....................................................................    1       1    seq
....................uuccuaugcauauCcuucuu.....................................................................    1       1    seq
....................Guccuaugcauauacuucuu.....................................................................    5       1    seq
....................uuccuaugcaCauacuucuu.....................................................................    5       1    seq
....................Cuccuaugcauauacuucuu.....................................................................    2       1    seq
....................uuccuaugcauauacCucuu.....................................................................    1       1    seq
....................uuccuaugcauGuacuucuu.....................................................................    1       1    seq
....................uuccuaugcauauaUuucuu.....................................................................    1       1    seq
....................uuAcuaugcauauacuucuu.....................................................................    3       1    seq
....................uuccuaugcauauGcuucuuu....................................................................    2       1    seq
....................uuccuaugcGuauacuucuuu....................................................................    6       1    seq
....................uuccuaugUauauacuucuuu....................................................................    41      1    seq
....................uuccuaugcauauacuucuCu....................................................................    1       1    seq
....................uuUcuaugcauauacuucuuu....................................................................    2       1    seq
....................uuccuaugcauauacuuAuuu....................................................................    3       1    seq
....................uuccuaugcauauacuCcuuu....................................................................    1       1    seq
....................uuccuaugcaCauacuucuuu....................................................................    16      1    seq
....................uAccuaugcauauacuucuuu....................................................................    5       1    seq
....................uuccuaugcauaCacuucuuu....................................................................    2       1    seq
....................uuccuauCcauauacuucuuu....................................................................    2       1    seq
....................uuGcuaugcauauacuucuuu....................................................................    1       1    seq
....................uuccuaugcauauacuuUuuu....................................................................    2       1    seq
....................uuAcuaugcauauacuucuuu....................................................................    1       1    seq
....................uuccuaugcauauacCucuuu....................................................................    2       1    seq
....................uuccuaugcauauacuucuuC....................................................................    2       1    seq
....................uuccuGugcauauacuucuuu....................................................................    1       1    seq
....................uuccuaugcauauaGuucuuu....................................................................    1       1    seq
....................uuccuaugcaAauacuucuuu....................................................................    1       1    seq
....................uuccuaugcauaAacuucuuu....................................................................    1       1    seq
....................uuccuaugcauauaAuucuuu....................................................................    3       1    seq
....................Auccuaugcauauacuucuuu....................................................................    10      1    seq
....................uuccuaugcauauacuucGuu....................................................................    1       1    seq
....................uuccuaugcauauacuucuuA....................................................................    37      1    seq
....................Guccuaugcauauacuucuuu....................................................................    15      1    seq
....................uuccuaugcauauacuucAuu....................................................................    1       1    seq
....................Cuccuaugcauauacuucuuu....................................................................    6       1    seq
....................uCccuaugcauauacuucuuu....................................................................    1       1    seq
....................uuccuaugcauUuacuucuuu....................................................................    2       1    seq
....................uuccuaCgcauauacuucuuu....................................................................    5       1    seq
....................uuccuaugcauauacuucuuu....................................................................    18986   0    seq
....................uuccuaugcauauacuucCuu....................................................................    8       1    seq
....................uuccuaugcauauacuucuuG....................................................................    4       1    seq
....................uucAuaugcauauacuucuuu....................................................................    2       1    seq
....................uuccuaugcauauaUuucuuu....................................................................    4       1    seq
....................uuccuauAcauauacuucuuu....................................................................    2       1    seq
....................uuccuaugAauauacuucuuu....................................................................    6       1    seq
....................uuccuaugcauauacuucuuug...................................................................    1015    0    seq
....................uuccuaugcauauacuucuuuC...................................................................    5       1    seq
....................uuccuaugcaCauacuucuuug...................................................................    1       1    seq
....................uuccCaugcauauacuucuuug...................................................................    1       1    seq
....................uuccuaugcauauacuucuuuA...................................................................    220     1    seq
....................uuccuaugcauauacuucuuuU...................................................................    328     1    seq
....................Guccuaugcauauacuucuuug...................................................................    1       1    seq
....................Auccuaugcauauacuucuuug...................................................................    1       1    seq
....................uuccuaugcauauacuucuuugC..................................................................    1       1    seq
....................uuccuaugcauauacuucuuugG..................................................................    2       1    seq
....................uuccuaugcauauacuucuuuUa..................................................................    3       1    seq
....................uuccuaugcauauacuucuuuga..................................................................    156     0    seq
....................uuccuaugcauauacuucuuugU..................................................................    53      1    seq
....................uuccuaugcGuauacuucuuuga..................................................................    1       1    seq
....................uuccuaugcauauacuuUuuuga..................................................................    1       1    seq
```

Mature **Star**

```
aucuggcucguuguuccuuuuuccuaugcauauacuucuuugagaauuugaucuaaagagguauagggcaugggaaaauggggcaacugagguacuucaccauucauuu
....................uuccuaugcauauacuucuuuAa..................................................................  31   1  seq
....................uuccuaugcauauacuucuuugaU.................................................................  1    1  seq
....................uuccuaugcauauacuucuuugag.................................................................  6    0  seq
....................uuccuaugcauauacuucuuugaA.................................................................  1    1  seq
....................uuccuaugcauauacuucuuugCga................................................................  1    1  seq
....................uuccuaugcauauacuucuuugaga................................................................  1    0  seq
.....................uccuaugcauauacuucuuu....................................................................  6    0  seq
.....................uccuaugcauauacuucuuug...................................................................  1    0  seq
.....................uccuaugcauauacuucuuugU..................................................................  1    1  seq
.......................cuaugcauauacuucuuu....................................................................  2    0  seq
.........................................gagaauuugaucuaaagagguauagggcaugggaaaau..............................  1    0  seq
.....................................................uaaagagguauagggcauggga..................................  8    0  seq
.....................................................uaaagagguauagggcaugggaa.................................  16   0  seq
.....................................................uaaagaggGauagggcaugggaa.................................  1    1  seq
.....................................................uaaagagguauagggcaugggaU.................................  2    1  seq
.....................................................uaaagagguauagggcaugggaaU................................  6    1  seq
.....................................................uaaagagguauagggcaugggaaa................................  1    0  seq
.....................................................uaaagagguauagggcaugggaaUa...............................  1    1  seq
......................................................aaagagguauagggcauggga..................................  2    0  seq
......................................................aaagagguauagggcaugggaa.................................  3    0  seq
......................................................aaagagguauagggcaugggaaU................................  1    1  seq
......................................................aaagagguauagggcaugggaaaau..............................  1    0  seq
.......................................................aagagguauagggcauggga..................................  4    0  seq
.......................................................aagagguauagggcaugggaa.................................  7    0  seq
.......................................................aagagguauagggcaugggaaU................................  2    1  seq
.......................................................aagagguauagggcaugggaaa................................  7    0  seq
.......................................................aagagguauagggcaugggaaaU...............................  3    1  seq
.......................................................aagagguauagggcaugggaaaa...............................  2    0  seq
.......................................................aagagguauagggcaugggaaaau..............................  1    0  seq
........................................................agagguauagggcauggg...................................  1    0  seq
........................................................agagguauagggcaugggC..................................  1    1  seq
........................................................agagguauagggcauggga..................................  35   0  seq
........................................................agagguauagggcaugggaa.................................  131  0  seq
........................................................agagguauagggcaugggaaC................................  1    1  seq
........................................................agagguauagggcaugggaaU................................  13   1  seq
........................................................agagguauagggcaugggaaG................................  1    1  seq
........................................................agagguauagggcaugggaaa................................  49   0  seq
........................................................agaggAauagggcaugggaaa................................  1    1  seq
........................................................agagguauagggcaugggaaaa...............................  15   0  seq
........................................................agagguauagggcaugggaaaU...............................  5    1  seq
........................................................agagguauagggcaugggaaaC...............................  2    1  seq
........................................................agagguauagggcaugggaaaau..............................  8    0  seq
........................................................agagguauagggcaugggaaaUu..............................  1    1  seq
........................................................agagguauagggcaugggaaaCu..............................  2    1  seq
........................................................agagguauagggcaugggaaaaC..............................  5    1  seq
........................................................agagguauagggcaugggaaaauU.............................  1    1  seq
........................................................agagguauagggcaugggaaaauA.............................  1    1  seq
..............................................................................uggggAaacugagguacuu............  1    1  seq
..............................................................................uggggcaacugagguacuu............  1    0  seq
..............................................................................uggggcaacugagguacuuc...........  2    0  seq
..............................................................................Cggggcaacugagguacuuc...........  1    1  seq
..............................................................................uggggcaacugagguacuuca..........  3    0  seq
..............................................................................uggggcaacugagguacuucaA.........  4    1  seq
..............................................................................uggggcaacugagguacuucac.........  29   0  seq
..............................................................................uggggcaacugagguacuucacc........  2    0  seq
```

Provisional ID	:	mun-novel99
Score total	:	13.6
Score for star read(s)	:	3.9
Score for read counts	:	6.8
Score for mfe	:	2
Score for randfold	:	1.6
Score for cons. seed	:	-0.6
Total read count	:	25
Mature read count	:	22
Loop read count	:	0
Star read count	:	3

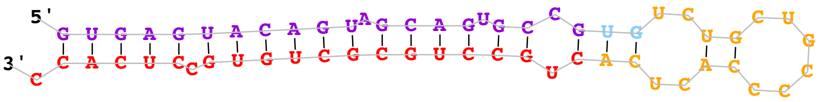

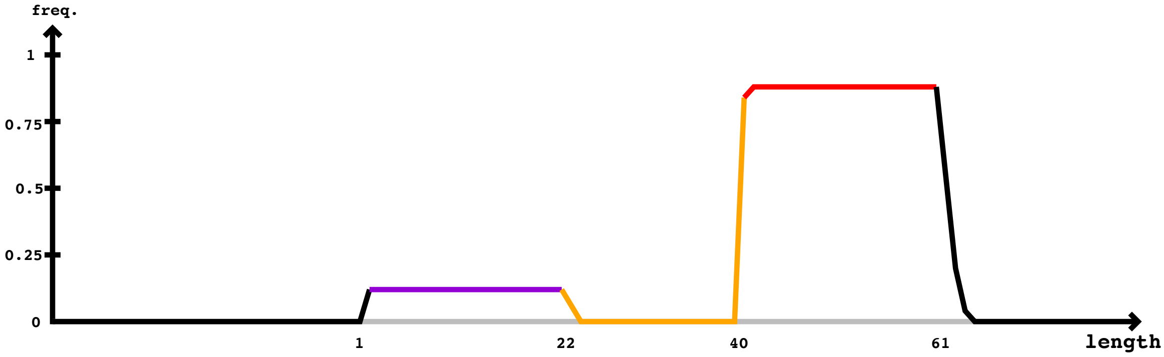

Star **Mature**

```
5'- caccauagagcacauacgccaggagcuggcggugaguacaguagcagugccgugucugcugccccacucacugccugcgcugugccucaccaccacccuucuccucgcagg  -3'   obs
    caccauagagcacauacgccaggagcuggcggugaguacaguagcagugccgugucugcugccccacucacugccugcgcugugccucaccaccacccuucuccucgcagg         exp
    .................((.(((((.(((.((((((((((((.((((.((.(((..((......))..))).)))))))))))))).)))))).))).....))))).))...   reads   mm   sample
    ...............................gugaguacaguagcagugccU...........................................................   1       1    seq
    ...............................gugaguacaguagcagugccg...........................................................   2       0    seq
    ......................................................................Gugccugcgcugugccucacc....................   1       1    seq
    ......................................................................cugccugcgcugugccucacc....................   7       0    seq
    ......................................................................cugccugcgcugugGcucacc....................   1       1    seq
    ......................................................................cugccugcgcugugccucaccC...................   4       1    seq
    ......................................................................cugccugcgcugugccucacca...................   4       0    seq
    ......................................................................cugccugcgcugugccucaccCc..................   2       1    seq
    ......................................................................cugccugcgcugugccucaccaU..................   1       1    seq
    ......................................................................cugccugcgcugugccucaccacU.................   1       1    seq
    .......................................................................ugccugcgcugugccucaccac..................   1       0    seq
```

Provisional ID	:	mun-miR-7-4
Score total	:	28.2
Score for star read(s)	:	3.9
Score for read counts	:	18.5
Score for mfe	:	1.2
Score for randfold	:	1.6
Score for cons. seed	:	3
Total read count	:	48
Mature read count	:	44
Loop read count	:	0
Star read count	:	4

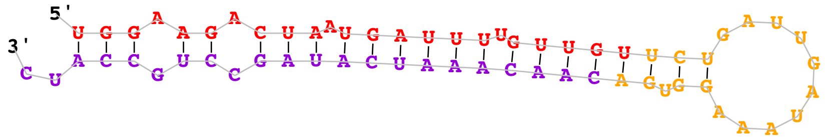

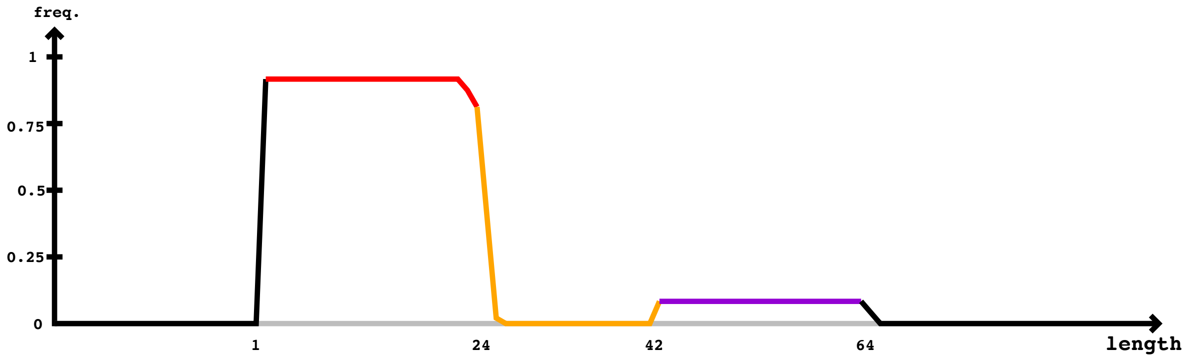

Mature **Star**

```
5'- ucucguggucugccucuguguggaagacuaaugauuuuguuguucugauugauaaaggugacaacaaaucauagccugccauccagcacagaccugcacccucgcugugccgu   -3'   obs
    ucucguggucugccucuguguggaagacuaaugauuuuguuguucugauugauaaaggugacaacaaaucauagccugccauccagcacagaccugcacccucgcugugccgu         exp
    ......((((((...(((..(((.((.(((.((((((.((((((((..........)).))))))))))))))).)).))).)))..)))..)))))).((((.......))))...   reads   mm   sample
    ....................uggaagacuaaugauuuuguu........................................................................   2       0    seq
    ....................uggaagacuaaugauuuuguug.......................................................................   3       0    seq
    ....................uggaagacuaaugauuuuguugu......................................................................   25      0    seq
    ....................uggaagacuaaugauuuuguuguu.....................................................................   13      0    seq
    ....................uggaagacuaaugauuuuguuguuU....................................................................   1       1    seq
    .............................................................caacaaaucauagccugccauc..............................   4       0    seq
```

Provisional ID	:	mun-novel98
Score total	:	0
Score for star read(s)	:	-1.3
Score for read counts	:	-2.8
Score for mfe	:	3.2
Score for randfold	:	1.6
Score for cons. seed	:	-0.6
Total read count	:	6
Mature read count	:	6
Loop read count	:	0
Star read count	:	0

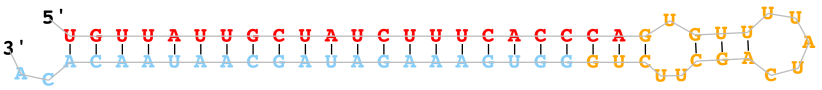

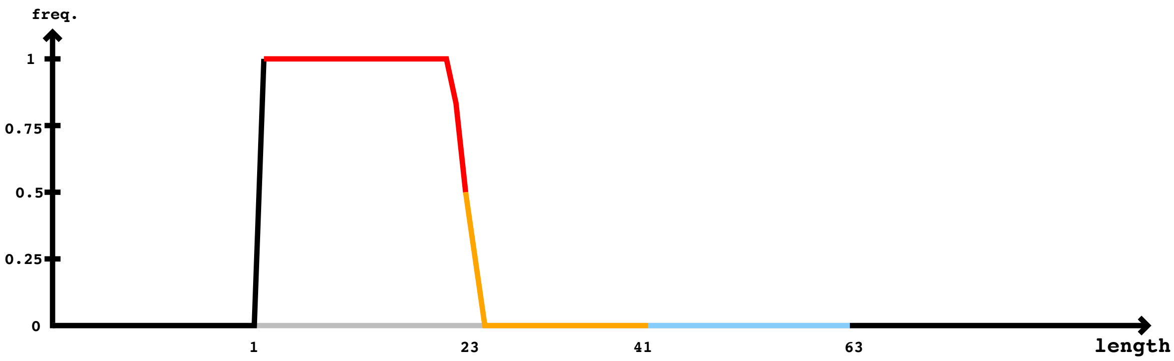

Mature Star

```
5'- gcuccugucccagcuccauguguuauugcuaucuuucacccaguguuuuaucagcuucugggugaaagauagcaauaacacauggagcugggacaggacugcccugggugga -3'   exp
    ..(((((((((((((((((((((((((((((((((((((((((.(((.....)))..)))))))))))))))))))))))))))))))))))))))))(..((...))..).    reads   mm   sample
    ....................uguuauugcuaucuuucacc........................................................................    1       0    seq
    ....................uguuauugcuaucuuucaccc.......................................................................    2       0    seq
    ....................uguuauugcuaucuuucaccca......................................................................    2       0    seq
    ....................uguuauugcuaucuuucacccaU.....................................................................    1       1    seq
```

Provisional ID	:	mun-miR-144
Score total	:	2901.1
Score for star read(s)	:	3.9
Score for read counts	:	2891.3
Score for mfe	:	1.3
Score for randfold	:	1.6
Score for cons. seed	:	3
Total read count	:	5683
Mature read count	:	4516
Loop read count	:	0
Star read count	:	1167

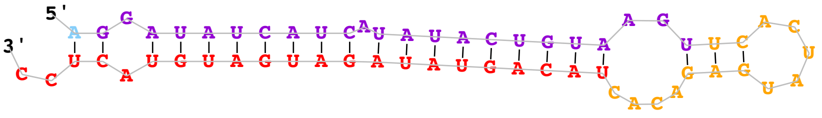

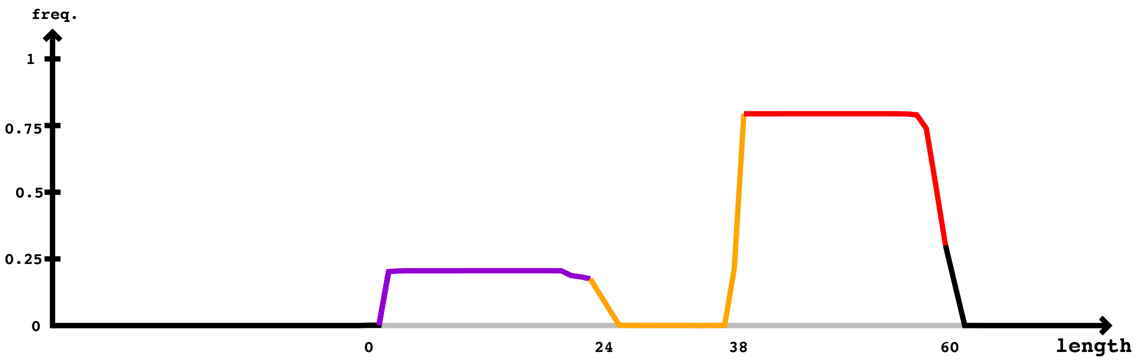

Star Mature

```
5'- ccccauggcacacaccacgcgcugcccugggcaggauaucaucauauacuguaaguucacuaugagacacuacaguauagaugauguacucccugugcugcaccacc  -3'  obs
    ccccauggcacacaccacgcgcugcccugggcaggauaucaucauauacuguaaguucacuaugagacacuacaguauagaugauguacucccugugcugcaccacc        exp
    .....(((......)))...((.((.(.(((.((.((((((((.((((((((..(((.....)))....)))))))))))))))).)).))).).)).))......   reads  mm  sample
    ...............................caggauaucaucauauacugu........................................................   6      0   seq
    .................................ggauaucaucauauacug.........................................................   2      0   seq
    .................................ggauaucaucauauacugu........................................................   95     0   seq
    .................................ggauaucaucauauacugCa.......................................................   1      1   seq
    .................................ggauaucaucauauacugua.......................................................   26     0   seq
    .................................ggauaucaucauauacuguaa......................................................   44     0   seq
    .................................ggauaucaucauauacuguaag.....................................................   157    0   seq
    .................................ggauaucaucauauacuguaaU.....................................................   1      1   seq
    .................................ggauaucaucaCauacuguaag.....................................................   1      1   seq
    .................................ggauaucaucauauacuguaaA.....................................................   3      1   seq
    .................................ggauaucaucauauacuguaGgu....................................................   1      1   seq
    .................................ggauaucaucauauacuguaagA....................................................   16     1   seq
    .................................ggauaucauUauauacuguaagu....................................................   1      1   seq
    .................................ggauaucaucauauacuguaagu....................................................   743    0   seq
    .................................ggauaucaAcauauacuguaagu....................................................   1      1   seq
    .................................ggauaucaucauauacuguaagG....................................................   1      1   seq
    .................................ggauaucaucaCauacuguaagu....................................................   1      1   seq
    .................................ggauaucaucauauacuguaaguu...................................................   38     0   seq
    .................................ggauaucaucauauacuguaaguA...................................................   5      1   seq
    .................................ggauaucaucauauacuguaaguuc..................................................   2      0   seq
    .................................ggauaucaucauauacuguaaguuU..................................................   4      1   seq
    .................................ggauaucaucauauacuguaaguucU.................................................   1      1   seq
    .................................ggauaucaucauauacuguaaguucacu...............................................   1      0   seq
    ..................................gauaucaucauauacuguaagu....................................................   7      0   seq
    ..................................gauaucaucauauacuguaaUu....................................................   1      1   seq
    ..................................gauaucaucauauacuguaagA....................................................   1      1   seq
    ..................................gauaucaucauauacuguaaguu...................................................   5      0   seq
    ...................................auaucaucauauacuguaagu....................................................   1      0   seq
    ...................................auaucaucauauacuguaaguu...................................................   2      0   seq
    ...................................auaucaucauauacuguaaguucacuauga...........................................   1      0   seq
    .........................................ucauauacuguaaguucacuaugaga.........................................   1      0   seq
    ...................................................................cacuacaguauagaugauguac...................   1      0   seq
    ...................................................................cacuacaguauagaugauguacu..................   1      0   seq
```

Star **Mature**

```
ccccauggcacacaccacgcgcugcccugggcaggauaucaucauauacuguaaguucacuaugagacacuacaguauagaugauguacucccugugcugcaccacc
....................................................................acuacaguauagaugaug......................	1	0	seq
....................................................................acuacaguauagaugauguacuc................	1	0	seq
.....................................................................cuacaguauagaugaugu.....................	4	0	seq
.....................................................................cuacaguauagaugaugua....................	4	0	seq
.....................................................................cuacaguauGgaugauguac...................	2	1	seq
.....................................................................cuacaguauagaugauguac...................	117	0	seq
.....................................................................Guacaguauagaugauguacu..................	1	1	seq
.....................................................................cuacaguauagaugauguaUu..................	8	1	seq
.....................................................................cuacaguauGgaugauguacu..................	11	1	seq
.....................................................................cuacaguauagaugauguacG..................	1	1	seq
.....................................................................cuacaguauagaugauguacu..................	403	0	seq
.....................................................................cuacaguauagaugauguacuc.................	290	0	seq
.....................................................................cuacaguauagaugauguacuU.................	2	1	seq
.....................................................................cuacagCauagaugauguacuc.................	1	1	seq
.....................................................................cuacaguauagaugaugGacuc.................	1	1	seq
.....................................................................cuacaguauagaugauguacucc................	1	0	seq
.....................................................................cuacaguauagaugauguacucU................	363	1	seq
......................................................................uacaguauagaugaugua....................	14	0	seq
......................................................................uacaguauagaugauguaA...................	1	1	seq
......................................................................uacaguauagaugauguac...................	170	0	seq
......................................................................Aacaguauagaugauguacu..................	1	1	seq
......................................................................uacagAauagaugauguacu..................	1	1	seq
......................................................................uacaguauagaugauguacu..................	783	0	seq
......................................................................uacaguauagaugaugGacu..................	1	1	seq
......................................................................uacaguauagaugaCguacu..................	1	1	seq
......................................................................uacCguauagaugauguacu..................	1	1	seq
......................................................................uacaguauGgaugauguacu..................	12	1	seq
......................................................................uacaguauagaugauguaUu..................	3	1	seq
......................................................................uacagGauagaugauguacu..................	1	1	seq
......................................................................Cacaguauagaugauguacuc.................	1	1	seq
......................................................................uacaguauagaugaugAacuc.................	1	1	seq
......................................................................uacaguauGgaugauguacuc.................	2	1	seq
......................................................................uacaguauagaugaugGacuc.................	1	1	seq
......................................................................uacaguauagaugauguacuc.................	959	0	seq
......................................................................Gacaguauagaugauguacuc.................	1	1	seq
......................................................................uacaguauagaugauCuacuc.................	1	1	seq
......................................................................uacaguauagaugauguacuU.................	2	1	seq
......................................................................uacaguauagaugauAuacuc.................	1	1	seq
......................................................................uacaguauagaugauguaUuc.................	2	1	seq
......................................................................uacaguauagaugauguacucG................	1	1	seq
......................................................................uacaguauagaugauguacucA................	3	1	seq
......................................................................uacaguauagaugauguacucc................	5	0	seq
......................................................................uacaguauagaugauguacucU................	1331	1	seq
......................................................................uacaguauagaugauguacuccU...............	2	1	seq
......................................................................uacaguauagaugauguacuccA...............	1	1	seq
......................................................................uacaguauagaugauguacucUccu.............	1	1	seq
..........................................................................guauagaugauguacucU................	1	1	seq
```

Provisional ID	:	mun-miR-489
Score total	:	34.5
Score for star read(s)	:	3.9
Score for read counts	:	27.7
Score for mfe	:	1.9
Score for randfold	:	1.6
Score for cons. seed	:	-0.6
Total read count	:	66
Mature read count	:	65
Loop read count	:	0
Star read count	:	1

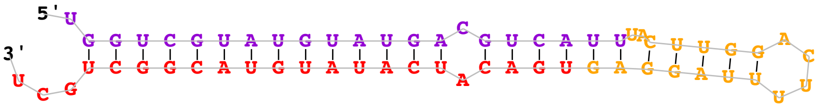

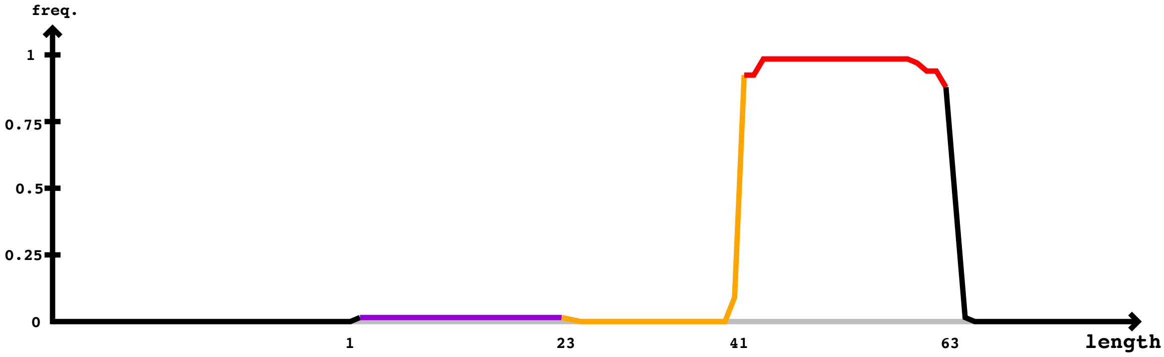


```
                                         Star                                       Mature
5'- auaacagucauuugucacgugguggcuugguggucguauguaugacgucauuuacuuggacuuuuaggagugacaucauauguacggcugcuaaacugcugcaugggacau   -3'   obs
    auaacagucauuugucacgugguggcuugguggucguauguaugacgucauuuacuuggacuuuuaggagugacaucauauguacggcugcuaaacugcugcaugggacau         exp
    ............((((..(..(..(.((((..(((((((((((((.((((((..(((((....))))))))))).)))))))))))))..)))).)..)..)....)))).   reads   mm   sample
    ..............................uggucguauguaugacgucauu...........................................................   1       0    seq
    .....................................................................gugacaucauauguacggcugc....................   3       0    seq
    .....................................................................gugacaucauauguacggcugcu...................   3       0    seq
    ......................................................................ugacaucauauguacggc.......................   1       0    seq
    ......................................................................ugacaucauauguacggcu......................   2       0    seq
    ......................................................................ugacaucauauguacggcugc....................   1       0    seq
    ......................................................................ugacaucauauguacggcugcu...................   43      0    seq
    ......................................................................ugacaucauauguacggcugcG...................   2       1    seq
    ......................................................................ugacaucauauguacggcugcC...................   1       1    seq
    ......................................................................ugacaucauauguacggcugcA...................   1       1    seq
    ......................................................................ugacaucauauguacggcugcuU..................   4       1    seq
    ........................................................................acaucauauguacggcugcu...................   1       0    seq
    ........................................................................acaucauauguacggcugcuU..................   1       1    seq
    ........................................................................acaucauauguacggcugcCa..................   1       1    seq
    ........................................................................acaucauauguacggcugcuaU.................   1       1    seq
```

Provisional ID	:	mun-miR-30b
Score total	:	89093
Score for star read(s)	:	3.9
Score for read counts	:	89082.4
Score for mfe	:	2.1
Score for randfold	:	1.6
Score for cons. seed	:	3
Total read count	:	174743
Mature read count	:	170876
Loop read count	:	1
Star read count	:	3866

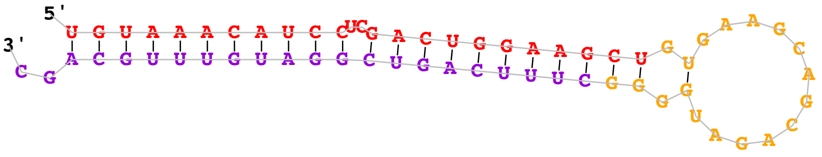

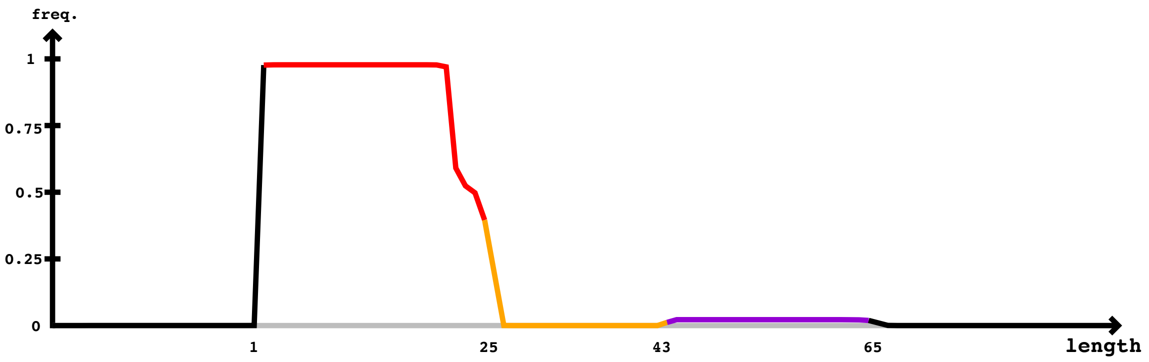

Mature Star

```
5'- ugcuguugacagugagcgacuguaaacauccucgacuggaagcugugaagcagcagauggggcuuucagucggauguuugcagcugccaacugccacagacgucaagaa  -3'   obs
    ugcuguugacagugagcgacuguaaacauccucgacuggaagcugugaagcagcagauggggcuuucagucggauguuugcagcugccaacugccacagacgucaagaa        exp
    ..((((.(.((((..(((.((((((((((((..((((((((((.(............).))))))))))))))))))))))).)))..))))).)))).........   reads   mm   sample
    ugcuguugacagugagcgac.........................................................................................   3       0    seq
    .gcuguugacagugagcgac.........................................................................................   2       0    seq
    ..cuguugacagugagcgac.........................................................................................   5       0    seq
    ..................aAuguaaacauccucgacuggaagc..................................................................   1       1    seq
    ...................Uuguaaacauccucgacugga.....................................................................   1       1    seq
    ...................cuguaaacauccucgacugga.....................................................................   11      0    seq
    ...................cuguaaacauccucgacuggaagcu.................................................................   4       0    seq
    ...................Auguaaacauccucgacuggaagcu.................................................................   1       1    seq
    ...................cuguaaacauccCcgacuggaagcu.................................................................   1       1    seq
    ....................uguaaacauccucgaGug.......................................................................   1       1    seq
    ....................uguaaacauccucgacug.......................................................................   92      0    seq
    ....................uguaaaAauccucgacug.......................................................................   1       1    seq
    ....................uguaaacauGcucgacugg......................................................................   1       1    seq
    ....................uguaaacauccucgacugg......................................................................   1238    0    seq
    ....................uguaaacauUcucgacugg......................................................................   1       1    seq
    ....................uguaaacauccAcgacugg......................................................................   1       1    seq
    ....................uguaaacauccucgacugU......................................................................   16      1    seq
    ....................uguaaacauccucgacugA......................................................................   19      1    seq
    ....................Cguaaacauccucgacugg......................................................................   2       1    seq
    ....................uguaaacauccucgacCgg......................................................................   1       1    seq
    ....................uguaaaAauccucgacugga.....................................................................   7       1    seq
    ....................uguaaGcauccucgacugga.....................................................................   6       1    seq
    ....................uCuaaacauccucgacugga.....................................................................   5       1    seq
    ....................uguaaacauccucgacugga.....................................................................   65462   0    seq
    ....................uguaaacaucUucgacugga.....................................................................   27      1    seq
    ....................uguaaacauccucgacugCa.....................................................................   10      1    seq
    ....................uguaaacaucGucgacugga.....................................................................   2       1    seq
    ....................Aguaaacauccucgacugga.....................................................................   30      1    seq
    ....................ugAaaacauccucgacugga.....................................................................   5       1    seq
    ....................uguaUacauccucgacugga.....................................................................   2       1    seq
    ....................uguaaacGuccucgacugga.....................................................................   3       1    seq
    ....................uAuaaacauccucgacugga.....................................................................   9       1    seq
    ....................uguaaacauccuGgacugga.....................................................................   1       1    seq
```

Mature Star

```
ugcuguugacagugagcgacuguaaacauccucgacuggaagcugugaagcagcagauggggcuuucagucggauguuugcagcugccaacugccacagacgucaagaa
....................uguaaacauccucgaGugga.....................................................................  2      1  seq
....................ugCaaacauccucgacugga.....................................................................  11     1  seq
....................ugGaaacauccucgacugga.....................................................................  3      1  seq
....................uguaaacauccAcgacugga.....................................................................  7      1  seq
....................uguaaacauccucgGcugga.....................................................................  4      1  seq
....................uguaaacauccucgacAgga.....................................................................  3      1  seq
....................uguaaacaucAucgacugga.....................................................................  11     1  seq
....................uguaGacauccucgacugga.....................................................................  3      1  seq
....................uguaaacauccucgacuggC.....................................................................  40     1  seq
....................uguaaaUauccucgacugga.....................................................................  3      1  seq
....................uguaaacauAcucgacugga.....................................................................  8      1  seq
....................uguaaacaCccucgacugga.....................................................................  7      1  seq
....................uguaaUcauccucgacugga.....................................................................  3      1  seq
....................uguaaacauUcucgacugga.....................................................................  38     1  seq
....................uguaaaGauccucgacugga.....................................................................  1      1  seq
....................uguaaacauccucgacGgga.....................................................................  1      1  seq
....................uguaaacauccucgUcugga.....................................................................  4      1  seq
....................Gguaaacauccucgacugga.....................................................................  59     1  seq
....................uguaaacauccucgaUugga.....................................................................  8      1  seq
....................uguaaacauccucgacugUa.....................................................................  8      1  seq
....................Cguaaacauccucgacugga.....................................................................  19     1  seq
....................uguaaacauccucUacugga.....................................................................  8      1  seq
....................uguaaacauccucgacuUga.....................................................................  9      1  seq
....................uguaaacauccGcgacugga.....................................................................  1      1  seq
....................uguaaacauccucgacuggG.....................................................................  239    1  seq
....................uguaaacauccucgacuAga.....................................................................  2      1  seq
....................uguaaacUuccucgacugga.....................................................................  2      1  seq
....................uUuaaacauccucgacugga.....................................................................  9      1  seq
....................uguaaacauccucAacugga.....................................................................  6      1  seq
....................uguaaacauGcucgacugga.....................................................................  3      1  seq
....................uguaaacauccuAgacugga.....................................................................  10     1  seq
....................uguaaacauccucgaAugga.....................................................................  9      1  seq
....................uguaaacauccucgacugAa.....................................................................  8      1  seq
....................uguaaCcauccucgacugga.....................................................................  2      1  seq
....................uguaaacauccucgacCgga.....................................................................  6      1  seq
....................uguaaacauccucCacugga.....................................................................  3      1  seq
....................uguaaacauccucgacuggU.....................................................................  126    1  seq
....................uguUaacauccucgacugga.....................................................................  2      1  seq
....................uguaaacauccucgacuCga.....................................................................  8      1  seq
....................uguGaacauccucgacugga.....................................................................  4      1  seq
....................uguaaacauccucgacuAgaa....................................................................  1      1  seq
....................uguaaacauAcucgacuggaa....................................................................  3      1  seq
....................uguaaacauccucgacugAaa....................................................................  1      1  seq
....................uguaaacaucUucgacuggaa....................................................................  9      1  seq
....................uguaaacauccucAacuggaa....................................................................  1      1  seq
....................ugAaaacauccucgacuggaa....................................................................  3      1  seq
....................uguaaacauccucgacGggaa....................................................................  1      1  seq
....................Cguaaacauccucgacuggaa....................................................................  1      1  seq
....................ugGaaacauccucgacuggaa....................................................................  1      1  seq
....................uguaaacauccuGgacuggaa....................................................................  1      1  seq
....................uguaaacauccucgacuggaC....................................................................  3      1  seq
....................uguaaacauccucgacugCaa....................................................................  1      1  seq
....................uguaaacauccucgacAggaa....................................................................  1      1  seq
....................Gguaaacauccucgacuggaa....................................................................  11     1  seq
....................uguaaacauccucgacuggaa....................................................................  11198  0  seq
....................uguaaacauccucgacuggGa....................................................................  3      1  seq
....................uAuaaacauccucgacuggaa....................................................................  1      1  seq
....................uCuaaacauccucgacuggaa....................................................................  1      1  seq
....................uguaaacauccucgacugUaa....................................................................  3      1  seq
....................uguaaacauccucgacuUgaa....................................................................  2      1  seq
....................uguaaacauccucgacuggaG....................................................................  26     1  seq
....................uguaaacauUcucgacuggaa....................................................................  107    1  seq
....................uguaaacauccucgacuggUa....................................................................  3      1  seq
....................uguaaacauccAcgacuggaa....................................................................  2      1  seq
....................uguaaacaAccucgacuggaa....................................................................  1      1  seq
....................uguaaaUauccucgacuggaa....................................................................  1      1  seq
....................uguaaacauccucgacuCgaa....................................................................  1      1  seq
....................uUuaaacauccucgacuggaa....................................................................  2      1  seq
....................ugCaaacauccucgacuggaa....................................................................  5      1  seq
....................uguaaacaucAucgacuggaa....................................................................  1      1  seq
```

Mature Star

```
ugcuguugacagugagcgacuguaaacauccucgacuggaagcugugaagcagcagauggggcuuucagucggauguuugcagcugccaacugccacagacgucaagaa
....................uguaaacauccucgacuggaU....................................................................  215    1  seq
....................uguaaacGuccucgacuggaa....................................................................  3      1  seq
....................Aguaaacauccucgacuggaa....................................................................  10     1  seq
....................uguaaacauccuAgacuggaa....................................................................  1      1  seq
....................uguaaacauccucUacuggaa....................................................................  4      1  seq
....................uguaaacauccucgacCggaag...................................................................  1      1  seq
....................uguaaacauccucgaUuggaag...................................................................  1      1  seq
....................uguaaacauccucgacuggaag...................................................................  3150   0  seq
....................Gguaaacauccucgacuggaag...................................................................  3      1  seq
....................uguaaacauccucUacuggaag...................................................................  1      1  seq
....................uguaaacaucUucgacuggaag...................................................................  10     1  seq
....................uguaaacGuccucgacuggaag...................................................................  1      1  seq
....................uguaaacauccuAgacuggaag...................................................................  1      1  seq
....................uguaaacauccucgacuUgaag...................................................................  2      1  seq
....................uguaaacauccucgacuggaaC...................................................................  3      1  seq
....................uguaaacaucAucgacuggaag...................................................................  1      1  seq
....................ugAaaacauccucgacuggaag...................................................................  1      1  seq
....................ugCaaacauccucgacuggaag...................................................................  1      1  seq
....................uguaaacauccucgacuggaGg...................................................................  2      1  seq
....................Aguaaacauccucgacuggaag...................................................................  2      1  seq
....................uguaaacaucGucgacuggaag...................................................................  1      1  seq
....................uguaaacauccucgacuggaUg...................................................................  1      1  seq
....................uguaaacauccucgacuggaaA...................................................................  1230   1  seq
....................uguaaacauccAcgacuggaag...................................................................  1      1  seq
....................uguaaacauccucgUcuggaag...................................................................  1      1  seq
....................uguaaacauUcucgacuggaag...................................................................  11     1  seq
....................ugGaaacauccucgacuggaag...................................................................  1      1  seq
....................uguaaacauccucgacuggaaU...................................................................  169    1  seq
....................uguaaacauccucgacuCgaag...................................................................  1      1  seq
....................ugGaaacauccucgacuggaagc..................................................................  1      1  seq
....................uguaaacauccAcgacuggaagc..................................................................  7      1  seq
....................uguaaacaucUucgacuggaagc..................................................................  33     1  seq
....................uguaaacaucGucgacuggaagc..................................................................  2      1  seq
....................uguaaacauccucgacuggaagU..................................................................  3377   1  seq
....................ugCaaacauccucgacuggaagc..................................................................  2      1  seq
....................uguaaacauccucgacAggaagc..................................................................  1      1  seq
....................uguaaacauccGcgacuggaagc..................................................................  1      1  seq
....................uguaaacauccucgaAuggaagc..................................................................  2      1  seq
....................uguaaacauccucgacuggaagG..................................................................  4      1  seq
....................uUuaaacauccucgacuggaagc..................................................................  4      1  seq
....................uguaaacauccucgacuggCagc..................................................................  1      1  seq
....................uguaaacauccucgacuggaagc..................................................................  12277  0  seq
....................uguaaacauccucgacugAaagc..................................................................  2      1  seq
....................uguaaacauccucgacuUgaagc..................................................................  6      1  seq
....................uguaaaUauccucgacuggaagc..................................................................  3      1  seq
....................uguaaacaucAucgacuggaagc..................................................................  3      1  seq
....................uCuaaacauccucgacuggaagc..................................................................  2      1  seq
....................uguaaacauUcucgacuggaagc..................................................................  10     1  seq
....................uguaaUcauccucgacuggaagc..................................................................  1      1  seq
....................uguaaacauccucgacugUaagc..................................................................  1      1  seq
....................uguaaacauGcucgacuggaagc..................................................................  1      1  seq
....................Aguaaacauccucgacuggaagc..................................................................  12     1  seq
....................ugAaaacauccucgacuggaagc..................................................................  2      1  seq
....................uguaaacauccucgacuggGagc..................................................................  3      1  seq
....................uAuaaacauccucgacuggaagc..................................................................  2      1  seq
....................Gguaaacauccucgacuggaagc..................................................................  5      1  seq
....................uguaaacauccucgacuggaaCc..................................................................  1      1  seq
....................Cguaaacauccucgacuggaagc..................................................................  1      1  seq
....................uguaaacauccucgacuggaGgc..................................................................  1      1  seq
....................uguaaacaGccucgacuggaagc..................................................................  1      1  seq
....................uguaaaAauccucgacuggaagc..................................................................  1      1  seq
....................uguaaacauccuAgacuggaagc..................................................................  3      1  seq
....................uguaaacauccucgUcuggaagc..................................................................  1      1  seq
....................uguaaacauccucgacugCaagc..................................................................  1      1  seq
....................uguaaacGuccucgacuggaagc..................................................................  1      1  seq
....................uguaaacauccucgacuggaCgc..................................................................  1      1  seq
....................uguaGacauccucgacuggaagc..................................................................  3      1  seq
....................uguaaacauccucgacuggaagA..................................................................  2040   1  seq
....................uguaaacauccuGgacuggaagc..................................................................  1      1  seq
....................uguaaacauccucgaUuggaagc..................................................................  1      1  seq
```

Mature Star

```
ugcuguugacagugagcgacuguaaacauccucgacuggaagcugugaagcagcagauggggcuuucagucggauguuugcagcugccaacugccacagacgucaagaa
....................uguaaacauccucgacuCgaagc.................................................................. 3 1 seq
....................uguaaGcauccucgacuggaagc.................................................................. 1 1 seq
....................uguaaCcauccucgacuggaagcu................................................................. 3 1 seq
....................uguaaUcauccucgacuggaagcu................................................................. 1 1 seq
....................ugGaaacauccucgacuggaagcu................................................................. 2 1 seq
....................uguaaacaucAucgacuggaagcu................................................................. 10 1 seq
....................uguaUacauccucgacuggaagcu................................................................. 2 1 seq
....................ugCaaacauccucgacuggaagcu................................................................. 11 1 seq
....................uguUaacauccucgacuggaagcu................................................................. 1 1 seq
....................uguaaacaucGucgacuggaagcu................................................................. 1 1 seq
....................uguaaacauccucgacugUaagcu................................................................. 8 1 seq
....................uguaaacUuccucgacuggaagcu................................................................. 2 1 seq
....................uguaaacGuccucgacuggaagcu................................................................. 7 1 seq
....................uguaaacauccucgacAggaagcu................................................................. 2 1 seq
....................uguaaacauccucgacuCgaagcu................................................................. 6 1 seq
....................uguaaacauccucgaUuggaagcu................................................................. 5 1 seq
....................uguaaacauccucgacuggaUgcu................................................................. 1 1 seq
....................uguaaacauccucgacuggaGgcu................................................................. 5 1 seq
....................uguaaacauccucgacuggaagGu................................................................. 4 1 seq
....................uguaaacaucUucgacuggaagcu................................................................. 23 1 seq
....................uguaaacauccucgaGuggaagcu................................................................. 2 1 seq
....................Aguaaacauccucgacuggaagcu................................................................. 41 1 seq
....................uguaaacauccucgacuggaaUcu................................................................. 9 1 seq
....................uAuaaacauccucgacuggaagcu................................................................. 4 1 seq
....................uguaGacauccucgacuggaagcu................................................................. 9 1 seq
....................uguaaacauccucgacuggGagcu................................................................. 8 1 seq
....................uguaaacauccucgacuggaaAcu................................................................. 3 1 seq
....................uguaaacauccucgacuggaaCcu................................................................. 1 1 seq
....................uguaaacauccucgacuggaagcC................................................................. 65 1 seq
....................uguaaacauccucgacuggaagcG................................................................. 437 1 seq
....................uguaaacauccucgacuAgaagcu................................................................. 3 1 seq
....................uguaaacaAccucgacuggaagcu................................................................. 2 1 seq
....................uguaaacauUcucgacuggaagcu................................................................. 11 1 seq
....................uguaaacauccucUacuggaagcu................................................................. 11 1 seq
....................uguaaacauccucgCcuggaagcu................................................................. 2 1 seq
....................Cguaaacauccucgacuggaagcu................................................................. 21 1 seq
....................uguaaacauccucgacuggaagcu................................................................. 66365 0 seq
....................uguaaacauccGcgacuggaagcu................................................................. 3 1 seq
....................ugAaaacauccucgacuggaagcu................................................................. 7 1 seq
....................uguaaacauccuGgacuggaagcu................................................................. 4 1 seq
....................uguaaacauccucgGcuggaagcu................................................................. 10 1 seq
....................uguaaaAauccucgacuggaagcu................................................................. 7 1 seq
....................uguaaacaGccucgacuggaagcu................................................................. 2 1 seq
....................uCuaaacauccucgacuggaagcu................................................................. 8 1 seq
....................uguaaacauccucgacuggaagUu................................................................. 146 1 seq
....................uUuaaacauccucgacuggaagcu................................................................. 6 1 seq
....................uguaaacauccucgUcuggaagcu................................................................. 2 1 seq
....................uguaaacaCccucgacuggaagcu................................................................. 5 1 seq
....................uguaaacauccucAacuggaagcu................................................................. 7 1 seq
....................uguaaacauccucgacCggaagcu................................................................. 14 1 seq
....................uguaaacauAcucgacuggaagcu................................................................. 7 1 seq
....................Gguaaacauccucgacuggaagcu................................................................. 52 1 seq
....................uguaaacauccucgaAuggaagcu................................................................. 7 1 seq
....................uguaaaUauccucgacuggaagcu................................................................. 14 1 seq
....................uguGaacauccucgacuggaagcu................................................................. 9 1 seq
....................uguaaGcauccucgacuggaagcu................................................................. 4 1 seq
....................uguaaacauccucgacuggaCgcu................................................................. 2 1 seq
....................uguaaacauccucgacuUgaagcu................................................................. 20 1 seq
....................uguaaacauccucgacuggaagAu................................................................. 13 1 seq
....................uguaaacauccucgacugAaagcu................................................................. 2 1 seq
....................uguaaacauccucCacuggaagcu................................................................. 4 1 seq
....................uguaaacauccucgacuggaagcA................................................................. 275 1 seq
....................uguaaacauccAcgacuggaagcu................................................................. 15 1 seq
....................uguaaacauccuAgacuggaagcu................................................................. 9 1 seq
....................uguaaacauccucgacugCaagcu................................................................. 1 1 seq
....................uguaaacauccucgacuggaagcuA................................................................ 10 1 seq
....................uguaaacauccucgacuggaagcCg................................................................ 1 1 seq
....................uguaaacauccucgacuggaagcuU................................................................ 1159 1 seq
....................uguaaacauccucgacuggaagcuC................................................................ 2 1 seq
....................uguaaacauccucgacuggaagcug................................................................ 1 0 seq
```

Mature **Star**

```
ugcuguugacagugagcgacuguaaacauccucgacuggaagcugugaagcagcagauggggcuuucagucggauguuugcagcugccaacugccacagacgucaagaa
....................uguaaacauccucgacuggaagcuUu...............................................................   39    1    seq
....................uguaaacauccuUgacuggaagcugu...............................................................   2     1    seq
....................uguaaacauccucgacuggaagcuCu...............................................................   2     1    seq
....................uguaaacauccucgacuggaagcuAu...............................................................   5     1    seq
....................uguaaacauccucgacuggaagcugu...............................................................   7     0    seq
....................uguaaacauccucgacuggaagcugGga.............................................................   1     1    seq
.....................guaaacauccucgacugga.....................................................................   10    0    seq
.....................guaaacauccucgacuggaa....................................................................   5     0    seq
.....................guaaacauccucgacuggaag...................................................................   1     0    seq
.....................guaaacauccucgacuggaaU...................................................................   1     1    seq
.....................guaaacauccucgacuggaaA...................................................................   1     1    seq
.....................guaaacauccucgacuggaagc..................................................................   7     0    seq
.....................guaaacaucUucgacuggaagc..................................................................   1     1    seq
.....................guaaacauccucgaAuggaagcu.................................................................   1     1    seq
.....................Uuaaacauccucgacuggaagcu.................................................................   1     1    seq
.....................guaaacauccucgacuggaagcG.................................................................   1     1    seq
.....................guaaacauccucgacuggaagcu.................................................................   143   0    seq
.....................guaaacauccucgacuggaagcA.................................................................   2     1    seq
.....................guaaacauccucgacuggaagcuU................................................................   2     1    seq
......................uaaacauccucgacuggaa....................................................................   1     0    seq
......................uaaacauccucgacuggaag...................................................................   1     0    seq
......................uaaacauccucgacuggaagc..................................................................   1     0    seq
......................uaaacauccucgacuggaagA..................................................................   1     1    seq
......................uaaacauccucgacuggaagcu.................................................................   7     0    seq
......................uaaacauccucgacuggaagcugu...............................................................   1     0    seq
.......................aaacauccucgacuggaagc..................................................................   1     0    seq
........................aacauccucgacuggaagcu.................................................................   1     0    seq
.........................acauccucgacuggaagcu.................................................................   1     0    seq
..........................cauccucgacuggaagcu.................................................................   2     0    seq
...........................................ugugaagcagcagauggggc..............................................   1     0    seq
..............................................................cuuucagucggauguuug.............................   1     0    seq
..............................................................cuuucagucggauguuugc............................   44    0    seq
..............................................................cuuucagucgUauguuugc............................   1     1    seq
..............................................................cuuucagucggauguuugcU...........................   1     1    seq
..............................................................cuuucagucggaugGuugca...........................   1     1    seq
..............................................................cuuucagucggauguuugca...........................   52    0    seq
..............................................................cuuucagucggauguuugcag..........................   252   0    seq
..............................................................cuuucagucAgauguuugcag..........................   1     1    seq
..............................................................Guuucagucggauguuugcag..........................   1     1    seq
..............................................................cuuucagucggauguuugcaU..........................   1     1    seq
..............................................................cuuucagCcggauguuugcag..........................   1     1    seq
..............................................................cuuucagucggauguuugGag..........................   1     1    seq
..............................................................cuuucagucggauguuugcaA..........................   18    1    seq
..............................................................cuuucagucggaugAuugcag..........................   1     1    seq
..............................................................cuuucagucggauguuugAagc.........................   1     1    seq
..............................................................cuuucGgucggauguuugcagc.........................   1     1    seq
..............................................................Guuucagucggauguuugcagc.........................   2     1    seq
..............................................................cuuucagucggauguuugUagc.........................   1     1    seq
..............................................................cuuucagucggauguCugcagc.........................   1     1    seq
..............................................................cuuucaguAggauguuugcagc.........................   1     1    seq
..............................................................cuuucagucggauguuuUcagc.........................   1     1    seq
..............................................................Uuuucagucggauguuugcagc.........................   1     1    seq
..............................................................cCuucagucggauguuugcagc.........................   1     1    seq
..............................................................cuuucagucggauguuugcagc.........................   1291  0    seq
..............................................................cuuuGagucggauguuugcagc.........................   1     1    seq
..............................................................cuuucagucggauguuugcagA.........................   8     1    seq
..............................................................cuuucagucggauguuugcagU.........................   92    1    seq
..............................................................cuuucagucggauguuugcagUu........................   9     1    seq
..............................................................cuuucagucggauguuugcagcG........................   3     1    seq
..............................................................cuuucagucggauguuuAcagcu........................   125   1    seq
..............................................................cuuucagucggauguuugcagcA........................   35    1    seq
..............................................................cuuucagucggauguuugcagcC........................   16    1    seq
..............................................................cuuucagucggauguuugcagcu........................   100   0    seq
..............................................................cuuucagucggauguuugcagcuA.......................   2     1    seq
..............................................................cuuucagucggauguuugcagcCg.......................   1     1    seq
..............................................................cuuucagucggauguuugcagcuU.......................   11    1    seq
..............................................................Uuuucagucggauguuugcagcug.......................   1     1    seq
...............................................................uuucagucggauguuugc............................   12    0    seq
...............................................................uuucagucggauguuugca...........................   10    0    seq
...............................................................uuucagucggauguuugcaU..........................   3     1    seq
```

Mature **Star**

ugcuguugacagugagcgacuguaaacauccucgacuggaagcugugaagcagcagauggggcuuucagucggauguuugcagcugccaacugccacagacgucaagaa			
....uuucagucggauguuugcag..........................	109	0	seq
....Cuucagucggauguuugcag...........................	1	1	seq
....uuucagucggauguuugcaA..........................	7	1	seq
....uuucagucggauguuugcUgc.........................	2	1	seq
....uuucGgucggauguuugcagc.........................	1	1	seq
....uuucagucggauguuugcagc.........................	662	0	seq
....uuucagucggauguuugcagU.........................	50	1	seq
....uuucagucggauguuugcagA.........................	11	1	seq
....uuucagucggauguuugcagcG........................	2	1	seq
....uuucagucggauguuugcagcC........................	98	1	seq
....uuucagucggauguuugcagUu........................	6	1	seq
....uuucagucggauguuugcagcA........................	2	1	seq
....uuucagucggauguuugcagcu........................	338	0	seq
....uuucagucggauguuugcagAu........................	1	1	seq
....Cuucagucggauguuugcagcu.........................	1	1	seq
....uuucagucggauguuuAcagcu........................	392	1	seq
....uuucagucggauguuugcUgcu........................	3	1	seq
....uuucagucggauguuugcagcuU.......................	65	1	seq
....uuucagucggauguuugcagcuA.......................	4	1	seq
....uuucagucggauguuugcagcugU......................	2	1	seq
....uucagucggauguuugcagc.........................	1	0	seq
....uucagucggauguuuAcagcu........................	2	1	seq

Provisional ID	:	mun-miR-218
Score total	:	900.5
Score for star read(s)	:	3.9
Score for read counts	:	890.3
Score for mfe	:	1.7
Score for randfold	:	1.6
Score for cons. seed	:	3
Total read count	:	1758
Mature read count	:	1748
Loop read count	:	0
Star read count	:	10

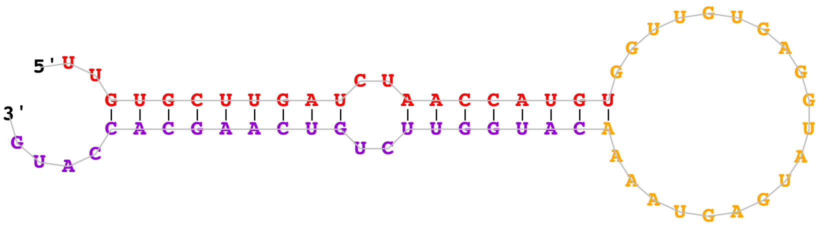

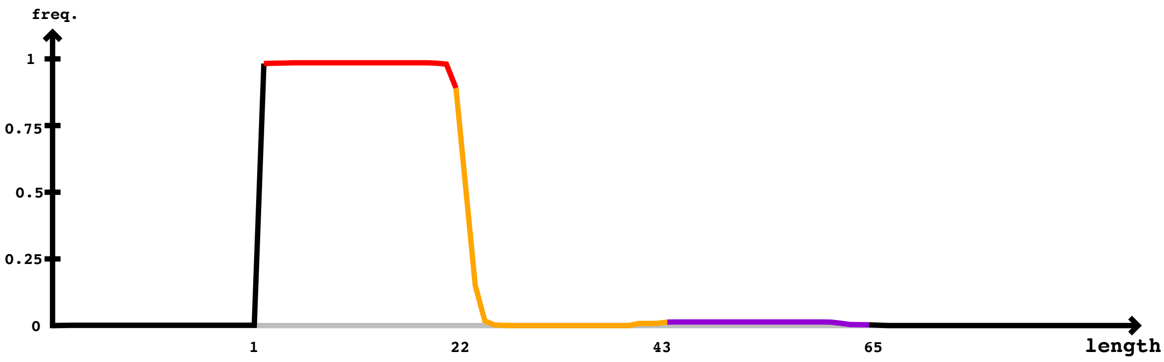

Mature **Star**

```
5'- uaauguagcgagauuuucuguugugcuugaucuaaccauguggguugugagguaugaguaaaacaugguucugucaagcaccauggaacgucacgcagcuuucuacagcaug  -3'  obs
    uaauguagcgagauuuucuguugugcuugaucuaaccauguggguugugagguaugaguaaaacaugguucugucaagcaccauggaacgucacgcagcuuucuacagcaug       exp
    .......(((.(((.((((((.(((((((((..((((((((....................))))))))..))))))))).)))))).))).))).(((......)))...  reads  mm  sample
    uaauguagcgagauuuucug...........................................................................................  2      0   seq
    ....................uugugcuugaucuaacca.........................................................................  3      0   seq
    ....................uugugcuugaucuaaccau........................................................................  6      0   seq
    ....................uugugcuugGucuaaccaug.......................................................................  1      1   seq
    ....................uugugcuugaucuaaccaug.......................................................................  158    0   seq
    ....................uugugcuugaucuaacAaugu......................................................................  1      1   seq
    ....................uugugAuugaucuaaccaugu......................................................................  1      1   seq
    ....................uugugcuugaucuaaccaugG......................................................................  1      1   seq
    ....................uugugcuugaucuaaccaugu......................................................................  951    0   seq
    ....................uugugcuugaucuaaccauguU.....................................................................  10     1   seq
    ....................uugugcuugaucuaaccaugug.....................................................................  331    0   seq
    ....................uugugcuugaucuaaccauguA.....................................................................  15     1   seq
    ....................uugugcuugaucuaaccaugugU....................................................................  179    1   seq
    ....................uugugcuugaucuaaccaugugg....................................................................  15     0   seq
    ....................uugugcuugaucuaaccaugugC....................................................................  1      1   seq
    ....................uugugcuugaucuaaccaugugA....................................................................  42     1   seq
    ....................uugugcuugaucuaaccaugugUu...................................................................  9      1   seq
    ....................uugugcuugaucuaaccaugugAu...................................................................  8      1   seq
    ....................uugugcuugaucuaaccauguggu...................................................................  8      0   seq
    ....................uugugcuugaucuaaccaugugUuu..................................................................  3      1   seq
    .....................ugugcuugaucuaaccaugu......................................................................  1      0   seq
    .....................ugugcuugaucuaaccauguggu...................................................................  1      0   seq
    ......................gugcuugaucuaaccaug.......................................................................  1      0   seq
    .......................ugcuugaucuaaccauguggu...................................................................  1      0   seq
    .......................ugcuugaucuaaccaugugguuU.................................................................  1      1   seq
    ...........................................................aaacaugguucugucaagca................................  1      0   seq
    ...........................................................aaacaugguucugucaagcac...............................  5      0   seq
    ...........................................................aaacaugguucugucaagcaA...............................  1      1   seq
    ...........................................................aaacaugguucugucaagcacc..............................  5      0   seq
    ...........................................................aaacaugguucugucaagcacU..............................  2      1   seq
    .............................................................acaugguucugucaagcac...............................  1      0   seq
    ..............................................................caugguucugucaagcacc..............................  2      0   seq
    ..............................................................caugguucugucaagcacca.............................  1      0   seq
```

Mature Star

```
uaauguagcgagauuuucuguugugcuugaucuaaccauguggguugugagguaugaguaaaacaugguucugucaagcaccauggaacgucacgcagcuuucuacagcaug
..............................................................caugguucugucaagcaccau............................	1	0	seq
..............................................................caugguucugucaagcaccauU...........................	3	1	seq
..............................................................caugguucugucaagcaccauC...........................	1	1	seq
...............................................................augguucugucaagcaccaugU..........................	1	1	seq
```

Provisional ID	:	mun-novel89
Score total	:	42.6
Score for star read(s)	:	3.9
Score for read counts	:	41.4
Score for mfe	:	0.1
Score for randfold	:	-2.2
Score for cons. seed	:	-0.6
Total read count	:	93
Mature read count	:	60
Loop read count	:	0
Star read count	:	33

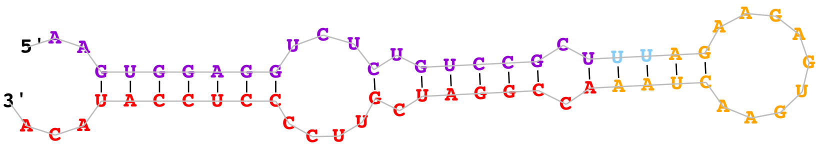

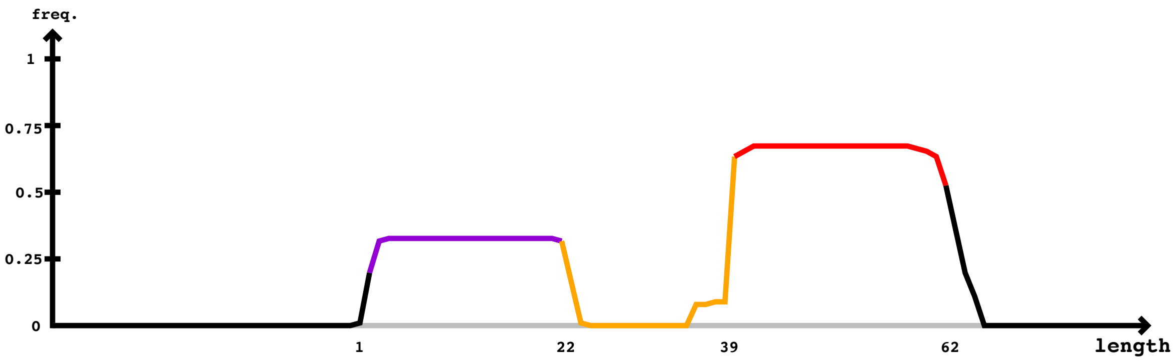

Star Mature

```
5'- uuuguccaccauugcagucuucccagagcaaaaguggaggucucuguccgcuuuagaagagugaacuaaaccggaucguuccccuccauacaccuacguuauccuguaaugu  -3'  obs
    uuuguccaccauugcagucuucccagagcaaaaguggaggucucuguccgcuuuagaagagugaacuaaaccggaucguuccccuccauacaccuacguuauccuguaaugu       exp
    .........((((((((..(..(...((.....(((((((...(.(((((.(((((.........))))).))))).)....)))))))....))..)..)..)))))))).   reads  mm  sample
    ..............................aaaguggaggucucuguccgcu............................................................   1      0   seq
    ...............................aaguggaggucucuguccgcu............................................................   16     0   seq
    ...............................aaguggaggCcucuguccgcu............................................................   1      1   seq
    ...............................aaguggaggucucuguccgcuu...........................................................   2      0   seq
    ................................aguggaggucucuguccgc.............................................................   1      0   seq
    ................................aguggaggucucuguccgcu............................................................   9      0   seq
    ................................aguggaggucucuguccgcuu...........................................................   1      0   seq
    ................................aguggaggucucuguccgcuuu..........................................................   1      0   seq
    .................................guggaggucucuguccgcuu...........................................................   1      0   seq
    .................................................................cuaaaccggaucguuccccuccauacacc..................   1      0   seq
    .................................................................cuaaaccggaucguuccccuccauacaccu.................   7      0   seq
    ...................................................................aaaccggaucguuccccucca........................   1      0   seq
    .....................................................................accggaucguuccccuccau.......................   1      0   seq
    .....................................................................accggaucguuccccuccauU......................   2      1   seq
    .....................................................................accggaucguuccccuccauac.....................   11     0   seq
    .....................................................................accggaucguuccccuccauacC....................   1      1   seq
    .....................................................................accggaucguuccccuccauacU....................   21     1   seq
    .....................................................................accggaucguuccccuccauaca....................   5      0   seq
    .....................................................................accgUaucguuccccuccauacac...................   1      1   seq
    .....................................................................accggaucguuccccuccauacac...................   2      0   seq
    .....................................................................accggaucguuccccuccauacaA...................   1      1   seq
    .....................................................................accggaucguuccccuccauacacc..................   8      0   seq
    .....................................................................accggaucguuccccuccauacaccu.................   2      0   seq
    ......................................................................ccggaucguuccccuccauacC....................   1      1   seq
    ......................................................................ccggaucguuccccuccauacaU...................   1      1   seq
    .......................................................................cggaucguuccccuccauacaccu.................   2      0   seq
```

Provisional ID	:	mun-novel3
Score total	:	3.3
Score for star read(s)	:	3.9
Score for read counts	:	-3.9
Score for mfe	:	2.4
Score for randfold	:	1.6
Score for cons. seed	:	-0.6
Total read count	:	4
Mature read count	:	3
Loop read count	:	0
Star read count	:	1

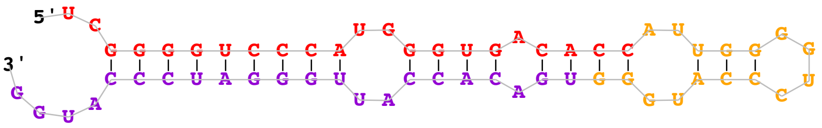

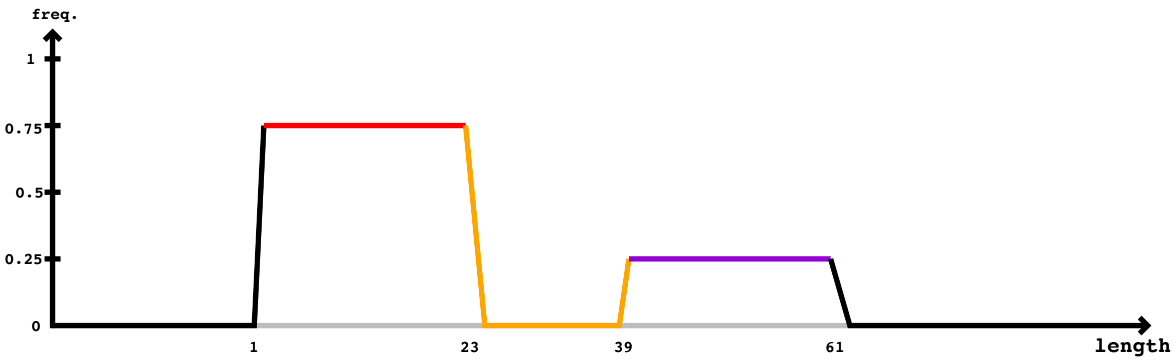

Mature **Star**

```
5'- cagugacaagggugacaccaucggggucccaugggugacaccauuggggucccauggguugacaccauugggaucccaugggugccaccauucccuguccccggcacagccgc   -3'    obs
    cagugacaagggugacaccaucggggucccaugggugacaccauuggggucccauggguugacaccauugggaucccaugggugccaccauucccuguccccggcacagccgc          exp
    (((.((....((((.((((...(((((((((..((((.((((..(((....)))..)))).))))..)))))))))...)))).)))).)).)))....((((...)))).   reads   mm   sample
    ....................ucggggucccaugggugacacc......................................................................   3       0    seq
    ..........................................................ugacaccauugggaucccaugg................................   1       0    seq
```

Provisional ID : mun-novel96
Score total : 2.6
Score for star read(s) : -1.3
Score for read counts : 0
Score for mfe : 2.9
Score for randfold : 1.6
Score for cons. seed : -0.6
Total read count : 27
Mature read count : 27
Loop read count : 0
Star read count : 0

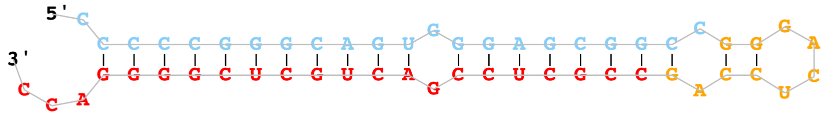

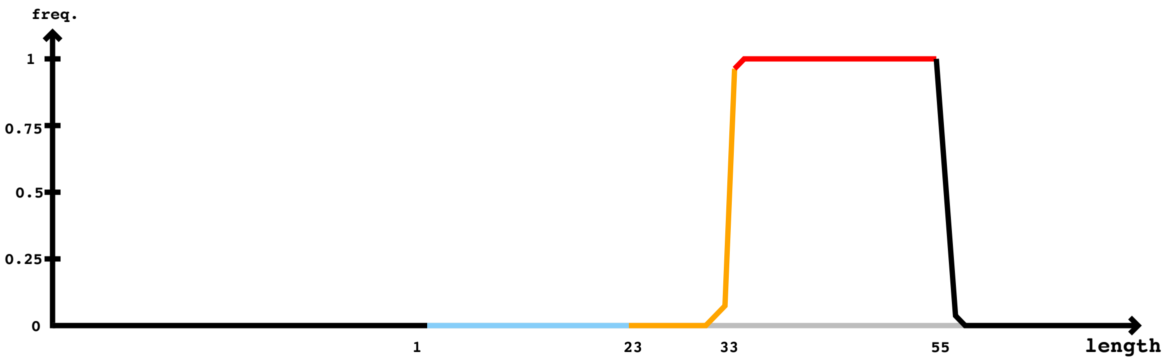

Star Mature

```
5'- cccgaaccgagcuccuccucgcguuuccaccguacggcccccgggcagugggagcggccgggacuccagccgcuccgacugcucggggaccgugcggggaagucgcuccug   -3'    exp
    ....................(((((((.((((((((.(((((((((((.((((((((.((....)).)))))))).))))))))))).))))))))))))).))).....   reads   mm   sample
    ...................................................................agccgcuccgacugcucggggacU....................   1       1    seq
    ....................................................................gccgcuccgacugcucggggacU....................   1       1    seq
    .....................................................................Gcgcuccgacugcucggggacc....................   1       1    seq
    .....................................................................ccgcuccgacugcucggggacU....................   19      1    seq
    .....................................................................ccgcuccgacugcucggggaccA...................   2       1    seq
    .....................................................................ccgcuccgacugcucggggaccU...................   1       1    seq
    .....................................................................ccgcuccgacugcucggggaccAu..................   1       1    seq
    ......................................................................cgcuccgacugcucggggaccA...................   1       1    seq
```

Provisional ID	:	mun-novel59
Score total	:	14.8
Score for star read(s)	:	3.9
Score for read counts	:	7.3
Score for mfe	:	2.6
Score for randfold	:	1.6
Score for cons. seed	:	-0.6
Total read count	:	26
Mature read count	:	25
Loop read count	:	0
Star read count	:	1

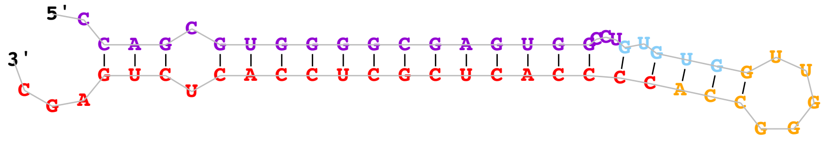

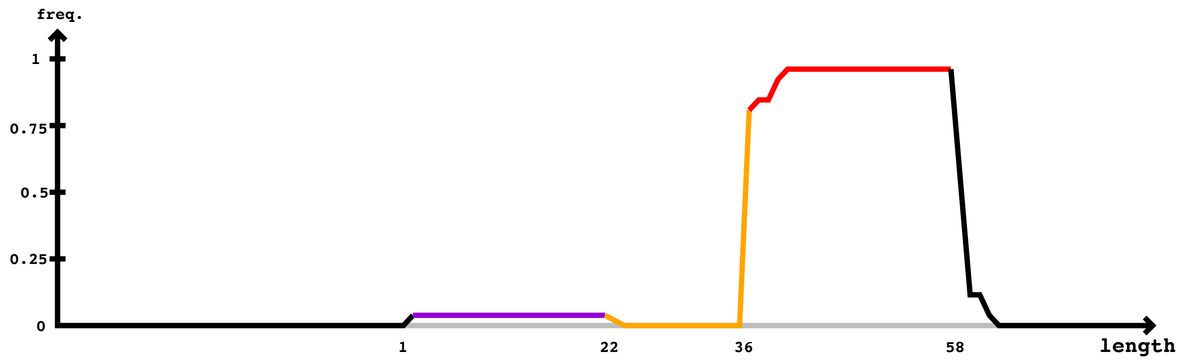

Star Mature

```
5'- aaaauggccuagcuaucgaugcugccuacaggagcccagcguggggcgaguggccugugugguugggccaccccacucgcuccacucugagcuccuguagauguggugccau  -3'   obs
    aaaauggccuagcuaucgaugcugccuacaggagcccagcguggggcgaguggccugugugguugggccaccccacucgcuccacucugagcuccuguagauguggugccau        exp
    ...((((((((((.......)))..((((((((((.(((.(((((((((((((...(.((((.....)))))))))))))))))).))).))))))))))....)).)))))   reads     mm        sample
    ...................................ccagcguggggcgaguggccA........................................................   1         1         seq
    ......................................................................ccccacucgcuccacucugagU....................   1         1         seq
    ......................................................................ccccacucgcuccacucugagc....................   13        0         seq
    ......................................................................ccccacucgcuccacucugagcu...................   3         0         seq
    ......................................................................ccccacucgcuccacucugagcA...................   3         1         seq
    ......................................................................ccccacucgcuccacucugagUu...................   1         1         seq
    .......................................................................cccacucgcuccacucugagcu...................   1         0         seq
    .........................................................................cacucgcuccacucugagcucU.................   1         1         seq
    .........................................................................cacucgcuccacucugagcuccu................   1         0         seq
    ..........................................................................acucgcuccacucugagcucU.................   1         1         seq
```

Provisional ID	:	mun-miR-135a-2
Score total	:	268.9
Score for star read(s)	:	3.9
Score for read counts	:	258.6
Score for mfe	:	1.8
Score for randfold	:	1.6
Score for cons. seed	:	3
Total read count	:	519
Mature read count	:	510
Loop read count	:	0
Star read count	:	9

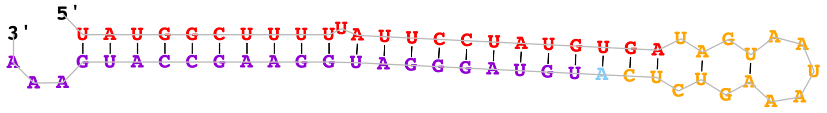

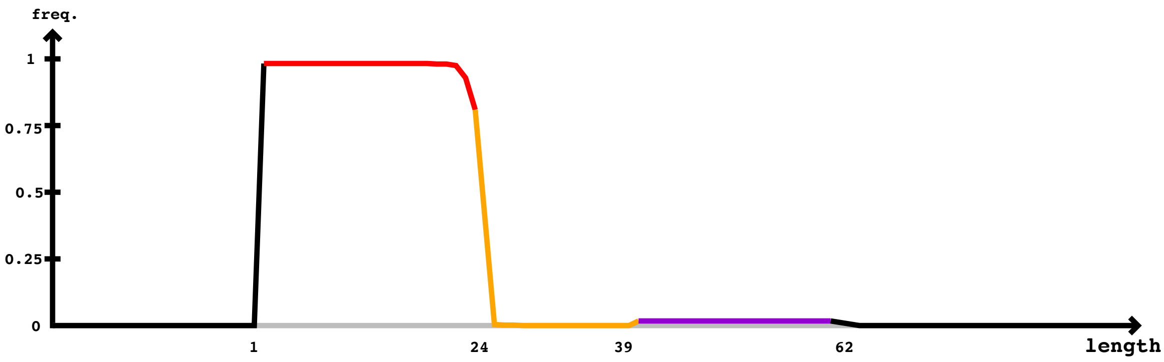

Mature Star

```
5'- auaaauucacucuaguguuuuauggcuuuuuauuccuaugugauaguaauaaagucucauguagggauggaagccaugaaauacauugugaaaaaucaucaacucagaugg  -3'   obs
    auaaauucacucuaguguuuuauggcuuuuuauuccuaugugauaguaauaaagucucauguagggauggaagccaugaaauacauugugaaaaaucaucaacucagaugg        exp
    .....((((....(((((((((((((((.(((((((((((.(.(.....).).))))))))))))))))))))))))))...)))))....((((......)))).   reads   mm   sample
    ....................uauggcuuuuuauuccua.........................................................................   1   0   seq
    ....................uauggcuuuuuauuccuaug.......................................................................   3   0   seq
    ....................uauggcuuuuuauuccuaugu......................................................................   24   0   seq
    ....................uauggcuuuuuauuccuaugug.....................................................................   59   0   seq
    ....................uauggcuuuuuauuccuauguU.....................................................................   1   1   seq
    ....................uauggcuuuuuauuccuauguA.....................................................................   2   1   seq
    ....................uauggcuuuuuauuccuaugugU....................................................................   41   1   seq
    ....................uauggcuuuuuauuccuaCguga....................................................................   1   1   seq
    ....................uauggcuuuuuauuccuauguAa....................................................................   2   1   seq
    ....................uauggcuuuuuauuccuaugugC....................................................................   1   1   seq
    ....................uauggcuuuuuauucUuauguga....................................................................   3   1   seq
    ....................uauggcuuuuuauuccuauguga....................................................................   316   0   seq
    ....................uauggcuuuuuauuccuaugugG....................................................................   25   1   seq
    ....................uauggcuuuuuauuccuaugugaC...................................................................   3   1   seq
    ....................uauggcuuuuuauuccuaugugaA...................................................................   9   1   seq
    ....................uauggcuuuuuauuccuaugugau...................................................................   16   0   seq
    ....................uauggcuuuuuauuccuaugugUu...................................................................   1   1   seq
    ....................uauggcuuuuuauuccuaugugaAa..................................................................   1   1   seq
    ....................uauggcuuuuuauuccuaugugauUgu................................................................   1   1   seq
    ..........................................................uguagggauggaagccaugaaa...............................   3   0   seq
    ..........................................................uguagggauggaagccaugaaU...............................   5   1   seq
    ..........................................................uguagggauggaagccaugaaau..............................   1   0   seq
```

Provisional ID	:	mun-miR-29a-2
Score total	:	4.7
Score for star read(s)	:	-1.3
Score for read counts	:	0
Score for mfe	:	1.4
Score for randfold	:	1.6
Score for cons. seed	:	3
Total read count	:	4282
Mature read count	:	4273
Loop read count	:	0
Star read count	:	9

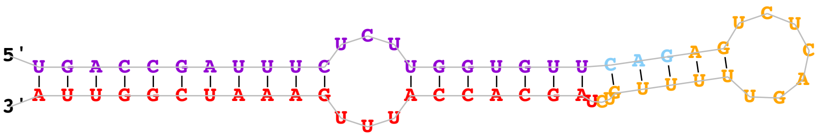

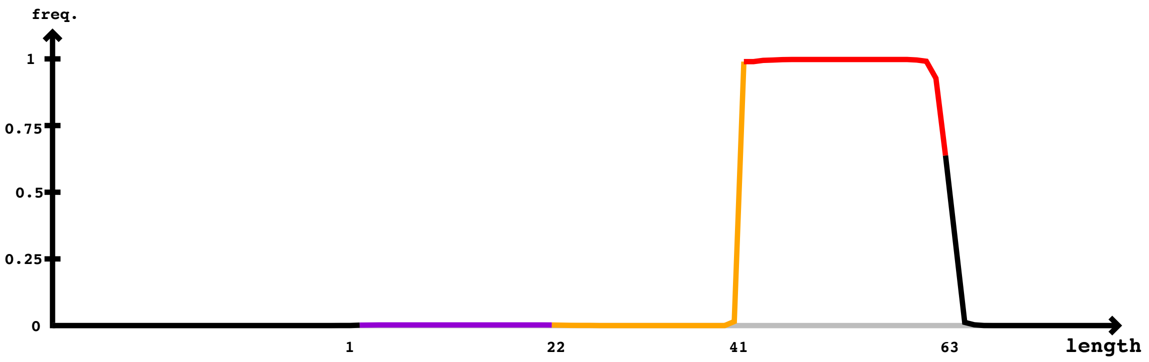

Star Mature

```
5'- cugagccaggaaaaugucucuuacacaggcugaccgauuucucuugguguucagagucucaguuuuugucuagcaccauuugaaaucgguuaugauguagggggaaaag  -3'   obs
    cugagccaggaaaaugucucuuacacaggcugaccgauuucucuugguguucagagucucaguuuuugucuagcaccauuugaaaucgguuaugauguagggggaaaag         exp
    ................(((((((((((...(((((((((((...((((((((((((.......)))))...)))))))...))))))))))))).)))))))))).....   reads   mm   sample
    ............................gcugaccgauuucucuuggugu...........................................................   1       0    seq
    ..............................ugaccgauuucucuugguguu..........................................................   3       0    seq
    ..............................ugaccgauuucucuugguguuA.........................................................   1       1    seq
    ..............................ugaccgauuucucuugguguuU.........................................................   1       1    seq
    ..............................ugaccgauuucucuugguguucU........................................................   1       1    seq
    ...............................gaccgauuucucuugguguu..........................................................   1       0    seq
    ................................accgauuucucuugguguucaga......................................................   2       0    seq
    .....................................................................cuagcaccauuugaaaucgg....................   4       0    seq
    .....................................................................cuagcaccauuugaaaucggu...................   5       0    seq
    .....................................................................cuagcaccauuugaaaucgguu..................   38      0    seq
    .....................................................................cuagcaccauuugaaaucAguu..................   3       1    seq
    .....................................................................cuagcaccauuugaaaucgguua.................   5       0    seq
    .....................................................................cuagcaccauuugaaaucgguuU.................   2       1    seq
    .....................................................................cuagcaccauuugaaaucgguAa.................   1       1    seq
    .....................................................................cuagcaccauuugaaaucgguuC.................   2       1    seq
    .....................................................................Guagcaccauuugaaaucgguua.................   1       1    seq
    .....................................................................cuagcaccauuugaaaucgguuGu................   2       1    seq
    .....................................................................cuagcaccauuugaaaucgguuaC................   1       1    seq
    .....................................................................cuagcaccauuugaaaucgguuau................   1       0    seq
    ......................................................................uagcaccauuugaaaucg.....................   8       0    seq
    ......................................................................uagcaccauuugaaaucgg....................   12      0    seq
    ......................................................................uagcaccauuugaaaucgA....................   2       1    seq
    ......................................................................uagcaccauuugaaaucgU....................   2       1    seq
    ......................................................................uagcaccauuugaaaucggu...................   245     0    seq
    ......................................................................uagcaccauuugaaaucggA...................   23      1    seq
    ......................................................................uagcaccauuugaaaucUguu..................   1       1    seq
    ......................................................................uagcaccauuugaaaCcgguu..................   1       1    seq
    ......................................................................uagcGccauuugaaaucgguu..................   1       1    seq
    ......................................................................uagcaccauuugaaaucggAu..................   4       1    seq
    ......................................................................Cagcaccauuugaaaucgguu..................   1       1    seq
    ......................................................................uagcaccauuugaaaucAguu..................   3       1    seq
    ......................................................................uagcaccauuugaaaucgguu..................   1171    0    seq
    ......................................................................uaUcaccauuugaaaucgguu..................   1       1    seq
```

Star **Mature**

cugagccaggaaaaugucucuuacacaggcugaccgauuucucuugguguucagagucucaguuuuugucuagcaccauuugaaaucgguuaugauguagggggaaaag			
....uagcaccauuugaaaucggGu..................	1	1	seq
....uagcacUauuugaaaucgguu..................	1	1	seq
....uagcaccauuugaaaucgguG..................	3	1	seq
....uagcaccauuugaaaucgguA..................	12	1	seq
....uagcaccauuugaaaucgCuu..................	1	1	seq
....Gagcaccauuugaaaucgguua.................	1	1	seq
....uagcaccauuugaaaucUguua.................	1	1	seq
....uagcaccauuugaaaucgguuC.................	27	1	seq
....uagcaccauuugaaaucgguua.................	1344	0	seq
....uagcaccauuugaaaucgguCa.................	1	1	seq
....uagcaccaCuugaaaucgguua.................	1	1	seq
....uagcaccauuugaaGucgguua.................	1	1	seq
....uagcacUauuugaaaucgguua.................	1	1	seq
....Aagcaccauuugaaaucgguua.................	1	1	seq
....uagUaccauuugaaaucgguua.................	1	1	seq
....uagcaccauCugaaaucgguua.................	1	1	seq
....uGgcaccauuugaaaucgguua.................	1	1	seq
....uagcaccauuugaaaucgguuU.................	97	1	seq
....uagcaccauuugaaaucgguuG.................	52	1	seq
....uagcaccauuugaaaucgguAa.................	85	1	seq
....uagcGccauuugaaaucgguua.................	1	1	seq
....uagcacUauuugaaaucgguuau................	1	1	seq
....uagcaccauuugaaaucgguuUu................	25	1	seq
....Gagcaccauuugaaaucgguuau................	1	1	seq
....uagcaccauuugaaaucgguuGu................	33	1	seq
....uagcaccauuugaaaucggGuau................	1	1	seq
....uagcaccauuugaaaucgguuaA................	488	1	seq
....uagcaccauuugaaaucgguuCu................	3	1	seq
....uagcaccauuugaaaucgguuaC................	109	1	seq
....uagcaccauuugaaaucgguuau................	346	0	seq
....uagcaccauuugaaaucgguAau................	5	1	seq
....uagcaccauuugaaaucgguuaG................	24	1	seq
....uagcaUcauuugaaaucgguuau................	1	1	seq
....uagcaccauuugaaaucgguuaAg...............	5	1	seq
....uagcaccauuugaaaucgguuauU...............	7	1	seq
....uagcaccauuugaaaucgguuaug...............	10	0	seq
....uagcaccauuugaaaucgguuaugU..............	5	1	seq
....uagcaccauuugaaaucgguuaGgau.............	1	1	seq
....gcaccauuugaaaucgguuau................	1	0	seq
....gcaccauuugaaaucgguuaug...............	15	0	seq
....gcaccauuGgaaaucgguuaug...............	1	1	seq
....gcaccauuugaaaucgguuaugU..............	2	1	seq
....gcaccauuugaaaucgguuauga..............	1	0	seq
....caccauuugaaaucgguua.................	1	0	seq
....caccauuugaaaucgguuauU...............	1	1	seq
....caccauuugaaaucgguuaugU..............	3	1	seq
....accauuugaaaucgguua.................	4	0	seq
....accauuugaaaucgguuU.................	1	1	seq
....accauuugaaaucgguuaA................	2	1	seq
....ccauuugaaaucgguuUu................	2	1	seq

Provisional ID	:	mun-novel81
Score total	:	0.3
Score for star read(s)	:	3.9
Score for read counts	:	-1.8
Score for mfe	:	1.1
Score for randfold	:	-2.2
Score for cons. seed	:	-0.6
Total read count	:	8
Mature read count	:	5
Loop read count	:	0
Star read count	:	3

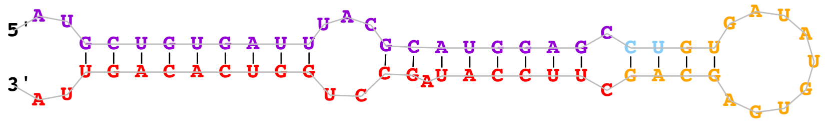

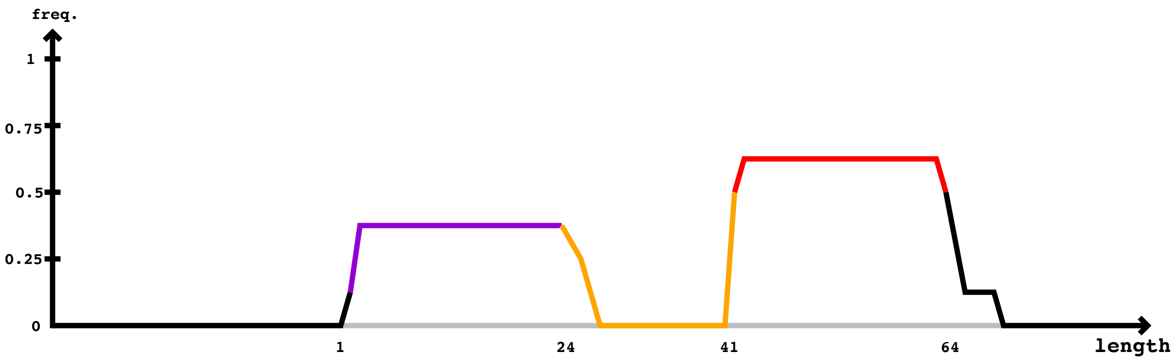

Star **Mature**

```
5'- cuucaguugguaggaaugggccgaaagugaugcugugauuuacgcauggagccugugauaugugagcagcuuccauagccuggucacaguuacagaccaaaccuugaaaccu   -3'    obs
    cuucaguugguaggaaugggccgaaagugaugcugugauuuacgcauggagccugugauaugugagcagcuuccauagccuggucacaguuacagaccaaaccuugaaaccu          exp
    .((((((((((.((......))....(((..((((((((...((((((((.((((.........)))).)))))).))..))))))))))))..)))))...)))))....   reads    mm      sample
    .............................augcugugauuuacgcauggagc............................................................   1        0       seq
    ..............................ugcugugauuuacgcauggagccu..........................................................   1        0       seq
    ..............................ugcugugauuuacgcauggagccuU.........................................................   1        1       seq
    .....................................................................cuuccauagccuggucacaguu.....................   1        0       seq
    .....................................................................cuuccauagccuggucacaguuU....................   3        1       seq
    ......................................................................uuccauagccuggucacaguuacagac...............   1        0       seq
```

Provisional ID	:	mun-novel4
Score total	:	486
Score for star read(s)	:	3.9
Score for read counts	:	475.3
Score for mfe	:	2.2
Score for randfold	:	1.6
Score for cons. seed	:	3
Total read count	:	944
Mature read count	:	926
Loop read count	:	0
Star read count	:	18

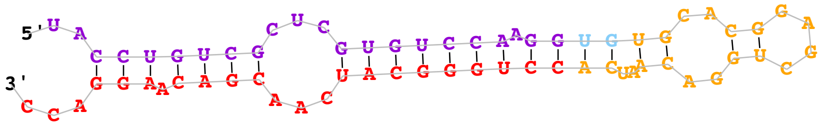

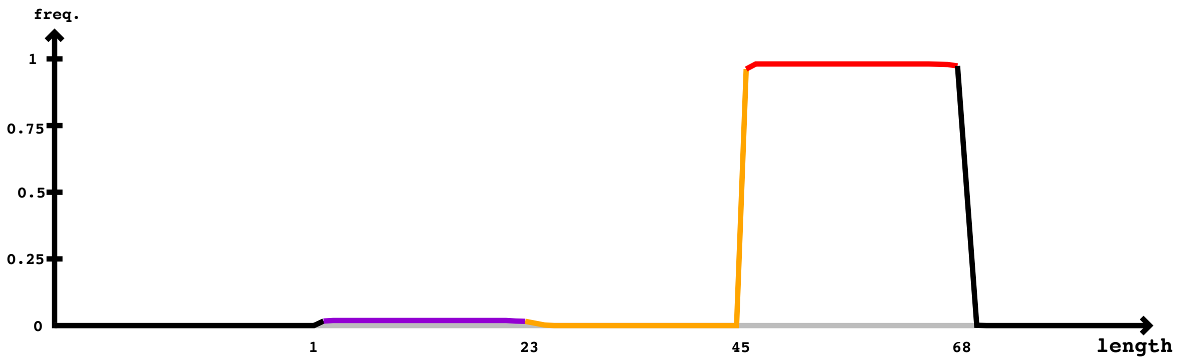


```
                          Star                                       Mature
5'- cgguggaggagcucgcucagcucgaguaccugucgcucguguccaaggugugcacggagcuggacaaucaccugggcaucaacgacaaggaccugggugagcggcuaucacc  -3'   obs
    cgguggaggagcucgcucagcucgaguaccugucgcucguguccaaggugugcacggagcuggacaaucaccugggcaucaacgacaaggaccugggugagcggcuaucacc        exp
    .((((...(((.((((((.((((.((.(((((((...(((((((.((((((..((....))..))..))))))))))...)))).))))).)))))))))))))).)))))   reads   mm   sample
    ..........................uaccugucgcucguguccaU..................................................................   2       1    seq
    ..........................uaccugucgcucguguccaaU.................................................................   1       1    seq
    ..........................uaccugucgcucgugCccaagg................................................................   1       1    seq
    ..........................uaccugucgcucguguccaagA................................................................   2       1    seq
    ..........................uaccugucgcucguguccaagU................................................................   5       1    seq
    ..........................uaccugucgcucgGguccaagg................................................................   1       1    seq
    ..........................uaccugucgcucguguccaaggC...............................................................   1       1    seq
    ..........................uaccugucgcucguguccaaggA...............................................................   2       1    seq
    ..........................uaccugucgcucguguccaagguU..............................................................   1       1    seq
    ...........................accugucgcucguguccaagU................................................................   1       1    seq
    ...........................accugucgcucguguccaaggGg..............................................................   1       1    seq
    ......................................................................ccugggcaucaacgacaGgg......................   1       1    seq
    ......................................................................ccugggcaucCacgacaagga.....................   1       1    seq
    ......................................................................ccugggcaucaacgacaaggaU....................   2       1    seq
    ......................................................................Acugggcaucaacgacaaggac....................   1       1    seq
    ......................................................................ccugggcaucaacgacaaggacG...................   4       1    seq
    ......................................................................ccugggcaucaacgacaaggacA...................   10      1    seq
    ......................................................................ccugggcaucaacgacaaggacU...................   875     1    seq
    ......................................................................ccugggcaucaacgacaaggacUu..................   13      1    seq
    ......................................................................ccugggcaucaacgacaaggacAu..................   1       1    seq
    .......................................................................cugggcaucaacgacaaggaA....................   2       1    seq
    .......................................................................cugggcaucaacgacaaggacU...................   14      1    seq
    .......................................................................cugggcaucaacgacaaggaccA..................   1       1    seq
    .......................................................................cugggcaucaacgacaaggaccuU.................   1       1    seq
```

Provisional ID	:	mun-novel26
Score total	:	23.2
Score for star read(s)	:	3.9
Score for read counts	:	12.9
Score for mfe	:	1.9
Score for randfold	:	1.6
Score for cons. seed	:	3
Total read count	:	37
Mature read count	:	35
Loop read count	:	0
Star read count	:	2

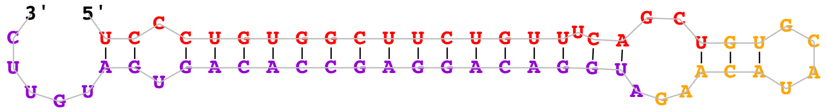

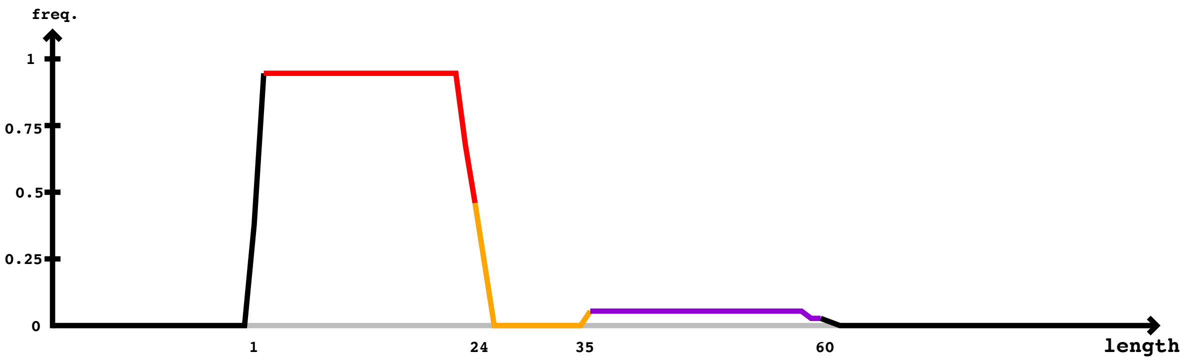

Mature Star

```
5'- auggucaugcccugggaaccucccuguggcuucuguuucagcugugcauacaagauggacaggagccacagugauguucagaggcaaucucugcucccuguaucccaaugcag  -3'  obs
    auggucaugcccugggaaccucccuguggcuucuguuucagcugugcauacaagauggacaggagccacagugauguucagaggcaaucucugcucccuguaucccaaugcag       exp
    .......(((..(((((...((.((((((((((((((.((..(((....)))...)))))))))))))))).))....(((.((((.....))))..)))..)))))..))).   reads  mm  sample
    ...................cucccuguggcuucuguuucag........................................................................   8      0   seq
    ...................cucccuguggcuucuguuucagc.......................................................................   3      0   seq
    ...................cucccuguggcuucuguuucagcu......................................................................   3      0   seq
    ....................Ccccuguggcuucuguuucag........................................................................   1      1   seq
    ....................ucccuguggcuucuguuucag........................................................................   1      0   seq
    ....................ucccuguggcuucuguuucagU.......................................................................   1      1   seq
    ....................ucccuguggcuucuguuucagc.......................................................................   4      0   seq
    ....................ucccuguggcuucuguuucagcu......................................................................   14     0   seq
    ......................................................auggacaggagccacagugaugu....................................   1      0   seq
    ......................................................auggacaggagccacagugauguuU..................................   1      1   seq
```

Provisional ID : mun-novel85
Score total : 1.8
Score for star read(s) : -1.3
Score for read counts : 0
Score for mfe : 2.1
Score for randfold : 1.6
Score for cons. seed : -0.6
Total read count : 1218
Mature read count : 1218
Loop read count : 0
Star read count : 0

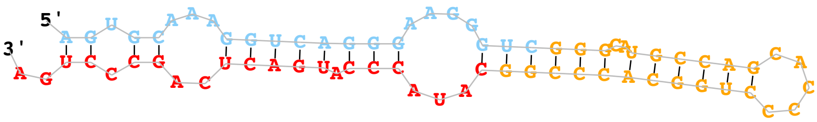

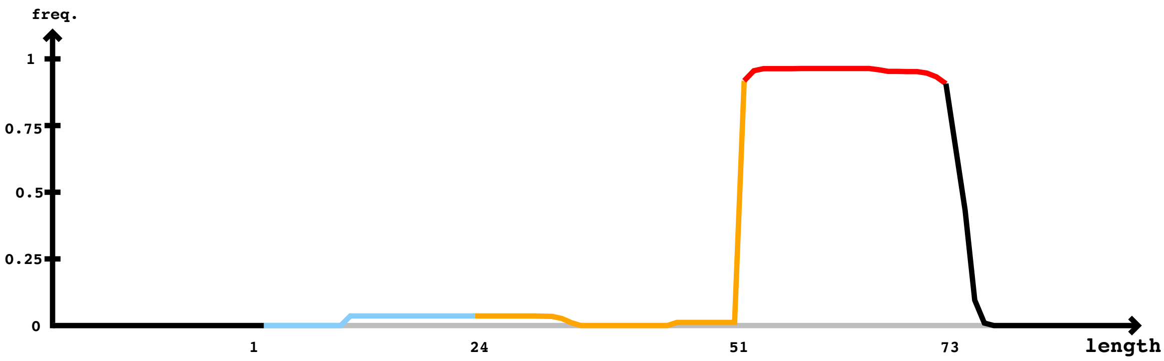

Star Mature

```
5'- uuccugguggccucgcagacagugcaaaggucagggaaggguc gggcaugccagcaccccuggcacccggcauacccaugacucagcccugagcucggccucuccuccugu -3'   exp
    .....((.((((..((...(((.((...((((((((....((((((..((((((.....))))))))))))...))).)))))..)).))).))..))))...))......   reads  mm  sample
    .............................gucagggaagggucgggcau..............................................................   1      0   seq
    .............................gucagggaagggucgggcaug.............................................................   1      0   seq
    .............................gucagggaagggucgggcaugU............................................................   4      1   seq
    .............................gucagggaagggucgggcaugc............................................................   6      0   seq
    .............................gucagggaagggucgggcaugcU...........................................................   2      1   seq
    .............................gucagggaagggucgggcaugcc...........................................................   18     0   seq
    .............................gucagggaagggucgggcaugcca..........................................................   3      0   seq
    .............................gucagggaagggucgggcaugccG..........................................................   1      1   seq
    .............................gucagggaagggucgggcaugccU..........................................................   10     1   seq
    ...............................................................cacccggcauacccaugacuU...........................   1      1   seq
    ...............................................................cacccggcauacccaugacuc...........................   5      0   seq
    ...............................................................cacccggcauacccaugacuca..........................   4      0   seq
    ...............................................................cacccggcauacccaugacucU..........................   4      1   seq
    ...............................................................cacccggcauacccaugacucagc........................   1      0   seq
    ......................................................................cauacccaugacucagccc......................   7      0   seq
    ......................................................................cauacccaugacucagcccu.....................   17     0   seq
    ......................................................................cauacccaugacucagcccug....................   33     0   seq
    ......................................................................cauacccaugacucagcccugU...................   2      1   seq
    ......................................................................cauacccaugacucagcccuga...................   459    0   seq
    ......................................................................Gauacccaugacucagcccuga...................   1      1   seq
    ......................................................................cauacccaugacucagcccCga...................   1      1   seq
    ......................................................................cauacccaugacuUagcccuga...................   3      1   seq
    ......................................................................cauacccaugacucagAccuga...................   1      1   seq
    ......................................................................cauacccaugacucagcccugG...................   3      1   seq
    ......................................................................cauaccUaugacucagcccuga...................   2      1   seq
    ......................................................................cauacccaugacucagcccugaU..................   4      1   seq
    ......................................................................cauacccaugacucagcccugaA..................   6      1   seq
    ......................................................................cauacccaugacucagcccugag..................   113    0   seq
    ......................................................................cauacccaugacucGgcccugagc.................   2      1   seq
    ......................................................................cauacccaugacucagccGugagc.................   1      1   seq
    ......................................................................cauacccaugacucagcccugagU.................   1      1   seq
    ......................................................................cauacccaugacucagcccugagA.................   39     1   seq
    ......................................................................cauacccaugUcucagcccugagc.................   1      1   seq
    ......................................................................Aauacccaugacucagcccugagc.................   2      1   seq
```

```
                         Star                                                  Mature
uuccugguggccucgcagacagugcaaaggucagggaaggguc gggcaugccagcaccccuggcacccggcauacccaugacucagcccugagcucggccucuccuccugu
......................................................................cauacccaugacucagcccugagc.................	356	0	seq
......................................................................cauacccaugacucagcccugagcu................	93	0	seq
......................................................................cauacccaugacucagcccugagcA................	1	1	seq
......................................................................cauacccaugacucagcccugagcuU...............	9	1	seq
......................................................................cauacccaugacucagcccugagcuA...............	2	1	seq
.......................................................................auacccaugacucagcccu.....................	1	0	seq
.......................................................................auacccaugacucagcccuga...................	6	0	seq
.......................................................................aCacccaugacucagcccuga...................	1	1	seq
.......................................................................auacccaugacucagcccugag..................	2	0	seq
.......................................................................auacccaugacucagcccugagA.................	6	1	seq
.......................................................................auacccaugacucagcccugagc.................	19	0	seq
.......................................................................auacccaugacucagcccugagcA................	1	1	seq
.......................................................................auacccaugacucagcccugagcu................	12	0	seq
........................................................................uacccaugacucagcccuga...................	4	0	seq
........................................................................uacccaugacucagcccugagc.................	2	0	seq
........................................................................uacccaugacucagcccugagAu................	1	1	seq
........................................................................uacccaugacucagcccugagcu................	2	0	seq
........................................................................uacccaugacucagcccugagcuU...............	1	1	seq
............................................................................caugacucagcccugagc.................	1	0	seq
```

Provisional ID	:	mun-miR-1416
Score total	:	39.4
Score for star read(s)	:	3.9
Score for read counts	:	29.7
Score for mfe	:	1.2
Score for randfold	:	1.6
Score for cons. seed	:	3
Total read count	:	70
Mature read count	:	69
Loop read count	:	0
Star read count	:	1

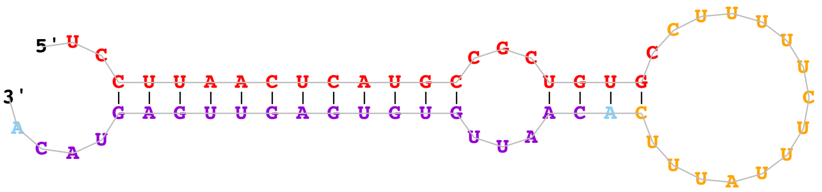

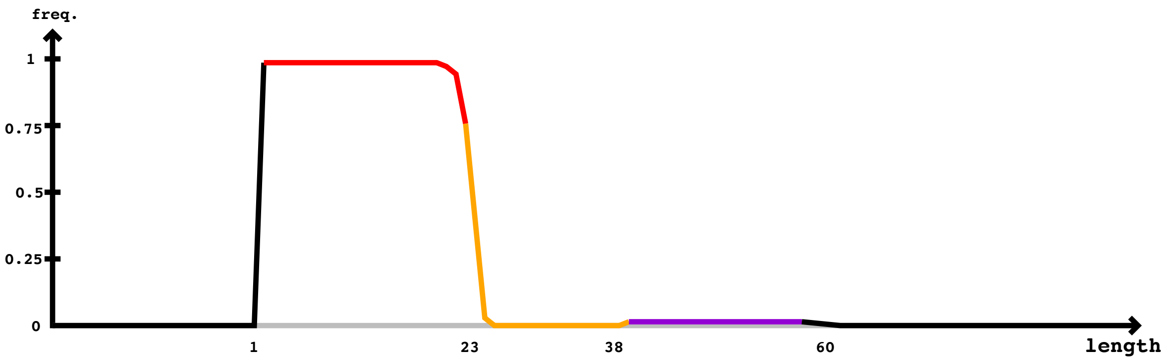

Mature **Star**

```
5'- agagcagggcuaauccucuuuccuuaacucaugccgcugugccuuuuucuuuauuucacaauugugugaguugaguacagagugucagcccggcccaguaucaccugcagc   -3'    obs
    agagcagggcuaauccucuuuccuuaacucaugccgcugugccuuuuucuuuauuucacaauugugugaguugaguacagagugucagcccggcccaguaucaccugcagc          exp
    .(.((.(((((.(..((((...((((((((((((...((((...............))))...))))))))))))...))))..).))))).)).)...............   reads   mm   sample
    ....................uccuuaacucaugccgcug........................................................................   1       0    seq
    ....................uccuuaacucaugccgcugu.......................................................................   2       0    seq
    ....................uccuuaacucaugccgcugug......................................................................   13      0    seq
    ....................uccuuaacucaugccgcugugc.....................................................................   20      0    seq
    ....................uccuuaacucaugccgcugugU....................................................................   17      1    seq
    ....................uccuuaacucaugccgcugugA....................................................................   11      1    seq
    ....................uccuuaacucaugccgcugugG....................................................................   3       1    seq
    ....................uccuuaacucaugccgcugugccu..................................................................   2       0    seq
    ..........................................................caauugugugaguugaguac.................................   1       0    seq
```

Provisional ID	:	mun-miR-490
Score total	:	57
Score for star read(s)	:	3.9
Score for read counts	:	46.5
Score for mfe	:	2
Score for randfold	:	1.6
Score for cons. seed	:	3
Total read count	:	103
Mature read count	:	100
Loop read count	:	0
Star read count	:	3

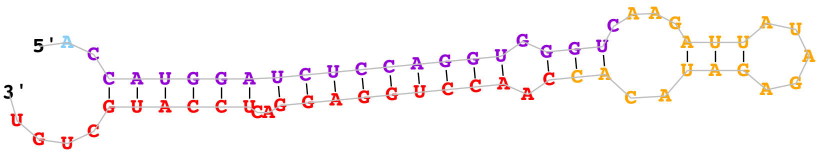

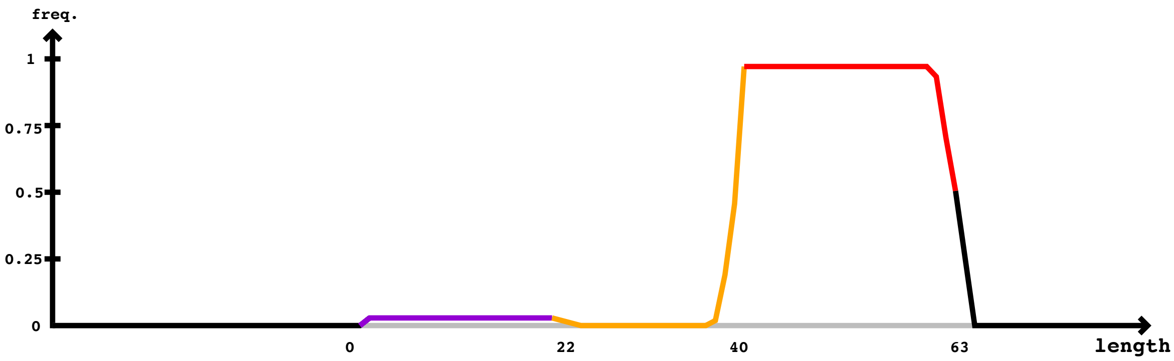

Star **Mature**

```
5'- agugcugauuucauaaguucaugguucgacaccauggaucuccaggugggucaagauuauagagauacaccaaccuggaggacuccaugcuguugagcuguucauaugcagc   -3'    obs
    agugcugauuucauaaguucaugguucgacaccauggaucuccaggugggucaagauuauagagauacaccaaccuggaggacuccaugcuguugagcuguucauaugcagc          exp
    ...((((....((((.(...(((((((((((.(((((((((((((((.(((....(((.....)))..))).)))))))))..)))))).))))))))))).).))))))))    reads    mm    sample
    ...............................ccauggaucuccaggugggu.............................................................    1        0     seq
    ...............................ccauggaucuccaggugggguc............................................................    2        0     seq
    ...................................................................caccaaccuggaggacuccaugcu.....................    2        0     seq
    ....................................................................accaaccuggaggacuccaugA......................    1        1     seq
    ....................................................................accaaccuggaggacuccaugc......................    2        0     seq
    ....................................................................accaaccuggaggacuccaugcu.....................    14       0     seq
    ....................................................................accaaccuggaggacuccaugcug....................    1        0     seq
    .....................................................................ccaaccuggaggacuccaugc......................    1        0     seq
    .....................................................................ccaaccuggaggacuccaugcu.....................    7        0     seq
    .....................................................................ccaaccuggaggacuccaugcug....................    11       0     seq
    .....................................................................ccaaccuggaggacuccaugcugu...................    9        0     seq
    ......................................................................caaccuggaggacuccaugcu.....................    1        0     seq
    ......................................................................caaccuggaggacuccaugcug....................    9        0     seq
    ......................................................................caaccuggaggacuccaugcugu...................    41       0     seq
    ......................................................................Aaaccuggaggacuccaugcugu...................    1        1     seq
    ......................................................................caaccuggaggacuccaugcuguu..................    2        0     seq
```

Provisional ID	:	mun-miR-137
Score total	:	5.4
Score for star read(s)	:	-1.3
Score for read counts	:	0
Score for mfe	:	2.1
Score for randfold	:	1.6
Score for cons. seed	:	3
Total read count	:	127
Mature read count	:	124
Loop read count	:	0
Star read count	:	3

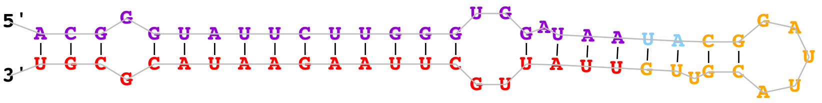

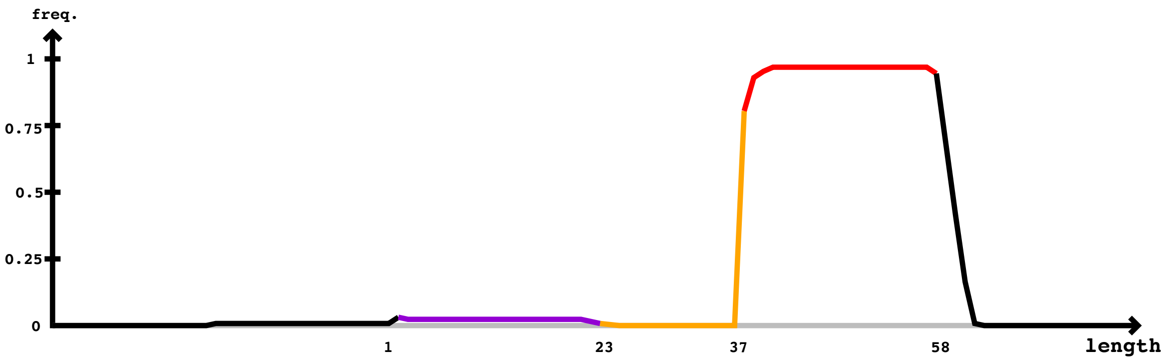

Star Mature

```
5'- agcaacagcagcagcuuucugacucucuucggugacggguauucuuggguggauaauacggauuacguuguuauugcuuaagaauacgcguagucgaggagaguaccggcg   -3'   obs
    agcaacagcagcagcuuucugacucucuucggugacggguauucuuggguggauaauacggauuacguuguuauugcuuaagaauacgcguagucgaggagaguaccggcg         exp
    .((....))....(((.....(((((((((((..(((.(((((((((((..(.(((((((.....)).))))))..))))))))))).)))..)))))))))))...))).   reads   mm   sample
    ...............uuucugacucucuucggugU............................................................................   1   1   seq
    ..................................acggguauucuuggguggau.........................................................   1   0   seq
    ..................................acggguauucuuggguggaua........................................................   1   0   seq
    ..................................acggguauucuuggguggauaa.......................................................   1   0   seq
    ......................................................................uuauugcuuaagaauacgcg.....................   2   0   seq
    ......................................................................uuauugcuuaagaauacgcU.....................   1   1   seq
    ......................................................................uCauugcuuaagaauacgcgu....................   1   1   seq
    ......................................................................uuauugcuuaagaauacgcgu....................   50   0   seq
    ......................................................................uuauugcuuaagaauacgcguU...................   2   1   seq
    ......................................................................uuauugcuuaagaauacgcgua...................   14   0   seq
    ......................................................................uuauugcuuaagaauacgcguaA..................   1   1   seq
    ......................................................................uuauugcuuaagaauacgcguaC..................   2   1   seq
    ......................................................................uuauugcuuaagaauacgcguag..................   19   0   seq
    ......................................................................uuauugcuuaagaauacgcguaU..................   5   1   seq
    ......................................................................uuauugcuuaagaauacgcguagu.................   6   0   seq
    .......................................................................uauugcuuaagaauacgcgu....................   1   0   seq
    .......................................................................uauugcuuaagaauacgcguaA..................   1   1   seq
    .......................................................................uauugcuuaagaauacgcguag..................   4   0   seq
    .......................................................................uauugcuuaagaauacgcguagu.................   9   0   seq
    .......................................................................uauugcuuaagaauacgcguagA.................   1   1   seq
    ........................................................................auugcuuaagaauacgcguaCu.................   1   1   seq
    ........................................................................auugcuuaagaauacgcguagA.................   2   1   seq
    .........................................................................uugcuuaagaauacgcguagu.................   1   0   seq
    .........................................................................uugcuuaagaauacgcguaguU................   1   1   seq
```

Provisional ID	:	mun-novel9
Score total	:	1.8
Score for star read(s)	:	-1.3
Score for read counts	:	0
Score for mfe	:	2.1
Score for randfold	:	1.6
Score for cons. seed	:	-0.6
Total read count	:	27
Mature read count	:	27
Loop read count	:	0
Star read count	:	0

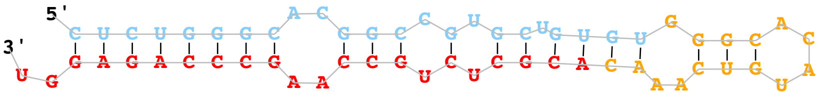

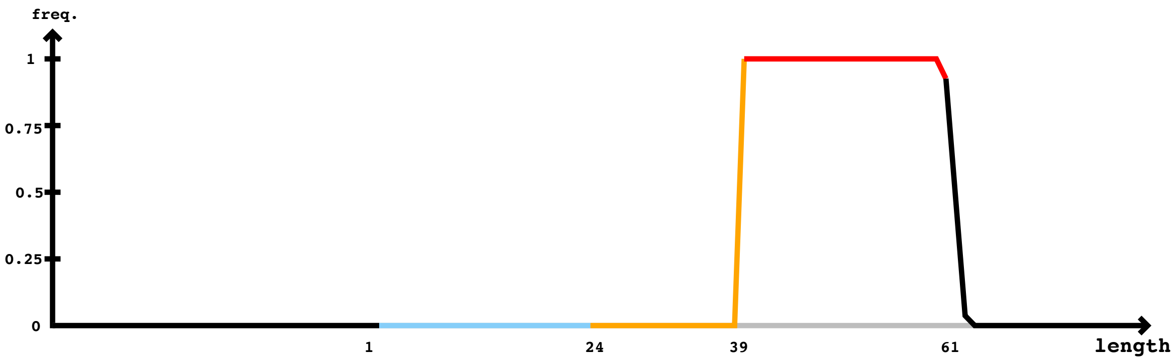

Star Mature

```
5'- acuacuggcacuuacagcagaaccugccauggcucugggcacggccgugcuguguggcacaugucaaacacgcucugccaagcccagagguauguuggggugaggcagca   -3'    exp
    .......((.(((((.......(((..((((.((((((((..(((.(.((.((((.(((....)))..)))))).).)))..)))))))).))))..))).)))))))....    reads      mm         sample
    ......................................................................acgcucugccaagcccagagg....................    2          0          seq
    ......................................................................acgcucugccaagcccagaggu...................    24         0          seq
    ......................................................................acgcucugccaagcccagagguUu.................    1          1          seq
```

Provisional ID	:	mun-miR-31-1
Score total	:	108.4
Score for star read(s)	:	3.9
Score for read counts	:	98
Score for mfe	:	1.9
Score for randfold	:	1.6
Score for cons. seed	:	3
Total read count	:	204
Mature read count	:	201
Loop read count	:	0
Star read count	:	3

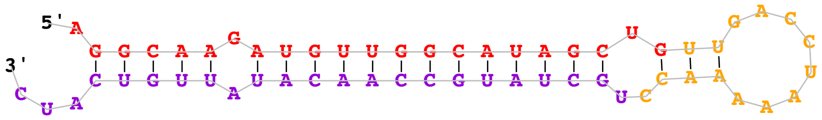

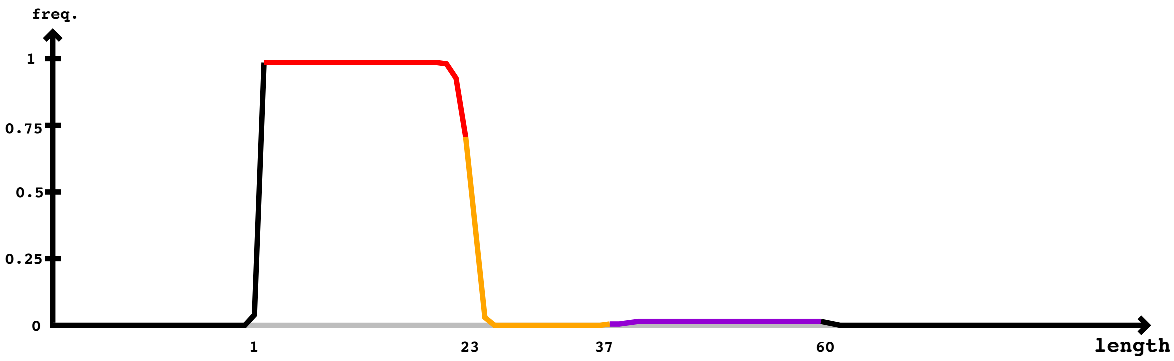


```
                      Mature                            Star
5'- uuuaugcagagcuggagggcaggcaagauguuggcauagcuguugaccuaaaaaaccugcuaugccaacauauugucaucuuuccuuucuguuugcggcagagggaaauucaa  -3'   obs
    uuuaugcagagcuggagggcaggcaagauguuggcauagcuguugaccuaaaaaaccugcuaugccaacauauugucaucuuuccuuucuguuugcggcagagggaaauucaa         exp
    .....((((((..((((((..(((((.(((((((((((((.(((........)))..))))))))))))).))))).))))))..)))))).))))))........(((.....)))..   reads   mm   sample
    ...................caggcaagauguuggcauagc........................................................................   4       0    seq
    ...................caggcaagauguuggcauagcu.......................................................................   4       0    seq
    ....................aggcaagauguuggcauag.........................................................................   1       0    seq
    ....................aggcaagauguuggcauagc........................................................................   7       0    seq
    ....................aggcaagauguuggcauagcu.......................................................................   41      0    seq
    ....................aggcaagauguuggcauagcug......................................................................   67      0    seq
    ....................aggcaagauguuggcauagcuU......................................................................   2       1    seq
    ....................aggcaagaugCuggcauagcug......................................................................   1       1    seq
    ....................aggcaagauguuggcauagcugu.....................................................................   60      0    seq
    ....................aggcaagauguuggcauagcugA.....................................................................   8       1    seq
    ....................aggcaagauguuggcauagcuguA....................................................................   1       1    seq
    ....................aggcaagauguuggcauagcuguu....................................................................   5       0    seq
    .........................................................ugcuaugccaacauauugucauc................................   1       0    seq
    ...........................................................cuaugccaacauauugucaucu...............................   1       0    seq
    ............................................................uaugccaacauauugucaucu...............................   1       0    seq
```

Provisional ID	:	mun-novel41
Score total	:	1.7
Score for star read(s)	:	-1.3
Score for read counts	:	0
Score for mfe	:	2
Score for randfold	:	1.6
Score for cons. seed	:	-0.6
Total read count	:	55
Mature read count	:	55
Loop read count	:	0
Star read count	:	0

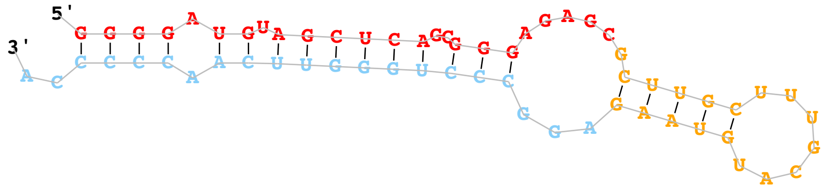

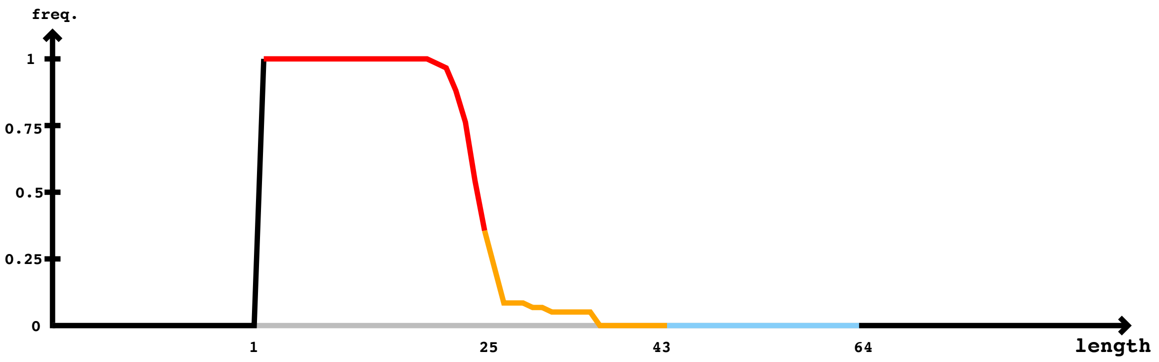

Mature **Star**

```
5'- gcgaggggcuguggaugcagggggauguagcucagcgggagagcgcuugcuuugcauguaagaggcccuggguucaacccccagcaucuccaguucnnnnnnnnnnnnnn   -3'   exp
    .....((((((..(((((.(((((.((.((((((..(((......(((((.......)))))...))))))))))).))))).))))).))))))..............   reads   mm   sample
    ....................ggggauguagcucagcgU........................................................................   1       1    seq
    ....................ggggauguagcucagcggg.......................................................................   1       0    seq
    ....................ggggauguagcucagcggga......................................................................   4       0    seq
    ....................ggggauguaAcucagcggga......................................................................   1       1    seq
    ....................ggggauguagcucagcgggag.....................................................................   7       0    seq
    ....................ggggauguagcucagcgggaga....................................................................   11      0    seq
    ....................ggggauguagcucagcgggagU....................................................................   2       1    seq
    ....................ggggauguagcucagcgggagag...................................................................   11      0    seq
    ....................ggggauguaAcucagcgggagagc..................................................................   1       1    seq
    ....................ggggauAuagcucagcgggagagc..................................................................   1       1    seq
    ....................ggggauguagcucagcgggagagc..................................................................   14      0    seq
    ....................ggggauAuagcucagcgggagagcgcuu..............................................................   1       1    seq
    ....................ggggauguagcucagcgggagagcgcuugc............................................................   1       0    seq
    ....................ggggauguagcucagcgggagagcgcuugcuuugc.......................................................   2       0    seq
    ....................ggggauguagcucagcgggagagcgcuugcuuCgc.......................................................   1       1    seq
```

Provisional ID : mun-miR-99
Score total : 12671.6
Score for star read(s) : 3.9
Score for read counts : 12661.1
Score for mfe : 2
Score for randfold : 1.6
Score for cons. seed : 3
Total read count : 24846
Mature read count : 24320
Loop read count : 0
Star read count : 526

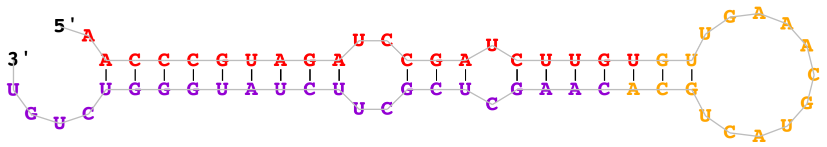

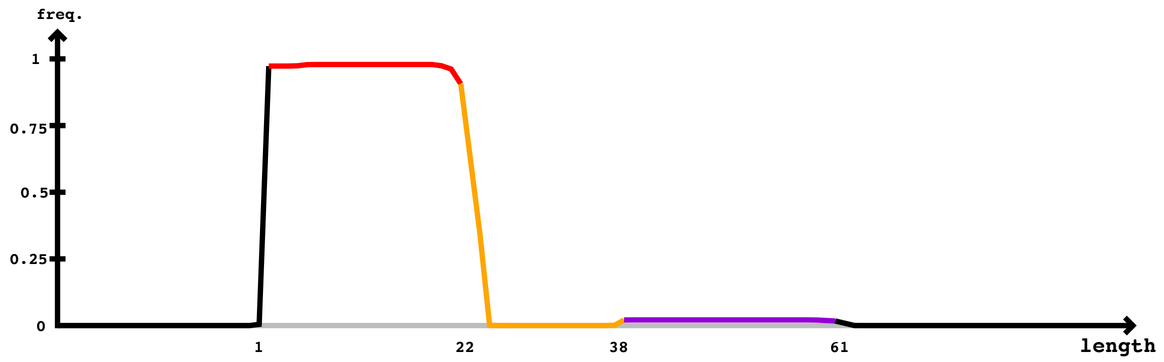

Mature Star

```
5'- cagcgaugccaauuggcauaaacccguagauccgaucuuguguugaaacguacugcacaagcucgcuucuaugggucugugucaguauggugaucuggcaaaaguuuaug -3' obs
    cagcgaugccaauuggcauaaacccguagauccgaucuuguguugaaacguacugcacaagcucgcuucuaugggucugugucaguauggugaucuggcaaaaguuuaug     exp
    ..((((((((((((((((((.(((((((((..(((.(((((((...........))))))).)))..))))))))).))))))))).)))).)))..))........... reads mm sample
    ...................aaacccguagauccgaucuug...................................................................... 7 0 seq
    ...................aGacccguagauccgaucuug...................................................................... 1 1 seq
    ...................aaacccguagauccgaucuugA..................................................................... 3 1 seq
    ...................Caacccguagauccgaucuugu..................................................................... 1 1 seq
    ...................aaacccguagauccgaucuugu..................................................................... 95 0 seq
    ...................aGacccguagauccgaucuugu..................................................................... 2 1 seq
    ...................aaacccguagauccgaucuugug.................................................................... 5 0 seq
    ...................aaacccguagauccgaucuugugA................................................................... 1 1 seq
    ....................Gacccguagauccgaucu........................................................................ 2 1 seq
    ....................aacccguagauccgaucA........................................................................ 2 1 seq
    ....................aacccguagauccgaucu........................................................................ 112 0 seq
    ....................aacccguagaucUgaucu........................................................................ 1 1 seq
    ....................aacccguagaucUgaucuu....................................................................... 6 1 seq
    ....................aacccguagauccgaucuu....................................................................... 294 0 seq
    ....................aacccgGagauccgaucuu....................................................................... 1 1 seq
    ....................aacccguagauccgGucuu....................................................................... 1 1 seq
    ....................aacccguagauUcgaucuu....................................................................... 1 1 seq
    ....................aacccguagauccgaucuG....................................................................... 2 1 seq
    ....................Gacccguagauccgaucuu....................................................................... 3 1 seq
    ....................aacccguagauccgaucuGg...................................................................... 1 1 seq
    ....................aacccUuagauccgaucuug...................................................................... 1 1 seq
    ....................aacccguagauccgaucuuA...................................................................... 5 1 seq
    ....................aacccAuagauccgaucuug...................................................................... 1 1 seq
    ....................aacccguagauccgaucuuU...................................................................... 2 1 seq
    ....................aacccguagauccgaucuug...................................................................... 1340 0 seq
    ....................aacccguagauccgaucuCg...................................................................... 1 1 seq
    ....................aacccguagaucGgaucuug...................................................................... 1 1 seq
    ....................aacccguagaucUgaucuug...................................................................... 7 1 seq
    ....................aacccguagauUcgaucuug...................................................................... 1 1 seq
    ....................Gacccguagauccgaucuug...................................................................... 16 1 seq
    ....................aacccguagauccgaGcuugu..................................................................... 4 1 seq
    ....................aacccguagauAcgaucuugu..................................................................... 3 1 seq
    ....................aacccguagauccgaucuugC..................................................................... 29 1 seq
```

Mature   Star

```
cagcgaugccaauuggcauaaacccguagauccgaucuuguguugaaacguacugcacaagcucgcuucuaugggucugugucaguauggugaucuggcaaaaguuuaug
....................aaccAguagauccgaucuugu.....................................................................  2     1  seq
....................aacAcguagauccgaucuugu.....................................................................  1     1  seq
....................aacccguagauccgCucuugu.....................................................................  1     1  seq
....................aacccguagauccgaucuugG.....................................................................  11    1  seq
....................aaccUguagauccgaucuugu.....................................................................  26    1  seq
....................aacccguagauccgaCcuugu.....................................................................  1     1  seq
....................Cacccguagauccgaucuugu.....................................................................  2     1  seq
....................aacccguagaucUgaucuugu.....................................................................  168   1  seq
....................aacccAuagauccgaucuugu.....................................................................  3     1  seq
....................aaUccguagauccgaucuugu.....................................................................  1     1  seq
....................aacccguagauccgaucuugA.....................................................................  182   1  seq
....................aacccguagauccgauGuugu.....................................................................  1     1  seq
....................aacccguagauccgUucuugu.....................................................................  2     1  seq
....................aacccguagauccgGucuugu.....................................................................  1     1  seq
....................aacccguagauccgaucuugu.....................................................................  9134  0  seq
....................aacccguagauccgaucAugu.....................................................................  1     1  seq
....................aacccguagaucGgaucuugu.....................................................................  2     1  seq
....................Uacccguagauccgaucuugu.....................................................................  3     1  seq
....................aacccguagauccgauAuugu.....................................................................  2     1  seq
....................aacccguagaCccgaucuugu.....................................................................  1     1  seq
....................aacccguaCauccgaucuugu.....................................................................  1     1  seq
....................Gacccguagauccgaucuugu.....................................................................  109   1  seq
....................aaAccguagauccgaucuugu.....................................................................  1     1  seq
....................aacccguagauccgauUuugu.....................................................................  6     1  seq
....................aacccUuagauccgaucuugu.....................................................................  2     1  seq
....................aacUcguagauccgaucuugu.....................................................................  3     1  seq
....................aacccguagauUcgaucuugu.....................................................................  5     1  seq
....................aGcccguagauccgaucuugu.....................................................................  3     1  seq
....................aacccguagauccgaucuuUu.....................................................................  5     1  seq
....................aacccgGagauccgaucuugu.....................................................................  1     1  seq
....................aacccguagauccCaucuugu.....................................................................  1     1  seq
....................aacccguagaucAgaucuugu.....................................................................  4     1  seq
....................aacccguagaCccgaucuugug....................................................................  1     1  seq
....................aacccguagauccgaucuuguA....................................................................  69    1  seq
....................aacccguagauccgUucuugug....................................................................  2     1  seq
....................aaccUguagauccgaucuugug....................................................................  1     1  seq
....................aacccguagauccgaucuuguC....................................................................  1     1  seq
....................aacccgAagauccgaucuugug....................................................................  1     1  seq
....................aacccAuagauccgaucuugug....................................................................  2     1  seq
....................aacccguagauccgaucuugug....................................................................  3944  0  seq
....................aacccguagauccgauUuugug....................................................................  1     1  seq
....................aacccguGgauccgaucuugug....................................................................  1     1  seq
....................aacccguagaucUgaucuugug....................................................................  23    1  seq
....................aacccguagauccUaucuugug....................................................................  2     1  seq
....................aacccguagGuccgaucuugug....................................................................  1     1  seq
....................aacccgCagauccgaucuugug....................................................................  3     1  seq
....................aacccguagauccgaucuuCug....................................................................  1     1  seq
....................aacccgGagauccgaucuugug....................................................................  1     1  seq
....................aacccguagauccgaucuuUug....................................................................  1     1  seq
....................aacccCuagauccgaucuugug....................................................................  1     1  seq
....................aacAcguagauccgaucuugug....................................................................  1     1  seq
....................aacccguagauccAaucuugug....................................................................  1     1  seq
....................aacccguagauccgaucuuguU....................................................................  12    1  seq
....................aGcccguagauccgaucuugug....................................................................  3     1  seq
....................aacccguagauccgaucuugGg....................................................................  7     1  seq
....................Gacccguagauccgaucuugug....................................................................  25    1  seq
....................aacccguagauccgaucuugAg....................................................................  1     1  seq
....................aacccguagauccgaCcuugug....................................................................  2     1  seq
....................aacccguagauccgaucuugCg....................................................................  11    1  seq
....................Cacccguagauccgaucuugug....................................................................  1     1  seq
....................aacccguagauccgaucuugugG...................................................................  624   1  seq
....................aacccguagaucUgaucuugugu...................................................................  1     1  seq
....................aacccguagauUcgaucuugugu...................................................................  1     1  seq
....................aacccguagauccgaucuugugu...................................................................  101   0  seq
....................aacccguagauccgaucuugugA...................................................................  1457  1  seq
....................aacccguagauccgaucuuguUu...................................................................  2     1  seq
....................aacccguagaucAgaucuugugu...................................................................  1     1  seq
....................aacccguagauccgaAcuugugu...................................................................  6203  1  seq
....................aacccguagauccgaucuuguAu...................................................................  3     1  seq
....................aacccgCagauccgaucuugugu...................................................................  1     1  seq
```

Mature **Star**

```
cagcgaugccaauuggcauaaacccguagauccgaucuuguguugaaacguacugcacaagcucgcuucuaugggucugugucaguauggugaucuggcaaaaguuuaug
....................aacccguagauccgaucuugugC...................................................................  3    1  seq
....................aaccUguagauccgaucuugugu....................................................................  2    1  seq
....................aacccguagauccgaAcuuguguu..................................................................  9    1  seq
....................aacccguagauccgaucuugugGu..................................................................  1    1  seq
....................aacccguagauccgaucuuguguA..................................................................  1    1  seq
....................aacccguagauccgaucuugugAu..................................................................  3    1  seq
....................aacccguagauccgaucuuguguGg.................................................................  1    1  seq
....................aacccguagauccgaAcuuguguug.................................................................  1    1  seq
.....................acccguagauccgaucuugugG...................................................................  1    1  seq
.....................acccguagauccgaAcuugugu...................................................................  3    1  seq
......................cccguagauccgaucuugu.....................................................................  1    0  seq
.......................ccguagauccgaucuugu.....................................................................  7    0  seq
.......................ccguagauccgaucuugug....................................................................  11   0  seq
.......................ccguagauccgaucuugugA...................................................................  1    1  seq
.......................ccguagauccgaucuugugG...................................................................  1    1  seq
.......................ccguagauccgaAcuugugu...................................................................  5    1  seq
........................cguagauccgaucuugug....................................................................  72   0  seq
........................cguagauccgaucuugugu...................................................................  6    0  seq
........................cguagauccgaAcuugugu...................................................................  16   1  seq
........................cguagauccgaucuugugA...................................................................  13   1  seq
.........................guagauccgaucuugugu...................................................................  1    0  seq
.........................guagauccgaAcuugugu...................................................................  3    1  seq
.........................guagauccgaucuugugA...................................................................  6    1  seq
........................................................acaagcucgcuucuauggguc.................................  2    0  seq
........................................................acaagcucgcuucuaugggucu................................  3    0  seq
........................................................acaagcucgcuucuaugggucug...............................  3    0  seq
........................................................acaagcucgcuuUuaugggucugu..............................  1    1  seq
........................................................acaagcucgcuucuaugggucugu..............................  9    0  seq
.........................................................caagcucgcuucuauUgguc.................................  1    1  seq
.........................................................caagcucgcuucuauggguc.................................  5    0  seq
.........................................................caagcucgcuucuaugggucA................................  1    1  seq
.........................................................caGgcucgcuucuaugggucu................................  1    1  seq
.........................................................caagcucgcuucuaugggucu................................  38   0  seq
.........................................................caagcucgcuucuaugggucug...............................  39   0  seq
.........................................................caagcucgcuucuaugggGcug...............................  1    1  seq
.........................................................caagcucgcuucuaugggucuU...............................  1    1  seq
.........................................................caGgcucgcuucuaugggucug...............................  5    1  seq
.........................................................caagcucgcuucuaugggGcugu..............................  2    1  seq
.........................................................Gaagcucgcuucuaugggucugu..............................  1    1  seq
.........................................................caagcucgcuucuaugAgucugu..............................  1    1  seq
.........................................................caGgcucgcuucuaugggucugu..............................  7    1  seq
.........................................................caagcucgcuucuaugggguAugu.............................  1    1  seq
.........................................................caagcucgcuucuaugggucugu..............................  372  0  seq
.........................................................caagcucgcuucuaugggucugG..............................  2    1  seq
.........................................................caagcucgcuucuaugggCcugu..............................  1    1  seq
.........................................................caagcucgcuucuaugggucuguU.............................  21   1  seq
.........................................................caagcucgcuucuaugggucuguC.............................  1    1  seq
.........................................................caagcucgcuucuaugggucuguCu............................  1    1  seq
.........................................................caagcucgcuucuaugggucuguUu............................  2    1  seq
..........................................................aGgcucgcuucuaugggucugu..............................  2    1  seq
..........................................................aagcucgcuucuaugggGcugu..............................  1    1  seq
..........................................................aagcucgcuucuaugggucugu..............................  1    0  seq
```

Provisional ID	:	mun-novel70
Score total	:	4.9
Score for star read(s)	:	-1.3
Score for read counts	:	0
Score for mfe	:	1.6
Score for randfold	:	1.6
Score for cons. seed	:	3
Total read count	:	20
Mature read count	:	20
Loop read count	:	0
Star read count	:	0

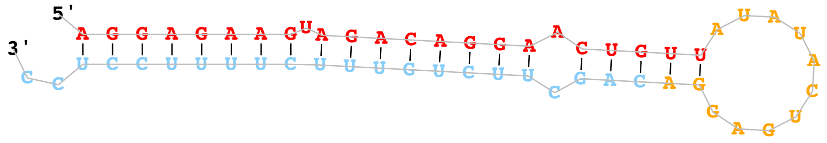

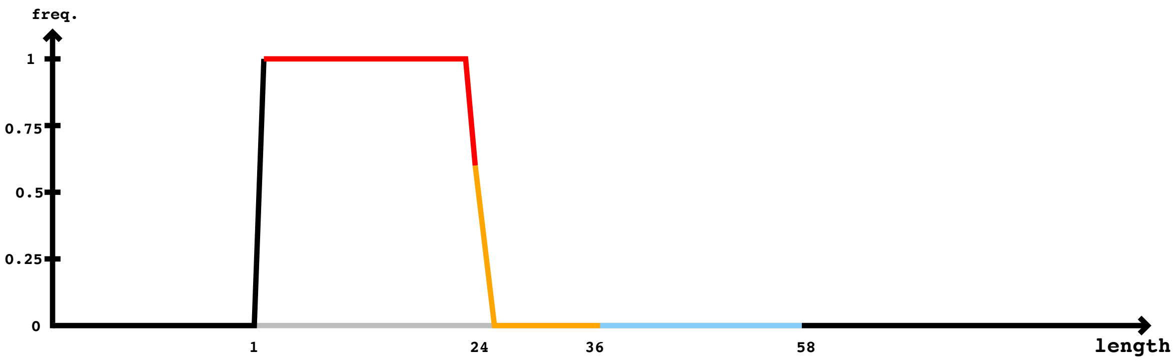

Mature Star

```
5'- uacaggguaaaaaugugaggaggagaaguagacaggaacuguuauauacugaggacagcuucuguuucuuuuccuccuugcacugguuggggugggggaagaagguagaagaa   -3'    exp
    ..(((........((..((((((((((.((((((((.(((((..........))))).))))))))))))))))))..))))).............................   reads      mm        sample
    ....................aggagaaguagacaggaacugu.......................................................................   8          0         seq
    ....................aggagaaguagacaggaacuguu......................................................................   12         0         seq
```

Provisional ID	:	mun-miR-130c
Score total	:	1988.6
Score for star read(s)	:	3.9
Score for read counts	:	1978.2
Score for mfe	:	1.9
Score for randfold	:	1.6
Score for cons. seed	:	3
Total read count	:	3892
Mature read count	:	3860
Loop read count	:	0
Star read count	:	32

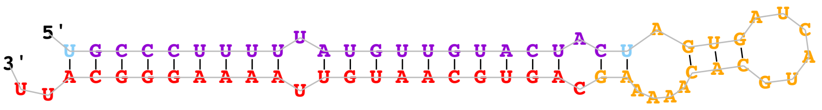

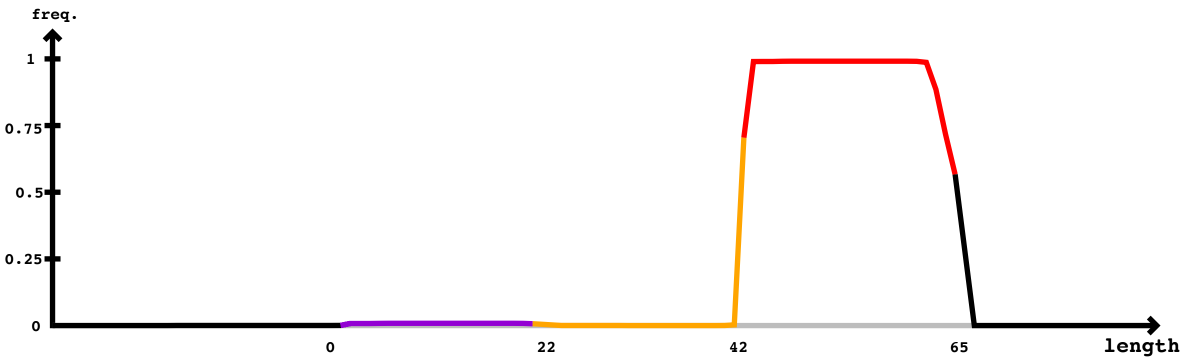

Star **Mature**

```
5'- cuuuuuccuucgaguggugucuguccagugcccuuuuuauguuguacuacuagugaucaugcacaaaaagcagugcaauguuaaaagggcauuggccagcaaugcuaucuacg   -3'   obs
    cuuuuuccuucgaguggugucuguccagugcccuuuuuauguuguacuacuagugaucaugcacaaaaagcagugcaauguuaaaagggcauuggccagcaaugcuaucuacg         exp
    ...........(((..((.(((.(((((((((((((.(((((((((.((.(((......)))....)).))))))))).))))))))))))).)))))..)))..........   reads   mm   sample
    ...........gaguggugucuguccagu....................................................................................   1       0    seq
    ............................ugcccuuuuuauguuguacua................................................................   1       0    seq
    .............................gcccuuuuuauguuguac..................................................................   1       0    seq
    .............................gcccuuuuuauguuguacu.................................................................   5       0    seq
    .............................gcccuuuuuauguuguacua................................................................   3       0    seq
    .............................gcccuuuuuauguuguacuac...............................................................   12      0    seq
    .............................gcccuuuuuauguuguacuaA...............................................................   1       1    seq
    .............................gcccuuuuuauguuguacuacu..............................................................   6       0    seq
    .............................gcccuuuuuauguuguacuacuagugauc.......................................................   1       0    seq
    ...............................cuuuuuauguuguacuacu...............................................................   1       0    seq
    ...............................cuuuuuauguuguacuacA...............................................................   1       1    seq
    ................................uuuuuauguuguacuacu...............................................................   1       0    seq
    ...................................................................agcagugcaauguuaaaagggc........................   1       0    seq
    ....................................................................gcagugcaauguuaaaagggc........................   1       0    seq
    ....................................................................gcagugcaauguuaaaagggcU.......................   4       1    seq
    ....................................................................gcagugcaauguuaaaagggcaA......................   1       1    seq
    ....................................................................gcagugcaauguuaaaagggcUu......................   3       1    seq
    .....................................................................cagugcaauguuaaaagg..........................   2       0    seq
    .....................................................................cagugcaauguuaaaaggg.........................   14      0    seq
    .....................................................................cagGgcaauguuaaaaggg.........................   1       1    seq
    .....................................................................caguUcaauguuaaaagggc........................   1       1    seq
    .....................................................................cagugcaauguuaaaagggA........................   3       1    seq
    .....................................................................cagugcaauguuaaaagggU........................   4       1    seq
    .....................................................................Uagugcaauguuaaaagggc........................   1       1    seq
    .....................................................................cagugcaauguuaaaagggc........................   354     0    seq
    .....................................................................Gagugcaauguuaaaagggc........................   1       1    seq
    .....................................................................cagugcaauguuaaaagggcC.......................   21      1    seq
    .....................................................................cagGgcaauguuaaaagggca.......................   1       1    seq
    .....................................................................caUugcaauguuaaaagggca.......................   1       1    seq
    .....................................................................caguCcaauguuaaaagggca.......................   1       1    seq
    .....................................................................Gagugcaauguuaaaagggca.......................   1       1    seq
    .....................................................................cagugcaauguuaaaagggcU.......................   61      1    seq
    .....................................................................cagugcaauguuaaaaggCca.......................   4       1    seq
```

```
                             Star                                    Mature
cuuuuuccuucgaguggugucuguccagugcccuuuuuauguuguacuacuagugaucaugcacaaaaagcagugcaauguuaaaagggcauuggccagcaaugcuaucuacg
......................................................................cagugcaauguuaaaagggAa......................    2     1    seq
......................................................................cagugcaauguuaaaagggca......................    509   0    seq
......................................................................caAugcaauguuaaaagggca......................    1     1    seq
......................................................................cagugcaauguuaaaagggcaA.....................    28    1    seq
......................................................................cagugcaauguuaaaagggAau.....................    1     1    seq
......................................................................cagugcaauguuaaaagggUau.....................    1     1    seq
......................................................................cagugcaauguuaaaagggcau.....................    456   0    seq
......................................................................cGgugcaauguuaaaagggcau.....................    1     1    seq
......................................................................cagugcaauguuaaaagggcCu.....................    2     1    seq
......................................................................cagugcaauguuaaaagggcaC.....................    15    1    seq
......................................................................cagugcaauguuaaaagggcaG.....................    1     1    seq
......................................................................cagugcaauguuaaaagggcUu.....................    2     1    seq
......................................................................cagugcaauguCaaaagggcauu....................    1     1    seq
......................................................................cagugcaauguuaaaagUgcauu....................    1     1    seq
......................................................................cagugcaauguuaaaagggcauG....................    14    1    seq
......................................................................cagugcaauguuaaaagggcaCu....................    3     1    seq
......................................................................cagugcaauguuGaaagggcauu....................    1     1    seq
......................................................................caguCcaauguuaaaagggcauu....................    1     1    seq
......................................................................cagugcaauguuaaCagggcauu....................    1     1    seq
......................................................................cagugcaauguuaaaagggcUuu....................    1     1    seq
......................................................................caAugcaauguuaaaagggcauu....................    1     1    seq
......................................................................cagugcaauguuaaaagggcaAu....................    9     1    seq
......................................................................Aagugcaauguuaaaagggcauu....................    1     1    seq
......................................................................cagugcaGuguuaaaagggcauu....................    1     1    seq
......................................................................cagugcaauguuaaaagggcauC....................    4     1    seq
......................................................................cagugcaauguuaaaagggcauA....................    130   1    seq
......................................................................cagCgcaauguuaaaagggcauu....................    3     1    seq
......................................................................cagugcaauguuaaaagggcauu....................    1068  0    seq
......................................................................cagugcaauguuaaaagggcauuA...................    1     1    seq
......................................................................cagugcaauguuaaaagggcauuU...................    3     1    seq
.......................................................................agugcaauguuaaaagggc.......................    29    0    seq
.......................................................................agugcaauguuaaaagggca......................    48    0    seq
.......................................................................agugcaauguuaaUagggcau.....................    1     1    seq
.......................................................................agugcaauguuaaaGgggcau.....................    1     1    seq
.......................................................................agugcaauguuaaaagggcaA.....................    1     1    seq
.......................................................................agugcaauguuaaaagggcau.....................    71    0    seq
.......................................................................agugcaauguuaaaagggcaC.....................    1     1    seq
.......................................................................agugcaauguuaaaagggUau.....................    1     1    seq
.......................................................................agugcaauguuaaaagUgcauu....................    2     1    seq
.......................................................................aUugcaauguuaaaagggcauu....................    1     1    seq
.......................................................................agugcaauguuaaaagggcauG....................    11    1    seq
.......................................................................agugcaauguuaaaagggcauC....................    1     1    seq
.......................................................................agugcaaugAuaaaagggcauu....................    1     1    seq
.......................................................................agugcGauguuaaaagggcauu....................    1     1    seq
.......................................................................agugcaauguuaaaagggcauu....................    906   0    seq
.......................................................................agugcaauguuaaaagggcauA....................    29    1    seq
.......................................................................agugcaauguAaaaagggcauu....................    1     1    seq
.......................................................................agugcaauguuaaaagggcaAu....................    1     1    seq
.......................................................................agugcaauCuuaaaagggcauu....................    1     1    seq
.......................................................................agugcaauguuaaaagggcauuU...................    2     1    seq
.......................................................................agugcaauguuaaaagggcauug...................    1     0    seq
........................................................................gugcaauguuaaaagggcauu....................    1     0    seq
..........................................................................gcaauguuaaaagggcauu....................    3     0    seq
...........................................................................caauguuaaaagggcauu....................    1     0    seq
```

Provisional ID	:	mun-miR-92-2
Score total	:	5.4
Score for star read(s)	:	-1.3
Score for read counts	:	0
Score for mfe	:	2.1
Score for randfold	:	1.6
Score for cons. seed	:	3
Total read count	:	92223
Mature read count	:	92218
Loop read count	:	1
Star read count	:	4

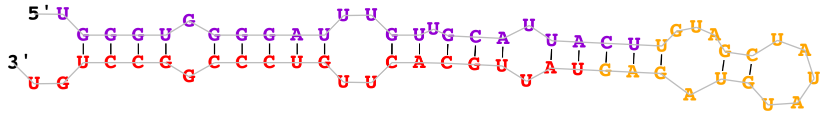

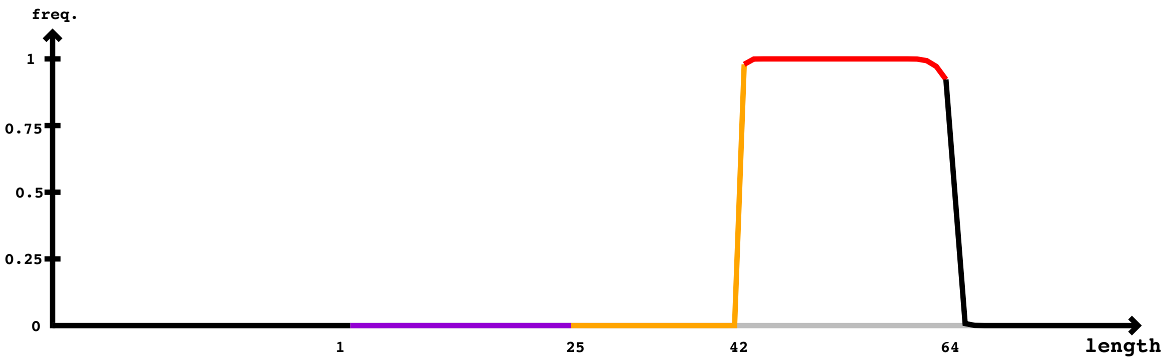

Star **Mature**

```
5'- ugugaaggugccuugcgcugguuucuccauggguggggauuuguugcauuacuuguagcuauauguagaguauugcacuugucccggccuguggaggaaaggaggacuugu   -3'   obs
    ugugaaggugccuugcgcugguuucuccaugggugggggauuuguugcauuacuuguagcuauauguagaguauugcacuugucccggccuguggaggaaaggaggacuugu         exp
    .....((((.((((.(.....(((((((((((((.(((((..((.(((.(((((...((.....)).))))).))))).)))))..))))).))))))))))))).))))))))..   reads   mm   sample
    ..........ccuugcgcugguuucuccau.................................................................................   1   0   seq
    .............................uggguggggauuuguugcauuacu..........................................................   1   0   seq
    ..............................gggugggggauuuguugcauuaA...........................................................   1   1   seq
    ..............................gggugggggauuuguugcauuacuuguagc....................................................   1   0   seq
    ................................guggggauuuguugcauuacuuU........................................................   1   1   seq
    .................................uggggauuuguugcauuacuug........................................................   1   0   seq
    ....................................................uuguagcuauauguagagu........................................   1   0   seq
    .....................................................................Cuauugcacuugucccggccugu...................   1   1   seq
    .....................................................................guauugcacuugucccggccugu...................   1   0   seq
    .....................................................................Uuauugcacuugucccggccugu...................   1   1   seq
    .....................................................................Auauugcacuugucccggccugu...................   2   1   seq
    ......................................................................uauugcacuugucccggc.......................   27   0   seq
    ......................................................................uauugcacuCgucccggc.......................   22   1   seq
    ......................................................................uauugcacuugucccggcU......................   4   1   seq
    ......................................................................uauugcacuugucccggcA......................   6   1   seq
    ......................................................................uauugcacuuguUccggcc......................   1   1   seq
    ......................................................................uauugcacuugucccgAcc......................   1   1   seq
    ......................................................................uauugcacuugucGcggcc......................   1   1   seq
    ......................................................................uauugcacuCgucccggcc......................   72   1   seq
    ......................................................................uauugcacuugucUcggcc......................   1   1   seq
    ......................................................................uauugcacuugucccggcc......................   466   0   seq
    ......................................................................uauugcacuAgucccggcc......................   1   1   seq
    ......................................................................uauugcacuuguUccggccu.....................   1   1   seq
    ......................................................................uauugcacuuguccAggccu.....................   1   1   seq
    ......................................................................uauugcacuuUucccggccu.....................   2   1   seq
    ......................................................................uauugcacuugucccggccG.....................   14   1   seq
    ......................................................................Gauugcacuugucccggccu.....................   1   1   seq
    ......................................................................uauugcacuuguccUggccu.....................   1   1   seq
    ......................................................................uauugcacuugucUcggccu.....................   3   1   seq
    ......................................................................uauuUcacuugucccggccu.....................   2   1   seq
    ......................................................................uauugcaAuugucccggccu.....................   1   1   seq
    ......................................................................uauugcacuugucccggccC.....................   3   1   seq
    ......................................................................uauugcacuugucccggccA.....................   62   1   seq
```

Star Mature

```
ugugaaggugccuugcgcugguuucuccauggguggggauuuguugcauuacuuguagcuauauguagaguauugcacuugucccggccuguggaggaaaggaggacuugu
......................................................................uauugcacuugCcccggccu.....................   1     1  seq
......................................................................uauugcacuugucccgAccu.....................   1     1  seq
......................................................................uauugcacuugucccggcUu.....................   2     1  seq
......................................................................uauugcacuugucccggccu.....................   1358  0  seq
......................................................................uauugcacuugucccggAcu.....................   1     1  seq
......................................................................uauugcacuAgucccggccu.....................   1     1  seq
......................................................................uauugcacuCgucccggccu.....................   514   1  seq
......................................................................uauugAacuugucccggccu.....................   1     1  seq
......................................................................uauugcacuugucccggccGg....................   48    1  seq
......................................................................uauugcacuuAucccggccug....................   1     1  seq
......................................................................Aauugcacuugucccggccug....................   2     1  seq
......................................................................uauugcacuuguGccggccug....................   2     1  seq
......................................................................uauugAacuugucccggccug....................   2     1  seq
......................................................................uauuAcacuugucccggccug....................   1     1  seq
......................................................................uauugcacuugucccggGcug....................   2     1  seq
......................................................................uauugcacuugucccggccuA....................   20    1  seq
......................................................................uauugcacuuguccUggccug....................   1     1  seq
......................................................................uauugcacuugucccggccug....................   4232  0  seq
......................................................................uauCgcacuugucccggccug....................   2     1  seq
......................................................................Gauugcacuugucccggccug....................   3     1  seq
......................................................................uauugcacuugAcccggccug....................   1     1  seq
......................................................................uauugcacuugucccggUcug....................   1     1  seq
......................................................................uauugcacuugucUcggccug....................   1     1  seq
......................................................................uauugcacAugucccggccug....................   1     1  seq
......................................................................uauugcUcuugucccggccug....................   1     1  seq
......................................................................uauugcacuuguccAggccug....................   1     1  seq
......................................................................uauugcacuugucccgUccug....................   1     1  seq
......................................................................uGuugcacuugucccggccug....................   1     1  seq
......................................................................uauugcacuugucccgAccug....................   1     1  seq
......................................................................uauugcCcuugucccggccug....................   1     1  seq
......................................................................uauugcacuugucccggccuU....................   112   1  seq
......................................................................uauugcGcuugucccggccug....................   1     1  seq
......................................................................uauugcacuuguAccggccug....................   1     1  seq
......................................................................uauugcacuugucccggccCg....................   2     1  seq
......................................................................uauugcacuCgucccggccug....................   1     1  seq
......................................................................uauugcacuugucccggAcug....................   1     1  seq
......................................................................uauugcacCugucccggccug....................   2     1  seq
......................................................................uauugcacuuguUccggccug....................   2     1  seq
......................................................................uauugcacuuCucccggccug....................   1     1  seq
......................................................................uauugcacuugucccggccAg....................   1     1  seq
......................................................................Cauugcacuugucccggccug....................   1     1  seq
......................................................................uauugcacuugucccAgccug....................   1     1  seq
......................................................................uauugcacuugucccggccuAu...................   28    1  seq
......................................................................uauugcacuugucUcggccugu...................   12    1  seq
......................................................................uauGgcacuugucccggccugu...................   1     1  seq
......................................................................uauugcacuugucccgUccugu...................   14    1  seq
......................................................................uauugcacuugucccCgccugu...................   3     1  seq
......................................................................uauugcacuugucccggccugG...................   414   1  seq
......................................................................uauugcGcuugucccggccugu...................   15    1  seq
......................................................................uauugcacuCgucccggccugu...................   8     1  seq
......................................................................uauugcacuugucccggccCgu...................   12    1  seq
......................................................................uauugUacuugucccggccugu...................   16    1  seq
......................................................................uauugcacuugCcccggccugu...................   18    1  seq
......................................................................uauugAacuugucccggccugu...................   9     1  seq
......................................................................uauugcacuuguccUggccugu...................   18    1  seq
......................................................................uaGugcacuugucccggccugu...................   1     1  seq
......................................................................uauugcacuugucccggccAgu...................   3     1  seq
......................................................................uauugcacuugGcccggccugu...................   3     1  seq
......................................................................uauugcacGugucccggccugu...................   1     1  seq
......................................................................uauugcacuugucccggccuUu...................   102   1  seq
......................................................................uaCugcacuugucccggccugu...................   6     1  seq
......................................................................uauugcacuuUucccggccugu...................   11    1  seq
......................................................................uauugcacuugucccggcGugu...................   1     1  seq
......................................................................uauugcacuuAucccggccugu...................   8     1  seq
......................................................................uauugcacuugucccgAccugu...................   9     1  seq
......................................................................uUuugcacuugucccggccugu...................   2     1  seq
......................................................................uauuAcacuugucccggccugu...................   4     1  seq
......................................................................uauugcacuuguccAggccugu...................   18    1  seq
......................................................................uauugcacuAgucccggccugu...................   1     1  seq
......................................................................uauugcacuugucccggcUugu...................   6     1  seq
```

Star **Mature**

ugugaaggugccuugcgcugguuucuccaugggugggga uuuguugcauuacuuguagcuauauguagaguauugcacuugucccggccuguggaggaaaggaggacuugu

....uauugcacuugucccggGcugu...................	4	1	seq
....Gauugcacuugucccggccugu...................	41	1	seq
....uauugcacuugucccggccugu...................	70312	0	seq
....uauugcacuugucAcggccugu...................	19	1	seq
....uGuugcacuugucccggccugu...................	6	1	seq
....uauAgcacuugucccggccugu...................	4	1	seq
....Cauugcacuugucccggccugu...................	18	1	seq
....uauugcacAugucccggccugu...................	2	1	seq
....uauugcacuugucccUgccugu...................	19	1	seq
....uauugcacCugucccggccugu...................	5	1	seq
....uauugcaGuugucccggccugu...................	1	1	seq
....uauCgcacuugucccggccugu...................	6	1	seq
....uaAugcacuugucccggccugu...................	2	1	seq
....uCuugcacuugucccggccugu...................	2	1	seq
....uauugcaAuugucccggccugu...................	8	1	seq
....uauugcacuugucccggccGgu...................	1	1	seq
....uauuCcacuugucccggccugu...................	4	1	seq
....uauugcacuuguAccggccugu...................	5	1	seq
....uauugcacuuguccGggccugu...................	5	1	seq
....uauugcacuugAcccggccugu...................	14	1	seq
....uauugcacuugucccggUcugu...................	2	1	seq
....uauugcacuugucGcggccugu...................	4	1	seq
....uauugcacuugucccggccugA...................	5025	1	seq
....uauugcacuugucccgCccugu...................	1	1	seq
....Aauugcacuugucccggccugu...................	36	1	seq
....uauugcaUuugucccggccugu...................	6	1	seq
....uauugcacuuguUccggccugu...................	33	1	seq
....uauugcacuugucccggccuCu...................	3	1	seq
....uauugcacuugucccAgccugu...................	5	1	seq
....uauugcacuugucccggAcugu...................	14	1	seq
....uauugcacuuguGccggccugu...................	2	1	seq
....uauugcacuugucccggccugC...................	169	1	seq
....uauugGacuugucccggccugu...................	3	1	seq
....uauuUcacuugucccggccugu...................	11	1	seq
....uauugcUcuugucccggccugu...................	2	1	seq
....uauugcacuuCucccggccugu...................	4	1	seq
....uauugcacuugucccggcAugu...................	4	1	seq
....uauugcacuugucccggccugAg..................	25	1	seq
....uauugcacuugucccggccuguC..................	67	1	seq
....uauugcacuugucccggccuguA..................	6035	1	seq
....uauugcacuugucccggccugug..................	56	0	seq
....uauugcacuugucccggccuguAg.................	485	1	seq
....uauugcacuugucccggccugugA.................	17	1	seq
....uauugcacuugucccggccuguCg.................	1	1	seq
....uauugcacuugucccggccugugU.................	18	1	seq
....uauugcacuugucccggccugugg.................	6	0	seq
....uauugcacuugucccggccugAgg.................	1	1	seq
....uauugcacuugucccggccuguAga................	54	1	seq
....auugcacuugucccggcc......................	7	0	seq
....auugcacuCgucccggccu.....................	8	1	seq
....auugcacuugucccggccu.....................	10	0	seq
....auugcacuugucccggccGg....................	1	1	seq
....auugcacuugucccggccug....................	59	0	seq
....auugcacuugucccggccuU....................	1	1	seq
....auugcacuugucccggAcugu...................	1	1	seq
....auugcacuugucccggccugG...................	9	1	seq
....auugcacuugucccUgccugu...................	1	1	seq
....auugcacuugucccggccuUu...................	1	1	seq
....auugcacuugucccgUccugu...................	2	1	seq
....auugcacuuguAccggccugu...................	1	1	seq
....auugcacuugucGcggccugu...................	1	1	seq
....auugcacuugucUcggccugu...................	2	1	seq
....Uuugcacuugucccggccugu...................	1	1	seq
....auugcacuugucccggccugA...................	12	1	seq
....Guugcacuugucccggccugu...................	1	1	seq
....auugcacuugucccggccugu...................	1351	0	seq
....auugcacuugCcccggccugu...................	1	1	seq
....auugcacuugucccggccugC...................	1	1	seq
....Uuugcacuugucccggccugug..................	1	1	seq
....auugcacuugucccggUcugug..................	1	1	seq

```
                              Star                                          Mature
ugugaaggugccuugcgcugguuucuccaugggugggggauuuguugcauuacuuguagcuauauguagaguauugcacuugucccggccuguggaggaaaggaggacuugu
.......................................................................auugcacuugucccggccugAg..................   1     1     seq
.......................................................................aCugcacuugucccggccugug..................   1     1     seq
.......................................................................auugcacuugucccggccuguC..................   14    1     seq
.......................................................................auugcacuugucccggccuguA..................   90    1     seq
.......................................................................auugcacuugucccggccugug..................   144   0     seq
.......................................................................auugcacuugucccggccugugA.................   38    1     seq
.......................................................................auugcacuugucccggccugugU.................   62    1     seq
.......................................................................auugcacuugucccggccugugg.................   8     0     seq
.......................................................................auugcacuugucccggccuguAg.................   6     1     seq
.......................................................................auugcacuugucccggccugugUa................   4     1     seq
........................................................................uugcacuugucccggccu.....................   4     0     seq
........................................................................uugcacuugucccggccugA...................   1     1     seq
........................................................................uugcacuugucccggccugu...................   18    0     seq
........................................................................uugcacuugucccggccuguC..................   1     1     seq
........................................................................uugcacuugucccggccuguA..................   10    1     seq
........................................................................uugcacuugucccggccugugg.................   1     0     seq
..........................................................................gcacuugucccggccugu...................   2     0     seq
```

Provisional ID		mun-novel67
Score total	:	1.6
Score for star read(s)	:	-1.3
Score for read counts	:	0
Score for mfe	:	1.9
Score for randfold	:	1.6
Score for cons. seed	:	-0.6
Total read count	:	42
Mature read count	:	42
Loop read count	:	0
Star read count	:	0

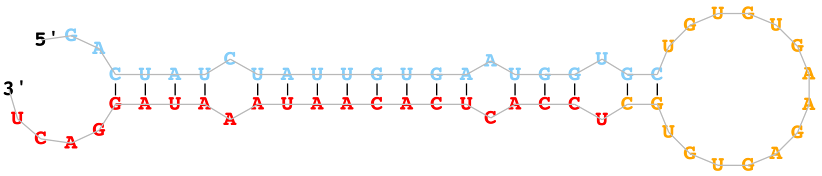

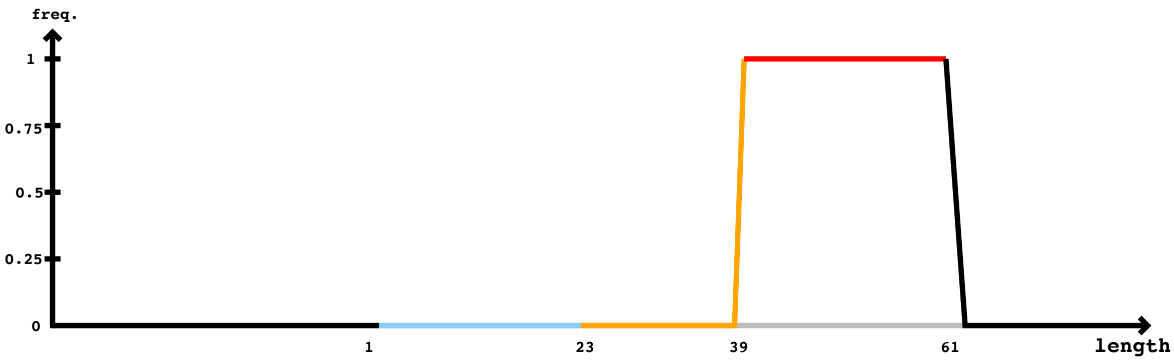

Star **Mature**

```
5'- uugccucaccuugggaauugccaaauccuuaggacuaucuauugugaauggugcugugugaagagugugcuccacucacaauaaaauaggacuggauuuggcaggcauggaga   -3'    exp
    .((((...((...))...((((((((((..((..((((.((((((((.(((.((..............)).))).))))))))).))))..))))))))))))))))......    reads      mm         sample
    ......................................................................uccacucacaauaaaauaggacA....................    1          1          seq
    ......................................................................uccacucacaauaaaauaggacu....................    40         0          seq
    ......................................................................uccacucaUaauaaaauaggacu....................    1          1          seq
```

Provisional ID	:	mun-miR-29a
Score total	:	2202.2
Score for star read(s)	:	3.9
Score for read counts	:	2192.4
Score for mfe	:	1.3
Score for randfold	:	1.6
Score for cons. seed	:	3
Total read count	:	4312
Mature read count	:	4269
Loop read count	:	0
Star read count	:	43

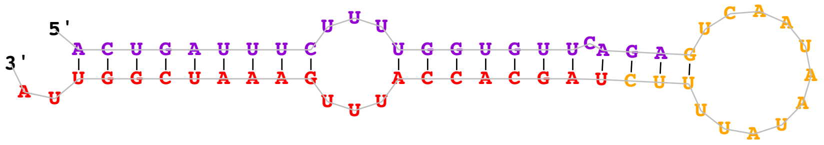

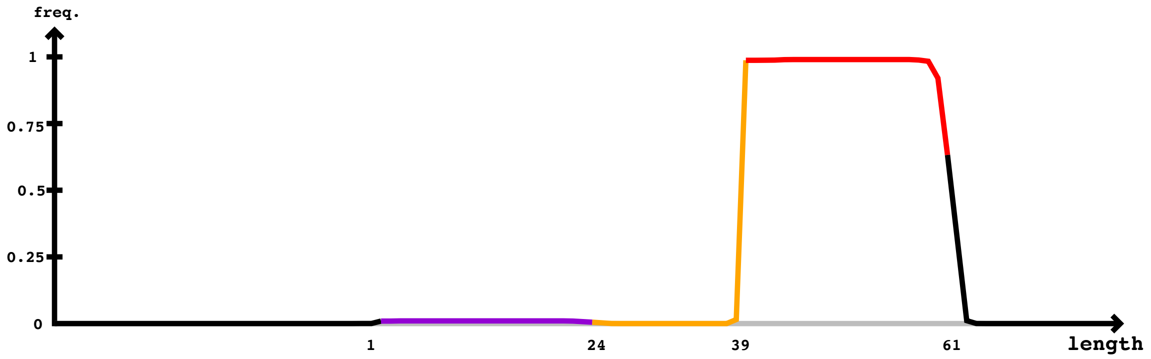

Star Mature

```
5'- cgugcagaucucaguaaccccuuuagaggaugacugauuucuuuugguguucagagucaauaauauuuucuagcaccauuugaaaucgguuauagugauuggggaauug  -3'  obs
    cgugcagaucucaguaaccccuuuagaggaugacugauuucuuuugguguucagagucaauaauauuuucuagcaccauuugaaaucgguuauagugauuggggaauug        exp
    .................((((..(.....((((((((((((...(((((((.((((...........)))))))))))...))))))))))))....)..)))).....  reads  mm  sample
    ..............................ugacugauuucuuuugguguucaga......................................................  1  0  seq
    ................................acugauuucuuuugguguuc.........................................................  3  0  seq
    ................................acugauuucuuuugguguuca........................................................  9  0  seq
    ................................acugauuucuuuugguguucag.......................................................  8  0  seq
    ................................acugauuucuuuugguguucagU......................................................  10  1  seq
    ................................acugauuucuuuugguguucaga......................................................  9  0  seq
    ..................................ugauuucuuuugguguucag.......................................................  1  0  seq
    ..................................ugauuucuuuugguguucaga......................................................  1  0  seq
    ..................................ugauuucuuuugguguucagagu....................................................  1  0  seq
    .....................................................................cuagcaccauuugaaaucgg....................  4  0  seq
    .....................................................................cuagcaccauuugaaaucggu...................  5  0  seq
    .....................................................................cuagcaccauuugaaaucAguu..................  3  1  seq
    .....................................................................cuagcaccauuugaaaucgguu..................  38  0  seq
    .....................................................................cuagcaccauuugaaaucgguuU.................  2  1  seq
    .....................................................................Guagcaccauuugaaaucgguua.................  1  1  seq
    .....................................................................cuagcaccauuugaaaucgguua.................  5  0  seq
    .....................................................................cuagcaccauuugaaaucgguuC.................  2  1  seq
    .....................................................................cuagcaccauuugaaaucgguAa.................  1  1  seq
    .....................................................................cuagcaccauuugaaaucgguuau................  1  0  seq
    .....................................................................cuagcaccauuugaaaucgguuGu................  2  1  seq
    .....................................................................cuagcaccauuugaaaucgguuaC................  1  1  seq
    ......................................................................uagcaccauuugaaaucg.....................  8  0  seq
    ......................................................................uagcaccauuugaaaucgg....................  12  0  seq
    ......................................................................uagcaccauuugaaaucgA....................  2  1  seq
    ......................................................................uagcaccauuugaaaucgU....................  2  1  seq
    ......................................................................uagcaccauuugaaaucggu...................  245  0  seq
    ......................................................................uagcaccauuugaaaucggA...................  23  1  seq
    ......................................................................uagcaccauuugaaaucggAu..................  4  1  seq
    ......................................................................uagcaccauuugaaaucgguG..................  3  1  seq
    ......................................................................Cagcaccauuugaaaucgguu..................  1  1  seq
    ......................................................................uagcaccauuugaaaucUguu..................  1  1  seq
    ......................................................................uagcacUauuugaaaucgguu..................  1  1  seq
    ......................................................................uagcaccauuugaaaucggGu..................  1  1  seq
```

Star **Mature**

```
cgugcagaucucaguaaccccuuuagaggaugacugauuucuuuugguguucagagucaauaaauauuuucuagcaccauuugaaaucgguuauagugauuggggaauug
.......................................................................uagcaccauuugaaaCcgguu..................	1	1	seq
.......................................................................uagcaccauuugaaaucgguu..................	1171	0	seq
.......................................................................uagcaccauuugaaaucgCuu..................	1	1	seq
.......................................................................uaUcaccauuugaaaucgguu..................	1	1	seq
.......................................................................uagcaccauuugaaaucAguu..................	3	1	seq
.......................................................................uagcGccauuugaaaucgguu..................	1	1	seq
.......................................................................uagcaccauuugaaaucgguA..................	12	1	seq
.......................................................................uagcaccaCuugaaaucgguua.................	1	1	seq
.......................................................................Gagcaccauuugaaaucgguua.................	1	1	seq
.......................................................................uagcaccauCugaaaucgguua.................	1	1	seq
.......................................................................uagcaccauuugaaaucUguua.................	1	1	seq
.......................................................................uagcaccauuugaaaucgguua.................	1344	0	seq
.......................................................................uagcaccauuugaaGucgguua.................	1	1	seq
.......................................................................uagcaccauuugaaaucgguCa.................	1	1	seq
.......................................................................uGgcaccauuugaaaucgguua.................	1	1	seq
.......................................................................uagcacUauuugaaaucgguua.................	1	1	seq
.......................................................................uagcaccauuugaaaucgguuC.................	27	1	seq
.......................................................................uagcaccauuugaaaucgguuU.................	97	1	seq
.......................................................................uagcaccauuugaaaucgguuG.................	52	1	seq
.......................................................................uagcGccauuugaaaucgguua.................	1	1	seq
.......................................................................Aagcaccauuugaaaucgguua.................	1	1	seq
.......................................................................uagUaccauuugaaaucgguua.................	1	1	seq
.......................................................................uagcaccauuugaaaucgguAa.................	85	1	seq
.......................................................................uagcaUcauuugaaaucgguuau................	1	1	seq
.......................................................................uagcaccauuugaaaucgguuaC................	109	1	seq
.......................................................................uagcaccauuugaaaucgguuGu................	33	1	seq
.......................................................................uagcaccauuugaaaucggGuau................	1	1	seq
.......................................................................uagcaccauuugaaaucgguAau................	5	1	seq
.......................................................................uagcacUauuugaaaucgguuau................	1	1	seq
.......................................................................uagcaccauuugaaaucgguuUu................	25	1	seq
.......................................................................uagcaccauuugaaaucgguuCu................	3	1	seq
.......................................................................uagcaccauuugaaaucgguuaG................	24	1	seq
.......................................................................uagcaccauuugaaaucgguuaA................	488	1	seq
.......................................................................Gagcaccauuugaaaucgguuau................	1	1	seq
.......................................................................uagcaccauuugaaaucgguuau................	346	0	seq
.......................................................................uagcaccauuugaaaucgguuaua...............	1	0	seq
.......................................................................uagcaccauuugaaaucgguuauU...............	7	1	seq
.......................................................................uagcaccauuugaaaucgguuaGa...............	2	1	seq
.......................................................................uagcaccauuugaaaucgguuaAa...............	35	1	seq
.......................................................................uagcaccauuugaaaucgguuaAag..............	1	1	seq
.........................................................................gcaccauuugaaaucgguuau................	1	0	seq
..........................................................................caccauuugaaaucgguua.................	1	0	seq
..........................................................................caccauuugaaaucgguuauU...............	1	1	seq
...........................................................................accauuugaaaucgguU..................	1	1	seq
...........................................................................accauuugaaaucgguua.................	4	0	seq
...........................................................................accauuugaaaucgguuaA................	2	1	seq
............................................................................ccauuugaaaucgguuUu................	2	1	seq
```

Provisional ID	:	mun-novel101
Score total	:	1.6
Score for star read(s)	:	-1.3
Score for read counts	:	0
Score for mfe	:	1.9
Score for randfold	:	1.6
Score for cons. seed	:	-0.6
Total read count	:	86
Mature read count	:	78
Loop read count	:	3
Star read count	:	5

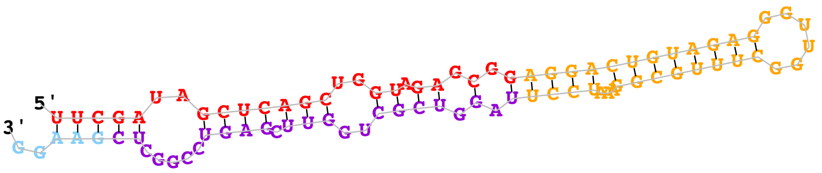

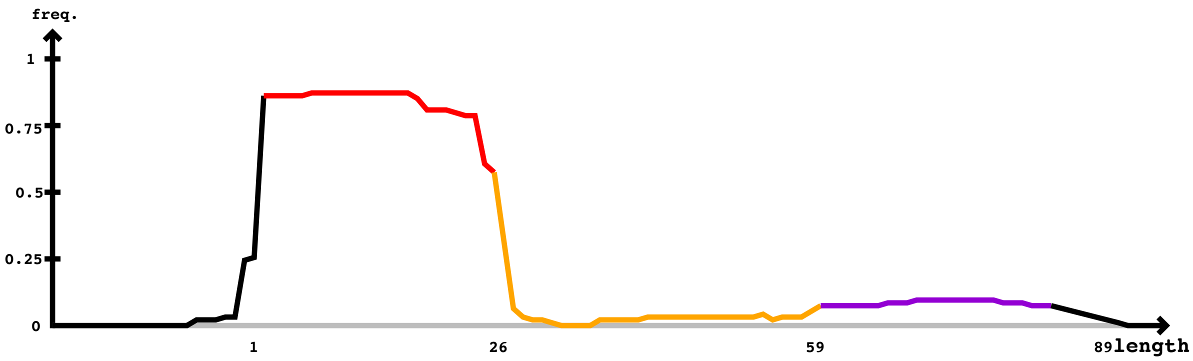


```
                          Mature                                                    Star
5'- ggaggcgguugucaggucccuucgauagcucagcugguagagcggaggacuguagaggguuggcuuugcggaaauccuuaggucgcugguucgaguccggcucgaaggagacau  -3'   obs
    ggaggcgguugucaggucccuucgauagcucagcugguagagcggaggacuguagaggguuggcuuugcggaaauccuuaggucgcugguucgaguccggcucgaaggagacau         exp
    ...(((....)))..((((((((((..(((((((..((.((.(.(((((((((((((......))))))))...))))).).))))..))).)))).....))))))).)))..   reads   mm   sample
    .............aggucccuucgauagcucagcugU.............................................................................   1       1    seq
    .............aggucccuucgauagcucagcugguagagc.......................................................................   1       0    seq
    ................ucccuucgauagcucagcugguag..........................................................................   1       0    seq
    ..................ccuucCauagcucagcug..............................................................................   1       1    seq
    ..................ccuucUauagcucagcug..............................................................................   1       1    seq
    ..................ccuucgauagcucagcugU.............................................................................   3       1    seq
    ..................ccuucgauaAcucagcugguaga.........................................................................   1       1    seq
    ..................ccuucgauagcucagcuggCagagc.......................................................................   9       1    seq
    ..................ccuucgauagcucagcuggAagagc.......................................................................   2       1    seq
    ..................ccuucgauagcucagcuggGagagc.......................................................................   1       1    seq
    ..................ccuucCauagcucagcugguagagc.......................................................................   1       1    seq
    ..................ccuucgauagcucagcugguagagcggaggaA................................................................   1       1    seq
    ...................cuucgauagcucagcugguagagcgU.....................................................................   1       1    seq
    ....................uucgauagcucagcuggGagagc.......................................................................   1       1    seq
    ....................uucgauagcucagcugguagagU.......................................................................   1       1    seq
    ....................uucgauagcucagcCgguagagc.......................................................................   1       1    seq
    ....................uucgaAagcucagcugguagagcg......................................................................   1       1    seq
    ....................uucgauagcucagcGgguagagcg......................................................................   1       1    seq
    ....................uucgauagcucaUcugguagagcg......................................................................   1       1    seq
    ....................uucgauagcucagcugguagagcgU.....................................................................   45      1    seq
    ....................uucgauagcucagcugguagagcggU....................................................................   2       1    seq
    ....................uucgauagcucagcugguagagcggaU...................................................................   3       1    seq
    ....................uucgauagcucagcugguagagcggagU..................................................................   1       1    seq
    .........................uagcucagcugguagagcggagAacu...............................................................   1       1    seq
    .......................................................aggguuggcuuugcggaa.........................................   2       0    seq
    ............................................................uggcuuugcggaaauccuuaggucg.............................   1       0    seq
    .......................................................................aaauccuuaggucgcugguucgaguc.................   1       0    seq
    ..........................................................................uccuuaggucgcugguucgGguccggcuc...........   1       1    seq
    .............................................................................uuaggucgcugguucgUguccgg..............   1       1    seq
    .............................................................................uuaggucgcugguucgGguccggcuc...........   1       1    seq
    ..............................................................................uaggucgcugguucgGguccggcuc...........   1       1    seq
    ..............................................................................uaggucgcugguucgUguccggcuc...........   1       1    seq
    .....................................................................................cugguucgUguccggcuc...........   2       1    seq
```

```
                    Mature                                                     Star
ggaggcgguugucaggucccuucgauagcucagcugguagagcggaggacuguagaggguuggcuuugcggaaauccuuaggucgcugguucgaguccggcucgaaggagacau
........................................................................................guucgaguccggcucgaaggaC....    1           1           seq
```

Provisional ID	:	mun-novel44
Score total	:	43.9
Score for star read(s)	:	3.9
Score for read counts	:	33.8
Score for mfe	:	1.7
Score for randfold	:	1.6
Score for cons. seed	:	3
Total read count	:	78
Mature read count	:	76
Loop read count	:	0
Star read count	:	2

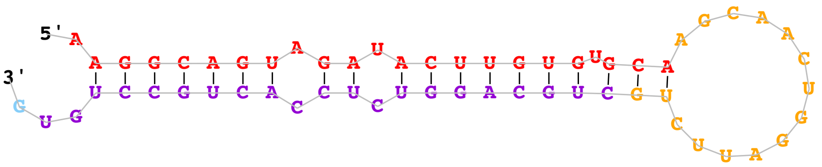

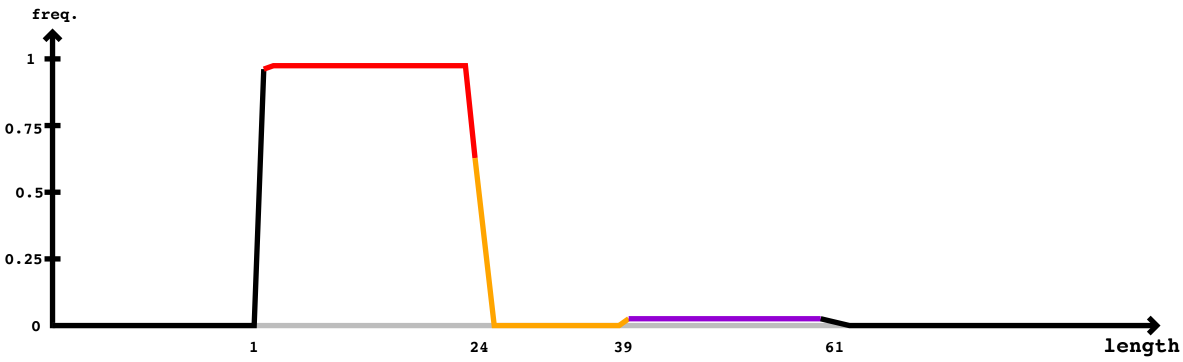


```
                            Mature                                      Star
5'- aaagagagagaacgucuccaaaggcaguagauacuugugugcaagcaacuggauucugcugcaggucuccacugccuguggugggguucuuggacaccaaccucacauggcaa   -3'   obs
    aaagagagagaacgucuccaaaggcaguagauacuugugugcaagcaacuggauucugcugcaggucuccacugccuguggugggguucuuggacaccaaccucacauggcaa         exp
    ...(((.((((((.((.(((.(((((((.((.(((((((.(((.............)))))))))).)).))))))).))).)).))))))((...))...))).........   reads   mm   sample
    ....................aaggcaguagauacuuguCugc.......................................................................   2       1    seq
    ....................aaggcaguagauacuugugugc.......................................................................   25      0    seq
    ....................aaggcaguagauacuugugugcU......................................................................   2       1    seq
    ....................aaggcaguagauacuugugugca......................................................................   41      0    seq
    ....................aaggcaguagauacuugugugcaa.....................................................................   4       0    seq
    ....................aaggcaguagauacuugugugcaU.....................................................................   1       1    seq
    .....................aggcaguagauacuugugugca......................................................................   1       0    seq
    ..........................................................cugcaggucuccacugccugu..................................   2       0    seq
```

Provisional ID	:	mun-novel94
Score total	:	940.9
Score for star read(s)	:	3.9
Score for read counts	:	930
Score for mfe	:	2.4
Score for randfold	:	1.6
Score for cons. seed	:	3
Total read count	:	1836
Mature read count	:	1474
Loop read count	:	0
Star read count	:	362

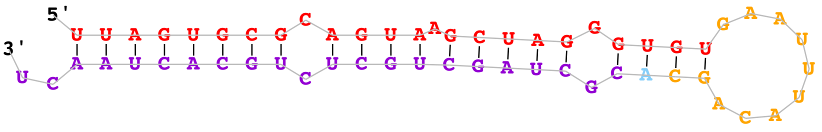

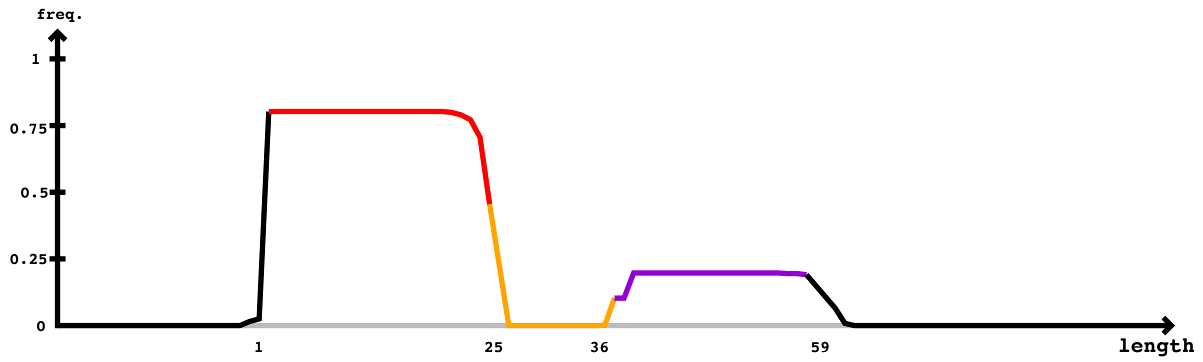

Mature Star

```
5'- ugcuugucuacauggggaaguuagugcgcaguaagcuagggugugaauuuacagcacgcuagcugcucugcacuaacucccccuguggaugcugagugugcacuaagagcgccu   -3'   obs
    ugcuugucuacauggggaaguuagugcgcaguaagcuagggugugaauuuacagcacgcuagcugcucugcacuaacucccccuguggaugcugagugugcacuaagagcgccu         exp
    .((..(((((((.(((((.(((((((((.((((.(((((.((((.........)))).))))))))).))))))))))))))).)))))))))..((.((((.......))))))   reads   mm   sample
    ..................aguuagugcgcaCuaagcuag...........................................................................   1   1   seq
    ..................aguuagugcgcaguaagcuag...........................................................................   5   0   seq
    ..................Gguuagugcgcaguaagcuagg..........................................................................   1   1   seq
    ..................aguuagugcgcaguaagcuagg..........................................................................   8   0   seq
    ..................aguuagugcgcaguaagcuaggg.........................................................................   4   0   seq
    ..................aguuagugcgcaguaagcuaggU.........................................................................   2   1   seq
    ..................aguuagugcgcaguaagcuagggA........................................................................   2   1   seq
    ..................aguuagugcgcaguaagcuagggu........................................................................   3   0   seq
    ..................aguuagugcgcaguaagcuaggguU.......................................................................   2   1   seq
    ...................guuagugcgcaguaagcuagg..........................................................................   1   0   seq
    ...................guuagugcgcaguaagcuaggg.........................................................................   3   0   seq
    ...................guuagugcgcaguaagcuagggA........................................................................   8   1   seq
    ...................guuagugcgcaguaagcuagggu........................................................................   3   0   seq
    ...................guuagugcgcaguaagcuaggguU.......................................................................   3   1   seq
    ....................uuagugcgcaguaagcuagU..........................................................................   1   1   seq
    ....................uuagugcgcaguaagcuagg..........................................................................   6   0   seq
    ....................uuagugcgcaguaagcuaggg.........................................................................   25   0   seq
    ....................uuagugcgcaguaagcuagggA........................................................................   11   1   seq
    ....................uuagAgcgcaguaagcuagggu........................................................................   1   1   seq
    ....................uuagugcgcaguaagcuagggC........................................................................   1   1   seq
    ....................uuagugcgcaguaagcuagggu........................................................................   92   0   seq
    ....................uuagGgcgcaguaagcuagggug.......................................................................   1   1   seq
    ....................uuagugcgcaguaagcuagggGg.......................................................................   3   1   seq
    ....................uuagugcgcaguaagcuaggguA.......................................................................   5   1   seq
    ....................uuagugcgcaguaagcuagggug.......................................................................   417   0   seq
    ....................uuagugcgcaguaagcuaUggug.......................................................................   1   1   seq
    ....................Guagugcgcaguaagcuagggug.......................................................................   2   1   seq
    ....................uuagugcgcaguaagcuagggAg.......................................................................   1   1   seq
    ....................uuagugcgcaguaagcuaggguU.......................................................................   24   1   seq
    ....................uuagugcAcaguaagcuagggug.......................................................................   1   1   seq
    ....................uuagugcgcaguaagcuagggugC......................................................................   2   1   seq
    ....................uuagugcgcaguaagcuaggguUu......................................................................   12   1   seq
    ....................uuagugcgcaguaagcuagggugG......................................................................   28   1   seq
```

```
                    Mature                                  Star
ugcuugucuacauggggaaguuagugcgcaguaagcuagggugugaauuuacagcacgcuagcugcucugcacuaacucccccuguggaugcugagugugcacuaagagcgccu
....................Guagugcgcaguaagcuagggugu......................................................................  1     1  seq
....................uuagugcgcaguaagcuagggugA......................................................................  35    1  seq
....................uuagugcgcaguaagcuGgggugu......................................................................  1     1  seq
....................uuagugcgcaguaagcuagggAgu......................................................................  1     1  seq
....................uuagugcgcaguaagcuaggguAu......................................................................  8     1  seq
....................uuagugcUcaguaagcuagggugu......................................................................  1     1  seq
....................uuagugcgcaguaagcuaggUugu......................................................................  1     1  seq
....................uuagugcgcaguaagcuaggCugu......................................................................  1     1  seq
....................uuGgugcgcaguaagcuagggugu......................................................................  3     1  seq
....................uuagugcgcaguaagcuagggGgu......................................................................  2     1  seq
....................uuagugcgcaguaagcuagggugu......................................................................  715   0  seq
....................uuagugcgcaguaagcuaggguguU.....................................................................  22    1  seq
....................uuagugcgcaguaagcuaggguguA.....................................................................  2     1  seq
.....................uagugcgcaguaagcuagggug.......................................................................  1     0  seq
.......................................................acgcuagcugcucugcacuaa......................................  1     0  seq
.......................................................acgcuagcugcucugcacuaac.....................................  1     0  seq
........................................................cgcuagcugcucugcacu........................................  4     0  seq
........................................................cgcuagcugcucugcacuaa......................................  5     0  seq
........................................................cgcuagcugcucugcacuaU......................................  1     1  seq
........................................................cgcuaUcugcucugcacuaac.....................................  1     1  seq
........................................................cgcuagcugcucugcacuaac.....................................  12    0  seq
........................................................cgcuagcugcucugUacuaacu....................................  1     1  seq
........................................................cgcuGgcugcucugcacuaacu....................................  1     1  seq
........................................................cgcuagcugcucugcacuaacA....................................  1     1  seq
........................................................cgcuagcugcucugcacuaacu....................................  154   0  seq
........................................................cgcuagcugcucugcacuaacuc...................................  2     0  seq
........................................................cgcuagcugcucugcacuaacucU..................................  5     1  seq
..........................................................cuagcugcucugcacuaa......................................  1     0  seq
..........................................................cuagcugcucugcacuaac.....................................  2     0  seq
..........................................................cuagcugcucugcacuaacu....................................  54    0  seq
..........................................................cuagcugcucugcacuaacuc...................................  2     0  seq
..........................................................cuagcugcucugcacuaacucU..................................  86    1  seq
..........................................................cuagcugcucugcacuaacucc..................................  13    0  seq
..........................................................cuagcugcucugcacuaacuccA.................................  1     1  seq
..........................................................cuagcugcucugcacuaacuccU.................................  14    1  seq
```

Provisional ID : mun-miR-363
Score total : 4.7
Score for star read(s) : -1.3
Score for read counts : 0
Score for mfe : 1.4
Score for randfold : 1.6
Score for cons. seed : 3
Total read count : 7630
Mature read count : 7624
Loop read count : 0
Star read count : 6

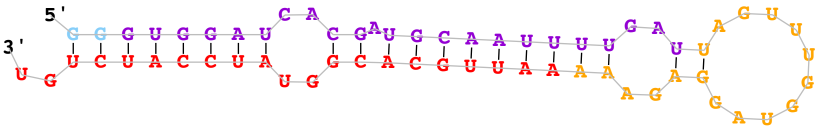

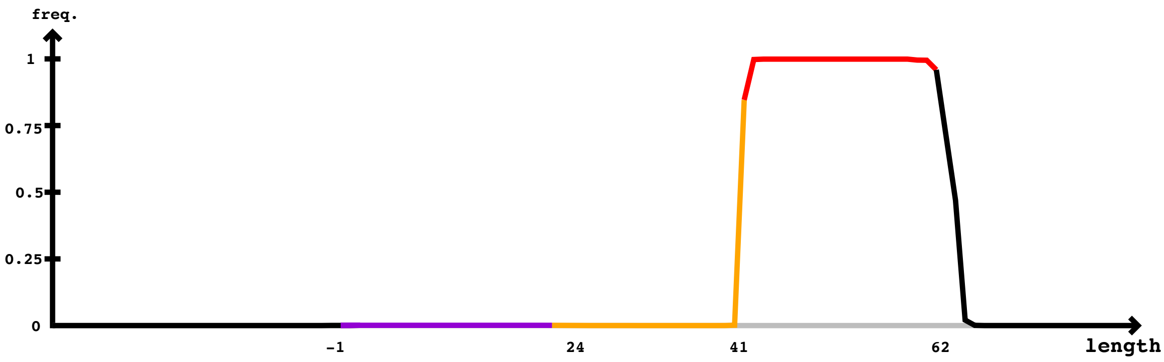

Star Mature

```
5'- cuuugcugagcucuguuuugcuguugucggguggaucacgaugcaauuuugauuaguuugguaggagaaaaauugcacgguauccaucuguaaaccgcaggaccuucaucg  -3'  obs
    cuuugcugagcucuguuuugcuguugucgggguggaucacgaugcaauuuugauuaguuugguaggagaaaaauugcacgguauccaucuguaaaccgcaggaccuucaucg        exp
    .......((.....(((((((.(((..(((((((((..((.(((((((((..((..........))..)))))))))))..)))))))))..))).))))))).....)).   reads   mm   sample
    ...........................cggguggaucacgaugcaauuuu.............................................................   1       0    seq
    ...........................cggguggaucacgaugcaauuuug............................................................   1       0    seq
    ...........................cggguggaucacgaugcaauuuugauuaguuu....................................................   1       0    seq
    ..............................guggaucacgaugcaauuuug............................................................   1       0    seq
    ..............................guggaucacgaugcaauuuugau..........................................................   3       0    seq
    ....................................................................aaaauugcacgguauccaucu......................   1       0    seq
    .....................................................................aaauugcacgguauccaucug.....................   6       0    seq
    .....................................................................aaauugcacgguauccaucugu....................   3       0    seq
    ......................................................................aauugcacgguauccauc.......................   26      0    seq
    ......................................................................aauugcacggCauccauc.......................   1       1    seq
    ......................................................................aauugcacgguauccaucu......................   4       0    seq
    ......................................................................aauugcacgguauccaucuU.....................   2       1    seq
    ......................................................................aauugcacCguauccaucug.....................   1       1    seq
    ......................................................................aauuUcacgguauccaucug.....................   1       1    seq
    ......................................................................aauugcacgguauccaucug.....................   242     0    seq
    ......................................................................aauugcacgguUuccaucug.....................   2       1    seq
    ......................................................................aauugcacggGauccaucug.....................   1       1    seq
    ......................................................................aauugcacgguaucUaucugu....................   2       1    seq
    ......................................................................aauugcacgguauccaucugA....................   3       1    seq
    ......................................................................aGuugcacgguauccaucugu....................   1       1    seq
    ......................................................................aauugcacggCauccaucugu....................   2       1    seq
    ......................................................................aauugcacgguauccaAcugu....................   1       1    seq
    ......................................................................aauugcacgguauccaucugG....................   8       1    seq
    ......................................................................aauugcacgUuauccaucugu....................   1       1    seq
    ......................................................................aauugcacgguauccaucuUu....................   4       1    seq
    ......................................................................aauugcacggGauccaucugu....................   4       1    seq
    ......................................................................aauugcacgguauccaucugu....................   2851    0    seq
    ......................................................................aauugcacAguauccaucugu....................   1       1    seq
    ......................................................................aauugcaGgguauccaucugu....................   1       1    seq
    ......................................................................aauugcacgguauUcaucugu....................   3       1    seq
    ......................................................................aauugcacgguGuccaucugu....................   1       1    seq
    ......................................................................aauugcaUgguauccaucugu....................   2       1    seq
    ......................................................................aaCugcacgguauccaucugu....................   1       1    seq
```

Star Mature

```
cuuugcugagcucuguuuugcuguugucggguggaucacgaugcaauuuugauuaguuugguaggagaaaaauugcacgguauccaucuguaaaccgcaggaccuucaucg
......................................................................Gauugcacgguauccaucugu....................    4     1   seq
......................................................................aauugcacggAauccaucugu....................    4     1   seq
......................................................................aauugcacUguauccaucugu....................    2     1   seq
......................................................................aauugcacgguauccaucugAa...................    4     1   seq
......................................................................aGuugcacgguauccaucugua...................    1     1   seq
......................................................................aauugcacggGauccaucugua...................    1     1   seq
......................................................................aauugcacgguauccaAcugua...................    1     1   seq
......................................................................aauugcacgguauccaucugua...................    388   0   seq
......................................................................aauugcacgguauccaucuguU...................    85    1   seq
......................................................................Gauugcacgguauccaucugua...................    1     1   seq
......................................................................aauugcacgguaucUaucugua...................    1     1   seq
......................................................................aauugcacggCauccaucugua...................    1     1   seq
......................................................................aauugcacgguauccaucuguC...................    2     1   seq
......................................................................aauugcacgguauccaucuguaG..................    51    1   seq
......................................................................aauugcacgguauccaucuguaU..................    1675  1   seq
......................................................................aauugcacggGauccaucuguaa..................    4     1   seq
......................................................................aUuugcacgguauccaucuguaa..................    1     1   seq
......................................................................aauugcacgguauccaucuguaa..................    913   0   seq
......................................................................aauugcacgguauccaucuguaC..................    24    1   seq
......................................................................aauugcacggAauccaucuguaa..................    1     1   seq
......................................................................aauugcacgguauccaucuguGa..................    1     1   seq
......................................................................aauugcacgguauccaucuguUa..................    3     1   seq
......................................................................aauugcacgguaucAaucuguaa..................    1     1   seq
......................................................................aauugcacgguaucUaucuguaa..................    1     1   seq
......................................................................aauugcacgguauccaucuguaUa.................    1     1   seq
......................................................................aauugcacgguauccaucuguaaa.................    25    0   seq
......................................................................aauugcacgguauccaucuguaaU.................    76    1   seq
......................................................................aauugcacgguauccaucuguaGa.................    3     1   seq
......................................................................aauugcacgguauccaucuguaaC.................    1     1   seq
......................................................................aauugcacgguauccaucuguaaaU................    5     1   seq
.......................................................................auugcacgguauccaucug.....................    20    0   seq
.......................................................................auugcacggGauccaucugu....................    1     1   seq
.......................................................................auugcacgCuauccaucugu....................    1     1   seq
.......................................................................auugcacgguauccaucugA....................    1     1   seq
.......................................................................auugcacgguauccaucuAu....................    1     1   seq
.......................................................................auugcacgguauccaucugu....................    274   0   seq
.......................................................................auugcacgguauccaucuguU...................    9     1   seq
.......................................................................auugcacgguauccaucugua...................    64    0   seq
.......................................................................auugcacgguauccaucuguaa..................    249   0   seq
.......................................................................auugcacgguauccaucuguaU..................    468   1   seq
.......................................................................auugcacgguauccaucuguaC..................    10    1   seq
.......................................................................auugcacgguauccaucuguaG..................    9     1   seq
.......................................................................Uuugcacgguauccaucuguaa..................    1     1   seq
.......................................................................auugcacgguUuccaucuguaa..................    1     1   seq
.......................................................................auugcacggAauccaucuguaa..................    1     1   seq
.......................................................................auugcacgguauccauAuguaa..................    1     1   seq
.......................................................................auugcacggGauccaucuguaa..................    1     1   seq
.......................................................................auugcacgguauccaucuguaUa.................    1     1   seq
.......................................................................auugcacgguauccaucuguaaa.................    10    0   seq
.......................................................................auugcacgguauccaucuguaaC.................    3     1   seq
.......................................................................auugcacgguauccaucuguaCa.................    1     1   seq
.......................................................................auugcacgguauccaucuguaaG.................    1     1   seq
.......................................................................auugcacgguauccaucuguaGa.................    1     1   seq
.......................................................................auugcacgguauccaucuguaaU.................    26    1   seq
.......................................................................auugcacgguauccaucuguaaaU................    1     1   seq
........................................................................uugcacgguauccaucuguaU..................    9     1   seq
........................................................................uugcacgguauccaucuguaaU.................    2     1   seq
```

Provisional ID	:	mun-miR-301
Score total	:	4.4
Score for star read(s)	:	-1.3
Score for read counts	:	0
Score for mfe	:	1.1
Score for randfold	:	1.6
Score for cons. seed	:	3
Total read count	:	8109
Mature read count	:	8095
Loop read count	:	0
Star read count	:	14

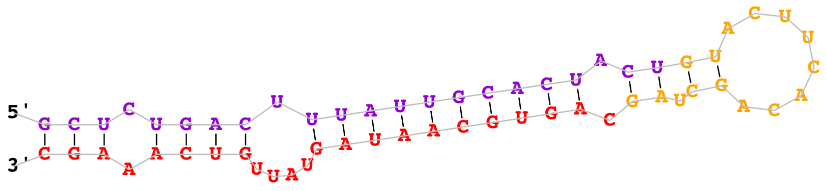

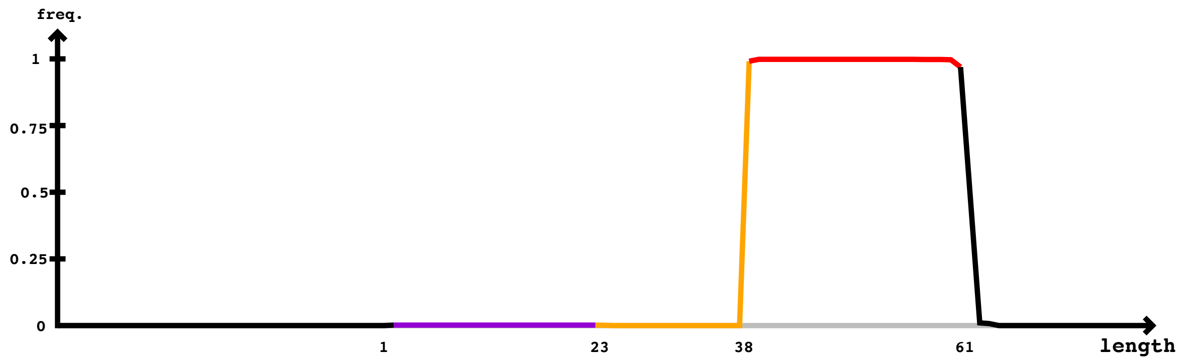

Star **Mature**

```
5'- augacuuguacagaauucuugcugcuaacgaaugcucugacuuuauugcacuacuguacuucacagcuagcagugcaauaguauugucaaagcaucugaaagcagagucuca   -3'   obs
    augacuuguacagaauucuugcugcuaacgaaugcucugacuuuauugcacuacuguacuucacagcuagcagugcaauaguauugucaaagcaucugaaagcagagucuca         exp
    ..(((......((....))..(((((..((.(((((.((((.((((((((((.((((........)).)).))))))))))....)))).))))).))..))))).)))...    reads   mm   sample
    .................................gcucugacuuuauugcacuacu.........................................................    14      0    seq
    ......................................................................cagugcaauaguauuguc........................    4       0    seq
    ......................................................................cagugcaauaguauugucU.......................    1       1    seq
    ......................................................................cagugcaauaguauugucaaa.....................    8       0    seq
    ......................................................................cagugcaauaguauuUucaaag....................    1       1    seq
    ......................................................................cagugUaauaguauugucaaag....................    1       1    seq
    ......................................................................cagugcaauagCauugucaaag....................    1       1    seq
    ......................................................................cagugcaauaguauugucaaag....................    220     0    seq
    ......................................................................cagugcaauaguauugucaGagc...................    3       1    seq
    ......................................................................Aagugcaauaguauugucaaagc...................    6       1    seq
    ......................................................................cagugcaauaguauugAcaaagc...................    2       1    seq
    ......................................................................Uagugcaauaguauugucaaagc...................    2       1    seq
    ......................................................................cagugUaauaguauugucaaagc...................    1       1    seq
    ......................................................................cagugcaauaguGuugucaaagc...................    1       1    seq
    ......................................................................caguUcaauaguauugucaaagc...................    2       1    seq
    ......................................................................Gagugcaauaguauugucaaagc...................    5       1    seq
    ......................................................................cagugcaauaguauugucaaagA...................    2       1    seq
    ......................................................................cagugcaauaguauuguAaaagc...................    4       1    seq
    ......................................................................cagugcaauaguauugucaaGgc...................    2       1    seq
    ......................................................................cagugcGauaguauugucaaagc...................    2       1    seq
    ......................................................................cagAgcaauaguauugucaaagc...................    2       1    seq
    ......................................................................cagugcaauaguauugucaaaCc...................    1       1    seq
    ......................................................................cagugcaauaguauugucaaagU...................    4       1    seq
    ......................................................................cagugcaaAaguauugucaaagc...................    1       1    seq
    ......................................................................cagugcaauaguauuAucaaagc...................    1       1    seq
    ......................................................................cagugcaauaguauCgucaaagc...................    1       1    seq
    ......................................................................cagugcaauagAauugucaaagc...................    4       1    seq
    ......................................................................cagugcaauaguauAgucaaagc...................    1       1    seq
    ......................................................................cagGgcaauaguauugucaaagc...................    2       1    seq
    ......................................................................cagugcaauagCauugucaaagc...................    4       1    seq
    ......................................................................cagugcaauagGauugucaaagc...................    3       1    seq
    ......................................................................cagCgcaauaguauugucaaagc...................    1       1    seq
    ......................................................................cagugcaauaguaCugucaaagc...................    1       1    seq
```

Star Mature

```
augacuuguacagaauucuugcugcuaacgaaugcucugacuuuauugcacuacuguacuucacagcuagcagugcaauaguauugucaaagcaucugaaagcagagucuca
......................................................................cagugcaauaguauugucaaagc...................  7260  0  seq
......................................................................cagugcaauaUuauugucaaagc...................  1  1  seq
......................................................................caUugcaauaguauugucaaagc...................  2  1  seq
......................................................................cagugcaauaguauugucaaagcG..................  2  1  seq
......................................................................cagugcaauagGauugucaaagca..................  1  1  seq
......................................................................cagugcaauaguauugucaaagcU..................  372  1  seq
......................................................................cagugcaauaguauugucaaagca..................  27  0  seq
......................................................................cagugUaauaguauugucaaagca..................  1  1  seq
......................................................................cagugcaauaguauugucaaagcUu.................  3  1  seq
......................................................................cagugcaauaguauugucaaagcaC.................  1  1  seq
......................................................................cagugcaauaguauugucaaagcau.................  17  0  seq
......................................................................cagugcaauaguauugucaaagcauA................  1  1  seq
......................................................................cagugcaauaguauugucaaagcauc................  1  0  seq
......................................................................cagugcaauaguauugucaaagcauU................  55  1  seq
.......................................................................agugcaauaguauugucaaagc...................  46  0  seq
.......................................................................agugcaauaguauugucaaagca..................  2  0  seq
.......................................................................agugcaauaguauugucaaagcU..................  5  1  seq
.......................................................................agugcaauaguauugucaaagcau.................  1  0  seq
.......................................................................agugcaauaguauugucaaagcauU................  3  1  seq
```

Provisional ID	:	mun-novel71
Score total	:	79.3
Score for star read(s)	:	3.9
Score for read counts	:	73
Score for mfe	:	1.4
Score for randfold	:	1.6
Score for cons. seed	:	-0.6
Total read count	:	155
Mature read count	:	111
Loop read count	:	0
Star read count	:	44

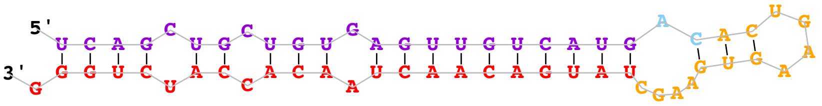

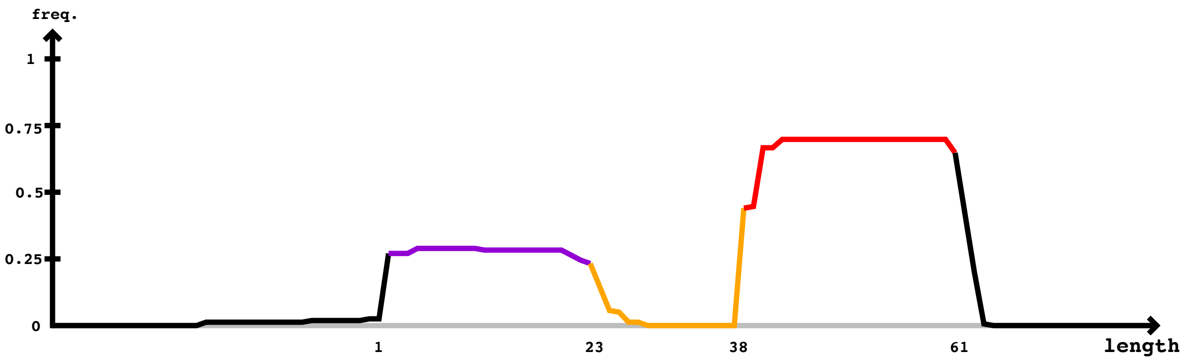

Star Mature

```
5'- aauauucaacgugacgaaauggaguggaggcccucagcugcugugaguugucaugacacugaagugaagcuaugacaacuaacaccaucugggaucuuccugucuucccacau  -3'   obs
    aauauucaacgugacgaaauggaguggaggcccucagcugcugugaguugucaugacacugaagugaagcuaugacaacuaacaccaucugggaucuuccugucuucccacau         exp
    ..........(((..((...(((..(((((..(((((.((.(((.((((((((((.(((....)))....)))))))))).))).)).)))))..)))))..))))).)))..        reads   mm   sample
    ..............cgaaauggaguggaggccc................................................................................        2       0    seq
    .........................ggaggcccucagGugcug......................................................................        1       1    seq
    ...............................ccucagcugcugugaguuguc.............................................................        1       0    seq
    .................................ucagcugcugugaguuguc.............................................................        2       0    seq
    .................................ucagcugcugugaguuguca............................................................        3       0    seq
    .................................ucagcugcugugaguugucau...........................................................        2       0    seq
    .................................ucagcugcugugaguugucaug..........................................................        14      0    seq
    .................................Gcagcugcugugaguugucaug..........................................................        1       1    seq
    .................................ucagcugcugugaguugucauga.........................................................        3       0    seq
    .................................ucagcugcugugaguugucaugU.........................................................        10      1    seq
    .................................ucagcugcugugaguugucaugacU.......................................................        4       1    seq
    .................................ucagcugcugugaguugucaugacacu.....................................................        2       0    seq
    ....................................gcugcugugaguugucaugac........................................................        1       0    seq
    ....................................gcugcugugaguugucaugacU.......................................................        1       1    seq
    ....................................gcugcugugaguugucaugaca.......................................................        1       0    seq
    ......................................................................uaugacaacuaacaccaucugg.....................        8       0    seq
    ......................................................................uaugacaacuaacaccaucuggg....................        39      0    seq
    ......................................................................uaugacaacuaacaccaucuggC....................        1       1    seq
    ......................................................................uaugacaacuaacaccaucuggU....................        10      1    seq
    ......................................................................uaugacaacuaacaccaucuggA....................        1       1    seq
    ......................................................................uaugacaacuaacaAcaucuggga...................        1       1    seq
    ......................................................................uaugacaacuaacaccaucuggga...................        6       0    seq
    ......................................................................uaugacaacuaacaccaucugggaA..................        3       1    seq
    ......................................................................uaugacaacuaacaccaucugggau..................        1       0    seq
    .......................................................................augacaacuaacaccaucuggU....................        1       1    seq
    ........................................................................ugacaacuaacaccaucugggU...................        4       1    seq
    ........................................................................ugacaacuaacaccaucuggga...................        8       0    seq
    ........................................................................ugacaacuaacaccaucugggau..................        23      0    seq
    ..........................................................................acaacuaacaccaucugggau..................        4       0    seq
    ..........................................................................acaacuaacaccaucugggauc.................        1       0    seq
```

Provisional ID	:	mun-miR-1788
Score total	:	17
Score for star read(s)	:	3.9
Score for read counts	:	6.2
Score for mfe	:	2.3
Score for randfold	:	1.6
Score for cons. seed	:	3
Total read count	:	24
Mature read count	:	19
Loop read count	:	0
Star read count	:	5

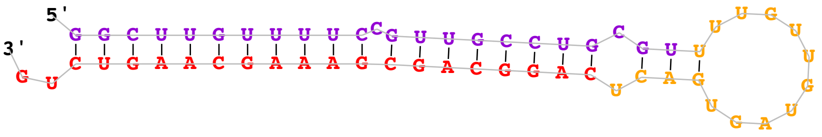

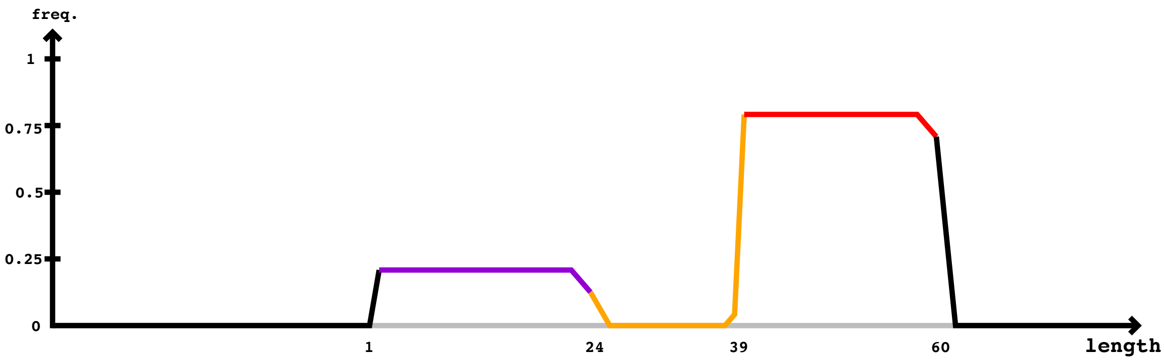

Star **Mature**

```
5'- gcccgcuccccaccucccgccgcugucucuggggcuuguuuuccguugccugcguuuuguuguagugacucaggcagcgaaagcaagucugggaggcugcggagccacugc   -3'   obs
    gcccgcuccccaccucccgccgcugucucuggggcuuguuuuccguugccugcguuuuguuguagugacucaggcagcgaaagcaagucugggaggcugcggagccacugc         exp
    ....(((((.((((((((.(((.......)))((((((((((.((((((((.(((..........))).))))))))))))))))))).)))))).)).))))).....   reads   mm   sample
    ................................ggcuuguuuuccguugccugc..........................................................   1       0    seq
    ................................ggcuuguuuuccguugccugcg.........................................................   1       0    seq
    ................................ggcuuguuuuccguugccugcgu........................................................   3       0    seq
    .....................................................................ucaggcagcgaaagcaagucug....................   1       0    seq
    ......................................................................caggcagcgaaagcaaguc......................   1       0    seq
    ......................................................................caggcagcgaaagcaagucu.....................   1       0    seq
    ......................................................................caggcagcgaaagcaagucug....................   14      0    seq
    ......................................................................caggcagcgaaagcaagucugU...................   2       1    seq
```

Provisional ID	:	mun-novel83
Score total	:	38.1
Score for star read(s)	:	3.9
Score for read counts	:	27.7
Score for mfe	:	2
Score for randfold	:	1.6
Score for cons. seed	:	3
Total read count	:	66
Mature read count	:	60
Loop read count	:	0
Star read count	:	6

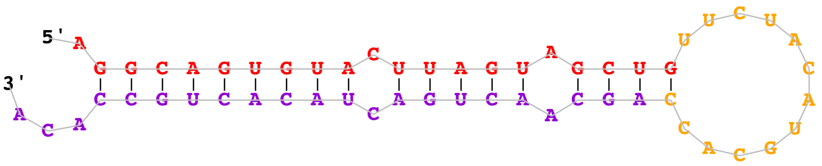

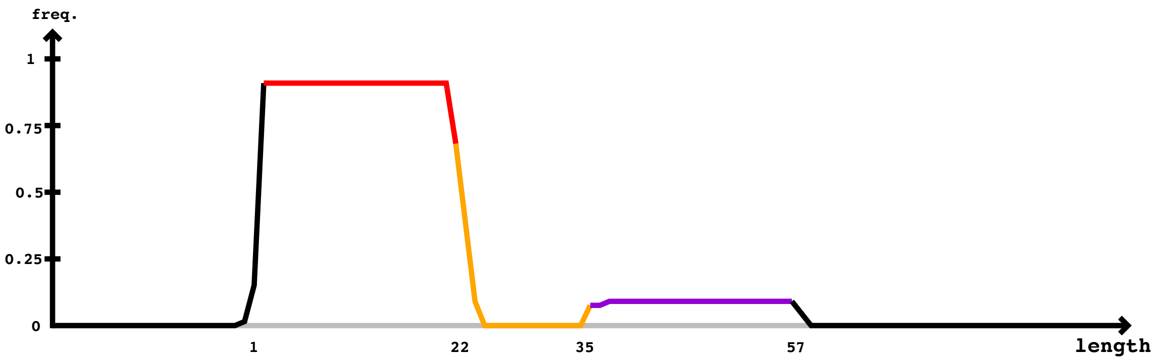

Mature **Star**

```
5'- ugcugcuguguguggugauuaggcaguguacuuaguagcuguucuacaugcaccagcaacugacuacacugccacauuaccacaucacugcugagacaagaauauuauca   -3'   obs
    ugcugcuguguguggugauuaggcaguguacuuaguagcuguucuacaugcaccagcaacugacuacacugccacauuaccacaucacugcugagacaagaauauuauca         exp
    ....((.((((((((((((..(((((((((.(((((.((((............)))).))))).)))))))))..))))))))).))).))...................   reads   mm   sample
    ..................uuaggcaguguacuuaguagcu......................................................................   1       0    seq
    ...................uaggcaguguacuuaguagcu......................................................................   4       0    seq
    ...................uaggcaguguacuuaguagcA......................................................................   1       1    seq
    ...................uaggcaguguacuuaguagcug.....................................................................   4       0    seq
    ....................aggcaguguacuuaguagcu......................................................................   9       0    seq
    ....................aggcaguguacuuaguagcug.....................................................................   31      0    seq
    ....................aggcaguguacuuaguagcugu....................................................................   4       0    seq
    ....................aggcaguguacuuaguagcuguA...................................................................   2       1    seq
    ....................aggcaguguacuuaguagcuguu...................................................................   4       0    seq
    ......................................................agcaacugacuacacugccaca..................................   5       0    seq
    ........................................................caacugacuacacugccacaA.................................   1       1    seq
```

Provisional ID	:	mun-miR-29b
Score total	:	4.9
Score for star read(s)	:	-1.3
Score for read counts	:	0
Score for mfe	:	1.6
Score for randfold	:	1.6
Score for cons. seed	:	3
Total read count	:	603
Mature read count	:	587
Loop read count	:	0
Star read count	:	16

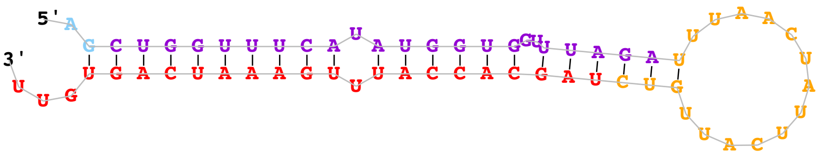

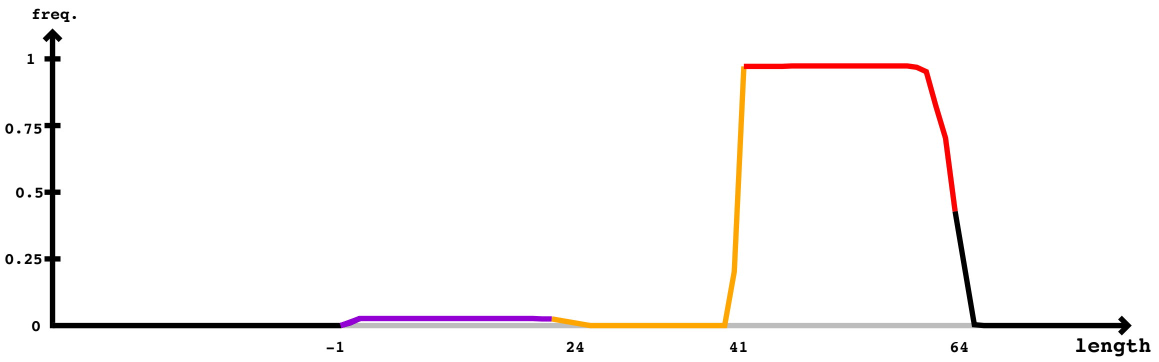

Star **Mature**

```
5'- ucccagcuggaaauaagcucccucaggaagcugguuucauauggugguuuagauuuaacuauucauuguc uagcaccauuugaaaucaguguucuuggaggaagaaugcc -3'   obs
    ucccagcuggaaauaagcucccucaggaagcugguuucauauggugguuuagauuuaacuauucauugucuagcaccauuugaaaucaguguucuuggaggaagaaugcc        exp
    ....((((.......))))((..(((((((((((((((.((((((..((((((.............))))))))))))).)))))))))).))))))..)).........   reads   mm   sample
    ............................gcugguuucauauggugguuua............................................................   1   0   seq
    ............................gcugguuucauauggugguuuag...........................................................   1   0   seq
    ............................gcugguuucauauggugguuuaga..........................................................   3   0   seq
    ............................gcuUguuucauauggugguuuaga..........................................................   1   1   seq
    ..............................cugguuucauauggugguu.............................................................   1   0   seq
    ..............................cugguuucauauggugguuuag..........................................................   1   0   seq
    ..............................cugguuucauauggugguuuagU.........................................................   3   1   seq
    ..............................cugguuucauauggugguuuaga.........................................................   2   0   seq
    ..............................cugguuucauauggugguuuagau........................................................   3   0   seq
    .....................................................................cuagcaccauuugaaauca......................   2   0   seq
    .....................................................................cuagcaccauuugaaaucag.....................   8   0   seq
    .....................................................................cuagcaccauuugaaaucaA.....................   1   1   seq
    .....................................................................cuagcacUauuugaaaucagu....................   1   1   seq
    .....................................................................cuagcaccauuugaaaucagG....................   1   1   seq
    .....................................................................cuagcaccauuugaaaucagu....................   40  0   seq
    .....................................................................cuagcaccauuugaaaucaguC...................   2   1   seq
    .....................................................................cuagcaccauuugaaaucaguA...................   1   1   seq
    .....................................................................cuagcaccauuugaaaucaguU...................   3   1   seq
    .....................................................................cuagcaccauuugaaaucagug...................   30  0   seq
    .....................................................................cuagcaccauuugaaaucagugA..................   12  1   seq
    .....................................................................Auagcaccauuugaaaucagugu..................   1   1   seq
    .....................................................................cuagcaccauuugaaaucagugu..................   18  0   seq
    .....................................................................cuagcaccauuugaaaucagugG..................   1   1   seq
    .....................................................................cuagcaccauuugaaaucaguguu.................   1   0   seq
    ......................................................................uagcaccauuugaaauca......................   1   0   seq
    ......................................................................uagcaccauuugaaaucag.....................   1   0   seq
    ......................................................................uagcaccauuugaaaucagA....................   1   1   seq
    ......................................................................uagcaccauuugaaaucagu....................   35  0   seq
    ......................................................................uagcaccGuuugaaaucagug...................   1   1   seq
    ......................................................................uagcaccauuugaaaucaguC...................   5   1   seq
    ......................................................................uagcaccauuugaaaucagug...................   24  0   seq
    ......................................................................uagcaccauuugaaaucGgug...................   3   1   seq
    ......................................................................uagcaccauuugaaaucaguU...................   3   1   seq
```

Star Mature

ucccagcuggaaauaagcucccucaggaagcugguuucauauggugguuuagauuuaacuauucauugucuagcaccauuugaaaucaguguucuuggaggaagaaugcc			
....uagcaccauuugaaaucaguUu..................	4	1	seq
....uagcaccauuugaaaucagugC..................	2	1	seq
....uagcaccauuugaaaucagugG..................	5	1	seq
....uagcaccauuugaaaucagugu..................	110	0	seq
....uagcaccauuugaaaucagugA..................	9	1	seq
....uagcaccauuugaaaucaguCu..................	3	1	seq
....uagcaccauuugaaaucCgugu..................	1	1	seq
....uagcaccauuugaaaucaguguu.................	248	0	seq
....uagcaccauuugaaaucaguguA.................	3	1	seq
....uagcaccauuugaaaucagugAu.................	1	1	seq
....uagcaccauuugaaaucaguguuc................	1	0	seq
....uagcaccauuugaaaucaguguuU................	1	1	seq
....uagcaccauuugaaaucaguguuGu...............	1	1	seq
....uagcaccauuugaaaucaguguuAu...............	1	1	seq
....ccauuugaaaucaguguu.................	1	0	seq

Provisional ID	:	mun-miR-184-1
Score total	:	5
Score for star read(s)	:	-1.3
Score for read counts	:	0
Score for mfe	:	1.7
Score for randfold	:	1.6
Score for cons. seed	:	3
Total read count	:	265
Mature read count	:	265
Loop read count	:	0
Star read count	:	0

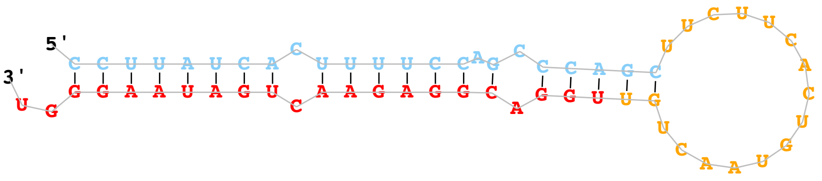

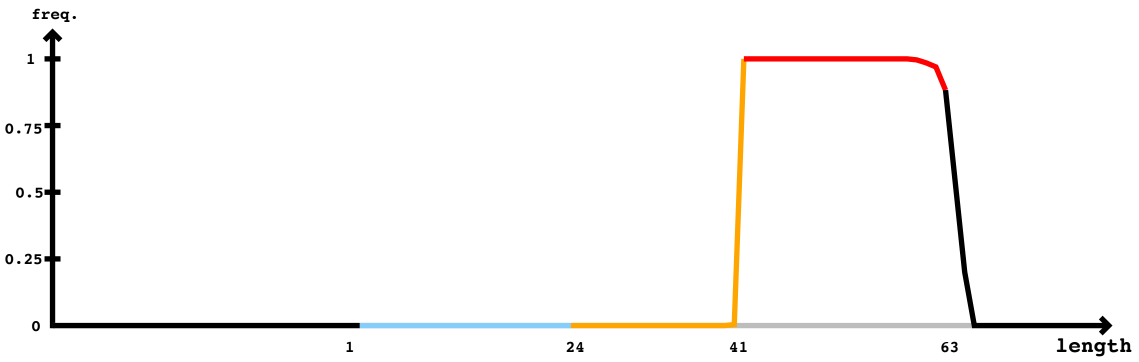

Star **Mature**

```
5'- ggcagacgucuccugaggccgcucucaucuccuuaucacuuuuccagcccagcuucuucacuguaacuguuggacggagaacugauaagggugaugcgagaggcacgag   -3'    exp
    ((.((....))))....(((.(((.(((..((((((((.((((((.(.(((((...............))))).))))))).))))))))..))).))).)))..... reads     mm       sample
    .....................................................................uuggacggagaacugauaaggU.................  1         1        seq
    ......................................................................uggacggagaacugauaa....................  1         0        seq
    ......................................................................uggacggagaacugauaaA...................  2         1        seq
    ......................................................................uggacggagaacugauaag...................  1         0        seq
    ......................................................................uggacggagaacugauaagA..................  1         1        seq
    ......................................................................uggacggagaacugauaagg..................  3         0        seq
    ......................................................................uggacggagaacugauaaggA.................  1         1        seq
    ......................................................................uggacggagaacugauaaggU.................  2         1        seq
    ......................................................................uggacggagaacugauaaggg.................  19        0        seq
    ......................................................................uggacggaUaacugauaagggu................  1         1        seq
    ......................................................................uggacggagaacugauaagggA................  10        1        seq
    ......................................................................uggacggagaacugauaagggC................  1         1        seq
    ......................................................................uggacggagaacugauaagggu................  109       0        seq
    ......................................................................uggacggagaacugauaaggguU...............  41        1        seq
    ......................................................................uggacggagaacugauaagggAa...............  2         1        seq
    ......................................................................uggacggagaacugauaagggCa...............  1         1        seq
    ......................................................................uggacggagaacugauaagggua...............  16        0        seq
    ......................................................................uggacggagaacugauaaggguau..............  38        0        seq
    ......................................................................uggacggagaacugauaaggguUu..............  12        1        seq
    ......................................................................uggacggagaacugauaaggguaA..............  3         1        seq
```

Provisional ID	:	mun-novel58
Score total	:	32.2
Score for star read(s)	:	3.9
Score for read counts	:	25.1
Score for mfe	:	2.2
Score for randfold	:	1.6
Score for cons. seed	:	-0.6
Total read count	:	61
Mature read count	:	39
Loop read count	:	0
Star read count	:	22

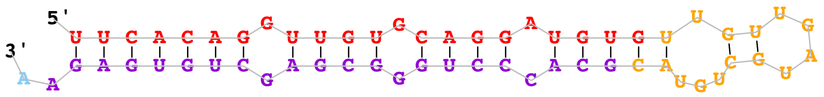

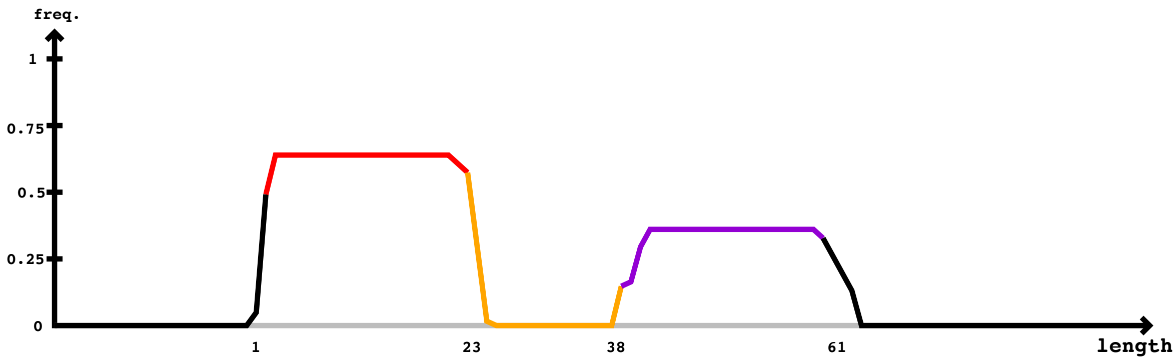

Mature   Star

```
5'- aucucugcaugugcaggcuuuucacagguugugcaggauguguuguugaugcuguacgcacccugggcgagcugugagaagccugccucaaguucagccucaggagccuuuc  -3'   obs
    aucucugcaugugcaggcuuuucacagguugugcaggauguguuguugaugcuguacgcacccugggcgagcugugagaagccugccucaaguucagccucaggagccuuuc         exp
    ..((((((.((.(((((((((((((((.((((.((((.(((((.((....))...))))).)))).)))).)))))))))))))))..)).))).........))))......   reads   mm   sample
    ...................uuucacagguugugcaggaug........................................................................   1   0   seq
    ...................uuucacaggCugugcaggaugu.......................................................................   1   1   seq
    ...................uuucacagguugugcaggauguAu.....................................................................   1   1   seq
    ....................uucacagguugugcaggaug........................................................................   1   0   seq
    ....................uucacagguugugcaggaugu.......................................................................   1   0   seq
    ....................uucacaggCugugcaggaugug......................................................................   1   1   seq
    ....................uucacagguugugcaggaugug......................................................................   10   0   seq
    ....................uucacagguugugcaggauguU......................................................................   1   1   seq
    ....................uucacagguugugcaggaugugA.....................................................................   1   1   seq
    ....................uucacagguugugcaggaugugu.....................................................................   8   0   seq
    ....................uucacagguugugcaggaugugG.....................................................................   3   1   seq
    ....................uucacagguugugcaggauguguu....................................................................   1   0   seq
    .....................ucacagguugugcaggaugugu.....................................................................   9   0   seq
    .........................................................gcacccugggcgagcugugag..................................   2   0   seq
    .........................................................gcacccugggcgagcugugaga.................................   4   0   seq
    .........................................................Acacccugggcgagcugugaga.................................   2   1   seq
    .........................................................gcacccugggcgagcugugagU.................................   1   1   seq
    ..........................................................cacccugggcgagcugugaga.................................   1   0   seq
    ...........................................................acccugggcgagcugugaga.................................   1   0   seq
    ...........................................................acccugggcgagcugugagaaU...............................   1   1   seq
    ...........................................................acccugggcgagcugugagaag...............................   2   0   seq
    ...........................................................acccugggcgagcugugagaagU..............................   4   1   seq
    ............................................................cccugggcgagcugugagaagU..............................   1   1   seq
    ............................................................cccugggcgagcugugagaagc..............................   2   0   seq
    ............................................................cccugggcgagcugugagaagA..............................   1   1   seq
```

Provisional ID : mun-miR-214-1
Score total : 148.7
Score for star read(s) : 3.9
Score for read counts : 141.3
Score for mfe : 2.4
Score for randfold : 1.6
Score for cons. seed : -0.6
Total read count : 289
Mature read count : 266
Loop read count : 0
Star read count : 23

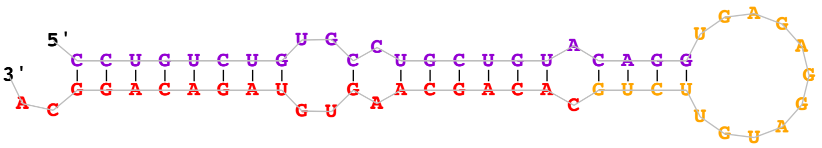

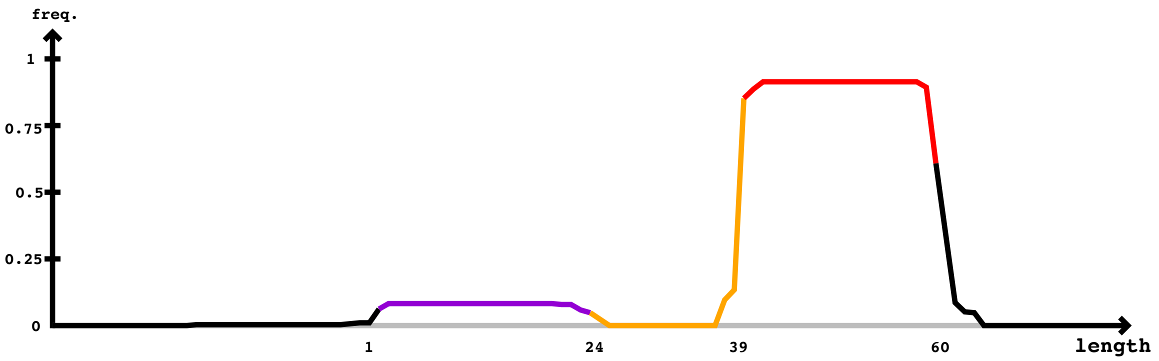

Star Mature

```
5'- gcugucccucaggcuggguugucaugcgacugccugucugugccugcuguacaggugagaggauguucugcacagcaaguguagacaggcagacacaugacaacuccguc  -3'   obs
    gcugucccucaggcuggguugucaugcgacugccugucugugccugcuguacaggugagaggauguucugcacagcaaguguagacaggcagacacaugacaacuccguc         exp
    ...........(((.(((((((((((.(.(((((((((((..(.((((((.((((...........)))).)))))).)..))))))))))).).))))))))))).)))  reads  mm  sample
    .............cuggguugucaugcgacug..............................................................................  1      0   seq
    .............................cugccugucugugccugcuguU...........................................................  1      1   seq
    ..............................ugccugucugugccugcuguacU.........................................................  1      1   seq
    ................................ccugucugugccugcuguaca.........................................................  5      0   seq
    ................................ccugucugugccugcuguacag........................................................  3      0   seq
    ................................ccugucugugccugcuguacagA.......................................................  1      1   seq
    ................................ccugucugugccugcuguacagU.......................................................  6      1   seq
    ................................ccugucugugccugcuguacagUu......................................................  1      1   seq
    .................................cugucugugccugcuguacagg.......................................................  2      0   seq
    .................................cugucugugccugcuguacagU.......................................................  3      1   seq
    .................................cugucugugccugcuguacaggA......................................................  1      1   seq
    ....................................................................ugcacagcaGguguagacagg.....................  1      1   seq
    ....................................................................ugcacagcaaguguagacaggc....................  19     0   seq
    ....................................................................ugcGcagcaaguguagacaggc....................  1      1   seq
    ....................................................................ugcacagcaGguguagacaggc....................  6      1   seq
    ....................................................................ugcacagcaaguguagacaggca...................  1      0   seq
    .....................................................................gcacagcaaguguagacagg.....................  1      0   seq
    .....................................................................gcacagcaaguguagacaggc....................  3      0   seq
    .....................................................................gcacagcaaguguagacaggcC...................  1      1   seq
    .....................................................................gcacagcaaguguagacaggca...................  1      0   seq
    .....................................................................gcacagcaaguguagacaggcaU..................  5      1   seq
    ......................................................................cacagcaaguguagacagg.....................  2      0   seq
    ......................................................................cacagcaGguguagacagg.....................  2      1   seq
    ......................................................................cacagcGaguguagacaggc....................  4      1   seq
    ......................................................................cacGgcaaguguagacaggc....................  2      1   seq
    ......................................................................cacagcaGguguagacaggc....................  6      1   seq
    ......................................................................cacagcaaguguagacaggc....................  42     0   seq
    ......................................................................cacGgcaaguguagacaggca...................  3      1   seq
    ......................................................................cacagcaaguguagacaggca...................  47     0   seq
    ......................................................................cacagcaGguguagacaggca...................  31     1   seq
    ......................................................................cacagcaaguguagacaggcC...................  8      1   seq
    ......................................................................cacagcaaguguagacaggcaA..................  4      1   seq
    ......................................................................cacagcaaguguagacaggcaU..................  37     1   seq
```

```
                                                   Star                                    Mature
gcugucccucaggcuggguugucaugcgacugccugucugugccugcuguacaggugagaggauguucugcacagcaaguguagacaggcagacacaugacaacuccguc
......................................................................cacagcaGguguagacaggcag..................	1	1	seq
......................................................................cacagcaaguguagacaggcaC..................	9	1	seq
......................................................................cacagcaaguguagacaggcGg..................	1	1	seq
......................................................................cacagcaaguguagacaggcag..................	3	0	seq
......................................................................cacagcaaguguagacaggcaga.................	1	0	seq
......................................................................cacagcaaguguagacaggcaUa.................	4	1	seq
......................................................................cacagcaaguguagacaggcagU.................	2	1	seq
.......................................................................acagcaaguguagacaggcagU.................	3	1	seq
.......................................................................acagcaaguguagacaggcagacC...............	7	1	seq
........................................................................cagcaGguguagacaggcagac................	1	1	seq
........................................................................cagcaaguguagacaggcagacG...............	1	1	seq
........................................................................cagcaaguguagacaggcagacC...............	5	1	seq
........................................................................cagcaaguguagacaggcagacU...............	1	1	seq
```

Provisional ID :mun-miR-205a
Score total : 736
Score for star read(s) : 3.9
Score for read counts : 725.6
Score for mfe : 1.9
Score for randfold : 1.6
Score for cons. seed : 3
Total read count : 1435
Mature read count : 1433
Loop read count : 0
Star read count : 2

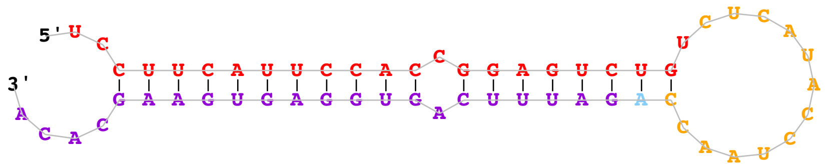

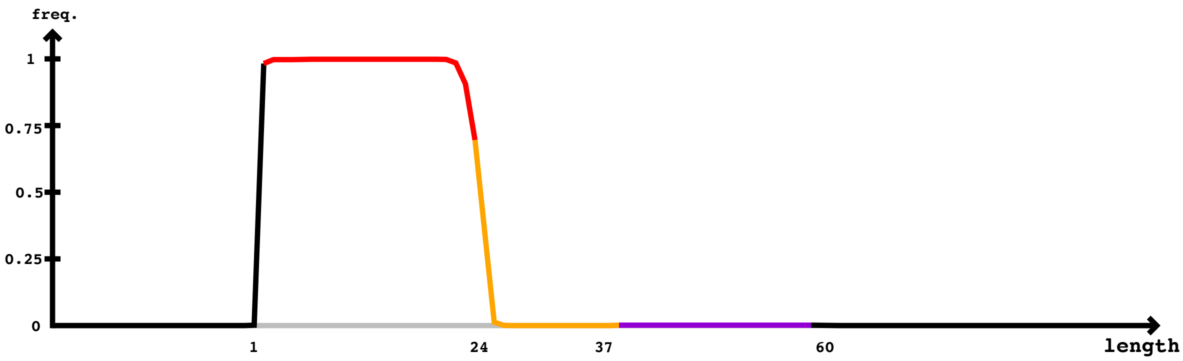

Mature **Star**

```
5'- acgauccauggauucuguuguccuucauuccaccggagucugucucauaccuaaccagauuucaguggagugaagcacaagagacauggagaugaaucagcaacaagagcaac  -3'  obs
    acgauccauggauucuguuguccuucauuccaccggagucugucucauaccuaacccagauuucaguggagugaagcacaagagacauggagaugaaucagcaacaagagcaac       exp
    ....((((((...(((.((((.(((((((((((.((((((((.............)))))))).))))))))))).)))).))))))))).........((.......))...  reads  mm  sample
    ...................guccuucauuccaccggagucug.......................................................................  1      0   seq
    ...................guccuucauuccaccggagucugu......................................................................  1      0   seq
    ....................uccuucauuccaccggagu..........................................................................  1      0   seq
    ....................uccuucauuccaccggaguc.........................................................................  19     0   seq
    ....................uccuucauuccaccggagucG........................................................................  3      1   seq
    ....................uccuucauuccaUcggagucu........................................................................  1      1   seq
    ....................uccuucauuUcaccggagucu........................................................................  1      1   seq
    ....................uccuucauuccacAggagucu........................................................................  1      1   seq
    ....................uccuucauuccaccggagucA........................................................................  3      1   seq
    ....................uccuucauuccaccggagucu........................................................................  103    0   seq
    ....................uccuucauuccaccggagGcug.......................................................................  1      1   seq
    ....................uccuucauuccaccggaUucug.......................................................................  1      1   seq
    ....................uccuucauuccaccggagucug.......................................................................  279    0   seq
    ....................uccuucauuccaccggagucuA.......................................................................  1      1   seq
    ....................uccuAcauuccaccggagucug.......................................................................  1      1   seq
    ....................Gccuucauuccaccggagucugu......................................................................  1      1   seq
    ....................Accuucauuccaccggagucugu......................................................................  1      1   seq
    ....................uccuucauucUaccggagucugu......................................................................  1      1   seq
    ....................uccuucauuccaccggagucuUu......................................................................  1      1   seq
    ....................uccuucauuccaccggGgucugu......................................................................  2      1   seq
    ....................uccuucauuccaccggagucugG......................................................................  10     1   seq
    ....................uccuucauuccaccggagGcugu......................................................................  1      1   seq
    ....................uccuucauuccaccggagucugC......................................................................  2      1   seq
    ....................uccuucauuccUccggagucugu......................................................................  2      1   seq
    ....................uccuucauuccaccggagucugA......................................................................  65     1   seq
    ....................uccuucauuccaccggagCcugu......................................................................  1      1   seq
    ....................ucUuucauuccaccggagucugu......................................................................  1      1   seq
    ....................uccuucauuccaccggagucugu......................................................................  803    0   seq
    ....................uccuucauuccaccggagucuguU.....................................................................  70     1   seq
    ....................uccuucauuccaccggagucuguA.....................................................................  14     1   seq
    ....................uccuucauuccaccggagucuguUu....................................................................  13     1   seq
    ....................uccuucauuccaccggagucuguAu....................................................................  4      1   seq
    ....................uccuucauuccaccggagucugucuc...................................................................  1      0   seq
```

```
                      Mature                           Star
acgauccauggauucuguuguccuucauuccaccggagucugucucauaccuaaccagauuucaguggagugaagcacaagagacauggagaugaaucagcaacaagagcaac
.....................ccuucauuccaccggaAucug.......................................................................	19	1	seq
.....................ccuucauuccaccggaAucugu......................................................................	2	1	seq
........................ucauuccaccggaAucugu......................................................................	1	1	seq
.........................cauuccaccggagucugA......................................................................	1	1	seq
.........................................................gauuucaguggagugaagcaca..................................	2	0	seq
```

Provisional ID	:	mun-novel68
Score total	:	64.1
Score for star read(s)	:	3.9
Score for read counts	:	61.8
Score for mfe	:	1.2
Score for randfold	:	-2.2
Score for cons. seed	:	-0.6
Total read count	:	133
Mature read count	:	131
Loop read count	:	1
Star read count	:	1

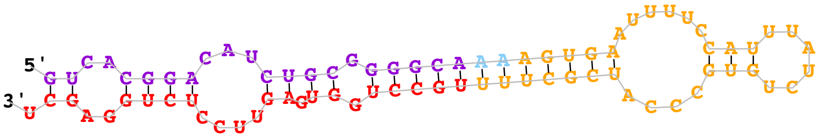

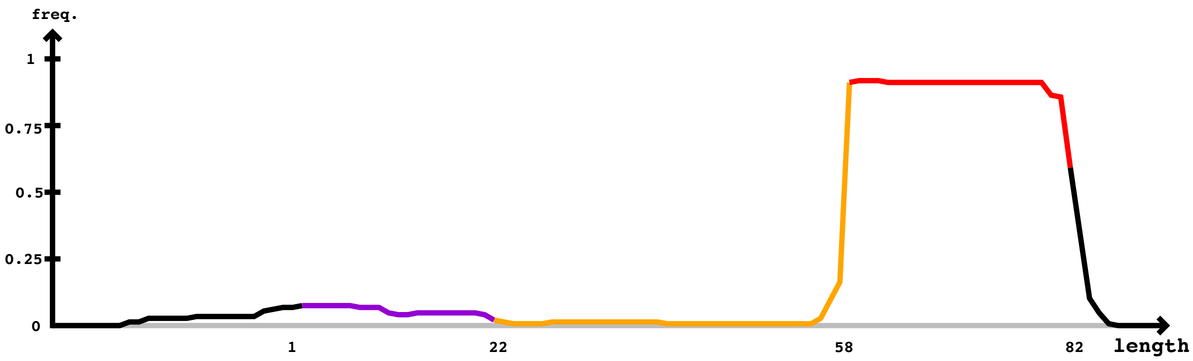

Star **Mature**

```
5'- acucugugucgccgcucagauucagucacggacaucugcggggcaaaagugaauuuuccauuuaucugugcccaucgcuuuugccuggugaguuccucuggagcugcuuucauc  -3'   obs
    acucugugucgccgcucagauucagucacggacaucugcggggcaaaagugaauuuuccauuuaucugugcccaucgcuuuugccuggugaguuccucuggagcugcuuucauc         exp
    ..((((.((....)).))))..((((..((((...((((.((((((((((((......(((......)))....)))))))))))).)).))....))))..))))........   reads   mm   sample
    ......ugucgccgcucagauucagucacg....................................................................................   1       0    seq
    ......ugucUccgcucagauucagucacggac.................................................................................   1       1    seq
    ........ucgccgcucagauucagucacggac.................................................................................   1       0    seq
    ........ucgccgcucagauucagucacggaca................................................................................   1       0    seq
    .............gcucagauucagucacggac.................................................................................   1       0    seq
    ....................uucagucacggacaucugcggggc......................................................................   3       0    seq
    .....................ucagucacggacaucugcgggg.......................................................................   1       0    seq
    ......................cagucacggacaucugcggggca.....................................................................   1       0    seq
    ........................gAcacggacaucugcggggca.....................................................................   1       1    seq
    ....................................ugcggggcaaaagugaauuuuccauu....................................................   1       0    seq
    ..................................................gaauuuuccauuuaucugugcccaucgcuuuugcc.............................   1       0    seq
    ..............................................................................uuuugccuggugaguuccucuggagc..........   3       0    seq
    ...............................................................................uuugccuggugaguuccucugga............   2       0    seq
    ...............................................................................uuugccuggugaguuccucuggagc..........   4       0    seq
    ...............................................................................uuugccuggugaguuccucuggagcG.........   1       1    seq
    ...............................................................................uuugccuggugaguuccucuggagcu.........   3       0    seq
    ................................................................................uugccuggugaguuccucugga............   1       0    seq
    ................................................................................uugccuggugaguuccucuggagc..........   3       0    seq
    ................................................................................uugccuggugaguuccucuggagcu.........   5       0    seq
    ................................................................................uugccuggugaguuccucuggagcug........   1       0    seq
    .................................................................................ugccuggugaguuccucugga............   3       0    seq
    .................................................................................ugccuggugaguuccucuggC............   1       1    seq
    .................................................................................ugccuggugaguuccucuggag...........   1       0    seq
    .................................................................................ugccuggugaguuccucuggagc..........   27      0    seq
    .................................................................................ugccuggugagGuccucuggagc..........   1       1    seq
    .................................................................................ugccuggugaguuccucuggagcG.........   1       1    seq
    .................................................................................ugccuggugaguuccucuggagcu.........   56      0    seq
    .................................................................................ugccuggugaguuccucuggagcug........   2       0    seq
    .................................................................................ugccuggugaguuccucuggagcuU........   3       1    seq
    .................................................................................ugccuggugaguuccucuggagcugc.......   8       0    seq
    .................................................................................ugccuggugaguuccucuggagcugcu......   6       0    seq
    .................................................................................ugccuggugaguuccucuggagcugcuu.....   1       0    seq
    ..................................................................................gccuggugaguuccucuggagc..........   1       0    seq
```

Star **Mature**

acucugugucgccgcucagauucagucacggacaucugcggggcaaaagugaauuuuccauuuaucugugcccaucgcuuuugccuggugaguuccucuggagcugcuuucauc

Provisional ID	:	mun-miR-20b
Score total	:	348.1
Score for star read(s)	:	3.9
Score for read counts	:	337.6
Score for mfe	:	2
Score for randfold	:	1.6
Score for cons. seed	:	3
Total read count	:	674
Mature read count	:	655
Loop read count	:	0
Star read count	:	19

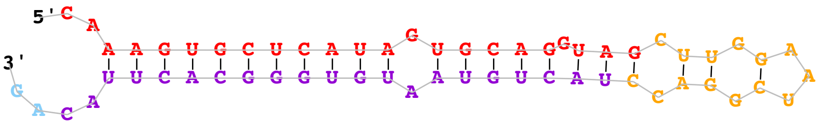

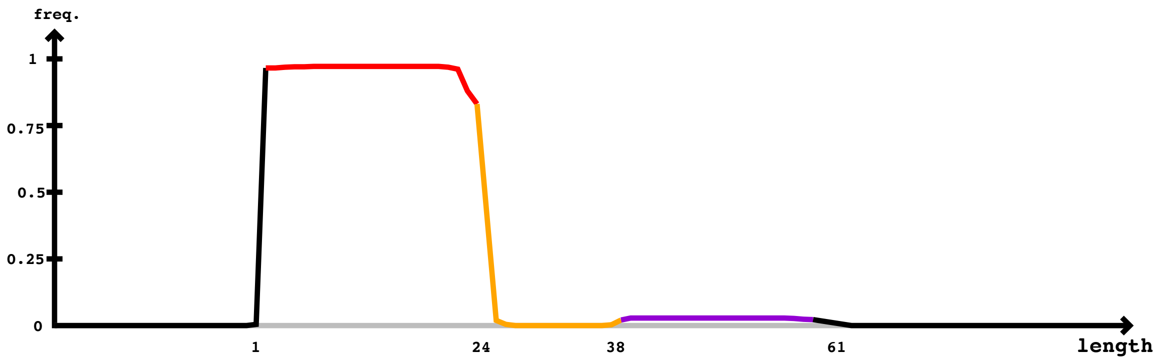

Mature   Star

```
5'- uaguuuuguccuagcaguaucaaagugcucauagugcagguagcuuggaaucggaccuacuguaaugugggcacuuacaguacugcuagauaaagcgcauccuuagcuag -3' obs
    uaguuuuguccuagcaguaucaaagugcucauagugcagguagcuuggaaucggaccuacuguaaugugggcacuuacaguacugcuagauaaagcgcauccuuagcuag     exp
    ..((((((((.(((((((((..(((((((((((.(((((.(((.((.(...).)).)))))))).)))))))))))...)))))))))))))))))..............   reads  mm  sample
    ...................ucaaagugcucauagugcaggu.....................................................................   2      0   seq
    ...................ucaaagugcucauagugcagguaU...................................................................   1      1   seq
    ....................caaagugcucauagugcag.......................................................................   2      0   seq
    ....................caaagugcucauagugcagg......................................................................   5      0   seq
    ....................caaagugcucauagugcaggu.....................................................................   52     0   seq
    ....................caaagugcuUauagugcaggu.....................................................................   1      1   seq
    ....................caaagugcucauagugAaggua....................................................................   1      1   seq
    ....................caaagugcucauagugcagguU....................................................................   1      1   seq
    ....................caaagugcucauagugcaggua....................................................................   31     0   seq
    ....................caaagugcucauagugcagguGg...................................................................   1      1   seq
    ....................caaagugcucauagugcagguag...................................................................   268    0   seq
    ....................caaagugcucauagugcagguUg...................................................................   1      1   seq
    ....................caaagAgcucauagugcagguag...................................................................   1      1   seq
    ....................caaagugcucauagugcagguaU...................................................................   34     1   seq
    ....................Uaaagugcucauagugcagguag...................................................................   2      1   seq
    ....................caaagugcucauagugcagguaA...................................................................   6      1   seq
    ....................caaagugcucauagugcagguagA..................................................................   21     1   seq
    ....................caaagugcucauagugcagguagc..................................................................   4      0   seq
    ....................caaagugcucauagugcagguagU..................................................................   203    1   seq
    ....................caaagugcucauaCugcagguagc..................................................................   1      1   seq
    ....................caaagugcucauagugcagguagAu.................................................................   1      1   seq
    ....................caaagugcucauagugcagguagcu.................................................................   2      0   seq
    ....................caaagugcucauagugcagguagUu.................................................................   7      1   seq
    ....................caaagugcucauagugcagguagUuu................................................................   3      1   seq
    ......................aagugcucauagugcagguag...................................................................   2      0   seq
    .......................agugcucauagugcagguag...................................................................   1      0   seq
    .........................ugcucauagugcagguagU..................................................................   1      1   seq
    ........................................................cuacuguaaugugggcacuuacU...............................   2      1   seq
    .........................................................uacuguaaugugggcacu...................................   1      0   seq
    .........................................................uacuguaaugugggcacuu..................................   1      0   seq
    .........................................................uacuguaaugugggcacuua.................................   1      0   seq
    .........................................................uacuguaaugugggcacuuaA................................   1      1   seq
    .........................................................uacuguaaugugggcacuuac................................   6      0   seq
```

Mature Star

```
uaguuuguccuagcaguaucaaagugcucauagugcagguagcuuggaaucggaccuacuguaaugugggcacuuacaguacugcuagauaaagcgcauccuuagcuag
........................................................uacuguaaugugggcacuuaca...............................	2	0	seq
.........................................................acuguaaugugggcacuu..................................	1	0	seq
.........................................................acuguaaugugggcacuuac................................	1	0	seq
.........................................................acuguaaugugggcacuuacU...............................	1	1	seq
.........................................................acuguaaugugggcacuuaca...............................	1	0	seq
.........................................................acuguaaugugggcacuuacagu.............................	1	0	seq
```

Provisional ID	:	mun-miR-456
Score total	:	550.3
Score for star read(s)	:	3.9
Score for read counts	:	540
Score for mfe	:	1.8
Score for randfold	:	1.6
Score for cons. seed	:	3
Total read count	:	1071
Mature read count	:	1070
Loop read count	:	0
Star read count	:	1

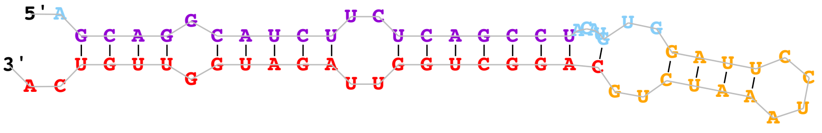

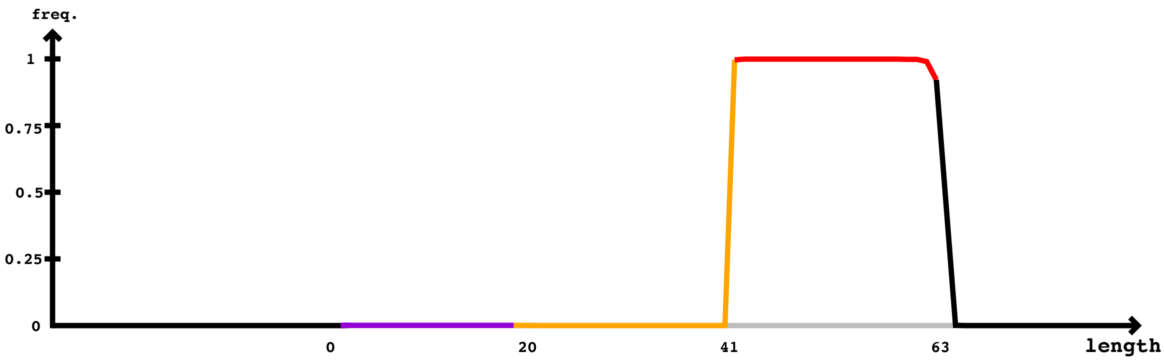

Star **Mature**

```
5'- uaaacugcugugugugcgugugugugugagcaggcaucuucucagccuacauguggauuccuaaaucugcaggcugguuagaugguugucaugcauucauuucauaucugc   -3'    obs
    uaaacugcugugugugcgugugugugugagcaggcaucuucucagccuacauguggauuccuaaaucugcaggcugguuagaugguugucaugcauucauuucauaucugc          exp
    ......((.(((((...(((.((((((((.(((.(((((..((((((((....(..((((....))))..))))))))..))))).))))))))))).))).)))))..))    reads     mm     sample
    .............................gcaggcaucuucucagccu...............................................................    1         0      seq
    .....................................................................caggcugguuagaugguu........................    1         0      seq
    .....................................................................caggcugguuagaugguugA......................    1         1      seq
    .....................................................................caggcugguuagaugguugu......................    8         0      seq
    .....................................................................caggcugguuagaugguuguU.....................    2         1      seq
    .....................................................................caggcugguuagaugguuguc.....................    70        0      seq
    .....................................................................caggcugguuagaugguugucU....................    629       1      seq
    .....................................................................caggcugguuagaugguuguca....................    53        0      seq
    .....................................................................caggcugguuagaugguugucC....................    218       1      seq
    .....................................................................caggcugguuagaugguugucG....................    1         1      seq
    .....................................................................caggcugguuagaugguugucCu...................    68        1      seq
    .....................................................................caggcugguuagaugguugucaA...................    2         1      seq
    .....................................................................caggcugguuagaugguugucUu...................    6         1      seq
    .....................................................................caggcugguuagaugguugucau...................    7         0      seq
    .....................................................................caggcugguuagaugguugucauU..................    1         1      seq
    ......................................................................aggcugguuagaugguuguc.....................    1         0      seq
    ......................................................................aggcugguuagaugguugucU....................    1         1      seq
    ......................................................................aggcugguuagaugguugucCu...................    1         1      seq
```

Provisional ID	:	mun-novel92
Score total	:	44
Score for star read(s)	:	3.9
Score for read counts	:	37.9
Score for mfe	:	1.2
Score for randfold	:	1.6
Score for cons. seed	:	-0.6
Total read count	:	86
Mature read count	:	75
Loop read count	:	0
Star read count	:	11

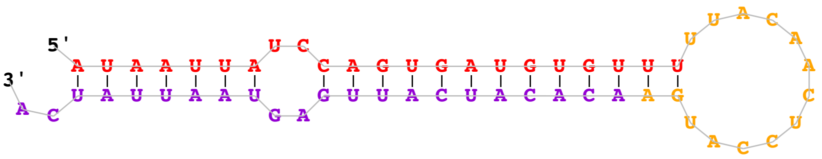

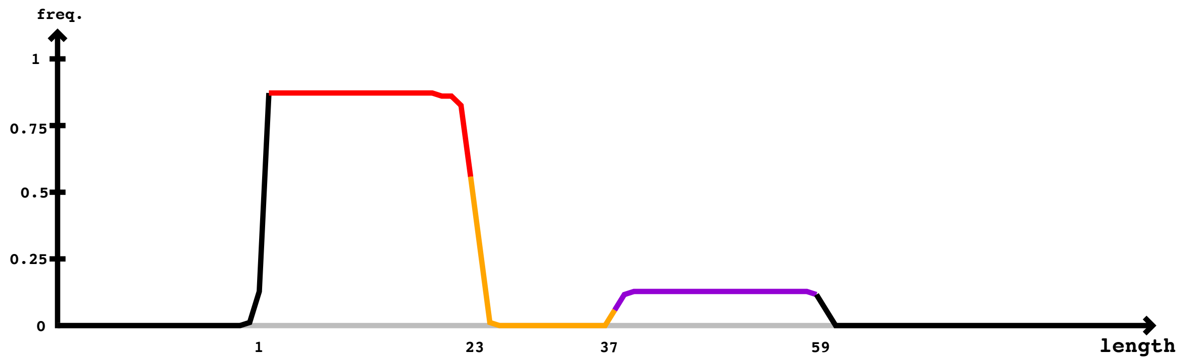

Mature **Star**

```
5'- cauauagguugaaacuauugauaauuauccagugauguguuuuuacaacuccaugaacacaucauugaguaauuaucaagagucucuacucuuucaccuuuacgcagucaaa  -3'   obs
    cauauagguugaaacuauugauaauuauccagugauguguuuuuacaacuccaugaacacaucauugaguaauuaucaagagucucuacucuuucaccuuuacgcagucaaa         exp
    ..((.((((.(((....((((((((((..((((((((((((............))))))))))))..))))))))))((((....)))).))))))).))............   reads   mm   sample
    ..................ugauaauuauccagugaugugu........................................................................   1       0    seq
    ...................gauaauuauccagugaugu..........................................................................   1       0    seq
    ...................gauaauuauccagugauguguu.......................................................................   8       0    seq
    ...................gauaauuauccagugauguguuC......................................................................   1       1    seq
    ....................auaauuauccagugaugugu........................................................................   2       0    seq
    ....................auaauuauccagugauguguu.......................................................................   14      0    seq
    ....................auaauuauUcagugauguguu.......................................................................   1       1    seq
    ....................auaauuauccagugauguguuu......................................................................   41      0    seq
    ....................auaauuauccagugauguguuA......................................................................   1       1    seq
    ....................auaaCuauccagugauguguuu......................................................................   1       1    seq
    ....................auaauuauccCgugauguguuu......................................................................   1       1    seq
    ....................auaauuauccagugauguguuuu.....................................................................   2       0    seq
    ....................auaauuauccagugauguguuuuu....................................................................   1       0    seq
    ........................................................acacaucauugaguaauuauc...................................   1       0    seq
    ........................................................acacaucauugaguaauuauca..................................   3       0    seq
    ........................................................acacaucauugaguaauuaucaU.................................   1       1    seq
    .........................................................cacaucauugaguaauuauca..................................   3       0    seq
    .........................................................cacaucauugaguaauuaucaa.................................   2       0    seq
    ..........................................................acaucauugaguaauuaucaa.................................   1       0    seq
```

Provisional ID	:	mun-novel51
Score total	:	16.3
Score for star read(s)	:	3.9
Score for read counts	:	8.8
Score for mfe	:	2.6
Score for randfold	:	1.6
Score for cons. seed	:	-0.6
Total read count	:	29
Mature read count	:	23
Loop read count	:	0
Star read count	:	6

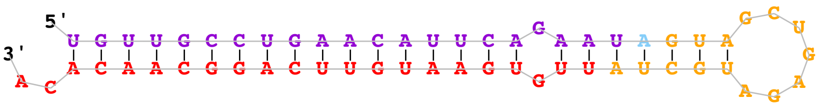

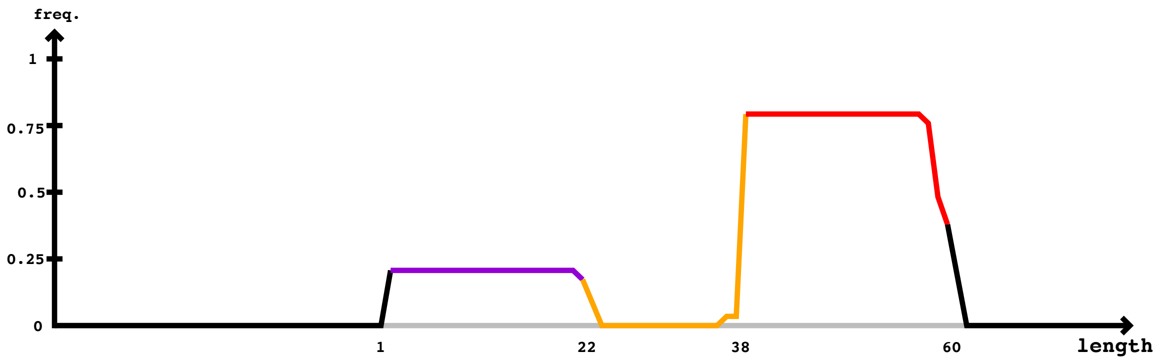

Star   Mature

```
5'- agaaaacugcaggacugagacuuccugaaguuguguugccugaacauucagaauaguagcugagaugcuauugugaauguucaggcaacacaacuucaggaaguaauuca  -3'   obs
    agaaaacugcaggacugagacuuccugaaguuguguugccugaacauucagaauaguagcugagaugcuauugugaauguucaggcaacacaacuucaggaaguaauuca         exp
    ................((.(((((((((((((((((((((((((((((((.(((((((.......))))))).)))))))))))))))))))))))))))))))...)).  reads   mm   sample
    .................................uguugccugaacauucagaa.........................................................  1       0    seq
    .................................uguugccugaacauucagaau........................................................  3       0    seq
    .................................uguugccugaacauucagaaA........................................................  2       1    seq
    ....................................................................uauugugaauguucaggcaacC....................  1       1    seq
    ......................................................................uugugaauguucaggcaac.....................  1       0    seq
    ......................................................................uugugaGuguucaggcaaca....................  1       1    seq
    ......................................................................uugugaauguucaggcaaca....................  6       0    seq
    ......................................................................uugugaauguucaggcaacac...................  3       0    seq
    ......................................................................uugugaauguucaggcaacaca..................  4       0    seq
    ......................................................................uugugaauguucaggcaacacU..................  7       1    seq
```

Provisional ID	:	mun-novel52
Score total	:	0.5
Score for star read(s)	:	-1.3
Score for read counts	:	-4.4
Score for mfe	:	1.7
Score for randfold	:	1.6
Score for cons. seed	:	3
Total read count	:	3
Mature read count	:	3
Loop read count	:	0
Star read count	:	0

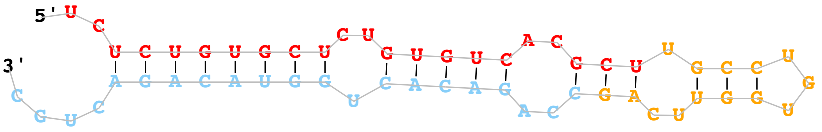

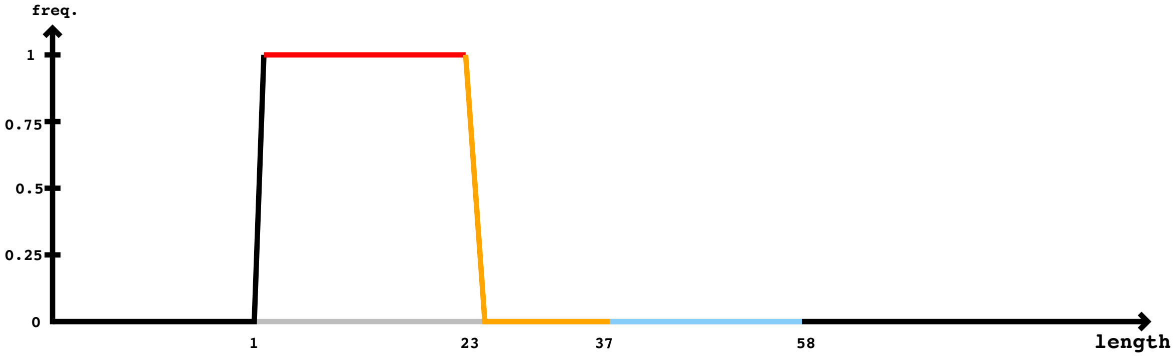

Mature　Star

```
5'- cccugauuuccaccacccuuucucugugcucugugucacgcuugccugugguucagccagacacugguacagacugcugggcugcuggagcauucgccagugugagaaucuu  -3'   exp
    ....((((..(((..(((....((((((((..(((((..(((.(((...)))..)))..))))).)))))))).....)))..(((((........))))))))..))))..   reads   mm   sample
    ....................ucucugugcucugugucacgcu......................................................................   3       0    seq
```

Provisional ID	:	mun-miR-19b-2
Score total	:	3376.4
Score for star read(s)	:	3.9
Score for read counts	:	3366
Score for mfe	:	1.9
Score for randfold	:	1.6
Score for cons. seed	:	3
Total read count	:	6614
Mature read count	:	6613
Loop read count	:	0
Star read count	:	1

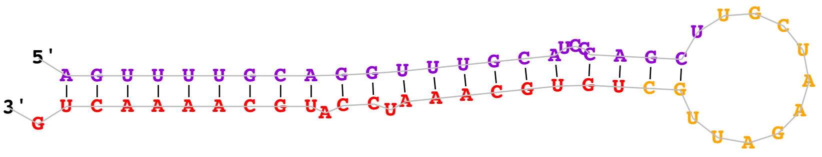

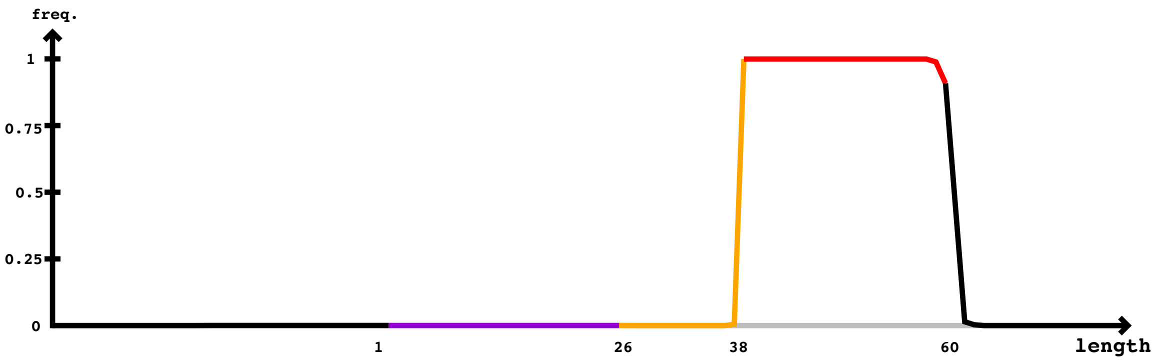

Star Mature

```
5'- caggcucuggagaagauggugcugauuacagucaguuuugcagguuugcaucccagcuugcuaagauugcugugcaaauccaugcaaaacugacuguggugguggugaau  -3'  obs
    caggcucuggagaagauggugcugauuacagucaguuuugcagguuugcaucccagcuugcuaagauugcugugcaaauccaugcaaaacugacuguggugguggugaau       exp
    ..(((.(((.......))).))).((((((((((((((((((((((((((...((((...........))))))))).)).)))))))))))))))))))..........  reads  mm  sample
    ..............gauggugcugauuacaguc.............................................................................  1      0   seq
    ...............auggugcugauuacaguc.............................................................................  1      0   seq
    .................................aguuuugcagguuugcaucccagcu....................................................  1      0   seq
    .....................................................................cugugcaaauccaugcaaaacu...................  14     0   seq
    .....................................................................cugugcaaauccaugcaaaacug..................  10     0   seq
    .....................................................................cugugcaaauccaugcaaaacugU.................  5      1   seq
    .....................................................................cugugcaaauccaugcaaaacuga.................  2      0   seq
    .....................................................................cugugcaaauccaugcaaaacugaU................  1      1   seq
    ......................................................................ugugcaaauccaugcaaaa.....................  1      0   seq
    ......................................................................uguAcaaauccaugcaaaac....................  1      1   seq
    ......................................................................ugugcaaauccaugcaaaac....................  70     0   seq
    ......................................................................ugCgcaaauccaugcaaaac....................  1      1   seq
    ......................................................................ugugcaaauccauUcaaaacu...................  1      1   seq
    ......................................................................uCugcaaauccaugcaaaacu...................  1      1   seq
    ......................................................................ugugcaaauccauCcaaaacu...................  1      1   seq
    ......................................................................Ggugcaaauccaugcaaaacu...................  3      1   seq
    ......................................................................Agugcaaauccaugcaaaacu...................  1      1   seq
    ......................................................................ugugcaaauccaugUaaaacu...................  31     1   seq
    ......................................................................ugCgcaaauccaugcaaaacu...................  1      1   seq
    ......................................................................ugugcaaauccaugcaaaacA...................  1      1   seq
    ......................................................................ugugcaaauccaugcaaaacu...................  476    0   seq
    ......................................................................ugugcaaauccaugcaaaacGg..................  7      1   seq
    ......................................................................ugAgcaaauccaugcaaaacug..................  1      1   seq
    ......................................................................uguAcaaauccaugcaaaacug..................  2      1   seq
    ......................................................................ugugcaaauccaugcaaaacug..................  3382   0   seq
    ......................................................................ugugcaaauccaugAaaaacug..................  1      1   seq
    ......................................................................ugugcaaauccaugcaaaGcug..................  1      1   seq
    ......................................................................ugugcaaaCccaugcaaaacug..................  2      1   seq
    ......................................................................ugugcaaauccaugcaaaacuA..................  3      1   seq
    ......................................................................Agugcaaauccaugcaaaacug..................  2      1   seq
    ......................................................................ugugcaGauccaugcaaaacug..................  1      1   seq
    ......................................................................ugugcUaauccaugcaaaacug..................  1      1   seq
    ......................................................................ugugAaaauccaugcaaaacug..................  1      1   seq
```

```
                                 Star                                  Mature
caggcucuggagaagauggugcugauuacagucaguuuugcagguuugcaucccagcuugcuaagauugcugugcaaauccaugcaaaacugacuguggugguggugaau
......................................................................ugugcaaauccaugcaaaacCg..................  1     1  seq
......................................................................ugugcaaauccaugcaaaaAug..................  1     1  seq
......................................................................ugugcaaauccaugcaaaacuU..................  2     1  seq
......................................................................ugugcaaauccaCgcaaaacug..................  1     1  seq
......................................................................ugugcaaauccaugUaaaacug..................  20    1  seq
......................................................................ugugcaaaucAaugcaaaacug..................  1     1  seq
......................................................................uguUcaaauccaugcaaaacug..................  1     1  seq
......................................................................uAugcaaauccaugcaaaacug..................  1     1  seq
......................................................................Ggugcaaauccaugcaaaacug..................  3     1  seq
......................................................................ugugcaaauccauCcaaaacug..................  2     1  seq
......................................................................uCugcaaauccaugcaaaacug..................  1     1  seq
......................................................................ugugcaaauccaugcaaaacuUa.................  1     1  seq
......................................................................ugugcaaauccaugcaaaacugC.................  27    1  seq
......................................................................ugugcaaauccaugUaaaacuga.................  1     1  seq
......................................................................ugugcaaGuccaugcaaaacuga.................  1     1  seq
......................................................................Ggugcaaauccaugcaaaacuga.................  5     1  seq
......................................................................ugugcaaauccaCgcaaaacuga.................  1     1  seq
......................................................................ugugcaaauccauUcaaaacuga.................  1     1  seq
......................................................................ugugcaaauccaugAaaaacuga.................  1     1  seq
......................................................................ugugcaaauccaugcaaaacugU.................  784   1  seq
......................................................................ugugcGaauccaugcaaaacuga.................  1     1  seq
......................................................................uUugcaaauccaugcaaaacuga.................  2     1  seq
......................................................................Agugcaaauccaugcaaaacuga.................  1     1  seq
......................................................................ugugcaaauccauCcaaaacuga.................  2     1  seq
......................................................................ugugcaaauccaugcaaaacugG.................  14    1  seq
......................................................................ugugcaaauccaugcaaaacuga.................  1617  0  seq
......................................................................ugugcaaaCccaugcaaaacuga.................  1     1  seq
......................................................................ugugcaaaucUaugcaaaacugac................  7     1  seq
......................................................................ugugcaaauccaugcaaaacugac................  19    0  seq
......................................................................ugugcaaauccaugcaaaacugaA................  7     1  seq
......................................................................ugugcaaauccaugcaaaacugaU................  40    1  seq
......................................................................ugugcaaauccaugcaaaacugaUu...............  2     1  seq
......................................................................ugugcaaauccaugcaaaacugaAu...............  1     1  seq
......................................................................ugugcaaauccaugcaaaacugacu...............  8     0  seq
......................................................................ugugcaaaucUaugcaaaacugacu...............  9     1  seq
........................................................................ugUaaauccaugcaaaacug..................  1     1  seq
```

Provisional ID : mun-miR-10a-1
Score total : 246276.2
Score for star read(s) : 3.9
Score for read counts : 246266.1
Score for mfe : 1.7
Score for randfold : 1.6
Score for cons. seed : 3
Total read count : 483052
Mature read count : 482961
Loop read count : 0
Star read count : 91

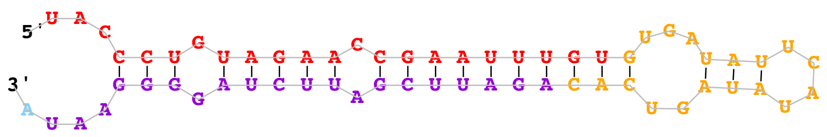

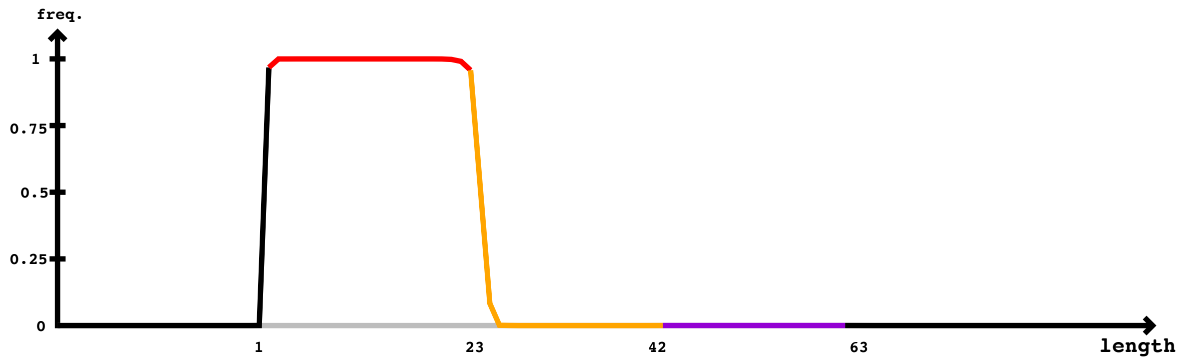

Mature Star

```
5'- guuauuacguugucuauauauacccuguagaaccgaauuugugugauauucauauagucacagauucgauucuaggggaauauauggucgaugcaaaaacuucaccguucug   -3'    obs
    guuauuacguugucuauauauacccuguagaaccgaauuugugugauauucauauagucacagauucgauucuaggggaauauauggucgaugcaaaaacuucaccguucug          exp
    (((.((.(((((.((((((((.((((.(((((.((((((((((...(((....)))..)))))))))).))))))))).)))))))).))))).)).)))............   reads      mm        sample
    .................auauacccuguagaaccgaauuugu......................................................................   1          0         seq
    ..................uauacccuguagaaccgaauuu........................................................................   1          0         seq
    ..................uCuacccuguagaaccgaauuugu......................................................................   2          1         seq
    ..................uauacccuguagaaccgaauuugu......................................................................   2          0         seq
    ...................auacccuguagaaccgaauuu........................................................................   5          0         seq
    ...................Cuacccuguagaaccgaauuug.......................................................................   1          1         seq
    ...................auacccuguagaaccgaauuug.......................................................................   4          0         seq
    ...................auacccuguagaaccgaauuugu......................................................................   64         0         seq
    ...................auacccuguagaaccgaauuugA......................................................................   2          1         seq
    ...................Cuacccuguagaaccgaauuugu......................................................................   14         1         seq
    ...................auacccuguagaaccgaauuugug.....................................................................   2          0         seq
    ...................Cuacccuguagaaccgaauuugugu....................................................................   1          1         seq
    ...................auacccuguagaaccgaauuugugu....................................................................   1          0         seq
    ....................uacccuguagUaccgaau..........................................................................   1          1         seq
    ....................uacccuguagCaccgaau..........................................................................   1          1         seq
    ....................uacccuguagaaccgaaA..........................................................................   5          1         seq
    ....................uacccuguagaaccgaUu..........................................................................   1          1         seq
    ....................uacccuguagaaccgaau..........................................................................   78         0         seq
    ....................uacccuguagaGccgaau..........................................................................   1          1         seq
    ....................uacccuguagaaccgaauA.........................................................................   2          1         seq
    ....................uacccuguagaaccgaauu.........................................................................   749        0         seq
    ....................uacccugCagaaccgaauu.........................................................................   1          1         seq
    ....................uacccuguagaaAcgaauu.........................................................................   1          1         seq
    ....................uacccuguagaaUcgaauu.........................................................................   1          1         seq
    ....................uacccuguagaaccUaauu.........................................................................   1          1         seq
    ....................Gacccuguagaaccgaauu.........................................................................   2          1         seq
    ....................uCcccuguagaaccgaauu.........................................................................   1          1         seq
    ....................uaccAuguagaaccgaauu.........................................................................   1          1         seq
    ....................uacccuguUgaaccgaauu.........................................................................   1          1         seq
    ....................uacccuguagaaccgCauu.........................................................................   1          1         seq
    ....................uacccuguagaacUgaauuu........................................................................   1          1         seq
    ....................uacccuguagaaccgaauuG........................................................................   1          1         seq
    ....................uacccuguagaaccAaauuu........................................................................   2          1         seq
```

Mature Star

```
guuauuacguugucuauauauacccuguagaaccgaauuugugugauauucauauagucacagauucgauucuaggggaauauauggucgaugcaaaaacuucaccguucug
....................uacccuguagaCccgaauuu........................................................................  1      1   seq
....................Aacccuguagaaccgaauuu........................................................................  3      1   seq
....................uacccuguagaaAcgaauuu........................................................................  1      1   seq
....................uacccAguagaaccgaauuu........................................................................  1      1   seq
....................uacccuguagaaccgaaGuu........................................................................  2      1   seq
....................uacccuguagaaccgaauAu........................................................................  1      1   seq
....................uacccuguagaaccgGauuu........................................................................  2      1   seq
....................uacccuguagaaccgaauuu........................................................................  3420   0   seq
....................uacccuguagaaccgaauCu........................................................................  1      1   seq
....................uacccuguagaGccgaauuu........................................................................  1      1   seq
....................uacccugAagaaccgaauuu........................................................................  2      1   seq
....................uacccuguaUaaccgaauuu........................................................................  1      1   seq
....................uacccuguagaaUcgaauuu........................................................................  2      1   seq
....................Cacccuguagaaccgaauuu........................................................................  2      1   seq
....................uacccuguagaaccgaauuA........................................................................  25     1   seq
....................uacccuAuagaaccgaauuug.......................................................................  2      1   seq
....................uacccuguagaaccgUauuug.......................................................................  5      1   seq
....................uaccAuguagaaccgaauuug.......................................................................  6      1   seq
....................uaccUuguagaaccgaauuug.......................................................................  2      1   seq
....................uacccuguagaaccgGauuug.......................................................................  5      1   seq
....................uacccuguagaGccgaauuug.......................................................................  2      1   seq
....................uacAcuguagaaccgaauuug.......................................................................  1      1   seq
....................uaUccuguagaaccgaauuug.......................................................................  1      1   seq
....................uacccuguagaaccgaaAuug.......................................................................  3      1   seq
....................uacccuguagaaccgaauCug.......................................................................  1      1   seq
....................uacccugCagaaccgaauuug.......................................................................  6      1   seq
....................uGcccuguagaaccgaauuug.......................................................................  2      1   seq
....................uacccuguagaaccgaauuuU.......................................................................  45     1   seq
....................uacccugAagaaccgaauuug.......................................................................  3      1   seq
....................uacccuguagaaccAaauuug.......................................................................  1      1   seq
....................uacccuguagaaccCaauuug.......................................................................  2      1   seq
....................uacccuguaUaaccgaauuug.......................................................................  1      1   seq
....................uaAccuguagaaccgaauuug.......................................................................  2      1   seq
....................uacccuguagaaAcgaauuug.......................................................................  1      1   seq
....................uacccuguagaaccgaGuuug.......................................................................  3      1   seq
....................uacccuguagaaccUaauuug.......................................................................  3      1   seq
....................uacccuguagaacAgaauuug.......................................................................  6      1   seq
....................uacccuguagaaccgaaCuug.......................................................................  1      1   seq
....................uacccuguaCaaccgaauuug.......................................................................  2      1   seq
....................uacccuguagaaccgCauuug.......................................................................  3      1   seq
....................uacccAguagaaccgaauuug.......................................................................  1      1   seq
....................uacccuguagaaccgaCuuug.......................................................................  1      1   seq
....................uacccuguagaaUcgaauuug.......................................................................  6      1   seq
....................Gacccuguagaaccgaauuug.......................................................................  17     1   seq
....................uacccugGagaaccgaauuug.......................................................................  6      1   seq
....................uacccuguagaCccgaauuug.......................................................................  1      1   seq
....................uacccuguagaaccgaauuug.......................................................................  15554  0   seq
....................uacccuguagaaccgaauuuA.......................................................................  32     1   seq
....................Cacccuguagaaccgaauuug.......................................................................  7      1   seq
....................uacccuguagaacUgaauuug.......................................................................  7      1   seq
....................Aacccuguagaaccgaauuug.......................................................................  11     1   seq
....................uacccuUuagaaccgaauuug.......................................................................  2      1   seq
....................uacccuguagaaccgaUuuug.......................................................................  2      1   seq
....................uacccuguagUaccgaauuug.......................................................................  1      1   seq
....................uacccugGagaaccgaauuugu......................................................................  26     1   seq
....................uacccuguagaaccgUauuugu......................................................................  22     1   seq
....................uacccuguagaaccgGauuugu......................................................................  69     1   seq
....................uacccuguagaaccgaaAuugu......................................................................  52     1   seq
....................uacccuCuagaaccgaauuugu......................................................................  10     1   seq
....................uacccuguagaaccgaGuuugu......................................................................  34     1   seq
....................uacccuguagaaccgCauuugu......................................................................  27     1   seq
....................uUcccuguagaaccgaauuugu......................................................................  9      1   seq
....................uacccuAuagaaccgaauuugu......................................................................  33     1   seq
....................uacccuguagaaccgaauuuAu......................................................................  27     1   seq
....................uacccuguUgaaccgaauuugu......................................................................  11     1   seq
....................uacccuguagaaccgaauuugC......................................................................  51     1   seq
....................uacGcuguagaaccgaauuugu......................................................................  17     1   seq
....................uaAccuguagaaccgaauuugu......................................................................  47     1   seq
....................uacccuguagaaGcgaauuugu......................................................................  5      1   seq
....................uacccuUuagaaccgaauuugu......................................................................  70     1   seq
```

```
                    Mature                                  Star
guuauuacguugucuauauauacccuguagaaccgaauuugugugauauucauauagucacagauucgauucuaggggaauauauggucgaugcaaaaacuucaccguucug
....................uacccuguagaaccgaauuugu......................................................................   367714   0   seq
....................uacccuguagaaUcgaauuugu......................................................................   120      1   seq
....................uacccugCagaaccgaauuugu......................................................................   83       1   seq
....................Cacccuguagaaccgaauuugu......................................................................   105      1   seq
....................uacUcuguagaaccgaauuugu......................................................................   22       1   seq
....................Gacccuguagaaccgaauuugu......................................................................   290      1   seq
....................uacccuguagGaccgaauuugu......................................................................   24       1   seq
....................uacccuguaCaaccgaauuugu......................................................................   14       1   seq
....................uacccuguCgaaccgaauuugu......................................................................   2        1   seq
....................uaccGuguagaaccgaauuugu......................................................................   9        1   seq
....................Aacccuguagaaccgaauuugu......................................................................   215      1   seq
....................uacccuguagaaccgaaCuugu......................................................................   20       1   seq
....................uacccuguagaGccgaauuugu......................................................................   33       1   seq
....................uCcccuguagaaccgaauuugu......................................................................   3        1   seq
....................uacccuguagaaccgaaGuugu......................................................................   14       1   seq
....................uacccuguagaaccgaauuuCu......................................................................   6        1   seq
....................uacccugAagaaccgaauuugu......................................................................   47       1   seq
....................uaUccuguagaaccgaauuugu......................................................................   15       1   seq
....................uacccuguagaacAgaauuugu......................................................................   81       1   seq
....................uacccuguagaCccgaauuugu......................................................................   59       1   seq
....................uacccuguagaaccgaauuAgu......................................................................   8        1   seq
....................uacccuguagaaAcgaauuugu......................................................................   44       1   seq
....................uacccAguagaaccgaauuugu......................................................................   14       1   seq
....................uacccuguagCaccgaauuugu......................................................................   7        1   seq
....................uaccAuguagaaccgaauuugu......................................................................   55       1   seq
....................uacccuguaAaaccgaauuugu......................................................................   29       1   seq
....................uacccuguagaaccgaauuuUu......................................................................   110      1   seq
....................uaGccuguagaaccgaauuugu......................................................................   8        1   seq
....................uacAcuguagaaccgaauuugu......................................................................   75       1   seq
....................uacccuguagaaccgaCuuugu......................................................................   10       1   seq
....................uacccuguagaaccgaauCugu......................................................................   17       1   seq
....................uacccuguaUaaccgaauuugu......................................................................   52       1   seq
....................uacccCguagaaccgaauuugu......................................................................   48       1   seq
....................uacccuguagaaccgaauGugu......................................................................   2        1   seq
....................uacccuguagaaccUaauuugu......................................................................   59       1   seq
....................uGcccuguagaaccgaauuugu......................................................................   75       1   seq
....................uacccuguGgaaccgaauuugu......................................................................   30       1   seq
....................uacccuguagaaccAaauuugu......................................................................   52       1   seq
....................uacccuguagaaccgaauuGgu......................................................................   3        1   seq
....................uacccuguagaaccCaauuugu......................................................................   28       1   seq
....................uacccuguagUaccgaauuugu......................................................................   12       1   seq
....................uacccuguagaacUgaauuugu......................................................................   119      1   seq
....................uacccuguagaacGgaauuugu......................................................................   9        1   seq
....................uacccuguagaaccgaauuCgu......................................................................   24       1   seq
....................uaccUuguagaaccgaauuugu......................................................................   32       1   seq
....................uacccuguagaaccgaUuuugu......................................................................   29       1   seq
....................uacccuguagaaccgaauAugu......................................................................   7        1   seq
....................uacccuguagaaccgaauuugA......................................................................   2424     1   seq
....................uacccGguagaaccgaauuugu......................................................................   7        1   seq
....................uacccuguagaaccgaauuugG......................................................................   235      1   seq
....................uacccuguagaaccgaauuuCug.....................................................................   3        1   seq
....................uGcccuguagaaccgaauuugug.....................................................................   3        1   seq
....................uacccuguagaaccgGauuugug.....................................................................   7        1   seq
....................uacccuguagaaUcgaauuugug.....................................................................   6        1   seq
....................uaccUuguagaaccgaauuugug.....................................................................   1        1   seq
....................uacccuguaAaaccgaauuugug.....................................................................   1        1   seq
....................uacccuguagaaccgaauCugug.....................................................................   2        1   seq
....................uacAcuguagaaccgaauuugug.....................................................................   4        1   seq
....................uacccAguagaaccgaauuugug.....................................................................   1        1   seq
....................uacccuguagaaccgaauuuAug.....................................................................   1        1   seq
....................uacccuguGgaaccgaauuugug.....................................................................   4        1   seq
....................uacccuguagaaccAaauuugug.....................................................................   7        1   seq
....................uacccuguagaaccgaauuCgug.....................................................................   2        1   seq
....................uacccuguagaaAcgaauuugug.....................................................................   5        1   seq
....................uacccuguaUaaccgaauuugug.....................................................................   5        1   seq
....................uacccuguagaacAgaauuugug.....................................................................   12       1   seq
....................uacccuguagaaccgaauuugAg.....................................................................   4        1   seq
....................uacccuguagaaccgaaGuugug.....................................................................   3        1   seq
....................uacccuguagaaccgaauuugGg.....................................................................   33       1   seq
....................uaccAuguagaaccgaauuugug.....................................................................   3        1   seq
```

```
                    Mature                                   Star
guuauuacguugucuauauauacccuguagaaccgaauuugugugauauucauauagucacagauucgauucuaggggaauauauggucgaugcaaaaacuucaccguucug
....................uacccuguagaaccgaauuuUug.....................................................................	8	1	seq
....................uacccuguagaaccgaauuugCg.....................................................................	5	1	seq
....................uacccuguagaaccCaauuugug.....................................................................	2	1	seq
....................uacccuguagaGccgaauuugug.....................................................................	3	1	seq
....................uacccugCagaaccgaauuugug.....................................................................	10	1	seq
....................uacccuguagaaccgaGuuugug.....................................................................	5	1	seq
....................uacccuguagaacGgaauuugug.....................................................................	2	1	seq
....................uacccuguagaaccgaUuuugug.....................................................................	3	1	seq
....................uacccuguagaaccgaauGugug.....................................................................	1	1	seq
....................uacccuguagaaccUaauuugug.....................................................................	4	1	seq
....................uacccuguagaacUgaauuugug.....................................................................	5	1	seq
....................uaccGuguagaaccgaauuugug.....................................................................	1	1	seq
....................uacUcuguagaaccgaauuugug.....................................................................	4	1	seq
....................Gacccuguagaaccgaauuugug.....................................................................	31	1	seq
....................uacccGguagaaccgaauuugug.....................................................................	1	1	seq
....................uacccuAuagaaccgaauuugug.....................................................................	2	1	seq
....................uacccuguagaaccgaauuuguC.....................................................................	24	1	seq
....................uacccuguagaaccgaaCuugug.....................................................................	9	1	seq
....................uacGcuguagaaccgaauuugug.....................................................................	3	1	seq
....................uacccuUuagaaccgaauuugug.....................................................................	4	1	seq
....................uacccuguagaaccgaauuGgug.....................................................................	2	1	seq
....................uacccuguagaaccgaaAuugug.....................................................................	9	1	seq
....................Cacccuguagaaccgaauuugug.....................................................................	13	1	seq
....................uacccuguagaaccgaauuugug.....................................................................	36835	0	seq
....................uacccuguagaaccgCauuugug.....................................................................	1	1	seq
....................uaUccuguagaaccgaauuugug.....................................................................	1	1	seq
....................uacccuguagaaccgaauuAgug.....................................................................	1	1	seq
....................uacccuguagaaccgaauuuguA.....................................................................	2128	1	seq
....................uacccuguagaCccgaauuugug.....................................................................	10	1	seq
....................Aacccuguagaaccgaauuugug.....................................................................	22	1	seq
....................uacccugGagaaccgaauuugug.....................................................................	4	1	seq
....................uacccCguagaaccgaauuugug.....................................................................	6	1	seq
....................uaAccuguagaaccgaauuugug.....................................................................	1	1	seq
....................uacccuguagGaccgaauuugug.....................................................................	2	1	seq
....................uacccuguagCaccgaauuugug.....................................................................	3	1	seq
....................uacccuguaCaaccgaauuugug.....................................................................	1	1	seq
....................uacccuguagaaccgaauuuguU.....................................................................	2847	1	seq
....................uacccugAagaaccgaauuugug.....................................................................	5	1	seq
....................uacccuguagaaccgUauuugug.....................................................................	5	1	seq
....................uacccuguagaaGcgaauuugug.....................................................................	1	1	seq
....................uacccuguagaaccgaauuugCgu....................................................................	7	1	seq
....................uacccuguagaaccgaauAugugu....................................................................	2	1	seq
....................uacccuguagaaccgaauuugugA....................................................................	11242	1	seq
....................uaccAuguagaaccgaauuugugu....................................................................	2	1	seq
....................uacccuguagaaccgaauuugAgu....................................................................	3	1	seq
....................uacccuUuagaaccgaauuugugu....................................................................	2	1	seq
....................uacccuguagaaccgaauuuguCu....................................................................	5	1	seq
....................uacAcuguagaaccgaauuugugu....................................................................	3	1	seq
....................uacccuguagaaccgaUuuugugu....................................................................	2	1	seq
....................uacccuguagaaccgaauuAgugu....................................................................	2	1	seq
....................uacccuguagaaccgaauuuguAu....................................................................	140	1	seq
....................uacccuguagaaccAaauuugugu....................................................................	1	1	seq
....................uacccuCuagaaccgaauuugugu....................................................................	2	1	seq
....................uacccuguagaaccgaauuuAugu....................................................................	1	1	seq
....................uacccuguagaacUgaauuugugu....................................................................	4	1	seq
....................uaAccuguagaaccgaauuugugu....................................................................	1	1	seq
....................Cacccuguagaaccgaauuugugu....................................................................	8	1	seq
....................uacUcuguagaaccgaauuugugu....................................................................	2	1	seq
....................uacccuguagaaccgaauuCgugu....................................................................	2	1	seq
....................uacccuguagaaccgaauuuUugu....................................................................	6	1	seq
....................uacccuguagaaccCaauuugugu....................................................................	1	1	seq
....................uacccuguagaaAcgaauuugugu....................................................................	1	1	seq
....................uacccuguagaaccgaauuugugC....................................................................	68	1	seq
....................uacccuguagaacAgaauuugugu....................................................................	4	1	seq
....................uacccuguaUaaccgaauuugugu....................................................................	7	1	seq
....................uacccGguagaaccgaauuugugu....................................................................	1	1	seq
....................uacccuguagaaccgaauuugugG....................................................................	2554	1	seq
....................uacccuguagaCccgaauuugugu....................................................................	2	1	seq
....................uacccuguagaaccgaGuuugugu....................................................................	2	1	seq
....................uacccuguagCaccgaauuugugu....................................................................	2	1	seq
```

Mature   Star

```
guuauuacguugucuauauauacccuguagaaccgaauuugugugauauucauauagucacagauucgauucuaggggaauauauggucgaugcaaaacuucaccguucug
....................uacccuguagaaccgaaCuugugu...................................................................  1      1  seq
....................uacccuguagaaccgaauuuguUu...................................................................  149    1  seq
....................uacccuguagaaccgaaAuugugu...................................................................  5      1  seq
....................uacccuguGgaaccgaauuugugu...................................................................  3      1  seq
....................Gacccuguagaaccgaauuugugu...................................................................  12     1  seq
....................uacccuguagaaccgaaGuugugu...................................................................  1      1  seq
....................uacccugCagaaccgaauuugugu...................................................................  2      1  seq
....................uaccUuguagaaccgaauuugugu...................................................................  2      1  seq
....................uacccugAagaaccgaauuugugu...................................................................  1      1  seq
....................uacccuguUgaaccgaauuugugu...................................................................  1      1  seq
....................uaUccuguagaaccgaauuugugu...................................................................  3      1  seq
....................uacccuAuagaaccgaauuugugu...................................................................  1      1  seq
....................uacccuguagaaccUaauuugugu...................................................................  1      1  seq
....................uacccuguaCaaccgaauuugugu...................................................................  1      1  seq
....................uacccuguagaaccgaauuugGgu...................................................................  18     1  seq
....................uacccuguagGaccgaauuugugu...................................................................  2      1  seq
....................uacccuguagaaccgUauuugugu...................................................................  2      1  seq
....................uacccAguagaaccgaauuugugu...................................................................  1      1  seq
....................Aacccuguagaaccgaauuugugu...................................................................  9      1  seq
....................uacccCguagaaccgaauuugugu...................................................................  2      1  seq
....................uaccGuguagaaccgaauuugugu...................................................................  1      1  seq
....................uacccuguagaaccgaauuugugu...................................................................  17510  0  seq
....................uacGcuguagaaccgaauuugugu...................................................................  1      1  seq
....................uacccuguagaaccgaauuugugAg..................................................................  7      1  seq
....................uacccuguagaaccgaauuugugug..................................................................  64     0  seq
....................uacccuguagaUccgaauuugugug..................................................................  2      1  seq
....................uacccuguagaaccgaauuuguguA..................................................................  39     1  seq
....................uacccuguagaaccgaauuuguguU..................................................................  135    1  seq
....................uacccuguagaaccgaauuuguguC..................................................................  1      1  seq
....................uacccuguagaaccgaauuuguguAa.................................................................  5      1  seq
....................uacccuguagaaccgaauuuguguUa.................................................................  1      1  seq
....................uacccuguagaaccgaauuuguguga.................................................................  64     0  seq
....................uacccuguagaaccgaauuugugugU.................................................................  28     1  seq
....................uacccuguagaaccgaauuugugugG.................................................................  5      1  seq
....................uacccuguagaaccgaauuugugugau................................................................  6      0  seq
....................uacccuguagaaccgaauuuguguAau................................................................  1      1  seq
....................uacccuguagaaccgaauuugugugaua...............................................................  5      0  seq
....................uacccuguagaaccgaauuugugugauU...............................................................  1      1  seq
....................uacccuguagaaccgaauuugugugauauu.............................................................  1      0  seq
....................uacccuguagaaccgaauuugugugauauucauU.........................................................  1      1  seq
.....................acccuguagaaUcgaauu........................................................................  1      1  seq
.....................acccuguagaaccgaauu........................................................................  25     0  seq
.....................acccuguagaaccgaauuu.......................................................................  95     0  seq
.....................acccuguagaaccgUauuug......................................................................  1      1  seq
.....................acccuguagaaccgaauuug......................................................................  253    0  seq
.....................acccuguagaaccgaauuuA......................................................................  2      1  seq
.....................acccuguagaaccCaauuug......................................................................  1      1  seq
.....................acccCguagaaccgaauuug......................................................................  2      1  seq
.....................acccugCagaaccgaauuugu.....................................................................  1      1  seq
.....................acccuguagaacAgaauuugu.....................................................................  3      1  seq
.....................acccuguagaaccgaauuuUu.....................................................................  2      1  seq
.....................acccuguagaacUgaauuugu.....................................................................  1      1  seq
.....................acccuguagaaccgaaCuugu.....................................................................  2      1  seq
.....................acccuguagaaUcgaauuugu.....................................................................  4      1  seq
.....................acccuguagaaccgaauuugC.....................................................................  1      1  seq
.....................acccuguagaaccgaauuugu.....................................................................  4085   0  seq
.....................acccuguagaaAcgaauuugu.....................................................................  1      1  seq
.....................acccuguagaaccgaauuugA.....................................................................  52     1  seq
.....................Ccccuguagaaccgaauuugu.....................................................................  1      1  seq
.....................acccuguagaCccgaauuugu.....................................................................  6      1  seq
.....................acccuguagaaccgaauuugG.....................................................................  3      1  seq
.....................aAccuguagaaccgaauuugu.....................................................................  1      1  seq
.....................acUcuguagaaccgaauuugu.....................................................................  1      1  seq
.....................acccuguagaaccCaauuugu.....................................................................  2      1  seq
.....................acccuguagaaccAaauuugu.....................................................................  1      1  seq
.....................acccuguagaaccgaauuCgu.....................................................................  1      1  seq
.....................Gcccuguagaaccgaauuugu.....................................................................  3      1  seq
.....................acccuguagaaccgaauuuAu.....................................................................  1      1  seq
.....................accAuguagaaccgaauuugu.....................................................................  1      1  seq
.....................acccuguaUaaccgaauuugu.....................................................................  1      1  seq
```

```
                    Mature                                  Star
guauuacguugucuauauauacccuguagaaccgaauuugugugauauucauauagucacagauucgauucuaggggaauauauggucgaugcaaaaacuucaccguucug
....................acccuguagaGccgaauuugu......................................................................	2	1	seq
....................acccuguagaaccgaUuugu.......................................................................	1	1	seq
....................acccuguagaaccCaauuugug.....................................................................	1	1	seq
....................acccuguagaCccgaauuugug.....................................................................	6	1	seq
....................acccuguagaaccUaauuugug.....................................................................	1	1	seq
....................acccuguagaacAgaauuugug.....................................................................	2	1	seq
....................accAuguagaaccgaauuugug.....................................................................	1	1	seq
....................acccuguagaaUcgaauuugug.....................................................................	2	1	seq
....................acccuguagaaccgaauuugCg.....................................................................	1	1	seq
....................acccuguUgaaccgaauuugug.....................................................................	1	1	seq
....................acccuguagaaccgaauuCgug.....................................................................	1	1	seq
....................acccuguagaaccgaauuuguA.....................................................................	33	1	seq
....................acccuguagGaccgaauuugug.....................................................................	1	1	seq
....................Ccccuguagaaccgaauuugug.....................................................................	1	1	seq
....................acccuguagaaccgaauuuguU.....................................................................	26	1	seq
....................acccuguagaaccgaauuugug.....................................................................	2816	0	seq
....................acccuguagaGccgaauuugug.....................................................................	1	1	seq
....................acccuguagaaccgaauuuUug.....................................................................	2	1	seq
....................aAccuguagaaccgaauuugug.....................................................................	1	1	seq
....................acccCguagaaccgaauuugug.....................................................................	2	1	seq
....................acccuguagaaccgGauuugug.....................................................................	1	1	seq
....................acccuguagaaccgaauuugGg.....................................................................	3	1	seq
....................Gcccuguagaaccgaauuugug.....................................................................	1	1	seq
....................acccuguagaaAcgaauuugug.....................................................................	1	1	seq
....................acccuguagaacUgaauuugug.....................................................................	1	1	seq
....................acccuguagaaccgaaAuugug.....................................................................	1	1	seq
....................acccCguagaaccgaauuugugu....................................................................	1	1	seq
....................acccuguagaaccgaaAuugugu....................................................................	1	1	seq
....................acAcuguagaaccgaauuugugu....................................................................	2	1	seq
....................acccuguagaaccgaauuugCgu....................................................................	3	1	seq
....................acccuguagaaccgaauuuguUu....................................................................	3	1	seq
....................Gcccuguagaaccgaauuugugu....................................................................	4	1	seq
....................acccuguagaaccgaauuugGgu....................................................................	3	1	seq
....................acccuguagaaccgaauuugAgu....................................................................	1	1	seq
....................acccuguagaaccgCauuugugu....................................................................	2	1	seq
....................acccuguagaaccgaauuuUugu....................................................................	2	1	seq
....................acccuguagaaccgaauuugugA....................................................................	2099	1	seq
....................acccuguagaCccgaauuugugu....................................................................	3	1	seq
....................acccuguagaaccgaauuuguAu....................................................................	1	1	seq
....................acccuguagaaccgaGuuugugu....................................................................	1	1	seq
....................acccuguagaGccgaauuugugu....................................................................	1	1	seq
....................acccuguagaaUcgaauuugugu....................................................................	5	1	seq
....................acccuguagaaccgaauuugugG....................................................................	573	1	seq
....................acccuguagaaccgaauCugugu....................................................................	2	1	seq
....................acccuguagaaccgaauuCgugu....................................................................	1	1	seq
....................acccuguaUaaccgaauuugugu....................................................................	2	1	seq
....................Ucccuguagaaccgaauuugugu....................................................................	1	1	seq
....................acccuguagaaccgaauuugugC....................................................................	7	1	seq
....................acccuguagaaccgaauuugugu....................................................................	5078	0	seq
....................acccuguagaaccgaauuuguguU...................................................................	44	1	seq
....................acccuguagaUccgaauuugugug...................................................................	3	1	seq
....................acccuguagaaccgaauuuguguA...................................................................	13	1	seq
....................acccuguagaaccgaauuugugug...................................................................	36	0	seq
....................acccuguagaaccgaauuuguguAa..................................................................	2	1	seq
....................acccuguagaaccgaauuuguguga..................................................................	35	0	seq
....................acccuguagaaccgaauuugugugU..................................................................	10	1	seq
....................acccuguagaaccgaauuugugugG..................................................................	7	1	seq
.....................cccuguagaaccgaauuug.......................................................................	1	0	seq
.....................Uccuguagaaccgaauuugu......................................................................	1	1	seq
.....................Accuguagaaccgaauuugu......................................................................	1	1	seq
.....................cccuguagaaccgaauuugu......................................................................	6	0	seq
.....................cccuguagaaccgaauuugug.....................................................................	2	0	seq
.....................cccuguagaaccgaauuugugG....................................................................	2	1	seq
.....................cccuguagaaccgaauuugugA....................................................................	4	1	seq
.....................cccuguagaaccgaauuugugu....................................................................	16	0	seq
......................ccuguagaaccgaauuug.......................................................................	2	0	seq
......................ccuguagaaccgaauuugu......................................................................	19	0	seq
......................ccuguagaaccgaauuugug.....................................................................	5	0	seq
......................ccuguagaaccgaauuugugG....................................................................	1	1	seq
.......................Auguagaaccgaauuugu......................................................................	1	1	seq
```

Mature Star

```
guuauuacguugucuauauauacccuguagaaccgaauuugugugauauucauauagucacagauucgauucuaggggaauauauggucgaugcaaaaacuucaccguucug
........................cuguagaaccgaauuugu......................................................................	6	0	seq
........................cuguagaaccgaauuugug.....................................................................	2	0	seq
.........................uguagaaccgaauuugug.....................................................................	1	0	seq
.........................uguagaaccgaauuugugG....................................................................	1	1	seq
...........................................................acagauucgauucuaggggaC................................	1	1	seq
...........................................................acagauucgauucuaggggaau...............................	19	0	seq
............................................................cagauucgauucuaggggaa................................	1	0	seq
............................................................cagauucgauucAaggggaau...............................	1	1	seq
............................................................cagauucgauucuaggggaau...............................	8	0	seq
............................................................cagauucgauucuaggggaaA...............................	1	1	seq
............................................................cagauucgauucuaggggaaua..............................	2	0	seq
............................................................cagauucgauucuaggggaauU..............................	3	1	seq
.............................................................agauucgauucuaggggaU................................	4	1	seq
.............................................................agauucgauucuaggggaa................................	1	0	seq
.............................................................agauucgauucuaggggaau...............................	38	0	seq
.............................................................agauucgauucuaggggaaA...............................	6	1	seq
.............................................................agauucgauucuaggggaauU..............................	1	1	seq
.............................................................agauucgauucuaggggaaua..............................	2	0	seq
.............................................................agauucgauucuaggggaauau.............................	3	0	seq
```

Provisional ID : mun-miR-101
Score total : 5.5
Score for star read(s) : -1.3
Score for read counts : 0
Score for mfe : 2.2
Score for randfold : 1.6
Score for cons. seed : 3
Total read count : 48834
Mature read count : 48517
Loop read count : 0
Star read count : 317

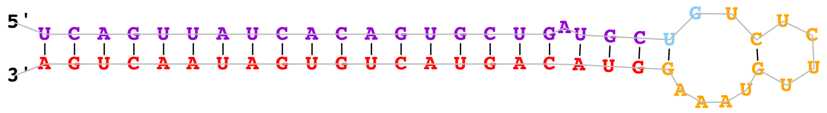

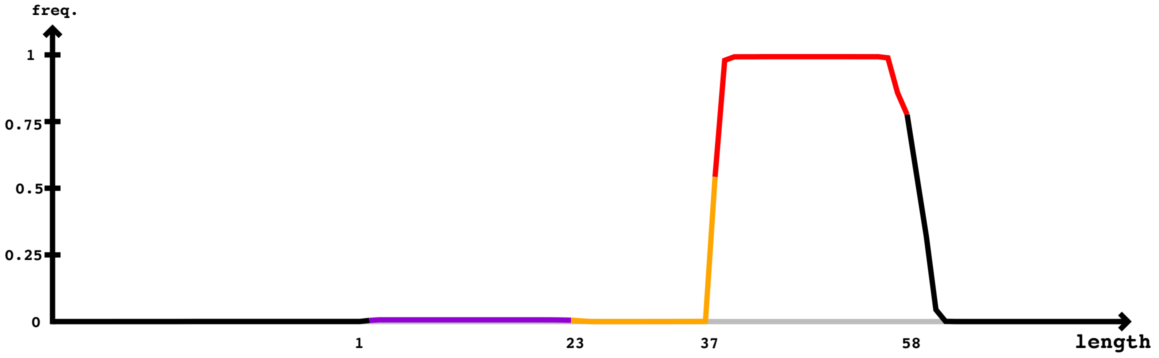

Star **Mature**

```
5'- ggggccgcagugacugacaggcugcccuggcucaguuaucacagugcugaugcugucucuuguaaaggguacaguacugugauaaacugaaggauggcagccaucugagcuu  -3'  obs
    ggggccgcagugacugacaggcugcccuggcucaguuaucacagugcugaugcugucucuuguaaaggguacaguacugugauaaacugaaggauggcagccaucugagcuu       exp
    .(((((.(((...)))...(((((((.....((((((((((((((((((.((((..(....)....))))))))))))))))))))))).....)))))))....).))))  reads  mm  sample
    ....ccgcagugacugacaggcugcccuggc................................................................................  1  0  seq
    .....cgcagugacugacaggcugcccuggc................................................................................  1  0  seq
    .....cgcagugacugacaggcugcccuggcu...............................................................................  1  0  seq
    ..........ugacugacaggcugcccuggc................................................................................  2  0  seq
    ..........ugacugacaggcugcccuggcu...............................................................................  1  0  seq
    ...........gacugacaggcugcccuggcu...............................................................................  1  0  seq
    ............acugacaggcugcccuggc................................................................................  1  0  seq
    ............acugacaggcugcccuggcu...............................................................................  2  0  seq
    .............cugacaggcugcccuggcu...............................................................................  5  0  seq
    ...............................ucaguuaucacagugcug..............................................................  2  0  seq
    ...............................ucaguuaucacagugcuga.............................................................  5  0  seq
    ...............................ucaguuaucacagugcugau............................................................  15  0  seq
    ...............................ucaguuaucacagugcugaug...........................................................  22  0  seq
    ...............................ucaguuaucacagugcugauA...........................................................  6  1  seq
    ...............................Gcaguuaucacagugcugaug...........................................................  1  1  seq
    ...............................ucaguuaucacagugcugaugA..........................................................  1  1  seq
    ...............................ucaguuaucacagugcugaugU..........................................................  1  1  seq
    ...............................ucaguuaucacagugcugaugc..........................................................  116  0  seq
    ...............................ucagGuaucacagugcugaugc..........................................................  1  1  seq
    ...............................ucaguuaucacagugcugaugcu.........................................................  26  0  seq
    ...............................ucaguuaucacagugcugaugcuU........................................................  1  1  seq
    ...............................ucaguuaucacagugcugaugcug........................................................  2  0  seq
    ................................caguuaucacagugcugau............................................................  8  0  seq
    ................................caguuaucacagugcugaug...........................................................  10  0  seq
    ................................caguuaucacagugcugaugc..........................................................  67  0  seq
    ................................caguuaucacagugcugaugU..........................................................  3  1  seq
    ................................caguuaucacagugcugaugA..........................................................  1  1  seq
    ................................caguuaucacagugcugaugcu.........................................................  22  0  seq
    ................................caguuaucacagugcugaugcug........................................................  5  0  seq
    ................................caguuaucacagugcugaugcuU........................................................  2  1  seq
    ...................................uuaucacagugcugaugcugucucuuguaaagg...........................................  1  0  seq
    ............................................................uguaaagguacaguacugugau.............................  2  0  seq
    ............................................................uguaaagguacaguacugugauaacu.........................  1  0  seq
```

Star **Mature**

```
ggggccgcagugacugacaggcugcccuggcucaguuaucacagugcugaugcugucucuuguaaagguacaguacugugauaacugaaggauggcagccaucugagcuu
............................................................uguaaagguacaguacugugauaacugaag....................	1	0	seq
..............................................................uaaagguacaguacugugauaac.........................	2	0	seq
...............................................................aaagguacaguacugugauaacu........................	1	0	seq
................................................................aagguacaguacugugauaacu........................	5	0	seq
.................................................................agguacaguacugugauaacu........................	2	0	seq
..................................................................Uguacaguacugugauaacu........................	1	1	seq
..................................................................Cguacaguacugugauaacu........................	2	1	seq
..................................................................gguacaguacugugauaacu........................	5	0	seq
..................................................................gguacaguacugugauaacug.......................	5	0	seq
..................................................................Cguacaguacugugauaacuga......................	3	1	seq
..................................................................gguacaguacugugauaacuga......................	3	0	seq
...................................................................guacaguacugugauaac.........................	205	0	seq
...................................................................guacaguacGgugauaacu........................	1	1	seq
...................................................................guacaguacuCugauaacu........................	2	1	seq
...................................................................guacaguacuUugauaacu........................	1	1	seq
...................................................................guacaguacugugauaaUu........................	1	1	seq
...................................................................gGacaguacugugauaacu........................	1	1	seq
...................................................................guacaguacugugauaGcu........................	1	1	seq
...................................................................guacaguacugugauaacG........................	11	1	seq
...................................................................guaUaguacugugauaacu........................	1	1	seq
...................................................................Auacaguacugugauaacu........................	3	1	seq
...................................................................guacaguacuguCauaacu........................	2	1	seq
...................................................................guacaUuacugugauaacu........................	3	1	seq
...................................................................guacagGacugugauaacu........................	1	1	seq
...................................................................guacaguacCgugauaacu........................	1	1	seq
...................................................................guacaguCcugugauaacu........................	1	1	seq
...................................................................guacagAacugugauaacu........................	3	1	seq
...................................................................guacaguacugugauaacC........................	1	1	seq
...................................................................gCacaguacugugauaacu........................	3	1	seq
...................................................................guacGguacugugauaacu........................	2	1	seq
...................................................................guaAaguacugugauaacu........................	4	1	seq
...................................................................guacaguacugugauaacu........................	6153	0	seq
...................................................................guacaguacugAgauaacu........................	1	1	seq
...................................................................guacaguacAgugauaacu........................	1	1	seq
...................................................................Cuacaguacugugauaacu........................	2	1	seq
...................................................................Uuacaguacugugauaacu........................	2	1	seq
...................................................................guacaguacugugauaacA........................	2	1	seq
...................................................................guacaguacugCgauaacu........................	1	1	seq
...................................................................guacaguacuguAauaacu........................	1	1	seq
...................................................................guacaguacugGgauaacug.......................	1	1	seq
...................................................................guacaUuacugugauaacug.......................	2	1	seq
...................................................................Uuacaguacugugauaacug.......................	2	1	seq
...................................................................guacaguaGugugauaacug.......................	1	1	seq
...................................................................guacaguacugugGuaacug.......................	1	1	seq
...................................................................guacaguacugugauaaAug.......................	1	1	seq
...................................................................Auacaguacugugauaacug.......................	4	1	seq
...................................................................guacagGacugugauaacug.......................	1	1	seq
...................................................................guacaguacuUugauaacug.......................	1	1	seq
...................................................................guacaguacuguCauaacug.......................	1	1	seq
...................................................................guacaguacugugauaacuA.......................	9	1	seq
...................................................................guacaguaUugugauaacug.......................	3	1	seq
...................................................................guacaguacugAgauaacug.......................	2	1	seq
...................................................................gCacaguacugugauaacug.......................	1	1	seq
...................................................................Cuacaguacugugauaacug.......................	3	1	seq
...................................................................guacaguacugugauaacuU.......................	5	1	seq
...................................................................guUcaguacugugauaacug.......................	1	1	seq
...................................................................guacaguacAgugauaacug.......................	1	1	seq
...................................................................guacaguacugugauaacug.......................	3488	0	seq
...................................................................guacaguacugugauaacGg.......................	9	1	seq
...................................................................guacaguacugugauaacugU......................	581	1	seq
...................................................................guacaguacuguCauaacuga......................	2	1	seq
...................................................................guacaguacugCgauaacuga......................	1	1	seq
...................................................................guacaguaAugugauaacuga......................	2	1	seq
...................................................................Cuacaguacugugauaacuga......................	2	1	seq
...................................................................guacaguacugugUuaacuga......................	1	1	seq
...................................................................guacaguacugugauaUcuga......................	2	1	seq
...................................................................guacaguaUugugauaacuga......................	4	1	seq
...................................................................guacaguacugugaCaacuga......................	2	1	seq
...................................................................guacaguacugugauaacuUa......................	1	1	seq
...................................................................guacaguacugGgauaacuga......................	2	1	seq
```

```
                                                 Star                              Mature
ggggccgcagugacugacaggcugcccuggcucaguuaucacagugcugaugcugucucuuguaaaagguacaguacugugauaacugaaggauggcagccaucugagcuu
....................................................................guacagGacugugauaacuga......................   1      1      seq
....................................................................guacaguacugugGuaacuga......................   1      1      seq
....................................................................guacagAacugugauaacuga......................   2      1      seq
....................................................................guacagCacugugauaacuga......................   5      1      seq
....................................................................guacaguacAgugauaacuga......................   2      1      seq
....................................................................guacaguacugugauaGcuga......................   3      1      seq
....................................................................guacaguacuAugauaacuga......................   1      1      seq
....................................................................guacaguacugugauaacAga......................   1      1      seq
....................................................................Uuacaguacugugauaacuga......................   1      1      seq
....................................................................Auacaguacugugauaacuga......................   5      1      seq
....................................................................guacaguacugugauaacugG......................   75     1      seq
....................................................................gCacaguacugugauaacuga......................   1      1      seq
....................................................................guacaguacugugauaacCga......................   1      1      seq
....................................................................guacaguacuUugauaacuga......................   2      1      seq
....................................................................guacaUuacugugauaacuga......................   2      1      seq
....................................................................guacaguacugugauaacugC......................   5      1      seq
....................................................................guacaAuacugugauaacuga......................   1      1      seq
....................................................................guacaguacCgugauaacuga......................   3      1      seq
....................................................................guacaguaGugugauaacuga......................   1      1      seq
....................................................................guacaguacugugauaaUuga......................   1      1      seq
....................................................................guacaguacugugauaacuga......................   9581   0      seq
....................................................................guacaguacugugauaaAuga......................   2      1      seq
....................................................................guacaguacugAgauaacuga......................   4      1      seq
....................................................................guacaguacuguAauaacuga......................   1      1      seq
....................................................................guaAaguacugugauaacugaa.....................   2      1      seq
....................................................................guacagGacugugauaacugaa.....................   1      1      seq
....................................................................guGcaguacugugauaacugaa.....................   1      1      seq
....................................................................guacaguacuguUauaacugaa.....................   1      1      seq
....................................................................Cuacaguacugugauaacugaa.....................   1      1      seq
....................................................................guacaguacugugauaGcugaa.....................   1      1      seq
....................................................................Auacaguacugugauaacugaa.....................   3      1      seq
....................................................................guacaguacugGgauaacugaa.....................   1      1      seq
....................................................................Uuacaguacugugauaacugaa.....................   3      1      seq
....................................................................guacaguacugugauaacugaG.....................   1      1      seq
....................................................................guacaguacugugauaacugaa.....................   3707   0      seq
....................................................................guacaguacugugauaacugaC.....................   820    1      seq
....................................................................guacaguacugugauaacugaU.....................   444    1      seq
....................................................................guacaguacugugauaacugUa.....................   2      1      seq
....................................................................guacaguGcugugauaacugaa.....................   1      1      seq
....................................................................guacaguacugugauaaUugaa.....................   1      1      seq
....................................................................guacaguaAugugauaacugaa.....................   1      1      seq
....................................................................guacaguacugugaCaacugaa.....................   1      1      seq
....................................................................guacaUuacugugauaacugaa.....................   1      1      seq
....................................................................guacagCacugugauaacugaa.....................   1      1      seq
....................................................................guacaAuacugugauaacugaa.....................   1      1      seq
....................................................................gCacaguacugugauaacugaa.....................   2      1      seq
....................................................................guacaguacGgugauaacugaa.....................   1      1      seq
....................................................................guacaguacugugGuaacugaa.....................   1      1      seq
....................................................................guacaguacugugauaacugaag....................   30     0      seq
....................................................................guacaguacugugauaacugaaU....................   293    1      seq
....................................................................guacaguacugugauaacugaaA....................   840    1      seq
....................................................................guacaguacugugauaacugaaC....................   70     1      seq
....................................................................guacaguacugugauaacugaCg....................   1      1      seq
....................................................................guacaguacugugauaacugaagU...................   1      1      seq
....................................................................guacaguacugugauaacugaaAg...................   1      1      seq
.....................................................................uacagCacugugauaacu........................   1      1      seq
.....................................................................uacaguacugugauaacu........................   164    0      seq
.....................................................................uacaguacugugauaacG........................   1      1      seq
.....................................................................uacagCacugugauaacug.......................   1      1      seq
.....................................................................uacagAacugugauaacug.......................   1      1      seq
.....................................................................uacaguacugugauaacug.......................   461    0      seq
.....................................................................Cacaguacugugauaacuga......................   1      1      seq
.....................................................................Aacaguacugugauaacuga......................   3      1      seq
.....................................................................uacaguacuguAauaacuga......................   1      1      seq
.....................................................................uacaguacugugauaacuUa......................   1      1      seq
.....................................................................uacagGacugugauaacuga......................   1      1      seq
.....................................................................uacaguacugugauaGcuga......................   2      1      seq
.....................................................................uacaguacugugauaaAuga......................   2      1      seq
.....................................................................uacaUuacugugauaacuga......................   1      1      seq
.....................................................................uacaguacugugauaacuga......................   2504   0      seq
```

Star **Mature**

```
ggggccgcagugacugacaggcugcccuggcucaguuaucacagugcugaugcugucucuuguaaagguacaguacugugauaacugaaggauggcagccaucugagcuu
....................................................................uacaguacugugGuaacuga......................  1     1  seq
....................................................................uacaguacugugauaacugC......................  1     1  seq
....................................................................uacaguacuAugauaacuga......................  1     1  seq
....................................................................uacaguacugugauaacugU......................  107   1  seq
....................................................................uacagAacugugauaacuga......................  2     1  seq
....................................................................uacaguacugugUuaacuga......................  2     1  seq
....................................................................uacaguacuguUauaacuga......................  2     1  seq
....................................................................uacaguacuUugauaacuga......................  2     1  seq
....................................................................uacaCuacugugauaacuga......................  1     1  seq
....................................................................uacGguacugugauaacuga......................  1     1  seq
....................................................................uaAaguacugugauaacuga......................  1     1  seq
....................................................................uacaguaUugugauaacuga......................  1     1  seq
....................................................................uacaguacugugauaacGga......................  1     1  seq
....................................................................uacaguacugugauaacugG......................  17    1  seq
....................................................................Gacaguacugugauaacuga......................  1     1  seq
....................................................................uacaguacugugauaGcugaa.....................  2     1  seq
....................................................................uacaguacugugauaaGugaa.....................  1     1  seq
....................................................................uacaguacuCugauaacugaa.....................  1     1  seq
....................................................................uacaguaAugugauaacugaa.....................  2     1  seq
....................................................................uacaguacugugauaacGgaa.....................  1     1  seq
....................................................................uacaguacugugGuaacugaa.....................  2     1  seq
....................................................................Gacaguacugugauaacugaa.....................  4     1  seq
....................................................................uacaguacugugauaacugaa.....................  3295  0  seq
....................................................................uacaguacuUugauaacugaa.....................  1     1  seq
....................................................................uacaguacugugauaacugaG.....................  1     1  seq
....................................................................uacaUuacugugauaacugaa.....................  2     1  seq
....................................................................uacaguacuAugauaacugaa.....................  1     1  seq
....................................................................uacaguacugCgauaacugaa.....................  3     1  seq
....................................................................uacagGacugugauaacugaa.....................  2     1  seq
....................................................................uacaguacugugaCaacugaa.....................  2     1  seq
....................................................................uacagAacugugauaacugaa.....................  1     1  seq
....................................................................Cacaguacugugauaacugaa.....................  2     1  seq
....................................................................uacaguacCgugauaacugaa.....................  1     1  seq
....................................................................uacaguacugugauaacugaC.....................  765   1  seq
....................................................................uacaguacugugauaacCgaa.....................  1     1  seq
....................................................................uacaguacugugauaacuAaa.....................  1     1  seq
....................................................................uacaguUcugugauaacugaa.....................  1     1  seq
....................................................................uacGguacugugauaacugaa.....................  3     1  seq
....................................................................uacagCacugugauaacugaa.....................  1     1  seq
....................................................................uacaguacugugauaacugaU.....................  167   1  seq
....................................................................Aacaguacugugauaacugaa.....................  2     1  seq
....................................................................uacaguaAugugauaacugaag....................  1     1  seq
....................................................................Cacaguacugugauaacugaag....................  4     1  seq
....................................................................uacaCuacugugauaacugaag....................  1     1  seq
....................................................................uacaguacugugauaacugaaC....................  1650  1  seq
....................................................................uacagGacugugauaacugaag....................  2     1  seq
....................................................................uGcaguacugugauaacugaag....................  1     1  seq
....................................................................uacaguGcugugauaacugaag....................  1     1  seq
....................................................................Gacaguacugugauaacugaag....................  2     1  seq
....................................................................uacaguacugugauaacugaCg....................  10    1  seq
....................................................................uacaguacugugauaUcugaag....................  1     1  seq
....................................................................Aacaguacugugauaacugaag....................  3     1  seq
....................................................................uacaguacugugauaacugaaA....................  1974  1  seq
....................................................................uacaguacugugauaacuCaag....................  1     1  seq
....................................................................uacaguacuUugauaacugaag....................  1     1  seq
....................................................................uacaguacugugauaaUugaag....................  1     1  seq
....................................................................uacaguacugugauaacugaag....................  4132  0  seq
....................................................................uacaguacugugauaacugaaU....................  4229  1  seq
....................................................................uacaUuacugugauaacugaag....................  1     1  seq
....................................................................uacagCacugugauaacugaag....................  1     1  seq
....................................................................uacagAacugugauaacugaag....................  1     1  seq
....................................................................uacaguacugugauaacugaGg....................  1     1  seq
....................................................................uacGguacugugauaacugaag....................  1     1  seq
....................................................................uacaguacugugauaacugaagg...................  13    0  seq
....................................................................uacaguacugugauaacugaaAg...................  1     1  seq
....................................................................uacaguacugugauaacugaagC...................  35    1  seq
....................................................................uacaguacugugauaacugaagU...................  1710  1  seq
....................................................................uacaguacugugauaacugaagga..................  1     0  seq
....................................................................uacaguacugugauaacugaagUa..................  11    1  seq
....................................................................uacaguacugugauaacugaagUau.................  1     1  seq
```

```
                               Star                                   Mature
ggggccgcagugacugacaggcugcccuggcucaguuaucacagugcugaugcugucucuuguaaagguacaguacugugauaacugaaggauggcagccaucugagcuu
.....................................................................acaguacugugauaacuga......................      13      0      seq
.....................................................................acaguacugugauaacugU......................      1       1      seq
.....................................................................acaguacugugauaacugaC.....................      2       1      seq
.....................................................................acaguacugugauaacugaa.....................      36      0      seq
.....................................................................acagAacugugauaacugaa.....................      1       1      seq
.....................................................................acaguacugugauaacugaaA....................      26      1      seq
.....................................................................acaguacugugauaaUugaag....................      1       1      seq
.....................................................................acaguacugugauaacugaaU....................      42      1      seq
.....................................................................acaguacugugauaacugaaC....................      4       1      seq
.....................................................................acaguacugugauaacugaag....................      131     0      seq
.....................................................................acaguacugugauaacugaagU...................      359     1      seq
.....................................................................acaguacugugauaacugaagg...................      3       0      seq
.....................................................................acaguacugugauaacugaagC...................      3       1      seq
.....................................................................acaguacugugauaacugaagUa..................      20      1      seq
.....................................................................acaguacugugauaacugaagUau.................      5       1      seq
......................................................................caguacugugauaacugaa.....................      1       0      seq
......................................................................caguacugugauaacugaaU....................      1       1      seq
......................................................................caguacugugauaacugaaA....................      2       1      seq
......................................................................caguacugugauaacugaag....................      1       0      seq
......................................................................caguacugugauaacugaagU...................      3       1      seq
.......................................................................aguacugugauaacugaa.....................      1       0      seq
.......................................................................aguacugugauaacugaagU...................      1       1      seq
........................................................................guacugugauaacugaaU....................      4       1      seq
........................................................................guacugugauaacugaaA....................      1       1      seq
........................................................................guacugugauaacugaag....................      3       0      seq
........................................................................guacugugauaacugaagU...................      4       1      seq
```

Provisional ID	:	mun-miR-219a
Score total	:	10.9
Score for star read(s)	:	3.9
Score for read counts	:	3.7
Score for mfe	:	2.3
Score for randfold	:	1.6
Score for cons. seed	:	-0.6
Total read count	:	19
Mature read count	:	13
Loop read count	:	0
Star read count	:	6

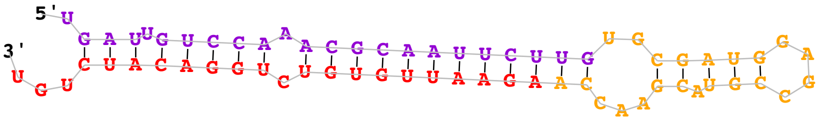

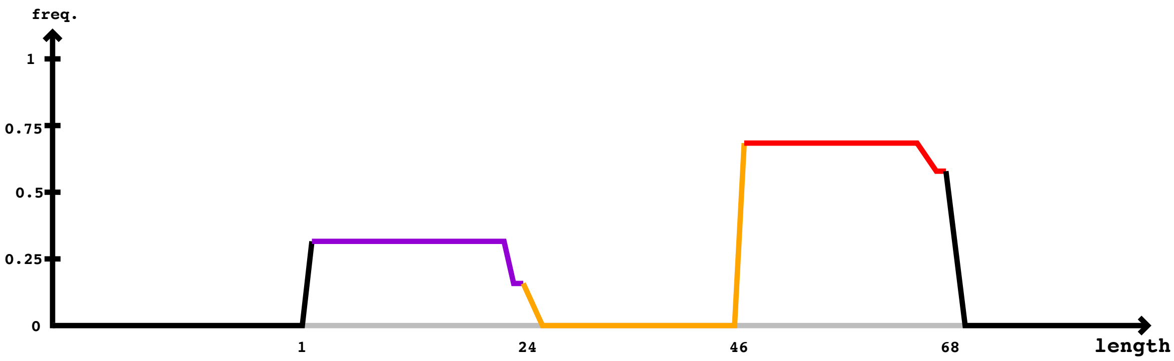

Star Mature

```
5'- agcccgggaugggaaucuccgcuccugauuguccaaacgcaauucuugugcgauggagccguacgaaccaagaauugugucuggacaucuguagcagagguuccgacuccac   -3'    obs
    agcccgggaugggaaucuccgcuccugauuguccaaacgcaauucuugugcgauggagccguacgaaccaagaauugugucuggacaucuguagcagagguuccgacuccac          exp
    .....((..(.((((((((.(((.(.(((.(((((.(((((((((((..(((((....))).))...))))))))))).)))))))).).))).)))))))).)..))..   reads   mm   sample
    .........................ugauuguccaaacgcaauucu..................................................................   3       0    seq
    .........................ugauuguccaaacgcaauucuug................................................................   3       0    seq
    ......................................................................agaauugugucuggacauc.......................   1       0    seq
    ......................................................................agaauugugucuggacaucu......................   1       0    seq
    ......................................................................agaauugugucuggacaucugu....................   11      0    seq
```

Provisional ID	:	mun-novel53
Score total	:	1913
Score for star read(s)	:	3.9
Score for read counts	:	1902.3
Score for mfe	:	2.2
Score for randfold	:	1.6
Score for cons. seed	:	3
Total read count	:	3743
Mature read count	:	3307
Loop read count	:	0
Star read count	:	436

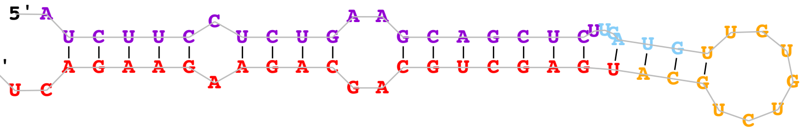

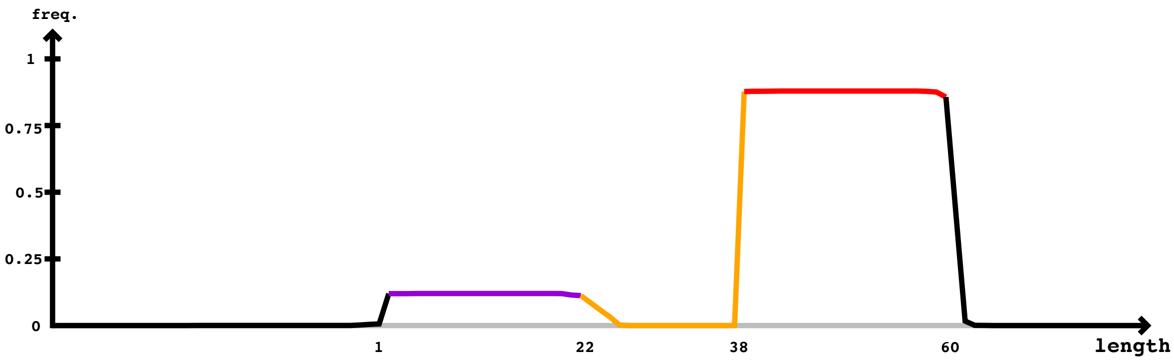

Star **Mature**

```
5'- uucagagcacacugugcagacuuaaaugugaggaucuuccucugaagcagcucuugauguugugucugcaugagcugcagcagaagaagacucucacauuuaagacuucuac   -3'   obs
    uucagagcacacugugcagacuuaaaugugaggaucuuccucugaagcagcucuugauguugugucugcaugagcugcagcagaagaagacucucacauuuaagacuucuac         exp
    ......((((...))))...((((((((((((..(((((.((((..(((((((...((((.......)))))))))))..)))).)))))..))))))))))))........   reads   mm   sample
    .............gugcagacuuaaaugugagg...............................................................................   1       0    seq
    ..............................aggaucuuccucugaagcagcu............................................................   6       0    seq
    ..............................aggaucuuccucugaagcagcuU...........................................................   1       1    seq
    ...............................ggaucuuccucugaagcagc.............................................................   1       0    seq
    ...............................ggaucuuccucugaagcagcu............................................................   2       0    seq
    ...............................ggaucuuccucugaagcagcuc...........................................................   3       0    seq
    ...............................ggaucuuccucugaagcagcucu..........................................................   3       0    seq
    ................................gaucuuccucugaagcagcu............................................................   1       0    seq
    ................................gaucuuccucugaagcagcuc...........................................................   1       0    seq
    ................................gaucuuccucugaagcagcucu..........................................................   3       0    seq
    ................................gaucuuccucugaagcagcucuu.........................................................   1       0    seq
    .................................aucuuccucugaagcagcu............................................................   11      0    seq
    .................................aucuuccucugaagcagcuc...........................................................   3       0    seq
    .................................aucuuccucugaagcagcuU...........................................................   1       1    seq
    .................................aucuuccucugaagcagcucu..........................................................   115     0    seq
    .................................aucuuccucugaagcagcucuu.........................................................   79      0    seq
    .................................aucuuccucugaagcagcucuuC........................................................   1       1    seq
    .................................aucuuccucugaagcagcucuug........................................................   60      0    seq
    .................................aucuuccucugaagcagcucuuU........................................................   33      1    seq
    .................................aucuuccucugaagcagcucuuA........................................................   3       1    seq
    .................................aucuuccucugaagcagcucuuga.......................................................   3       0    seq
    .................................aucuuccucugaagcagcucuugU.......................................................   107     1    seq
    .................................aucuuccucugaagcagcucuugC.......................................................   3       1    seq
    .................................aucuuccucugaagcagcucuugUu......................................................   7       1    seq
    .................................aucuuccucugaagcagcucuugau......................................................   1       0    seq
    .................................aucuuccucugaagcagcucuugCu......................................................   1       1    seq
    .....................................uccucugaagcagcucuuga.......................................................   1       0    seq
    .....................................uccucugaagcagcucuugau......................................................   1       0    seq
    .....................................................................Cugagcugcagcagaagaagacu....................   2       1    seq
    ......................................................................ugagcugcagcagaagaaU.......................   1       1    seq
    ......................................................................ugagcugcagcagaagaag.......................   4       0    seq
    ......................................................................ugagcugcagcagaagaagU......................   1       1    seq
    ......................................................................ugagcugcagcagaagaaga......................   8       0    seq
```

```
                                 Star                                  Mature
uucagagcacacugugcagacuuaaaugugaggaucuucccucugaagcagcucuugauguugugucugcaugagcugcagcagaagaagacucucacauuuaagacuucuac
.......................................................................ugagcugcagcagaagaagac.....................  66    0  seq
.......................................................................ugagcugcagcagaagaagaA.....................  1     1  seq
.......................................................................ugagcugcagcagaagaGgac.....................  2     1  seq
.......................................................................ugagcugcGgcagaagaagac.....................  2     1  seq
.......................................................................ugagcugcagAagaagaagacu....................  1     1  seq
.......................................................................ugagcugcagcagaagaagacG....................  11    1  seq
.......................................................................ugagcugcagcagaagaagacu....................  2373  0  seq
.......................................................................ugagcugcagcGgaagaagacu....................  13    1  seq
.......................................................................ugagcugcagcagaagaaUacu....................  2     1  seq
.......................................................................Agagcugcagcagaagaagacu....................  1     1  seq
.......................................................................ugagcugcagcaCaagaagacu....................  1     1  seq
.......................................................................ugagcugcaUcagaagaagacu....................  1     1  seq
.......................................................................ugagcCgcagcagaagaagacu....................  1     1  seq
.......................................................................ugagcugcagcagaagaGgacu....................  23    1  seq
.......................................................................ugagcugcagcagaGgaagacu....................  181   1  seq
.......................................................................ugagcugcagcagaagaagaUu....................  1     1  seq
.......................................................................ugagcugcagcagaagaagCcu....................  1     1  seq
.......................................................................ugagcugcagcagaUgaagacu....................  1     1  seq
.......................................................................ugagUugcagcagaagaagacu....................  1     1  seq
.......................................................................ugagcugcagcagaagaagacC....................  5     1  seq
.......................................................................ugagcugcagcaUaagaagacu....................  2     1  seq
.......................................................................ugagcugcagcagCagaagacu....................  2     1  seq
.......................................................................ugagcugcagcagaagaagacA....................  12    1  seq
.......................................................................ugagcugcagUagaagaagacu....................  1     1  seq
.......................................................................Ggagcugcagcagaagaagacu....................  4     1  seq
.......................................................................ugagcugcagcagaagaagacuc...................  468   0  seq
.......................................................................ugagcugcagcagaagaagacuU...................  3     1  seq
.......................................................................ugagcugcagcagaagaagacuA...................  1     1  seq
.......................................................................ugagcAgcagcagaagaagacuc...................  1     1  seq
.......................................................................ugagcugcagcagaGgaagacuc...................  26    1  seq
.......................................................................ugagcugcagcGgaagaagacuc...................  10    1  seq
.......................................................................ugagcugcagcagaagaaUacuc...................  1     1  seq
.......................................................................ugagcugcagcagaagaGgacuc...................  3     1  seq
.......................................................................Agagcugcagcagaagaagacuc...................  1     1  seq
.......................................................................ugagcugcagcagaGgaagacucu..................  5     1  seq
.......................................................................ugagcugcagcagaagaagacucu..................  49    0  seq
.......................................................................ugagcugcagcGgaagaagacucu..................  1     1  seq
.......................................................................ugagcugcagcagaagaGgacucu..................  3     1  seq
.......................................................................ugagcugcagcagaagaagacucG..................  1     1  seq
.......................................................................ugagcugcagcagaagaagacucA..................  1     1  seq
........................................................................gagcugcagcagaagaagacu....................  1     0  seq
........................................................................gagcugcagcagaGgaagacu....................  1     1  seq
........................................................................gagcugcagcGgaagaagacuc...................  1     1  seq
........................................................................gagcugcagcagaagaagacuc...................  1     0  seq
.........................................................................gcugcagcagaagaagacuc....................  1     0  seq
.........................................................................gcugcagcagaagaagacucuc..................  1     0  seq
..........................................................................cugcagcagaagaagacucucU.................  2     1  seq
```

Provisional ID : mun-miR-135b
Score total : 271.9
Score for star read(s) : 3.9
Score for read counts : 261.2
Score for mfe : 2.2
Score for randfold : 1.6
Score for cons. seed : 3
Total read count : 524
Mature read count : 510
Loop read count : 0
Star read count : 14

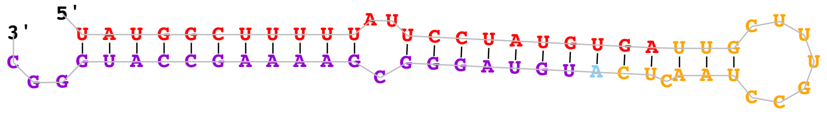

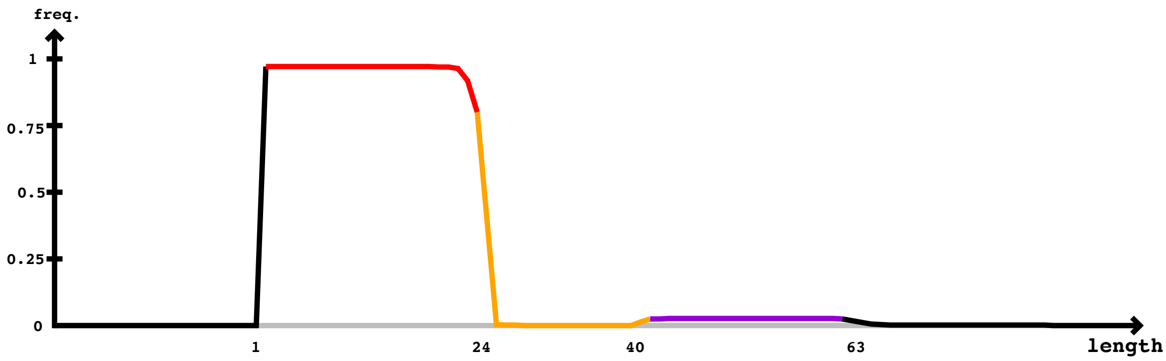

Mature Star

```
5'- guaagcccucugcugugguc uauggcuuuuuauuccuauguga uugcuuugccuaacuca uguagggcgaaaagccaugggc uacacagaggagggacugcaccuuccagg -3' obs
```

```
5'- guaagcccucugcugugguc uauggcuuuuuauuccuaugugauugcuuugccuaacucauguagggcgaaaagccaugggcuacacagaggagggacugcaccuuccagg -3'   obs
    guaagcccucugcuguggucuauggcuuuuuauuccuaugugauugcuuugccuaacucauguagggcgaaaagccaugggcuacacagaggagggacugcaccuuccagg        exp
    (((..((((((.(((((((((((((((((((..(((((((((((((.......))).))))))))))).))))))))))))).....))))).))))))..))).......... reads  mm  sample
    ....................uauggcuuuuuauuccua.........................................................................  1    0   seq
    ....................uauggcuuuuuauuccuaug.......................................................................  3    0   seq
    ....................uauggcuuuuuauuccuaugu......................................................................  24   0   seq
    ....................uauggcuuuuuauuccuauguU.....................................................................  1    1   seq
    ....................uauggcuuuuuauuccuaugug.....................................................................  59   0   seq
    ....................uauggcuuuuuauuccuauguA.....................................................................  2    1   seq
    ....................uauggcuuuuuauuccuauguAa....................................................................  2    1   seq
    ....................uauggcuuuuuauuccuaCguga....................................................................  1    1   seq
    ....................uauggcuuuuuauuccuauguga....................................................................  316  0   seq
    ....................uauggcuuuuuauuccuaugugG....................................................................  25   1   seq
    ....................uauggcuuuuuauuccuaugugU....................................................................  41   1   seq
    ....................uauggcuuuuuauucUuauguga....................................................................  3    1   seq
    ....................uauggcuuuuuauuccuaugugC....................................................................  1    1   seq
    ....................uauggcuuuuuauuccuaugugaC...................................................................  3    1   seq
    ....................uauggcuuuuuauuccuaugugaA...................................................................  9    1   seq
    ....................uauggcuuuuuauuccuaugugau...................................................................  16   0   seq
    ....................uauggcuuuuuauuccuaugugUu...................................................................  1    1   seq
    ....................uauggcuuuuuauuccuaugugaAu..................................................................  1    1   seq
    ....................uauggcuuuuuauuccuaugugauugU................................................................  1    1   seq
    ...........................................................auguagggcgaaaagccaugg...............................  1    0   seq
    ...........................................................auguagggcgaaaagccaugggc.............................  1    0   seq
    ...........................................................auguagggcgaaaagccaugggU.............................  3    1   seq
    ...........................................................auguagggcgaaaagccaugggUu............................  2    1   seq
    ............................................................uguagggcgaaaagccaugggU.............................  4    1   seq
    ............................................................uguagggcgaaaagccaugggc.............................  1    0   seq
    ............................................................uguagggcgaaaagccaugggcuU...........................  1    1   seq
    ..............................................................uagggcgaaaagccaugggcuaU..........................  1    1   seq
    .................................................................................cuacacagaggagggacugca.........  1    0   seq
```

Provisional ID	:	mun-let-7g-2
Score total	:	1.3
Score for star read(s)	:	-1.3
Score for read counts	:	0
Score for mfe	:	-1.9
Score for randfold	:	1.6
Score for cons. seed	:	3
Total read count	:	124
Mature read count	:	124
Loop read count	:	0
Star read count	:	0

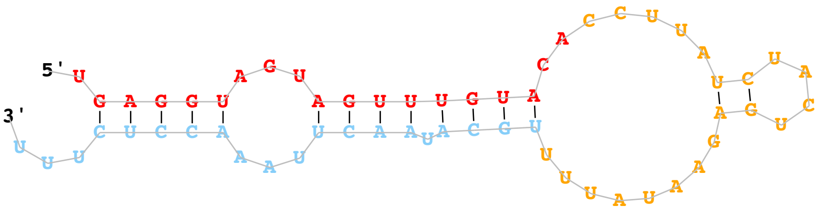

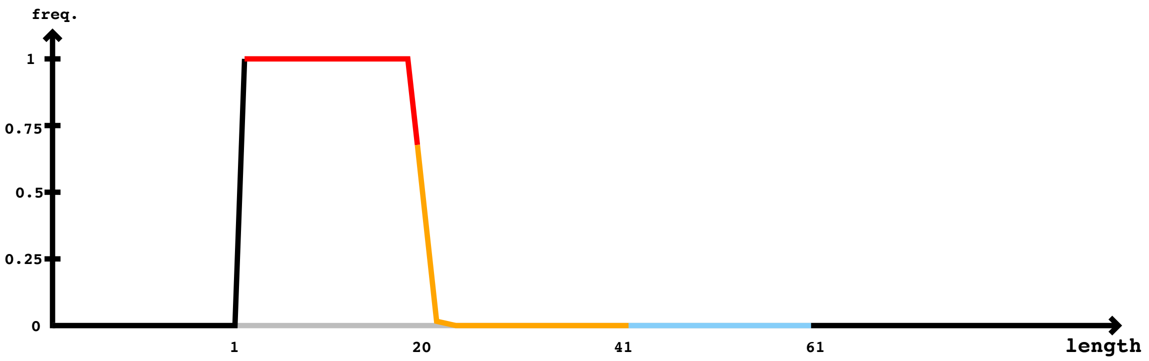

Mature Star

```
5'- agaaaaauagauugcguuugagguaguaguuuguacaccuuaucuacugagaauauuuugcauaacuuaaaccucuuucugcaauuuauuuuguuaccuguccugcaca   -3'    exp
    ...((((((((((((...(((((...((((((((.......((....))........)))).))))...)))))....))))))))))))...................   reads   mm   sample
    ..................ugagguaguaguuuguac.........................................................................   40      0    seq
    ..................ugagguaguaguuuguacU........................................................................   1       1    seq
    ..................ugagguaguaguuuguacG........................................................................   1       1    seq
    ..................ugagguaguaguuuguaca........................................................................   74      0    seq
    ..................ugagguaguaguuuguacaU.......................................................................   3       1    seq
    ..................ugagguaguaguuuguacaA.......................................................................   3       1    seq
    ..................ugagguaguaguuuguacaGc......................................................................   1       1    seq
    ..................ugagguaguaguuuguacacUu.....................................................................   1       1    seq
```

Provisional ID	:	mun-miR-184
Score total	:	5.2
Score for star read(s)	:	3.9
Score for read counts	:	-1.3
Score for mfe	:	1.7
Score for randfold	:	1.6
Score for cons. seed	:	-0.6
Total read count	:	9
Mature read count	:	8
Loop read count	:	0
Star read count	:	1

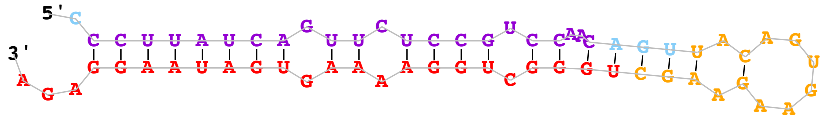

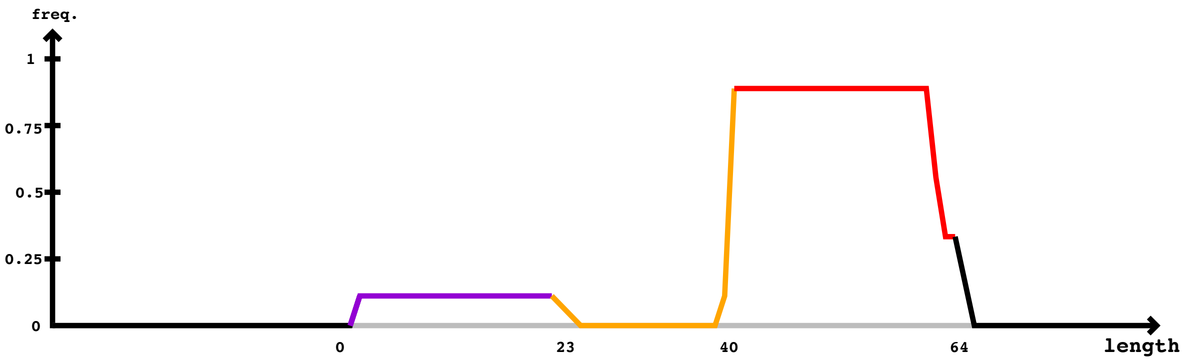

Star Mature

```
5'- gccaggacgaugcucgugccucucgcauacccuuaucaguucuccguccaacaguuacagugaagaagcugggcuggaaaagugauaaggagaugagagcggccucaggagac  -3'   obs
    gccaggacgaugcucgugccucucgcauacccuuaucaguucuccguccaacaguuacagugaagaagcugggcuggaaaagugauaaggagaugagagcggccucaggagac         exp
    .((...(((.....)))(((.(((.(((..((((((((.((.((((.((..(((((.(......).)))))))).)))).)).))))))))..))).))).)))....))....   reads   mm   sample
    ..............................ccuuaucaguucuccguccaac.............................................................   1       0    seq
    ....................................................................cugggcuggaaaagugauaagU.......................   1       1    seq
    .....................................................................ugggcuggaaaagugauaagg.......................   2       0    seq
    .....................................................................ugggcuggaaaagugauaagga......................   2       0    seq
    .....................................................................ugggcuggaaaagugauaaggagU....................   3       1    seq
```

Provisional ID	:	mun-novel28
Score total	:	5.6
Score for star read(s)	:	3.9
Score for read counts	:	-1.3
Score for mfe	:	2.1
Score for randfold	:	1.6
Score for cons. seed	:	-0.6
Total read count	:	9
Mature read count	:	6
Loop read count	:	2
Star read count	:	1

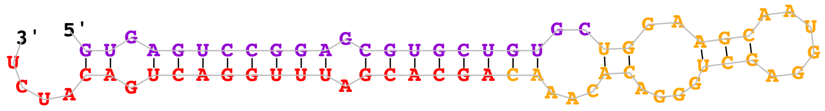

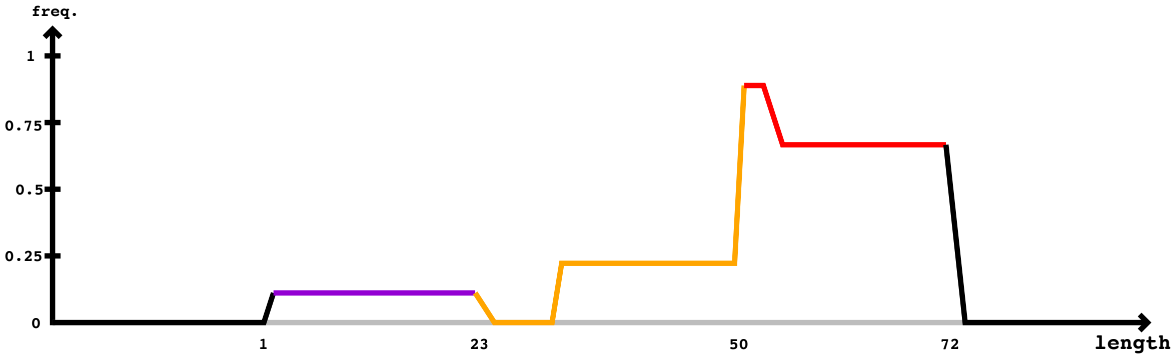


```
                             Star                                                 Mature
5'- ggagccccacggaucugccuggugaguccggagcgugcugugcuggaagcaauggagcugggacacaaacagcacgauuuggacugacaucuguggcgucuguguccucugc   -3'   obs
    ggagccccacggaucugccuggugaguccggagcgugcugugcuggaagcaauggagcugggacacaaacagcacgauuuggacugacaucuguggcgucuguguccucugc         exp
    ((((...(((((((..(((.((((((((((((.((((((((..((..(((......)))....))...)))))))).))))))))..))))...))))))))))..)))).. reads   mm   sample
    .....................gugaguccggagcgugcugugc.....................................................................  1      0    seq
    ...................................................auggagcugggacacaaacagc.......................................  1      0    seq
    ...................................................auggagcugggacacaaacagca......................................  1      0    seq
    ......................................................................agcacgauuuggacugacaucu....................  6      0    seq
```

Provisional ID	:	mun-miR-18b
Score total	:	4.8
Score for star read(s)	:	-1.3
Score for read counts	:	0
Score for mfe	:	1.5
Score for randfold	:	1.6
Score for cons. seed	:	3
Total read count	:	214
Mature read count	:	199
Loop read count	:	3
Star read count	:	12

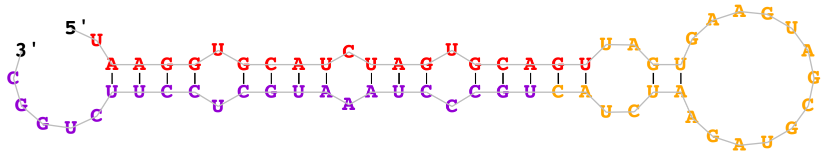

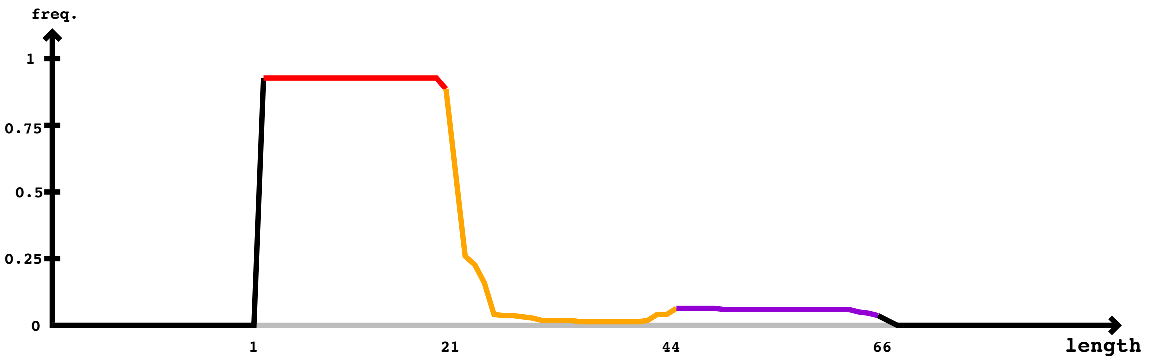

Mature **Star**

```
5'- auugcgaacgguucuuguguuaaggugcaucuagugcaguuagugaaguagcguagaaucuacugcccuaaaugcuccuucuggcacaagcugcugccugcacauccaa  -3'  obs
    auugcgaacgguucuuguguuaaggugcaucuagugcaguuagugaaguagcguagaaucuacugcccuaaaugcuccuucuggcacaagcugcugccugcacauccaa       exp
    ..((((..((((.((((((((((((.((((.(((.(((((..((..............))..))))).))).)))).)))..)))))))))..)))).)))).......  reads  mm  sample
    ....................uaaggugcaucuagugcag......................................................................  8      0   seq
    ....................uaaggugcaucuaguUcag......................................................................  1      1   seq
    ....................uaaggugcaucuagugGagu.....................................................................  1      1   seq
    ....................uaaggugcaucuagugcagu.....................................................................  110    0   seq
    ....................uaaggugcaucuagugUagu.....................................................................  1      1   seq
    ....................uaaggugcaucuagugcaguu....................................................................  27     0   seq
    ....................uaaggugcaucuagugcaguuU...................................................................  2      1   seq
    ....................uaaggugcaucuagugcaguua...................................................................  6      0   seq
    ....................uaaggugcaucuagugcaguuag..................................................................  11     0   seq
    ....................uaaggugcaucuagugcaguuaU..................................................................  4      1   seq
    ....................uaaggugcaucuagugcaguuaA..................................................................  1      1   seq
    ....................uaaggugcaucuagugcaguuaUu.................................................................  2      1   seq
    ....................uaaggugcaucuagugcaguuagA.................................................................  2      1   seq
    ....................uaaggugcaucuagugcaguuagu.................................................................  22     0   seq
    ....................uaaggugcaucuagugcaguuagug................................................................  1      0   seq
    ....................uaaggugcaucuagugcaguuagugaa..............................................................  1      0   seq
    ....................uaaggugcaucuagugcaguuagugaag.............................................................  1      0   seq
    ....................uaaggugcaucuagugcaguuagugaagu............................................................  2      0   seq
    ....................uaaggugcaucuagugcaguuagugaaguagcg........................................................  1      0   seq
    ........................................uagugaaguagcguagaaucu................................................  1      0   seq
    ..........................................gugaaguagcguagaaucu................................................  1      0   seq
    ...........................................ugaaguagcguagaaucuacugccc.........................................  1      0   seq
    ............................................................uacugcccuaaaugcuccuucu...........................  1      0   seq
    .............................................................acugcccuaaaugcuccuucu...........................  1      0   seq
    .............................................................acugcccuaaaugcuccuucug..........................  1      0   seq
    .............................................................acugcccuaaaugcuccuucugA..........................  1      1   seq
    .............................................................acugcccuaaaugcuccuucugU..........................  1      1   seq
    .............................................................acugcccuaaaugcuccuucuggc........................  2      0   seq
    .............................................................acugcccuaaaugcuccuucuggcU.......................  1      1   seq
    ...............................................................ugcccuaaaugcuccuucuggc........................  4      0   seq
    ...............................................................ugcccuaaaugcuccuucuggcU.......................  1      1   seq
```

Provisional ID	:	mun-miR-21
Score total	:	57810
Score for star read(s)	:	3.9
Score for read counts	:	57799.5
Score for mfe	:	2
Score for randfold	:	1.6
Score for cons. seed	:	3
Total read count	:	113383
Mature read count	:	110620
Loop read count	:	4
Star read count	:	2759

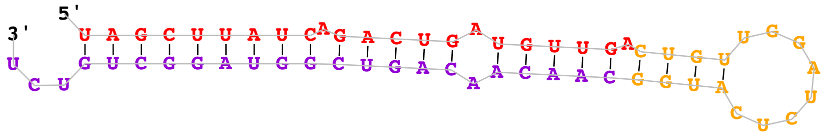

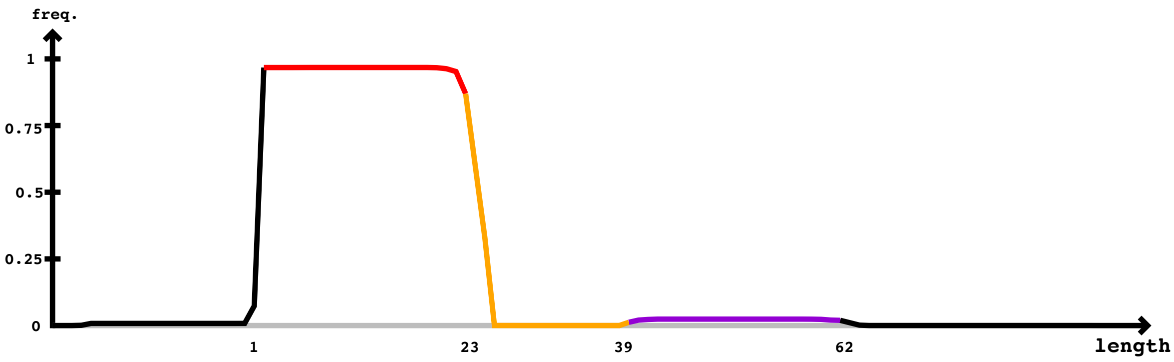

Mature **Star**

```
5'- ggcuguaccauccugucggauagcuuaucagacugauguugacuguuggaucucauggcaacaacagucgguaggcugucugacauuuugguaucuuucaucugaccguuug   -3'    obs
    ggcuguaccauccugucggauagcuuaucagacugauguugacuguuggaucucauggcaacaacagucgguaggcugucugacauuuugguaucuuucaucugaccguuug          exp
    ....(((((...(((((((((((((((.(((((.(((((.((((........)))))))).))))))))))))))))))))...)))))).................   reads   mm   sample
    ggcuguaccauccugucgga............................................................................................   1   0   seq
    .gcuguaccauccugucgg.............................................................................................   3   0   seq
    .gcugGaccauccugucgga............................................................................................   1   1   seq
    .gcuguaccauccugucgga............................................................................................   114   0   seq
    ..cuguaGcauccugucgga............................................................................................   1   1   seq
    ..cuguaccauAcugucgga............................................................................................   1   1   seq
    ..cuguaccUuccugucgga............................................................................................   1   1   seq
    ..Auguaccauccugucgga............................................................................................   1   1   seq
    ..cuguaccauccugucgCa............................................................................................   1   1   seq
    ..cuguaccauccugucggC............................................................................................   1   1   seq
    ..cuguaccauccugucggG............................................................................................   2   1   seq
    ..cuguaccauccugucggU............................................................................................   1   1   seq
    ..cuguaccauccugucgga............................................................................................   785   0   seq
    ..................gauagcuuaucagacugaugu.........................................................................   1   0   seq
    ..................gauagcuuaucagacugauguu........................................................................   7   0   seq
    ...................auagcuuaucagacugau...........................................................................   2   0   seq
    ...................auagcuuaucagacugaug..........................................................................   7   0   seq
    ...................auagcuuaucagacugauA..........................................................................   5   1   seq
    ...................auagcuuaucagacugaugu.........................................................................   45   0   seq
    ...................auagcuuaucagacugaugA.........................................................................   2   1   seq
    ...................auagcuuaucagUcugauguu........................................................................   1   1   seq
    ...................auagcuuaucagacugauguu........................................................................   410   0   seq
    ...................auagcuuaucagacugauguug.......................................................................   1216   0   seq
    ...................Cuagcuuaucagacugauguug.......................................................................   1   1   seq
    ...................auagcuuaucagaUugauguug.......................................................................   1   1   seq
    ...................auagcAuaucagacugauguug.......................................................................   1   1   seq
    ...................auagcuuaucagacugauguuA.......................................................................   37   1   seq
    ...................auagcuuaucagacugauguuU.......................................................................   9   1   seq
    ...................auagcuuaucagacAgauguug.......................................................................   1   1   seq
    ...................auagcuuaucaUacugauguug.......................................................................   1   1   seq
    ...................auagcuuaucagacugauCuug.......................................................................   1   1   seq
    ...................auagAuuaucagacugauguuga......................................................................   3   1   seq
    ...................auagcuuaucagacugauguuga......................................................................   3872   0   seq
```

Mature   Star

```
ggcuguaccauccugucggauagcuuaucagacugauguugacuguuggaucucauggcaacaacagucgguaggcugucugacauuuugguaucuuucaucugaccguuug
...................auagcuuaucagacugaugGuga......................................................................    1     1    seq
...................auagcuuaucagacugaugCuga......................................................................    1     1    seq
...................auagcuuaucagGcugauguuga......................................................................    1     1    seq
...................Guagcuuaucagacugauguuga......................................................................    4     1    seq
...................auagcuuauAagacugauguuga......................................................................    2     1    seq
...................Cuagcuuaucagacugauguuga......................................................................    2     1    seq
...................auagcuuauUagacugauguuga......................................................................    1     1    seq
...................auagcuuaucagacugauguuAa......................................................................    1     1    seq
...................auagcuuaucagacAgauguuga......................................................................    1     1    seq
...................auagcuuaucagacugGuguuga......................................................................    1     1    seq
...................auagcuuaucagacuUauguuga......................................................................    1     1    seq
...................auagcuuaucagacugauguugG......................................................................    12    1    seq
...................auagcuuaucagacugauguugU......................................................................    68    1    seq
...................auagcuuaucagaGugauguuga......................................................................    1     1    seq
...................auagcuuaucagacugaugAuga......................................................................    1     1    seq
...................auagcuuaucagacugauguugC......................................................................    3     1    seq
...................aAagcuuaucagacugauguuga......................................................................    1     1    seq
...................auagcuuaucagacugauguCgac.....................................................................    1     1    seq
...................auagcuuaucagacugauguugaG.....................................................................    1     1    seq
...................Cuagcuuaucagacugauguugac.....................................................................    2     1    seq
...................auagcuuaucagacugauguugac.....................................................................    439   0    seq
...................auagcuuaucagacugauguugaA.....................................................................    34    1    seq
...................auagcuuaucagacugauguugaU.....................................................................    27    1    seq
...................auagcuuaucagacugauguugacA....................................................................    35    1    seq
...................Guagcuuaucagacugauguugacu....................................................................    1     1    seq
...................aAagcuuaucagacugauguugacu....................................................................    1     1    seq
...................auagcuuaucagacugauguugacG....................................................................    3     1    seq
...................auagcuuaucagacugauguugaAu....................................................................    2     1    seq
...................auagUuuaucagacugauguugacu....................................................................    1     1    seq
...................auagcuuaucaCacugauguugacu....................................................................    1     1    seq
...................Cuagcuuaucagacugauguugacu....................................................................    4     1    seq
...................auagcuuaucagacugauguugacC....................................................................    444   1    seq
...................auagcuuaucagacugauguugacu....................................................................    776   0    seq
...................auagcuuaucagacugauguugacuA...................................................................    1     1    seq
...................auagcuuaucagacugauguugacuguuggaucucauggc.....................................................    2     0    seq
....................uagcuuaucagacugauA..........................................................................    12    1    seq
....................uagcuuaucagacugUug..........................................................................    1     1    seq
....................uagcuuaucagacugauU..........................................................................    1     1    seq
....................uagcuuaucagacugaug..........................................................................    64    0    seq
....................uagcuuaucagacugaugC.........................................................................    1     1    seq
....................uagcuuaucaUacugaugu.........................................................................    1     1    seq
....................uagcuuaucagacugaugu.........................................................................    376   0    seq
....................uagcuuaucagacugaugA.........................................................................    4     1    seq
....................uagcuuaucagacugUugu.........................................................................    1     1    seq
....................uagcuuaucaAacugaugu.........................................................................    1     1    seq
....................uagcuuaucagacugaugG.........................................................................    1     1    seq
....................uagcuuauUagacugauguu........................................................................    1     1    seq
....................uagcuuaucagaUugauguu........................................................................    2     1    seq
....................uagcuuaucagaAugauguu........................................................................    2     1    seq
....................uagcuuaucagacugauguu........................................................................    774   0    seq
....................uagcuuauGagacugauguu........................................................................    1     1    seq
....................uCgcuuaucagacugauguu........................................................................    1     1    seq
....................uagcuuaucagacugauCuu........................................................................    1     1    seq
....................uagcuuaucagacugauguA........................................................................    3     1    seq
....................Aagcuuaucagacugauguu........................................................................    1     1    seq
....................uaUcuuaucagacugauguu........................................................................    1     1    seq
....................uagcuuaucagacugaugGu........................................................................    1     1    seq
....................uagcuuaucUgacugauguu........................................................................    1     1    seq
....................uagcuuaucagacugauguuA.......................................................................    46    1    seq
....................uCgcuuaucagacugauguug.......................................................................    1     1    seq
....................uagcuuaucagacugaugAug.......................................................................    1     1    seq
....................uagcuuauAagacugauguug.......................................................................    3     1    seq
....................uagcuuaucagacuAauguug.......................................................................    1     1    seq
....................uagcuuaucagacugaAguug.......................................................................    3     1    seq
....................uagcuuaCcagacugauguug.......................................................................    2     1    seq
....................Aagcuuaucagacugauguug.......................................................................    3     1    seq
....................uagcuuaucagacGgauguug.......................................................................    1     1    seq
....................uGgcuuaucagacugauguug.......................................................................    1     1    seq
....................uagcuuGucagacugauguug.......................................................................    1     1    seq
....................Gagcuuaucagacugauguug.......................................................................    10    1    seq
```

```
                    Mature                  Star
ggcuguaccauccugucggauagcuuaucagacugauguugacuguuggaucucauggcaacaacagucgguaggcugucugacauuuugguaucuuucaucugaccguuug
....................uagAuuaucagacugauguug....................................................................... 1 1 seq
....................uagcuuaucagacugGuguug....................................................................... 2 1 seq
....................uagcuuaucagacugaCguug....................................................................... 1 1 seq
....................uagcuuaucagacCgauguug....................................................................... 1 1 seq
....................uagcuuaucagacugauguug....................................................................... 8109 0 seq
....................uagcuuaucagacugauguCg....................................................................... 1 1 seq
....................uagcuuaucagaUugauguug....................................................................... 7 1 seq
....................uagcuuaucagacugauguGg....................................................................... 3 1 seq
....................Cagcuuaucagacugauguug....................................................................... 4 1 seq
....................uagcuuaucagacugauUuug....................................................................... 3 1 seq
....................uagcuuaucUgacugauguug....................................................................... 3 1 seq
....................uagcuuaucagacugaugCug....................................................................... 2 1 seq
....................uagcuuaucagacugaGguug....................................................................... 1 1 seq
....................uagcuuaucagacugauAuug....................................................................... 3 1 seq
....................uagcuuauUagacugauguug....................................................................... 2 1 seq
....................uagcuuaucagacugauguuU....................................................................... 8 1 seq
....................uagcuuaucaUacugauguug....................................................................... 1 1 seq
....................uaUcuuaucagacugauguug....................................................................... 3 1 seq
....................uagcuuaucagacuCauguug....................................................................... 2 1 seq
....................uagcuuaucagacugaugGug....................................................................... 1 1 seq
....................uagcuuaucagacugaugGuga...................................................................... 4 1 seq
....................uagcuuaucagacCgauguuga...................................................................... 5 1 seq
....................uagcuuaucagacugauguugC...................................................................... 3 1 seq
....................uagcuuaCcagacugauguuga...................................................................... 6 1 seq
....................uagcCuaucagacugauguuga...................................................................... 7 1 seq
....................uagcuuaucagacugauguuUa...................................................................... 5 1 seq
....................uagcuuaucagacugaugCuga...................................................................... 5 1 seq
....................Cagcuuaucagacugauguuga...................................................................... 10 1 seq
....................uagcuuaucagacGgauguuga...................................................................... 2 1 seq
....................uagcuuaucagacugaugAuga...................................................................... 5 1 seq
....................uagcuuaucagaAugauguuga...................................................................... 5 1 seq
....................uagcuGaucagacugauguuga...................................................................... 1 1 seq
....................uagcuuCucagacugauguuga...................................................................... 1 1 seq
....................uagcuuaucUgacugauguuga...................................................................... 1 1 seq
....................uagcuuaucagacugaGguuga...................................................................... 1 1 seq
....................uGgcuuaucagacugauguuga...................................................................... 2 1 seq
....................uagcuuaAcagacugauguuga...................................................................... 4 1 seq
....................uagUuuaucagacugauguuga...................................................................... 2 1 seq
....................uagcuuaucagacugauguAga...................................................................... 2 1 seq
....................uagcuuaucagaUugauguuga...................................................................... 2 1 seq
....................Aagcuuaucagacugauguuga...................................................................... 29 1 seq
....................uagcAuaucagacugauguuga...................................................................... 2 1 seq
....................uagcuuauAagacugauguuga...................................................................... 7 1 seq
....................uagcuuaucaAacugauguuga...................................................................... 1 1 seq
....................uaUcuuaucagacugauguuga...................................................................... 11 1 seq
....................uagcuuaucGgacugauguuga...................................................................... 4 1 seq
....................uagcuuauGagacugauguuga...................................................................... 1 1 seq
....................uagcuuaucagCcugauguuga...................................................................... 1 1 seq
....................uagAuuaucagacugauguuga...................................................................... 4 1 seq
....................uagcuuaGcagacugauguuga...................................................................... 1 1 seq
....................uagcuuaucagacugauAuuga...................................................................... 4 1 seq
....................uUgcuuaucagacugauguuga...................................................................... 4 1 seq
....................uagcuuaucagacuUauguuga...................................................................... 10 1 seq
....................uagcuuaucagacugauCuuga...................................................................... 4 1 seq
....................uagcuuaucagacugaAguuga...................................................................... 4 1 seq
....................uagcuuaucagaGugauguuga...................................................................... 2 1 seq
....................uagcuuauUagacugauguuga...................................................................... 13 1 seq
....................uagcuCaucagacugauguuga...................................................................... 1 1 seq
....................uagcuuaucagacugUuguuga...................................................................... 2 1 seq
....................uagcuuaucagacugauUuuga...................................................................... 7 1 seq
....................Gagcuuaucagacugauguuga...................................................................... 22 1 seq
....................uagcuuaucagacuCauguuga...................................................................... 5 1 seq
....................uagcuuaucagacugauguuAa...................................................................... 8 1 seq
....................uagcuuaucagacAgauguuga...................................................................... 2 1 seq
....................uagcuuaucagacugaCguuga...................................................................... 7 1 seq
....................uaAcuuaucagacugauguuga...................................................................... 3 1 seq
....................uagcuuaucagacugauguCga...................................................................... 1 1 seq
....................uagcuuaucagacugauguugU...................................................................... 102 1 seq
....................uagcuuGucagacugauguuga...................................................................... 3 1 seq
....................uagcuuaucagacuAauguuga...................................................................... 1 1 seq
```

```
                    Mature                                Star
ggcuguaccauccugucggauagcuuaucagacugauguugacuguuggaucucauggcaacaacagucgguaggcugucugacauuuugguaucuuucaucugaccguuug
....................uagcuuaucagUcugauguuga......................................................................  2      1  seq
....................uagcuuaucaCacugauguuga......................................................................  2      1  seq
....................uaCcuuaucagacugauguuga......................................................................  1      1  seq
....................uagcuuaucagacugauguugG......................................................................  127    1  seq
....................uagcuuUucagacugauguuga......................................................................  2      1  seq
....................uagcuuaucaUacugauguuga......................................................................  6      1  seq
....................uagcuuaucagacugGuguuga......................................................................  5      1  seq
....................uagcuuaucagacugauguuga......................................................................  39587  0  seq
....................uagcuuaucCgacugauguuga......................................................................  3      1  seq
....................uagcuuaucagGcugauguugac.....................................................................  1      1  seq
....................uagcuuUucagacugauguugac.....................................................................  1      1  seq
....................uagcuuaucagacugauguugac.....................................................................  16005  0  seq
....................uUgcuuaucagacugauguugac.....................................................................  3      1  seq
....................uagcuuaucagacugauguuAac.....................................................................  1      1  seq
....................uGgcuuaucagacugauguugac.....................................................................  1      1  seq
....................uagcuuaucagCcugauguugac.....................................................................  1      1  seq
....................uagcuuaucagacuCauguugac.....................................................................  3      1  seq
....................uagcuuaucaCacugauguugac.....................................................................  1      1  seq
....................uagcuuaucagacugUuguugac.....................................................................  1      1  seq
....................uaUcuuaucagacugauguugac.....................................................................  4      1  seq
....................uagcuuaucagacugauguugUc.....................................................................  4      1  seq
....................uagcuuaucagacugauUuugac.....................................................................  4      1  seq
....................Aagcuuaucagacugauguugac.....................................................................  10     1  seq
....................uagcuuaucagacugauguugaA.....................................................................  804    1  seq
....................uagcuuaucagacugCuguugac.....................................................................  2      1  seq
....................uagcuuaucagacugauguuCac.....................................................................  2      1  seq
....................uagcuuaucagacugauguugGc.....................................................................  5      1  seq
....................uaAcuuaucagacugauguugac.....................................................................  1      1  seq
....................uagcuuaucagacugauCuugac.....................................................................  1      1  seq
....................uagcuuaucagacuUauguugac.....................................................................  4      1  seq
....................uagUuuaucagacugauguugac.....................................................................  1      1  seq
....................uagcuuaucagacugauguugaU.....................................................................  211    1  seq
....................uagcuuaAcagacugauguugac.....................................................................  2      1  seq
....................uagcuuaucagaUugauguugac.....................................................................  1      1  seq
....................uagcuuaucagacugaAguugac.....................................................................  1      1  seq
....................uagcuuGucagacugauguugac.....................................................................  4      1  seq
....................Cagcuuaucagacugauguugac.....................................................................  6      1  seq
....................uagAuuaucagacugauguugac.....................................................................  1      1  seq
....................uagcuuauUagacugauguugac.....................................................................  4      1  seq
....................uagcuuaucagaAugauguugac.....................................................................  3      1  seq
....................uagcuuaucaUacugauguugac.....................................................................  6      1  seq
....................uagcuuaucagacugaugCugac.....................................................................  1      1  seq
....................uagcuuaucagacugGuguugac.....................................................................  2      1  seq
....................uagcuuauAagacugauguugac.....................................................................  3      1  seq
....................uagcuuaucagUcugauguugac.....................................................................  1      1  seq
....................uagcuuaucagacugaugAugac.....................................................................  1      1  seq
....................uagcuuaucUgacugauguugac.....................................................................  1      1  seq
....................uagcuuaucGgacugauguugac.....................................................................  3      1  seq
....................uagcuuaucagacCgauguugac.....................................................................  2      1  seq
....................uagcuuaucagacugauguuUac.....................................................................  1      1  seq
....................uagcCuaucagacugauguugac.....................................................................  2      1  seq
....................uagcuuaucagacugauguugaG.....................................................................  7      1  seq
....................uagcuuaucagacugaGguugac.....................................................................  1      1  seq
....................Gagcuuaucagacugauguugac.....................................................................  7      1  seq
....................Cagcuuaucagacugauguugacu....................................................................  8      1  seq
....................uagcuuaucagacuUauguugacu....................................................................  2      1  seq
....................uagcuuaucagacGgauguugacu....................................................................  1      1  seq
....................uagcuuaucagacugauguGgacu....................................................................  1      1  seq
....................uagcuuaucCgacugauguugacu....................................................................  1      1  seq
....................uagcuuaucagacugGuguugacu....................................................................  1      1  seq
....................uagcuuaucagacugauUuugacu....................................................................  9      1  seq
....................uagcuuaucagacugaGguugacu....................................................................  1      1  seq
....................uagcuuaucagacugauguugUcu....................................................................  2      1  seq
....................uagcuuaucagacugaAguugacu....................................................................  2      1  seq
....................uagcuuaucagacugauguugacC....................................................................  5630   1  seq
....................uagcuCaucagacugauguugacu....................................................................  7      1  seq
....................Gagcuuaucagacugauguugacu....................................................................  12     1  seq
....................uagcuuaucagacuCauguugacu....................................................................  1      1  seq
....................uagcuuaucagGcugauguugacu....................................................................  3      1  seq
....................uagcuuaucagacugauguugaUu....................................................................  12     1  seq
```

```
                    Mature                                  Star
ggcuguaccauccugucggauagcuuaucagacugauguugacuguuggaucucauggcaacaacagucgguaggcugucugacauuuugguaucuuucaucugaccguuug
....................uagcuuaucagacugauCuugacu....................................................................  5      1  seq
....................uagcuuaucagacugauguuAacu....................................................................  1      1  seq
....................uagcuuaucagaUugauguugacu....................................................................  5      1  seq
....................uagcuuUucagacugauguugacu....................................................................  1      1  seq
....................uagcuuaucagacugauguCgacu....................................................................  2      1  seq
....................uagcuuaucagacugauguAgacu....................................................................  3      1  seq
....................uagcuuauAagacugauguugacu....................................................................  3      1  seq
....................uagcuuaucUgacugauguugacu....................................................................  1      1  seq
....................uaUcuuaucagacugauguugacu....................................................................  4      1  seq
....................uagcuuaucagacugauguugGcu....................................................................  4      1  seq
....................uagcuuaucagacAgauguugacu....................................................................  1      1  seq
....................uagcCuaucagacugauguugacu....................................................................  4      1  seq
....................Aagcuuaucagacugauguugacu....................................................................  20     1  seq
....................uagcuuaucagacugaugGugacu....................................................................  2      1  seq
....................uagcuuaucagacugauguuUacu....................................................................  3      1  seq
....................uagcuuaucagaAugauguugacu....................................................................  6      1  seq
....................uagcuuaucagacugauguugacu....................................................................  27917  0  seq
....................uagcuuaucagacugaugCugacu....................................................................  4      1  seq
....................uagUuuaucagacugauguugacu....................................................................  2      1  seq
....................uagcuuaucagacuAauguugacu....................................................................  3      1  seq
....................uagcuuaucGgacugauguugacu....................................................................  1      1  seq
....................uagcuuauUagacugauguugacu....................................................................  4      1  seq
....................uagcuuGucagacugauguugacu....................................................................  1      1  seq
....................uagcuuaucaCacugauguugacu....................................................................  2      1  seq
....................uagcuuaucagacugauguuCacu....................................................................  2      1  seq
....................uagcuuaucagacCgauguugacu....................................................................  3      1  seq
....................uagcuuaucagacugauguugaGu....................................................................  3      1  seq
....................uagcuuaAcagacugauguugacu....................................................................  1      1  seq
....................uagcuuaucagacugaCguugacu....................................................................  3      1  seq
....................uagcuuaucagacugauAuugacu....................................................................  2      1  seq
....................uagAuuaucagacugauguugacu....................................................................  5      1  seq
....................uagcuuaucaUacugauguugacu....................................................................  6      1  seq
....................uagcuuaucagacugauguugaAu....................................................................  11     1  seq
....................uagcuAaucagacugauguugacu....................................................................  2      1  seq
....................uagcuuaucagacugauguugacG....................................................................  180    1  seq
....................uagcuuaucagacugaugAugacu....................................................................  2      1  seq
....................uGgcuuaucagacugauguugacu....................................................................  1      1  seq
....................uagcuuaucagCcugauguugacu....................................................................  2      1  seq
....................uagcuuaucagacugauguugacA....................................................................  2474   1  seq
....................uagcuuaucagacugauguugacuA...................................................................  2      1  seq
....................uagcuuaucagacugauguugacuU...................................................................  11     1  seq
....................uagcuuaucagacugauguugacuUu..................................................................  2      1  seq
....................uagcuuaucagacugauguugacugu..................................................................  2      0  seq
....................uagcuuaucagacugauguugacuguu.................................................................  3      0  seq
....................uagcuuaucagacugauguugacuguugg...............................................................  2      0  seq
....................uagcuuaucagacugauguugacuguuggaucu...........................................................  1      0  seq
....................uagcuuaucagacugauguugacuguuggaucucauggc.....................................................  1      0  seq
.....................agcuuaucagacugauguug........................................................................  1      0  seq
.....................agcuuaucagacugauguuga......................................................................  2      0  seq
.....................agcuuaucagacugauguugacu....................................................................  3      0  seq
.....................agcuuaucagacugauguugacC....................................................................  1      1  seq
......................gcuuaucagacugauguugacu....................................................................  1      0  seq
......................gcuuaucagacugauguugacC....................................................................  1      1  seq
.......................cuuaucagacugauguugacu....................................................................  1      0  seq
........................uuaucagacugauguuga......................................................................  4      0  seq
........................uuaucagacugauguugac.....................................................................  1      0  seq
........................uuaucagacugauguugaA.....................................................................  1      1  seq
........................uuaucagacugauguugacC....................................................................  1      1  seq
........................uuaucagacugauguugacA....................................................................  1      1  seq
........................uuaucagacugauguugacu....................................................................  1      0  seq
.........................uaucagacugauguugac.....................................................................  4      0  seq
.........................uaucagacugauguugacu....................................................................  1      0  seq
..........................aucagacugauguugacuguu.................................................................  1      0  seq
..............................gacugauguugacuguuggaucucauggU.....................................................  1      1  seq
.........................................acuguuggaucucauggca....................................................  3      0  seq
.........................................acuguuggaucucauggcaU...................................................  1      1  seq
...........................................uguuggaucucauggcaacaacagucgguaggcugucu...............................  3      0  seq
.............................................uuggaucucauggcaacaacagucgguaggcugucu...............................  1      0  seq
....................................................ucauggcaacaacagucggu........................................  1      0  seq
......................................................auggcaacaacagucgguaggcu...................................  1      0  seq
```

Mature **Star**

```
ggcuguaccauccugucggauagcuuaucagacugauguugacuguuggaucucauggcaacaacagucgguaggcugucugacauuuugguaucuuucaucugaccguuug
..........................................................caacaacagucgguaggc....................................	3	0	seq
..........................................................caacaacagucgguaggcu...................................	17	0	seq
..........................................................caacaacagucgguaggcA...................................	1	1	seq
..........................................................caacaacagucgguaggcuU..................................	8	1	seq
..........................................................caacaacagucgguaggcug..................................	50	0	seq
..........................................................caacaacUgucgguaggcugu.................................	1	1	seq
..........................................................Uaacaacagucgguaggcugu.................................	2	1	seq
..........................................................caacaacagucgguaggcuCu.................................	1	1	seq
..........................................................caacaacagucgguaggcGgu.................................	1	1	seq
..........................................................caacaacagucgguaggcugA.................................	50	1	seq
..........................................................caacaacagucgguaggcugu.................................	195	0	seq
..........................................................caacaacagucgguaggcugG.................................	1	1	seq
..........................................................caacaaGagucgguaggcugu.................................	1	1	seq
..........................................................caacaacagucgguaggcugC.................................	7	1	seq
..........................................................caacaacagucgguaggcuAu.................................	14	1	seq
..........................................................caacaacagucgguaggcuUu.................................	20	1	seq
..........................................................Aaacaacagucgguaggcugu.................................	1	1	seq
..........................................................caacaacagucgguaggUugu.................................	1	1	seq
..........................................................caacaaUagucgguaggcugu.................................	1	1	seq
..........................................................caacaacagucgguaggcuguU................................	8	1	seq
..........................................................caacaacGgucgguaggcuguc................................	1	1	seq
..........................................................caacaaUagucgguaggcuguc................................	1	1	seq
..........................................................caacaacaUucgguaggcuguc................................	1	1	seq
..........................................................caacaacagucgguaggcuguc................................	101	0	seq
..........................................................Uaacaacagucgguaggcuguc................................	1	1	seq
..........................................................caacaacagucgguaggcugCc................................	1	1	seq
..........................................................caacaacagGcgguaggcugucu...............................	1	1	seq
..........................................................caacaacagAcgguaggcugucu...............................	1	1	seq
..........................................................caacaacagucggGaggcugucu...............................	1	1	seq
..........................................................caacaacagucgguaggcuUucu...............................	1	1	seq
..........................................................caacaaUagucgguaggcugucu...............................	3	1	seq
..........................................................caacaacagucgguaggcugucu...............................	872	0	seq
..........................................................Aaacaacagucgguaggcugucu...............................	2	1	seq
..........................................................caacaacagucggAaggcugucu...............................	1	1	seq
..........................................................caacaacagucgguaggcuguUu...............................	4	1	seq
..........................................................caacaacagucgguaggcugucC...............................	1	1	seq
..........................................................caacaacagucgguaggcugucA...............................	15	1	seq
..........................................................caacaacagucgguaggcugucG...............................	5	1	seq
..........................................................caacaacagucgguaggcuguAu...............................	1	1	seq
..........................................................caacaacagucggCaggcugucu...............................	1	1	seq
..........................................................caacaacagucgguaggcugucAg..............................	1	1	seq
..........................................................caacaacagucgguaggcugucuU..............................	2	1	seq
...........................................................aacaacagucgguaggcu...................................	2	0	seq
...........................................................aacaacagucggCaggcu...................................	1	1	seq
...........................................................aacaacagucgguaggcuU..................................	4	1	seq
...........................................................aacaacagucgguaggcug..................................	12	0	seq
...........................................................aacaacagucgguaggcuUu.................................	2	1	seq
...........................................................aacaacagucgguaggcugA.................................	1	1	seq
...........................................................aacaacagucgguaggcugu.................................	8	0	seq
...........................................................aacaacagucgguaggcuguc................................	8	0	seq
...........................................................aacaacagucgguaggcugAcu...............................	1	1	seq
...........................................................aacaacagucgguaggcugucu...............................	888	0	seq
...........................................................aacaacagucgguagCcugucu...............................	1	1	seq
...........................................................aacaacagucgguaggcugucA...............................	1	1	seq
...........................................................aacaacagucgguaggcuUucu...............................	3	1	seq
...........................................................Uacaacagucgguaggcugucu...............................	1	1	seq
...........................................................aacaacagucgguaggcugucuU..............................	1	1	seq
............................................................acaacagucgguaggcuAu.................................	1	1	seq
............................................................acaacagucgguaggcugu.................................	1	0	seq
............................................................acaacagucgguaggcuguc................................	3	0	seq
............................................................acaacagucgguaggcugucC...............................	2	1	seq
............................................................acaacagucgguaggcugucu...............................	235	0	seq
............................................................acaacagucgguaggcugucug..............................	6	0	seq
............................................................acaacagucgguaggcugucugU.............................	26	1	seq
.............................................................caacagucgguaggcugucu...............................	1	0	seq
.............................................................caacagucgguaggcugucug..............................	2	0	seq
.............................................................caacagucgguaggcugucugU.............................	143	1	seq
.............................................................caacagucgguaggcugucuga.............................	1	0	seq
.............................................................caacagucgguaggcugucugG.............................	1	1	seq
.............................................................caacagucgguaggcugucugaU............................	1	1	seq
```

```
                    Mature                                  Star
ggcuguaccauccugucggauagcuuaucagacugauguugacuguuggaucucauggcaacaacagucgguaggcugucugacauuuugguaucuuucaucugaccguuug
...............................................................acagucgguaggcugucu...............................    1    0    seq
................................................................................ugacauuuugguaucuuucaucugacU.....    1    1    seq
................................................................................ugacauuuugguaucuuucaucugacc.....    6    0    seq
................................................................................ugacauuuugguaucuuucaucugaccA....    1    1    seq
................................................................................ugacauuuugguaucuuucaucugaccg....    1    0    seq
................................................................................ugacauuuugguaucuuucaucugaccgu...    2    0    seq
.................................................................................gacauuuugguaucuuucaucugaccgu...    1    0    seq
```

Provisional ID	:	mun-novel73
Score total	:	1.8
Score for star read(s)	:	-1.3
Score for read counts	:	0
Score for mfe	:	2.1
Score for randfold	:	1.6
Score for cons. seed	:	-0.6
Total read count	:	13
Mature read count	:	13
Loop read count	:	0
Star read count	:	0

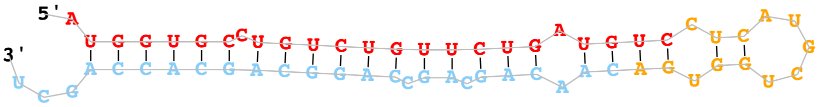

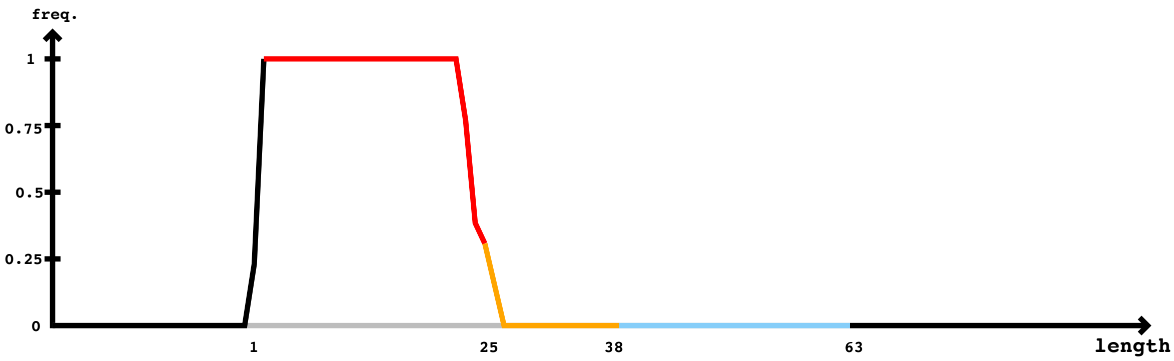

Mature Star

```
5'- ggcaaaggccaggaagcgacauggugccugucuguucugaugucucaugcuggugacaacagcagccaggcagcaccagcugccugguccccuugcaccaaaggauggucau   -3'    exp
    .((((.(((((((.(((....((((((.(((((((((((.((((.((.....)).)))).))).)).))))))))))))))).))))).)).)))).(((.....)))....    reads    mm    sample
    ...................cauggugccugucuguucugau.......................................................................    2    0    seq
    ...................cauggugccugucuguucugauU......................................................................    1    1    seq
    ....................auggugccugucuguucugau.......................................................................    1    0    seq
    ....................auggugccugucuguucugaug......................................................................    4    0    seq
    ....................auggugccugucuguucugaugu.....................................................................    1    0    seq
    ....................auggugccugucuguucugauguc....................................................................    4    0    seq
```

Provisional ID : mun-novel48
Score total : 1.2
Score for star read(s) : -1.3
Score for read counts : 0
Score for mfe : 1.5
Score for randfold : 1.6
Score for cons. seed : -0.6
Total read count : 55
Mature read count : 55
Loop read count : 0
Star read count : 0

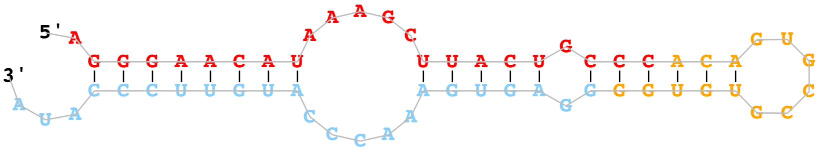

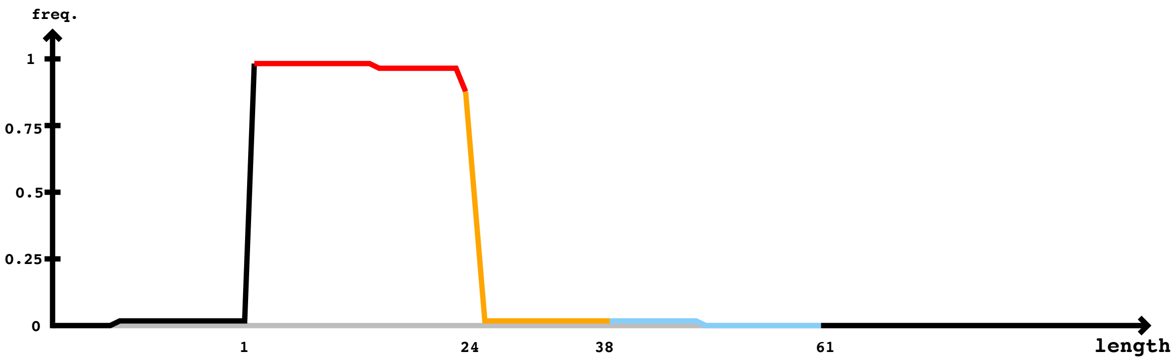

Mature **Star**

```
5'- auaacugugccuacagcaaagggaacauaaagcuuacugcccacagugccguguggggagugaaacccauguucccauaaccccuggaccgggauacacccguuuauacacu  -3'   exp
    ....((((....))))....((((((((.....(((((.((((((......)))))).))))).....))))))))........(((((.(((.....))))))))......   reads   mm   sample
    .....ugugccGacagcaaagggaacauaaag................................................................................   1       1    seq
    ...................agggaacauaaagcuuacugcc.......................................................................   5       0    seq
    ...................agggaacauaaagcuuacugccG......................................................................   1       1    seq
    ...................agggaacauaaagcuuacugccU......................................................................   37      1    seq
    ...................agggaacauaaagcuuacugccc......................................................................   11      0    seq
    ...................agggaacauaaagcuuacugcccU.....................................................................   1       1    seq
    ...........................................caguggccguguggggagugaaac..............................................   1       0    seq
```

Provisional ID : mun-novel15
Score total : 2
Score for star read(s) : -1.3
Score for read counts : 0
Score for mfe : 2.3
Score for randfold : 1.6
Score for cons. seed : -0.6
Total read count : 603
Mature read count : 596
Loop read count : 5
Star read count : 2

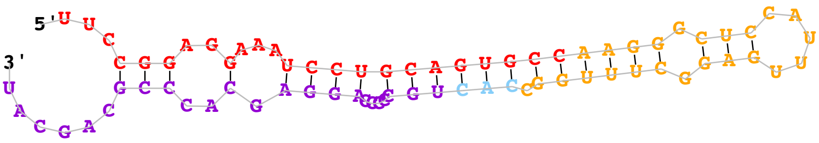

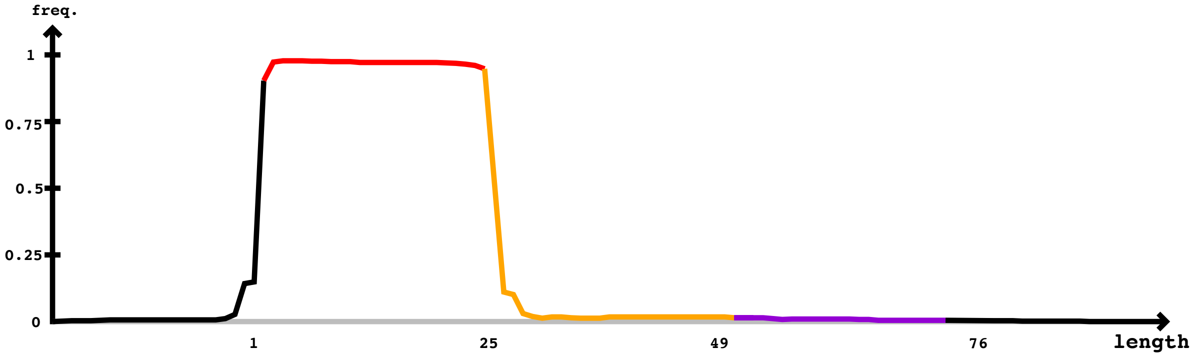


```
                         Mature                                              Star
5'- ugaggagcuuagaggggauauuccggaggaaauccugcagugccaagggcuccauuugaggcuuuggccacugcccccaggagcacccgcagcauccccagcccuccggcucau  -3'   obs
    ugaggagcuuagaggggauauuccggaggaaauccugcagugccaagggcuccauuugaggcuuuggccacugcccccaggagcacccgcagcauccccagcccuccggcucau         exp
    .((((.(((....((((((.((.(((..(...((((((((((((((((.(((.....))).)))))).))))))....)))).)..))).)).)))))))))))))........   reads      mm      sample
    ugaggagcuuagaggggauauuccg.........................................................................................   1          0       seq
    ugaggagcuuagaggggauauuccgga........................................................................................   1          0       seq
    ...ggagcuuagaggggauauuccggagga....................................................................................   1          0       seq
    ....gagcuuagaggggauauuccggagga....................................................................................   1          0       seq
    ................gauauuccggaggaaauccugcagugcc......................................................................   3          0       seq
    .................auauuccggaggaaauccugcagugcc......................................................................   6          0       seq
    .................auauuccggaggaaauccugcagugccU.....................................................................   1          1       seq
    .................auauuccggaggaaauccugcagugcca.....................................................................   3          0       seq
    ..................uauuccggaggaaauccugca...........................................................................   1          0       seq
    ..................uauuccggaggaaauccugcag..........................................................................   1          0       seq
    ..................uauuccggaggaaauccugcagu.........................................................................   2          0       seq
    ..................uauuccggaggaaauccugcagugc.......................................................................   1          0       seq
    ..................uauuccggaggaaauccugAagugcc......................................................................   1          1       seq
    ..................uauuccggaggaaauccugcagugcc......................................................................   23         0       seq
    ..................uauuccggaggaaGuccugcagugcc......................................................................   2          1       seq
    ..................uauuccggaggaaauccugcagAgcca.....................................................................   1          1       seq
    ..................uauuccggaggaaauccugcagugccG.....................................................................   1          1       seq
    ..................uauuccggaggaaauccugcagugccU.....................................................................   1          1       seq
    ..................uauuccggaggaaauccugcagugUca.....................................................................   1          1       seq
    ..................Gauuccggaggaaauccugcagugcca.....................................................................   1          1       seq
    ..................uauuccggaggaaGuccugcagugcca.....................................................................   2          1       seq
    ..................uauuAcggaggaaauccugcagugcca.....................................................................   1          1       seq
    ..................uauuccggaggaaauccugcagugcca.....................................................................   28         0       seq
    ..................uauuccggaggaaauccugcagugUcaag...................................................................   2          1       seq
    ..................uauuccggaggaaauccugcagugccaag...................................................................   1          0       seq
    ..................uauuccggaggaaauccugcagugccaaA...................................................................   1          1       seq
    ..................uauuccggaggaaauccugcagugccaagg..................................................................   1          0       seq
    ..................uauuccggaggaaauccugcagugccaagggc................................................................   1          0       seq
    ...................auccggaggaaauccugcagugcc.......................................................................   1          0       seq
    ...................auccggaggaaauccugcagugcca......................................................................   2          0       seq
    ...................auccggaggaaauccugcagugccaaA....................................................................   1          1       seq
    ....................uuccggaggaaGuccugcagug........................................................................   1          1       seq
    ....................uuccggaggaaauccugcagug........................................................................   2          0       seq
```

Mature **Star**

```
ugaggagcuuagaggggauauuccggaggaaauccugcagugccaagggcuccauuugaggcuuuggccacugcccccaggagcacccgcagcauccccagcccuccggcucau
....................uuccggaggaaauccugcagugc.......................................................................	6	0	seq
....................uuccggaggaaGuccugcagugc.......................................................................	1	1	seq
....................uuccggaggaaGuccugcagugcc......................................................................	11	1	seq
....................uuccggaggaaauccugcagugcU......................................................................	1	1	seq
....................uuccggaggaaauccugcagugcA......................................................................	3	1	seq
....................uuccggaggaaauccugcagugAc......................................................................	1	1	seq
....................Guccggaggaaauccugcagugcc......................................................................	1	1	seq
....................uuccggaggaaauccugcagugcc......................................................................	271	0	seq
....................uuccggaggaaauccugcagugccU.....................................................................	45	1	seq
....................uuccggaggaaauccugcagugUca.....................................................................	1	1	seq
....................uuccggaggaaauccugcagGgcca.....................................................................	1	1	seq
....................uuccggaggaaGuccugcagugcca.....................................................................	7	1	seq
....................uuccggaggaaauccugcagugccG.....................................................................	5	1	seq
....................uuccggaggaaauccugcagugcca.....................................................................	82	0	seq
....................uuccggaggaaauccugcagugccaU....................................................................	4	1	seq
....................uuccggaggaaauccugcagugccaa....................................................................	1	0	seq
....................uuccggaggaaauccugcagugccaag...................................................................	24	0	seq
....................uuccggaggaaauccugcagugAcaag...................................................................	1	1	seq
....................uuccggaggaaauccugcagugccaagU..................................................................	1	1	seq
....................uuccggaggaaGuccugcagugccaagg..................................................................	1	1	seq
....................uuccggaggaaauccugcagugccaagg..................................................................	1	0	seq
....................uuccggaggaaauccugcagugccaaggg.................................................................	3	0	seq
....................uuccggaggaaauccugcagugccaagggcu...............................................................	1	0	seq
....................uuccggaggaaauccugcagugccaagggcucc.............................................................	1	0	seq
.....................uccggaggaaauccugcagugc.......................................................................	1	0	seq
.....................uccggaggaaauccugcagugcc......................................................................	16	0	seq
.....................uccggaggaaauccugcagugccU.....................................................................	2	1	seq
.....................uccggaggaaauccugcagugcca.....................................................................	5	0	seq
.....................uccggaggaaauccugcagugccaa....................................................................	1	0	seq
.....................uccggaggaaGuccugcagugccaag...................................................................	1	1	seq
.....................uccggaggaaauccugcagugccaag...................................................................	13	0	seq
.....................uccggaggaaauccugAagugccaag...................................................................	1	1	seq
.....................uccggaggaaauccugcagugccaagg..................................................................	2	0	seq
.....................uccggaggaaauccugcagugccaaggg.................................................................	1	0	seq
.....................uccggaggaaauccugcagugUcaagggcuc..............................................................	1	1	seq
......................ccggaggaaauccugcagugcca.....................................................................	1	0	seq
......................ccggaggaaauccugcagugccaagA.................................................................	1	1	seq
......................ccggaggaaauccugcagugccaagggcuc..............................................................	1	0	seq
...........................................caagggcuccGuuugaggcuuuggcc.............................................	1	1	seq
............................................aagggcuccGuuugaggcuuuggccac...........................................	1	1	seq
.............................................agggcuccGuuugaggcuuuggcc.............................................	1	1	seq
..................................................uccGuuugaggcuuuggccacug.........................................	1	1	seq
..................................................uccGuuugaggcuuuggccacugc........................................	2	1	seq
..................................................uccGuuugaggcuuuggccacugcccc.....................................	1	1	seq
...................................................ccGuuugaggcuuuggccacug.........................................	1	1	seq
........................................................ugaggcuuuggGcacugcccccagga................................	1	1	seq
........................................................ugaggcuuuggGcacugcccccaggagc..............................	2	1	seq
.......................................................................ugcccccaggagcaGccgcagcau...................	1	1	seq
...........................................................................cccaggagcaGccgcagcaucccc...............	1	1	seq
.............................................................................caggagcaGccgcagcauccccagcccuc........	1	1	seq
```

Provisional ID	:	mun-miR-29b-2
Score total	:	4.6
Score for star read(s)	:	-1.3
Score for read counts	:	0
Score for mfe	:	1.3
Score for randfold	:	1.6
Score for cons. seed	:	3
Total read count	:	606
Mature read count	:	587
Loop read count	:	0
Star read count	:	19

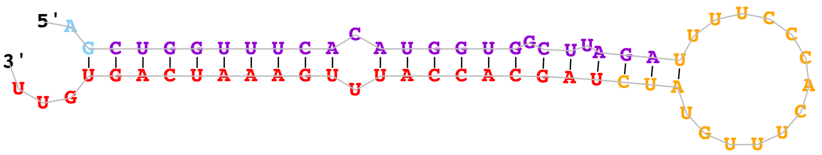

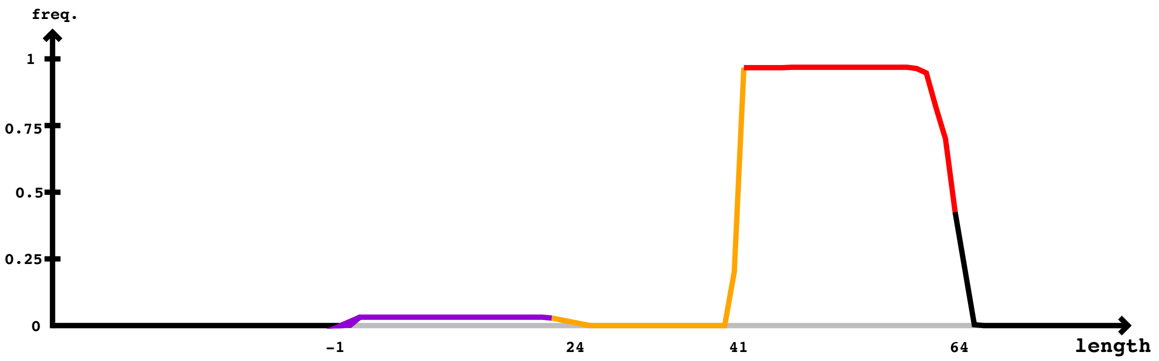


```
                              Star                                    Mature
5'- ccaagcaccagaugugucuuccucuggaagcugguuucacaugguggcuuagauuuucccacuuuguaucuagcaccauuugaaaucaguguucuaggagcaagaauuac  -3'   obs
    ccaagcaccagaugugucuuccucuggaagcugguuucacaugguggcuuagauuuucccacuuuguaucuagcaccauuugaaaucaguguucuaggagcaagaauuac         exp
    ....((((.....))))((((..((((((((((((((((.((((((.((.((((.............)))))))))))).)))))))))).))))))..).)))......  reads  mm  sample
    .............................gcugguuucacaugguggcuuag..........................................................  1      0   seq
    ..............................cugguuucacaugguggcuu............................................................  2      0   seq
    ..............................cugguuucacaugguggcuuaA..........................................................  1      1   seq
    ..............................cugguuucacaugguggcuuag..........................................................  2      0   seq
    ..............................cugguuucacaugguggcuuaga.........................................................  5      0   seq
    ..............................cugguuucacaugguggcuuagU.........................................................  6      1   seq
    ..............................cugguuucacaugguggcuuagau........................................................  2      0   seq
    .....................................................................cuagcaccauuugaaauca......................  2      0   seq
    .....................................................................cuagcaccauuugaaaucag.....................  8      0   seq
    .....................................................................cuagcaccauuugaaaucaA.....................  1      1   seq
    .....................................................................cuagcacUauuugaaaucagu....................  1      1   seq
    .....................................................................cuagcaccauuugaaaucagG....................  1      1   seq
    .....................................................................cuagcaccauuugaaaucagu....................  40     0   seq
    .....................................................................cuagcaccauuugaaaucagug...................  30     0   seq
    .....................................................................cuagcaccauuugaaaucaguU...................  3      1   seq
    .....................................................................cuagcaccauuugaaaucaguA...................  1      1   seq
    .....................................................................cuagcaccauuugaaaucaguC...................  2      1   seq
    .....................................................................cuagcaccauuugaaaucagugu..................  18     0   seq
    .....................................................................Auagcaccauuugaaaucagugu..................  1      1   seq
    .....................................................................cuagcaccauuugaaaucagugA..................  12     1   seq
    .....................................................................cuagcaccauuugaaaucagugG..................  1      1   seq
    .....................................................................cuagcaccauuugaaaucaguguu.................  1      0   seq
    ......................................................................uagcaccauuugaaauca......................  1      0   seq
    ......................................................................uagcaccauuugaaaucag.....................  1      0   seq
    ......................................................................uagcaccauuugaaaucagA....................  1      1   seq
    ......................................................................uagcaccauuugaaaucagu....................  35     0   seq
    ......................................................................uagcaccauuugaaaucaguU...................  3      1   seq
    ......................................................................uagcaccauuugaaaucaguC...................  5      1   seq
    ......................................................................uagcaccauuugaaaucagug...................  24     0   seq
    ......................................................................uagcaccGuuugaaaucagug...................  1      1   seq
    ......................................................................uagcaccauuugaaaucGgug...................  3      1   seq
    ......................................................................uagcaccauuugaaaucagugA..................  9      1   seq
    ......................................................................uagcaccauuugaaaucagugC..................  2      1   seq
```

```
                                   Star                                              Mature
ccaagcaccagaugugucuuccucuggaagcugguuucacaugguggcuuagauuuucccacuuuguaucuagcaccauuugaaaucaguguucuaggagcaagaauuac
......................................................................uagcaccauuugaaaucaguCu..................    3    1    seq
......................................................................uagcaccauuugaaaucagugG..................    5    1    seq
......................................................................uagcaccauuugaaaucaguUu..................    4    1    seq
......................................................................uagcaccauuugaaaucCgugu..................    1    1    seq
......................................................................uagcaccauuugaaaucagugu..................    110  0    seq
......................................................................uagcaccauuugaaaucaguguA.................    3    1    seq
......................................................................uagcaccauuugaaaucagugAu.................    1    1    seq
......................................................................uagcaccauuugaaaucaguguu.................    248  0    seq
......................................................................uagcaccauuugaaaucaguguuU................    1    1    seq
......................................................................uagcaccauuugaaaucaguguuc................    1    0    seq
......................................................................uagcaccauuugaaaucaguguuGu...............    1    1    seq
......................................................................uagcaccauuugaaaucaguguuAu...............    1    1    seq
...........................................................................ccauuugaaaucaguguu.................    1    0    seq
```

Provisional ID : mun-miR-1711
Score total : 596535.8
Score for star read(s) : 3.9
Score for read counts : 596525.1
Score for mfe : 2.2
Score for randfold : 1.6
Score for cons. seed : 3
Total read count : 1170070
Mature read count : 1169548
Loop read count : 0
Star read count : 522

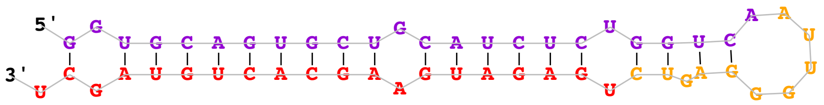

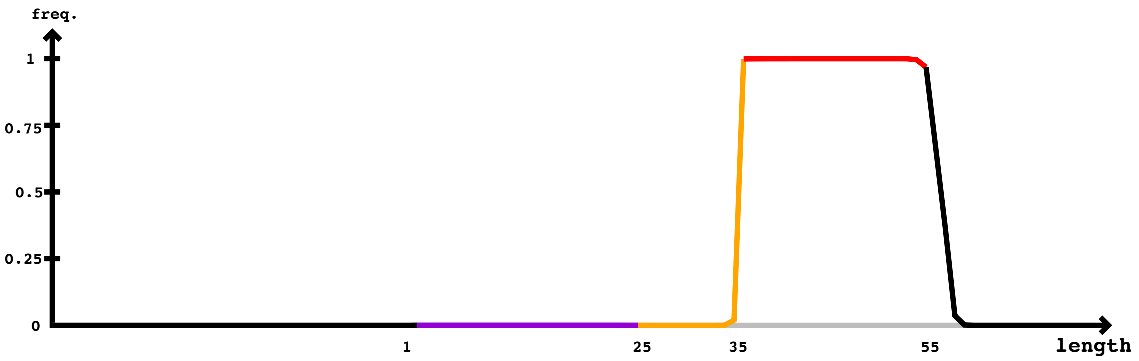

Star Mature

```
5'- ggaugaccacaggcuccuccaugucucccagcccaaggugcagugcugcaucucuggucaauugggagucugagaugaagcacuguagcucgggaagggaggaacugc   -3'   obs
    ggaugaccacaggcuccuccaugucucccagcccaaggugcagugcugcaucucuggucaauugggagucugagaugaagcacuguagcucgggaagggaggaacugc         exp
    (((.((((...)).)).)))...((((((..(((.((.((((((((.((((((.((((......)).)).)))))).)))))))).)).)))..))))))........   reads   mm   sample
    .................uUcaugucucccagcccaa........................................................................   1   1   seq
    ...................................aggugcagugcugcaucuc......................................................   1   0   seq
    ...................................aggugcagugcugcaucucugg...................................................   1   0   seq
    ...................................aggugcagugcugcaucucugU...................................................   2   1   seq
    ...................................aggugcagugcugcaucucuggA..................................................   3   1   seq
    ...................................aggugcagugcugcaucucuggu..................................................   1   0   seq
    ....................................ggugcagugcugcaucuc......................................................   3   0   seq
    ....................................ggugcagugcugcaucucugg...................................................   46   0   seq
    ....................................ggugcagugcugcaucucuggu..................................................   22   0   seq
    ....................................ggugcagugcugcaucucuggA..................................................   36   1   seq
    ....................................ggugcagugcugcaucucuggG..................................................   1   1   seq
    ....................................Agugcagugcugcaucucugguc.................................................   1   1   seq
    ....................................ggugcagugcugcaucucuggAc.................................................   1   1   seq
    ....................................ggugcagugcugcaucucugguA.................................................   17   1   seq
    ....................................ggugcagugcugcaucucugguU.................................................   12   1   seq
    ....................................ggugcagugcugcaucucugguc.................................................   168   0   seq
    ....................................ggugcagugcugcaCcucugguc.................................................   1   1   seq
    ....................................ggugcagugcugcaucucuggucC................................................   1   1   seq
    ....................................ggugcagugcugcaucucuggucU................................................   200   1   seq
    ....................................ggugcagugcugcaucucugguAa................................................   1   1   seq
    ....................................ggugcagugcugcaucucugguca................................................   1   0   seq
    ....................................ggugcagugcugcaucucuggucaauugggaguc......................................   2   0   seq
    .....................................gugcagugcugcaucucugguc.................................................   2   0   seq
    .....................................gugcagugcugcaucucuggucU................................................   1   1   seq
    ...........................................................aauugggagucugagaugaagcacuguagU...................   1   1   seq
    ............................................................auugggagucugagaugaagcacuguagcucU................   1   1   seq
    ...............................................................gggagucugagaugaagcacuguagcuc.................   1   0   seq
    ..................................................................agucugagaugaagcacugua.....................   1   0   seq
    ..................................................................agucugagaugaagcacuguag....................   8   0   seq
    ..................................................................agucugagaugaagcacuguagc...................   5   0   seq
    ..................................................................agucugagaugaagcacuguagU...................   5   1   seq
    ..................................................................agucugagaugaagcacuguagcu..................   10   0   seq
    ..................................................................agucugGgaugaagcacuguagcu..................   1   1   seq
```

```
                                                Star                            Mature
ggaugaccacaggcuccuccaugucucccagcccaaggugcagugcugcaucucuggucaauugggagucugagaugaagcacuguagcucgggaagggaggaacugc
..................................................................agucugagaugaagcacuguagcuc.................   2     0   seq
...................................................................gucugagaugaagcacuguagU...................   2     1   seq
...................................................................gucugagaugaagcacuguagc...................   3     0   seq
...................................................................gucugagaugaagcacuguagA...................   1     1   seq
...................................................................Aucugagaugaagcacuguagc...................   1     1   seq
...................................................................gucugagaugaagcacuguagcA..................   1     1   seq
...................................................................gucugagaugaagcacuguagcu..................   1     0   seq
...................................................................Aucugagaugaagcacuguagcu..................   5     1   seq
....................................................................ucugagaugaagcacuguag....................   18    0   seq
....................................................................ucugagaugaagcacuguaA....................   2     1   seq
....................................................................ucugagaugaagcacuguagc...................   37    0   seq
....................................................................ucugagaugaagcacuguagU...................   9     1   seq
....................................................................ucugagaugaagcacuguagA...................   1     1   seq
....................................................................ucugagaugaagcacuguagcG..................   1     1   seq
....................................................................Gcugagaugaagcacuguagcu..................   1     1   seq
....................................................................ucugagaugaagcacuguagcC..................   1     1   seq
....................................................................ucugagaugaagcacuguagUu..................   9     1   seq
....................................................................ucugagaugaagcacuguagcu..................   37    0   seq
....................................................................ucugagaugaagcacuguagcA..................   3     1   seq
....................................................................ucugagaugaagcacuguagcuG.................   2     1   seq
....................................................................ucugagaugaagcacuguagcuc.................   86    0   seq
....................................................................ucugagaugaagcacuguagcuU.................   38    1   seq
....................................................................ucugagaugaagcacuguagUuc.................   1     1   seq
....................................................................ucugagaugaagcacuguagcucU................   11    1   seq
....................................................................ucugagaugaagcacuguagcucg................   2     0   seq
....................................................................ucugagaugaagcacuguagcucgg...............   1     0   seq
.....................................................................cugagaugaagcacugua.....................   7     0   seq
.....................................................................cugGgaugaagcacuguag....................   1     1   seq
.....................................................................cugagaugaagcacuguaA....................   25    1   seq
.....................................................................cugagaugaagcacuguaU....................   1     1   seq
.....................................................................cugagaugaagcacuguag....................   648   0   seq
.....................................................................Uugagaugaagcacuguag....................   1     1   seq
.....................................................................cugagaugaagcacuguagc...................   1601  0   seq
.....................................................................Gugagaugaagcacuguagc...................   2     1   seq
.....................................................................cugagaugUagcacuguagc...................   1     1   seq
.....................................................................cugagauUaagcacuguagc...................   1     1   seq
.....................................................................cugaUaugaagcacuguagc...................   1     1   seq
.....................................................................Uugagaugaagcacuguagc...................   4     1   seq
.....................................................................cugGgaugaagcacuguagc...................   4     1   seq
.....................................................................cugagaugaagcacuguagA...................   55    1   seq
.....................................................................cugagaugGagcacuguagc...................   2     1   seq
.....................................................................cugagaugaagcacuguagU...................   31    1   seq
.....................................................................cugagaugaagcacugCagc...................   2     1   seq
.....................................................................cugagaugaaUcacuguagc...................   1     1   seq
.....................................................................cugagaugaagcacugAagc...................   1     1   seq
.....................................................................cuAagaugaagcacuguagc...................   1     1   seq
.....................................................................cugaAaugaagcacuguagcu..................   1     1   seq
.....................................................................cugagaugaagcacuguagcG..................   34    1   seq
.....................................................................cugagaugaagcacCguagcu..................   1     1   seq
.....................................................................cugagaugaagcacuguGgcu..................   1     1   seq
.....................................................................cugagaCgaagcacuguagcu..................   1     1   seq
.....................................................................cugagaugaagAacuguagcu..................   1     1   seq
.....................................................................cugagaugaagcacuguaUcu..................   1     1   seq
.....................................................................Gugagaugaagcacuguagcu..................   5     1   seq
.....................................................................cugagaugaaCcacuguagcu..................   3     1   seq
.....................................................................cugagaugaGgcacuguagcu..................   12    1   seq
.....................................................................cugagaugaagcacuguagcu..................   9114  0   seq
.....................................................................cugagaugaagcaAuguagcu..................   1     1   seq
.....................................................................cugagaugaagcacuguagcA..................   82    1   seq
.....................................................................cugagaugaagcacugGagcu..................   3     1   seq
.....................................................................cugagaugaagcacuAuagcu..................   1     1   seq
.....................................................................cugGgaugaagcacuguagcu..................   9     1   seq
.....................................................................cugagaugaagcacugCagcu..................   2     1   seq
.....................................................................cugaUaugaagcacuguagcu..................   3     1   seq
.....................................................................cugagaugaagcacugAagcu..................   2     1   seq
.....................................................................cugagaugGagcacuguagcu..................   5     1   seq
.....................................................................cugagaugaaAcacuguagcu..................   1     1   seq
.....................................................................cugagaugaUgcacuguagcu..................   2     1   seq
.....................................................................cugagauUaagcacuguagcu..................   1     1   seq
.....................................................................cugagaugaagcGcuguagcu..................   1     1   seq
```

Star **Mature**

```
ggaugaccacaggcuccuccaugucucccagcccaaggugcagugcugcaucucuggucaauugggagucugagaugaagcacuguagcucgggaagggaggaacugc
.....................................................................cugagauCaagcacuguagcu..................  1     1   seq
.....................................................................cugagGugaagcacuguagcu..................  1     1   seq
.....................................................................cugagaugaagcacuCuagcu..................  1     1   seq
.....................................................................Uugagaugaagcacuguagcu..................  10    1   seq
.....................................................................cugagaugaagUacuguagcu..................  9     1   seq
.....................................................................cugagaugaCgcacuguagcu..................  1     1   seq
.....................................................................cCgagaugaagcacuguagcu..................  3     1   seq
.....................................................................cugagaugaaUcacuguagcu..................  3     1   seq
.....................................................................cugagaugaagcacuguagUu..................  9     1   seq
.....................................................................cugagaugaagcacuguagcC..................  5     1   seq
.....................................................................Augagaugaagcacuguagcu..................  7     1   seq
.....................................................................cugagaugaagcacuguagUuc.................  6     1   seq
.....................................................................Gugagaugaagcacuguagcuc.................  4     1   seq
.....................................................................cugagaugaagcacugCagcuc.................  1     1   seq
.....................................................................cugagaugaagcacuguaAcuc.................  1     1   seq
.....................................................................cugGgaugaagcacuguagcuc.................  4     1   seq
.....................................................................cugagaugGagcacuguagcuc.................  2     1   seq
.....................................................................cugagaugaagcacuCuagcuc.................  1     1   seq
.....................................................................cugagGugaagcacuguagcuc.................  1     1   seq
.....................................................................Uugagaugaagcacuguagcuc.................  1     1   seq
.....................................................................cugagaugaagcacuguagcuA.................  69    1   seq
.....................................................................cugagaCgaagcacuguagcuc.................  2     1   seq
.....................................................................cugagaugaagcacuAuagcuc.................  3     1   seq
.....................................................................cugagaugaaUcacuguagcuc.................  2     1   seq
.....................................................................cugagaugaagUacuguagcuc.................  2     1   seq
.....................................................................cugagaugaagcUcuguagcuc.................  1     1   seq
.....................................................................cugagaugaagcacuguCgcuc.................  1     1   seq
.....................................................................cugagaugaagcacCguagcuc.................  1     1   seq
.....................................................................cugagaugaagcacuguagcuG.................  10    1   seq
.....................................................................cugagaugaagcacuguagcuU.................  380   1   seq
.....................................................................cugagaugaagcacuguaUcuc.................  1     1   seq
.....................................................................cugagaugaagcaUuguagcuc.................  3     1   seq
.....................................................................cugagaugaGgcacuguagcuc.................  9     1   seq
.....................................................................Augagaugaagcacuguagcuc.................  6     1   seq
.....................................................................cugagaugaagcGcuguagcuc.................  1     1   seq
.....................................................................cugagaugaagcaAuguagcuc.................  1     1   seq
.....................................................................cugagaugaagcacuguagcuc.................  5153  0   seq
.....................................................................cuAagaugaagcacuguagcuc.................  2     1   seq
.....................................................................cugagaugaagcacuguagcCc.................  1     1   seq
.....................................................................cugagaugGagcacuguagcucg................  1     1   seq
.....................................................................cugagaugaagcacuguagcucg................  56    0   seq
.....................................................................cugagaugaagcacuguagcucU................  4367  1   seq
.....................................................................cugagaugaagcacuguagcucA................  192   1   seq
.....................................................................cugagaugaagcacuguagcucC................  59    1   seq
.....................................................................cugagaugaagcacuguagcuAg................  4     1   seq
.....................................................................cugagaugaagcacuguagcuUg................  1     1   seq
.....................................................................cugagaugaagcacuguagcucgU...............  24    1   seq
.....................................................................cugagaugaagcacuguagcucUg...............  2     1   seq
.....................................................................cugagaugaagcacuguagcucgg...............  2     0   seq
......................................................................ugagaugaagcacuUuag....................  1     1   seq
......................................................................ugagaCgaagcacuguag....................  1     1   seq
......................................................................ugagaugaagcacuguaU....................  2     1   seq
......................................................................ugagaugGagcacuguag....................  1     1   seq
......................................................................uUagaugaagcacuguag....................  1     1   seq
......................................................................ugagaugaagcacuguag....................  3500  0   seq
......................................................................ugagaugCagcacuguag....................  2     1   seq
......................................................................ugaCaugaagcacuguag....................  1     1   seq
......................................................................ugagaugaagcacuguaA....................  6     1   seq
......................................................................ugagaugaagcacugAag....................  1     1   seq
......................................................................Cgagaugaagcacuguag....................  2     1   seq
......................................................................ugCgaugaagcacuguag....................  1     1   seq
......................................................................ugaAaugaagcacuguag....................  1     1   seq
......................................................................ugagaugaaCcacuguag....................  3     1   seq
......................................................................ugagauAaagcacuguag....................  1     1   seq
......................................................................ugagaugaagAacuguag....................  1     1   seq
......................................................................ugaUaugaagcacuguag....................  2     1   seq
......................................................................ugagaugaagcGcuguag....................  3     1   seq
......................................................................ugagGugaagcacuguag....................  1     1   seq
......................................................................ugagaugaagcaUuguag....................  1     1   seq
......................................................................ugagaugaagcacuAuag....................  1     1   seq
```

Star Mature

```
ggaugaccacaggcuccuccaugucucccagcccaaggugcagugcugcaucucuggucaauugggagucugagaugaagcacuguagcucgggaagggaggaacugc
......................................................................Ggagaugaagcacuguag....................   1   1   seq
......................................................................ugagaugaagcacugGag....................   2   1   seq
......................................................................ugagCugaagcacuguag....................   1   1   seq
......................................................................ugagaugaagcacugCagc...................   5   1   seq
......................................................................ugagaugaagcacuAuagc...................   2   1   seq
......................................................................ugaUaugaagcacuguagc...................   7   1   seq
......................................................................ugagaCgaagcacuguagc...................   4   1   seq
......................................................................ugagaugaagcacuguagA...................   187   1   seq
......................................................................ugagaugaagcacuguagU...................   515   1   seq
......................................................................ugagaugaCgcacuguagc...................   2   1   seq
......................................................................ugagaugGagcacuguagc...................   83   1   seq
......................................................................ugagaugaagcacAguagc...................   2   1   seq
......................................................................ugagaugaaCcacuguagc...................   5   1   seq
......................................................................ugagaugaagcacugGagc...................   5   1   seq
......................................................................Agagaugaagcacuguagc...................   12   1   seq
......................................................................ugagaugaagcacugAagc...................   5   1   seq
......................................................................ugagaugaaUcacuguagc...................   7   1   seq
......................................................................ugagaugaagcGcuguagc...................   6   1   seq
......................................................................ugagaugaagcacuguagc...................   30017   0   seq
......................................................................uAagaugaagcacuguagc...................   1   1   seq
......................................................................ugagaugaagcacuguGgc...................   2   1   seq
......................................................................ugagaugCagcacuguagc...................   1   1   seq
......................................................................ugagaugaagcacCguagc...................   5   1   seq
......................................................................uUagaugaagcacuguagc...................   6   1   seq
......................................................................ugagaugaagcacuCuagc...................   1   1   seq
......................................................................ugagaugaagAacuguagc...................   7   1   seq
......................................................................ugagaugaagUacuguagc...................   23   1   seq
......................................................................ugagaugaGgcacuguagc...................   77   1   seq
......................................................................Cgagaugaagcacuguagc...................   8   1   seq
......................................................................ugagaugaagcacuguaAc...................   1   1   seq
......................................................................ugagaugaagcUcuguagc...................   2   1   seq
......................................................................ugagaugaagcacuUuagc...................   6   1   seq
......................................................................ugaAaugaagcacuguagc...................   1   1   seq
......................................................................ugagaugaagcacuguagG...................   4   1   seq
......................................................................ugagaugaagcacuguaUc...................   11   1   seq
......................................................................Ggagaugaagcacuguagc...................   22   1   seq
......................................................................ugagaGgaagcacuguagc...................   1   1   seq
......................................................................ugagauUaagcacuguagc...................   4   1   seq
......................................................................ugGgaugaagcacuguagc...................   3   1   seq
......................................................................ugagauAaagcacuguagc...................   4   1   seq
......................................................................ugagaugaaAcacuguagc...................   5   1   seq
......................................................................ugagGugaagcacuguagc...................   2   1   seq
......................................................................uCagaugaagcacuguagc...................   1   1   seq
......................................................................ugagaugaagcaUuguagc...................   1   1   seq
......................................................................ugagaugaagcaGuguagcu..................   15   1   seq
......................................................................ugagaugaagcaUuguagcu..................   34   1   seq
......................................................................ugagaugUagcacuguagcu..................   20   1   seq
......................................................................ugagUugaagcacuguagcu..................   20   1   seq
......................................................................uUagaugaagcacuguagcu..................   89   1   seq
......................................................................uCagaugaagcacuguagcu..................   15   1   seq
......................................................................ugagaugaagcacuguCgcu..................   6   1   seq
......................................................................ugagaugaagcacuguaUcu..................   107   1   seq
......................................................................ugagaugaaCcacuguagcu..................   44   1   seq
......................................................................Agagaugaagcacuguagcu..................   231   1   seq
......................................................................ugagaugaUgcacuguagcu..................   30   1   seq
......................................................................ugagaGgaagcacuguagcu..................   8   1   seq
......................................................................ugagaugaagcacuguagcu..................   412920   0   seq
......................................................................ugaAaugaagcacuguagcu..................   19   1   seq
......................................................................Cgagaugaagcacuguagcu..................   137   1   seq
......................................................................ugagaCgaagcacuguagcu..................   45   1   seq
......................................................................ugagauAaagcacuguagcu..................   26   1   seq
......................................................................Ggagaugaagcacuguagcu..................   280   1   seq
......................................................................ugagaugaagcacAguagcu..................   27   1   seq
......................................................................ugagaugaagcacuguagAu..................   71   1   seq
......................................................................ugagaugaagcacCguagcu..................   44   1   seq
......................................................................ugagaugaagcacuguaAcu..................   20   1   seq
......................................................................uAagaugaagcacuguagcu..................   42   1   seq
......................................................................ugagaugaagGacuguagcu..................   12   1   seq
......................................................................ugagaugaagcacuguGgcu..................   36   1   seq
......................................................................ugaCaugaagcacuguagcu..................   16   1   seq
```

Star Mature

```
ggaugaccacaggcuccuccaugucucccagcccaaggugcagugcugcaucucuggucaauugggagucugagaugaagcacuguagcucgggaagggaggaacugc
......................................................................ugCgaugaagcacuguagcu..................	5	1	seq
......................................................................ugagaugaagcacugAagcu..................	73	1	seq
......................................................................ugagauUaagcacuguagcu..................	78	1	seq
......................................................................ugagaugaagcacuguagcA..................	1265	1	seq
......................................................................ugagaugaagcaAuguagcu..................	49	1	seq
......................................................................ugagaugaaUcacuguagcu..................	69	1	seq
......................................................................ugagaugaCgcacuguagcu..................	17	1	seq
......................................................................ugagaugaagcacuUuagcu..................	84	1	seq
......................................................................ugagaugGagcacuguagcu..................	766	1	seq
......................................................................ugagaugaagcacuguagcG..................	1597	1	seq
......................................................................ugagaugaaAcacuguagcu..................	29	1	seq
......................................................................ugagaugaagcacuguagUu..................	140	1	seq
......................................................................ugagaugaagcacugGagcu..................	42	1	seq
......................................................................ugagaugaagcacugCagcu..................	55	1	seq
......................................................................ugagaugaagcacuguagGu..................	8	1	seq
......................................................................ugagaugaagcacuguUgcu..................	6	1	seq
......................................................................ugUgaugaagcacuguagcu..................	13	1	seq
......................................................................ugagaugaagcCcuguagcu..................	5	1	seq
......................................................................ugagaugaagAacuguagcu..................	91	1	seq
......................................................................ugGgaugaagcacuguagcu..................	33	1	seq
......................................................................ugaUaugaagcacuguagcu..................	73	1	seq
......................................................................ugagaugaagcacuguagcC..................	173	1	seq
......................................................................ugagaugaagcGcuguagcu..................	68	1	seq
......................................................................ugagaugaagUacuguagcu..................	267	1	seq
......................................................................ugagaugCagcacuguagcu..................	10	1	seq
......................................................................ugagCugaagcacuguagcu..................	8	1	seq
......................................................................ugagaugaagcUcuguagcu..................	24	1	seq
......................................................................ugagGugaagcacuguagcu..................	60	1	seq
......................................................................ugagaugaagcacuCuagcu..................	17	1	seq
......................................................................ugagaugaGgcacuguagcu..................	377	1	seq
......................................................................ugagaugaagcacuguaCcu..................	8	1	seq
......................................................................ugagaugaagcacuAuagcu..................	31	1	seq
......................................................................ugagaugaagcacGguagcu..................	11	1	seq
......................................................................ugagaAgaagcacuguagcu..................	8	1	seq
......................................................................ugagauCaagcacuguagcu..................	14	1	seq
......................................................................ugagaugaaUcacuguagcuc.................	37	1	seq
......................................................................ugCgaugaagcacuguagcuc.................	3	1	seq
......................................................................ugagaugaagcacuguagcuU.................	28998	1	seq
......................................................................uCagaugaagcacuguagcuc.................	12	1	seq
......................................................................ugagaugaagGacuguagcuc.................	6	1	seq
......................................................................ugaUaugaagcacuguagcuc.................	57	1	seq
......................................................................ugagauUaagcacuguagcuc.................	31	1	seq
......................................................................ugagaugaagcacuguGgcuc.................	23	1	seq
......................................................................ugagaugaagcacuguagcuc.................	232964	0	seq
......................................................................ugagCugaagcacuguagcuc.................	4	1	seq
......................................................................ugagaugCagcacuguagcuc.................	2	1	seq
......................................................................ugagaugaagcacuguagcAc.................	10	1	seq
......................................................................ugagaugaagAacuguagcuc.................	55	1	seq
......................................................................Agagaugaagcacuguagcuc.................	112	1	seq
......................................................................ugagaugaagcaAuguagcuc.................	34	1	seq
......................................................................ugagGugaagcacuguagcuc.................	32	1	seq
......................................................................ugagaugaGgcacuguagcuc.................	276	1	seq
......................................................................ugUgaugaagcacuguagcuc.................	9	1	seq
......................................................................ugagaugaaCcacuguagcuc.................	15	1	seq
......................................................................ugagaugaagcacuguUgcuc.................	18	1	seq
......................................................................ugagaugUagcacuguagcuc.................	7	1	seq
......................................................................ugaAaugaagcacuguagcuc.................	11	1	seq
......................................................................ugagaugaagcacuguCgcuc.................	1	1	seq
......................................................................ugagaugaagcUcuguagcuc.................	11	1	seq
......................................................................ugagaugaagcacugCagcuc.................	32	1	seq
......................................................................ugagaugaagcacuguaUcuc.................	74	1	seq
......................................................................ugagaugaagcacugGagcuc.................	25	1	seq
......................................................................ugagaugaagcacuguagGuc.................	4	1	seq
......................................................................ugagaugaUgcacuguagcuc.................	14	1	seq
......................................................................uUagaugaagcacuguagcuc.................	41	1	seq
......................................................................ugagaGgaagcacuguagcuc.................	4	1	seq
......................................................................ugagaugaaAcacuguagcuc.................	20	1	seq
......................................................................Ggagaugaagcacuguagcuc.................	151	1	seq
......................................................................ugagaugaagcacuAuagcuc.................	20	1	seq
......................................................................ugagauCaagcacuguagcuc.................	18	1	seq
```

```
                                          Star                    Mature
ggaugaccacaggcuccuccaugucucccagcccaaggugcagugcugcaucucuggucaauugggagucugagaugaagcacuguagcucgggaagggaggaacugc
......................................................................ugagaugaagcacuguagcuG.................	901	1	seq
......................................................................ugagaugaagcacuguagcCc.................	40	1	seq
......................................................................ugagaugaagcacuguaCcuc.................	7	1	seq
......................................................................ugagaugaagcacuguagUuc.................	358	1	seq
......................................................................ugagaAgaagcacuguagcuc.................	5	1	seq
......................................................................ugagaCgaagcacuguagcuc.................	23	1	seq
......................................................................ugagaugaagcacuUuagcuc.................	50	1	seq
......................................................................ugagaugaagcacuguagcGc.................	6	1	seq
......................................................................ugagaugaagcacuguaAcuc.................	15	1	seq
......................................................................ugagaugaagcacuguagAuc.................	42	1	seq
......................................................................ugagaugaCgcacuguagcuc.................	4	1	seq
......................................................................ugagaugaagcacuguagcuA.................	1972	1	seq
......................................................................Cgagaugaagcacuguagcuc.................	81	1	seq
......................................................................ugagUugaagcacuguagcuc.................	7	1	seq
......................................................................ugaCaugaagcacuguagcuc.................	8	1	seq
......................................................................ugagaugaagcacAguagcuc.................	21	1	seq
......................................................................ugagaugaagcaGuguagcuc.................	4	1	seq
......................................................................ugGgaugaagcacuguagcuc.................	26	1	seq
......................................................................ugagaugGagcacuguagcuc.................	1948	1	seq
......................................................................uAagaugaagcacuguagcuc.................	17	1	seq
......................................................................ugagaugaagUacuguagcuc.................	115	1	seq
......................................................................ugagauAaagcacuguagcuc.................	11	1	seq
......................................................................ugagaugaagcacGguagcuc.................	7	1	seq
......................................................................ugagaugaagcacuCuagcuc.................	22	1	seq
......................................................................ugagaugaagcGcuguagcuc.................	43	1	seq
......................................................................ugagaugaagcacugAagcuc.................	34	1	seq
......................................................................ugagaugaagcacCguagcuc.................	34	1	seq
......................................................................ugagaugaagcaUuguagcuc.................	61	1	seq
......................................................................ugagaugaagcCcuguagcuc.................	5	1	seq
......................................................................ugagaugaagcacuguagcuUg................	35	1	seq
......................................................................ugagaugaaCcacuguagcucg................	7	1	seq
......................................................................ugagaugaagcacugAagcucg................	10	1	seq
......................................................................ugGgaugaagcacuguagcucg................	3	1	seq
......................................................................ugagaugUagcacuguagcucg................	5	1	seq
......................................................................ugUgaugaagcacuguagcucg................	1	1	seq
......................................................................ugagaugaagUacuguagcucg................	9	1	seq
......................................................................uCagaugaagcacuguagcucg................	6	1	seq
......................................................................ugaAaugaagcacuguagcucg................	6	1	seq
......................................................................ugagaugaaAcacuguagcucg................	5	1	seq
......................................................................ugagaugaagcaAuguagcucg................	16	1	seq
......................................................................ugagaugGagcacuguagcucg................	43	1	seq
......................................................................ugagaugaagcacuguUgcucg................	11	1	seq
......................................................................ugagauCaagcacuguagcucg................	5	1	seq
......................................................................ugagaugaagcacuguaCcucg................	6	1	seq
......................................................................ugagaugaagcacuguaUcucg................	21	1	seq
......................................................................ugagaugaagcacuguaAcucg................	1	1	seq
......................................................................ugagaugaagcacuguagcGcg................	1	1	seq
......................................................................ugagUugaagcacuguagcucg................	4	1	seq
......................................................................ugagaugaaUcacuguagcucg................	15	1	seq
......................................................................ugagaAgaagcacuguagcucg................	3	1	seq
......................................................................Agagaugaagcacuguagcucg................	42	1	seq
......................................................................ugagaugaagcacuguagcucg................	72228	0	seq
......................................................................ugaUaugaagcacuguagcucg................	9	1	seq
......................................................................uUagaugaagcacuguagcucg................	10	1	seq
......................................................................ugagaugaUgcacuguagcucg................	2	1	seq
......................................................................ugagaugaagcGcuguagcucg................	15	1	seq
......................................................................ugagaugaagcacuguGgcucg................	7	1	seq
......................................................................ugagaugaagcaUuguagcucg................	2	1	seq
......................................................................ugCgaugaagcacuguagcucg................	1	1	seq
......................................................................ugagaugaagcacuguagcucA................	8510	1	seq
......................................................................uAagaugaagcacuguagcucg................	5	1	seq
......................................................................ugagGugaagcacuguagcucg................	9	1	seq
......................................................................ugagaugaagcacugCagcucg................	8	1	seq
......................................................................Cgagaugaagcacuguagcucg................	26	1	seq
......................................................................ugagaugaagcacuguagcucC................	3612	1	seq
......................................................................Ggagaugaagcacuguagcucg................	44	1	seq
......................................................................ugagaugaagcacCguagcucg................	9	1	seq
......................................................................ugagaugaagAacuguagcucg................	13	1	seq
......................................................................ugagaugaagcacuguagAucg................	9	1	seq
......................................................................ugaCaugaagcacuguagcucg................	5	1	seq
```

```
                                                    Star                            Mature
ggaugaccacaggcuccuccaugucucccagcccaaggugcagugcugcaucucuggucaauugggagucugagaugaagcacuguagcucgggaagggaggaacugc
......................................................................ugagaugaagcacugGagcucg................    11      1    seq
......................................................................ugagaugaagcacuguCgcucg................    3       1    seq
......................................................................ugagaugaagGacuguagcucg................    1       1    seq
......................................................................ugagaugaagcacGguagcucg................    2       1    seq
......................................................................ugagaugaagcacuguagUucg................    89      1    seq
......................................................................ugagauAaagcacuguagcucg................    3       1    seq
......................................................................ugagaugaagcacuCuagcucg................    9       1    seq
......................................................................ugagaugaagcacuguagcAcg................    3       1    seq
......................................................................ugagauUaagcacuguagcucg................    9       1    seq
......................................................................ugagaugaagcacuguagcuGg................    5       1    seq
......................................................................ugagaugaagcacuguagcuAg................    56      1    seq
......................................................................ugagaugaagcacuguagcucU................    295852  1    seq
......................................................................ugagaugaagcacuAuagcucg................    4       1    seq
......................................................................ugagaugaagcacuUuagcucg................    12      1    seq
......................................................................ugagaCgaagcacuguagcucg................    10      1    seq
......................................................................ugagaugaagcaGuguagcucg................    2       1    seq
......................................................................ugagaugaagcacuguagcCcg................    11      1    seq
......................................................................ugagaugaagcacAguagcucg................    1       1    seq
......................................................................ugagaugaagcUcuguagcucg................    1       1    seq
......................................................................ugagaugaGgcacuguagcucg................    179     1    seq
......................................................................ugagaugaagcacuUuagcucgg...............    1       1    seq
......................................................................ugagaugaagcacuguagcucgC...............    501     1    seq
......................................................................ugagaugaagcacuguagcuAgg...............    1       1    seq
......................................................................ugagaugaagcacCguagcucgg...............    1       1    seq
......................................................................Agagaugaagcacuguagcucgg...............    4       1    seq
......................................................................ugagaugaagcGcuguagcucgg...............    2       1    seq
......................................................................ugagaugaagcacuguaAcucgg...............    1       1    seq
......................................................................ugagaugaGgcacuguagcucgg...............    11      1    seq
......................................................................ugagaugaagcacuguagcucgA...............    1598    1    seq
......................................................................ugagaugaagcacuguagcucgU...............    32411   1    seq
......................................................................ugagaugaagUacuguagcucgg...............    2       1    seq
......................................................................ugagaugaagcacuguagcGcgg...............    1       1    seq
......................................................................ugagaugaagcacuguagcCcgg...............    2       1    seq
......................................................................ugaUaugaagcacuguagcucgg...............    1       1    seq
......................................................................ugagaugaagcacuguagcuUgg...............    9       1    seq
......................................................................ugagaugaagcacuguagcucUg...............    102     1    seq
......................................................................ugagGugaagcacuguagcucgg...............    1       1    seq
......................................................................ugagaugaagcacuguagcucAg...............    16      1    seq
......................................................................ugagaugaagcacuguagcucgg...............    5906    0    seq
......................................................................ugagaugaagcUcuguagcucgg...............    2       1    seq
......................................................................ugagaugaagcacuguagcucCg...............    1       1    seq
......................................................................ugagauUaagcacuguagcucgg...............    2       1    seq
......................................................................uUagaugaagcacuguagcucgg...............    1       1    seq
......................................................................Ggagaugaagcacuguagcucgg...............    4       1    seq
......................................................................ugagaugGagcacuguagcucgg...............    1       1    seq
......................................................................Cgagaugaagcacuguagcucgg...............    1       1    seq
......................................................................ugagaugaagcacuguaUcucgg...............    1       1    seq
......................................................................ugagaugaagcacuguagcucgUg..............    8       1    seq
......................................................................ugagaugaagcacuguagcucggA..............    19      1    seq
......................................................................ugagaugaagcacuguagcucUgg..............    8       1    seq
......................................................................ugagaugaagcacuguagcucggU..............    1141    1    seq
......................................................................ugagaugaagcacuguagcucggC..............    3       1    seq
......................................................................ugagaugaagcacuguagcucggg..............    6       0    seq
......................................................................ugagaugaagcacuguagcuUggga.............    2       1    seq
......................................................................ugagaugaagcacuguagcucUgga.............    3       1    seq
......................................................................ugagaugaagcacuguagcucggAa.............    1       1    seq
......................................................................ugagaugaagcacuguagcucgUga.............    1       1    seq
......................................................................ugagaugaagcacuguagcucggUa.............    10      1    seq
......................................................................ugagaugaagcacuguagcucUggaa............    4       1    seq
......................................................................ugagaugaagcacuguagcucgggaa............    1       0    seq
.......................................................................Uagaugaagcacuguagc...................    1       1    seq
.......................................................................Uagaugaagcacuguagcu..................    3       1    seq
.......................................................................gagaugaagcacuguagcu..................    22      0    seq
.......................................................................gagaugaagcacuguagcuU.................    2       1    seq
.......................................................................Uagaugaagcacuguagcuc.................    1       1    seq
.......................................................................gagaugaagcacuguagcuc.................    13      0    seq
.......................................................................gagaugaagcacuguagcucA................    2       1    seq
.......................................................................gagaugaagcacuguagcucU................    19      1    seq
.......................................................................gagaugaagcacuguagcucg................    12      0    seq
.......................................................................gagaugaagcacuguaUcucgg...............    1       1    seq
```

Star **Mature**

```
ggaugaccacaggcuccuccaugucucccagcccaaggugcagugcugcaucucuggucaauugggagucugagaugaagcacuguagcucgggaagggaggaacugc
.......................................................................gagaugaagcacuguagcucgg...............	258	0	seq
.......................................................................gaCaugaagcacuguagcucgg...............	1	1	seq
.......................................................................gagaugUagcacuguagcucgg...............	1	1	seq
.......................................................................gagaugaagcacuguagcucgA...............	3	1	seq
.......................................................................gagaugaagcacuguagcucgU...............	11	1	seq
.......................................................................gagaugaagcacuguagcucggA..............	3	1	seq
.......................................................................gagaugaagcacuguagcucggU..............	148	1	seq
.......................................................................gagaugaagcacuguagcucggC..............	4	1	seq
........................................................................agaugaagcacuguagcu..................	12	0	seq
........................................................................agaugaagcacuguagcuc.................	2	0	seq
........................................................................Ugaugaagcacuguagcuc.................	1	1	seq
........................................................................agaugaagcacuguagcucU................	10	1	seq
........................................................................agaugaagcacuguagcucg................	8	0	seq
........................................................................agaugaagcacuguagcuUgg...............	1	1	seq
........................................................................agaugaagcacuguagcucgU...............	22	1	seq
........................................................................agaugaagcacuguagcucgg...............	87	0	seq
........................................................................agaugaaAcacuguagcucgg...............	1	1	seq
........................................................................agaugaagcacuguagcucggA..............	1	1	seq
........................................................................agaugaagcacuguagcucggC..............	1	1	seq
........................................................................agaugaagcacuguagcucggU..............	52	1	seq
........................................................................agaugaagcacuguagcucggUa.............	3	1	seq
.........................................................................gaugaagcacuguagcuG.................	1	1	seq
.........................................................................gaugaagcacuguagcuU.................	2	1	seq
.........................................................................gaugGagcacuguagcuc.................	1	1	seq
.........................................................................gaugaagcacuguagcuc.................	29	0	seq
.........................................................................gaugaagcacuguagcucg................	7	0	seq
.........................................................................gaugaagcacuguagcucU................	40	1	seq
.........................................................................gaugaagcacuguagcucgg...............	4	0	seq
.........................................................................gaugaagcacuguagcucgU...............	8	1	seq
.........................................................................gaugaagcacuguagcucggU..............	46	1	seq
.........................................................................gaugaagcacuguagcucggUa.............	5	1	seq
.........................................................................gaugaagcacuguagcucggga.............	3	0	seq
.........................................................................gaugaagcacuguagcucgggaa............	2	0	seq
.........................................................................gaugaagcacuguagcucggUaa............	2	1	seq
..........................................................................augaagcacuguagcucU................	7	1	seq
..........................................................................augaagcacuguagcucg................	1	0	seq
..........................................................................augaagcacuguagcucgU...............	3	1	seq
..........................................................................augaagcacuguagcucggga.............	1	0	seq
..........................................................................augaagcacuguagcucgggaa............	1	0	seq
..........................................................................augaagcacuguagcucgggaaU...........	1	1	seq
...........................................................................ugaagcacuguagcucgU...............	5	1	seq
...........................................................................ugaagcacuguagcucgg...............	1	0	seq
...........................................................................ugaagcacuguagcucgggaa............	1	0	seq
```

Provisional ID	:	mun-miR-222
Score total	:	1965.1
Score for star read(s)	:	3.9
Score for read counts	:	1955.3
Score for mfe	:	1.3
Score for randfold	:	1.6
Score for cons. seed	:	3
Total read count	:	3847
Mature read count	:	3747
Loop read count	:	0
Star read count	:	100

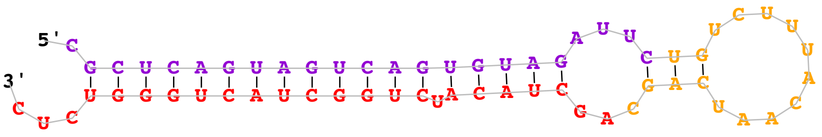

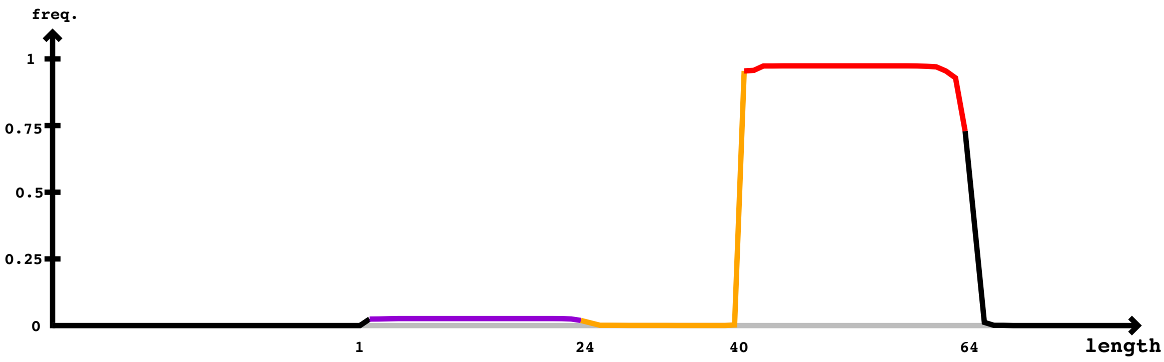

Star Mature

```
5'- gucaacccagaacauguaguugcccaucaaucgcucaguagucaguguagauucugucuuuacaaucagcagcuacaucuggcuacugggucucugaugacaucuccuauc  -3'  obs
    gucaacccagaacauguaguugcccaucaaucgcucaguagucaguguagauucugucuuuacaaucagcagcuacaucuggcuacugggucucugaugacaucuccuauc       exp
    ..((((.((.....))..))))..(((((...((((((((((((((((((...(((..........)))...))))).)))))))))))))...)))))............  reads  mm  sample
    ...............................cgcucaguagucaguguaga............................................................  1      0   seq
    ...............................cgcucaguagucaguguagau...........................................................  4      0   seq
    ...............................Agcucaguagucaguguagau...........................................................  1      1   seq
    ...............................cgcucaguagucaguguagauu..........................................................  21     0   seq
    ...............................cgcucaguagucaguguagaCu..........................................................  1      1   seq
    ...............................cgcucaguagucaguguagauuc.........................................................  53     0   seq
    ...............................cgcucaguagucaUuguagauuc.........................................................  1      1   seq
    ...............................Agcucaguagucaguguagauuc.........................................................  1      1   seq
    ...............................cgcucaguagucaguguagauucu........................................................  10     0   seq
    ...............................cgcucaguagucaguguagauucuguc.....................................................  1      0   seq
    ................................gcucaguagucaguguagauucugucuuuac................................................  1      0   seq
    .................................cucaguagucaguguagauucC........................................................  1      1   seq
    .................................cucaguagucaguguagauucu........................................................  2      0   seq
    ..................................ucaguagucaguguagauucu........................................................  2      0   seq
    ..................................ucaguagucaguguagauucugu......................................................  1      0   seq
    .....................................................cugucuuuacaaucagcagcuacaucuggcuacugggucuc.................  1      0   seq
    .....................................................................cagcuacaucuggcuacuggguc...................  3      0   seq
    .....................................................................cagcuacaucuggcuacugggucu..................  5      0   seq
    ......................................................................agcuacaucuggcuacug.......................  1      0   seq
    ......................................................................agcuacaucuggcuacugg......................  5      0   seq
    ......................................................................agcuacaucuggcuacuggg.....................  8      0   seq
    ......................................................................agcuacaucuggcuacugggA....................  6      1   seq
    ......................................................................agcuacaucuggcuacugggu....................  54     0   seq
    ......................................................................agcuacaucuggcuacuggguU...................  3      1   seq
    ......................................................................Ggcuacaucuggcuacuggguc...................  1      1   seq
    ......................................................................agcuacaucuggcuacuggguc...................  91     0   seq
    ......................................................................agcuacaucuggcuacugggCc...................  1      1   seq
    ......................................................................agcuacaucuggcuacuggguA...................  1      1   seq
    ......................................................................agcuacaucuggcuacugggucG..................  1      1   seq
    ......................................................................agcuacaucuggcuacugggucu..................  724    0   seq
    ......................................................................agcuacaucuggcuacuggguAu..................  2      1   seq
    ......................................................................agcuacaucuggcuacugggCcu..................  1      1   seq
    ......................................................................agcuacGucuggcuacugggucu..................  1      1   seq
```

Star **Mature**

```
gucaacccagaacauguaguugcccaucaaucgcucaguagucaguguagauucugucuuuacaaucagcagcuacaucuggcuacugggucucugaugacaucuccuauc
......................................................................agcuacaucuggcuacugggucA..................  12  1  seq
......................................................................agcuacaucuggcuUcugggucu..................  1  1  seq
......................................................................Cgcuacaucuggcuacugggucu..................  1  1  seq
......................................................................agcuacaucuggcuacugggucC..................  2  1  seq
......................................................................agcuacaucuggcuaAugggucu..................  1  1  seq
......................................................................agcuacaucuggcuacuggguUu..................  4  1  seq
......................................................................agcuacaucuggcuacugggucuc.................  1808  0  seq
......................................................................agcCacaucuggcuacugggucuc.................  1  1  seq
......................................................................agcuacaucuggcuacugggucuA.................  1  1  seq
......................................................................agcuacaucuggcuacugAgucuc.................  1  1  seq
......................................................................agcuacaucuUgcuacugggucuc.................  1  1  seq
......................................................................agcuacauUuggcuacugggucuc.................  1  1  seq
......................................................................Ggcuacaucuggcuacugggucuc.................  2  1  seq
......................................................................agcuacaucuggcuacuggUucuc.................  1  1  seq
......................................................................agAuacaucuggcuacugggucuc.................  1  1  seq
......................................................................agcuacaucuggcuacugggGcuc.................  3  1  seq
......................................................................agcuacauAuggcuacugggucuc.................  1  1  seq
......................................................................agcuacaucuggcuacugggucuU.................  63  1  seq
......................................................................agcuacaucuggcuGcugggucuc.................  1  1  seq
......................................................................agcuacaucuggcuacugggucuUu................  26  1  seq
......................................................................agcuacaucuggcuacugggAcucu................  1  1  seq
......................................................................agcuacaucuggcuacugggucucA................  10  1  seq
......................................................................agcuacaucuggcuacugggucucu................  766  0  seq
......................................................................agcuacaucuggcuacugggGcucu................  4  1  seq
......................................................................agcuaAaucuggcuacugggucucu................  1  1  seq
......................................................................agcuacaucuggUuacugggucucu................  1  1  seq
......................................................................agcuacaucAggcuacugggucucu................  1  1  seq
......................................................................agcuacaucuggcuacugggucGcu................  1  1  seq
......................................................................agcuacaucuggcuacugggucucG................  1  1  seq
......................................................................agcuacaucuggcuacugggucucC................  3  1  seq
......................................................................agcuacaucuggcuaAugggucucu................  1  1  seq
......................................................................agcuacaucuggcuacugggucucuC...............  1  1  seq
......................................................................agcuacaucuggcuacugggucucuA...............  2  1  seq
......................................................................agcuacaucuggcuacugggucucuU...............  38  1  seq
......................................................................agcuacaucuggcuacugggucucuUau.............  3  1  seq
.......................................................................gcuacaucuggcuacugUgucu..................  1  1  seq
.......................................................................gcuacaucuggcuacugggucu..................  4  0  seq
.......................................................................gcuacaucuggcuacugggucuc.................  2  0  seq
.......................................................................gcuacaucuggcuacugggucucu................  1  0  seq
........................................................................cuacaucuggcuacugggucu..................  8  0  seq
........................................................................cuacaucuggcuacugggucuU.................  2  1  seq
........................................................................cuacaucuggcuacugggucuc.................  23  0  seq
........................................................................cuacaucuggcuacugggucucu................  15  0  seq
........................................................................cuacaucuggcuacugggucucC................  7  1  seq
........................................................................cuacaucuggcuacugggucuUu................  4  1  seq
........................................................................cuacaucuggcuacugggucucA................  3  1  seq
........................................................................cuacaucuggcuacugggucucuU...............  2  1  seq
..........................................................................acaucuggcuacugggucucu................  1  0  seq
```

Provisional ID		mun-novel45
Score total	:	28.2
Score for star read(s)	:	3.9
Score for read counts	:	24.1
Score for mfe	:	-0.7
Score for randfold	:	1.6
Score for cons. seed	:	-0.6
Total read count	:	59
Mature read count	:	46
Loop read count	:	6
Star read count	:	7

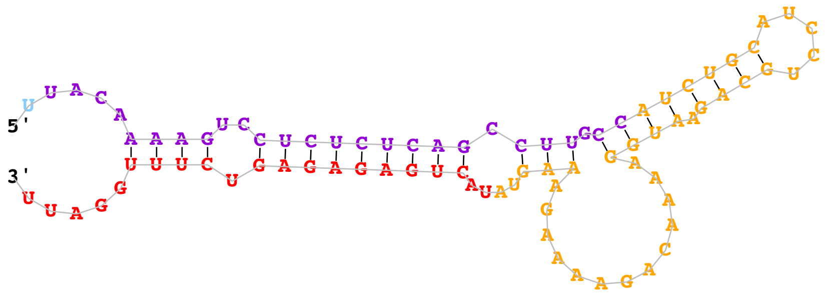

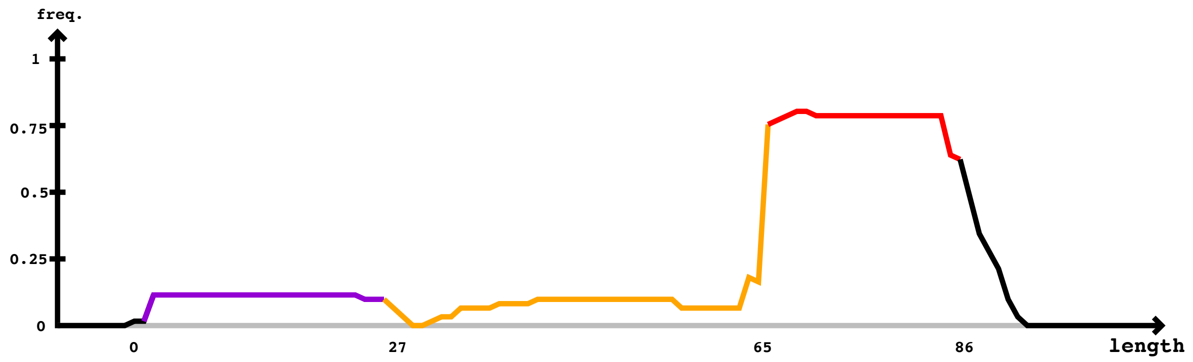

Star **Mature**

```
5'- gaaagaauuacaaaaguccucucucagccuugccaucugcauccugcagaauggaaaacagaaaagaaaguauacugagagagucuuuggauuaggaaagaaaugacagaaag  -3'   obs
    gaaagaauuacaaaaguccucucucagccuugccaucugcauccugcagaauggaaaacagaaaagaaaguauacugagagagucuuuggauuaggaaagaaaugacagaaag         exp
    .....((((...((((..(((((((((.(((.((((((((.....)))).)))).............)))....))))))))).)))).))))....................    reads   mm   sample
    ......auuacaaaaguccucucucagccu...................................................................................    1       0    seq
    ........uacaaaaguccucucucagccuugcc...............................................................................    5       0    seq
    ........uacaaaaguccucucucagcUuugcca..............................................................................    1       1    seq
    .....................................ugcauccugcagaauggaaaacagaa..................................................    1       0    seq
    ......................................gcauccugcagaauggaaaacagaa..................................................    1       0    seq
    ........................................auccugcagaauggaaaacagaaaagaaaguC.........................................    1       1    seq
    ........................................auccugcagaauggaaaacagaaaagaaaguCuac.....................................    1       1    seq
    ............................................ugcagaauggaaaacagaaaagaaagu..........................................    1       0    seq
    ................................................gaauggaaaacagaaaagaaaguauacug....................................    1       0    seq
    ......................................................................uauacugagagagucuAuggauu....................    1       1    seq
    ......................................................................uauacugagagagucuuuggauu....................    3       0    seq
    ......................................................................uauacugagagagucuAuggauuag..................    1       1    seq
    ......................................................................uauacugagagagucuuuggauuagg.................    1       0    seq
    ......................................................................uauacugagagagucuuuggauuagga................    1       0    seq
    ........................................................................uacugagagagucuuugga......................    9       0    seq
    ........................................................................uacugagagagucuuuggauu....................    9       0    seq
    ........................................................................uacugagagagucuuuggauua...................    3       0    seq
    ........................................................................uacugagagagucuuuggauuag..................    3       0    seq
    ........................................................................uacugagagagucuuuggauuagA.................    1       1    seq
    ........................................................................uacugagagagucuuuggauuagg.................    2       0    seq
    ........................................................................uacugagagagCcuuuggauuagga................    1       1    seq
    ........................................................................uacugagagagucuuuggauuagga................    4       0    seq
    ........................................................................uacugagagagucuuuggauuaggaa...............    3       0    seq
    ........................................................................uacugagagagucuuuggauuaggaaa..............    2       0    seq
    .........................................................................acugagagagucuuuggau.....................    1       0    seq
    ..........................................................................cugagagagucuuuggauu....................    1       0    seq
    ...........................................................................ugagagagucuuuggauuagga................    1       0    seq
    ...........................................................................ugagagagucuuuggauuaggaa...............    1       0    seq
```

Provisional ID	:	mun-miR-17a
Score total	:	2267.3
Score for star read(s)	:	3.9
Score for read counts	:	2257.1
Score for mfe	:	1.7
Score for randfold	:	1.6
Score for cons. seed	:	3
Total read count	:	4439
Mature read count	:	3680
Loop read count	:	0
Star read count	:	759

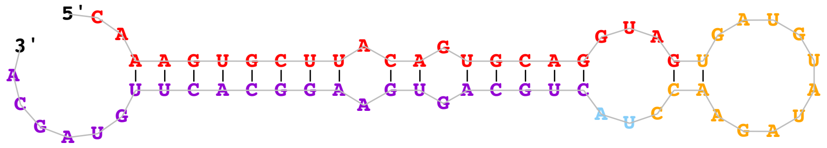

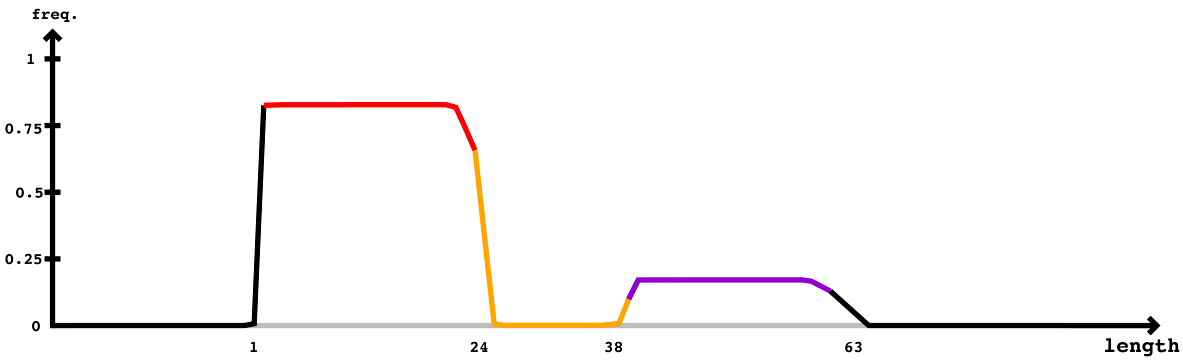

Mature Star

```
5'- ugguuucgucagaguaaugucaaagugcuuacagugcagguagugauguauagaaccuacugcagugaaggcacuuguagcauuauguugacaacugccucaggagaucuugc -3'  obs
    ugguuucgucagaguaaugucaaagugcuuacagugcagguagugauguauagaaccuacugcagugaaggcacuuguagcauuauguugacaacugccucaggagaucuugc       exp
    .(((...(((((.(((((((..((((((((.((.(((((...((..........))...))))).)).))))))))...))))))).)))))....))).((((....)))).  reads  mm  sample
    ugguuucgucagaguaaugu.............................................................................................  1  0  seq
    ...................ucaaagugcuuacagugcag..........................................................................  1  0  seq
    ...................ucaaagugcuuacagugcagg.........................................................................  1  0  seq
    ...................ucaaagugcuuacagugcaggu........................................................................  11  0  seq
    ...................ucaaagugcuuacagugcagguU.......................................................................  2  1  seq
    ...................ucaaagugcuuacagugcaggua.......................................................................  7  0  seq
    ...................ucaaagugcuuacagugcagguag......................................................................  2  0  seq
    ...................ucaaagugcuuacagugcagguaU......................................................................  5  1  seq
    ...................ucaaagugcuuacagugcagguaUu.....................................................................  1  1  seq
    ....................caaagugcuuacagugcag..........................................................................  1  0  seq
    ....................caaagugcuuacagugcagU.........................................................................  2  1  seq
    ....................caaagugcuuacagugcagg.........................................................................  39  0  seq
    ....................caaagugcuuacagugcaggu........................................................................  329  0  seq
    ....................caaagugcuuaUagugcaggu........................................................................  1  1  seq
    ....................caaagugcuuacagugUaggu........................................................................  1  1  seq
    ....................caaagugcuuacagugcaggA........................................................................  6  1  seq
    ....................caaagugcuuacagugcaggAa.......................................................................  1  1  seq
    ....................caaagugcuuacagugcagguU.......................................................................  31  1  seq
    ....................caaagugcuuacagugcagguC.......................................................................  2  1  seq
    ....................caaagugcuuacagugcagguG.......................................................................  6  1  seq
    ....................caaagugcuuacagugcaggCa.......................................................................  3  1  seq
    ....................caUagugcuuacagugcaggua.......................................................................  1  1  seq
    ....................caaagugcuuacagCgcaggua.......................................................................  1  1  seq
    ....................caaagugcuuacagugcaggua.......................................................................  317  0  seq
    ....................caaagugcuuacagugcagguGg......................................................................  1  1  seq
    ....................caaagGgcuuacagugcagguag......................................................................  1  1  seq
    ....................caaagugAuuacagugcagguag......................................................................  1  1  seq
    ....................caaaguAcuuacagugcagguag......................................................................  1  1  seq
    ....................caaagugcuuacagugcaAguag......................................................................  1  1  seq
    ....................caaagAgcuuacagugcagguag......................................................................  1  1  seq
    ....................caaagugcuuacaUugcagguag......................................................................  1  1  seq
    ....................caaagugcuuacagugcaggGag......................................................................  4  1  seq
    ....................caaagugcuuacagugAagguag......................................................................  1  1  seq
```

Mature **Star**

```
ugguuucgucagaguaaugucaaagugcuuacagugcagguagugauguauagaaccuacugcagugaaggcacuuguagcauuauguugacaacugccucaggagaucuugc
....................caaagugcuuacaguUcagguag......................................................................   2      1   seq
....................caaagugcuuacagugcagguag......................................................................   1652   0   seq
....................caaagugcuuacagugcaggCag......................................................................   1      1   seq
....................caaagugcuuacagugcagguaC......................................................................   16     1   seq
....................caaagugcuuacagugcagguaU......................................................................   221    1   seq
....................caaagugcuuacagGgcagguag......................................................................   1      1   seq
....................caaagugcuuacagugcagguUg......................................................................   5      1   seq
....................Uaaagugcuuacagugcagguag......................................................................   1      1   seq
....................caaagugcuuacagugcagUuag......................................................................   1      1   seq
....................Gaaagugcuuacagugcagguag......................................................................   1      1   seq
....................caaagugcuuacagugcagguaA......................................................................   73     1   seq
....................caaagugcuuacagugcagguaAu.....................................................................   3      1   seq
....................caaagugcuuacagugcagguagA.....................................................................   63     1   seq
....................caaagugcuuacaUugcagguagu.....................................................................   1      1   seq
....................Aaaagugcuuacagugcagguagu.....................................................................   82     1   seq
....................caaagGgcuuacagugcagguagu.....................................................................   1      1   seq
....................caaagugcuuacagugcagguagG.....................................................................   1      1   seq
....................caaagugcuuacagugcagguaUu.....................................................................   29     1   seq
....................caaagugcuuacagugcaggCagu.....................................................................   1      1   seq
....................caaagugcuuacagugcGgguagu.....................................................................   1      1   seq
....................caaagugcuuacagugcagguaCu.....................................................................   1      1   seq
....................caaagugcuuacagugcagguagu.....................................................................   711    0   seq
....................caaagugcuuacagugcagguagug....................................................................   1      0   seq
....................caaagugcuuacagugcagguaguU....................................................................   19     1   seq
.....................aaagugcuuacagugcaggua.......................................................................   1      0   seq
.....................aaagugcuuacagugcagguag......................................................................   4      0   seq
......................aagugcuuacagugcagguaU......................................................................   1      1   seq
......................aagugcuuacagugcagguaUu.....................................................................   1      1   seq
......................aagugcuuacagugcagguagu.....................................................................   2      0   seq
.............................uacagugcagguagugauguauagaaccu.......................................................   2      0   seq
..........................................gugauguauagaaccuacugcagugaaggcacuuguagc................................   1      0   seq
...........................................ugauguauagaaccuacugcagugaaggcacuuguagcU...............................   1      1   seq
........................................................cuacugcagugaaggcacuug....................................   1      0   seq
........................................................cuacugcagugaaggcacuugu...................................   8      0   seq
........................................................cuacugcagugaaggcacuuguU..................................   1      1   seq
........................................................cuacugcagugaaggcacuugua..................................   1      0   seq
.........................................................uacugcagugaaggcacuugu...................................   4      0   seq
.........................................................uacugcagugaaggcacuugua..................................   14     0   seq
.........................................................uacugcagugaaggcacuuguU..................................   1      1   seq
.........................................................uacugcagugaaggcacuuguag.................................   2      0   seq
.........................................................uacugcagugaaggcacuuguagU................................   1      1   seq
.........................................................uacugcagugaaggcacuuguagcU...............................   1      1   seq
..........................................................acugcagugaaggcacuug....................................   17     0   seq
..........................................................acugcagugaCggcacuug....................................   1      1   seq
..........................................................acugcagugaagCcacuug....................................   1      1   seq
..........................................................acugcagugaaggcacuugu...................................   65     0   seq
..........................................................acugcagugaaggcacuugC...................................   1      1   seq
..........................................................acugcagugaaggcacuuguU..................................   4      1   seq
..........................................................acugcagugaaggcacuugua..................................   57     0   seq
..........................................................acugcagugaaggcacuugCa..................................   1      1   seq
..........................................................Gcugcagugaaggcacuuguag.................................   2      1   seq
..........................................................acugcagugaaggcacuuguaA.................................   5      1   seq
..........................................................acugcagugaaggcacuuguaU.................................   3      1   seq
..........................................................acugcagugaaggcacuuguag.................................   39     0   seq
..........................................................acugcagugaaggcacuuguagU................................   37     1   seq
..........................................................acugcagugaaggcacuuguagc................................   56     0   seq
..........................................................acugcagugaaggcacuuguagA................................   4      1   seq
..........................................................acugcagugaaggcacuuguagcU...............................   108    1   seq
..........................................................acugcagugaaggcacuuguagAa...............................   1      1   seq
..........................................................acugcagugaaggcacuuguagcUu..............................   3      1   seq
...........................................................cugcagugaaggcacuugu...................................   4      0   seq
...........................................................cugcagugaaggcacuugua..................................   4      0   seq
...........................................................cugcagugaaggcacuuguag.................................   10     0   seq
...........................................................cugcagugaaggcacuuguaU.................................   1      1   seq
...........................................................cugcagugaaggcacuuguagc................................   80     0   seq
...........................................................cugcagugaaggcacuuguagU................................   16     1   seq
...........................................................cugcagugaaggcacuuguagA................................   2      1   seq
...........................................................cugcagugaaggcacuuguaCc................................   1      1   seq
...........................................................cugcagugaaggcacuuguagca...............................   9      0   seq
...........................................................cugcagugaaggcacuuguagcG...............................   1      1   seq
```

Mature **Star**

```
ugguuucgucagaguaaugucaaagugcuuacagugcagguagugauguauagaaccuacugcagugaaggcacuuguagcauuauguugacaacugccucaggagaucuugc
...........................................................cugcagugaaggcacuuguagcU...............................	185	1	seq
...........................................................cugcagugaaggcacuuguagcUu..............................	5	1	seq
............................................................ugcagugaaggcacuuguagc................................	1	0	seq
................................................................gugaaggcacuuguagcU...............................	1	1	seq
.................................................................................auuauguugacaacugccucagg.........	1	0	seq
```

Provisional ID	:	mun-miR-1b
Score total	:	23.1
Score for star read(s)	:	3.9
Score for read counts	:	12.9
Score for mfe	:	1.7
Score for randfold	:	1.6
Score for cons. seed	:	3
Total read count	:	37
Mature read count	:	33
Loop read count	:	0
Star read count	:	4

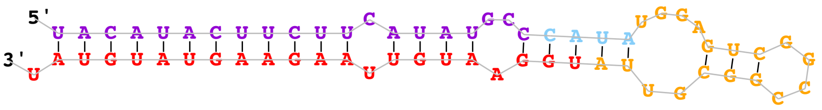

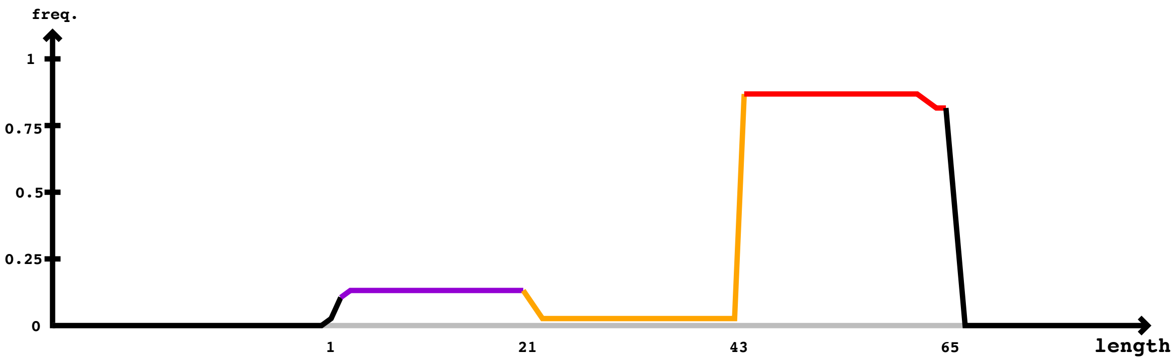

Star Mature

```
5'- cacagugcucaccucucccucccaacccuacauacuucuucauaugcccauauggagucggccggcguuauggaauguuaagaaguauguaucuuugggcugggacccccgc  -3'  obs
    cacagugcucaccucucccucccaacccuacauacuucuucauaugcccauauggagucggccggcguuauggaauguuaagaaguauguaucuuugggcugggacccccgc       exp
    ...............((((.(((((...((((((((((((.((((..(((((....(((....)))..))))).)))).))))))))))))...)))))..)))).......   reads   mm   sample
    ...........................cuacauacuucuucaCaugcc................................................................   1       1    seq
    ............................uacauacuucuucauaugcc................................................................   2       0    seq
    ............................uacauacuucuucauaugccc...............................................................   1       0    seq
    .............................acauacuucuucauaugcccauauggagucggccggcguua..........................................   1       0    seq
    ......................................................................uggaauguuaagaaguaug.......................   1       0    seq
    ......................................................................uggaauguuaagaaguaugu......................   1       0    seq
    ......................................................................uggaauguuaagaaguauguau....................   23      0    seq
    ......................................................................uggaauguuaagaaguauguaA....................   2       1    seq
    ......................................................................uggaauguuaagaaguauguaCc...................   1       1    seq
    ......................................................................uggaauguuaagaaguauguauU...................   5       1    seq
```

Provisional ID	:	mun-miR-1677
Score total	:	53.4
Score for star read(s)	:	3.9
Score for read counts	:	43
Score for mfe	:	1.9
Score for randfold	:	1.6
Score for cons. seed	:	3
Total read count	:	96
Mature read count	:	85
Loop read count	:	0
Star read count	:	11

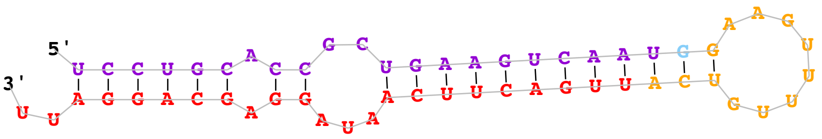

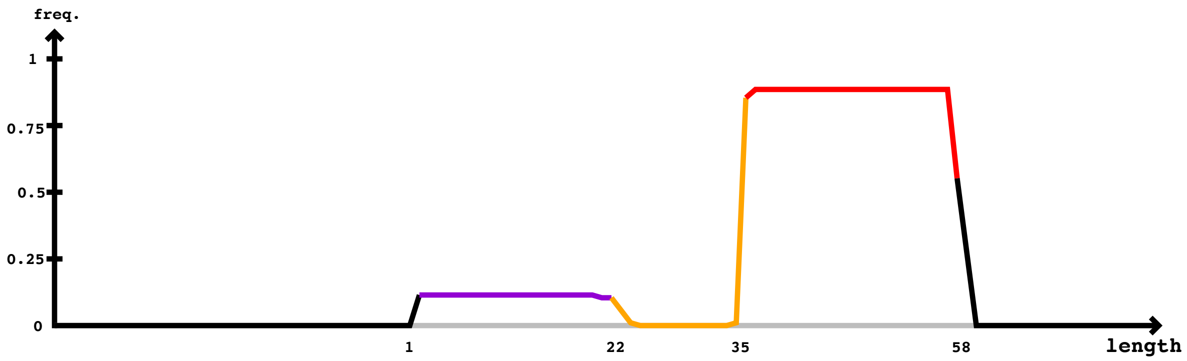


```
                                     Star                                  Mature
5'- agaaaacagauguuagcuguguugcucagggucugauccugcaccgcugaagucaauggaaguuuugucauugacuucaauaggagcaggauuugaaccuuaugcucuaucag   -3'   obs
    agaaaacagauguuagcuguguugcucagggucugauccugcaccgcugaagucaauggaaguuuugucauugacuucaauaggagcaggauuugaaccuuaugcucuaucag         exp
    .((..((((.......))))...((..((((((.((((((((.((..((((((((((((........))))))))))))...)).)))))))).).)))))..))....))..   reads   mm   sample
    ....................................uccugcaccgcugaaguca..........................................................   1       0    seq
    ....................................uccugcaccgcugaagucaau........................................................   5       0    seq
    ....................................uccugcaccgcugaagucaaug.......................................................   3       0    seq
    ....................................uccugcaccgcugaagucaauU.......................................................   1       1    seq
    ....................................uccugcaccgcugaagucaaugA......................................................   1       1    seq
    .....................................................................auugacuucaauaggagcaggau.....................   1       0    seq
    ......................................................................uugacuucaauaggagcaggau.....................   30      0    seq
    ......................................................................Augacuucaauaggagcaggau.....................   1       1    seq
    ......................................................................uugacuucaauaggagcaggauu....................   47      0    seq
    ......................................................................uugacuucaauaggagcaggaCu....................   2       1    seq
    ......................................................................uugacuucaauaggagcaggauuu...................   1       0    seq
    .......................................................................ugacuucaauaggagcaggauu....................   3       0    seq
```

Provisional ID : mun-novel34
Score total : 0
Score for star read(s) : -1.3
Score for read counts : -1.3
Score for mfe : 1.7
Score for randfold : 1.6
Score for cons. seed : -0.6
Total read count : 9
Mature read count : 9
Loop read count : 0
Star read count : 0

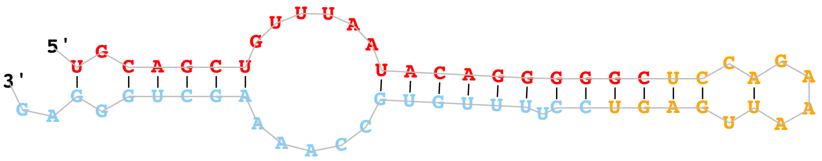

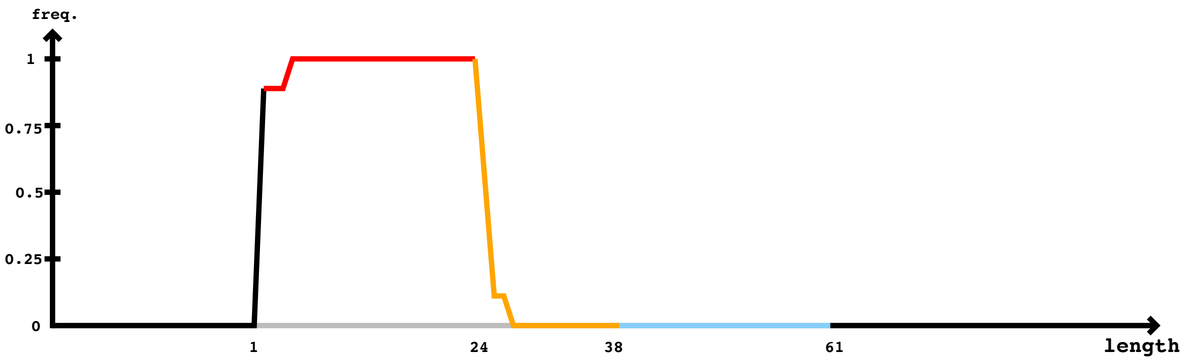

Mature Star

```
5'- auuaguagguguacaguaccugcagcuguuuaauacagggggcuccagaaauugaguccuuuugugccaaaagcugggagcugugauggcuaagucaggagagcggccugagg   -3'    exp
    .(((((.....((((((.((..(((((......((((((((((((.(....).)))))).)))))).....))))))).))))))...))))).(((((.......)))))..    reads    mm    sample
    ....................ugcagcuguuuaauacagggggc......................................................................    6        0     seq
    ....................ugcagcuguuuaauacagggggcu.....................................................................    2        0     seq
    .......................agcuguuuaauacagggggcucc...................................................................    1        0     seq
```

Provisional ID	:	mun-miR-155
Score total	:	4.9
Score for star read(s)	:	-1.3
Score for read counts	:	0
Score for mfe	:	1.6
Score for randfold	:	1.6
Score for cons. seed	:	3
Total read count	:	764
Mature read count	:	764
Loop read count	:	0
Star read count	:	0

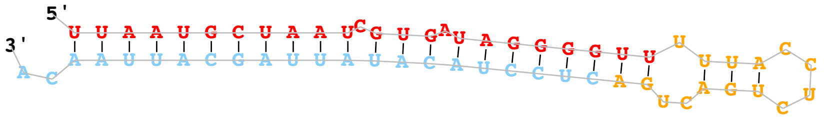

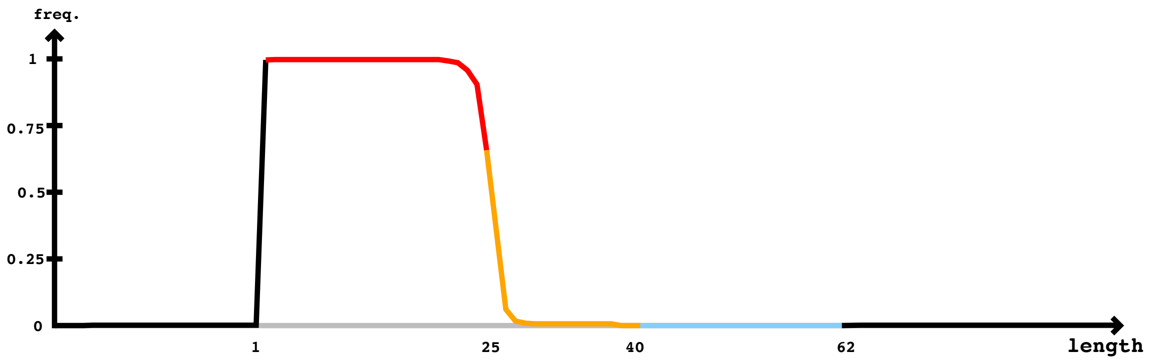

Mature Star

```
5'- cuucuguaggcuguauguuguuaaugcuaaucgugauagggguuuuuaccucugacugacuccuacauauuagcauuaacacuguaucaugccucuuaccagaauucac   -3'   exp
    .((((.((((((((..((((((((((((.(((.((((((((.(((....)))..)))))))))))))))))))))))..))).)).)))).....))))).....   reads   mm   sample
    ..ucuguaggcuguauguug.........................................................................................   1   0   seq
    ....................uuaaugcuaaucgugauag......................................................................   4   0   seq
    ....................uuaaugcuaaucgugauagU.....................................................................   1   1   seq
    ....................uuaaugcuaaucgugauagg.....................................................................   4   0   seq
    ....................uuaaugcuaaucgugauaggA....................................................................   1   1   seq
    ....................uuaaugcuaaucgugauaggU....................................................................   5   1   seq
    ....................uuaaugcuaaucgugauaggg....................................................................   16   0   seq
    ....................uuaaugcuaaucgugauagggg...................................................................   28   0   seq
    ....................uuaaugcuaaucgugauagggA...................................................................   6   1   seq
    ....................uuaaugcuaaucgugauagggU...................................................................   6   1   seq
    ....................uuaaCgcuaaucgugauagggg...................................................................   1   1   seq
    ....................uuaaugcuaaucgugauaggggu..................................................................   167   0   seq
    ....................uuaaugcuaaucCugauaggggu..................................................................   1   1   seq
    ....................uuaaugcuaaucgugauagggUu..................................................................   2   1   seq
    ....................uuaaugcuaaucgugauaggggG..................................................................   3   1   seq
    ....................uuaaugcuaaucgugauagggAu..................................................................   9   1   seq
    ....................uuaaugcuaaucgugauaggggA..................................................................   8   1   seq
    ....................uuaauAcuaaucgugauagggguu.................................................................   1   1   seq
    ....................uuaaugcuaaucgugauaggggCu.................................................................   1   1   seq
    ....................uuaaugcuaaucgugauagggguu.................................................................   358   0   seq
    ....................uuaaugcuaaucgugauagggguC.................................................................   1   1   seq
    ....................uuaaugcuaaucgugauagggguG.................................................................   3   1   seq
    ....................uuaaugcuaaucgugauaggggGu.................................................................   2   1   seq
    ....................uuaaugcuaaucgugauagggguA.................................................................   5   1   seq
    ....................Guaaugcuaaucgugauagggguu.................................................................   1   1   seq
    ....................uuaaugcuaaucgugauaggggAu.................................................................   15   1   seq
    ....................uuaaugcuaaucgugauagggAuu.................................................................   1   1   seq
    ....................uuaaugcuaaucgugauagggguuC................................................................   1   1   seq
    ....................uuaaugcuaaucgugauagggguAu................................................................   1   1   seq
    ....................uuaaugcuaaucgugauagggguuA................................................................   10   1   seq
    ....................uuaaugcuaaucgugauagggguuu................................................................   57   0   seq
    ....................uuaaugcuaaucgugauaggggGuu................................................................   1   1   seq
    ....................uuGaugcuaaucgugauagggguuu................................................................   1   1   seq
    ....................uuaaugcuaaucgugauagggguuAu...............................................................   3   1   seq
```

```
                    Mature                                   Star
cuucuguaggcuguauguuguuaaugcuaaucgugauagggguuuuuaccucugacugacuccuacauauuagcauuaacacuguaucaugccucuuaccagaauucac
....................uuaaugcuaaucgugauagggguAuu...............................................................    1     1     seq
....................uuaaugcuaaucgugauagggguuuu...............................................................    29    0     seq
....................uuaaugcuaaucgugauagggguuuA...............................................................    1     1     seq
....................uuaaugcuaaucgugauagggguuuuu..............................................................    3     0     seq
....................uuaaugcuaaucgugauagggguuuuA..............................................................    1     1     seq
....................uuaaugcuaaucgugauagggguuuCu..............................................................    1     1     seq
....................uuaaugcuaaucgugauagggguuuAu..............................................................    1     1     seq
....................uuaaugcuaaucgugauagggguuuuuU.............................................................    2     1     seq
....................uuaaugcuaaucgugauagggguuuuuaccucugacu....................................................    5     0     seq
.....................uaaugcuaaucgugauagggguu.................................................................    1     0     seq
.................................................................................cuguaucaugccucuuaccagaauucac    1     0     seq
```

Provisional ID		mun-novel95
Score total	:	7.4
Score for star read(s)	:	3.9
Score for read counts	:	0.6
Score for mfe	:	1.8
Score for randfold	:	1.6
Score for cons. seed	:	-0.6
Total read count	:	13
Mature read count	:	7
Loop read count	:	0
Star read count	:	6

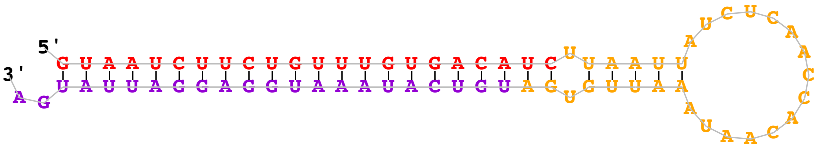

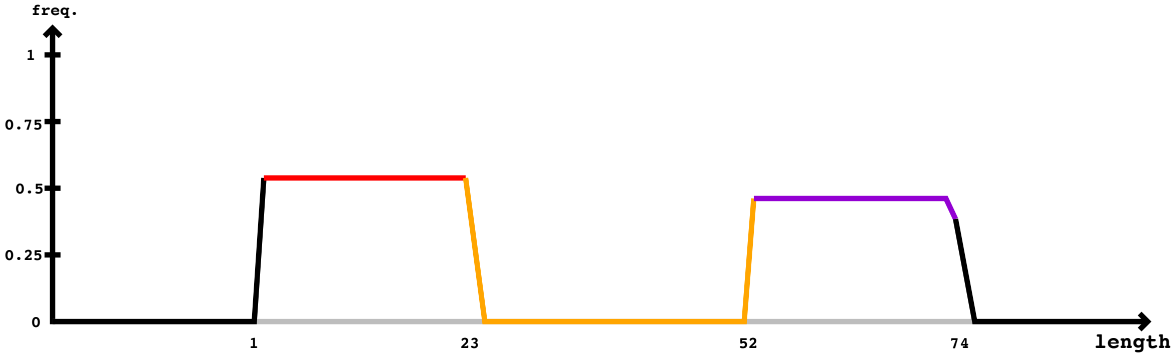


```
                    Mature                                                   Star
5'- cuaauugccuaccugaauuuguaaucuucuguuugugacaucuuaauuaucucaaccacaauaaauugugaugucauaaauggaggauuaugacuuggguuggaaaugcaa   -3'    obs
    cuaauugccuaccugaauuuguaaucuucuguuugugacaucuuaauuaucucaaccacaauaaauugugaugucauaaauggaggauuaugacuuggguuggaaaugcaa          exp
    ...(((.((.(((..((.(..((((((((((((((((((((.(((((...............))))).))))))))))))))))))))..).))..))).)).)))....    reads    mm    sample
    ....................guaaucuucuguuugugacauc.....................................................................    7        0     seq
    .......................................................................ugucauaaauggaggauuaug...................    1        0     seq
    .......................................................................ugucauaaauggaggauuauga..................    5        0     seq
```

Provisional ID : mun-miR-460b
Score total : 105.6
Score for star read(s) : 3.9
Score for read counts : 95
Score for mfe : 2.1
Score for randfold : 1.6
Score for cons. seed : 3
Total read count : 198
Mature read count : 132
Loop read count : 0
Star read count : 66

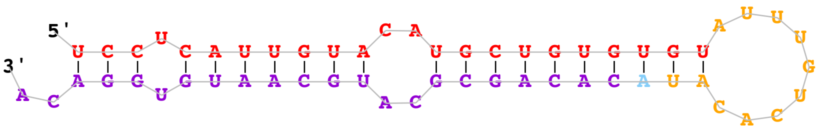

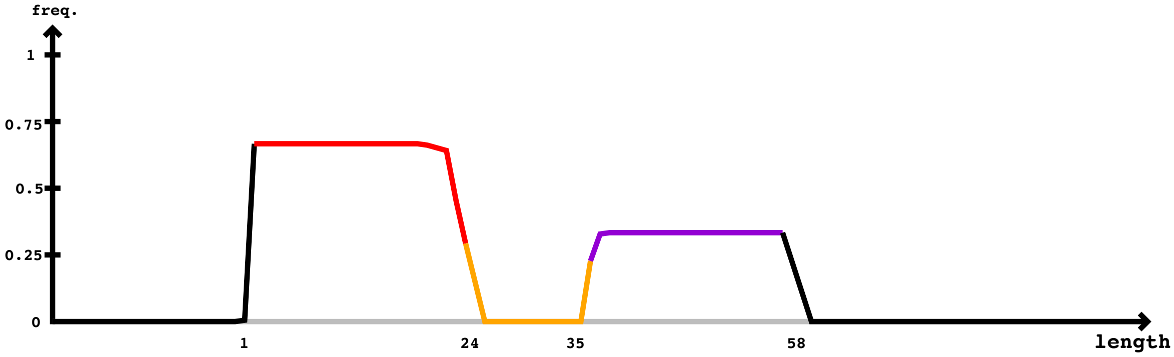


```
                     Mature                                   Star
5'- cucuucuggcucuauaguguccucauuguacaugcugugugugauuugucacauacacagcgcaugcaauguggacauaauggagcucaacuuacgccugaggagauaaaauc  -3'   obs
    cucuucuggcucuauaguguccucauuguacaugcugugugugauuugucacauacacagcgcaugcaauguggacauaauggagcucaacuuacgccugaggagauaaaauc        exp
    (((((.((((((((.((((((.(((((((..((((((((((.........))))))))))..))))))).)))))).))))))))...........)))))........     reads   mm   sample
    ..................Auccucauuguacaugcug...........................................................................     1       1    seq
    ...................uccucauuguacaugcugu..........................................................................     2       0    seq
    ...................uccucauuguacaugcugug.........................................................................     2       0    seq
    ...................uccucauuguacaugcugugA........................................................................     1       1    seq
    ...................uccucauuguacaugcugugG........................................................................     4       1    seq
    ...................uccucauuguacaugcugugu........................................................................     32      0    seq
    ...................uccucauuguacaugcuguguU.......................................................................     1       1    seq
    ...................uccucauuguacaugcugugug.......................................................................     31      0    seq
    ...................uccucauuguacaugcugugugG......................................................................     6       1    seq
    ...................uccucauuguacaugcugugugA......................................................................     45      1    seq
    ...................uccucauuguacaugcugugugu......................................................................     6       0    seq
    ...................uccucauuguacaugcugugugAa.....................................................................     1       1    seq
    ......................................................cacagcgcGugcaauguggac.....................................     1       1    seq
    ......................................................cacagcgcaugcaauguggac.....................................     13      0    seq
    ......................................................cacagcgcaugcaauCuggac.....................................     1       1    seq
    ......................................................cacagcgcaugcaauguggacU....................................     22      1    seq
    ......................................................cacagcgcaugcaauguggaca....................................     6       0    seq
    ......................................................cacagcgcaugcaauguggacC....................................     2       1    seq
    .......................................................acagcgcaugcaauguggac.....................................     5       0    seq
    .......................................................acagcgcaugcaauguggaca....................................     2       0    seq
    .......................................................acagcgcaugcaauguggacU....................................     10      1    seq
    .......................................................acagcgcaugcaauguggacC....................................     1       1    seq
    .......................................................acagcgcauUcaauguggaca....................................     1       1    seq
    .......................................................acagcgcaugcaauguggacau...................................     1       0    seq
    ........................................................cagcgcaugcaauguggac.....................................     1       0    seq
```

Provisional ID	:	mun-novel57
Score total	:	4.2
Score for star read(s)	:	-1.3
Score for read counts	:	0
Score for mfe	:	0.9
Score for randfold	:	1.6
Score for cons. seed	:	3
Total read count	:	253
Mature read count	:	251
Loop read count	:	0
Star read count	:	2

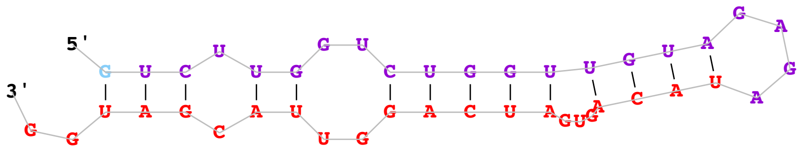

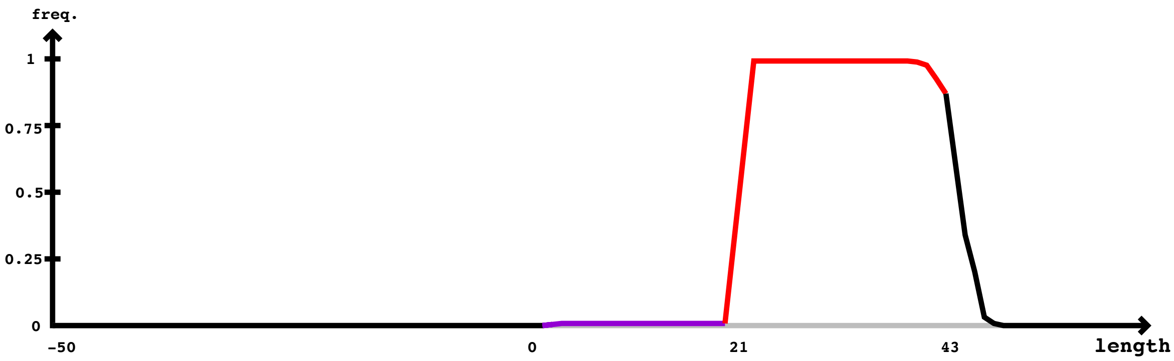

Star Mature

```
5'- ugcuguguuguuacaagcaugcaucuguguguacuagugcuagagaaaagucuuggucugguuguagagauacagugaucagguuacgauggauuucucaaguaacaaccuc  -3'   obs
    ugcuguguuguuacaagcaugcaucuguguguacuagugcuagagaaaagucuuggucugguuguagagauacagugaucagguuacgauggauuucucaaguaacaaccuc         exp
    ......((((((((..((((((....))))))..........((((((.(((.((..(((((((((....))))...)))))..)).)))...))))))..))))))))...  reads    mm       sample
    ..................................................ucuuggucugguuguagaga..........................................  1        0        seq
    ...................................................cuuggucugguuguagaga..........................................  1        0        seq
    ......................................................................uacagugaucagguuacg........................  1        0        seq
    ......................................................................uacagugaucagguuacga.......................  2        0        seq
    ......................................................................uacagugaucagguuacgau......................  13       0        seq
    ......................................................................uacagugaucagguuacgauA.....................  2        1        seq
    ......................................................................uacagugaucaggCuacgaug.....................  1        1        seq
    ......................................................................uacagugaucagguuacgauU.....................  1        1        seq
    ......................................................................uacagugaucagguuacgaug.....................  10       0        seq
    ......................................................................uacagugaucagguuacgaugg....................  59       0        seq
    ......................................................................uacagugaucagguuacgaugU....................  5        1        seq
    ......................................................................uacagugaucagguuacgauggU...................  28       1        seq
    ......................................................................uacagugaucagguuacgaugga...................  38       0        seq
    ......................................................................uacagugaucagguuacgauggC...................  1        1        seq
    ......................................................................uacagugaucagguuacgauggUu..................  2        1        seq
    ......................................................................uacagugaucagguuacgauggaA..................  4        1        seq
    ......................................................................uacagugaucagguuacgauggau..................  14       0        seq
    ......................................................................uacagugaucagguuacgauggaAu.................  1        1        seq
    ......................................................................uacagugaucagguuacgauggauu.................  17       0        seq
    ......................................................................uacagugaucagguuacgauggUuu.................  1        1        seq
    ......................................................................uacagugaucagguuacgauggauuu................  3        0        seq
    ......................................................................uacagugaucagguuacgauggauuuc...............  2        0        seq
    .......................................................................acagugaucagguuacga.......................  1        0        seq
    .......................................................................acagugaucagguuacgaugga...................  3        0        seq
    .......................................................................acagugaucagguuacgauggau..................  14       0        seq
    .......................................................................acagugaucagguuacgauggaA..................  1        1        seq
    .......................................................................acagugaucagguuacgauggauu.................  23       0        seq
    .......................................................................acagugaucagguuacgauggauA.................  1        1        seq
    .......................................................................acagugaucagguuacgauggauuu................  3        0        seq
```

Provisional ID	:	mun-novel79
Score total	:	1.4
Score for star read(s)	:	-1.3
Score for read counts	:	0
Score for mfe	:	1.7
Score for randfold	:	1.6
Score for cons. seed	:	-0.6
Total read count	:	92
Mature read count	:	91
Loop read count	:	1
Star read count	:	0

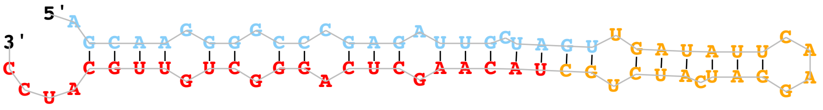

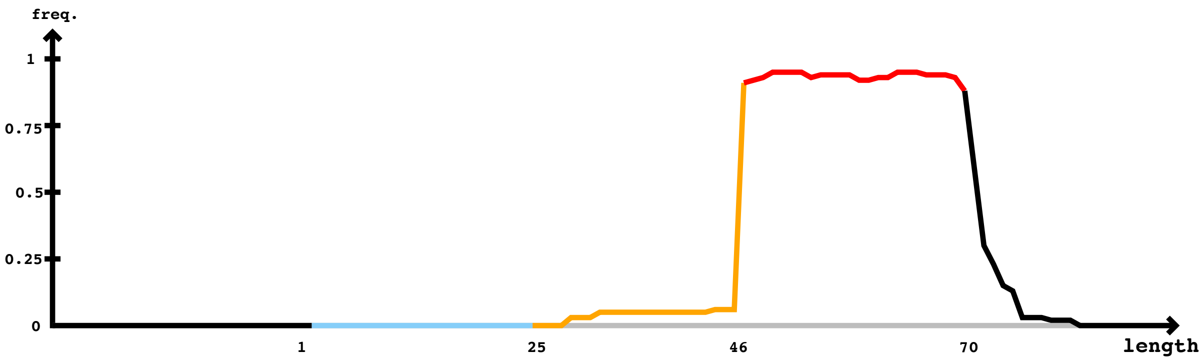

Star Mature

```
5'- gguuccaugugauauagcccuagggagcaaggggcccgagauugcuaguugauauucaaggaucaucugcuacaagcucagggcuguugcaucccgacuagaaggaaaugggcaa  -3'   exp
    ................((((..((((((((.((.((.(((.(((.((((.((((((....))).))).))))))).))).)).)).)))).))))..((....))....))))..   reads   mm   sample
    ....................................................uauucaaggaucaGcugcu............................................   1       1    seq
    ....................................................uauucaaggaucaGcugcuacaagc......................................   2       1    seq
    .......................................................ucaaggaucaucugcuacaagcucagA.................................   1       1    seq
    .......................................................ucaaggaucaucugcuacaagcucagg.................................   1       0    seq
    ...................................................................ugcuacaagcucaggAcuguug..........................   1       1    seq
    ......................................................................uacaagcucaggAcuguugcau.......................   1       1    seq
    ......................................................................uacaagcucaggAcuguugcauc......................   4       1    seq
    ......................................................................uacaagcucagggcuguugcauc......................   1       0    seq
    ......................................................................uacaagcucagggcuguugcaucc.....................   1       0    seq
    ......................................................................uacaagcucaggAcuguugcaucc.....................   40      1    seq
    ......................................................................uacaagcucagggGuguugcaucc.....................   1       1    seq
    ......................................................................uacaagcucagggcuguugcauccc....................   5       0    seq
    ......................................................................uacaagcucaggAcuguugcauccc....................   8       1    seq
    ......................................................................uacaagcucagggcuguugcaucccg...................   3       0    seq
    ......................................................................uacaagcucaggAcuguugcaucccg...................   3       1    seq
    ......................................................................uacaagcucagggcuguugcaucccga..................   4       0    seq
    ......................................................................uacaagcucaggAcuguugcaucccga..................   2       1    seq
    ......................................................................uacaagcucagggcuguugcaucccgG..................   1       1    seq
    ......................................................................uacaagcucaggAcuguugcaucccgac.................   1       1    seq
    ......................................................................uacaagcucagggcuguugcaucccgacu................   9       0    seq
    ......................................................................uacaagcucagggcuguugcaucccgacA................   1       1    seq
    .......................................................................acaagcucaggAcuguugcaucc.....................   1       1    seq
    .......................................................................acaagcucaggAcuguugcauccc....................   1       1    seq
    ........................................................................caagcucagggcuguugcaucccgac.................   1       0    seq
    .........................................................................aagcucaggAcuguugcaucc.....................   1       1    seq
    .........................................................................aagcucaggAcuguugcaucccga..................   1       1    seq
    ..............................................................................caggAcuguugcaucccg...................   1       1    seq
    ....................................................................................uguugcauccGgacuaga.............   1       1    seq
    ......................................................................................uugcaucccgacuagaagg..........   1       0    seq
    ......................................................................................uugcaucccgUcuagaagg..........   1       1    seq
```

Provisional ID	:	mun-novel30
Score total	:	34507.8
Score for star read(s)	:	3.9
Score for read counts	:	34500.5
Score for mfe	:	2.4
Score for randfold	:	1.6
Score for cons. seed	:	-0.6
Total read count	:	67683
Mature read count	:	67633
Loop read count	:	0
Star read count	:	50

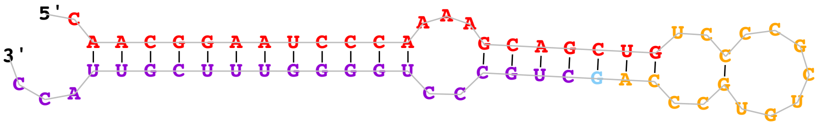

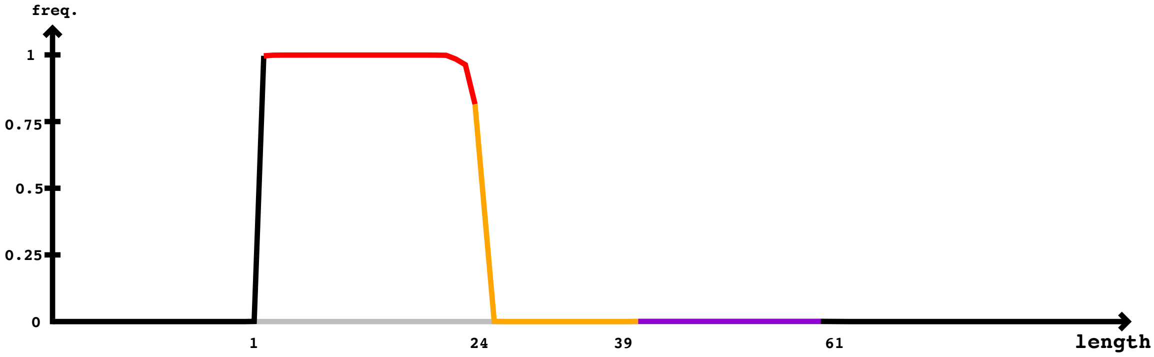

Mature Star

```
5'- cuuuguggugccggcgcgggcaacggaaucccaaaagcagcuguccccgcugugcccagcugcccugggguuucguuacccgcgcugccgccccugccucgccccacagc  -3'  obs
    cuuuguggugccggcgcgggcaacggaaucccaaaagcagcuguccccgcugugcccagcugcccugggguuucguuacccgcgcugccgccccugccucgccccacagc       exp
    ..((((((.(((((((((((.((((((((((((...(((((((..(.......)..)))))))..)))))))))))).)))))))))..((....))...)).)))))).  reads  mm  sample
    ...................gcaacggaaucccaaaagcag......................................................................  5     0   seq
    ...................gcaacggaaucccaaaagcagc.....................................................................  9     0   seq
    ...................gcaacggaaucccaaaagcagcu....................................................................  16    0   seq
    ...................gcaacggaaucccaaaagcagcuC...................................................................  1     1   seq
    ...................gcaacggaaucccaaaagcagcuU...................................................................  2     1   seq
    ...................gcaacggaaucccaaaagcagcug...................................................................  2     0   seq
    ...................Ccaacggaaucccaaaagcagcug...................................................................  4     1   seq
    ....................caacggaaucccaaaagc........................................................................  11    0   seq
    ....................caacggaaucccaaaagcU.......................................................................  2     1   seq
    ....................caacggaaucccaaaaAca.......................................................................  1     1   seq
    ....................caacggaaucccaaaagUa.......................................................................  1     1   seq
    ....................caacggaaucccaaaagca.......................................................................  43    0   seq
    ....................Uaacggaaucccaaaagcag......................................................................  3     1   seq
    ....................caacggaauUccaaaagcag......................................................................  1     1   seq
    ....................caacggaaucccaaaagcag......................................................................  898   0   seq
    ....................caacggaaucccaaaagUag......................................................................  17    1   seq
    ....................caacggaaucccaaaagcaC......................................................................  1     1   seq
    ....................caacggaaucccaaaagcaA......................................................................  19    1   seq
    ....................caacggaaucUcaaaagcag......................................................................  5     1   seq
    ....................caacggaauccUaaaagcag......................................................................  3     1   seq
    ....................caacggaaucccCaaagcag......................................................................  2     1   seq
    ....................Gaacggaaucccaaaagcag......................................................................  1     1   seq
    ....................caacggaaucccaaaagcaU......................................................................  1     1   seq
    ....................caacggaauUccaaaagcagc.....................................................................  1     1   seq
    ....................caacggaaucccaaaagAagc.....................................................................  1     1   seq
    ....................caacggaauccUaaaagcagc.....................................................................  1     1   seq
    ....................caacggaaucccaaaagcagU.....................................................................  3     1   seq
    ....................caacgUaaucccaaaagcagc.....................................................................  1     1   seq
    ....................caacggaaucccaaaagcagG.....................................................................  1     1   seq
    ....................caacggaaucccaaaagUagc.....................................................................  17    1   seq
    ....................caacggaaucccaaaagcagA.....................................................................  19    1   seq
    ....................caacggaaucccaaaagcagc.....................................................................  1365  0   seq
    ....................caacggaaucUcaaaagcagc.....................................................................  14    1   seq
```

Mature   Star

```
cuuuguggugccggcgcgggcaacggaaucccaaaagcagcuguccccgcugugcccagcugcccugggguuucguuacccgcgcugccgccccugccucgccccacagc
....................caGcggaaucccaaaagcagc.....................................................................	1	1	seq
....................caacggaauccAaaaagcagc.....................................................................	1	1	seq
....................Aaacggaaucccaaaagcagc.....................................................................	2	1	seq
....................caacggaaucccaaaaAcagc.....................................................................	1	1	seq
....................caacggaaucccaaaagcagcA....................................................................	63	1	seq
....................caacggaauccUaaaagcagcu....................................................................	8	1	seq
....................caacggaaucccaaaagAagcu....................................................................	6	1	seq
....................Uaacggaaucccaaaagcagcu....................................................................	7	1	seq
....................caacggaaucccaaaagcaUcu....................................................................	1	1	seq
....................caacggaaucccaaaagcaAcu....................................................................	1	1	seq
....................caacggaaucGcaaaagcagcu....................................................................	1	1	seq
....................caacAgaaucccaaaagcagcu....................................................................	1	1	seq
....................caacggaaucccaaaaAcagcu....................................................................	1	1	seq
....................caacggaaucccaCaagcagcu....................................................................	1	1	seq
....................caacggaauccAaaaagcagcu....................................................................	1	1	seq
....................caacggaaucccaaaaCcagcu....................................................................	2	1	seq
....................Gaacggaaucccaaaagcagcu....................................................................	6	1	seq
....................caacggaaucccaaaagGagcu....................................................................	1	1	seq
....................caacggaaucccaUaagcagcu....................................................................	1	1	seq
....................caacggaaucccaaaCgcagcu....................................................................	1	1	seq
....................caacggaaucccaaaGgcagcu....................................................................	2	1	seq
....................caacggaaucccaaaaUcagcu....................................................................	1	1	seq
....................caacgAaaucccaaaagcagcu....................................................................	1	1	seq
....................caacggaaucccCaaagcagcu....................................................................	2	1	seq
....................caacCgaaucccaaaagcagcu....................................................................	1	1	seq
....................caacggaUucccaaaagcagcu....................................................................	1	1	seq
....................caacUgaaucccaaaagcagcu....................................................................	1	1	seq
....................Aaacggaaucccaaaagcagcu....................................................................	8	1	seq
....................caacggaaucccaaaagcGgcu....................................................................	1	1	seq
....................caacggaauUccaaaagcagcu....................................................................	4	1	seq
....................caacggaauAccaaaagcagcu....................................................................	2	1	seq
....................caacggaaucccaaaagcagcC....................................................................	4	1	seq
....................caacggaaucccaaaagUagcu....................................................................	190	1	seq
....................caaAggaaucccaaaagcagcu....................................................................	2	1	seq
....................caacggaaucccaaaagcagcu....................................................................	9590	0	seq
....................caacggaaucAcaaaagcagcu....................................................................	2	1	seq
....................caacggGaucccaaaagcagcu....................................................................	2	1	seq
....................caacggaaucccaaaagcagcG....................................................................	30	1	seq
....................caacggaaucccaaaagcagUu....................................................................	1	1	seq
....................caGcggaaucccaaaagcagcu....................................................................	3	1	seq
....................caacgCaaucccaaaagcagcu....................................................................	4	1	seq
....................caacggaaucUcaaaagcagcu....................................................................	45	1	seq
....................caaGggaaucccaaaagcagcu....................................................................	1	1	seq
....................cGacggaaucccaaaagcagcu....................................................................	1	1	seq
....................caacggUaucccaaaagcagcu....................................................................	1	1	seq
....................caacggaaucccaaaagcUgcug...................................................................	6	1	seq
....................caacggaauccAaaaagcagcug...................................................................	10	1	seq
....................Aaacggaaucccaaaagcagcug...................................................................	45	1	seq
....................caacggaaucAcaaaagcagcug...................................................................	8	1	seq
....................caacggaaucccaaaagcagGug...................................................................	3	1	seq
....................cGacggaaucccaaaagcagcug...................................................................	3	1	seq
....................caacggaaGcccaaaagcagcug...................................................................	1	1	seq
....................caacggaaucccaaaGgcagcug...................................................................	3	1	seq
....................caacggaaucccaGaagcagcug...................................................................	5	1	seq
....................caacggaaucccaaaaUcagcug...................................................................	11	1	seq
....................caaGggaaucccaaaagcagcug...................................................................	2	1	seq
....................caacggaaucccaaaagAagcug...................................................................	8	1	seq
....................caacgAaaucccaaaagcagcug...................................................................	3	1	seq
....................caacgCaaucccaaaagcagcug...................................................................	5	1	seq
....................caacggaauGccaaaagcagcug...................................................................	3	1	seq
....................caacggaaucccaaGagcagcug...................................................................	6	1	seq
....................caacggUaucccaaaagcagcug...................................................................	3	1	seq
....................caacggaUucccaaaagcagcug...................................................................	1	1	seq
....................caacggaaucccaaaagcagcuC...................................................................	192	1	seq
....................caaUggaaucccaaaagcagcug...................................................................	3	1	seq
....................caacggaaAcccaaaagcagcug...................................................................	5	1	seq
....................caacggaaucccaaaagcaAcug...................................................................	4	1	seq
....................caacggaaucccaaCagcagcug...................................................................	1	1	seq
....................caacggaaucccaaaagcaUcug...................................................................	9	1	seq
....................caacggaaucccaUaagcagcug...................................................................	3	1	seq
```

Mature Star

cuuuguggugccggcgcgggcaacggaaucccaaaagcagcugucccccgcugugcccagcugcccuggggguuucguuacccgcgcugccgccccugccucgccccacagc			
....................caGcggaaucccaaaagcagcug..	16	1	seq
....................caacggaaucccaaaagUagcug..	227	1	seq
....................Uaacggaaucccaaaagcagcug..	6	1	seq
....................caacggaaucccaCaagcagcug..	2	1	seq
....................caacggaaucccaaaagGagcug..	1	1	seq
....................caacggaaucccaaaCgcagcug..	1	1	seq
....................caacUgaaucccaaaagcagcug..	7	1	seq
....................caacggaaucccaaaagcagcAg..	3	1	seq
....................caacgUaaucccaaaagcagcug..	4	1	seq
....................caacggaaucccaaaagcagcCg..	10	1	seq
....................caacggaaucccaaaagcGgcug..	6	1	seq
....................caacggaaucccaaaagcagcuA..	150	1	seq
....................caacggaGucccaaaagcagcug..	2	1	seq
....................caacAgaaucccaaaagcagcug..	8	1	seq
....................caCcggaaucccaaaagcagcug..	2	1	seq
....................caacCgaaucccaaaagcagcug..	2	1	seq
....................caacggaaucccaaaagcagcug..	47861	0	seq
....................caacggaauUccaaaagcagcug..	5	1	seq
....................caacggaaucGcaaaagcagcug..	3	1	seq
....................caacggCaucccaaaagcagcug..	2	1	seq
....................caacggaaCcccaaaagcagcug..	2	1	seq
....................caacggaauccUaaaagcagcug..	20	1	seq
....................caacggaaucUcaaaagcagcug..	8	1	seq
....................caacggaaucccaaaagcagAug..	6	1	seq
....................caacggaaucccGaaagcagcug..	6	1	seq
....................Gaacggaaucccaaaagcagcug..	42	1	seq
....................caacggGaucccaaaagcagcug..	6	1	seq
....................caacggaaucccaaaagcaCcug..	2	1	seq
....................caacggaaucccaaaagcagcuU..	1688	1	seq
....................caaAggaaucccaaaagcagcug..	14	1	seq
....................caacggaaucccaaaaAcagcug..	18	1	seq
....................caacggaaucccCaagcagcug..	6	1	seq
....................caacggaaucccaaaagcagUug..	1	1	seq
....................caacggaauccGaaaagcagcug..	3	1	seq
....................caacggaaucccaaaaCcagcug..	2	1	seq
....................caacggaauAccaaaagcagcug..	7	1	seq
....................caUcggaaucccaaaagcagcug..	2	1	seq
....................caacggaaucccaaaagcagcGg..	75	1	seq
....................caacggaauccAaaaagcagcugu...	1	1	seq
....................caacggaaucccaaaagcaCcugu...	1	1	seq
....................caacggaaucccaaaagcagcugC...	4	1	seq
....................caacgUaaucccaaaagcagcugu...	3	1	seq
....................caacggaaucccaaaagcagcAgu...	1	1	seq
....................caacggaaucccaaaagcagcuUu...	341	1	seq
....................caGcggaaucccaaaagcagcugu...	3	1	seq
....................Gaacggaaucccaaaagcagcugu...	2	1	seq
....................caacggaaucccaaaagcaAcugu...	1	1	seq
....................caacggaaucccaaaagcagcugA...	126	1	seq
....................caacggaauUccaaaagcagcugu...	1	1	seq
....................caacggaaucccaaaaAcagcugu...	3	1	seq
....................caacggaaucccaaaagUagcugu...	12	1	seq
....................caacggaaucccaaaagcagcuAu...	49	1	seq
....................Uaacggaaucccaaaagcagcugu...	2	1	seq
....................caacggaaucccaaaagcagcugu...	3778	0	seq
....................caacggaaucccaGaagcagcugu...	1	1	seq
....................caacggaaucccaaaagAagcugu...	2	1	seq
....................caacggaauccUaaaagcagcugu...	1	1	seq
....................caacggUaucccaaaagcagcugu...	2	1	seq
....................caacggaaucccaaaagcGgcugu...	2	1	seq
....................caacggaaucUcaaaagcagcugu...	2	1	seq
....................caaAggaaucccaaaagcagcugu...	1	1	seq
....................caacggaaucccaaaagcagcugG...	9	1	seq
....................caacggaaucccaaaagcagcuCu...	11	1	seq
....................caacggaaucccaaaGgcagcugu...	1	1	seq
....................Aaacggaaucccaaaagcagcugu...	1	1	seq
....................caacgAaaucccaaaagcagcugu...	1	1	seq
....................caacggaaucccaaaagcagcuguA..	12	1	seq
....................caacggaaucccaaaagcagcuguU..	17	1	seq
....................caacggaaucccaaaagcagcuguG..	1	1	seq
....................caacggaaucccaaaagcagcuguccc..	1	0	seq

```
                   Mature                                Star
cuuguggugccggcgcgggcaacggaaucccaaaagcagcuguccccgcugugcccagcugcccugggguuucguuacccgcgcugccgccccugccucgccccacagc
...................caacggaaucccaaaagcagcuguccccgc............................................................ 2   0 seq
....................aacggaaucccaaaagcag...................................................................... 1   0 seq
....................aacggaaucccaaaagcagc..................................................................... 1   0 seq
....................aacggaaucccaaaagcagcu.................................................................... 15  0 seq
....................aacggaaucccaaaagcaUcug................................................................... 1   1 seq
....................aacggaaucccaaaagcagcuU................................................................... 3   1 seq
....................aGcggaaucccaaaagcagcug................................................................... 1   1 seq
....................aacggaaucccaaaagcagcug................................................................... 125 0 seq
....................aacggaauccUaaaagcagcug................................................................... 1   1 seq
....................aacggaaucccaaaagcagcugu.................................................................. 17  0 seq
.....................acggaaucccaaaagcagcug................................................................... 8   0 seq
.....................acggaaucccaaaagcagcuC................................................................... 1   1 seq
.....................acggaaucccaaaagcagcuUu.................................................................. 1   1 seq
.....................acggaaucccaaaagcagcugu.................................................................. 16  0 seq
.....................acggaaucccaaaagcagcuguA................................................................. 1   1 seq
........................gaaucccaaaagcagcug................................................................... 1   0 seq
.........................................................gcugcccugggguuucguuacc.............................. 3   0 seq
..........................................................cugcccugggguuucguuaU............................... 1   1 seq
..........................................................cugcccugggguuucguuac............................... 9   0 seq
..........................................................cugcccugggguuucguuGcc.............................. 1   1 seq
..........................................................cugcccugggguuucguuacc.............................. 19  0 seq
..........................................................cugcccugggguuucguuacU.............................. 6   1 seq
..........................................................cugcccugggguuucguuaccU............................. 7   1 seq
..........................................................cugcccugggguuucguuaccc............................. 1   0 seq
..........................................................cugcccugggguuucguuaccA............................. 1   1 seq
..........................................................cugcccugggguuucguuacccU............................ 1   1 seq
...........................................................ugcccugggguuucguuaccc............................ 1   0 seq
```

Provisional ID : mun-miR-16a
Score total : 3120
Score for star read(s) : 3.9
Score for read counts : 3110.1
Score for mfe : 1.4
Score for randfold : 1.6
Score for cons. seed : 3
Total read count : 6112
Mature read count : 6075
Loop read count : 17
Star read count : 20

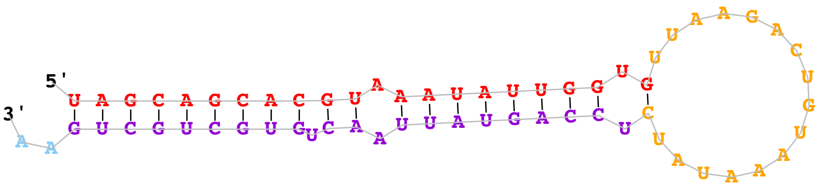

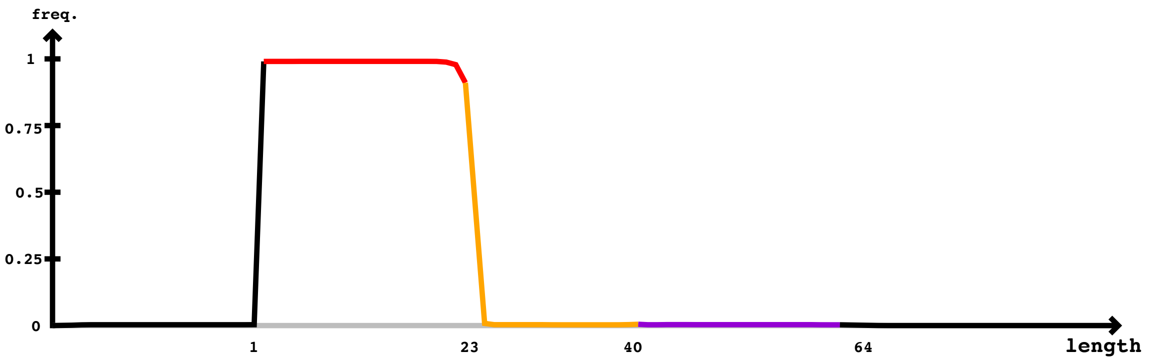

Mature Star

```
5'- ugcugaugucugucauaccuuagcagcacguaaauauugguguuaagacuguaaauaucuccaguauuaacugugcugcugaaguaaggcuagccacuuccgcauguga  -3'   obs
    ugcugaugucugucauaccuuagcagcacguaaauauugguguuaagacuguaaauaucuccaguauuaacugugcugcugaaguaaggcuagccacuuccgcauguga         exp
    (((.((.((..(((.(((.(((((((((((.((((((((.(..................).)))))))).)).))))))))).))).)))..))....)).))).....   reads   mm   sample
    ugcugaugucugucauaccu...........................................................................................   8       0    seq
    .gcugaugucugucauaccu...........................................................................................   8       0    seq
    ..cugaugucugucauaccu...........................................................................................   3       0    seq
    ...................Guagcagcacguaaauauuggug...................................................................   1       1    seq
    ....................uagcagcacguaaauauu.......................................................................   1       0    seq
    ....................uagcagcacguaaauauug......................................................................   15      0    seq
    ....................uagcagcacguaaauauuU......................................................................   1       1    seq
    ....................uagcagcacgAaaauauugg.....................................................................   1       1    seq
    ....................uagcagcacguaaauauugg.....................................................................   56      0    seq
    ....................uagcagcacguaaauauugU.....................................................................   2       1    seq
    ....................uagcagcacAuaaauauuggu....................................................................   1       1    seq
    ....................uagcagcacguaaauauuggA....................................................................   6       1    seq
    ....................uagcagcacUuaaauauuggu....................................................................   1       1    seq
    ....................uagcagcacguaaauauuggu....................................................................   408     0    seq
    ....................uagcagcacguaaauauugguC...................................................................   3       1    seq
    ....................uagcagcacguaaauaGuggug...................................................................   1       1    seq
    ....................uagcagcGcguaaauauuggug...................................................................   1       1    seq
    ....................uagcagcacguaaauauugUug...................................................................   1       1    seq
    ....................uagcagcacguaaauauuggGg...................................................................   4       1    seq
    ....................uagcagcacguGaauauuggug...................................................................   1       1    seq
    ....................uaUcagcacguaaauauuggug...................................................................   1       1    seq
    ....................uagcagcacgGaaauauuggug...................................................................   1       1    seq
    ....................Aagcagcacguaaauauuggug...................................................................   2       1    seq
    ....................uagcagUacguaaauauuggug...................................................................   1       1    seq
    ....................uagcagcacguaaauaCuggug...................................................................   1       1    seq
    ....................uagcagcacguaaauauuggCg...................................................................   5       1    seq
    ....................uagcagcacguaaauauuAgug...................................................................   2       1    seq
    ....................uagcagcacguaGauauuggug...................................................................   1       1    seq
    ....................Cagcagcacguaaauauuggug...................................................................   1       1    seq
    ....................uagcagcacguaaauUuuggug...................................................................   1       1    seq
    ....................uagcagcacguaaauauugCug...................................................................   1       1    seq
    ....................Gagcagcacguaaauauuggug...................................................................   2       1    seq
    ....................uagcagcacgCaaauauuggug...................................................................   2       1    seq
```

```
                    Mature                                   Star
ugcugaugucugucauaccuuagcagcacguaaaauauugguguuaagacuguaaaauaucuccaguauuaacugugcugcugaaguaaggcuagccacuuccgcauguga
....................uagcagcaUguaaaauauuggug....................................................................	5	1	seq
....................uagcagcacguaaaauauuggAg....................................................................	2	1	seq
....................uUgcagcacguaaaauauuggug....................................................................	1	1	seq
....................uagcagcacguaaaauauugguA....................................................................	68	1	seq
....................uagcagcacCuaaaauauuggug....................................................................	1	1	seq
....................uagcagcacguaaaauauugguU....................................................................	12	1	seq
....................uagcagcacUuaaaauauuggug....................................................................	1	1	seq
....................uagcagcacguaaaauauuggug....................................................................	4114	0	seq
....................uagcagcacguaaaauauuggugG...................................................................	74	1	seq
....................uaUcagcacguaaaauauuggugu...................................................................	1	1	seq
....................uagcagcacguaaaauauugguUu...................................................................	1	1	seq
....................uagcagcacguaaaauauuCgugu...................................................................	1	1	seq
....................uagcagcacguaaaauauuggugA...................................................................	571	1	seq
....................uagcagcaAguaaaauauuggugu...................................................................	1	1	seq
....................Cagcagcacguaaaauauuggugu...................................................................	1	1	seq
....................uagcagcacguaaaauauuggugu...................................................................	653	0	seq
....................uagcagcacguaaaauauuggugC...................................................................	3	1	seq
....................uagcagcacguaaaauauugguAu...................................................................	8	1	seq
....................uagcagcacguaaaauauugguguu..................................................................	25	0	seq
....................uagcagcacguaaaauauuggugAu..................................................................	5	1	seq
....................uagcagcacguaaaauauugguguuaagacu............................................................	2	0	seq
....................uagcagcacguaaaauauugguguuaagacug...........................................................	1	0	seq
.....................agcagcacguaaaauauuggugu...................................................................	1	0	seq
........................agcacguaaaauauuggug....................................................................	1	0	seq
...........................................uuaagacuguaaaauaucu.................................................	16	0	seq
...........................................uuaagacuguaaaauaucuccagu............................................	1	0	seq
...........................................................ucuccaguauuaacugugcug...............................	1	0	seq
............................................................cuccaguauuaacugugcugU..............................	1	1	seq
............................................................cuccaguauuaacugugcugcug............................	2	0	seq
............................................................cuccaguauuaacugugcugcugU...........................	1	1	seq
.............................................................uccaguauuaacugugcug...............................	2	0	seq
.............................................................uccaguauuaacugugcugcug............................	3	0	seq
.............................................................uccaguauuaacugugcugcugU...........................	2	1	seq
.............................................................uccaguauuaacugugcugcugaaU.........................	1	1	seq
..............................................................ccaguauuaacugugcugcuga...........................	1	0	seq
..............................................................ccaguauuaacugugcugcugU...........................	2	1	seq
..............................................................ccaguauuaacugugcugcugaa..........................	1	0	seq
..............................................................ccaguauuaacugugcugcugaU..........................	1	1	seq
................................................................aguauuaacugugcugcugaaU.........................	2	1	seq
................................................................aguauuaacugugcugcugaaAu........................	1	1	seq
```

Provisional ID	:	mun-miR-365
Score total	:	245.9
Score for star read(s)	:	3.9
Score for read counts	:	235.7
Score for mfe	:	1.8
Score for randfold	:	1.6
Score for cons. seed	:	3
Total read count	:	474
Mature read count	:	469
Loop read count	:	0
Star read count	:	5

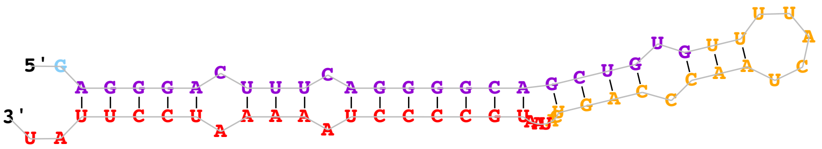

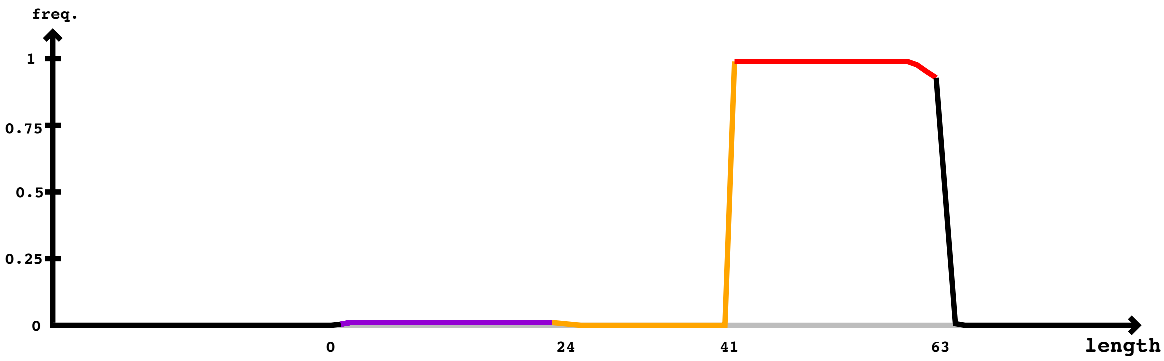

Star Mature

```
5'- ggacagacugagcggcagcaagaaaaaugagggacuuucaggggcagcuguguuuuacuaacccagucauaaugccccuaaaaauccuuauuguucuugcaauggucaacu   -3'   obs
    ggacagacugagcggcagcaagaaaaaugagggacuuucaggggcagcuguguuuuacuaacccagucauaaugccccuaaaaauccuuauuguucuugcaauggucaacu         exp
    ........(((.((...(((((((.((((((((..(((.(((((((((((.(((.....))).)))).....))))))))))..)))))))).)))))))..)).)))...    reads   mm   sample
    ............................gagggacuuucaggggcagcugA............................................................    1       1    seq
    ............................gagggacuuucaggggcagcuguA...........................................................    1       1    seq
    .............................agggacuuucaggggcagcugug...........................................................    2       0    seq
    .............................agggacuuucaggggcagcugugu..........................................................    1       0    seq
    .....................................................................uaaugccccuaaaaauccu.......................    6       0    seq
    .....................................................................uaaugccccuaaaaauccuu......................    12      0    seq
    .....................................................................uaaugccccuaaaaauccuuU.....................    3       1    seq
    .....................................................................uaaugccccuaaaaauccuua.....................    8       0    seq
    .....................................................................uaaugccccuaaaaauccuuaC....................    19      1    seq
    .....................................................................uaaugccccuaaaaauccuuaA....................    7       1    seq
    .....................................................................uaaugccccuaaaaauccuuau....................    328     0    seq
    .....................................................................uaaugccccuaaaaauccuuUu....................    1       1    seq
    .....................................................................uaaugccccuGaaaauccuuau....................    12      1    seq
    .....................................................................uaaugccccuaaUaauccuuau....................    1       1    seq
    .....................................................................uaaugccccuaaaaauccuuaG....................    1       1    seq
    .....................................................................uaaugcccAuaaaaauccuuau....................    1       1    seq
    .....................................................................uaaugccccuGaaaauccuuauu...................    3       1    seq
    .....................................................................uaaugccccuaaaaauccuuaCu...................    10      1    seq
    .....................................................................uaaugccccuaaaaauccuuaGu...................    1       1    seq
    .....................................................................Caaugccccuaaaaauccuuauu...................    1       1    seq
    .....................................................................uaaugccccuaaaaauccuuaAu...................    3       1    seq
    .....................................................................uaaugccccuaaaaauccuuauA...................    2       1    seq
    .....................................................................uaaugccccuaaaaauccuuauu...................    47      0    seq
    .....................................................................uaaugccccuaaaaauccuuauuU..................    3       1    seq
```

Provisional ID	:	mun-miR-181b-1
Score total	:	4454
Score for star read(s)	:	3.9
Score for read counts	:	4444.3
Score for mfe	:	1.3
Score for randfold	:	1.6
Score for cons. seed	:	3
Total read count	:	8729
Mature read count	:	8708
Loop read count	:	1
Star read count	:	20

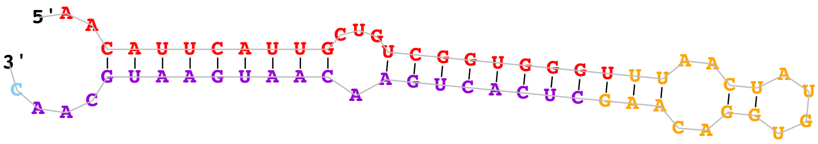

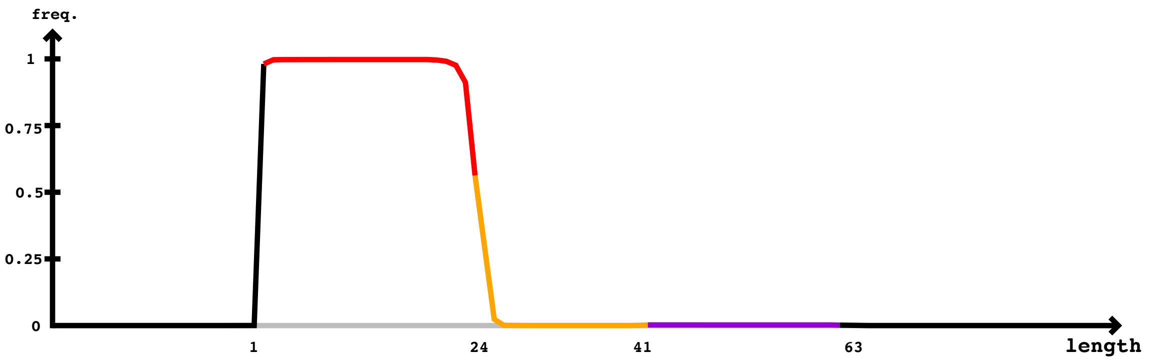

Mature Star

```
5'- uuacaaaaaaggucacaaucaacauucauugcugucgguggguuuaacuauguggacaagcucacugaacaaugaaugcaacuguggccccacauuuugcaguuaccga -3'   obs
    uuacaaaaaaggucacaaucaacauucauugcugucgguggguuuaacuauguggacaagcucacugaacaaugaaugcaacuguggccccacauuuugcaguuaccga       exp
    ...(((((..(((((((.....(((((((((...(((((((((((..((....))..))))))))))).)))))))))....))))))).....)))))..........   reads  mm  sample
    .................aucaacauucauugcugucggugggu..................................................................   1      0   seq
    ...................caacauucauugcugucggugggu..................................................................   1      0   seq
    ....................aacauucauugcugucgg.......................................................................   16     0   seq
    ....................aacauucauugcugucggu......................................................................   39     0   seq
    ....................aacauucaAugcugucggu......................................................................   1      1   seq
    ....................aacauucauugcugucggug.....................................................................   118    0   seq
    ....................aacauucauugcugucgguA.....................................................................   8      1   seq
    ....................aacauucauugcugucgguU.....................................................................   2      1   seq
    ....................aacauucauugcuguUggug.....................................................................   1      1   seq
    ....................aacauucauugcugucggGg.....................................................................   1      1   seq
    ....................aacauucauugcugucggugA....................................................................   298    1   seq
    ....................aacauucauugcugucggCgg....................................................................   1      1   seq
    ....................Uacauucauugcugucggugg....................................................................   1      1   seq
    ....................aacauucauugcugucggGgg....................................................................   8      1   seq
    ....................aacauucauuCcugucggugg....................................................................   1      1   seq
    ....................aacauucauugcugucggugg....................................................................   185    0   seq
    ....................aacauucauugcugucggugU....................................................................   65     1   seq
    ....................aacauucauCgcugucgguggg...................................................................   1      1   seq
    ....................aacauucauugcugucgguggC...................................................................   1      1   seq
    ....................aacauucauugcugucgguggU...................................................................   37     1   seq
    ....................aacauucauugcAgucgguggg...................................................................   1      1   seq
    ....................aacauucauugcugucggCggg...................................................................   1      1   seq
    ....................aacauAcauugcugucgguggg...................................................................   1      1   seq
    ....................Gacauucauugcugucgguggg...................................................................   1      1   seq
    ....................aacauucCuugcugucgguggg...................................................................   1      1   seq
    ....................aacauucauugGugucgguggg...................................................................   1      1   seq
    ....................aacauucauugcugucgguggA...................................................................   780    1   seq
    ....................aacauucauugcugucUguggg...................................................................   3      1   seq
    ....................aacauucauugcugucggugAg...................................................................   2      1   seq
    ....................aacauucauugcuguAgguggg...................................................................   1      1   seq
    ....................aacauucauugcugucAguggg...................................................................   1      1   seq
    ....................aacauucauugcuUucgguggg...................................................................   1      1   seq
    ....................aacauucauugcugucggAggg...................................................................   1      1   seq
```

Mature Star

```
uuacaaaaaaggucacaaucaacauucauugcugucgguggguuuaacuauguggacaagcucacugaacaaugaaugcaacuguggccccacauuuugcaguuaccga
....................aacauucGuugcugucgguggg...................................................................  1     1  seq
....................aacauucauugcugucgguUgg...................................................................  1     1  seq
....................aGcauucauugcugucgguggg...................................................................  1     1  seq
....................aacauucauugcugucggGggg...................................................................  6     1  seq
....................aacauucauugcugAcgguggg...................................................................  1     1  seq
....................aacauucauugcCgucgguggg...................................................................  1     1  seq
....................aacauucaAugcugucgguggg...................................................................  1     1  seq
....................aacauucauugcugucgguggg...................................................................  2179  0  seq
....................aacauucauugcugucUgugggu..................................................................  1     1  seq
....................aacauucauugcugucgguggAu..................................................................  40    1  seq
....................aacauucauCgcugucggugggu..................................................................  1     1  seq
....................aacauuUauugcugucggugggu..................................................................  1     1  seq
....................Gacauucauugcugucggugggu..................................................................  2     1  seq
....................aacauucauugcugucggugggu..................................................................  2613  0  seq
....................aacauucauugcugucggugggA..................................................................  417   1  seq
....................aacauucauugcugucggCgggu..................................................................  2     1  seq
....................aacauucaAugcugucggugggu..................................................................  1     1  seq
....................aacauucauugcugucggAgggu..................................................................  2     1  seq
....................aacauucauugUugucggugggu..................................................................  1     1  seq
....................aacauucauugcugucggugggG..................................................................  104   1  seq
....................aacauucauugcugucggGgggu..................................................................  1     1  seq
....................aacauAcauugcugucggugggu..................................................................  1     1  seq
....................aacauucauugcugucgguggUu..................................................................  7     1  seq
....................aacauucaCugcugucggugggu..................................................................  1     1  seq
....................aacauucauugcuUucggugggu..................................................................  1     1  seq
....................aacauucauugcugGcggugggu..................................................................  2     1  seq
....................aacauucauugcuguUggugggu..................................................................  2     1  seq
....................aacauucauugcugucgguggCu..................................................................  3     1  seq
....................aacauucauugcuguAggugggu..................................................................  1     1  seq
....................aaUauucauugcugucggugggu..................................................................  1     1  seq
....................aacauuAauugcugucggugggu..................................................................  1     1  seq
....................aacauucauugcugucggugggC..................................................................  4     1  seq
....................aacauucUuugcugucggugggu..................................................................  1     1  seq
....................aacauucauugcugucggugUgu..................................................................  1     1  seq
....................aacauucaAugcugucgguggguu.................................................................  1     1  seq
....................aacauucauugcugucggGggguu.................................................................  2     1  seq
....................Gacauucauugcugucgguggguu.................................................................  1     1  seq
....................aacauucauugcugucggAggguu.................................................................  1     1  seq
....................aacauucauugcugucgguggguG.................................................................  10    1  seq
....................aacauucauugcugucgguggCuu.................................................................  1     1  seq
....................aacauucauugcuguAgguggguu.................................................................  1     1  seq
....................aacauucauugcugucgguggguu.................................................................  1327  0  seq
....................aacauucauugcugucggugggGu.................................................................  4     1  seq
....................aacauucauugcugucggugggAu.................................................................  28    1  seq
....................aacauucauugcugucgguggguA.................................................................  20    1  seq
....................aacauGcauugcugucgguggguu.................................................................  1     1  seq
....................aacauucauugcugucggugggGuu................................................................  1     1  seq
....................aacauucauugcugucggCggguuu................................................................  1     1  seq
....................aacauucauugcugucgguggguuA................................................................  5     1  seq
....................aacauucauuUcugucgguggguuu................................................................  1     1  seq
....................aacauucauugcugucggugggCuu................................................................  1     1  seq
....................aacauucauugcugucgguggguuC................................................................  4     1  seq
....................aacauucauugcugucgguggguCu................................................................  1     1  seq
....................aacauucauugcugucgguggguuu................................................................  153   0  seq
....................aacauucauugcugucgguggguuG................................................................  2     1  seq
....................aacauucauugcugucgguggguAu................................................................  11    1  seq
....................aacauucauugcugucgguggguuua...............................................................  1     0  seq
....................aacauucauugcugucgguggguuuC...............................................................  1     1  seq
....................aacauucauugcugucgguggguuCa...............................................................  1     1  seq
....................aacauucauugcugucgguggguuuaacu............................................................  1     0  seq
....................aacauucauugcugucgguggguuuaacuauguggaca...................................................  1     0  seq
.....................acauucauugcugucggug.....................................................................  1     0  seq
.....................acauucauugcugucggugg....................................................................  2     0  seq
.....................acauucauugcugucggugA....................................................................  1     1  seq
.....................acauucauugcugucgguggg...................................................................  14    0  seq
.....................acauucauugcugucggGggg...................................................................  1     1  seq
.....................acauucauugcugucgguggA...................................................................  6     1  seq
.....................acauucauugcugucggugggA..................................................................  2     1  seq
.....................acauucauugcugucggugggu..................................................................  35    0  seq
.....................acauucauugcugucggugggG..................................................................  3     1  seq
```

```
                    Mature                                      Star
uuacaaaaaaggucacaaucaacauucauugcugucggugggguuuaacuauguggacaagcucacugaacaaugaaugcaacuguggccccacauuuugcaguuaccga
.....................acauucauugcugucgguggguA.................................................................   1   1   seq
.....................acauucauugcugucgguggguu.................................................................   52  0   seq
.....................acauucauugcugucgguggguCu................................................................   1   1   seq
.....................acauucauugcugucgguggguuu................................................................   18  0   seq
......................cauucauugcugucgguggguu.................................................................   3   0   seq
......................cauucauugcugucgguggguuu................................................................   2   0   seq
........................uucauugcugucgguggg...................................................................   1   0   seq
........................uucauugcugucggugggu..................................................................   1   0   seq
...........................auugcugucgguggguuuaa..............................................................   1   0   seq
............................................uaacuauguggacaagcucacu...........................................   1   0   seq
...........................................................gcucacugaacaaugaaugca.............................   5   0   seq
............................................................cucacugaacaaugaaugca.............................   1   0   seq
............................................................cucacugaacaaugaaugcaU............................   8   1   seq
............................................................cucacugaacaaugaaugcaa............................   2   0   seq
............................................................cucacugaacaaugaaugcaaU...........................   2   1   seq
............................................................cucacugaacaaugaaugcaacu..........................   1   0   seq
............................................................cucacugaacaaugaaugcaacA..........................   1   1   seq
```

Provisional ID	:	mun-miR-338-1
Score total	:	5.4
Score for star read(s)	:	-1.3
Score for read counts	:	0
Score for mfe	:	2.1
Score for randfold	:	1.6
Score for cons. seed	:	3
Total read count	:	3112
Mature read count	:	3031
Loop read count	:	0
Star read count	:	81

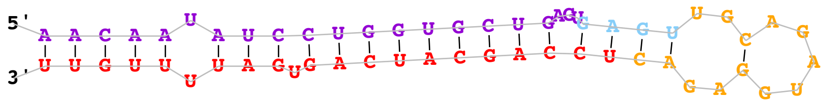

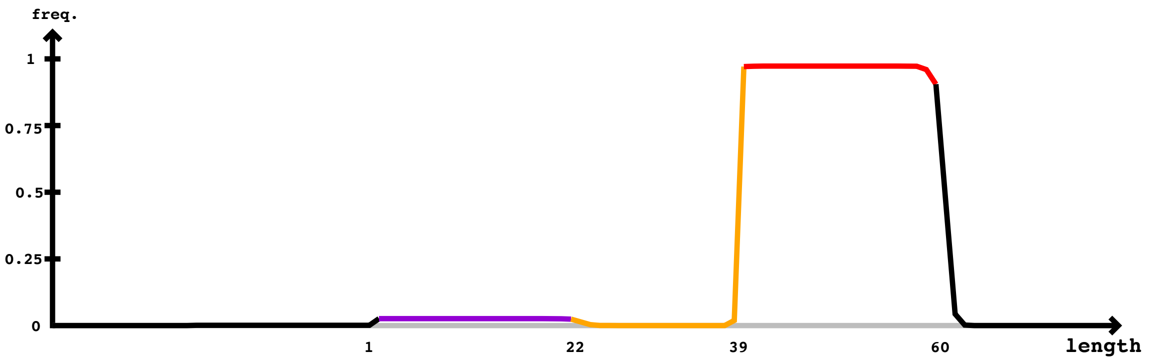


```
                                   Star                                    Mature
5'- ucgcucccgccccaccacugcuccuccugcccaacaauauccuggugcugagugaguugcagauggagacuccagcaucagugauuuuguugaggagggggugcauuga  -3'   obs
    ucgcucccgccccaccacugcuccuccugcccaacaauauccuggugcugagugaguugcagauggagacuccagcaucagugauuuuguugaggagggggugcauuga         exp
    ..((....)).....((.(((.((((((.((((((((.(((((((((((((...((((..(.....)..)))))))))))))).))).)))))).)))))))).))).)).   reads   mm   sample
    .............accacugcuccuccugccc.............................................................................   3       0    seq
    ................................aacaauauccuggugcug...........................................................   1       0    seq
    ................................aacaauauccuggugcuga..........................................................   1       0    seq
    ................................aacaauauccuggugcugag.........................................................   4       0    seq
    ................................aacaauauccuggugcugagA........................................................   1       1    seq
    ................................aacaauauccuggugcugagu........................................................   59      0    seq
    ................................aacaauauccuggugcugagug.......................................................   4       0    seq
    ................................aacaauauccuggugcugagugU......................................................   7       1    seq
    ................................aacaauauccuggugcugaguga......................................................   3       0    seq
    ................................aacaauauccuggugcugagugagu....................................................   1       0    seq
    .....................................................................cuccagcaucagugauuu......................   1       0    seq
    .....................................................................cuccagcaucagugauuuug....................   3       0    seq
    .....................................................................cuccagcaucagugauuuugu...................   1       0    seq
    .....................................................................cuccagcaucagugauuuuguA..................   1       1    seq
    .....................................................................cuccagcaucagugauuuuguu..................   43      0    seq
    .....................................................................cuccagcaucagugauuuuguug.................   9       0    seq
    .....................................................................cuccagcaucagugauuuuguuga................   2       0    seq
    ......................................................................uccagcaucagugauuuu.....................   1       0    seq
    ......................................................................uccagcaucagugauuuug....................   36      0    seq
    ......................................................................uccagcaucagugauuuugA...................   4       1    seq
    ......................................................................uccagcaucagugauuuugu...................   163     0    seq
    ......................................................................uccagcaucagugGuuuugu...................   1       1    seq
    ......................................................................uccagcaucagugauuuugAu..................   1       1    seq
    ......................................................................uccaAcaucagugauuuuguu..................   1       1    seq
    ......................................................................uccagcaucagugauuuuCuu..................   1       1    seq
    ......................................................................uccagcaucagugauuuuAuu..................   1       1    seq
    ......................................................................uccagcauUagugauuuuguu..................   1       1    seq
    ......................................................................uccagcaucagugauAuuguu..................   1       1    seq
    ......................................................................uccagcaucagugaCuuuguu..................   1       1    seq
    ......................................................................Gccagcaucagugauuuuguu..................   1       1    seq
    ......................................................................uccagcaucagugaAuuuguu..................   1       1    seq
    ......................................................................uccagcaucagCgauuuuguu..................   1       1    seq
    ......................................................................uccagcaucaAugauuuuguu..................   1       1    seq
```

```
                                Star                                 Mature
ucgcucccgccccaccacugcuccuccugcccaacaauauccuggugcugagugaguugcagauggagacuccagcaucagugauuuuguugaggaggggugcauuga
......................................................................uccagUaucagugauuuuguu.................  2     1  seq
......................................................................uccagcaucagugauuuuguu.................  1719  0  seq
......................................................................uccagcaucagugauuuuguA.................  19    1  seq
......................................................................uccagcaucagugauuuuguG.................  1     1  seq
......................................................................uccagcaucagugauuuugAug................  1     1  seq
......................................................................uccagcaucagugauuuuguuA................  50    1  seq
......................................................................uccGgcaucagugauuuuguug................  1     1  seq
......................................................................uccagcaucagugauuuuguug................  812   0  seq
......................................................................uccagcaucagAgauuuuguug................  1     1  seq
......................................................................uccagcaucagugauCuuguug................  1     1  seq
......................................................................uccagcauAagugauuuuguug................  1     1  seq
......................................................................uUcagcaucagugauuuuguug................  2     1  seq
......................................................................uccagcaucagugauuuuguuU................  6     1  seq
......................................................................uccagcaucagugauuuuguugG...............  1     1  seq
......................................................................uccagcaucagugauuuuguAga...............  1     1  seq
......................................................................uccagcaucagugauuuuguuAa...............  4     1  seq
......................................................................uccagcaucagugauuuuguuga...............  118   0  seq
......................................................................uccagcaucagugauuuuguugU...............  3     1  seq
......................................................................uccagcaucagugauuuuguugaA..............  6     1  seq
.......................................................................ccagcaucagugauuuuguu.................  3     0  seq
.......................................................................ccagcaucagugauuuuguug................  1     0  seq
........................................................................cagcaucagugauuuugu..................  2     0  seq
```

Provisional ID : mun-miR-125-2
Score total : 20000.5
Score for star read(s) : 3.9
Score for read counts : 19990.4
Score for mfe : 1.7
Score for randfold : 1.6
Score for cons. seed : 3
Total read count : 39222
Mature read count : 38063
Loop read count : 0
Star read count : 1159

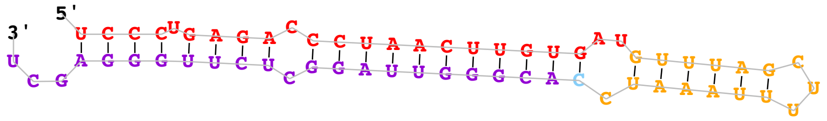

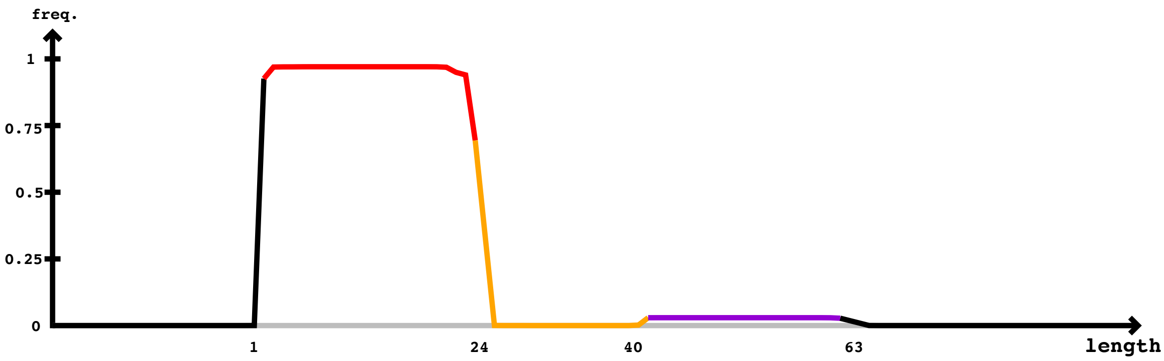


```
                     Mature                                      Star
5'- guuguugcgccccucucaaucccugagacccuaacuugugauguuuagcuuuuaaauccacggguuaggcucuugggagcugugaguugugcuuugacaucuucauuuua  -3'   obs
    guuguugcgccccucucaaucccugagacccuaacuugugauguuuagcuuuuaaauccacggguuaggcucuugggagcugugaguugugcuuugacaucuucauuuua        exp
    (((....((((..(((.((.((((.((((.(((((((((((..((((((...)))))).))))))))))).))))))))..)).)))..))))...))).........  reads  mm  sample
    ..................Cucccugagacccuaacuugu.......................................................................  1  1  seq
    ..................Cucccugagacccuaacuuguga.....................................................................  2  1  seq
    ...................ucccugagacccuaaAuu.........................................................................  1  1  seq
    ...................ucccugagacUcuaacuu.........................................................................  3  1  seq
    ...................ucccugagaUccuaacuug........................................................................  1  1  seq
    ...................ucccugagacUcuaacuug........................................................................  22  1  seq
    ...................ucccugaUacccuaacuug........................................................................  1  1  seq
    ...................ucccugagacccuaacCug........................................................................  1  1  seq
    ...................ucccugagCcccuaacuug........................................................................  1  1  seq
    ...................ucccugagacccuaacuuU........................................................................  23  1  seq
    ...................Acccugagacccuaacuug........................................................................  1  1  seq
    ...................ucccugagacccuaacuuA........................................................................  18  1  seq
    ...................ucccugagacccuaacGugu.......................................................................  1  1  seq
    ...................ucccugagacccuCacuugu.......................................................................  2  1  seq
    ...................ucccugagacccuaaUuugu.......................................................................  3  1  seq
    ...................Acccugagacccuaacuugu.......................................................................  9  1  seq
    ...................uccAugagacccuaacuugu.......................................................................  7  1  seq
    ...................ucccugagacccuaUcuugu.......................................................................  1  1  seq
    ...................ucccugagacccAaacuugu.......................................................................  1  1  seq
    ...................ucccugagacccuaacCugu.......................................................................  2  1  seq
    ...................ucccugagacccuaacuuUu.......................................................................  4  1  seq
    ...................ucccugagacccuGacuugu.......................................................................  1  1  seq
    ...................ucccCgagacccuaacuugu.......................................................................  4  1  seq
    ...................ucccugagGcccuaacuugu.......................................................................  3  1  seq
    ...................ucccugagacUcuaacuugu.......................................................................  312  1  seq
    ...................ucccGgagacccuaacuugu.......................................................................  1  1  seq
    ...................ucccugagacAcuaacuugu.......................................................................  1  1  seq
    ...................Gcccugagacccuaacuugu.......................................................................  17  1  seq
    ...................ucccugagaUccuaacuugu.......................................................................  17  1  seq
    ...................ucccugagaGccuaacuugu.......................................................................  3  1  seq
    ...................uUccugagacccuaacuugu.......................................................................  4  1  seq
    ...................ucccugagacccuaacuugA.......................................................................  260  1  seq
    ...................uccGugagacccuaacuugu.......................................................................  2  1  seq
```

```
                    Mature                                   Star
guuguugcgccccucucaaucccugagacccuaacuugugauguuuagcuuuuaaauccacggguuaggcucuugggagcugugaguugugcuuugacaucuucauuuua
...................Ccccugagacccuaacuugu.......................................................................	4	1	seq
...................ucccugagaAccuaacuugu.......................................................................	3	1	seq
...................ucccugagacccuaacuGgu.......................................................................	1	1	seq
...................ucccugagacccuaacuuCu.......................................................................	1	1	seq
...................ucccugagaccGuaacuugu.......................................................................	1	1	seq
...................uAccugagacccuaacuugu.......................................................................	2	1	seq
...................ucccugagacccuaacuugC.......................................................................	2	1	seq
...................ucccuUagacccuaacuugu.......................................................................	3	1	seq
...................ucccugagacccuaaAuugu.......................................................................	4	1	seq
...................ucccugagaccUuaacuugu.......................................................................	31	1	seq
...................ucccugagacccuaacuugG.......................................................................	18	1	seq
...................ucccugaUacccuaacuugu.......................................................................	5	1	seq
...................ucccugagaccAuaacuugu.......................................................................	2	1	seq
...................ucccugagacGcuaacuugu.......................................................................	1	1	seq
...................ucAcugagacccuaacuugu.......................................................................	4	1	seq
...................ucccugaUacccuaacuugug......................................................................	2	1	seq
...................ucccugagaUccuaacuugug......................................................................	1	1	seq
...................ucccugagaccUuaacuugug......................................................................	1	1	seq
...................ucUcugagacccuaacuugug......................................................................	1	1	seq
...................ucAcugagacccuaacuugug......................................................................	5	1	seq
...................ucccugagacccuaacuugGg......................................................................	6	1	seq
...................ucGcugagacccuaacuugug......................................................................	1	1	seq
...................uccUugagacccuaacuugug......................................................................	1	1	seq
...................ucccugagacUcuaacuugug......................................................................	94	1	seq
...................ucccugagacccuaacuuguU......................................................................	119	1	seq
...................Gcccugagacccuaacuugug......................................................................	4	1	seq
...................ucccugagacccuaaUuugug......................................................................	1	1	seq
...................uccAugagacccuaacuugug......................................................................	3	1	seq
...................uUccugagacccuaacuugug......................................................................	6	1	seq
...................ucccugagacccuaacuuguA......................................................................	115	1	seq
...................ucccugagacccuaaAuugug......................................................................	3	1	seq
...................Ccccugagacccuaacuugug......................................................................	4	1	seq
...................Acccugagacccuaacuugug......................................................................	4	1	seq
...................ucccugaCacccuaacuugug......................................................................	1	1	seq
...................ucccugGgacccuaacuugug......................................................................	1	1	seq
...................ucccugagGcccuaacuugug......................................................................	1	1	seq
...................ucccugagacAcuaacuugug......................................................................	1	1	seq
...................ucccugagacccuaacCugug......................................................................	2	1	seq
...................ucccugagacccuaacuuAug......................................................................	1	1	seq
...................ucccuUagacccuaacuugug......................................................................	1	1	seq
...................ucccugagacccuaacuugAg......................................................................	2	1	seq
...................ucccuAagacccuaacuugug......................................................................	1	1	seq
...................ucccugagacccCaacuugug......................................................................	1	1	seq
...................ucccugagacccuGacuugug......................................................................	1	1	seq
...................ucccugCgacccuaacuugug......................................................................	1	1	seq
...................ucccuUagacccuaacuuguga.....................................................................	2	1	seq
...................ucccugagacAcuaacuuguga.....................................................................	4	1	seq
...................ucccugagacccuaacuugGga.....................................................................	16	1	seq
...................uccUugagacccuaacuuguga.....................................................................	3	1	seq
...................ucccugagacUcuaacuuguga.....................................................................	1942	1	seq
...................ucccugagacccuaacuugugG.....................................................................	3977	1	seq
...................uAccugagacccuaacuuguga.....................................................................	2	1	seq
...................ucccCgagacccuaacuuguga.....................................................................	5	1	seq
...................ucccugagaAccuaacuuguga.....................................................................	4	1	seq
...................ucccugagCcccuaacuuguga.....................................................................	1	1	seq
...................ucccugagacccuaacuugCga.....................................................................	6	1	seq
...................ucccugCgacccuaacuuguga.....................................................................	1	1	seq
...................ucGcugagacccuaacuuguga.....................................................................	2	1	seq
...................Gcccugagacccuaacuuguga.....................................................................	17	1	seq
...................uccAugagacccuaacuuguga.....................................................................	4	1	seq
...................ucccugagacccuaacuugugU.....................................................................	2776	1	seq
...................ucccugagGcccuaacuuguga.....................................................................	5	1	seq
...................ucccugagacccuaacuuguAa.....................................................................	343	1	seq
...................ucccugagUcccuaacuuguga.....................................................................	2	1	seq
...................ucccugagaUccuaacuuguga.....................................................................	3	1	seq
...................ucccugagacccuaacuugAga.....................................................................	4	1	seq
...................ucccugagacccuaacAuguga.....................................................................	1	1	seq
...................ucccugagacccuaaAuuguga.....................................................................	3	1	seq
...................ucccuCagacccuaacuuguga.....................................................................	1	1	seq
...................ucccugGgacccuaacuuguga.....................................................................	3	1	seq
```

Mature **Star**

```
guuguugcgccccucucaaucccugagacccuaacuugugauguuuagcuuuuaaauccacggguuaggcucuugggagcugugaguugugcuuugacaucuucauuuua
...................ucccugagacccuaacCuguga.....................................................................	4	1	seq
...................ucccugagacccuaacuuCuga.....................................................................	3	1	seq
...................ucccugagacccCaacuuguga.....................................................................	5	1	seq
...................uUccugagacccuaacuuguga.....................................................................	13	1	seq
...................ucccugagacccuaGcuuguga.....................................................................	3	1	seq
...................ucccugagacccuGacuuguga.....................................................................	4	1	seq
...................Acccugagacccuaacuuguga.....................................................................	17	1	seq
...................ucccugagacccuaacuuguCa.....................................................................	3	1	seq
...................ucccugagacccuaUcuuguga.....................................................................	2	1	seq
...................ucccugagaccAuaacuuguga.....................................................................	8	1	seq
...................ucccugaUacccuaacuuguga.....................................................................	4	1	seq
...................ucccAgagacccuaacuuguga.....................................................................	3	1	seq
...................ucccugagaccUuaacuuguga.....................................................................	24	1	seq
...................ucccugaAacccuaacuuguga.....................................................................	1	1	seq
...................uccGugagacccuaacuuguga.....................................................................	2	1	seq
...................ucccugagacccuaaUuuguga.....................................................................	3	1	seq
...................Ccccugagacccuaacuuguga.....................................................................	9	1	seq
...................ucccugagacccuaacuuUuga.....................................................................	6	1	seq
...................ucUcugagacccuaacuuguga.....................................................................	1	1	seq
...................uGccugagacccuaacuuguga.....................................................................	1	1	seq
...................ucAcugagacccuaacuuguga.....................................................................	1	1	seq
...................ucccuAagacccuaacuuguga.....................................................................	4	1	seq
...................ucccugagacccuaacuugugC.....................................................................	98	1	seq
...................ucccugagacccuaacuuguUa.....................................................................	10	1	seq
...................ucccugagacccuaacuugugaA....................................................................	23916	1	seq
...................ucccugagacccuaacuuAugau....................................................................	1	1	seq
...................ucccugagacccuaacuugugUu....................................................................	33	1	seq
...................ucccugagacccuaacuugugCu....................................................................	2	1	seq
...................ucccugagacccuaacuuguAau....................................................................	9	1	seq
...................ucccugagacccuaacuugugau....................................................................	1482	0	seq
...................ucccugagacccuaacuuCugau....................................................................	1	1	seq
...................ucccugagacccuaacAugugau....................................................................	1	1	seq
...................ucccugagacccuaacuugGgau....................................................................	1	1	seq
...................ucccuAagacccuaacuugugau....................................................................	1	1	seq
...................ucccugagacccuaacuugCgau....................................................................	1	1	seq
...................ucccugagacccuaaUuugugau....................................................................	1	1	seq
...................ucccugagaccUuaacuugugau....................................................................	3	1	seq
...................ucccugagacccuaacuugugGu....................................................................	9	1	seq
...................ucccugagacUcuaacuugugau....................................................................	11	1	seq
...................ucccugaCacccuaacuugugau....................................................................	1	1	seq
...................ucccugagacccuaacuuguUau....................................................................	1	1	seq
...................uccAugagacccuaacuugugau....................................................................	1	1	seq
...................ucccugagacccuaacuugugaug...................................................................	5	0	seq
...................ucccugagacccuaacuugugauU...................................................................	51	1	seq
...................ucccugagacccuaacuugugauC...................................................................	2	1	seq
...................ucccugagacccuaacuugugaAg...................................................................	154	1	seq
...................ucccugagacccuaacuugugauA...................................................................	70	1	seq
...................ucccugagacccuaacuugugaugu..................................................................	3	0	seq
...................ucccugagacccuaacuugugaAgu..................................................................	3	1	seq
...................ucccugagacccuaacuugugauAu..................................................................	5	1	seq
...................ucccugagacccuaacuugugauguu.................................................................	1	0	seq
...................ucccugagacccuaacuugugauguuu................................................................	1	0	seq
...................ucccugagacccuaacuugugauguuua...............................................................	2	0	seq
....................cccugagacccuaacuGgu.......................................................................	1	1	seq
....................cccugagacccuaacuugA.......................................................................	1	1	seq
....................cccugagCcccuaacuugu.......................................................................	1	1	seq
....................Uccugagacccuaacuugu.......................................................................	1	1	seq
....................cccugagacUcuaacuugu.......................................................................	2	1	seq
....................cccuUagacccuaacuuguga.....................................................................	1	1	seq
....................cccugagacccuaaUuuguga.....................................................................	2	1	seq
....................Accugagacccuaacuuguga.....................................................................	1	1	seq
....................Gccugagacccuaacuuguga.....................................................................	2	1	seq
....................cccugagacccuaacuugugG.....................................................................	209	1	seq
....................cccugagacccuaacuugugU.....................................................................	5	1	seq
....................cccugagacccuaacCuguga.....................................................................	1	1	seq
....................Uccugagacccuaacuuguga.....................................................................	3	1	seq
....................cccuCagacccuaacuuguga.....................................................................	1	1	seq
....................cccugagaccUuaacuuguga.....................................................................	2	1	seq
....................cccugagacccAaacuuguga.....................................................................	1	1	seq
....................cccugagacUcuaacuuguga.....................................................................	30	1	seq
```

Mature **Star**

```
guuguugcgccccucucaaucccugagacccuaacuugugauguuuagcuuuuaaauccacggguuaggcucuugggagcugugaguugugcuuugacaucuucauuuua
....................cccugagacUcuaacuugugau....................................................................  1    1  seq
....................cccugagacccuaacuugugau....................................................................  406  0  seq
....................Accugagacccuaacuugugau....................................................................  1    1  seq
....................cccugagacccuaacuugugaA....................................................................  959  1  seq
....................cccugagacccuaacuugugauU...................................................................  5    1  seq
....................cccugagacccuaacuugugaAg...................................................................  10   1  seq
....................cccugagacccuaacuugugaug...................................................................  2    0  seq
....................cccugagacccuaacuugugauA...................................................................  49   1  seq
.....................ccugagacccuaacuuguAa.....................................................................  1    1  seq
.....................ccugagacccuaacuugugG.....................................................................  7    1  seq
.....................ccugagacccuaacuugugU.....................................................................  1    1  seq
.....................ccugagacUcuaacuuguga.....................................................................  1    1  seq
.....................ccugagacccuaacuugugaA....................................................................  11   1  seq
.....................ccugagacccuaacuugugau....................................................................  2    0  seq
......................cugagacccuaacuugugG.....................................................................  1    1  seq
......................cugagacccuaacuugugaA....................................................................  2    1  seq
.......................ugagacccuaacuugugU.....................................................................  1    1  seq
.......................ugagacccuaacuugugG.....................................................................  7    1  seq
.......................ugagacccuaacuugugaA....................................................................  6    1  seq
........................gagacccuaacuugugau....................................................................  1    0  seq
..........................................................cacggguuaggcucuuggg.................................  1    0  seq
..........................................................cacggguuaggcucuuggga................................  8    0  seq
..........................................................cacggguuaggcucuugggag...............................  7    0  seq
..........................................................cacggguuaggcucuugggaU...............................  1    1  seq
..........................................................cacggguuaggcucuugggaA...............................  7    1  seq
..........................................................cacggguuaggcucuugggagc..............................  21   0  seq
..........................................................cacggguuaggcucuugggagU..............................  17   1  seq
..........................................................cacggguuaggcucuugggagA..............................  7    1  seq
..........................................................cacggguuaggcucuugggagUu.............................  1    1  seq
..........................................................cacggguuaggcucuugggagcu.............................  3    0  seq
..........................................................cacggguuaggcucuugggagAu.............................  1    1  seq
..........................................................cacggguuaggcucuugggagcuU............................  2    1  seq
...........................................................acggguuaggcucuuggga................................  6    0  seq
...........................................................acggguuaggcucuugggaU...............................  1    1  seq
...........................................................acggguuaggcucuugggag...............................  30   0  seq
...........................................................acggguuaggcucuugggaA...............................  19   1  seq
...........................................................acggguuaggcucuugggagA..............................  10   1  seq
...........................................................acggguuaggcucuugggagc..............................  76   0  seq
...........................................................acggguuaggcucuugggagU..............................  10   1  seq
...........................................................acggguuaggcucuugggagUu.............................  2    1  seq
...........................................................acggguuaggcucuugggagcA.............................  17   1  seq
...........................................................acgUguuaggcucuugggagcu.............................  1    1  seq
...........................................................acUgguuaggcucuugggagcu.............................  1    1  seq
...........................................................acggguuaggcucuugggagcu.............................  784  0  seq
...........................................................Ucggguuaggcucuugggagcu.............................  1    1  seq
...........................................................acggguuaggcucuugggagcG.............................  8    1  seq
...........................................................acggguuaggcucuugggagcuC............................  2    1  seq
...........................................................acggguuaggcucuugggagcuA............................  6    1  seq
...........................................................acggguuaggcucuugggagcuU............................  96   1  seq
...........................................................acggguuaggcucuugggagcuUu...........................  8    1  seq
...........................................................acggguuaggcucuugggagcuCu...........................  1    1  seq
............................................................cggguuaggcucuugggag...............................  1    0  seq
............................................................cggguuaggcucuugggagcu.............................  2    0  seq
............................................................cggguuaggcucuugggagcuU............................  1    1  seq
```

Provisional ID : mun-miR-181a-1
Score total : 90023.7
Score for star read(s) : 3.9
Score for read counts : 90014.3
Score for mfe : 0.9
Score for randfold : 1.6
Score for cons. seed : 3
Total read count : 176571
Mature read count : 175555
Loop read count : 0
Star read count : 1016

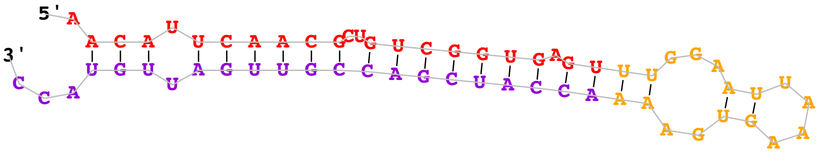

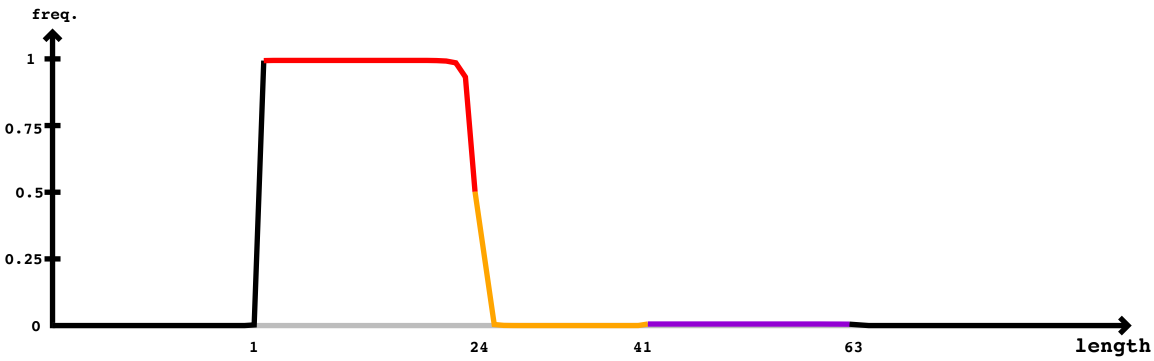


```
                    Mature                                  Star
5'- uuguagugguugcuucagugaacauucaacgcugucggugaguuuggaauuaaagugaaaaccaucgaccguugauuguacccuccagcuaaccauccuccuccuuacuu   -3'   obs
    uuguagugguugcuucagugaacauucaacgcugucggugaguuuggaauuaaagugaaaaccaucgaccguugauuguacccuccagcuaaccauccuccuccuuacuu         exp
    .....((((((((..((.(.(((.((((((..(((((((.((((...((....))..))))))))))))))))).))).).))..))).))))))................    reads    mm    sample
    ..................uCaacauucaacgcugucggugag....................................................................    1        1     seq
    ..................uCaacauucaacgcugucggugagu...................................................................    1        1     seq
    ..................Ggaacauucaacgcugucggugagu...................................................................    1        1     seq
    ...................gaacauucaacgcugucg.........................................................................    1        0     seq
    ...................gaacauucaacgcugucggu.......................................................................    1        0     seq
    ...................Caacauucaacgcugucggug......................................................................    1        1     seq
    ...................gaacauucaacgcugucggug......................................................................    10       0     seq
    ...................gaacaCucaacgcugucgguga.....................................................................    1        1     seq
    ...................gaacauucaacgcugucggugG.....................................................................    17       1     seq
    ...................gaacauucaacgcugucggugU.....................................................................    12       1     seq
    ...................gaacauucaacgcugucgguga.....................................................................    85       0     seq
    ...................gaacauucaacgcugucggugaA....................................................................    2        1     seq
    ...................gaacauucaacgcugucggAgag....................................................................    1        1     seq
    ...................Caacauucaacgcugucggugag....................................................................    6        1     seq
    ...................gaacauucaacgcugucggugaU....................................................................    6        1     seq
    ...................gaacauucaacgcugucggugag....................................................................    230      0     seq
    ...................gaacaCucaacgcugucggugag....................................................................    1        1     seq
    ...................gaacauucaacgcugucggGgag....................................................................    1        1     seq
    ...................gaacauucaacgcugucggugagu...................................................................    42       0     seq
    ...................Caacauucaacgcugucggugagu...................................................................    5        1     seq
    ...................gaacauucaacgcugucggugaguuuU................................................................    1        1     seq
    ....................aacauucaacgcugucgg........................................................................    109      0     seq
    ....................aacauucaacgcugucgU........................................................................    1        1     seq
    ....................aacauucaacgcugucgA........................................................................    1        1     seq
    ....................aacauucaacgcugucggu.......................................................................    286      0     seq
    ....................aacauAcaacgcugucggu.......................................................................    1        1     seq
    ....................aacauucaacgcugGcggu.......................................................................    1        1     seq
    ....................aacauucaacgAugucggu.......................................................................    1        1     seq
    ....................aacauucaacgcugucggA.......................................................................    2        1     seq
    ....................aacauucaacgcugucgCu.......................................................................    2        1     seq
    ....................aacauucaacgcugucggG.......................................................................    3        1     seq
    ....................aacauuUaacgcugucggu.......................................................................    1        1     seq
    ....................aacauucaaUgcugucggu.......................................................................    1        1     seq
```

```
                    Mature                                  Star
uuguagugguugcuucagugaacauucaacgcugucggugaguuuggaauuaaagugaaaaccaucgaccguugauuguacccuccagcuaaccauccuccuccuuacuu
....................aacauucaacgcugucggCg......................................................................  5      1  seq
....................aacauucaacgcuCucggug......................................................................  1      1  seq
....................aacauucaacgcugAcggug......................................................................  1      1  seq
....................aacauucaacgcugucggug......................................................................  1125   0  seq
....................aacCuucaacgcugucggug......................................................................  1      1  seq
....................aacauucaacgcugucggAg......................................................................  2      1  seq
....................aacauucaacgcugucgCug......................................................................  1      1  seq
....................aacauucaacCcugucggug......................................................................  1      1  seq
....................aacauucaacgAugucggug......................................................................  1      1  seq
....................aacauucaacgcuUucggug......................................................................  1      1  seq
....................aacauucaacgcugucggGg......................................................................  9      1  seq
....................aacauucaacgcugGcggug......................................................................  2      1  seq
....................aacauucaacgcugucgguU......................................................................  7      1  seq
....................aaAauucaacgcugucggug......................................................................  1      1  seq
....................aacauucaacgcugucgguA......................................................................  14     1  seq
....................aacGuucaacgcugucggug......................................................................  1      1  seq
....................aacauucGacgcugucggug......................................................................  1      1  seq
....................aacauucaacgUugucggug......................................................................  3      1  seq
....................aacGuucaacgcugucgguga.....................................................................  2      1  seq
....................aacauucaaUgcugucgguga.....................................................................  2      1  seq
....................aacauucaacgcugucgUuga.....................................................................  2      1  seq
....................Uacauucaacgcugucgguga.....................................................................  1      1  seq
....................aacauuUaacgcugucgguga.....................................................................  2      1  seq
....................aacauucaacgcugucgguUa.....................................................................  1      1  seq
....................aacauucaacgcugucUguga.....................................................................  2      1  seq
....................aacauucaacgcugucggCga.....................................................................  6      1  seq
....................Gacauucaacgcugucgguga.....................................................................  5      1  seq
....................aacauucaacgcugCcgguga.....................................................................  1      1  seq
....................aacauucaacgUugucgguga.....................................................................  15     1  seq
....................aacauucaacgGugucgguga.....................................................................  1      1  seq
....................aacauucaacgcCgucgguga.....................................................................  1      1  seq
....................aacauucaacgcugucggAga.....................................................................  6      1  seq
....................aacauucaacgcugucggGga.....................................................................  28     1  seq
....................aacauucaacgcugucgAuga.....................................................................  3      1  seq
....................aaAauucaacgcugucgguga.....................................................................  1      1  seq
....................aacauucaacUcugucgguga.....................................................................  2      1  seq
....................aacauucaacgcugucggugU.....................................................................  1406   1  seq
....................aacauucaacgcugAcgguga.....................................................................  2      1  seq
....................Cacauucaacgcugucgguga.....................................................................  1      1  seq
....................aacauucaacgcugucgCuga.....................................................................  1      1  seq
....................aacauucaaAgcugucgguga.....................................................................  1      1  seq
....................aacauucaacgcugucgguga.....................................................................  6698   0  seq
....................aaUauucaacgcugucgguga.....................................................................  1      1  seq
....................aacauucaacAcugucgguga.....................................................................  2      1  seq
....................aacauucaacgcugucggugG.....................................................................  1035   1  seq
....................aacauucaacgcugucAguga.....................................................................  2      1  seq
....................aacauCcaacgcugucgguga.....................................................................  1      1  seq
....................aacauucaacgcuguAgguga.....................................................................  3      1  seq
....................aacauucaacgcugGcgguga.....................................................................  2      1  seq
....................aacaCucaacgcugucgguga.....................................................................  2      1  seq
....................aacauucaacgcuguUgguga.....................................................................  2      1  seq
....................aacauucaCcgcugucgguga.....................................................................  1      1  seq
....................aacauucaacCcugucgguga.....................................................................  2      1  seq
....................aacauucaacgcugucgguAa.....................................................................  20     1  seq
....................aacauucaacgcuCucggugag....................................................................  9      1  seq
....................aacaCucaacgcugucggugag....................................................................  8      1  seq
....................aacauucaacgcugAcggugag....................................................................  5      1  seq
....................aacauAcaacgcugucggugag....................................................................  3      1  seq
....................aacauucaacgcugGcggugag....................................................................  6      1  seq
....................aacauucaaGgcugucggugag....................................................................  4      1  seq
....................Uacauucaacgcugucggugag....................................................................  15     1  seq
....................aacauucaacgcuUucggugag....................................................................  16     1  seq
....................aacauucaacgcugucggugag....................................................................  72685  0  seq
....................Cacauucaacgcugucggugag....................................................................  13     1  seq
....................aacauucaacgcuguGggugag....................................................................  4      1  seq
....................aacauucUacgcugucggugag....................................................................  1      1  seq
....................aacauucaacUcugucggugag....................................................................  9      1  seq
....................aGcauucaacgcugucggugag....................................................................  5      1  seq
....................aacauucaacgcuguAggugag....................................................................  9      1  seq
....................aacauucaacgcugucggugUg....................................................................  8      1  seq
```

```
                    Mature                                    Star
uuguagugguugcuucagugaacauucaacgcugucggugaguuuggaauuaaagugaaaaccaucgaccguugauuguacccuccagcuaaccauccuccuccuuacuu
....................aaUauucaacgcugucggugag....................................................................  4     1  seq
....................aacauucaacgAugucggugag....................................................................  13    1  seq
....................aacauucGacgcugucggugag....................................................................  14    1  seq
....................aacauucaGcgcugucggugag....................................................................  4     1  seq
....................aacUuucaacgcugucggugag....................................................................  3     1  seq
....................aacauucaacgcAgucggugag....................................................................  1     1  seq
....................aacaGucaacgcugucggugag....................................................................  1     1  seq
....................aacauuAaacgcugucggugag....................................................................  9     1  seq
....................aaAauucaacgcugucggugag....................................................................  8     1  seq
....................aacauucaacgcugucggugaU....................................................................  986   1  seq
....................aacauucaacgcugucgCugag....................................................................  2     1  seq
....................aacauucaacgcugucAgugag....................................................................  10    1  seq
....................aacauucaacgcugucggugaA....................................................................  1260  1  seq
....................aacauucaacgcugucgguAag....................................................................  4     1  seq
....................aacauucaaAgcugucggugag....................................................................  20    1  seq
....................aacauucaacgcugucgUugag....................................................................  19    1  seq
....................aacaAucaacgcugucggugag....................................................................  2     1  seq
....................aacauucCacgcugucggugag....................................................................  1     1  seq
....................aacauucaacgcuguUggugag....................................................................  14    1  seq
....................aacauucaaUgcugucggugag....................................................................  9     1  seq
....................aacauucaacgcugucggGgag....................................................................  118   1  seq
....................aaGauucaacgcugucggugag....................................................................  2     1  seq
....................aacauuGaacgcugucggugag....................................................................  4     1  seq
....................aacauucaacgUugucggugag....................................................................  60    1  seq
....................aacauucaacgcugucggugaC....................................................................  24    1  seq
....................aacauucaUcgcugucggugag....................................................................  1     1  seq
....................aacGuucaacgcugucggugag....................................................................  10    1  seq
....................aacauuUaacgcugucggugag....................................................................  7     1  seq
....................aacauucaacgcugucgguCag....................................................................  3     1  seq
....................aacauucaacgcugucggAgag....................................................................  24    1  seq
....................aacauucaacAcugucggugag....................................................................  8     1  seq
....................aacauucaacgcugucUgugag....................................................................  9     1  seq
....................aacauucaacgcCgucggugag....................................................................  8     1  seq
....................aacauucaacgcugucgAugag....................................................................  4     1  seq
....................aacauucaacgcugucCgugag....................................................................  3     1  seq
....................aacauucaacCcugucggugag....................................................................  6     1  seq
....................aacauucaacgcuAucggugag....................................................................  8     1  seq
....................Gacauucaacgcugucggugag....................................................................  66    1  seq
....................aacauucaacgcugucgguUag....................................................................  12    1  seq
....................aacauucaCcgcugucggugag....................................................................  2     1  seq
....................aUcauucaacgcugucggugag....................................................................  8     1  seq
....................aacauucaacgcugucggugGg....................................................................  72    1  seq
....................aacauucaacgcugucggCgag....................................................................  38    1  seq
....................aacauucaacgcugCcggugag....................................................................  4     1  seq
....................aacauCcaacgcugucggugag....................................................................  4     1  seq
....................aacauucaacgcGgucggugag....................................................................  1     1  seq
....................aacauucaacAcugucggugagu...................................................................  17    1  seq
....................aacauucaacgcugucggCgagu...................................................................  41    1  seq
....................aacauucaacgcugucggugGgu...................................................................  11    1  seq
....................aacauucaacgcugucggAgagu...................................................................  31    1  seq
....................aacauucaacgAugucggugagu...................................................................  11    1  seq
....................aGcauucaacgcugucggugagu...................................................................  6     1  seq
....................aacauucaacgcCgucggugagu...................................................................  8     1  seq
....................aacauucaacgcugucggugUgu...................................................................  6     1  seq
....................aacauucaacgcuguAggugagu...................................................................  7     1  seq
....................aacauucaGcgcugucggugagu...................................................................  9     1  seq
....................aacauucaacgcugucggGgagu...................................................................  120   1  seq
....................aacauucaacgcuguGggugagu...................................................................  4     1  seq
....................aacauucaacgcuCucggugagu...................................................................  7     1  seq
....................aacauucaacgcugucggugagC...................................................................  22    1  seq
....................aacGuucaacgcugucggugagu...................................................................  4     1  seq
....................aCcauucaacgcugucggugagu...................................................................  1     1  seq
....................aacauucaacgcAgucggugagu...................................................................  5     1  seq
....................aacauucUacgcugucggugagu...................................................................  1     1  seq
....................aacauucaacgcuguUggugagu...................................................................  16    1  seq
....................aacauuUaacgcugucggugagu...................................................................  9     1  seq
....................aacauucaacgcugucggugaUu...................................................................  25    1  seq
....................aacCuucaacgcugucggugagu...................................................................  1     1  seq
....................aacauucaacgcugCcggugagu...................................................................  10    1  seq
....................aacauucaacgGugucggugagu...................................................................  1     1  seq
```

```
                    Mature                                    Star
uuguagugguugcuucagugaacauucaacgcugucggugaguuuggaauuaaagugaaaaccaucgaccguugauuguacccuccagcuaaccauccuccuccuuacuu
....................aacauucaaUgcugucggugagu...................................................................   15      1   seq
....................aacauucaacgcugucggugaCu...................................................................   3       1   seq
....................aacauucaacgcugucgAugagu...................................................................   3       1   seq
....................Cacauucaacgcugucggugagu...................................................................   18      1   seq
....................aaGauucaacgcugucggugagu...................................................................   5       1   seq
....................aaUauucaacgcugucggugagu...................................................................   7       1   seq
....................aacauucaacgcGgucggugagu...................................................................   2       1   seq
....................aacauucaacgcugucggugagA...................................................................   3159    1   seq
....................aacauucaacgcugucggugagu...................................................................   80846   0   seq
....................aacauucaacgcugucgCugagu...................................................................   5       1   seq
....................aacauucaacCcugucggugagu...................................................................   5       1   seq
....................aacauucaacgcugucggugaAu...................................................................   79      1   seq
....................aacauucaacgcugAcggugagu...................................................................   9       1   seq
....................Uacauucaacgcugucggugagu...................................................................   19      1   seq
....................aacauucaacgcugGcggugagu...................................................................   8       1   seq
....................aacauucaacgcugucgUugagu...................................................................   11      1   seq
....................Gacauucaacgcugucggugagu...................................................................   74      1   seq
....................aacUuucaacgcugucggugagu...................................................................   3       1   seq
....................aacauCcaacgcugucggugagu...................................................................   4       1   seq
....................aacauuGaacgcugucggugagu...................................................................   6       1   seq
....................aacauucaacgcugucUgugagu...................................................................   18      1   seq
....................aacauucaacgUugucggugagu...................................................................   86      1   seq
....................aacauucaacgcuAucggugagu...................................................................   5       1   seq
....................aacaCucaacgcugucggugagu...................................................................   8       1   seq
....................aacauucaacgcugucCgugagu...................................................................   4       1   seq
....................aacauucaUcgcugucggugagu...................................................................   3       1   seq
....................aacauucGacgcugucggugagu...................................................................   14      1   seq
....................aacaGucaacgcugucggugagu...................................................................   1       1   seq
....................aaAauucaacgcugucggugagu...................................................................   7       1   seq
....................aacauAcaacgcugucggugagu...................................................................   6       1   seq
....................aacauucaacgcugucggugagG...................................................................   126     1   seq
....................aacauuAaacgcugucggugagu...................................................................   12      1   seq
....................aacauucaacgcugucggugCgu...................................................................   2       1   seq
....................aacauucaacgcugucAgugagu...................................................................   8       1   seq
....................aacauucaacgcugucgguAagu...................................................................   4       1   seq
....................aUcauucaacgcugucggugagu...................................................................   4       1   seq
....................aacauucaacgcugucgguUagu...................................................................   12      1   seq
....................aacauucaacUcugucggugagu...................................................................   11      1   seq
....................aacauucaaAgcugucggugagu...................................................................   10      1   seq
....................aacauucaacgcuUucggugagu...................................................................   14      1   seq
....................aaUauucaacgcugucggugaguu..................................................................   1       1   seq
....................aacGuucaacgcugucggugaguu..................................................................   1       1   seq
....................aacauucaacgcugucggugaguG..................................................................   7       1   seq
....................aacauucCacgcugucggugaguu..................................................................   1       1   seq
....................Cacauucaacgcugucggugaguu..................................................................   1       1   seq
....................aacauucaacgcugucggGgaguu..................................................................   4       1   seq
....................aacauucaacgcugucggugaguA..................................................................   46      1   seq
....................aacauucaacgcugucgCugaguu..................................................................   1       1   seq
....................aacauucaacgcugucggugagGu..................................................................   4       1   seq
....................aacauucaacgcugucgguCaguu..................................................................   1       1   seq
....................aacauucaacgcugucggCgaguu..................................................................   2       1   seq
....................aacauucaacgcugucggugagCu..................................................................   1       1   seq
....................aacauucaacUcugucggugaguu..................................................................   1       1   seq
....................aacauucaacgcugucggugaguu..................................................................   2659    0   seq
....................aacauucaacgcuguAggugaguu..................................................................   1       1   seq
....................aacauucaacgcugucggugaguC..................................................................   2       1   seq
....................aacauucaacgcugucggugaAuu..................................................................   1       1   seq
....................aacauucaacgcugucggugagAu..................................................................   11      1   seq
....................aacauucaacAcugucggugaguu..................................................................   1       1   seq
....................aacauucaacgcugucggugCguu..................................................................   1       1   seq
....................Gacauucaacgcugucggugaguu..................................................................   2       1   seq
....................aacauucaaAgcugucggugaguu..................................................................   1       1   seq
....................aacauucaacgcugucggugGguu..................................................................   1       1   seq
....................aacauucaacgcugucggugUguu..................................................................   1       1   seq
....................aacauucaacgcuguUggugaguu..................................................................   1       1   seq
....................aacauucaacgUugucggugaguu..................................................................   1       1   seq
....................aacauucaacgcuCucggugaguu..................................................................   1       1   seq
....................aacauucaacgcugCcggugaguu..................................................................   1       1   seq
....................aacauucaacgcuAucggugaguuu.................................................................   1       1   seq
....................aacGuucaacgcugucggugaguuu.................................................................   1       1   seq
```

Mature Star

```
uuguagugguugcuucagugaacauucaacgcugucggugaguuuggaauuaaagugaaaaccaucgaccguugauuguacccuccagcuaaccauccuccuccuuacuu
....................Gacauucaacgcugucggugaguuu.................................................................	1	1	seq
....................aacauucaacgcugucggGgaguuu.................................................................	1	1	seq
....................aacauucaacgcugucggugaguuu.................................................................	576	0	seq
....................aacauucaacgcugucggugaguuG.................................................................	2	1	seq
....................aacauucaacgcugucggCgaguuu.................................................................	1	1	seq
....................aacauucaacgcugucggugaguuA.................................................................	5	1	seq
....................Cacauucaacgcugucggugaguuu.................................................................	1	1	seq
....................aacauucaacgcugucggugaguAu.................................................................	3	1	seq
....................aacauucaacgcugucggugagAuu.................................................................	1	1	seq
....................aacauucaacgcugucggugaguuGg................................................................	4	1	seq
....................aacauucaacgcugucggugaguuug................................................................	1	0	seq
....................aacauucaacgcugucggugaguuuU................................................................	97	1	seq
....................aacauucaacgcugucggugaguuuA................................................................	1	1	seq
....................aacauucaacgcugucggugaguuugU...............................................................	5	1	seq
....................aacauucaacgcugucggugaguuGgga..............................................................	1	1	seq
....................aacauucaacgcugucggugaguuuggaa.............................................................	2	0	seq
.....................acauucaacgcugucgguga.............................................................................	2	0	seq
.....................acauucaacgcugucggugag............................................................................	19	0	seq
.....................acauucaacgcugucgUugag............................................................................	1	1	seq
.....................acauucaacgcugucggugagA...................................................................	5	1	seq
.....................acauucaacgcugucggugagG...................................................................	1	1	seq
.....................acauucaacgcugucggugagu...................................................................	73	0	seq
.....................acauucaacgcugucggugaguu..................................................................	9	0	seq
.....................acauucaacgcugucggugaguuu.................................................................	4	0	seq
......................cauucaacgcugucgguga.............................................................................	1	0	seq
```
[truncated: 288,900 more chars]
